# Straightforward Donor Atom Switching: From P,C,P- to Various Y,C,Y-Pincer (Y = O, S, Se) Organotin(IV) Compounds and Cations

**DOI:** 10.1021/acs.inorgchem.5c05899

**Published:** 2026-04-21

**Authors:** Richard Chlebík, Erik Kertész, Zdeňka Růžičková, Aleš Růžička, Roman Jambor, Zoltán Benkő, Libor Dostál

**Affiliations:** † Department of General and Inorganic Chemistry, 48252 University of Pardubice, Studentská 573, Pardubice CZ 532 10, Czech Republic; ‡ Department of Inorganic and Analytical Chemistry, Faculty of Chemical Technology and Biotechnology, Budapest University of Technology and Economics, Műegyetem rkp. 3, Budapest H-1111, Hungary; § HUN-REN-BME Computation Driven Chemistry Research Group, Műegyetem rkp. 3, Budapest H-1111, Hungary

## Abstract

The oxidation of phosphorus atoms in *P,C,P*-pincer organotin­(IV) compounds ArSnCl_3_ (**1**), ArSnPhCl_2_ (**2**) and ArSnPh_2_Cl (**3**) (Ar = 2,6-(*t*Bu_2_PO)_2_C_6_H_3_) with Me_3_NO (or H_2_O_2_), S or Se furnished compounds Ar^Y^SnCl_3_ (**1** ^**Y**^), Ar^Y^SnPhCl_2_ (**2** ^**Y**^), and Ar^Y^SnPh_2_Cl (**3** ^**Y**^) (Ar^Y^ **=** 2,6-(*t*Bu_2_(Y)­PO)_2_C_6_H_3_; Y = O, S or Se, except **3** ^**O**^). Their reactions with Na­[BArF] ([BArF] = [3,5-(CF_3_)_2_C_6_H_3_]_4_B) afforded salts containing organotin cations [Ar^Y^SnCl_2_]­[BArF] (**1** ^**Y+**^ **[BArF]** ^**–**^), [Ar^Y^SnPhCl]­[BArF] (**2** ^**Y+**^ **[BArF]** ^**–**^), and [Ar^Y^SnPh_2_]­[BArF] (**3** ^**Y+**^ **[BArF]** ^**–**^). The oxidation of compound **3** with 1 equiv of Me_3_NO gave compound [Ar^PO^SnPh_2_Cl] (**3** ^**PO**^) (Ar^PO^ = 2-(*t*Bu_2_PO)-6-(*t*Bu_2_(O)­PO)­C_6_H_3_) and its subsequent oxidation by S or Se allowed the isolation of mixed donor compounds [Ar^OY^SnPh_2_Cl] (**3** ^**OY**^) (Ar^OY^ = 2-(*t*Bu_2_(O)­PO)-6-(*t*Bu_2_(Y)­PO)­C_6_H_3_; Y = S or Se). Compounds **3** ^**PO**^ and **3** ^**OY**^ were converted to salts [Ar^PO^SnPh_2_]­[BArF] (**3** ^**PO+**^ **[BArF]** ^**–**^) and [Ar^OY^SnPh_2_]­[BArF] (**3** ^**OY+**^ **[BArF]** ^**–**^; Y = S or Se). All compounds were characterized by multinuclear NMR spectroscopy in solution, and their molecular structures (except **3** ^**OS**^) were determined by single crystal (*sc*) X-ray diffraction analysis. The intramolecular P/Y → Sn interactions and their influence on the structures of complexes were analyzed in detail using NMR data and solid-state structures. The experiments were corroborated by a DFT study including NBO and AIM-analysis, which reveals the role of chalcogen centers in tuning the thermodynamic stabilization in the pincer complexes.

## Graphical abstract

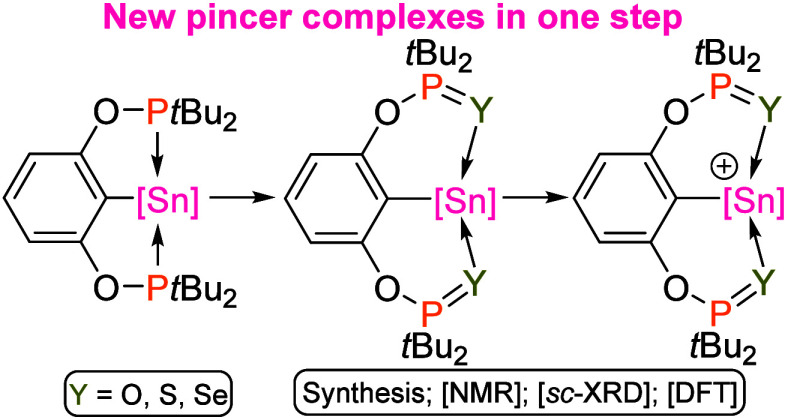

## Introduction

The classical van Koten’s monoanionic-*Y,C,Y*-pincer ligands^1–3^ found a widespread utilization in the field of organotin compounds, and various 2*e*-donating Y groups were involved in the development of this area over decades.^4–6^ Original studies relied on the utilization of *N,C,N*- chelating ligands, but they were shortly followed by *O,C,O*-, *O,C,S-* and *N,C,O*- analogues (Scheme 1A).^7–15^ The early studies mostly focused on the relationship between the tin coordination preferences, its Lewis acidity, and structure of the ligand. Nevertheless, more spectacular compounds with unique structures, interesting properties, and reactivity appeared in the literature. To mention a few examples, *N,C,N*-organotin­(IV) compounds were at the birth of, to that time unknown, heteroboroxines^16–18^ (Scheme 1B). The first example of thermodynamically stabilized distannyne was isolated using a bis­(amino)-*N,C,N*-pincer ligand^19^ revealing predominantly Sn–Sn single bond (Scheme 1C) and related group 14 counterparts^20–24^ have recently been introduced. These compounds exhibited interesting reactivity toward various substrates.^22,24–27^ On the contrary, the reduction of bis­(imino)-*N,C,N*-pincer tin­(II) compounds provided, in addition to the expected distannyne, a unique tin-containing aromatic system (Scheme 1D).^28^ It is noteworthy that hydridotetrylenes could be stabilized by pincer-ligands,^29,30^ while some of them exhibit remarkable coordination flexibility via redox hydrogen shuttling.^30^ Corresponding chlorostannylenes have been recognized as useful ligands for transition metals.^31–35^

**Scheme 1:**
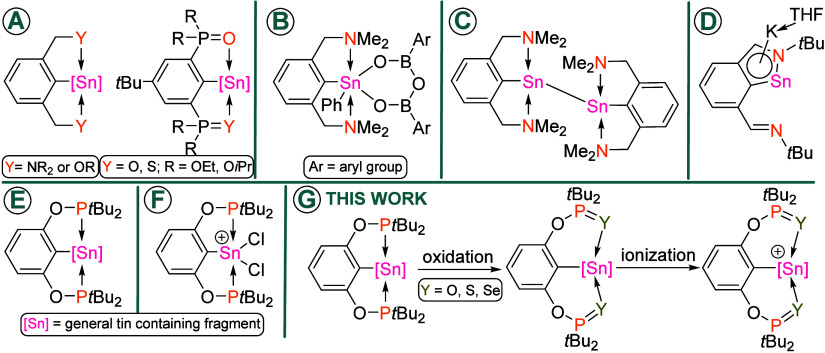
Various *Y,C,Y*-Ligands Used in the Realm of Tin Chemistry (**A**); Selected Examples of Their Applications (**B**–**D**); Recently Introduced *P,C,P*-Ligand for Tin Atom Coordination (**E**, **F**); Outline of the Present Study (**G**)

Despite the undisputed utility of pincer compounds, containing tin atoms in various oxidation states and bonding situations, this research remained, for a long time, focused mainly on ligands armed with nitrogen donor groups. We have recently demonstrated that *P,C,P*-pincer ligand (Ar = 2,6-(*t*Bu_2_PO)_2_C_6_H_3_; Scheme 1E),^36^ being otherwise very well established in the chemistry of transition metals,^37–40^ serves as a promising platform for organotin chemistry. This combination allowed, among others, the isolation of aryldichlorotin­(IV) cations (Scheme 1F), which were not accessible with *N,C,N-* or *O,C,O*-ligands,^41–45^ underlining remarkable potential of this ligand in the realm of tin chemistry.

To further develop this emerging area, here we examine the oxidation of *t*Bu_2_P groups in organotin­(IV) *P,C,P*-pincer compounds^36^ aiming at isolation of the corresponding *Y,C,Y*-pincer complexes (Y = O, S or Se) equipped with *t*Bu_2_P=Y functions using H_2_O_2_ (or Me_3_NO), S and Se as oxidizing agents (Scheme 1G). This synthetic protocol represents a straightforward and convenient way toward entirely new pincer ligands that remain even unused in transition metal coordination chemistry. Furthermore, this approach results in the switch of 5-membered to more flexible 6-membered chelates, while the influence of this change on the coordination preferences of the tin atom is elucidated. Furthermore, all new compounds were converted to corresponding *Y,C,Y*-chelated tin­(IV) cations to extend the scope of this study and to follow the influence of increasing tin atom Lewis acidity. In this work, a detailed study including *sc* X-ray diffraction analysis, multinuclear solution NMR spectroscopy and DFT investigation on target compounds is delivered.

## Results and Discussion

### Synthesis

The compounds ArSnCl_3_ (**1**), ArSnPhCl_2_ (**2**), and ArSnPh_2_Cl (**3**) (Ar = 2,6-(*t*Bu_2_PO)_2_C_6_H_3_) were prepared according to the literature procedures.^36^ Their oxidation with sulfur (2 equiv) or selenium (5 equiv, used in excess) cleanly provided compounds Ar^Y^SnCl_3_ (**1** ^**Y**^), Ar^Y^SnPhCl_2_ (**2** ^**Y**^), and Ar^Y^SnPh_2_Cl (**3** ^**Y**^) (Ar^Y^ **=** 2,6-(*t*Bu_2_(Y)­PO)_2_C_6_H_3_; Y = S or Se; Scheme 2). In the case of **1** and **2**, the use of H_2_O_2_ (30% water solution) as an oxidant in the presence of activated molecular sieves, to avoid the hydrolysis of the ligand, resulted in the expected compounds **1** ^**O**^ and **2** ^**O**^. However, the same procedure turned out to be useless for the synthesis of compound **3** ^**O**^ and always delivered only mixtures of decomposition products. Therefore, Me_3_NO was used as a water-free alternative and its reaction with **3** (1:1 ratio) furnished complex [Ar^PO^SnPh_2_Cl] (**3** ^**PO**^) (Ar^PO^ = 2-(*t*Bu_2_PO)-6-(*t*Bu_2_(O)­PO)­C_6_H_3_) with one oxidized phosphorus atom (Scheme 2), but an addition of a second equiv of Me_3_NO resulted only in substrate decay. It is also noteworthy that the reaction of **1** or **2** with one equiv of Me_3_NO yielded only inseparable mixture of products. However, isolated **3** ^**PO**^ could be further treated with S and Se, leading to mixed donor compounds [Ar^OY^SnPh_2_Cl] (**3** ^**OY**^) (Ar^OY^ = 2-(*t*Bu_2_(O)­PO)-6-(*t*Bu_2_(Y)­PO)­C_6_H_3_; Y = S or Se, Scheme 2).

**Scheme 2:**
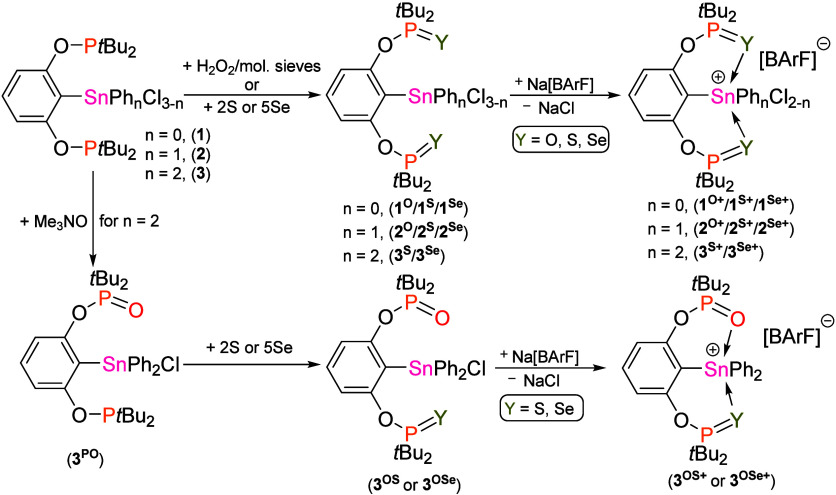
Synthesis of All the Compounds in This Study

Furthermore, all compounds mentioned above were used for the synthesis of the corresponding cations by reaction with Na­[BArF] ([BArF] = [3,5-(CF_3_)_2_C_6_H_3_]_4_B). In this way, sets of monorogano- [Ar^Y^SnCl_2_]­[BArF] (**1** ^**Y+**^ **[BArF]** ^**–**^), diorgano- [Ar^Y^SnPhCl]­[BArF] (**2** ^**Y+**^ **[BArF]** ^**–**^), and triorganotin­(IV) cations [Ar^Y^SnPh_2_]­[BArF] (**3** ^**Y+**^ **[BArF]** ^**–**^) could be obtained, while mixed donor cations [Ar^PO^SnPh_2_]­[BArF] (**3** ^**PO+**^ **[BArF]** ^**–**^) and [Ar^OY^SnPh_2_]­[BArF] (**3** ^**OY+**^ **[BArF]** ^**–**^) were also prepared (Scheme 2).

### Molecular Structures

The molecular structures of all new compounds (except for **1** ^**OS**^) were determined by *sc* X-ray diffraction analysis, the crystallographic data, and presentation of all of the structures along with selected structural parameters are given in SI, while generalized structures for each set of compounds are presented in Figures 1–4.

**Figure 1:**
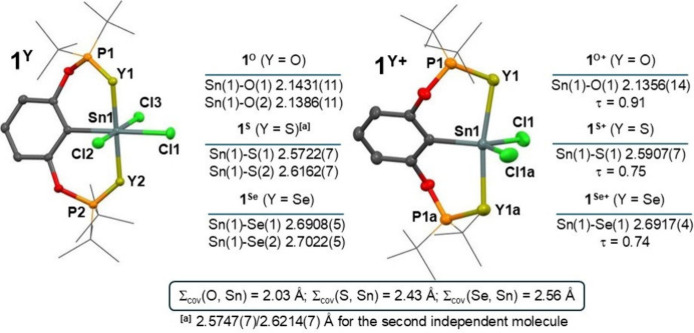
Generalized view on the molecular structures of **1** ^**Y**^ (*left*) and **1** ^**Y+**^ (*right*) along with relevant Sn–Y distances and τ values.^47^ For a detailed selection of structural parameters for all compounds, see SI. The [BArF] anion is omitted for **1** ^**Y+**^. Note: The labeling of atoms used in this figure is edited for the sake of compatibility among all compounds and does not necessarily correspond to the cif files in SI.

Both donor atoms are coordinated to the central tin atom in all monorganotin­(IV) compounds **1** ^**Y**^ (Figure 1), and observed distances Sn(1)-O­(1/2) 2.1431(11)/2.1386(11) Å; Sn(1)-S­(1/2) 2.5722(7)/2.6162(7) Å and Sn(1)-Se­(1/2) 2.6908(5)/2.7022(5) Å compare well to the Σ_cov_(Y, Sn) = 2.03 (O), 2.43 (S), 2.56 (Se) Å^46^ indicating the presence of strong intramolecular interactions leading to a distorted octahedral geometry around the central atom (Figure 1). The pincer ligand adopts a meridional coordination mode as illustrated by Y(1)–Sn(1)–Y(2) interatomic angles of 178.45(5)/161.79(3)/162.02(1)° for **1** ^**O**^/**1** ^**S**^/**1** ^**Se**^, respectively, but a slight distortion is obvious ongoing to heavier chalcogen. This gradual trend can be ascribed to the larger covalent radii of S and Se, which logically results in longer P–Y interatomic distances and consequently pushes the donor atom from ideal positions (*vide* further discussion). Interestingly, the coordination situation in **1** ^**Y**^ differs markedly from the parent **1**,^36^ where only one phosphorus atom is very tightly coordinated to the central atom (cf. Sn–P distance of 2.6313(9) Å; Σ_cov_(P, Sn) = 2.51 Å^46^) resulting in a distorted tetragonal pyramidal geometry (τ = 0.77).^36,47^ This discrepancy can be traced back to an extension of the chelate ring from 5-membered (in **1**) to 6-membered in (**1** ^**Y**^), which probably helps to accommodate the bulky *t*Bu_2_P groups around the central atom. Abstraction of one chlorine atom in **1** ^**Y+**^ resulted in a transformation into a trigonal bipyramidal array around the tin atom, being almost ideal in **1** ^**O+**^ (τ = 0.91) and a bit more distorted in **1** ^**S+**^ and **1** ^**Se+**^ (τ = 0.74 and 0.75, respectively). The axial positions are occupied by donor atoms and interatomic distances resemble those in parent **1** ^**Y**^, i.e. Sn(1)-O­(1/1a) 2.1356(14) Å (O(1)–Sn(1)–O­(1a) 177.43(5)°), Sn(1)-S­(1/1a) 2.5907(7) Å (S(1)–Sn(1)–S­(1a) 169.94(3)°) and Sn(1)-Se­(1/1a) 2.6917(4) Å (Se(1)–Sn(1)–Se­(1a) 169.61(2)°) for **1** ^**O+**^/**1** ^**S+**^/**1** ^**Se+**^, respectively.

Remarkable differences were observed in the structures of diorganotin­(IV) compounds **2** ^**Y**^ (Figure 2). In **2** ^**O**^, both oxygen atoms coordinate with the tin atom (Sn(1)-O­(1/2) 2.2003(13)/2.1919(14) Å, interatomic angle O(1)–Sn(1)–O(2) 178.15(5)°) leading to distorted octahedral coordination with both chlorine, oxygen and carbon atoms located mutually in *trans* positions. On the contrary, only one of the donor atoms is coordinated in **2** ^**S**^ and **2** ^**Se**^, while the second remains pendant, as demonstrated by fairly different distances (Sn(1)-S­(1/2) 2.8933(7)/5.8217(6) Å and Sn(1)-Se­(1/2) 2.9331(5)/6.0469(5) Å in **2** ^**S**^ and **2** ^**Se**^, respectively). This provides a five-coordinated tin atom with intermediate shape between the tetragonal pyramid and the trigonal bipyramid according to the τ values of 0.54 (**2** ^**S**^) and 0.60 (**2** ^**Se**^). The ionization of **2** ^**Y**^ resulted in an expected tight coordination of both donor atoms in **2** ^**Y+**^, while the interatomic distances approach values for covalent bonds, cf. Sn(1)-O­(1/2) 2.1601(17)/2.1537(17); Sn(1)-S­(1/2) 2.6442(9)/2.6383(8); Sn(1)-Se­(1/2) 2.7694(6)/2.7446(6) Å for **2** ^**O+**^/**2** ^**S+**^/**2** ^**Se+**^, respectively. The coordination polyhedron of the tin atom can be viewed as shifted in favor of a distorted trigonal bipyramid based on respective τ values 0.71/0.78/0.68.^47^

**Figure 2:**
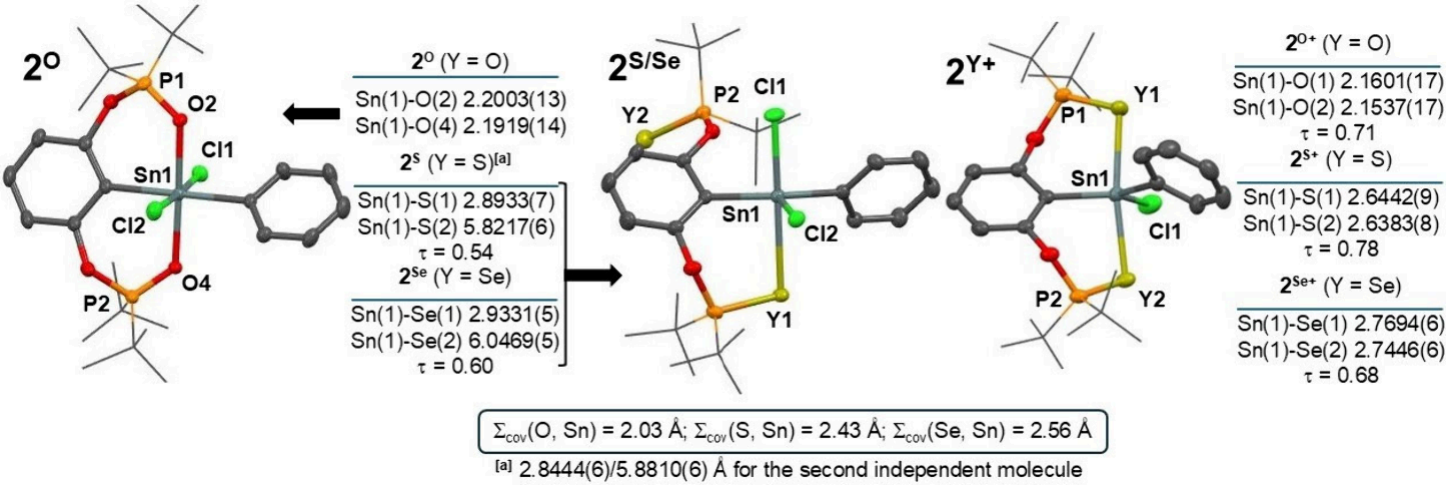
Generalized view on the molecular structures of **2** ^**O**^ (*left*), **2** ^**Y**^ (*middle*) and **2** ^**Y+**^ (*right*) along with the relevant Sn–Y distances and τ values.^47^ For a detailed selection of structural parameters for all compounds, see SI. Note: The labeling of atoms used in this figure is edited for the sake of compatibility among all compounds and does not necessarily correspond to the cif files in SI.

As mentioned above, a partial oxidation of **3** furnished compound **3** ^**PO**^ (Figure 3), while only the oxygen atom coordinates with the tin (Sn(1)–O(1) 2.3470(10) Å) leaving the P(1) atom pendant (Sn(1)–P(1) 4.8636(5) Å) and giving a trigonal bipyramidal geometry with Cl(1) and O(1) atoms located in axial positions (O(1)–Sn(1)–Cl(1) 176.74(3)°). Similarly, one chalcogen is bonded to the central atom in **3** ^**S**^ and **3** ^**Se**^ with interatomic distances Sn(1)–S(1) 2.8587(5) and Sn(1)–Se(1) 3.0253(5) Å, respectively. Again, a gradual distortion from an ideal trigonal bipyramidal array is illustrated by the τ values 0.85/0.77/0.70 for **3** ^**PO**^/**3** ^**S**^/**3** ^**Se**^, respectively. It is also noteworthy that significantly more distorted geometry was found for **2** ^**S**^ (τ = 0.54) and **2** ^**Se**^ (τ = 0.60).

**Figure 3:**
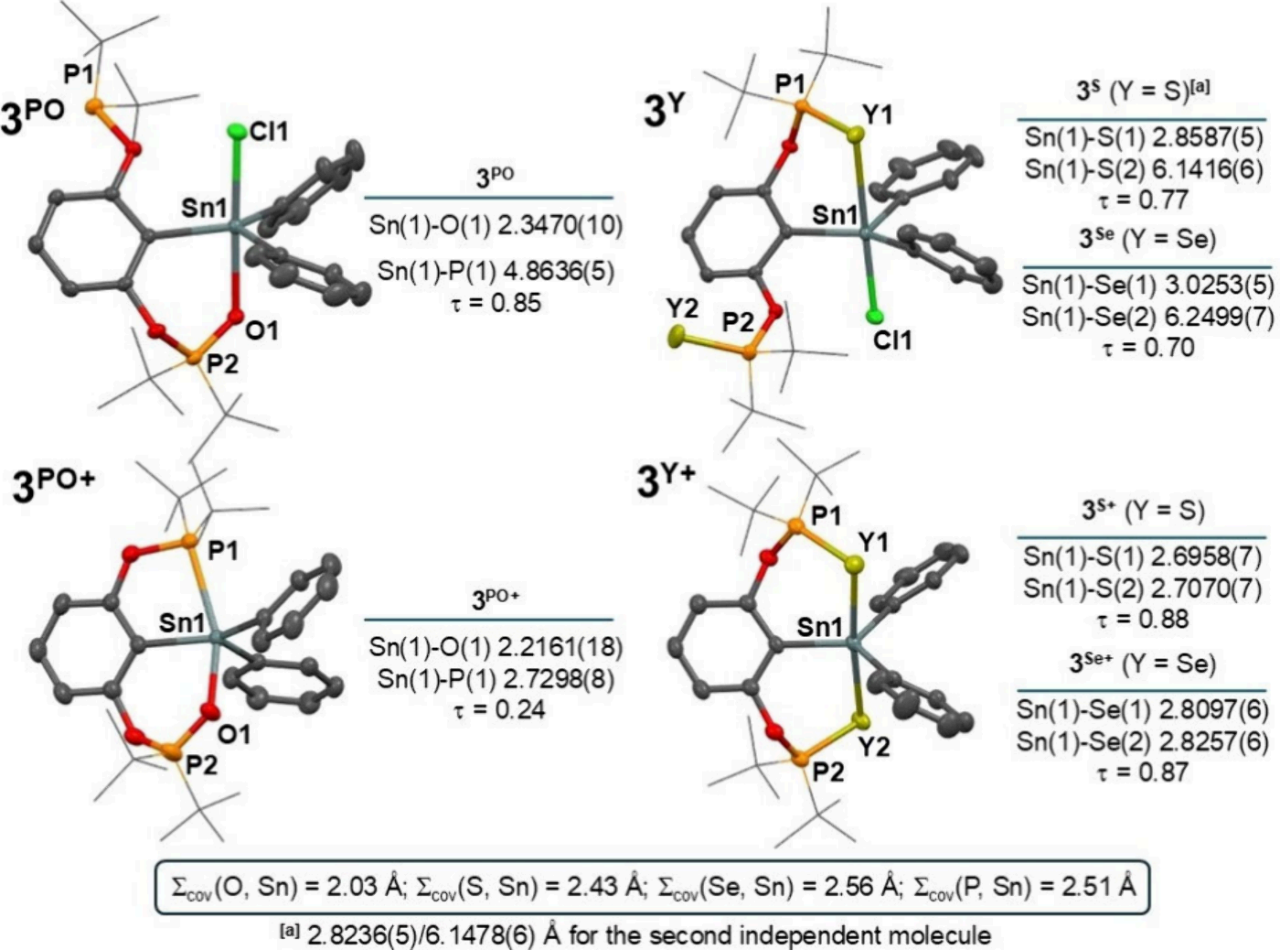
Generalized view on the molecular structures of **3** ^**PO**^ (*left up*), **3** ^**PO+**^ (*left down*), **3** ^**Y**^ (*right up*), and **3** ^**Y+**^ (*right down*) along with the relevant Sn–Y distances and τ values.^47^ For a detailed selection of structural parameters for all compounds, see SI. Note: The labeling of atoms used in this figure is edited for the sake of compatibility among all compounds and does not necessarily correspond to the cif files in SI.

The structures of cations **3** ^**S+**^ and **3** ^**Se+**^ resemble those of **1** ^**Y+**^ and **2** ^**Y+**^, with interatomic distances Sn(1)-S­(1/2) 2.6958(7)/2.7070(7); Sn(1)-Se­(1/2) 2.8097(6)/2.8257(6) Å and preferring a trigonal pyramidal geometry with heteroatoms in axial positions (cf. τ = 0.88 and 0.87 for **3** ^**S+**^ and **3** ^**Se+**^, respectively). The central atom is still five-coordinated in **3** ^**PO+**^ with short Sn(1)–P(1) 2.7298(8) and Sn(1)–O(1) 2.2161(18) Å distances, but the shape of the coordination polyhedron changed substantially to a slightly distorted square pyramid with one of the *ipso*-C­(phenyl) atoms in the apical position and with τ = 0.24, reflecting the presence of one five- and one six-membered ring. By this structure, **3** ^**PO+**^ resembles previously isolated purely *P,C,P*-chelated cations [ArSnCl_2_]^+^ (**1** ^**+**^, τ = 0.15), and [ArSnPh_2_]^+^ (**3** ^**+**^, τ = 0.05) (Ar = 2,6-(*t*Bu_2_PO)_2_C_6_H_3_).^36^

Finally, in the structure of the mixed donor **3** ^**OSe**^ (Figure 4) only the O(1) atom interacts with the tin (Sn(1)–O(1) 2.3558(14) Å) leading to trigonal bipyramidal coordination of the tin atom (τ = 0.87). The corresponding cations **3** ^**OS+**^ and **3** ^**OSe+**^ follow the trend found for their analogs with strongly coordinated heteroatoms (cf. Sn(1)–O(1) 2.225(2) and Sn(1)–S(1) 2.7065(8) Å in **3** ^**OS+**^, Sn(1)–O(1) 2.236(3) and Sn(1)–Se(1) 2.8031(6) Å in **3** ^**OSe+**^) in axial positions of distorted trigonal bipyramid (τ = 0.72 and 0.74, respectively).

**Figure 4:**
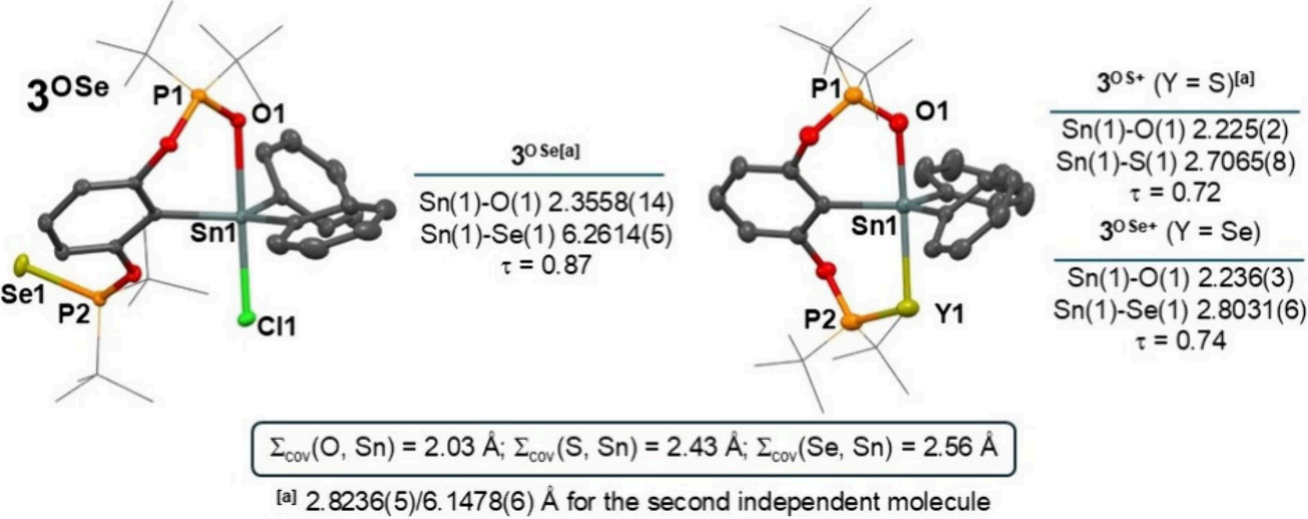
Generalized view on the molecular structures of **3** ^**OSe**^ (*left*) and **3** ^**OY+**^ (*left*) along with the relevant Sn–Y distances and τ values.^47^ For a detailed selection of structural parameters for all compounds, see SI. Note: The labeling of atoms used in this figure is edited for the sake of compatibility among all compounds and does not necessarily correspond to the cif files in SI.

In terms of trend-finding, the structure of the ionic compounds is more uniform compared to those of their neutral analogues. All symmetrical **1** ^**Y+**^ **-3** ^**Y+**^ and mixed donor cations **3** ^**OY+**^ contain two strong Y → Sn intramolecular interactions that make the central atom five-coordinated, adopting more or less distorted trigonal pyramidal coordination geometries (Y atoms in axial positions) according to obtained τ values. It is also evident that particular Sn–Y interatomic distances become slightly longer along the **1** ^**Y+**^ < **2** ^**Y+**^ < **3** ^**Y+**^ set reflecting a gradual attenuation of the tin atom’s Lewis acidity. Compound **3** ^**PO+**^ represents a clear exception among the isolated cations and, with both donor atoms coordinated, prefers a square pyramidal coordination instead of a trigonal bipyramidal reflecting the presence of one 5-membered chelate.

Regarding the neutral compounds, the coordination behavior is richer. All monorganotin­(IV) compounds **1** ^**Y**^ adopt octahedral geometry with meridionally coordinated pincer ligand and the same structural features are also encountered in the diorganotin­(IV) compound **2** ^**O**^. On the contrary, all other compounds, including **2** ^**S**^, **2** ^**Se**^, **3** ^**S**^, **3** ^**Se**^, as well as **3** ^**PO**^, **3** ^**OS**^, **3** ^**OSe**^ feature only one Y → Sn intramolecular interaction and importantly in the mixed donor compounds the oxygen donor always binds to the tin atom. Consequently, all these compounds contain five-coordinated tin centers, but whereas diorganotin­(IV) compounds **2** ^**S**^ and **2** ^**Se**^ revealed an intermediate structure between a square pyramid and a trigonal bipyramid, the rest of these derivatives exhibit geometries clearly shifted in favor of a trigonal bipyramid.

Clear trends can be also found in the structure of six-membered chelate rings. In all cases, a shortening of the Y → Sn interatomic distances reflects the increasing Lewis acidity of the tin atom (*vide supra*) and results in an elongation of present P=Y bonds at the same time. This becomes obvious when compared with interatomic distances for noncoordinated P=Y functions in **Ar** ^**Y**^ **Br** molecules (Table 1). Focusing on interatomic angles, the O–P–Y ones are only barely influenced by the substitution pattern at the tin atom and/or the nature of the Y donor atom falling within quite narrow interval from 109.13(8)° in **1** ^**O+**^ to 112.84(7)° in **3** ^**S+**^. By contrast, the P–Y–Sn interatomic angles differ significantly within this set of compounds. The values for P–O–Sn angles span over the interval between 121.05(6)° in **3** ^**PO**^ to 128.43(6)° in **1** ^**O**^, while these angles become significantly more acute ongoing to P–S–Sn (in the range from 99.44(2)° in **3** ^**S**^ to 103.49(3)° in **1** ^**S**^) and P–Se–Sn ones (from 93.89(2)° in **3** ^**Se**^ to 99.88(2)° in **1** ^**Se**^) and the differences between S and Se containing complexes are less evident. This finding is ascribed to the increasing covalent radii of heavier chalcogens leading to inherent elongation of the P=Y bond. Consequently, their placement into ideal position(s), according to the preferred tin atom coordination geometry, requires further deformation of the chelating rings. Considering these rings as very distant analogues of 1,4-cyclohexadienes bearing one C=C and one P=Y double bond, they adopt distorted slightly twisted boat conformation that becomes more puckered for heavier Y atoms as illustrated in Figure 5 for **1** ^**Y**^ and **1** ^**Y+**^.

**Figure 5:**
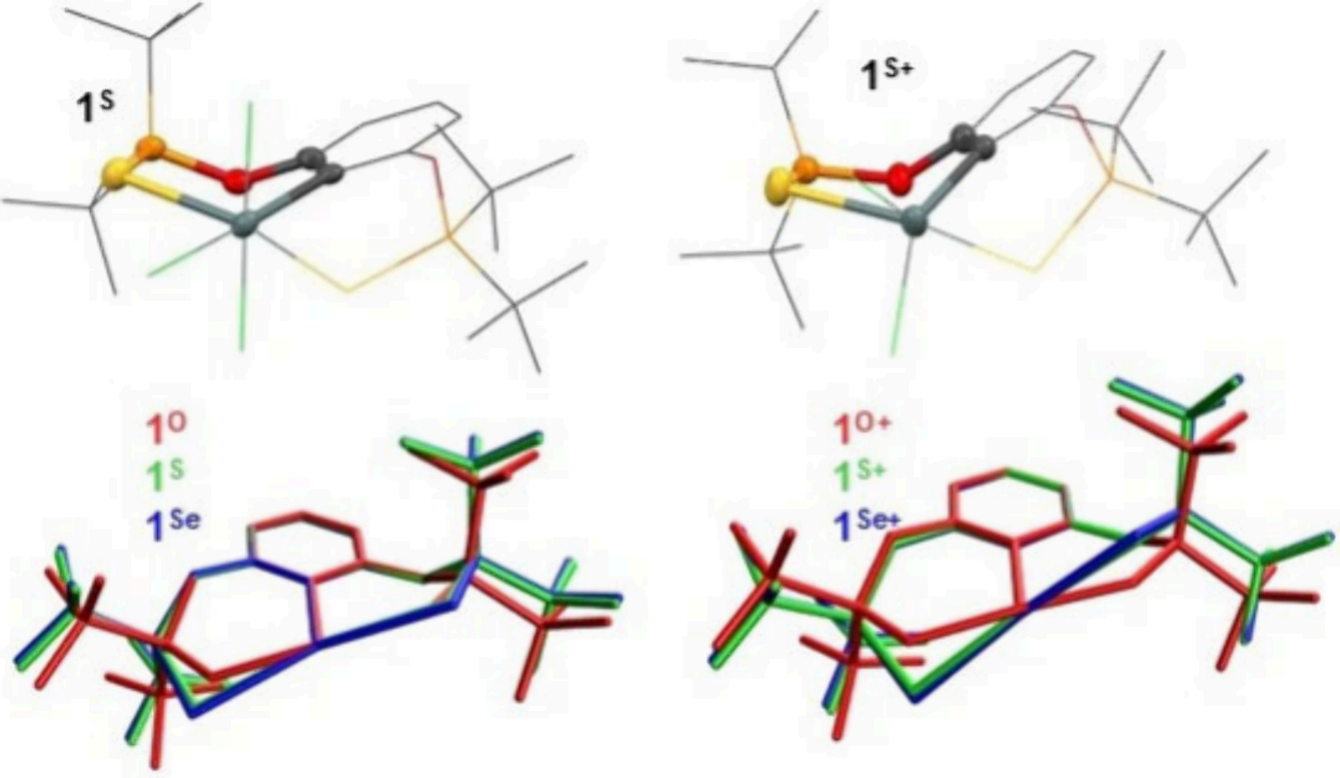
View on the boat conformation of chelate rings in **1** ^**S**^ (*left up*) and **1** ^**S+**^ (*right up*) along with overlapped structures of **1** ^**O**^/**1** ^**S**^/**1** ^**Se**^ (*left down*) and **1** ^**O+**^/**1** ^**S+**^/**1** ^**Se+**^ (*right down*).

**Table 1:** Selected Interatomic Distances [Å] and Interatomic Angles [°] for All of the Compounds in This Study

Compound	Sn–Y	P=Y	Sn–Y–P	Compound	Sn–Y	P=Y	Sn–Y–P
**Ar** ^**O**^ **Br** (Y = O)	–	1.469(2)	–	**3** ^**S**^ **(Y = S)** ^[^ ^a^ ^,^ ^b^ ^]^	2.8587(5)	1.9720(7)	99.44(2)
		1.4778(18)			6.1416(6)	1.9452(6)	-
**Ar** ^**S**^ **Br** (Y = S)	–	1.9331(6)	–	**3** ^**Se**^ **(Y = Se)** ^[^ ^b^ ^]^	3.0253(5)	2.1226(7)	93.89(2)
		1.9422(6)			6.2499(7)	2.0992(7)	-
**Ar** ^**Se**^ **Br** (Y = Se)	–	2.0985(15)	–	**3** ^**S+**^ **(Y = S)**	2.6958(7)	1.9923(8)	100.61(3)
		2.0905(14)			2.7070(7)	1.9816(8)	101.80(3)
**1** ^**O**^ (Y = O)	2.1431(11)	1.5136(11)	128.43(6)	**3** ^**Se+**^ **(Y = Se)**	2.8097(6)	2.1341(8)	96.26(2)
	2.1386(11)	1.5130(12)	126.98(6)		2.8257(6)	2.1326(8)	97.23(3)
**1** ^**S**^ (Y = S)^[^ ^a^ ^]^	2.5722(7)	2.0023(9)	103.09(3)	**3** ^**OS**^ **(Y = O)** ^[^ ^d^ ^]^	2.469	1.515	119.4
	2.6162(7)	1.9996(10)	103.49(3)				
**1** ^**Se**^ (Y = Se)	2.6908(5)	2.1634(5)	97.04(2)	**3** ^**OS**^ **(Y = S)** ^[^ ^d^ ^,^ ^e^ ^]^	6.058	1.955	–
	2.7022(5)	2.1565(6)	99.88(2)				
**1** ^**O+**^ **(Y = O)**	2.1356(14)	1.5148(14)	127.42(8)	**3** ^**OS**e^ **(Y = O)**	2.3558(14)	1.5034(15)	120.22(8)
	2.1356(14)	1.5148(14)	127.42(8)				
**1** ^**S+**^ **(Y = S)**	2.5907(7)	2.0006(9)	103.16(3)	**3** ^**OS**e^ **(Y = Se)** ^[^ ^e^ ^]^	6.2614(5)	2.0996(6)	–
	2.5907(7)	2.0006(9)	103.16(3)				
**1** ^**Se+**^ **(Y = Se)**	2.6917(4)	2.1519(8)	99.10(2)	**3** ^**OS+**^ **(Y = O)**	2.225(2)	1.518(2)	126.15(12)
	2.6917(4)	2.1519(8)	99.10(2)				
**2** ^**O**^ **(Y = O)**	2.2003(13)	1.5110(14)	126.41(7)	**3** ^**OS+**^ **(Y = S)**	2.7065(8)	1.9770(12)	101.00(4)
	2.1919(14)	1.5085(13)	126.57(8)				
**2** ^**S**^ **(Y = S)** ^[^ ^a^ ^,^ ^b^ ^]^	2.8933(7)	1.9808(6)	100.66(2)	**3** ^**OSe+**^ **(Y = O)**	2.236(3)	1.504(4)	126.37(16)
	5.8810(6)	1.9422(6)	-				
**2** ^**Se**^ **(Y = Se)** ^[^ ^b^ ^]^	2.9331(5)	2.1399(7)	95.94(2)	**3** ^**OSe+**^ **(Y = Se)**	2.8031(6)	2.1259(8)	95.08(3)
	6.0469(5)	2.0964(7)	-				
**2** ^**O+**^ **(Y = O)**	2.1601(17)	1.5023(19)	126.86(10)	**3** ^**PO**^ **(Y = O)** ^[^ ^c^ ^]^	2.3470(10)	1.5052(11)	121.05(6)
	2.1537(17)	1.5107(17)	127.93(10)				
**2** ^**S+**^ **(Y = S)**	2.6442(9)	1.9879(8)	102.90(3)	**3** ^**PO+**^ **(Y = O)**	2.2161(18)	1.5045(18)	128.27(10)
	2.6383(8)	1.9898(7)	101.38(3)				
**2** ^**Se+**^ **(Y = Se)**	2.7694(6)	2.1456(13)	99.85(3)	**3** ^**PO+**^ **(Y = P)**	2.7298(8)	–	–
	2.7446(6)	2.1508(14)	97.16(4)				

a Values for one of two structurally similar independent molecules in the unit cell.

b Only one of the P–Y functions is coordinated to the tin atom.

c *t*Bu_2_P function does not coordinate to the tin atom.

d Data taken from calculated structure.

e P–Y (Y = S or Se) function does not coordinate to the tin atom.

### NMR Studies

Multinuclear NMR spectroscopy, including ^1^H, ^11^B­{^1^H},^13^C­{^1^H}, ^19^F­{^1^H}, ^31^P­{^1^H}, ^77^Se­{^1^H} and ^119^Sn­{^1^H} isotopes (Table 2), was used for a detailed structure description of all compounds in solution. The variable temperature VT-^1^H and ^31^P­{^1^H} NMR spectroscopy was applied for explanation of dynamic processes in solution for selected compounds. The observed data are described below separately for monorogano-, diorgano- and triorganotin­(IV) compounds/cations, while they are compared with those previously obtained for parent *P,C,P*-pincer compounds **1**–**3** and related cations [ArSnPh_2_]­[BArF] (**1** ^**+**^ **[BArF]** ^**–**^), [ArSnPhCl]­[BArF] (**2** ^**+**^ **[BArF]** ^**–**^) and [ArSnCl_2_]­[BArF] (**3** ^**+**^ **[BArF]** ^**–**^) (Ar = 2,6-(*t*Bu_2_PO)_2_C_6_H_3_). Please note that in the following discussion the NMR data of the [BArF] anion are not mentioned, but its presence in all ionic compounds was clearly proved by observation of two singlets in ^1^H, a complex set of signals in ^13^C­{^1^H} (see SI), a singlet in ^11^B­{^1^H} (δ­(^11^B) ∼ −7 ppm) and a singlet in ^19^F­{^1^H} NMR spectra (δ­(^19^F) ∼ −62.5 ppm).

**Table 2:** Selected NMR Data for All Compounds^a^

Compound	δ(^119^Sn)	δ(^31^P) (*J*_Sn,P_)	δ(^77^Se) (*J*_Se,P_)	Compound	δ(^119^Sn)	δ(^31^P) (*J*_Sn,P_)	δ(^77^Se) (*J*_Se,P_)
**1** ^**O[**^ ^a^ ^**]**^	–632.6	85.0 (73)	–	**2** ^**Se+**^ **[BArF]** ^**–[**^ ^a^ ^**]**^	–322.7	141.7	–153.8 (635)
**1** ^**S[**^ ^b^ ^**]**^	–640.3	125.6	–	**3** ^**S[**^ ^b^ ^**]**^	–209.2	131.3	–
**1** ^**Se[**^ ^a^ ^**]**^	–773.4	131.0	–2.8 (621)	**3** ^**Se[**^ ^a^ ^**]**^	–231.4	141.6	–252.7^[^ ^e^ ^]^ (764)
**1** ^**O+**^ **[BArF]** ^**‑[**^ ^a^ ^**]**^	–393.1	91.8 (74)	–	**3** ^**S+**^ **[BArF]** ^**–[**^ ^c^ ^**]**^	–298.9	132.6	–
**1** ^**S+**^ **[BArF]** ^**‑[**^ ^c^ ^**]**^	–371.1	133.0	–	**3** ^**Se+**^ **[BArF]** ^**‑[**^ ^a^ ^**]**^	–311.7	141.4	–207.1 (660)
**1** ^**Se+**^ **[BArF]** ^**‑[**^ ^d^ ^**]**^	–692.1	134.4	6.6 (576)	**3** ^**PO[**^ ^a^ ^**]**^	–246.9	77.1; 159.2	–
**2** ^**O[**^ ^a^ ^**]**^	–529.5	82.9 (67)	–	**3** ^**OS[**^ ^a^ ^**]**^	–258.2^[^ ^f^ ^]^	78.9; 132.1	–
**2** ^**S[**^ ^b^ ^**]**^	–217.3	132.9	–	**3** ^**OS**e**[**^ ^a^ ^**]**^	–258.9	79.1; 141.8	–320.9^[^ ^f^ ^]^ (780)
**2** ^**Se[**^ ^a^ ^**]**^	–264.3	141.9	–222.2 (733)	**3** ^**PO+**^ **[BArF]** ^**–[**^ ^a^ ^**]**^	–255.2	84.4 (48)	–
						101.8 (960)	
**2** ^**O+**^ **[BArF]** ^**–[**^ ^a^ ^**]**^	–345.0	87.4 (55)	–	**3** ^**OS+**^ **[BArF]** ^**–[**^ ^a^ ^**]**^	–293.9	84.6; 132.4	–
**2** ^**S+**^ **[BArF]** ^**–[**^ ^c^ ^**]**^	–304.4	134.2	–	**3** ^**OSe+**^ **[BArF]** ^**–[**^ ^a^ ^**]**^	–298.1	84.4 (36)	–220.1 (647)
						139.9	

a Chemical shifts are given in [ppm] and coupling constants in [Hz].

b Acquired in CDCl_3_.

c Acquired in C_6_D_6_.

d Acquired in CD_2_Cl_2_.

e Acquired in CD_3_CN.

f Measured at 323 K.

g Measured at 263 K.

Compounds **1** ^**Y**^ and the corresponding cations **1** ^**Y+**^ revealed one set of signals in both ^1^H, ^13^C­{^1^H} NMR spectra indicating a symmetric coordination of the ligand to the tin atom in solution similarly as established above in the solid state. Accordingly, the ^31^P­{^1^H} NMR spectrum showed one signal at 85.0/125.6/131.0 ppm for **1** ^**O**^/**1** ^**S**^/**1** ^**Se**^ flanked by tin (^2^ *J*(^119/117^Sn,^31^P) = 73 Hz) and selenium satellites (^1^ *J*(^77^Se,^31^P) = 621 Hz) for **1** ^**O**^ and **1** ^**Se**^, respectively. Corresponding signals are only slightly deshielded in the ionic compounds **1** ^**O+**^/**1** ^**S+**^/**1** ^**Se+**^ with values 91.8/133.0/134.4 ppm, respectively, while the coupling constants are similar (^2^ *J*(^119/117^Sn,^31^P) = 74 Hz, for **1** ^**O+**^) and (^1^ *J*(^77^Se,^31^P) = 576 Hz, for **1** ^**Se+**^). The ^119^Sn­{^1^H} NMR spectra revealed one signal at −632/−640/−773 ppm for **1** ^**O**^/**1** ^**S**^/**1** ^**Se**^ that are deshielded in related cations −393/–371/–692 ppm for **1** ^**O+**^/**1** ^**S+**^/**1** ^**Se+**^, respectively. Finally, the ^77^Se­{^1^H} NMR spectra contained expected doublets due to the coupling with phosphorus nuclei at −2.8 ppm (^1^ *J*(^77^Se,^31^P) = 621 Hz) for **1** ^**Se**^ and slightly shifted to 6.6 ppm (^1^ *J*(^77^Se,^31^P) = 576 Hz) for **1** ^**Se+**^, but both are significantly deshielded compared to the value of −311 ppm (^1^ *J*(^77^Se,^31^P) = 800 Hz) detected for **Ar** ^**Se**^ **Br** lacking any Se → Sn interactions (Ar^Se^ **=** 2,6-(*t*Bu_2_(Se)­PO)_2_C_6_H_3_, for the synthesis see SI).

The molecular structures of **2** ^**O**^ vs **2** ^**S**^ and **2** ^**Se**^ as determined by *sc* X-ray diffraction analysis differ significantly, because in **2** ^**O**^ both donor groups are coordinated whereas in **2** ^**S**^ and **2** ^**Se**^ one of them remained pendant and this situation is also reflected in solution. The ^1^H, ^13^C­{^1^H} NMR spectra contained one set of relatively sharp signals for the ligand in all cases. The ^31^P­{^1^H} NMR spectrum of **2** ^**O**^ contained one signal at 82.9 ppm (^2^ *J*(^119/117^Sn,^31^P) = 67 Hz), while the ^119^Sn­{^1^H} NMR spectrum showed a signal at −530 ppm. Both values resemble those detected for **1** ^**O**^ above, thereby indicating coordination of both ligand arms to the tin atom giving an octahedral environment around the central atom. Surprisingly, **2** ^**S**^ and **2** ^**Se**^ showed only one signal in ^31^P­{^1^H} NMR spectra 132.9/141.9 ppm for **2** ^**S**^/**2** ^**Se**^ (^1^ *J*(^77^Se,^31^P) = 733 Hz), which contrasts with the geometry in the solid state, and most probably indicates a fluxional behavior comprising fast coordination/decoordination of both ligand arms. The VT-^1^H NMR spectra (in CDCl_3_; range 323–240 K) revealed sharpening of the signals upon heating and their expected broadening upon cooling, because proposed dynamic process is slowed down. Unfortunately, no clear decoalescence could be reached even at the probe limit of 240 K. Similarly, VT-^31^P­{^1^H} NMR spectra of **2** ^**Se**^ showed gradual broadening of the signal leading only to observation of a second very wide signal at 133 ppm at 240 K. By contrast, VT-^31^P­{^1^H} NMR spectra of **2** ^**S**^ contained two well developed signals at 133.8 and 126.8 ppm at 240 K, being indicative for the presence of two different P atoms (coordinated and noncoordinated one), which well corresponds to the structure determined in the solid state (Figures S152–S155). It is important to note that all the changes observed are fully reversible upon heating of the samples. The dynamic behavior of **2** ^**S**^/**2** ^**Se**^ in solution is further corroborated by ^119^Sn­{^1^H} NMR spectra exhibiting signals at −217 ppm for **2** ^**S**^ and −264 ppm for **2** ^**Se**^, which are significantly deshielded in comparison with six-coordinated **1** ^**S**^ (−640 ppm) and **1** ^**Se**^ (−773 ppm) indicating a lower coordination number caused by a fluxional behavior of the pincer ligand at ambient temperature. Finally, the ^77^Se­{^1^H} NMR spectrum of **2** ^**Se**^ revealed doublet at −222.2 ppm (^1^ *J*(^77^Se,^31^P) = 733 Hz) being significantly different from **1** ^**Se**^ (−2.8 ppm; (^1^ *J*(^77^Se,^31^P) = 621 Hz). The ^1^H, ^13^C­{^1^H} NMR spectra of diorganotin­(IV) cations **2** ^**O+**^/**2** ^**S+**^/**2** ^**Se+**^ are closely related and reflect a tight coordination of both Y donors, which in combination with nonsymmetric substitution at the tin caused the observation of two sets of signals for magnetically nonequivalent *t*Bu group. Two of them are oriented toward the chlorine atom, whereas the other two to the phenyl substituent. Signals at 87.4/134.2/141.7 ppm for **2** ^**O+**^/**2** ^**S+**^/**2** ^**Se+**^, (^2^ *J*(^119/117^Sn,^31^P) = 55 Hz, for **2** ^**O+**^) and (^1^ *J*(^77^Se,^31^P) = 635 Hz, for **2** ^**Se+**^), respectively, in ^31^P­{^1^H} NMR spectra are almost identical compared to neutral compounds. On the contrary, the triplet obtained at −345 ppm (^2^ *J*(^119/117^Sn,^31^P) = 55 Hz) in the ^119^Sn­{^1^H} NMR spectrum for **2** ^**O+**^ is significantly deshielded (cf. −530 ppm for **2** ^**O**^) which is consistent with both the positive charge on the tin atom and reduction of the coordination number from six to five. The signals for **2** ^**S+**^/**2** ^**Se+**^ at −304/−323 ppm are conversely shielded with respect to neutral **2** ^**S**^/**2** ^**Se**^ at −217/−264 ppm, this finding can be ascribed to two contradictory effects, i.e. positive charge on the tin atom (expected deshielding) vs tight coordination of both Y-donors not obtained in neutral complexes (shielding). The ^77^Se­{^1^H} NMR spectrum of **2** ^**Se+**^ showed a low-field shifted doublet at −153.8 ppm (^1^ *J*(^77^Se,^31^P) = 635 Hz) in comparison to **2** ^**Se**^.

The neutral triorganotin­(IV) compounds **3** ^**S**^/**3** ^**Se**^ exhibit one set of signals in ^1^H, ^13^C­{^1^H} NMR spectra for the ligand including the donor arms, even though only one of them is coordinated in the solid state (*vide supra*), again indicating the fluxional nature of the pincer ligand. The ^31^P­{^1^H} NMR spectra showed one signal at 131.3/141.6 ppm for **3** ^**S**^/**3** ^**Se**^, respectively, flanked by selenium satellites (^1^ *J*(^77^Se,^31^P) = 748 Hz) for the latter. The VT-^1^H,^31^P­{^1^H} NMR spectra showed only broadening of all signals upon cooling (Figures S156–S159), but no decoalescence could be reached even in the ^31^P­{^1^H} NMR spectra until the limit temperature 240 K. This contrasts with situation in **2** ^**S**^ and **2** ^**Se**^ and probably indicates lower energetic barrier for this process **3** ^**S**^/**3** ^**Se**^ (*vide infra*). This finding is also consistent with expected lower tin atom Lewis acidity in the latter. One signal was detected in the ^119^Sn­{^1^H} NMR spectra −209 (for **3** ^**S**^) and −231 ppm (for **3** ^**Se**^). The ^77^Se­{^1^H} NMR spectrum of **3** ^**Se**^ revealed a doublet at −252.7 ppm (^1^ *J*(^77^Se,^31^P) = 748 Hz). The ionization giving compounds **3** ^**S+**^ and **3** ^**Se+**^ resulted in NMR spectra following similar trends as found above for **2** ^**S**^/**2** ^**Se**^ vs **2** ^**S+**^/**2** ^**Se+**^. Consequently, signals at 132.6/141.4 ppm (^1^ *J*(^77^Se,^31^P) = 660 Hz) and −299/−312 ppm for **3** ^**S+**^/**3** ^**Se+**^ were detected in ^31^P­{^1^H} and ^119^Sn­{^1^H} NMR spectra, respectively.

The ^1^H NMR spectra of neutral mixed-donor species **3** ^**OS**^/**3** ^**OSe**^ showed quite broadened set of signals suggesting a dynamic structure of these compounds in solution at ambient temperature. The VT-^1^H NMR spectra in CDCl_3_ showed gradual sharpening of all lines upon cooling to 240 K resulting in the observation of three signals for the aromatic hydrogen atoms of nonsymmetrically substituted pincer ligand. Furthermore, four well separated and developed signals were detected for *t*Bu groups indicating their magnetic nonequivalence at this temperature (Figures S160–S163). This finding indicates that the structure at low temperature resembles that found in the solid state. The *t*Bu_2_P­(O) group coordinates the tin atom leaving *t*Bu_2_P­(S/Se) group pendant, which eventually makes all four *t*Bu groups different from the ^1^H NMR spectroscopy point of view (Figure S164). The ^31^P­{^1^H} NMR spectra contained two signals for each compound at 77.1/159.2 (**3** ^**PO**^), 78.9/132.1 (**3** ^**OS**^) and 79.1/141.8 ppm (**3** ^**OSe**^, ^1^ *J*(^77^Se,^31^P) = 780 Hz), former value always corresponds to the oxidized *t*Bu_2_P­(O) group, while the second to the *t*Bu_2_P, *t*Bu_2_P­(S) and *t*Bu_2_P­(Se) group, respectively. The ^119^Sn­{^1^H} NMR spectra are rather similar for all three compounds with chemical shifts of −247/–258/–259 ppm for **3** ^**PO**^/**3** ^**OS**^/**3** ^**OSe**^. This most probably reflects very similar structures for these three compounds that resemble those of **3** ^**PO**^ and **3** ^**OSe**^ observed in the solid state with coordinated *t*Bu_2_P­(O) group leaving the second ligand arm pendant. This conclusion seems to be nicely corroborated by the ^31^P­{^1^H} NMR spectrum of **3** ^**PO**^, where the signal for the *t*Bu_2_P moiety at 159.2 ppm resembles value for the parent ligand precursor.^36^ Furthermore, the ^77^Se­{^1^H} NMR spectrum of **3** ^**OSe**^ exhibited signal at −320.9 ppm (^1^ *J*(^77^Se,^31^P) = 780 Hz) both values again being very close to uncoordinated *t*Bu_2_P­(Se) groups in **Ar** ^**Se**^ **Br**, i.e. −311.2 ppm (^1^ *J*(^77^Se,^31^P) = 800 Hz).

In the cations **3** ^**PO+**^/**3** ^**OS+**^/**3** ^**OSe+**^, the second donor group is also coordinated to the tin in addition to the already attached *t*Bu_2_P­(O) function in the neutral species. This fact is well documented by the ^31^P­{^1^H} NMR spectrum of **3** ^**PO+**^ containing two signals at 84.4 (for *t*Bu_2_P­(O)) and 101.8 ppm (for *t*Bu_2_P, ^1^ *J*(^119/117^Sn,^31^P) = 960/920 Hz), the latter value is significantly shielded compared to **3** ^**PO**^. Furthermore, this value resembles that obtained in the related cation **3** ^**+**^ (i.e., [ArSnPh_2_]^+^, 105.7 ppm, ^1^ *J*(^119/117^Sn,^31^P) = 835 Hz).^36^ The values of δ­(^31^P) for remaining cations are logically less influenced compared to neutral precursors, because the phosphorus atoms are not directly connected to the tin atom (cf. 84.6/132.4 (**3** ^**OS+**^) and 84.4/139.9 ppm (**3** ^**OSe+**^, ^1^ *J*(^77^Se,^31^P) = 647 Hz). However, ^77^Se­{^1^H} NMR spectrum of **3** ^**OSe+**^ showed doublet at −220.1 ppm again being significantly shifted in comparison with **3** ^**OSe**^ (−320.9 ppm). The ^119^Sn­{^1^H} NMR spectra revealed signals at −255/–294/–298 ppm for **3** ^**PO+**^/**3** ^**OS+**^/**3** ^**OSe+**^, respectively, again exhibiting a similar trend as discussed above for **2** ^**S**^/**2** ^**Se**^ vs **2** ^**S+**^/**2** ^**Se+**^, and **3** ^**S**^/**3** ^**Se**^ vs **3** ^**S+**^/**3** ^**Se+**^, i.e. the influence of the positive charge at the tin atom ongoing from neutral to ionic species is compensated by the tight coordination of the second donor arms virtually causing a shielding of tin atoms in respective NMR spectra.

Altogether, the results of this comprehensive NMR study proved that the structures established by the help of *sc* X-ray diffraction analysis are mostly retained in solution. The neutral compounds **2** ^**S**^/**2** ^**Se**^ and **3** ^**S**^/**3** ^**Se**^ are exceptions, because in the solid state only one of the accessible donor groups, *i.e. t*Bu_2_P­(S) or *t*Bu_2_P­(Se), coordinates the tin in the solid state, whereas these compounds exhibit a fluxional behavior in solution at ambient temperature as proven by VT-NMR experiments (*vide supra*). This phenomenon comprises the fast coordination/decoordination process that is often encountered in similar pincer organotin­(IV) compounds. Similarly in the case of **3** ^**OS**^/**3** ^**OSe**^, *t*Bu_2_P­(O) function competes in the tin atom coordination with *t*Bu_2_P­(S/Se) ones resulting in fluxional structures at ambient conditions, but cooling of the solution resulted in slowing down the whole process sufficiently to recognize the structure, where only *t*Bu_2_P­(O) coordinates to the central atom, while *t*Bu_2_P­(S/Se) moieties remain pendant.

### Theoretical Studies

To shed light on the role of the chalcogen center in the coordination modes and the thermodynamic stability of Sn­(IV) complexes in this work, DFT calculations have been carried out using the ωB97X-D functional combined with def2-SVP and def2-TZVP basis sets (for detailed geometrical data, see SI). The same levels of theory were previously applied successfully to describe the bonding patterns of similar complexes.^36^ Regarding the cationic complexes, the geometries simulated without the weakly coordinating [BArF]^−^ counteranion are practically identical with those obtained by *sc* XRD analysis. The agreement of the calculated and experimentally determined structures is also satisfactory in the case of the neutral complexes (for a few exceptions, see below). On the optimized geometries, Natural Bond Orbital (NBO) analysis was conducted. In addition, to estimate the covalent character of the bonding interactions, Wiberg Bond Indices (WBI) were calculated. Furthermore, Atoms-in-Molecules (AIM) analysis was used to locate the bond-critical points (BCPs) and characterize the properties thereof (for details, see Tables S2–S9).

### Coordination Modes

To understand the difference in the coordination modes, it is useful to compare selected bonding descriptors, namely WBIs and the total energy density (*H*) in the Y → Sn BCPs. The WBIs account for the covalent character of the bonds, and in contrast to the corresponding interatomic distances, no reference is needed for comparison. To distinguish between the nature of the weak interactions, the sign of *H* at the bond critical point is conclusive, as it is negative in the case of stronger dative bonding, while positive for weaker noncovalent interactions (the values close to zero exclude a substantial covalent (dative) character).

Regarding the neutral complexes, two coordination modes can be differentiated: a more covalent, dative, and a weaker σ-hole interaction. In complexes **1** ^**Y**^ (Y = O, S, Se) and **2** ^**O**^ both chalcogen centers coordinate with the tin forming a medium strength dative bond (WBI = 0.25, 0.43, and 0.49 for **1** ^**O**^, **1** ^**S**^, and **1** ^**Se**^, respectively, complemented by negative *H* values (from −0.013 to −0.010). On the contrary, in complexes **2** ^**Y**^ (Y = S, Se), **3** ^**Y**^ (Y = O, S, Se) and mixed analogues (**3** ^**PO**^, **3** ^**OS**^, **3** ^**OSe**^) only one of the chalcogens coordinates to the tin center and a weaker σ-hole interaction is established. The WBI values are below 0.20 (WBI= 0.12, 0.15, and 0.15 for **3** ^**O**^, **3** ^**S**^, and **3** ^**Se**^, respectively), while the *H* values are practically zero, with Y–Sn–Cl interatomic angles close to linear. In these complexes, the calculated chalcogen-tin interatomic distances differ somewhat (0.2–0.3 Å) from those in the solid state, due to the weak nature of the interaction (in accordance with the fluxionality observed by NMR).

In terms of the cationic complexes, in cations **1** ^**Y+**^ the Y → Sn interactions are only slightly stronger than those in the corresponding neutral congeners (WBI = 0.28, 0.48, and 0.54 for **1** ^**O+**^, **1** ^**S+**^ and **1** ^**Se+**^, respectively, with even more negative *H* values (from −0.016 to −0.013). These features contrast starkly with the cationic complexes with P-donors (**1** ^**+**^–**3** ^**+**^), where two, relatively strong P → Sn dative bonds stabilize the tin center. Nonetheless, in complexes **2** ^**Y+**^ and **3** ^**Y+**^ still both chalcogens coordinate with the tin center, forming rather strong dative bonds, contrary to the neutral analogues (except **2** ^**O**^). A slight trend can also be described based on the bonding descriptors, namely the weakening of the Y → Sn interactions with the increasing number of phenyl substituents (WBI = 0.28, 0.25 and 0.23; *H*= −0.016, −0.012 and −0.009 for **1** ^**O+**^, **2** ^**O+**^ and **3** ^**O+**^, respectively), however, these differences are less substantial than those observed for the dependency on the chalcogen centers.

### Thermodynamic Aspects of Stabilization Effects

To assess the thermodynamic stability of the complexes and quantify the strength of the interactions between Sn and the chalcogen centers, a total interaction energy (Δ*E*_int_) was calculated from electronic energies using a hypothetical model reaction (Scheme 3).

**Scheme 3:**
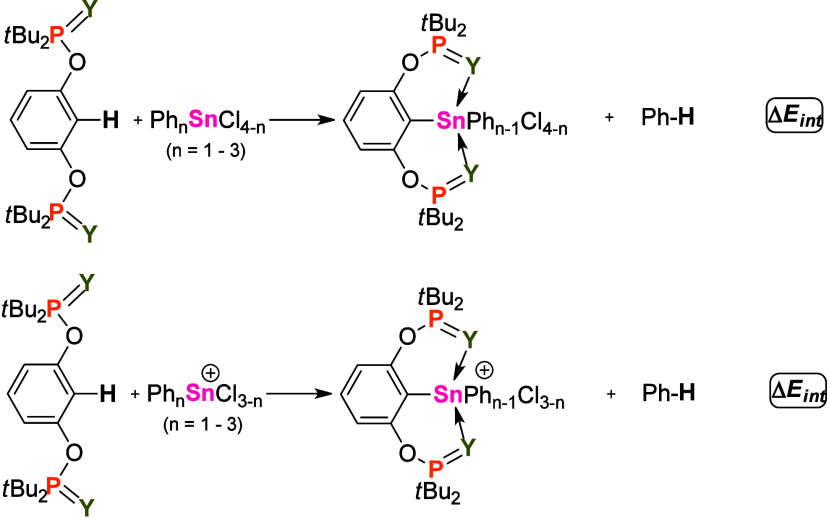
Model Isodesmic Reaction Equations Defining the Total Interaction Energy (Δ*E*_int_) for All Compounds

The more negative the energy values (Δ*E*_int_), the higher the relative stability of the complex, in line with a larger extent of stabilizing interactions (see Table S10). As descending in the chalcogen group (O, S, Se), the total interaction energy increases monotonously (becomes less negative) in the case of all the complexes. Again, the complexes with sulfur and selenium donors are highly similar (Δ*E*_int_ values within 2 kcal/mol). Compared to these, the analogues with oxygen donors proved to be more stable (by 7–20 kcal/mol). In the case of the neutral complexes, the total interaction energies are less significant (in the range from −16.8 to 4.6 kcal/mol) than those obtained for the cationic analogues (see below), indicating the absence of a stronger dative bond. These moderate Δ*E*_int_ values are consistent with the formation of a weaker dative or a σ-hole interaction. The positive values can be traced back to slight destabilization caused by the strained octahedral geometry around the tin center for complexes **1** ^**S**^ and **1** ^**Se**^. The range of these Δ*E*_int_ values are in line with the complex formation energies of model systems (that is, the reaction (PhO)­Ph_2_P=Y + SnPh_*x*_Cl_4–*x*_ = (PhO)­Ph_2_P=Y → SnPh_*x*_Cl_4–*x*_, for *x* = 0, 1, 2, 3 and 4) occupying a range between −16.3 to −1.4 kcal/mol (see Table S36). On the contrary to the neutral systems, in the cationic counterparts, the calculated total interaction energy values are more substantial (ranging between −88.2 to −56.6 kcal/mol), due to the higher Lewis acidity of the cationic tin centers (see for selected examples in Figure 6a, d, g). Again, the complex formation energies (according to the reaction (PhO)­Ph_2_P=Y + [SnPh_*x*_Cl_3‑x_]^+^ = [(PhO)­Ph_2_P=Y → SnPh_*x*_Cl_3‑x_]^+^, for *x* = 0, 1, 2 and 3) encompass a similar range as the Δ*E*_int_ values between −90.6 and −54.2 kcal/mol (see Table S36). Parallelly, the geometries also become less crowded, being in an intermediate shape between a tetragonal pyramid and a trigonal bipyramid.

**Figure 6:**
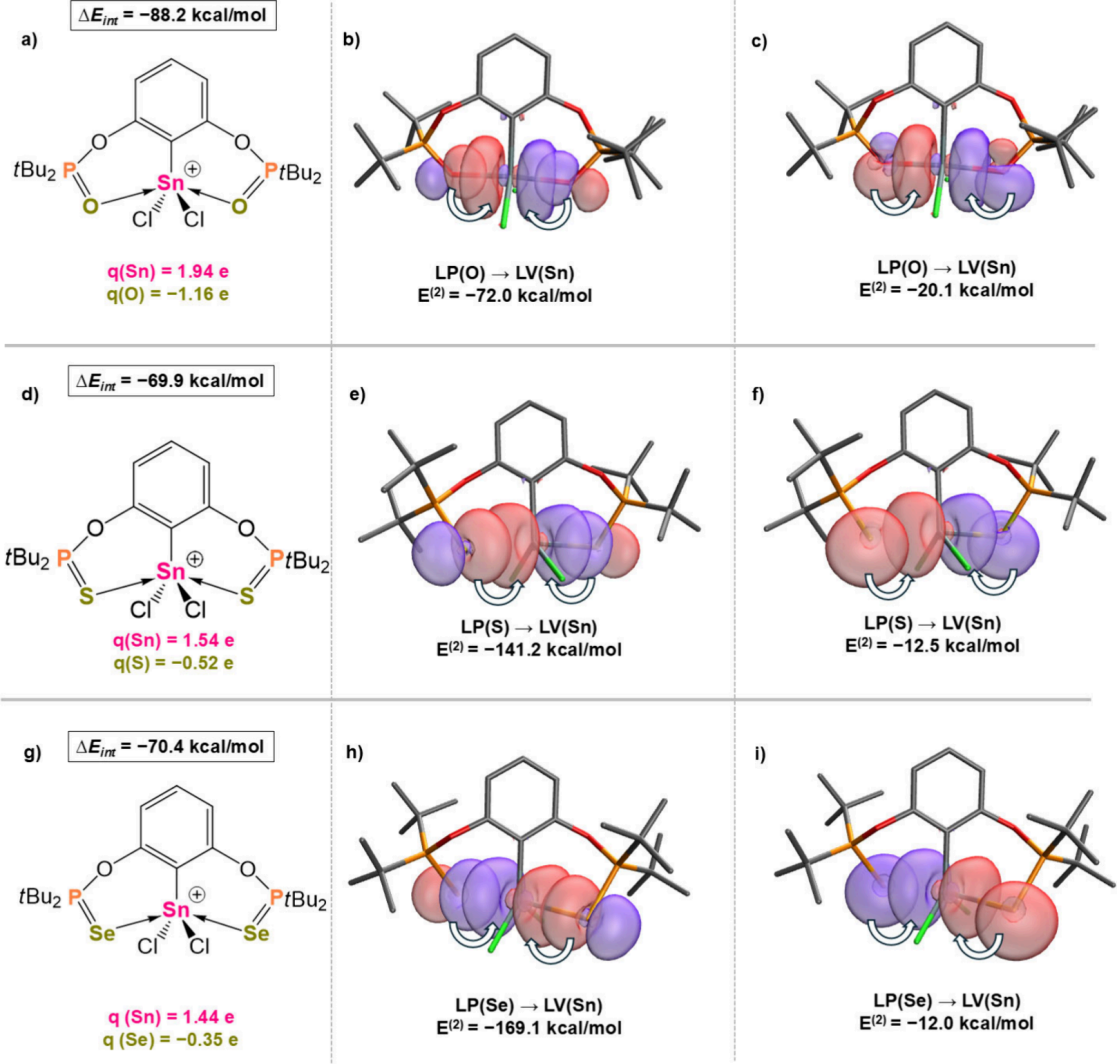
Total interaction energies (Δ*E*_int_) and NPA charges at the Sn and Y centers (a,d,g); donor–acceptor interactions (LP = lone pair, LV = lone valence) obtained from NBO analyses: (b,e,h) donation from the p-type lone pair at the Y center; (c,f,i) donation from the s-type lone pair at the Y center for **1** ^**O+**^, **1** ^**S+**^ and **1** ^**Se+**^, respectively at the ωB97X–D/def2–TZVP// ωB97X–D/def2–SVP level of theory.

To account for these tendencies, second-order perturbation energy values (E^(2)^) were employed to compare the relative covalent characters of the Y → Sn interactions (see Tables S11–S34). Such analysis is able to quantify the possible interactions between donor and acceptor NBOs by estimating their energetic increments by second-order perturbation theory analysis of the Fock-matrix in the NBO basis. To achieve comparable results, we considered the same Lewis description regarding donor–acceptor interactions around the tin center (specified with the “CHOOSE” command in the NBO).

As the E^(2)^ values are negligible for most of the neutral complexes, in the following we will focus on selected cationic examples (see Figure 6). The two lone pairs of chalcogen atoms donate to the tin center with differing strengths. The lone pair with practically pure p-character (higher than 99%) forms a stronger donor–acceptor interaction, which intensifies slightly when descending the group (*E* ^(2)^ = −82.0, −86.5 and −87.8 kcal/mol for **1** ^**O+**^, **1** ^**S+**^ and **1** ^**Se+**^, respectively, Figure 6b, e, h). This trend agrees with the WBI values (describing the covalency of the bond), as the donor ability increases substantially in the O < S < Se direction. On the contrary, the other lone pair at the same Y center, which has a substantial s-character (62.3%, 80.1% and 85.2% for Y = O, S, Se, respectively), also establishes an orbital interaction, however, with less significant E^(2)^ energies (−20.1, −12.5 and −12.0 kcal/mol, respectively). The latter orbital interaction markedly contrasts those found in the P-donor complexes (**1** ^**+**^ **-3** ^**+**^): while the lone pairs with high s-character at the P centers (55.4%) can establish an effective coordination toward the tin center in the five-membered ring, the donation from a similar lone pair of the chalcogen centers is restricted due to the poorer orbital overlap, as a consequence of the six-membered ring (Figure 6c, f, i). By summing up the contributions of each orbital interaction (−184.3, −307.5 and −362.1 kcal/mol, for **1** ^**O+**^, **1** ^**S+**^ and **1** ^**Se+**^, respectively) the observed tendency fails to explain the trend in the total interaction energies. Therefore, we sought additional stabilization effects that may contribute to the differing Δ*E*_int_ values.

Indeed, the electrostatic interactions that arise between the Y and Sn centers also change significantly upon descending group 16. Although energy decomposition analysis schemes would be preferable to quantify such interactions, in the present cases the fragmentation of the complexes is not reasonable due to the chelating nature of the ligand. Hence, we explored the NPA partial charges at the Sn and Y centers in complexes (**1** ^**Y+**^-**3** ^**Y+**^).

In line with the shrinking electronegativities of the chalcogen centers (EN = 3.44 > 2.58 > 2.55 for O, S and Se, respectively), their NPA charges gradually become less negative in the series **1** ^**O+**^< **1** ^**S+**^ < **1** ^**Se+**^ (−1.16 e < −0.52 e < −0.35 e). Parallelly, the charges at the tin center also decrease (1.94 *e* > 1.54 *e* > 1.44 e, for **1** ^**O+**^, **1** ^**S+**^ and **1** ^**Se+**^, respectively) and consequently the electrostatic nature of the interaction becomes less intense in the direction O > S > Se. In summary, the trend of the Δ*E*_int_ values is primarily governed by the strength of electrostatic interactions, which overrules the effect of orbital interactions.

## Conclusions

An extensive set of *Y,C,Y*-chelated organotin­(IV) compounds and cations (Y = O, S or Se), bearing entirely new types of pincer ligands, was synthesized using a straightforward and easy procedure based on the oxidation of *t*Bu_2_P functions in the parent compounds. Importantly, the derivatization of the ligand system is cleanly achieved in the presence of already attached metal center, without the need to synthesize new metal-free ligand precursors. This process not only switched between donor atoms from phosphorus to corresponding chalcogens but also resulted in extension from the 5-membered to 6-membered chelating ring, which definitely influenced the coordination behavior of these ligands. Therefore, remarkable coordination variability was obtained. The tin cations preferred a distorted trigonal bipyramidal array with Y atoms located in the axial positions, whereas a variety of coordination modes ranging from six coordinated octahedral, to five coordinated trigonal bipyramidal or almost ideal tetragonal pyramidal (including distorted intermediate cases) were discovered for neutral compounds.

The total stability of the complexes involved in our study was found to be influenced by the following: (i) the type of the chalcogen center, (ii) the charge of the complex (neutral or cationic), and (iii) the Lewis acidity of the tin center. In general, the covalency of the Y → Sn interaction increases in the O < S < Se direction. The observed relationship between the total interaction and second order perturbation energies reveals the role of the electrostatics in the stabilization of the oxygen-containing complexes. As the coordination sphere around the tin center is relatively saturated, the strengths of the formed Y → Sn interactions are limited. In neutral complexes, only a weaker dative or a σ-hole interaction can be formed, while in cationic complexes, parallel with the increasing Lewis acidity of the tin center, two relatively strong Y → Sn dative bonds are established in all cases.

Low-valent compounds of group 14 containing parent *P,C,P*- and oxidized *Y,C,Y*-pincers (Y = O, S, Se) are currently being intensively investigated by our group, and at the same time we are looking for other applications of these ligands in the realm of other *p*-block elements.

## Experimental section

### Hazards

No uncommon hazards are noted.

### General Procedures

All manipulations were carried out under an argon atmosphere using Schlenk tube technique. Solvents were dried using Pure Solv–Innovative Technology equipment. Deuterated solvents were dried and degassed by standard procedures and stored over potassium mirror or molecular sieves. All reagents except those referenced in the main text were purchased from commercial sources and used as delivered. ^1^H and ^13^C­{^1^H} NMR spectra were recorded on Bruker Ascend 500 MHz and Bruker Ultrashield 400 MHz spectrometers, using a 5 mm tunable broad-band probe or a cryo-probe Prodigy at 295 K (if another temperature is not defined). Appropriate chemical shifts in ^1^H and ^13^C­{^1^H} NMR spectra are given relative to the residual signals of the solvent [C_6_D_6_: δ­(^1^H) = 7.16 ppm and δ­(^13^C) = 128.39 ppm, CD_2_Cl_2_: δ­(^1^H) = 5.32 ppm and δ­(^13^C) = 54.0 ppm, CDCl_3_: δ­(^1^H) = 7.27 ppm and δ­(^13^C) = 77.23 ppm, CD_3_CN: δ­(^1^H) = 1.94 ppm and δ­(^13^C) = 1.39 ppm]. ^11^B­{^1^H}, ^19^F­{^1^H}, ^31^P­{^1^H}, ^77^Se­{^1^H} and ^119^Sn­{^1^H} NMR spectra were related to external standards BF_3_.Et_2_O (δ­(^11^B) = 0.00 ppm), CFCl_3_ (δ­(^19^F) = 0.00 ppm), 85% H_3_PO_4_ (δ­(^31^P) = 0.00 ppm), Ph_2_Se_2_ (δ­(^77^Se) = 460.00 ppm) and Me_4_Sn (δ­(^119^Sn) = 0.00 ppm), respectively. Elemental analyses were performed on Flash 2000 CHNS analyzer.

### Syntheses

#### Synthesis of [2,6-(*t*Bu_2_(O)­PO)_2_C_6_H_3_]­SnCl_3_ (1°)

Solution of **1** (170 mg; 0.27 mmol) in dichloromethane (20 mL) was transferred to Schlenk flask with dried molecular sieves. 30% solution of hydrogen peroxide in water (59 μL; 0.58 mmol) was slowly added, while resulting mixture was stirred vigorously. The reaction mixture was stirred for 5 min at room temperature and then molecular sieves were removed by filtration. Solvent was removed in vacuo and white powder was rigorously dried to remove any excess traces of water. Recrystallization from dichloromethane/hexane solution gave colorless crystals of compound **1** ^**O**^. Yield of **1** ^**O**^ was 106 mg, (59%), mp 293 °C. Single-crystals suitable for *sc*-XRD diffraction analysis were obtained from saturated solution using dichloromethane/hexane mixture. Anal. Calcd for C_22_H_39_Cl_3_O_4_P_2_Sn (MW 654.56): C, 40.4; H, 6.0%. Found: C, 40.6; H, 6.3%. ^**1**^ **H NMR** (500 MHz, CDCl_3_) δ (ppm): 1.47 [36H, d, ^3^ *J*(^31^P,^1^H) = 16.0 Hz, *t*Bu_2_(O)­P–C*H*_3_], 6.86 [2H, d, ^3^ *J*(^1^H,^1^H) = 8.2 Hz, ^4^ *J*(^119/117^Sn,^1^H) = 45.6 Hz, Ar-*H*], 7.23 [1H, t, ^3^ *J*(^1^H,^1^H) = 8.2 Hz, Ar-*H*]. ^**13**^ **C­{** ^**1**^ **H} NMR** (125.78 MHz, CDCl_3_) δ (ppm): 27.0 [s, *t*Bu_2_(O)­P-*C*H_3_], 38.0 [d, ^1^ *J*(^31^P,^13^C) = 73.1 Hz, *t*Bu_2_(O)­P-*C*], 117.6 [d, ^3^ *J*(^31^P,^13^C) = 7.2 Hz, ^3^ *J*(^119/117^Sn,^13^C) = 54.3 Hz, Ar-*C*], 130.4 [s, Ar-*C*], 136.6 [s, Ar-*C*] 157.5 [d, ^2^ *J*(^31^P,^13^C) = 12.7 Hz, Ar-*C*]. ^**31**^ **P­{** ^**1**^ **H} NMR** (202.5 MHz, CDCl_3_) δ (ppm): 85.0 [s, ^n^ *J*(^119/117^Sn,^31^P) = 73 Hz]. ^**119**^ **Sn­{** ^**1**^ **H} NMR** (186.5 MHz, CDCl_3_) δ (ppm): −632.6 [t, ^n^ *J*(^119/117^Sn,^31^P) = 73 Hz].

#### Synthesis of [2,6-(*t*Bu_2_(S)­PO)_2_C_6_H_3_]­SnCl_3_ (1^S^)

Elemental sulfur (21 mg; 0.66 mmol) was added in one portion to solution of **1** (366 mg; 0.33 mmol) in dichloromethane (20 mL). The reaction mixture was stirred for 3 days at room temperature and then was concentrated to 1/2 volume. Yellowish solution was layered with hexane. Crystallization at room temperature gave yellow crystals of compound **1** ^**S**^. Yield of **1** ^**S**^ was 128 mg, (57%), mp 234–236 °C. Single-crystals suitable for *sc*-XRD diffraction analysis were obtained from saturated solution using dichloromethane/hexane mixture. Anal. Calcd for C_22_H_39_Cl_3_O_2_P_2_S_2_Sn (MW 686.68): C, 38.5; H, 5.7%. Found: C, 38.7; H, 6.2%. ^**1**^ **H NMR** (500 MHz, CD_2_Cl_2_) δ (ppm): 1.46 [36H, d, ^3^ *J*(^31^P,^1^H) = 17.6 Hz, *t*Bu_2_(S)­P–C*H*_3_], 7.00 [2H, d, ^3^ *J*(^1^H,^1^H) = 8.1 Hz, ^4^ *J*(^119/117^Sn, ^1^H) = 45.5 Hz, Ar-*H*], 7.33 [1H, t, ^3^ *J*(^1^H,^1^H) = 8.1 Hz, Ar-*H*]. ^**13**^ **C­{** ^**1**^ **H} NMR** (125.78 MHz, CD_2_Cl_2_) δ (ppm): 27.5 [s, *t*Bu_2_(S)­P-*C*H_3_], 42.8 [d, ^1^ *J*(^31^P,^13^C) = 46.0 Hz, *t*Bu_2_(S)­P-*C*], 120.4 [d, ^3^ *J*(^31^P,^13^C) = 5.1 Hz, ^3^ *J*(^119/117^Sn,^13^C) = 42.5 Hz, Ar-*C*], 130.8 [s, Ar-*C*], 136.8 [s, Ar-*C*], 157.2 [d, ^1^ *J*(^31^P,^13^C) = 13.8 Hz, Ar-*C*]. ^**31**^ **P­{** ^**1**^ **H} NMR** (202.5 MHz, CD_2_Cl_2_) δ (ppm): 125.6 [s­(br)]. ^**119**^ **Sn­{** ^**1**^ **H} NMR** (186.5 MHz, CD_2_Cl_2_) δ (ppm): −640.3 [s].

#### Synthesis of [2,6-(*t*Bu_2_(Se)­PO)_2_C_6_H_3_]­SnCl_3_ (1^Se^)

Elemental selenium (168 mg; 2.13 mmol) was added in one portion to solution of **1** (265 mg; 0.43 mmol) in dichloromethane (20 mL). The reaction mixture was stirred for 24 h at room temperature and then unreacted selenium was removed by filtration. Resulting yellowish solution was concentrated to 1/2 volume and layered with hexane. Crystallization at room temperature gave yellow crystals of compound **1** ^**Se**^, whereas another batch of crystals could be obtained from the mother liquor by crystallization at −30 °C. Combined yield of **1** ^**Se**^ was 280 mg, (84%), mp 201–204 °C. Single-crystals suitable for *sc*-XRD diffraction analysis were obtained from saturated dichloromethane solution at 5 °C. Anal. Calcd for C_22_H_39_Cl_3_O_2_P_2_Se_2_Sn (MW 780.50): C, 33.9; H, 5.0%. Found: C, 33.8; H, 4.7%. ^**1**^ **H NMR** (500 MHz, CDCl_3_) δ (ppm): 1.50 [36H, d, ^2^ *J*(^31^P,^1^H) = 1.8 Hz, *t*Bu_2_(Se)­P–C*H*_3_], 6.96 [2H, d, ^3^ *J*(^1^H,^1^H) = 7.8 Hz, ^3^ *J*(^119/117^Sn,^1^H) = 41.9 Hz, Ar-*H*], 7.29 [1H, t, ^3^ *J*(^1^H,^1^H) = 7.8 Hz, Ar-*H*]. ^**13**^ **C­{** ^**1**^ **H} NMR** (125.78 MHz, CDCl_3_) δ (ppm): 27.6 [d, ^3^ *J*(^31^P,^13^C) = 4.9 Hz, *t*Bu_2_(Se)­P-*C*H_3_], 43.1 [d, ^1^ *J*(^31^P,^13^C) = 35.5 Hz *t*Bu_2_(Se)­P-*C*], 120.4 [d, ^3^ *J*(^31^P,^13^C) = 4.2 Hz, ^3^ *J*(^119/117^Sn,^13^C) = 38.1 Hz, Ar-*C*], 129.6 [s, Ar-*C*], 133.1 [s, Ar-*C*], 157.0 [d, ^2^ *J*(^31^P,^13^C) = 13.6 Hz, Ar-*C*]. ^**31**^ **P­{** ^**1**^ **H} NMR** (202.5 MHz, CDCl_3_) δ (ppm): 131.0 [s, ^1^ *J*(^77^Se,^31^P) = 621 Hz]. ^**77**^ **Se­{** ^**1**^ **H} NMR** (95.4 MHz, CDCl_3_) δ (ppm): −2.8 [d, ^1^ *J*(^77^Se,^31^P) = 621 Hz]. ^**119**^ **Sn­{** ^**1**^ **H} NMR** (186.5 MHz, CDCl_3_) δ (ppm): −773.4 [s­(br)].

#### Synthesis of {[2,6-(*t*Bu_2_(O)­PO)_2_C_6_H_3_]­SnCl_2_}­{[B­[3,5-(CF_3_)_2_C_6_H_3_]_4_} (1^O+^[BArF]^−^)

Solid Na­[BArF] (42 mg; 0.05 mmol) was added in one portion to solution of **1** ^**O**^ (31 mg; 0.05 mmol) in dichloromethane (10 mL). The reaction mixture was stirred for 1 h at room temperature and then incipient NaCl was removed by filtration. Resulting yellowish solution was layered with hexane. Crystallization at room temperature gave colorless crystals of compound **1** ^**O+**^ **[BArF]** ^**–**^. Yield of **1** ^**O+**^ **[BArF]** ^**–**^ was 58 mg, (83%), mp 218–221 °C. Single-crystals suitable for *sc*-XRD diffraction analysis were obtained by slow diffusion of hexane into saturated dichloromethane solution at room temperature. Anal. Calcd for C_54_H_51_BCl_2_F_24_O_4_P_2_Sn (MW 1480.23): C, 43.8; H, 3.5%. Found: C, 44.0; H, 3.8%. ^**1**^ **H NMR** (500 MHz, CDCl_3_) δ (ppm): 1.37 [36H, t, ^3^ *J*(^31^P,^1^H) = 16.8 Hz, *t*Bu_2_P­(O)–C*H*_3_], 6.98 [2H, d, ^3^ *J*(^1^H,^1^H) = 8.1 Hz, ^3^ *J*(^119/117^Sn,^1^H) = 49.1 Hz, Ar-*H*], 7.37 [1H, t, ^3^ *J*(^1^H,^1^H) = 8.1 Hz, Ar-*H*], 7.52 [4H, s, Ar-*H*], 7.71 [8H, s­(br), Ar-*H*]. ^**11**^ **B­{** ^**1**^ **H} NMR** (160.42 MHz, CDCl_3_) δ (ppm): −6.6 [s]. ^**13**^ **C­{** ^**1**^ **H} NMR** (125.78 MHz, CDCl_3_) δ (ppm): 25.8 [s, *t*Bu_2_(O)­P-*C*H_3_], 38.2 [d, ^1^ *J*(^31^P, ^13^C) = 67.8 Hz, *t*Bu_2_(O)­P-*C*], 117.6 [m, Ar-*C*], 119.4 [d, ^3^ *J*(^31^P,^13^C) = 6.6 Hz, ^3^ *J*(^119/117^Sn,^13^C) = 51.8 Hz, Ar-*C*], 124.7 [q, ^1^ *J*(^19^F,^13^C) = 272 Hz, *C*F_3_], 128.8 [s, Ar-*C*], 129.0 [qq, ^2^ *J*(^19^F,^13^C) = 31.6 Hz, ^4^ *J*(^19^F,^13^C) = 3.0 Hz, Ar-*C*], 134.9 [s, Ar-*C*], 136.6 [s, Ar-*C*], 159.1 [d, ^2^ *J*(^31^P,^13^C) = 11.8 Hz, Ar-*C*], 161.8 [q, ^1^ *J*(^13^C,^11^B) = 50 Hz, Ar-*C*]. ^**19**^ **F­{** ^**1**^ **H} NMR** (376.3 MHz, CDCl_3_) δ (ppm): −62.4 [s]. ^**31**^ **P­{** ^**1**^ **H} NMR** (202.5 MHz, CDCl_3_) δ (ppm): 91.8 [s, ^n^ *J*(^119/117^Sn,^31^P) = 73.6 Hz]. ^**119**^ **Sn­{** ^**1**^ **H} NMR** (186.5 MHz, CDCl_3_) δ (ppm): −393.1 [t, ^n^ *J*(^119/117^Sn,^31^P) = 73.6 Hz].

#### Synthesis of {[2,6-(*t*Bu_2_(S)­PO)_2_C_6_H_3_]­SnCl_2_}­{[B­[3,5-(CF_3_)_2_C_6_H_3_]_4_} (1^S+^[BArF]^−^)

Solid Na­[BArF] (107 mg; 0.12 mmol) was added in one portion to solution of **1** ^**S**^ (83 mg; 0.12 mmol) in dichloromethane (10 mL). The reaction mixture was stirred for 1 h at room temperature and then incipient NaCl was removed by filtration. Resulting yellowish solution was layered with hexane. Crystallization at room temperature gave yellowish crystals of compound **1** ^**S+**^ **[BArF]** ^**–**^. Yield of **1** ^**S+**^ **[BArF]** ^**–**^ was 115 mg, (63%), mp 249–253 °C. Single-crystals suitable for *sc*-XRD diffraction analysis were obtained by slow diffusion of hexane into saturated dichloromethane solution at room temperature. Anal. Calcd for C_54_H_51_BCl_2_F_24_O_2_P_2_S_2_Sn (MW 1514.45): C, 42.8; H, 3.4%. Found: C, 43.1; H, 3.8%. ^**1**^ **H NMR** (500 MHz, CD_2_Cl_2_) δ (ppm): 1.44 [36H, t, ^3^ *J*(^31^P,^1^H) = 18.8 Hz, *t*Bu_2_P­(S)–C*H*_3_], 7.29 [2H, m, Ar-*H*], 7.56 [4H, s­(br), Ar-*H*], 7.72 [9H, m­(br), Ar-*H*]. ^**11**^ **B­{** ^**1**^ **H} NMR** (160.42 MHz, CD_2_Cl_2_) δ (ppm): −5.0 [s]. ^**13**^ **C­{** ^**1**^ **H} NMR** (125.78 MHz, CD_2_Cl_2_) δ (ppm): 27.3 [s, *t*Bu_2_(S)­P-*C*H_3_], 43.4 [d, ^1^ *J*(^31^P, ^13^C) = 40.8 Hz, *t*Bu_2_(S)­P-*C*], 118.0 [m, Ar-*C*], 122.4 [d, ^3^ *J*(^31^P,^13^C) = 3.6 Hz, ^3^ *J*(^119/117^Sn,^13^C) = 45 Hz, Ar-*C*], 125.1 [q, ^1^ *J*(^19^F,^13^C) = 272 Hz, *C*F_3_], 128.8 [s, Ar-*C*], 129.3 [qq, ^2^ *J*(^19^F,^13^C) = 31.6 Hz, ^4^ *J*(^19^F,^13^C) = 3.0 Hz, Ar-*C*], 135.3 [s, Ar-*C*], 135.9 [s, Ar-*C*], 157.5 [d, ^2^ *J*(^31^P,^13^C) = 14.0 Hz, Ar-*C*], 162.2 [q, ^1^ *J*(^13^C,^11^B) = 50 Hz, Ar-*C*]. ^**19**^ **F­{** ^**1**^ **H} NMR** (376.3 MHz, CD_2_Cl_2_) δ (ppm): −62.8 [s]. ^**31**^ **P­{** ^**1**^ **H} NMR** (202.5 MHz, CD_2_Cl_2_) δ (ppm): 133.0 [s]. ^**119**^ **Sn­{** ^**1**^ **H} NMR** (186.5 MHz, CD_2_Cl_2_) δ (ppm): −371.1 [s].

#### Synthesis of {[2,6-(*t*Bu_2_(Se)­PO)_2_C_6_H_3_]­SnCl_2_}­{[B­[3,5-(CF_3_)_2_C_6_H_3_]_4_} (1^Se+^[BArF]^−^)

Solid Na­[BArF] (209 mg; 0.24 mmol) was added in one portion to solution of **1** ^**Se**^ (184 mg; 0.24 mmol) in dichloromethane (15 mL). The reaction mixture was stirred for 30 min at room temperature and then incipient NaCl was removed by filtration. Resulting yellowish solution was layered with hexane. Crystallization at room temperature gave yellow crystals of compound **1** ^**Se+**^ **[BArF]** ^**–**^. Yield of **1** ^**Se+**^ **[BArF]** ^**–**^ was 312 mg, (82%), mp 206–208 °C. Single-crystals suitable for *sc*-XRD diffraction analysis were obtained by slow diffusion of hexane into saturated dichloromethane solution at room temperature. Anal. Calcd for C_54_H_51_BCl_2_F_24_O_2_P_2_Se_2_Sn (MW 1608.27): C, 40.3; H, 3.2%. Found: C, 40.4; H, 3.0%. ^**1**^ **H NMR** (500 MHz, CD_3_CN) δ (ppm): 1.46 [36H, t, ^3^ *J*(^31^P,^1^H) = 18.4 Hz, *t*Bu_2_(Se)­P–C*H*_3_], 7.33 [2H, d, ^3^ *J*(^1^H,^1^H) = 8.3 Hz, ^3^ *J*(^119/117^Sn,^1^H) = 48.6 Hz, Ar-*H*], 7.58 [1H, t, ^3^ *J*(^1^H,^1^H) = 8.3 Hz, Ar-*H*], 7.67 [4H, s, Ar-*H*], 7.69 [8H, s­(br), Ar-*H*]. ^**11**^ **B­{** ^**1**^ **H} NMR** (160.42 MHz, CD_3_CN) δ (ppm): −6.7 [s]. ^**13**^ **C­{** ^**1**^ **H} NMR** (125.78 MHz, CD_3_CN) δ (ppm): 27.2 [s, *t*Bu_2_(Se)­P-*C*H_3_], 44.1 [d, ^1^ *J*(^31^P,^13^C) = 31.2 Hz *t*Bu_2_(Se)­P-*C*], 118.8 [m, Ar-*C*], 123.1 [d, ^3^ *J*(^31^P,^13^C) = 4.9 Hz, ^3^ *J*(^119/117^Sn,^13^C) = 37.6 Hz, Ar-*C*], 125.6 [q, ^1^ *J*(^19^F,^13^C) = 272 Hz, *C*F_3_], 130.0 [qq, ^2^ *J*(^19^F,^13^C) = 31.5 Hz, ^4^ *J*(^19^F,^13^C) = 2.8 Hz, Ar-*C*], 133.9 [s, Ar-*C*], 135.7 [s, Ar-*C*], 157.8 [d, ^2^ *J*(^31^P,^13^C) = 13.7 Hz, Ar-*C*], 162.7 [q, ^1^ *J*(^13^C,^11^B) = 49.6 Hz, Ar-*C*]. ^**19**^ **F­{** ^**1**^ **H} NMR** (376.3 MHz, CD_3_CN) δ (ppm): −63.2 [s]. ^**31**^ **P­{** ^**1**^ **H} NMR** (202.5 MHz, CD_3_CN) δ (ppm): 134.4 [s, ^1^ *J*(^77^Se,^31^P) = 576 Hz]. ^**77**^ **Se­{** ^**1**^ **H} NMR** (95.4 MHz, CD_3_CN) δ (ppm): 6.6 [d, ^1^ *J*(^77^Se,^31^P) = 576 Hz]. ^**119**^ **Sn­{** ^**1**^ **H} NMR** (186.5 MHz, CD_3_CN) δ (ppm): −692.1 [s­(br)].

#### Synthesis of [2,6-(*t*Bu_2_(O)­PO)_2_C_6_H_3_]­SnPhCl_2_ (2°)

Solution of **2** (237 mg; 0.36 mmol) in dichloromethane (20 mL) was transferred to Schlenk flask with dried molecular sieves. 30% solution of hydrogen peroxide in water (76 μL; 0.7465 mmol) was slowly added, while resulting mixture was stirred vigorously. The reaction mixture was stirred for 5 min at room temperature and then molecular sieves were removed by filtration. Solvent was removed in vacuo and white powder was rigorously dried to remove any excess traces of water. Recrystallization from dichloromethane/hexane solution gave colorless crystals of compound **2** ^**O**^. Yield of **2** ^**O**^ was 221 mg, (89%), mp 218–222 °C. Single-crystals suitable for *sc*-XRD diffraction analysis were obtained from saturated solution using dichloromethane/hexane mixture at −30 °C. Anal. Calcd for C_28_H_44_Cl_2_O_4_P_2_Sn (MW 696.21): C, 48.3; H, 6.4%. Found: C, 48.6; H, 6.6%. ^**1**^ **H NMR** (500 MHz, CDCl_3_) δ (ppm): 1.50 [36H, d, ^3^ *J*(^31^P,^1^H) = 15.4 Hz, *t*Bu_2_P­(O)–C*H*_3_], 6.91 [2H, d, ^3^ *J*(^1^H,^1^H) = 8.1 Hz, ^4^ *J*(^119/117^Sn, ^1^H) = 40.3 Hz, Ar-*H*], 7.23 [1H, t, ^3^ *J*(^1^H,^1^H) = 8.1 Hz, Ar-*H*], 7.30 [1H, m, Ar-*H*], 7.37 [2H, t, ^3^ *J*(^1^H,^1^H) = 7.4 Hz, Ar-*H*], 8.08 [2H, d, ^3^ *J*(^1^H,^1^H) = 6.9 Hz, ^3^ *J*(^119/117^Sn, ^1^H) = 141.1 Hz Ar-*H*]. ^**13**^ **C­{** ^**1**^ **H} NMR** (125.78 MHz, CDCl_3_) δ (ppm): 27.4 [s, *t*Bu_2_P­(O)-*C*H_3_], 37.8 [d, ^1^ *J*(^31^P,^13^C) = 74.0 Hz, *t*Bu_2_P­(O)-*C*], 118.3 [d, ^3^ *J*(^31^P,^13^C) = 6.8 Hz, ^3^ *J*(^119/117^Sn, ^13^C) = 53.6 Hz, Ar-*C*], 127.8 [s, ^3^ *J*(^119/117^Sn,^13^C) = 153.0/144.6 Hz, Ar-*C*], 128.1 [s, ^4^ *J*(^119/117^Sn, ^13^C) = 29.9 Hz, Ar-*C*], 130.0 [s, Ar-*C*], 133.6 [s, ^2^ *J*(^119/117^Sn,^13^C) = 85.8/81.5 Hz, Ar-*C*], 137.5 [s, Ar-*C*], 157.1 [t, ^3^ *J*(^31^P,^13^C) = 3.7 Hz, Ar-*C*], 157.4 [d, ^2^ *J*(^31^P,^13^C) = 10.9 Hz, Ar-*C*]. ^**31**^ **P­{** ^**1**^ **H} NMR** (202.5 MHz, CDCl_3_) δ (ppm): 82.9 [s, ^n^ *J*(^119/117^Sn, ^31^P) = 66.9 Hz]. ^**119**^ **Sn­{** ^**1**^ **H} NMR** (186.5 MHz, CDCl_3_) δ (ppm): −529.5 [t­(br), ^n^ *J*(^119/117^Sn, ^31^P) = 66.9 Hz].

#### Synthesis of [2,6-(*t*Bu_2_(S)­PO)_2_C_6_H_3_]­SnPhCl_2_ (2^S^)

Elemental sulfur (35 mg; 1.09 mmol) was added in one portion to a solution of **2** (366 mg; 0.55 mmol) in dichloromethane (20 mL). The reaction mixture was stirred for 24 h at room temperature and then concentrated to 1/2 of the original volume. Colorless solution was layered with hexane. Crystallization at room temperature gave colorless crystals of compound **2** ^**S**^ and another batch of crystals could be obtained from mother liquor by crystallization at −30 °C. Combined yield of **2** ^**S**^ was 342 mg, (85%), mp 179–182 °C. Single-crystals suitable for *sc*-XRD diffraction analysis were obtained by slow diffusion of hexane into saturated dichloromethane solution at room temperature. Anal. Calcd for C_28_H_44_Cl_2_O_2_P_2_S_2_Sn (MW 728.34): C, 46.2; H, 6.1%. Found: C, 46.0; H, 6.3%. ^**1**^ **H NMR** (500 MHz, C_6_D_6_) δ (ppm): 1.16 [36H, d, ^3^ *J*(^31^P,^1^H) = 16.6 Hz, *t*Bu_2_P­(S)–C*H*_3_], 6.85 [1H, t, ^3^ *J*(^1^H,^1^H) = 8.3 Hz, Ar-*H*], 7.02 [1H, t, ^3^ *J*(^1^H,^1^H) = 7.7 Hz, Ar-*H*], 7.08 [2H, t, ^3^ *J*(^1^H,^1^H) = 7.6 Hz, Ar-*H*], 7.53 [2H, d­(br), ^3^ *J*(^1^H,^1^H) = 6.9 Hz, Ar-*H*], 8.21 [4H, d, ^3^ *J*(^1^H,^1^H) = 7.2 Hz, ^2^ *J*(^119/117^Sn, ^1^H) = 102.5 Hz, Ar-*H*]. ^**13**^ **C­{** ^**1**^ **H} NMR** (125.78 MHz, C_6_D_6_) δ (ppm): 28.1 [s, *t*Bu_2_P­(S)-*C*H_3_], 42.4 [d, ^1^ *J*(^31^P,^13^C) = 53.0 Hz, *t*Bu_2_P­(S)-*C*], 119.8 [d, ^3^ *J*(^31^P,^13^C) = 4.5 Hz, ^3^ *J*(^119/117^Sn, ^13^C) = 41.9 Hz, Ar-*C*], 129.4 [s, ^3^ *J*(^119/117^Sn,^13^C) = 106.2 Hz, Ar-*C*], 130.7 [s, ^4^ *J*(^119/117^Sn, ^13^C) = 21.2 Hz, Ar-*C*], 131.8 [s, Ar-*C*], 136.2 [s, ^2^ *J*(^119/117^Sn,^13^C) = 68.4 Hz, Ar-*C*], 158.4 [d, ^2^ *J*(^31^P,^13^C) = 11.5 Hz, Ar-*C*]. ^**31**^ **P­{** ^**1**^ **H} NMR** (202.5 MHz, C_6_D_6_) δ (ppm): 132.9 [s]. ^**119**^ **Sn­{** ^**1**^ **H} NMR** (186.5 MHz, C_6_D_6_) δ (ppm): −217.3 [s].

#### Synthesis of [2,6-(*t*Bu_2_(Se)­PO)_2_C_6_H_3_]­SnPhCl_2_ (2^Se^)

Elemental selenium (275 mg; 3.48 mmol) was added in one portion to solution of **2** (462 mg; 0.70 mmol) in dichloromethane (20 mL). The reaction mixture was stirred for 1 h at room temperature and then unreacted selenium was removed by filtration. Resulting colorless solution was concentrated to 1/2 of the original volume and was layered with hexane. Crystallization at room temperature gave colorless crystals of compound **2** ^**Se**^, and another batch of crystals could be gained from mother liquor by crystallization at −30 °C. Combined yield of **2** ^**Se**^ was 482 mg, (84%), mp 197–199 °C. Single-crystals suitable for *sc*-XRD diffraction analysis were obtained from saturated solution using dichloromethane/hexane mixture at 5 °C. Anal. Calcd for C_28_H_44_Cl_2_O_2_P_2_Se_2_Sn (MW 822.16): C, 40.2; H, 5.4%. Found: C, 40.1; H, 5.7%. ^**1**^ **H NMR** (500 MHz, CDCl_3_) δ (ppm): 1.35 [36H, d, ^3^ *J*(^31^P,^1^H) = 17.2 Hz, *t*Bu_2_(Se)­P–C*H*_3_], 7.41 [4H, m, Ar-*H*], 7.63 [2H, d­(br), ^3^ *J*(^1^H,^1^H) = 8.1 Hz, Ar-*H*], 8.10 [2H, d, ^3^ *J*(^1^H,^1^H) = 7.2 Hz, ^3^ *J*(^119/117^Sn, ^1^H) = 109.0 Hz, Ar-*H*]. ^**13**^ **C­{** ^**1**^ **H} NMR** (125.78 MHz, CDCl_3_) δ (ppm): 28.1 [s, *t*Bu_2_(Se)­P-*C*H_3_], 43.1 [d, ^1^ *J*(^31^P,^13^C) = 40.6 Hz, *t*Bu_2_(Se)­P-*C*], 119.7 [d, ^3^ *J*(^31^P,^13^C) = 3.7 Hz, ^3^ *J*(^119/117^Sn,^13^C) = 41.8 Hz, Ar-*C*], 127.6 [s­(br), Ar-*C*], 128.9 [s, ^3^ *J*(^119/117^Sn,^13^C) = 110.8 Hz, Ar-*C*], 130.3 [s, ^4^ *J*(^119/117^Sn,^13^C) = 22.6 Hz, Ar-*C*], 130.8 [s, Ar-*C*], 135.2 [s, ^2^ *J*(^119/117^Sn,^13^C) = 71.1 Hz, Ar-*C*], 147.8 [s­(br), Ar-*C*], 157.2 [d, ^2^ *J*(^31^P,^13^C) = 11.6 Hz, Ar-*C*]. ^**31**^ **P­{** ^**1**^ **H} NMR** (202.5 MHz, CDCl_3_) δ (ppm): 141.9 [s, ^1^ *J*(^77^Se,^31^P) = 733 Hz]. ^**77**^ **Se­{** ^**1**^ **H} NMR** (95.4 MHz, CDCl_3_) δ (ppm): −222.2 [d, ^1^ *J*(^77^Se,^31^P) = 733 Hz]. ^**119**^ **Sn­{** ^**1**^ **H} NMR** (186.5 MHz, CDCl_3_) δ (ppm): −264.3 [s­(br)].

#### Synthesis of {[2,6-(*t*Bu_2_(O)­PO)_2_C_6_H_3_]­SnPhCl}­{[B­[3,5-(CF_3_)_2_C_6_H_3_]_4_} (2^O+^[BArF]^−^)

Solid Na­[BArF] (183 mg; 0.21 mmol) was added in one portion to solution of **2** ^**O**^ (144 mg; 0.21 mmol) in dichloromethane (10 mL). The reaction mixture was stirred for 30 min at room temperature and then incipient NaCl was removed by filtration. Solvent was removed in vacuo and resulting powder was washed with hexane (10 mL). Colorless powder of compound **2** ^**O+**^ **[BArF]** ^**–**^ was obtained. Yield of **2** ^**O+**^ **[BArF]** ^**–**^ was 287 mg, (91%), mp 204–207 °C. Single-crystals suitable for *sc*-XRD diffraction analysis were obtained from saturated dichloromethane solution at −30 °C. Anal. Calcd for C_60_H_56_BClF_24_O_4_P_2_Sn (MW 1523.98): C, 47.3; H, 3.7%. Found: C, 47.8; H, 4.3%. ^**1**^ **H NMR** (500 MHz, CDCl_3_) δ (ppm): 1.19 [18H, d, ^3^ *J*(^31^P,^1^H) = 16.3 Hz, *t*Bu_2_P­(O)–C*H*_3_], 1.37 [18H, d, ^3^ *J*(^31^P,^1^H) = 16.6 Hz, *t*Bu_2_P­(O)–C*H*_3_], 7.06 [2H, d, ^3^ *J*(^1^H,^1^H) = 8.3 Hz, ^4^ *J*(^119/117^Sn,^1^H) = 32.1 Hz, Ar-*H*], 7.45 [1H, t, ^3^ *J*(^1^H,^1^H) = 8.3 Hz, Ar-*H*], 7.53 [7H, m, Ar-*H*], 7.72 [8H, s, Ar-*H*], 7.77 [2H, m, ^3^ *J*(^119/117^Sn, ^1^H) = 108.2 Hz, Ar-*H*]. ^**11**^ **B­{** ^**1**^ **H} NMR** (160.42 MHz, CDCl_3_) δ (ppm): −6.6 [s]. ^**13**^ **C­{** ^**1**^ **H} NMR** (125.78 MHz, CDCl_3_) δ (ppm): 26.0 [s, *t*Bu_2_P­(O)-*C*H_3_], 26.4 [s, *t*Bu_2_P­(O)-*C*H_3_], 37.8 [d, ^1^ *J*(^31^P,^13^C) = 70.3 Hz *t*Bu_2_P­(O)-*C*], 117.7 [m, Ar-*C*], 119.5 [d, ^3^ *J*(^31^P,^13^C) = 6.5 Hz, ^3^ *J*(^119/117^Sn,^13^C) = 38.5 Hz, Ar-*C*], 120.2 [s, Ar-*C*], 124.8 [q, ^1^ *J*(^19^F,^13^C) = 273 Hz, *C*F_3_], 129.2 [qq, ^2^ *J*(^19^F,^13^C) = 32.0 Hz, ^4^ *J*(^19^F,^13^C) = 3.0 Hz, Ar-*C*], 130.0 [s, ^3^ *J*(^119/117^Sn,^13^C) = 116.0 Hz, Ar-*C*], 132.2 [s, ^4^ *J*(^119/117^Sn,^13^C) = 22.7 Hz, Ar-*C*], 134.9 [s, ^2^ *J*(^119/117^Sn, ^13^C) = 69.0 Hz, Ar–C], 135.0 [s, Ar-*C*], 135.5 [s, Ar-*C*], 139.6 [s, Ar-*C*]; 158.8 [d, ^2^ *J*(^31^P,^13^C) = 11.1 Hz, Ar–C]; 161.9 [q, ^1^ *J*(^13^C,^11^B) = 49.8 Hz, Ar-*C*]. ^**19**^ **F­{** ^**1**^ **H} NMR** (376.3 MHz, CDCl_3_) δ (ppm): −62.8 [s]. ^**31**^ **P­{** ^**1**^ **H} NMR** (202.5 MHz, CDCl_3_) δ (ppm): 87.4 [s, ^n^ *J*(^119/117^Sn, ^31^P) = 54.5 Hz]. ^**119**^ **Sn­{** ^**1**^ **H} NMR** (186.5 MHz, CDCl_3_) δ (ppm): −345.0 [t, ^n^ *J*(^119/117^Sn, ^31^P) = 54.5 Hz].

#### Synthesis of {[2,6-(*t*Bu_2_(S)­PO)_2_C_6_H_3_]­SnPhCl}­{[B­[3,5-(CF_3_)_2_C_6_H_3_]_4_} (2^S+^[BArF]^−^)

Solid Na­[BArF] (136 mg; 0.15 mmol) was added in one portion to solution of **2** ^**S**^ (112 mg; 0.15 mmol) in dichloromethane (10 mL). The reaction mixture was stirred for 30 min at room temperature and then incipient NaCl was removed by filtration. Colorless solution was concentrated to 1/2 of the original volume and layered with hexane. Crystallization at room temperature gave colorless crystals of compound **2** ^**S+**^ **[BArF]** ^**–**^. Yield of **2** ^**S+**^ **[BArF]** ^**–**^ was 201 mg, (84%), mp 178–181 °C. Single-crystals suitable for *sc*-XRD diffraction analysis were obtained by slow diffusion of hexane into saturated dichloromethane solution at room temperature. Anal. Calcd for C_60_H_56_BClF_24_O_2_P_2_S_2_Sn (MW 1556.11): C, 46.3; H, 3.6%. Found: C, 46.2; H, 3.4%. ^**1**^ **H NMR** (500 MHz, CD_2_Cl_2_) δ (ppm): 1.24 [18H, d, ^3^ *J*(^31^P,^1^H) = 17.8 Hz, *t*Bu_2_P­(S)–C*H*_3_], 1.47 [18H, d, ^3^ *J*(^31^P,^1^H) = 17.9 Hz, *t*Bu_2_P­(S)–C*H*_3_], 7.35 [2H, d, ^3^ *J*(^1^H,^1^H) = 8.5 Hz, ^4^ *J*(^119/117^Sn,^1^H) = 33.5 Hz, Ar-*H*], 7.56 [3H, m, Ar-*H*], 7.60 [4H, s, Ar-*H*], 7.72 [1H, t, ^3^ *J*(^1^H,^1^H) = 8.5 Hz, Ar-*H*], 7.76 [8H, s, Ar-*H*], 7.83 [2H, m, ^3^ *J*(^119/117^Sn, ^1^H) = 102.4 Hz, Ar-*H*]. ^**11**^ **B­{** ^**1**^ **H} NMR** (160.42 MHz, CD_2_Cl_2_) δ (ppm): −7.2 [s]. ^**13**^ **C­{** ^**1**^ **H} NMR** (125.78 MHz, CD_2_Cl_2_) δ (ppm): 27.2 [s, *t*Bu_2_P­(S)-*C*H_3_], 27.6 [s, *t*Bu_2_P­(S)-*C*H_3_], 42.9 [d, ^1^ *J*(^31^P, ^13^C) = 43.9 Hz, *t*Bu_2_(S)­P-*C*], 118.0 [m, Ar-*C*], 122.4 [d, ^3^ *J*(^31^P,^13^C) = 4.0 Hz, ^3^ *J*(^119/117^Sn,^13^C) = 35.3 Hz, Ar-*C*], 125.1 [q, ^1^ *J*(^19^F,^13^C) = 273 Hz, *C*F_3_], 129.4 [qq, ^2^ *J*(^19^F,^13^C) = 32.0 Hz, ^4^ *J*(^19^F,^13^C) = 3.0 Hz, Ar-*C*], 130.4 [s, ^3^ *J*(^119/117^Sn,^13^C) = 114.2 Hz, Ar-*C*], 132.0 [s, ^4^ *J*(^119/117^Sn,^13^C) = 22.1 Hz, Ar-*C*], 134.3 [s, ^2^ *J*(^119/117^Sn, ^13^C) = 69.9 Hz, Ar–C], 135.2 [s, Ar-*C*], 135.3 [s, Ar-*C*], 146.6 [s, Ar-*C*]; 157.5 [d, ^2^ *J*(^31^P,^13^C) = 13.4 Hz, Ar–C]; 162.3 [q, ^1^ *J*(^13^C,^11^B) = 49.8 Hz, Ar-*C*]. ^**19**^ **F­{** ^**1**^ **H} NMR** (376.3 MHz, CD_2_Cl_2_) δ (ppm): −62.8 [s]. ^**31**^ **P­{** ^**1**^ **H} NMR** (202.5 MHz, CD_2_Cl_2_) δ (ppm): 134.2 [s]. ^**119**^ **Sn­{** ^**1**^ **H} NMR** (186.5 MHz, CD_2_Cl_2_) δ (ppm): −304.4 [s].

#### Synthesis of {[2,6-(*t*Bu_2_(Se)­PO)_2_C_6_H_3_]­SnPhCl}­{[B­[3,5-(CF_3_)_2_C_6_H_3_]_4_} (2^Se+^[BArF]^−^)

Solid Na­[BArF] (410 mg; 0.46 mmol) was added in one portion to solution of **2** ^**Se**^ (380 mg; 0.46 mmol) in dichloromethane (20 mL). The reaction mixture was stirred for 30 min at room temperature and then incipient NaCl was removed by filtration. Colorless solution was concentrated to 1 of the original volume and layered with hexane. Crystallization at room temperature gave colorless crystals of compound **2** ^**Se+**^ **[BArF]** ^**–**^, and another batch of crystals could be obtained from mother liquor by crystallization at −30 °C. Combined yield of **2** ^**Se+**^ **[BArF]** ^**–**^ was 702 mg, (92%), mp 174–176 °C. Single-crystals suitable for *sc*-XRD diffraction analysis were obtained from saturated solution using dichloromethane/hexane mixture at 5 °C. Anal. Calcd for C_60_H_56_BClF_24_O_2_P_2_Se_2_Sn (MW 1649.93): C, 43.7; H, 3.4%. Found: C, 44.0; H, 3.5%. ^**1**^ **H NMR** (500 MHz, CDCl_3_) δ (ppm): 1.18 [18H, d, ^3^ *J*(^31^P,^1^H) = 17.6 Hz, *t*Bu_2_P­(Se)–C*H*_3_], 1.44 [18H, d, ^3^ *J*(^31^P,^1^H) = 18.1 Hz, *t*Bu_2_P­(Se)–C*H*_3_], 7.25 [2H, d, ^3^ *J*(^1^H,^1^H) = 8.2 Hz, ^4^ *J*(^119/117^Sn,^1^H) = 32.4 Hz, Ar-*H*], 7.48 [3H, m, Ar-*H*], 7.55 [5H, m, Ar-*H*], 7.73 [8H, s, Ar-*H*], 7.79 [2H, m, ^3^ *J*(^119/117^Sn, ^1^H) = 111.5 Hz, Ar-*H*]. ^**11**^ **B­{** ^**1**^ **H} NMR** (160.42 MHz, CDCl_3_) δ (ppm): −7.2 [s]. ^**13**^ **C­{** ^**1**^ **H} NMR** (125.78 MHz, CDCl_3_) δ (ppm): 27.1 [s, *t*Bu_2_P­(Se)-*C*H_3_], 27.7 [s, *t*Bu_2_P­(Se)-*C*H_3_], 43.3 [d, ^1^ *J*(^31^P,^13^C) = 33.1 Hz *t*Bu_2_P­(Se)-*C*], 117.7 [m, Ar-*C*], 121.4 [s­(br), Ar-*C*], 122.5 [s, ^3^ *J*(^119/117^Sn,^13^C) = 34.8 Hz, Ar-*C*], 124.8 [q, ^1^ *J*(^19^F,^13^C) = 273 Hz, *C*F_3_], 129.1 [qq, ^2^ *J*(^19^F,^13^C) = 32.0 Hz, ^4^ *J*(^19^F,^13^C) = 3.0 Hz, Ar-*C*], 130.0 [s, ^3^ *J*(^119/117^Sn,^13^C) = 113.9 Hz, Ar-*C*], 131.5 [s, ^4^ *J*(^119/117^Sn,^13^C) = 22.8 Hz, Ar-*C*], 133.7 [s, ^2^ *J*(^119/117^Sn, ^13^C) = 70.1 Hz, Ar–C], 134.0 [s, Ar-*C*], 135.0 [s, Ar-*C*], 146.6 [s, Ar-*C*]; 156.9 [d, ^2^ *J*(^31^P,^13^C) = 13.7 Hz, Ar–C]; 161.9 [q, ^1^ *J*(^13^C,^11^B) = 49.8 Hz, Ar-*C*]. ^**19**^ **F­{** ^**1**^ **H} NMR** (376.3 MHz, CDCl_3_) δ (ppm): −62.3 [s]. ^**31**^ **P­{** ^**1**^ **H} NMR** (202.5 MHz, CDCl_3_) δ (ppm): 141.7 [s, ^1^ *J*(^77^Se, ^31^P) = 635 Hz]. ^**77**^ **Se­{** ^**1**^ **H} NMR** (95.4 MHz, CDCl_3_) δ (ppm): −153.8 [d, ^1^ *J*(^77^Se,^31^P) = 635 Hz, ^1^ *J*(^119/117^Sn, ^77^Se) = 597 Hz,]. ^**119**^ **Sn­{** ^**1**^ **H} NMR** (186.5 MHz, CDCl_3_) δ (ppm): −322.7 [s­(br)].

#### Synthesis of [2,6-(*t*Bu_2_(S)­PO)­C_6_H_3_]­SnPh_2_Cl (3^S^)

Elemental sulfur (38 mg; 1.19 mmol) was added in one portion to solution of **3** (417 mg; 0.59 mmol) in dichloromethane (20 mL). The reaction mixture was stirred for 24 h at room temperature and then was concentrated to 1/2 of the original volume. Colorless solution was layered with hexane. Crystallization at room temperature gave colorless crystals of compound **3** ^**S**^, whereas another batch of crystals could be obtained from mother liquor by crystallization at −30 °C. Combined yield of **3** ^**S**^ was 285 mg, (90%), mp 151–154 °C. Single-crystals suitable for *sc*-XRD diffraction analysis were obtained by slow diffusion of hexane into saturated dichloromethane solution at room temperature. Anal. Calcd for C_34_H_49_ClO_2_P_2_S_2_Sn (MW 769.99): C, 53.0; H, 6.4%. Found: C, 53.4; H, 6.8%. ^**1**^ **H NMR** (500 MHz, C_6_D_6_) δ (ppm): 1.06 [36H, d, ^3^ *J*(^31^P,^1^H) = 16.5 Hz, *t*Bu_2_(S)­P–C*H*_3_], 6.99 [1H, t, ^3^ *J*(^1^H,^1^H) = 8.3 Hz, Ar-*H*], 7.08 [2H, t, ^3^ *J*(^1^H,^1^H) = 7.3 Hz, Ar-*H*], 7.14 [4H, t, ^3^ *J*(^1^H,^1^H) = 7.3 Hz, Ar-*H*], 7.63 [2H, d­(br), ^3^ *J*(^1^H,^1^H) = 8.3 Hz, Ar-*H*], 8.13 [4H, d, ^3^ *J*(^1^H,^1^H) = 7.2 Hz, ^2^ *J*(^119/117^Sn, ^1^H) = 72.5 Hz, Ar-*H*]. ^**13**^ **C­{** ^**1**^ **H} NMR** (125.78 MHz, C_6_D_6_) δ (ppm): 27.6 [s, *t*Bu_2_(S)­P-*C*H_3_], 41.9 [d, ^1^ *J*(^31^P,^13^C) = 54.3 Hz, *t*Bu_2_(S)­P-*C*], 119.2 [d, ^3^ *J*(^31^P,^13^C) = 3.7 Hz, ^3^ *J*(^119/117^Sn,^13^C) = 30.3 Hz, Ar-*C*], 125.9 [t, ^2^ *J*(^31^P,^13^C) = 4.6 Hz, Ar-*C*], 128.6 [s, ^3^ *J*(^119/117^Sn,^13^C) = 72 Hz, Ar-*C*], 129.2 [s, ^4^ *J*(^119/117^Sn,^13^C) = 14.8 Hz, Ar-*C*], 130.7 [s, Ar-*C*], 137.5 [s, ^2^ *J*(^119/117^Sn,^13^C) = 51.9 Hz, Ar-*C*], 145.5 [s, Ar-*C*], 159.0 [d, ^2^ *J*(^31^P,^13^C) = 11.6 Hz, Ar-*C*]. ^**31**^ **P­{** ^**1**^ **H} NMR** (202.5 MHz, C_6_D_6_) δ (ppm): 131.3 [s]. ^**119**^ **Sn­{** ^**1**^ **H} NMR** (186.5 MHz, C_6_D_6_) δ (ppm): −209.2 [s].

#### Synthesis of [2,6-(*t*Bu_2_(Se)­PO)­C_6_H_3_]­SnPh_2_Cl (3^Se^)

Elemental selenium (180 mg; 2.28 mmol) was added in one portion to solution of **3** (320 mg; 0.45 mmol) in dichloromethane (10 mL). The reaction mixture was stirred for 1 h at room temperature and then unreacted selenium was removed by filtration. Resulting colorless solution was concentrated to 1/4 of the original volume and was layered with hexane. Crystallization at −30 °C gave colorless crystals of compound **3** ^**Se**^. Yield of **3** ^**Se**^ was 221 mg, (89%), mp 218–22 °C. Single-crystals suitable for *sc*-XRD diffraction analysis were obtained from saturated solution using dichloromethane/hexane mixture at −30 °C. Anal. Calcd for C_34_H_49_ClO_2_P_2_Se_2_Sn (MW 863.81): C, 47.3; H, 5.7%. Found: C, 47.0; H, 5.8%.^**1**^ **H NMR** (500 MHz, CDCl_3_) δ (ppm): 1.17 [36H, d, ^3^ *J*(^31^P,^1^H) = 17.0 Hz, *t*Bu_2_(Se)­P–C*H*_3_], 7.34 [7H, m, Ar-*H*], 7.68 [2H, d, ^3^ *J*(^1^H,^1^H) = 8.3 Hz, Ar-*H*], 7.86 [4H, d, ^3^ *J*(^1^H,^1^H) = 6.9 Hz, ^2^ *J*(^119/117^Sn, ^1^H) = 74.0 Hz, Ar-*H*]. ^**13**^ **C­{** ^**1**^ **H} NMR** (125.78 MHz, CDCl_3_) δ (ppm): 27.9 [s, *t*Bu_2_(Se)­P-*C*H_3_], 42.9 [d, ^1^ *J*(^31^P,^13^C) = 42.4 Hz, *t*Bu_2_(Se)­P-*C*], 119.4 [d, ^3^ *J*(^31^P,^13^C) = 4.2 Hz, ^3^ *J*(^119/117^Sn,^13^C) = 30.9 Hz, Ar-*C*], 125.7 [7, ^4^ *J*(^31^P,^13^C) = 4.8 Hz, Ar-*C*], 128.5 [s, ^3^ *J*(^119/117^Sn,^13^C) = 77.6 Hz, Ar-*C*], 129.2 [s, ^4^ *J*(^119/117^Sn,^13^C) = 15.7 Hz, Ar-*C*], 130.2 [s, Ar-*C*], 137.0 [s, ^2^ *J*(^119/117^Sn,^13^C) = 51.8 Hz, Ar-*C*], 146.4 [s, Ar-*C*], 158.4 [d, ^2^ *J*(^31^P,^13^C) = 11.9 Hz, Ar-*C*]. ^**31**^ **P­{** ^**1**^ **H} NMR** (202.5 MHz, CDCl_3_) δ (ppm): 141.6 [s, ^1^ *J*(^77^Se,^31^P) = 748 Hz, *t*Bu_2_(Se)*P*]. ^**77**^ **Se­{** ^**1**^ **H} NMR** (95.4 MHz, CDCl_3_) δ (ppm): −252.7 [d, ^1^ *J*(^77^Se,^31^P) = 764 Hz] measured at 323 K at r.t. the signal was too broad to be detected. ^**119**^ **Sn­{** ^**1**^ **H} NMR** (186.5 MHz, CDCl_3_) δ (ppm): −231.4 [s].

#### Synthesis of {[2,6-(*t*Bu_2_(S)­PO)_2_C_6_H_3_]­SnPh_2_}­{[B­[3,5-(CF_3_)_2_C_6_H_3_]_4_} (3^S+^[BArF]^−^)

Solid Na­[BArF] (115 mg; 0.13 mmol) was added in one portion to solution of **3** ^**S**^ (100 mg; 0.13 mmol) in dichloromethane (10 mL). The reaction mixture was stirred for 30 min at room temperature and then incipient NaCl was removed by filtration. Colorless solution was concentrated to 1/2 of the original volume and layered with hexane. Crystallization at room temperature gave colorless crystals of compound **3** ^**S+**^ **[BArF]** ^**–**^. Yield of **3** ^**S+**^ **[BArF]** ^**–**^ was 130 mg, (63%), mp 218–220 °C. Single-crystals suitable for *sc*-XRD diffraction analysis were obtained from saturated solution using dichloromethane/hexane mixture at 5 °C. Anal. Calcd for C_66_H_61_BF_24_O_2_P_2_S_2_Sn (MW 1597.76): C, 49.6; H, 3.9%. Found: C, 49.9; H, 3.8%. ^**1**^ **H NMR** (500 MHz, CD_2_Cl_2_) δ (ppm): 1.15 [36H, d, ^3^ *J*(^31^P,^1^H) = 17.8 Hz, *t*Bu_2_(S)­P–C*H*_3_], 7.31 [2H, d, ^3^ *J*(^1^H,^1^H) = 8.4 Hz, Ar-*H*], 7.48 [6H, m, Ar-*H*], 7.57 [4H, m, Ar-*H*], 7.65 [1H, t, ^3^ *J*(^1^H,^1^H) = 8.4 Hz, Ar-*H*], 7.74 [8H, s, Ar-*H*], 7.77 [4H, m, ^3^ *J*(^119/117^Sn, ^1^H) = 78.8 Hz, Ar-*H*]. ^**11**^ **B­{** ^**1**^ **H} NMR** (160.42 MHz, CD_2_Cl_2_) δ (ppm): −7.0 [s]. ^**13**^ **C­{** ^**1**^ **H} NMR** (125.78 MHz, CD_2_Cl_2_) δ (ppm): 27.2 [s­(br), *t*Bu_2_(S)­P-*C*H_3_], 42.5 [s­(br), *t*Bu_2_(S)­P-*C*], 118.0 [m, Ar-*C*], 121.3 [t, ^n^ *J*(^31^P,^13^C) = 3.5 Hz, Ar-*C*], 122.0 [d, ^3^ *J*(^31^P,^13^C) = 3.6 Hz, ^3^ *J*(^119/117^Sn,^13^C) = 28.5 Hz, Ar-*C*], 125.1 [q, ^1^ *J*(^19^F,^13^C) = 273 Hz, *C*F_3_], 129.4 [qq, ^2^ *J*(^19^F,^13^C) = 31.8 Hz, ^4^ *J*(^19^F,^13^C) = 3.0 Hz, Ar-*C*], 130.0 [s, ^3^ *J*(^119/117^Sn,^13^C) = 82.1 Hz, Ar-*C*], 131.0 [s, ^4^ *J*(^119/117^Sn,^13^C) = 16.5 Hz, Ar-*C*], 134.3 [s, Ar-*C*], 135.3 [s, Ar-*C*], 136.4 [s, ^2^ *J*(^119/117^Sn,^13^C) = 52.5 Hz Ar-*C*], 143.2 [s, ^1^J­(^119/117^Sn,^13^C) = 858/819 Hz, Ar-*C*]; 158.2 [d, ^2^J­(^31^P,^13^C) = 13.2 Hz, Ar–C]; 162.3 [q, ^1^ *J*(^13^C,^11^B) = 50 Hz, Ar-*C*]. ^**19**^ **F­{** ^**1**^ **H} NMR** (376.3 MHz, CD_2_Cl_2_) δ (ppm): −62.8 [s]. ^**31**^ **P­{** ^**1**^ **H} NMR** (202.5 MHz, CD_2_Cl_2_) δ (ppm): 132.6 [s]. ^**119**^ **Sn­{** ^**1**^ **H} NMR** (186.5 MHz, CD_2_Cl_2_) δ (ppm): −298.9 [s].

#### Synthesis of {[2,6-(*t*Bu_2_(Se)­PO)_2_C_6_H_3_]­SnPh_2_}­{[B­[3,5-(CF_3_)_2_C_6_H_3_]_4_} (3^Se+^[BArF]^−^)

Solid Na­[BArF] (78 mg; 0.09 mmol) was added in one portion to solution of **3** ^**Se**^ (81 mg; 0.09 mmol) in dichloromethane (10 mL). The reaction mixture was stirred for 1 h at room temperature and then incipient NaCl was removed by filtration. Colorless solution was concentrated to 1/2 of the original volume and layered with hexane. Crystallization at −30 °C gave colorless crystals of compound **3** ^**Se+**^ **[BArF]** ^**–**^. Yield of **3** ^**Se+**^ **[BArF]** ^**–**^ was 103 mg, (69%), mp 224–226 °C. Single-crystals suitable for *sc*-XRD diffraction analysis were obtained from saturated solution using dichloromethane/hexane mixture at 5 °C. Anal. Calcd for C_66_H_61_BF_24_O_2_P_2_Se_2_Sn (MW 1691.58): C, 46.9; H, 3.6%. Found: C, 47.0; H, 3.8%. ^**1**^ **H NMR** (500 MHz, CDCl_3_) δ (ppm): 1.15 [36H, s­(br), *t*Bu_2_(Se)­P–C*H*_3_], 7.25 [2H, m, Ar-*H*], 7.47 [6H, m, Ar-*H*], 7.54 [5H, m, Ar-*H*], 7.73 [8H, m, Ar-*H*], 7.79 [4H, m, ^3^ *J*(^119/117^Sn, ^1^H) = 80.1 Hz, Ar-*H*]. ^**11**^ **B­{** ^**1**^ **H} NMR** (160.42 MHz, CDCl_3_) δ (ppm): −7.3 [s]. ^**13**^ **C­{** ^**1**^ **H} NMR** (125.78 MHz, CDCl_3_) δ (ppm): 27.3 [s­(br), *t*Bu_2_(Se)­P-*C*H_3_], 43.4 [s­(br), *t*Bu_2_(Se)­P-*C*], 117.7 [m, Ar-*C*], 120.6 [t, ^n^ *J*(^31^P,^13^C) = 3.6 Hz, Ar-*C*], 122.2 [s­(br), ^n^ *J*(^119/117^Sn,^13^C) = 27.3 Hz, Ar-*C*], 124.8 [q, ^1^ *J*(^19^F,^13^C) = 273 Hz, *C*F_3_], 129.1 [qq, ^2^ *J*(^19^F,^13^C) = 31.5 Hz, ^4^ *J*(^19^F,^13^C) = 2.8 Hz, Ar-*C*], 129.6 [s, ^3^ *J*(^119/117^Sn,^13^C) = 81.7 Hz, Ar-*C*], 130.5 [s, ^4^ *J*(^119/117^Sn,^13^C) = 16.7 Hz, Ar-*C*], 133.2 [s, Ar-*C*], 135.0 [s, Ar-*C*], 136.1 [s, ^2^ *J*(^119/117^Sn,^13^C) = 52.6 Hz Ar-*C*], 143.2 [d, ^1^ *J*(^119/117^Sn,^13^C) = 849/812 Hz, Ar-*C*], 157.5 [d, ^n^ *J*(^31^P,^13^C) = 13.5 Hz, Ar-*C*], 161.9 [q, ^1^ *J*(^13^C,^11^B) = 50 Hz, Ar-*C*]. ^**19**^ **F­{** ^**1**^ **H} NMR** (376.3 MHz, CDCl_3_) δ (ppm): −62.4 [s]. ^**31**^ **P­{** ^**1**^ **H} NMR** (202.5 MHz, CDCl_3_) δ (ppm): 141.4 [s, ^1^ *J*(^77^Se,^31^P) = 660 Hz, *t*Bu_2_(Se)*P*]. ^**77**^ **Se­{** ^**1**^ **H} NMR** (95.4 MHz, CDCl_3_) δ (ppm): −207.1 [d, ^1^ *J*(^77^Se,^31^P) = 660 Hz, ^1^ *J*(^119/117^Sn, ^77^Se) = 400/381 Hz]. ^**119**^ **Sn­{** ^**1**^ **H} NMR** (186.5 MHz, CDCl_3_) δ (ppm): −311.7 [s, ^1^ *J*(^119/117^Sn, ^77^Se) = 400 Hz].

#### Synthesis of [2-(*t*Bu_2_(O)­PO)-6-(*t*Bu_2_PO)­C_6_H_3_]­SnPh_2_Cl (3^PO^)

Solution of Me_3_NO (0.110 g; 1.46 mmol) in dichloromethane (10 mL) was added to solution of **3** (1.121 g; 1,46 mmol) in dichloromethane (20 mL). The reaction mixture was stirred for 1 h at room temperature. Resulting colorless solution was concentrated to 1/2 of the original volume and was layered with hexane. Crystallization at room temperature gave colorless crystals of compound **3** ^**PO**^, whereas another batch of crystals could be obtained from mother liquor by crystallization at −30 °C. Combined yield of **3** ^**PO**^ was 1.01 g, (88%), mp 179 °C. Single-crystals suitable for *sc*-XRD diffraction analysis were obtained by slow diffusion of hexane into saturated dichloromethane solution at room temperature. Anal. Calcd for C_34_H_49_ClO_3_P_2_Sn (MW 721.87): C, 56.6; H, 6.8%. Found: C, 56.8; H, 7.1%.^**1**^ **H NMR** (500 MHz, CDCl_3_) δ (ppm): 0.97 [36H, m, *t*Bu_2_P–C*H*_3_ and *t*Bu_2_(O)­P–C*H*_3_], 6.74 [1H, s­(br), Ar-*H*], 7.33 [7H, m, Ar-*H*], 7.67 [1H, t, ^3^ *J*(^1^H,^1^H) = 7.0 Hz, Ar-*H*], 7.93 [4H, d, ^3^ *J*(^1^H,^1^H) = 5.5 Hz, ^2^ *J*(^119/117^Sn, ^1^H) = 72.7 Hz, Ar-*H*]. ^**13**^ **C­{** ^**1**^ **H} NMR** (125.78 MHz, CDCl_3_) δ (ppm): 26.8 [s­(br), *t*Bu_2_(O)­P-*C*H_3_], 27.7 [d, ^2^ *J*(^31^P,^13^C) = 15.7 Hz *t*Bu_2_P-*C*H_3_], 35.7 [d, ^1^ *J*(^31^P,^13^C) = 26.2 Hz, *t*Bu_2_P-*C*], 37.2 [d, ^1^ *J*(^31^P,^13^C) = 75.6 Hz, *t*Bu_2_(O)­P-*C*], 113.4 [s­(br), Ar-*C*], 115.4 [d­(br), ^n^ *J*(^31^P,^13^C) = 29.4 Hz, Ar-*C*], 128.3 [s, ^3^ *J*(^119/117^Sn,^13^C) = 76.5 Hz, Ar-*C*], 128.9 [s, Ar-*C*], 131.4 [s, Ar-*C*], 136.8 [s, ^3^ *J*(^119/117^Sn,^13^C) = 53.4 Hz, Ar-*C*], 145.1 [s­(br), Ar-*C*], 157.9 [s­(br), Ar-*C*], 165.9 [d, ^2^J­(^31^P,^13^C) = 10.8 Hz, Ar–C]. ^**31**^ **P­{** ^**1**^ **H} NMR** (202.5 MHz, CDCl_3_) δ (ppm): 77.1 [s, *t*Bu_2_(O)*P*], 159.2 [s, *t*Bu_2_ *P*]. ^**119**^ **Sn­{** ^**1**^ **H} NMR** (186.5 MHz, CDCl_3_) δ (ppm): −246.9 [s].

#### Synthesis of [2-(*t*Bu_2_(O)­PO)-6-(*t*Bu_2_(S)­PO)­C_6_H_3_]­SnPh_2_Cl (3^OS^)

Elemental sulfur (6.5 mg; 0.2 mmol) was added in one portion to solution of **3** ^**PO**^ (158 mg; 0.2 mmol) in dichloromethane (10 mL). The reaction mixture was stirred for 24 h at room temperature and then was concentrated to 1/2 of the original volume. Colorless solution was layered with hexane. Crystallization at room temperature gave colorless crystals of compound **3** ^**OS**^, whereas another batch of crystals could be obtained from mother liquor by crystallization at −30 °C. Combined yield of **3** ^**OS**^ was 150 mg, (91%), mp 302–304 °C. Anal. Calcd for C_34_H_49_ClO_3_P_2_SSn (MW 753.93): C, 54.2; H, 6.6%. Found: C, 54.0; H, 6.8%.^**1**^ **H NMR** (500 MHz, CDCl_3_) δ (ppm): 0.80 [9H, d­(br), ^3^ *J*(^31^P,^1^H) = 11.3 Hz, *t*Bu_2_P­(O/S)–C*H*_3_], 0.96 [9H, d­(br), ^3^ *J*(^31^P,^1^H) = 13.9 Hz, *t*Bu_2_P­(O/S)–C*H*_3_], 1.13 [9H, d­(br), ^3^ *J*(^31^P,^1^H) = 12.0 Hz, *t*Bu_2_P­(O/S)–C*H*_3_], 1.46 [9H, d­(br), ^3^ *J*(^31^P,^1^H) = 13.9 Hz, *t*Bu_2_P­(O/S)–C*H*_3_], 6.90 [1H, d, ^3^ *J*(^1^H,^1^H) = 8.0 Hz, Ar-*H*], 7.25 [3H, m­(br), Ar-*H*], 7.36 [1H, t, ^3^ *J*(^1^H,^1^H) = 8.1 Hz, Ar-*H*], 7.46 [5H, s­(br), Ar-*H*], 7.97 [1H, d, ^3^ *J*(^1^H,^1^H) = 8.4 Hz, Ar-*H*], 8.23 [2H, s­(br), Ar-*H*]. ^**13**^ **C­{** ^**1**^ **H} NMR**: Reasonable ^13^C NMR spectrum could not be obtained due to a significant line broadening even at low temperature. ^**31**^ **P­{** ^**1**^ **H} NMR** (202.5 MHz, CDCl_3_) δ (ppm): 78.9 [s, *t*Bu_2_ *P*(O)], 132.1 [s, *t*Bu_2_ *P*(S)]. ^**119**^ **Sn­{** ^**1**^ **H} NMR** (186.5 MHz, CDCl_3_) δ (ppm): −258.2 [s].

#### Synthesis of [2-(*t*Bu_2_(O)­PO)-6-(*t*Bu_2_(Se)­PO)­C_6_H_3_]­SnPh_2_Cl (3^OSe^)

Elemental selenium (85 mg; 1.08 mmol) was added in one portion to solution of **3** ^**PO**^ (282 mg; 0.36 mmol) in dichloromethane (20 mL). The reaction mixture was stirred for 24 h at room temperature and then unreacted selenium was removed by filtration. Resulting colorless solution was concentrated to 1/2 of the original volume and was layered with hexane. Crystallization at room temperature gave colorless crystals of compound **3** ^**OSe**^, and another batch of crystals could be obtained from mother liquor by crystallization at −30 °C. Combined yield of **3** ^**OSe**^ was 271 mg, (70%), mp 264 °C. Single-crystals suitable for *sc*-XRD diffraction analysis were obtained from saturated solution using dichloromethane/hexane mixture at 5 °C. Anal. Calcd for C_34_H_49_ClO_3_P_2_SeSn (MW 800.84): C, 51.0; H, 6.2%. Found: C, 50.8; H, 6.2%.^**1**^ **H NMR** (500 MHz, CDCl_3_) δ (ppm): 0.80 [9H, d­(br), ^3^ *J*(^31^P,^1^H) = 14.6 Hz, *t*Bu_2_P­(O/Se)–C*H*_3_], 0.99 [9H, d­(br), ^3^ *J*(^31^P,^1^H) = 16.9 Hz, *t*Bu_2_P­(O/Se)–C*H*_3_], 1.13 [9H, d­(br), ^3^ *J*(^31^P,^1^H) = 14.6 Hz, *t*Bu_2_P­(O/Se)–C*H*_3_], 1.49 [9H, d­(br), ^3^ *J*(^31^P,^1^H) = 16.4 Hz, *t*Bu_2_P­(O/Se)–C*H*_3_], 6.92 [1H, d, ^3^ *J*(^1^H,^1^H) = 8.0 Hz, Ar-*H*], 7.24 [3H, m­(br), Ar-*H*], 7.38 [1H, t, ^3^ *J*(^1^H,^1^H) = 8.1 Hz, Ar-*H*], 7.46 [5H, s­(br), Ar-*H*], 7.99 [1H, d, ^3^ *J*(^1^H,^1^H) = 8.5 Hz, Ar-*H*], 8.23 [2H, s­(br), ^2^ *J*(^119/117^Sn, ^1^H) = 74.4 Hz, Ar-*H*]. ^**13**^ **C­{** ^**1**^ **H} NMR**: Reasonable ^13^C NMR spectrum could not be obtained due to a significant line broadening even at low temperature. ^**31**^ **P­{** ^**1**^ **H} NMR** (202.5 MHz, CDCl_3_) δ (ppm): 79.1 [s, *t*Bu_2_ *P*(O)], 141.8 [s, ^1^ *J*(^77^Se,^31^P) = 780 Hz, *t*Bu_2_ *P*(Se)]. ^**77**^ **Se­{** ^**1**^ **H} NMR** (95.4 MHz, CDCl_3_) δ (ppm): −320.9 [d, ^1^ *J*(^77^Se,^31^P) = 780 Hz] measured at 263 K at r.t. the signal was too broad to be detected. ^**119**^ **Sn­{** ^**1**^ **H} NMR** (186.5 MHz, CDCl_3_) δ (ppm): −258.9 [s].

#### Synthesis of {[2-(*t*Bu_2_(O)­PO)-6-(*t*Bu_2_PO)­C_6_H_3_]­SnPh_2_}­{[B­[3,5-(CF_3_)_2_C_6_H_3_]_4_} (3^PO+^[BArF]^−^)

Solid Na­[BArF] (226 mg; 0.26 mmol) was added in one portion to solution of **3** ^**PO**^ (200 mg; 0.26 mmol) in dichloromethane (10 mL). The reaction mixture was stirred for 30 min at room temperature and then incipient NaCl was removed by filtration. Colorless solution was concentrated to 1/2 of the original volume and layered with hexane. Crystallization at room temperature gave colorless crystals of compound **3** ^**PO+**^ **[BArF]** ^**–**^. Yield of **3** ^**PO+**^ **[BArF]** ^**–**^ was 353 mg, (86%), mp 189–191 °C. Single-crystals suitable for *sc*-XRD diffraction analysis were obtained by slow diffusion of hexane into saturated dichloromethane solution at room temperature. Anal. Calcd for C_66_H_61_BF_24_O_3_P_2_Sn (MW 1549.64): C, 51.2; H, 4.0%. Found: C, 51.6; H, 4.3%. ^**1**^ **H NMR** (500 MHz, CDCl_3_) δ (ppm): 1.07 [18H, d, ^3^ *J*(^31^P,^1^H) = 14.8 Hz, *t*Bu_2_P–C*H*_3_], 1.16 [18H, d, ^3^ *J*(^31^P,^1^H) = 16.0 Hz, *t*Bu_2_(O)­P–C*H*_3_], 6.92 [1H, d, ^3^ *J*(^1^H,^1^H) = 8.1 Hz, Ar-*H*], 7.10 [1H, d, ^3^ *J*(^1^H,^1^H) = 8.3 Hz, Ar-*H*], 7.45 [1H, t, ^3^ *J*(^1^H,^1^H) = 8.2 Hz, Ar-*H*], 7.50 [6H, m, Ar-*H*], 7.53 [4H, m, Ar-*H*], 7.67 [4H, m, ^3^ *J*(^119/117^Sn, ^1^H) = 76.6 Hz, Ar-*H*], 7.73 [8H, m, Ar-*H*]. ^**11**^ **B­{** ^**1**^ **H} NMR** (160.42 MHz, CDCl_3_) δ (ppm): −7.2 [s]. ^**13**^ **C­{** ^**1**^ **H} NMR** (125.78 MHz, CDCl_3_) δ (ppm): 26.1 [s, *t*Bu_2_(O)­P-*C*H_3_], 27.1 [d, ^2^ *J*(^31^P,^13^C) = 6.2 Hz *t*Bu_2_P-*C*H_3_], 37.2 [d, ^1^ *J*(^31^P,^13^C) = 72.7 Hz, *t*Bu_2_(O)­P-*C*], 39.2 [s, ^n^ *J*(^119/117^Sn,^13^C) = 14.4 Hz, *t*Bu_2_P-*C*], 115.2 [d, ^n^ *J*(^31^P,^13^C) = 55.6 Hz, Ar-*C*], 116.1 [m, Ar-*C*], 116.8 [d, ^n^ *J*(^31^P,^13^C) = 3.4 Hz, ^n^ *J*(^119/117^Sn,^13^C) = 32.6 Hz, Ar-*C*], 117.7 [m, Ar-*C*], 124.7 [q, ^1^ *J*(^19^F,^13^C) = 273 Hz, *C*F_3_], 129.2 [qq, ^2^ *J*(^19^F,^13^C) = 31.5 Hz, ^4^ *J*(^19^F,^13^C) = 2.8 Hz, Ar-*C*], 130.0 [s, ^3^ *J*(^119/117^Sn,^13^C) = 78.1 Hz, Ar-*C*], 131.5 [s, ^4^ *J*(^119/117^Sn,^13^C) = 15.8 Hz, Ar-*C*], 134.9 [s, Ar-*C*], 135.0 [s, Ar-*C*], 136.1 [s, ^2^ *J*(^119/117^Sn,^13^C) = 51.7 Hz Ar-*C*], 139.2 [d, ^n^ *J*(^31^P,^13^C) = 1.7 Hz, Ar-*C*], 139.5 [d, ^n^ *J*(^31^P,^13^C) = 1.8 Hz, Ar-*C*] 159.8 [m, Ar-*C*], 161.9 [q, ^1^ *J*(^13^C,^11^B) = 50 Hz, Ar-*C*]. ^**19**^ **F­{** ^**1**^ **H} NMR** (376.3 MHz, CDCl_3_) δ (ppm): −62.4 [s]. ^**31**^ **P­{** ^**1**^ **H} NMR** (202.5 MHz, CDCl_3_) δ (ppm): 84.4 [s, ^n^ *J*(^119/117^Sn,^31^P) = 48.2 Hz, *t*Bu_2_(O)*P*], 101.8 [s, ^n^ *J*(^119/117^Sn,^31^P) = 960/920 Hz, *t*Bu_2_ *P*]. ^**119**^ **Sn­{** ^**1**^ **H} NMR** (186.5 MHz, CDCl_3_) δ (ppm): −255.2 [dd, ^n^ *J*(^119/117^Sn,^31^P) = 960/920 Hz].

#### Synthesis of {[2-(*t*Bu_2_(O)­PO)-6-(*t*Bu_2_(S)­PO)­C_6_H_3_]­SnPh_2_}­{[B­[3,5-(CF_3_)_2_C_6_H_3_]_4_} (3^OS+^[BArF]^−^)

Solid Na­[BArF] (60 mg; 0.07 mmol) was added in one portion to solution of **3** ^**OS**^ (55 mg; 0.07 mmol) in dichloromethane (10 mL). The reaction mixture was stirred for 30 min at room temperature and then incipient NaCl was removed by filtration. Colorless solution was concentrated to 1/2 of the original volume and layered with hexane. Crystallization at room temperature gave colorless crystals of compound **3** ^**OS+**^ **[BArF]** ^**–**^. Yield of **3** ^**OS+**^ **[BArF]** ^**–**^ was 96 mg, (87%), mp 216–219 °C. Single-crystals suitable for *sc*-XRD diffraction analysis were obtained by slow diffusion of hexane into saturated dichloromethane solution at room temperature. Anal. Calcd for C_66_H_61_BF_24_O_3_P_2_SSn (MW 1581.70): C, 50.1; H, 3.9%. Found: C, 50.3; H, 4.1%. ^**1**^ **H NMR** (500 MHz, CDCl_3_) δ (ppm): 1.03 and 1.17 [18 + 18H, d, ^3^ *J*(^31^P,^1^H) = 16/17.4 Hz, *t*Bu_2_(O/S)­P–C*H*_3_], 7.16 [2H, m, Ar-*H*], 7.49 [6H, m, Ar-*H*], 7.53 [5H, m, Ar-*H*], 7.73 [12H, m, Ar-*H*]. ^**11**^ **B­{** ^**1**^ **H} NMR** (160.42 MHz, CDCl_3_) δ (ppm): −6.6 [s]. ^**13**^ **C­{** ^**1**^ **H} NMR** (125.78 MHz, CDCl_3_) δ (ppm): 26.1 and 27.0 [s, *t*Bu_2_(O/S)­P-*C*H_3_], 37.5 [d, ^1^ *J*(^31^P,^13^C) = 72.3 Hz, *t*Bu_2_(O)­P-*C*], 42.4 [d, ^1^ *J*(^31^P,^13^C) = 46.6 Hz, *t*Bu_2_(S)­P-*C*], 117.7 [m, Ar-*C*], 120.3 [d, ^n^ *J*(^31^P,^13^C) = 5.5 Hz, ^n^ *J*(^119/117^Sn,^13^C) = 28.7 Hz, Ar-*C*], 120.6 [d, ^n^ *J*(^31^P,^13^C) = 4.2 Hz, ^n^ *J*(^119/117^Sn,^13^C) = 29.4 Hz, Ar-*C*], 121.4 [m, Ar-*C*], 124.8 [q, ^1^ *J*(^19^F,^13^C) = 273 Hz, *C*F_3_], 129.1 [qq, ^2^ *J*(^19^F,^13^C) = 31.5 Hz, ^4^ *J*(^19^F,^13^C) = 2.8 Hz, Ar-*C*], 129.7 [s, ^3^ *J*(^119/117^Sn,^13^C) = 81.3 Hz, Ar-*C*], 131.9 [s, ^4^ *J*(^119/117^Sn,^13^C) = 16.5 Hz, Ar-*C*], 134.1 [s, Ar-*C*], 135.0 [s, Ar-*C*], 135.9 [s, ^2^ *J*(^119/117^Sn,^13^C) = 52.9 Hz Ar-*C*], 141.4 [d, ^1^ *J*(^119/117^Sn,^13^C) = 886/845 Hz, Ar-*C*], 158.2 [d, ^n^ *J*(^31^P,^13^C) = 10.5 Hz, Ar-*C*], 158.5 [d, ^n^ *J*(^31^P,^13^C) = 13.9 Hz, Ar-*C*], 161.9 [q, ^1^ *J*(^13^C,^11^B) = 50 Hz, Ar-*C*]. ^**19**^ **F­{** ^**1**^ **H} NMR** (376.3 MHz, CDCl_3_) δ (ppm): −62.4 [s]. ^**31**^ **P­{** ^**1**^ **H} NMR** (202.5 MHz, CDCl_3_) δ (ppm): 84.6 [s, *t*Bu_2_(O)*P*], 132.4 [s, *t*Bu_2_(S)*P*]. ^**119**^ **Sn­{** ^**1**^ **H} NMR** (186.5 MHz, CDCl_3_) δ (ppm): −293.9 [s].

#### Synthesis of {[2-(*t*Bu_2_(O)­PO)-6-(*t*Bu_2_(Se)­PO)­C_6_H_3_]­SnPh_2_}­{[B­[3,5-(CF_3_)_2_C_6_H_3_]_4_} (3^OSe+^[BArF]^−^)

Solid Na­[BArF] (118 mg; 0.13 mmol) was added in one portion to solution of **3** ^**OSe**^ (115 mg; 0.13 mmol) in dichloromethane (10 mL). The reaction mixture was stirred for 30 min at room temperature and then incipient NaCl was removed by filtration. Colorless solution was concentrated to 1/2 of the original volume and layered with hexane. Crystallization at room temperature gave colorless crystals of compound **3** ^**OSe+**^ **[BArF]** ^**–**^. Yield of **3** ^**OSe+**^ **[BArF]** ^**–**^ was 192 mg, (85%), mp 220–222 °C. Single-crystals suitable for *sc*-XRD diffraction analysis were obtained from saturated solution using dichloromethane/hexane mixture at −30 °C. Anal. Calcd for C_66_H_61_BF_24_O_3_P_2_SeSn (MW 1628.61): C, 48.7; H, 3.8%. Found: C, 49.0; H, 3.9%. ^**1**^ **H NMR** (500 MHz, CDCl_3_) δ (ppm): 1.02 and 1.20 [18 + 18H, d, ^3^ *J*(^31^P,^1^H) = 16/17.9 Hz, *t*Bu_2_(O/Se)­P–C*H*_3_], 7.18 [2H, m, Ar-*H*], 7.48 [6H, m, Ar-*H*], 7.54 [5H, m, Ar-*H*], 7.73 [8H, m, Ar-*H*], 7.75 [4H, m, ^3^ *J*(^119/117^Sn, ^1^H) = 77.3 Hz, Ar-*H*]. ^**11**^ **B­{** ^**1**^ **H} NMR** (160.42 MHz, CDCl_3_) δ (ppm): −7.2 [s]. ^**13**^ **C­{** ^**1**^ **H} NMR** (125.78 MHz, CDCl_3_) δ (ppm): 26.2 and 27.2 [s, *t*Bu_2_(O/Se)­P-*C*H_3_], 37.5 [d, ^1^ *J*(^31^P,^13^C) = 71.2 Hz, *t*Bu_2_(O)­P-*C*], 43.1 [d, ^1^ *J*(^31^P,^13^C) = 35.1 Hz, *t*Bu_2_(Se)­P-*C*], 117.7 [m, Ar-*C*], 120.6 [d, ^n^ *J*(^31^P,^13^C) = 5.6 Hz, ^n^ *J*(^119/117^Sn,^13^C) = 27.9 Hz, Ar-*C*], 121.0 [d, ^n^ *J*(^31^P,^13^C) = 3.5 Hz, ^n^ *J*(^119/117^Sn,^13^C) = 29.9 Hz, Ar-*C*], 121.3 [m, Ar-*C*], 124.8 [q, ^1^ *J*(^19^F,^13^C) = 273 Hz, *C*F_3_], 129.1 [qq, ^2^ *J*(^19^F,^13^C) = 31.5 Hz, ^4^ *J*(^19^F,^13^C) = 2.8 Hz, Ar-*C*], 129.6 [s, ^3^ *J*(^119/117^Sn,^13^C) = 81.8 Hz, Ar-*C*], 130.8 [s, ^4^ *J*(^119/117^Sn,^13^C) = 16.6 Hz, Ar-*C*], 133.8 [s, Ar-*C*], 135.0 [s, Ar-*C*], 135.9 [s, ^2^ *J*(^119/117^Sn,^13^C) = 52.6 Hz Ar-*C*], 141.8 [d, ^1^ *J*(^119/117^Sn,^13^C) = 881/841 Hz, Ar-*C*], 157.9 [d, ^n^ *J*(^31^P,^13^C) = 10.7 Hz, Ar-*C*], 158.6 [d, ^n^ *J*(^31^P,^13^C) = 13.7 Hz, Ar-*C*], 161.9 [q, ^1^ *J*(^13^C,^11^B) = 50 Hz, Ar-*C*]. ^**19**^ **F­{** ^**1**^ **H} NMR** (376.3 MHz, CDCl_3_) δ (ppm): −62.4 [s]. ^**31**^ **P­{** ^**1**^ **H} NMR** (202.5 MHz, CDCl_3_) δ (ppm): 84.4 [s, ^n^ *J*(^119/117^Sn,^31^P) = 36.4 Hz, *t*Bu_2_(O)*P*], 139.9 [s, ^1^ *J*(^77^Se,^31^P) = 647 Hz, *t*Bu_2_(Se)*P*]. ^**77**^ **Se­{** ^**1**^ **H} NMR** (95.4 MHz, CDCl_3_) δ (ppm): −220.1 [d, ^1^ *J*(^77^Se,^31^P) = 647 Hz]. ^**119**^ **Sn­{** ^**1**^ **H} NMR** (186.5 MHz, CDCl_3_) δ (ppm): −298.1 [d, ^n^ *J*(^119/117^Sn,^31^P) = 36.4 Hz, ^1^ *J*(^119/117^Sn, ^77^Se) = 440 Hz].

#### Synthesis of 2,6-(*t*Bu_2_(O)­PO)_2_C_6_H_3_Br (Ar^O^Br)

30% solution of hydrogen peroxide in water (0.24 mL; 2.35 mmol) was slowly added to solution of **ArBr** (526 mg; 1.10 mmol) in dichloromethane (20 mL). Mixture was stirred for 5 min and transferred to Schlenk flask with dried molecular sieves. After 10 min of vigorous stirring, the molecular sieves were removed by filtration. Solvent was removed in vacuo and white powder dried to remove any excess traces of water. Recrystallization from dichloromethane/hexane solution gave colorless crystals of compound **Ar** ^**O**^ **Br**. Yield of **Ar** ^**O**^ **Br** was 380 mg, (68%), mp 208–210 °C. Single-crystals suitable for *sc*-XRD diffraction analysis were obtained from saturated solution using dichloromethane/hexane mixture at −30 °C. Anal. Calcd for C_22_H_39_BrO_4_P_2_ (MW 509.40): C, 51.9; H, 7.8%. Found: C, 52.1; H, 7.9%. ^**1**^ **H NMR** (400 MHz, CDCl_3_) δ (ppm): 1.33 [36H, d, ^3^ *J*(^31^P,^1^H) = 15.0 Hz, *t*Bu_2_(S)­P–C*H*_3_], 7.08 [1H, t, ^3^ *J*(^1^H,^1^H) = 9.0 Hz, Ar-*H*], 7.64 [2H, d, ^3^ *J*(^1^H,^1^H) = 9.0 Hz, Ar-*H*]. ^**13**^ **C­{** ^**1**^ **H} NMR** (125.78 MHz, CDCl_3_) δ (ppm): 26.6 [s, *t*Bu_2_P-*C*H_3_], 37.5 [d, ^1^ *J*(^31^P,^13^C) = 80.2 Hz, *t*Bu_2_P-*C*], 104.7 [t, ^3^ *J*(^31^P,^13^C) = 7.3 Hz], 115.1 [d, ^3^ *J*(^31^P,^13^C) = 1.9 Hz, Ar-*C*], 128.6 [s, Ar-*C*], 152.4 [d, ^2^ *J*(^31^P,^13^C) = 9.4 Hz, Ar-*C*]. ^**31**^ **P­{** ^**1**^ **H} NMR** (202.5 MHz, CDCl_3_) δ (ppm): 69.9 [s].

#### Synthesis of 2,6-(*t*Bu_2_(S)­PO)_2_C_6_H_3_Br (Ar^S^Br)

Elemental sulfur (38 mg; 1.19 mmol) was added in one portion to solution of **ArBr** (280 mg; 0.59 mmol) in dichloromethane (10 mL). The reaction mixture was stirred for 24 h at room temperature and then was concentrated to 1/2 of the original volume. Yellowish solution was layered with hexane. Crystallization at room temperature gave colorless crystals of compound **Ar** ^**S**^ **Br**. Yield of **Ar** ^**S**^ **Br** was 285 mg, (90%), mp 208–210 °C. Single-crystals suitable for *sc*-XRD diffraction analysis were obtained by slow diffusion of hexane into saturated dichloromethane solution at room temperature. Anal. Calcd for C_22_H_39_BrO_2_P_2_S_2_ (MW 541.52): C, 48.8; H, 7.3%. Found: C, 48.9; H, 7.4%. ^**1**^ **H NMR** (500 MHz, C_6_D_6_) δ (ppm): 1.32 [36H, d, ^3^ *J*(^31^P,^1^H) = 16.6 Hz, *t*Bu_2_(S)­P–C*H*_3_], 6.74 [1H, t, ^3^ *J*(^1^H,^1^H) = 8.6 Hz, Ar-*H*], 8.31 [2H, d, ^3^ *J*(^1^H,^1^H) = 8.6 Hz, Ar-*H*]. ^**13**^ **C­{** ^**1**^ **H} NMR** (125.78 MHz, C_6_D_6_) δ (ppm): 27.8 [s, *t*Bu_2_P-*C*H_3_], 42.4 [d, ^1^ *J*(^31^P,^13^C) = 58.1 Hz, *t*Bu_2_P-*C*], 106.7 [t, ^3^ *J*(^31^P,^13^C) = 6.6 Hz], 116.6 [d, ^3^ *J*(^31^P,^13^C) = 3.9 Hz, Ar-*C*], 127.9 [s, Ar-*C*], 153.1 [d, ^2^ *J*(^31^P,^13^C) = 11.0 Hz, Ar-*C*]. ^**31**^ **P­{** ^**1**^ **H} NMR** (202.5 MHz, C_6_D_6_) δ (ppm): 128.9 [s].

#### Synthesis of 2,6-(*t*Bu_2_(Se)­PO)_2_C_6_H_3_Br (Ar^Se^Br)

Elemental selenium (1.9 g; 24.1 mmol) was added in one portion to solution of **ArBr** (2.3 g; 4.82 mmol) in dichloromethane (40 mL). The reaction mixture was stirred for 1 h at room temperature and then unreacted selenium was removed by filtration. Resulting colorless solution was concentrated to 1/4 of the original volume and was layered with hexane. Crystallization at −30 °C gave colorless crystals of compound **Ar** ^**Se**^ **Br**. Yield of **Ar** ^**Se**^ **Br** was 2.04 g, (67%), mp 261–263 °C. Single-crystals suitable for *sc*-XRD diffraction analysis were obtained from saturated solution using dichloromethane/hexane mixture at −30 °C. Anal. Calcd for C_22_H_39_BrO_2_P_2_Se_2_ (MW 635.35): C, 41.6; H, 6.2%. Found: C, 41.7; H, 6.4%. ^**1**^ **H NMR** (500 MHz, CDCl_3_) δ (ppm): 1.48 [36H, d, ^3^ *J*(^31^P,^1^H) = 16.5 Hz, *t*Bu_2_(Se)­P–C*H*_3_], 7.14 [1H, t, ^3^ *J*(^1^H,^1^H) = 8.2 Hz, Ar-*H*], 8.14 [2H, d, ^3^ *J*(^1^H,^1^H) = 8.2 Hz, Ar-*H*]. ^**13**^ **C­{** ^**1**^ **H} NMR** (125.78 MHz, CDCl_3_) δ (ppm): 27.9 [d, ^2^ *J*(^31^P,^13^C) = 1.9 Hz, *t*Bu_2_(Se)­P-*C*H_3_], 43.2 [d, ^2^ *J*(^31^P,^13^C) = 46.5 Hz, *t*Bu_2_(Se)­P-*C*], 106.5 [t, ^3^ *J*(^31^P,^13^C) = 5.9 Hz, Ar-*C*], 116.0 [d, ^3^ *J*(^31^P,^13^C) = 3.9 Hz, Ar-*C*], 126.7 [s, Ar-*C*], 152.3 [d, ^2^ *J*(^31^P,^13^C) = 10.7 Hz, Ar-*C*]. ^**31**^ **P­{** ^**1**^ **H} NMR** (202.5 MHz, CDCl_3_) δ (ppm): 138.9 [s, ^1^ *J*(^77^Se,^31^P) = 800 Hz, *t*Bu_2_(Se)*P*]. ^**77**^ **Se­{** ^**1**^ **H} NMR** (95.4 MHz, CDCl_3_) δ (ppm): −311.2 [d, ^1^ *J*(^77^Se,^31^P) = 800 Hz].

### Details for the Theoretical Studies

All the calculations were carried out using the Gaussian 16 suite of programs.^48^ All structures were optimized using the ωB97X-D functional combined with the def2-SVP basis set. Single point energy calculations were performed at the ωB97X-D/def2-TZVP level of theory and the Gibbs free energies values were corrected with the entropy term obtained at the ωB97X-D/def2-SVP level. Harmonic vibrational frequency calculations were carried out to characterize the stationary points located on the potential energy hypersurface. If the stationary point is a minimum, no imaginary frequencies were obtained. To obtain WBI values and NPA charges, NBO analysis was carried out with the NBO 7.0 program.^49^ For the Atoms-in-Molecules (AIM) the Multiwfn program was employed.^50^ The Natural Bond Orbitals were visualized using by the IQmol 2.15.3. program.^51^

## Supplementary Material



## Notes

Syntheses, NMR spectra, molecular structures determined by *sc* X-ray crystallography, details for *sc* X-ray crystallography, details for DFT (PDF)
