# Supplementary material for: Straightforward Donor Atom Switching: From P,C,P- to Various Y,C,Y-Pincer (Y = O, S, Se) Organotin(IV) Compounds and Cations
Source: Inorg Chem. 2026 Apr 21;65(17):9372–89. doi: 10.1021/acs.inorgchem.5c05899 (PMC13147332; doi:10.1021/acs.inorgchem.5c05899)
Supplement: Supplementary file 1 [file ic5c05899_si_001.pdf]

Supporting Information for

Straightforward Donor Atom Switching: From P,C,P- to Various Y,C,Y-Pincer (Y = O, S, Se) Organotin(IV) Compounds and Cations.

Richard Chlebík,[a] Erik Kertész,[b] Zdeňka Růžičková,[a] Aleš Růžička,[a] Roman Jambor,[a] Zoltán Benkő[b,c] and Libor Dostál[a,]**

[a]Department of General and Inorganic Chemistry, University of Pardubice, Studentská 573, CZ 532 10 Pardubice, Czech Republic. *E-mail: libor.dostal@upce.cz

[b]Department of Inorganic and Analytical Chemistry, Faculty of Chemical Technology and Biotechnology, Budapest University of Technology and Economics, Műegyetem rkp. 3, H-1111 Budapest, Hungary, *E-mail: benko.zoltan@vbk.bme.hu

[c]HUN-REN-BME Computation Driven Chemistry Research Group, H-1111 Budapest, Műegyetem rkp. 3, Hungary

Contents

General procedures S2

Synthesis and NMR spectra of studied compounds S3-S107

VT-NMR experiments S108-S114

Crystallographic data of studied compounds S115-S123

Details for the theoretical studies. S124-S209

References S210

General procedures

All manipulations were carried out under an argon atmosphere using Schlenk tube technique. Solvents were dried using Pure Solv–Innovative Technology equipment. Deuterated solvents were dried and degassed by standard procedures and stored over potassium mirror or molecular sieves. All reagents except those referenced in the main text were purchased from commercial sources and used as delivered. ^{1}H and ^{13}C{^{1}H} NMR spectra were recorded on Bruker Ascend 500 MHz and Bruker Ultrashield 400 MHz spectrometers, using a 5 mm tunable broad-band probe or a cryo-probe Prodigy at 295 K (if another temperature is not defined). Appropriate chemical shifts in ^{1}H and ^{13}C{^{1}H} NMR spectra are given relative to the residual signals of the solvent [C_6D_6: δ(^{1}H) = 7.16 ppm and δ(^{13}C) = 128.39 ppm, CD_2Cl_2: δ(^{1}H) = 5.32 ppm and δ(^{13}C) = 54.0 ppm, $CDCl_3$: δ(^{1}H) = 7.27 ppm and δ(^{13}C) = 77.23 ppm, CD_3CN: δ(^{1}H) = 1.94 ppm and δ(^{13}C) = 1.39 ppm]. ^{11}B{^{1}H}, ^{19}F{^{1}H}, ^{31}P{^{1}H}, ^{77}Se{^{1}H} and ^{119}Sn{^{1}H} NMR spectra were related to external standards BF_3.Et_2O (δ(^{11}B) = 0.00 ppm), $CFCl_3$ (δ(^{19}F) = 0.00 ppm), 85% H_3PO_4 (δ(^{31}P) = 0.00 ppm), Ph_2Se_2 (δ(^{77}Se) = 460.00 ppm) and Me_4Sn (δ(^{119}Sn) = 0.00 ppm), respectively. Elemental analyses were performed on Flash 2000 CHNS analyser.

Synthesis and NMR spectra of studied compounds.

Synthesis of [2,6-(tBu$_2$(O)PO)$_2$C$_6$H$_3$]SnCl$_3$ (1^O)

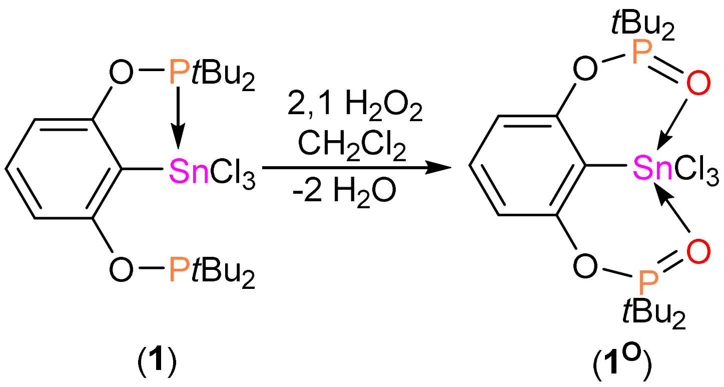

Solution of **1** (170 mg; 0.27 mmol) in dichloromethane (20 ml) was transferred to Schlenk flask with dried molecular sieves. 30% solution of hydrogen peroxide in water (59 µl; 0.58 mmol) was slowly added, while resulting mixture was stirred vigorously. The reaction mixture was stirred for 5 min at room temperature and then molecular sieves were removed by filtration. Solvent was removed in vacuo and white powder was rigorously dried to remove any excess traces of water. Recrystallisation from dichloromethane/hexane solution gave colorless crystals of compound **1^O**. Yield of **1^O** was 106 mg, (59 %), m. p. 293°C. Single-crystals suitable for *sc*-XRD diffraction analysis were obtained from saturated solution using dichloromethane/hexane mixture. Anal. Calcd for $C_{22}H_{39}Cl_3O_4P_2Sn$ (MW 654.56): C, 40.4; H, 6.0 %. Found: C, 40.6; H, 6.3 %. **^{1}H NMR** (500 MHz, CDCl$_3$) δ (ppm): 1.47 [36H, d, $^3J(^{31}P,^1H)$ = 16.0 Hz, tBu$_2$(O)P-CH_3], 6.86 [2H, d, $^3J(^1H,^1H)$ = 8.2 Hz, $^4J(^{119/117}Sn,^1H)$ = 45.6 Hz, Ar-*H*], 7.23 [1H, t, $^3J(^1H,^1H)$ = 8.2 Hz, Ar-*H*]. **^{13}C{^{1}H} NMR** (125.78 MHz, CDCl$_3$) δ (ppm): 27.0 [s, tBu$_2$(O)P-CH$_3$], 38.0 [d, $^1J(^{31}P,^{13}C)$ = 73.1 Hz, tBu$_2$(O)P-*C*], 117.6 [d, $^3J(^{31}P,^{13}C)$ = 7.2 Hz, $^3J(^{119/117}Sn,^{13}C)$ = 54.3 Hz, Ar-*C*], 130.4 [s, Ar-*C*], 136.6 [s, Ar-*C*] 157.5 [d, $^2J(^{31}P,^{13}C)$ = 12.7 Hz, Ar-*C*]. **^{31}P{^{1}H} NMR** (202.5 MHz, CDCl$_3$) δ (ppm): 85.0 [s, $^nJ(^{119/117}Sn,^{31}P)$ = 73 Hz]. **^{119}Sn{^{1}H} NMR** (186.5 MHz, CDCl$_3$) δ (ppm): -632.6 [t, $^nJ(^{119/117}Sn,^{31}P)$ = 73 Hz].

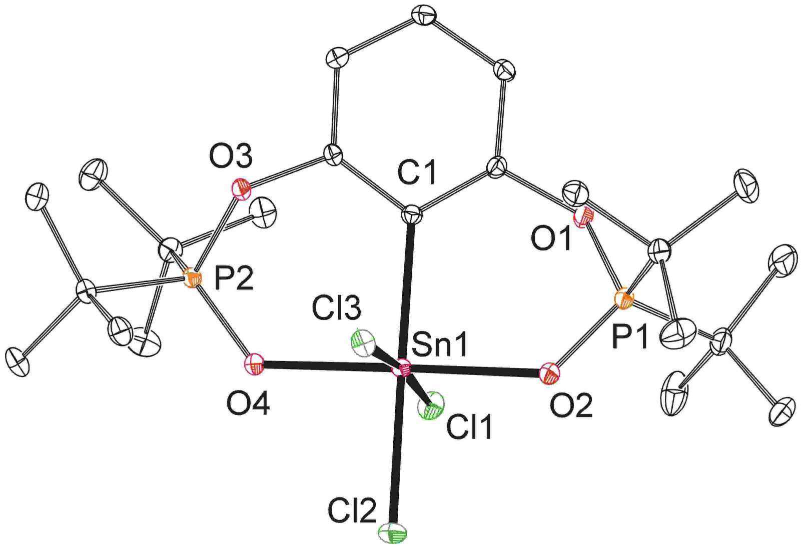

Figure S1. Molecular structure of **1**^{O}. ORTEP with 30% probability ellipsoid level. Hydrogen atoms and dichloromethane solvate molecule are omitted. Selected structural bond lengths [Å]: Sn(1)-C(1) 2.1882(15), Sn(1)-Cl(1) 2.4178(5), Sn(1)-Cl(2) 2.3773(5), Sn(1)-Cl(3) 2.4414(5), Sn(1)-O(2) 2.1431(11), Sn(1)-O(4) 2.1386(11); bonding angles [°]: C(1)-Sn(1)-Cl(2) 179.62(4), Cl(1)-Sn(1)-Cl(3) 174.65(2), O(2)-Sn(1)-O(4) 178.45(5).

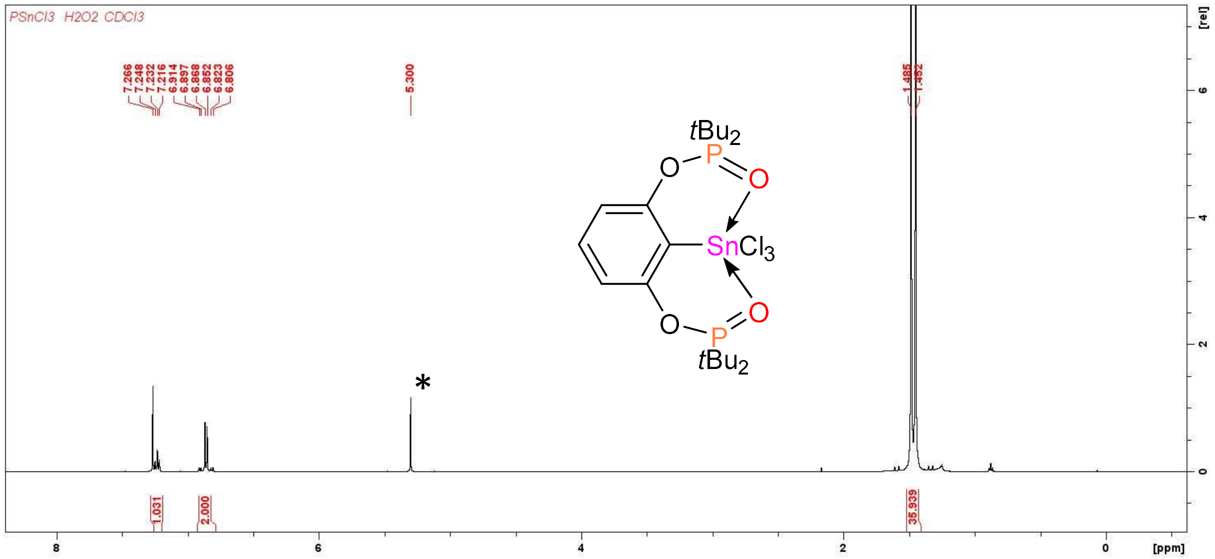

Figure S2. ^{1}H NMR spectrum of **1**^{O} (500 MHz, $CDCl_3$). *Signal of co-crystalized dichloromethane.

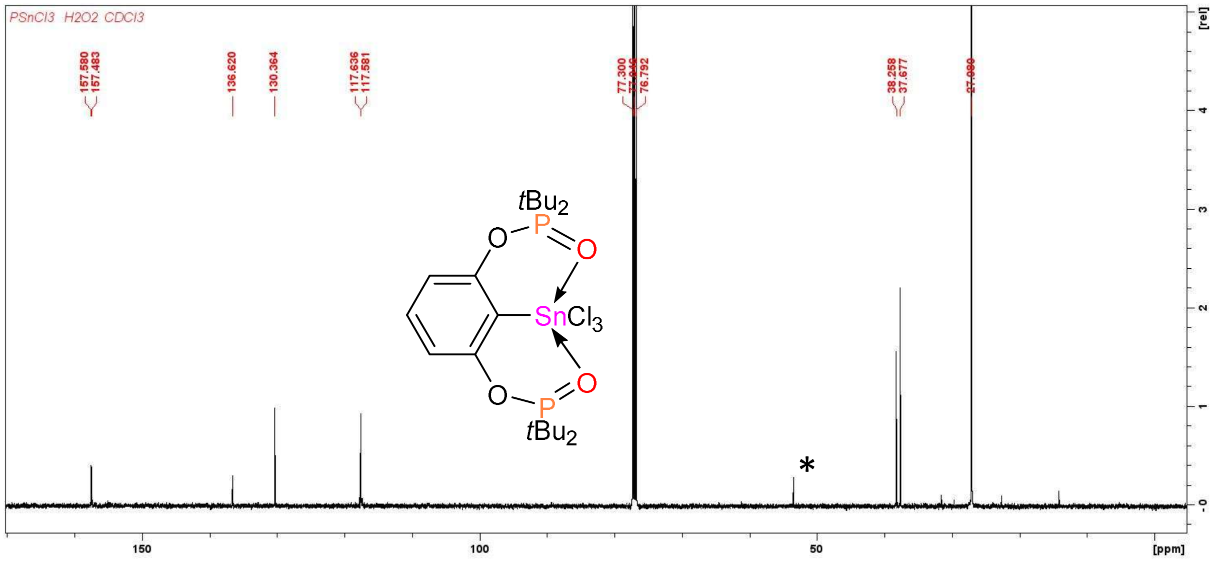

Figure S3. $^{13}C\{^{1}H\}$ NMR spectrum of $\mathbf{1^{O}}$ (125.76 MHz, $CDCl_3$). *Signal of co-crystalized dichloromethane.

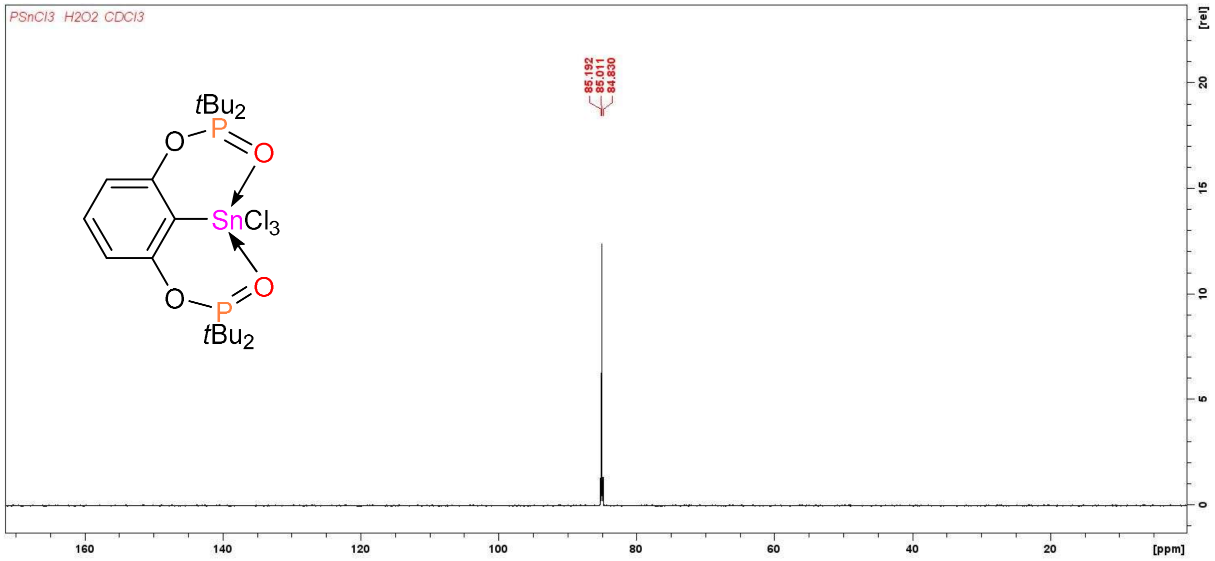

Figure S4. $^{31}P\{^{1}H\}$ NMR spectrum of $\mathbf{1^{O}}$ (202.5 MHz MHz, $CDCl_3$).

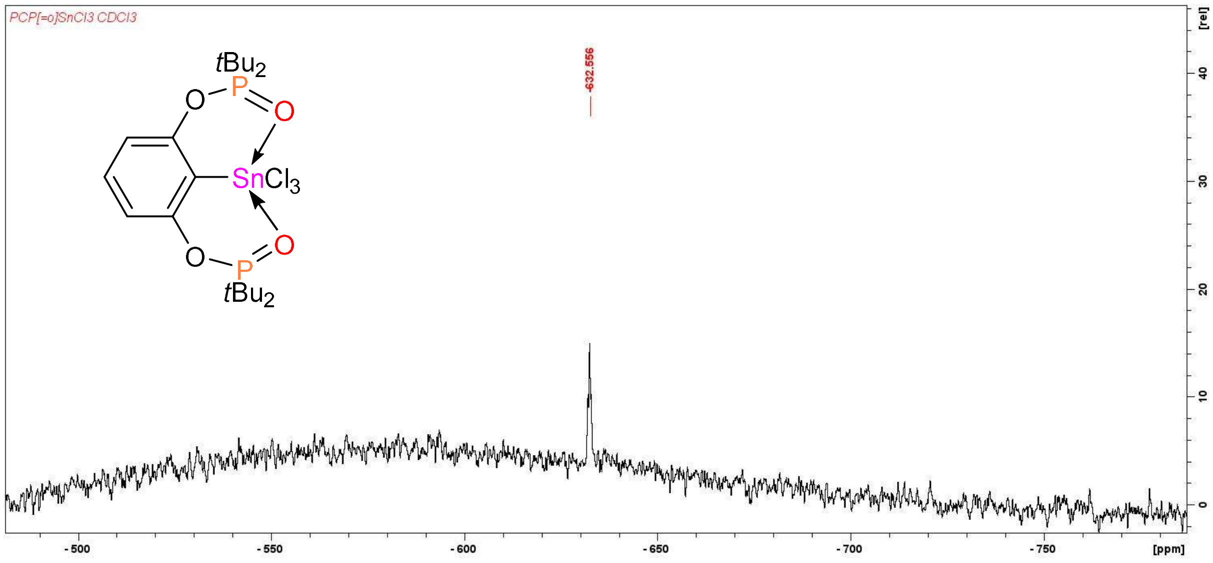

Figure S5. $^{119}Sn\{^{1}H\}$ NMR spectrum of $\mathbf{1^{O}}$ (186.5 MHz, $CDCl_3$).

Synthesis of [2,6-(tBu$_2$(S)PO)$_2$C$_6$H$_3$]SnCl$_3$ ($\mathbf{1^{S}}$)

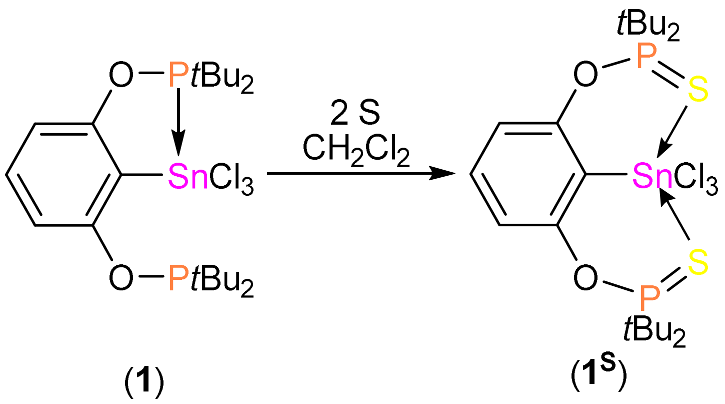

(**1**) (**1^{S}**)

Elemental sulfur (21 mg; 0.66 mmol) was added in one portion to solution of **1** (366 mg; 0.33 mmol) in dichloromethane (20 ml). The reaction mixture was stirred for 3 days at room temperature and then was concentrated to 1/2 volume. Yellowish solution was layered with hexane. Crystallization at room temperature gave yellow crystals of compound **1^{S}**. Yield of **1^{S}** was 128 mg, (57 %), m. p. 234-236°C. Single-crystals suitable for *sc*-XRD diffraction analysis were obtained from saturated solution using dichloromethane/hexane mixture. Anal. Calcd for $C_{22}H_{39}Cl_3O_2P_2S_2Sn$ (MW 686.68): C, 38.5; H, 5.7 %. Found: C, 38.7; H, 6.2 %. **^{1}H NMR** (500 MHz, CD_2Cl_2) δ (ppm): 1.46 [36H, d, $^{3}J(^{31}P,^{1}H)$ = 17.6 Hz, tBu$_2$(S)P-CH_3], 7.00 [2H, d,

$^{3}J(^{1}H,^{1}H)$ = 8.1 Hz, $^{4}J(^{119/117}Sn, ^{1}H)$ = 45.5 Hz, Ar-*H*], 7.33 [1H, t, $^{3}J(^{1}H,^{1}H)$ = 8.1 Hz, Ar-*H*]. **$^{13}C\{^{1}H\}$ NMR** (125.78 MHz, CD_2Cl_2) δ (ppm): 27.5 [s, *t*Bu2(S)P-*C*H3], 42.8 [d, $^{1}J(^{31}P,^{13}C)$ = 46.0 Hz, *t*Bu2(S)P-*C*], 120.4 [d, $^{3}J(^{31}P,^{13}C)$ = 5.1 Hz, $^{3}J(^{119/117}Sn,^{13}C)$ = 42.5 Hz, Ar-*C*], 130.8 [s, Ar-*C*], 136.8 [s, Ar-*C*], 157.2 [d, $^{1}J(^{31}P,^{13}C)$ = 13.8 Hz, Ar-*C*]. **$^{31}P\{^{1}H\}$ NMR** (202.5 MHz, CD_2Cl_2) δ (ppm): 125.6 [s(br)]. **$^{119}Sn\{^{1}H\}$ NMR** (186.5 MHz, CD_2Cl_2) δ (ppm): -640.3 [s].

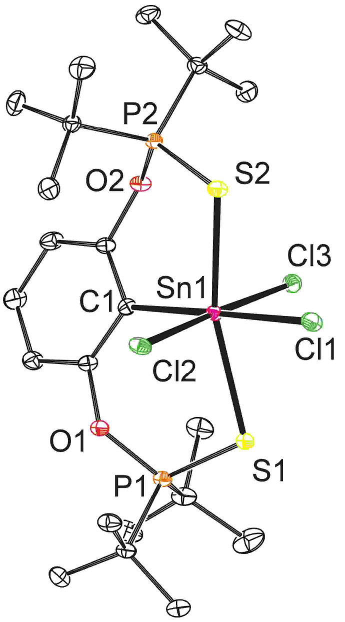

Figure S6. Molecular structure of **1**^{S}. ORTEP with 30% probability ellipsoid level. Only one of two independent molecules in the unit cell is presented. Hydrogen atoms are omitted. Selected structural bond lengths [Å]: Sn(1)-C(1) 2.187(2), Sn(1)-Cl(1) 2.4366(8), Sn(1)-Cl(2) 2.4598(7), Sn(1)-Cl(3) 2.4499(7), Sn(1)-S(1) 2.5722(7), Sn(1)-S(2) 2.6162(7); bonding angles [°]: Cl(1)-Sn(1)-C(1) 179.21(6), Cl(2)-Sn(1)-Cl(3) 177.70(3), S(1)-Sn(1)-S(2) 161.78(3). Selected structural bond lengths for the second independent molecule [Å]: Sn(2)-C(101) 2.180(3), Sn(2)-Cl(4) 2.4127(10), Sn(2)-Cl(5) 2.4511(7), Sn(2)-Cl(6) 2.4537(7), Sn(2)-S(3) 2.6214(8), Sn(2)-S(4) 2.5747(7); bonding angles [°]: Cl(4)-Sn(2)-C(101) 177.90(6), Cl(5)-Sn(2)-Cl(6) 176.39(3), S(3)-Sn(2)-S(3) 163.81(3).

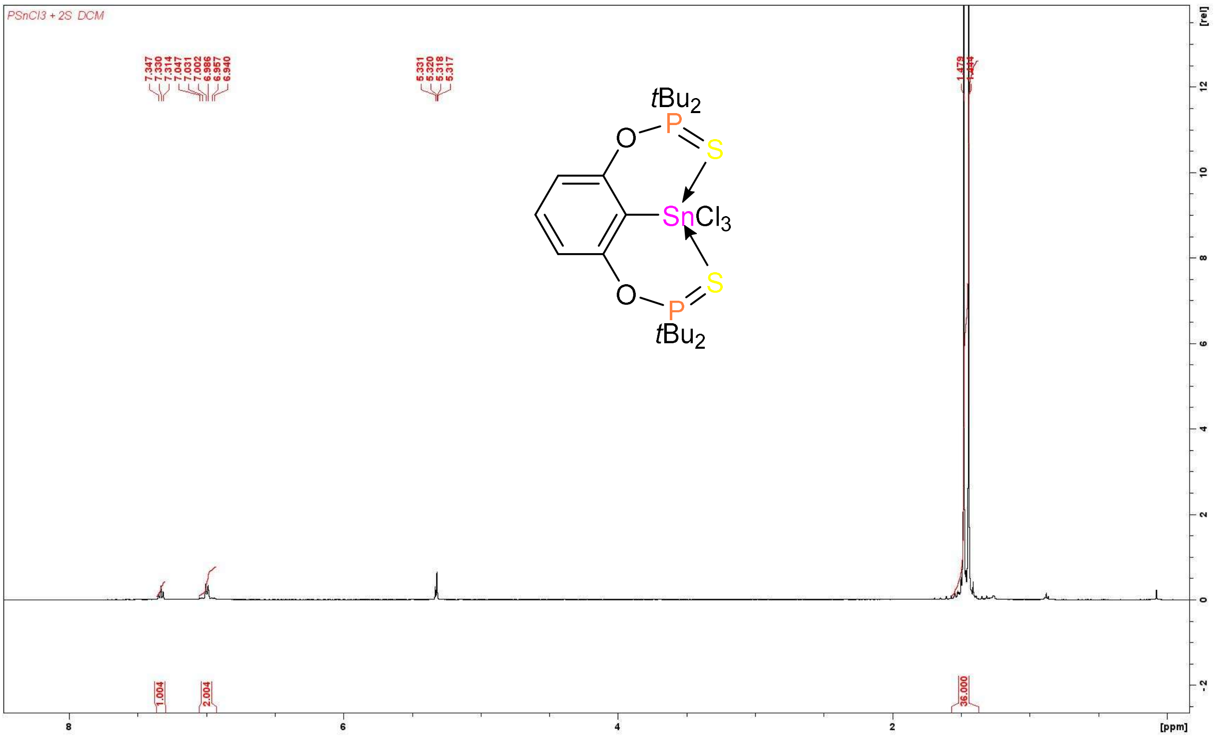

Figure S7. ^{1}H NMR spectrum of $\mathbf{1^S}$ (500 MHz, CD_2Cl_2).

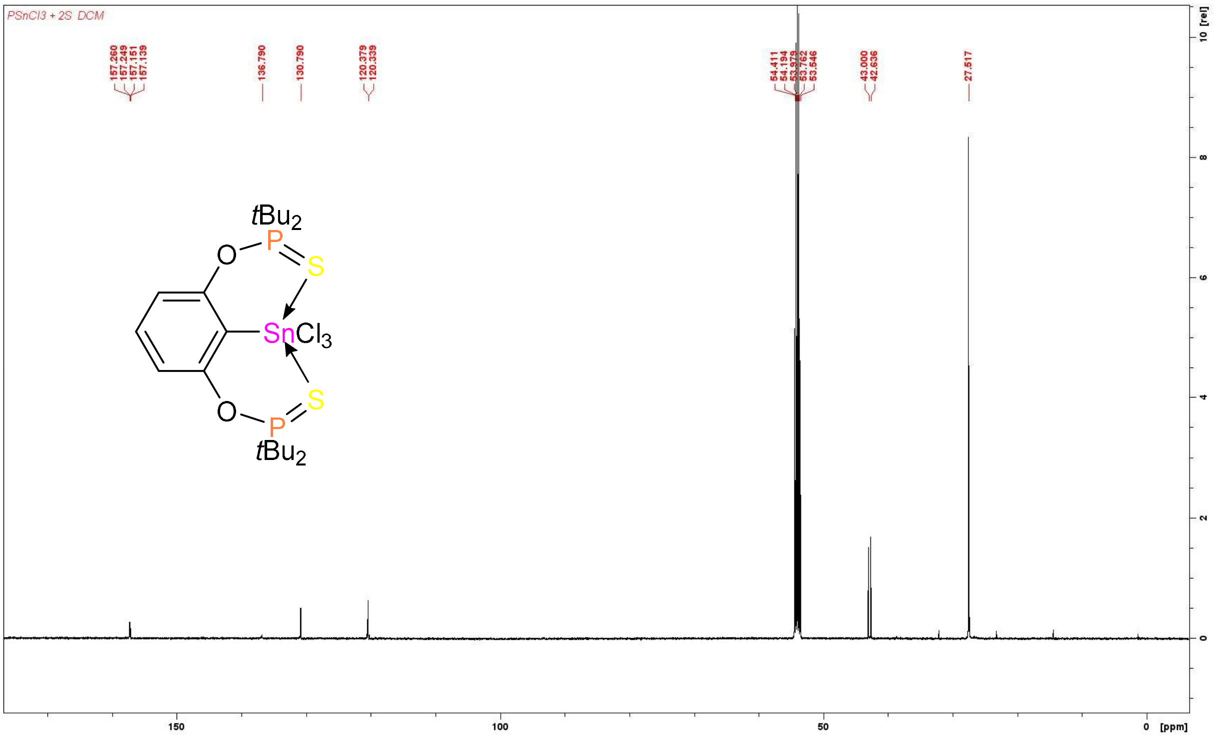

Figure S8. $^{13}C\{^{1}H\}$ NMR spectrum of $\mathbf{1^S}$ (125.76 MHz, CD_2Cl_2).

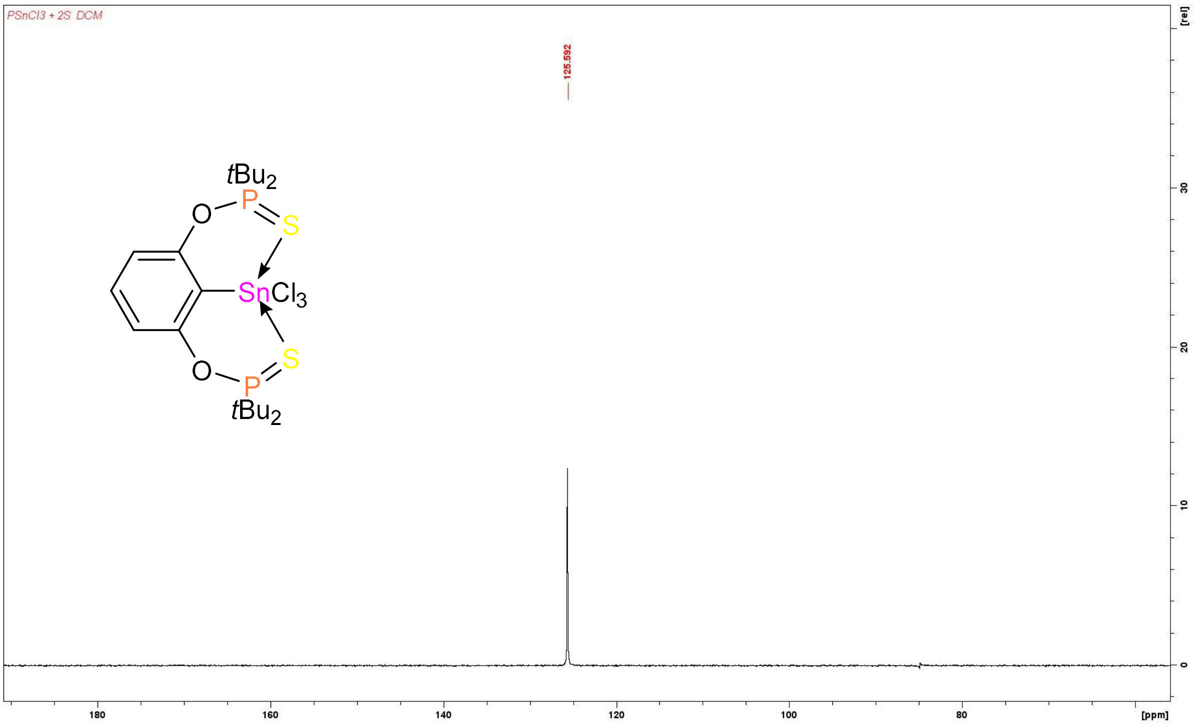

Figure S9. $^{31}P\{^{1}H\}$ NMR spectrum of **1**^{S} (202.5 MHz, CD_2Cl_2).

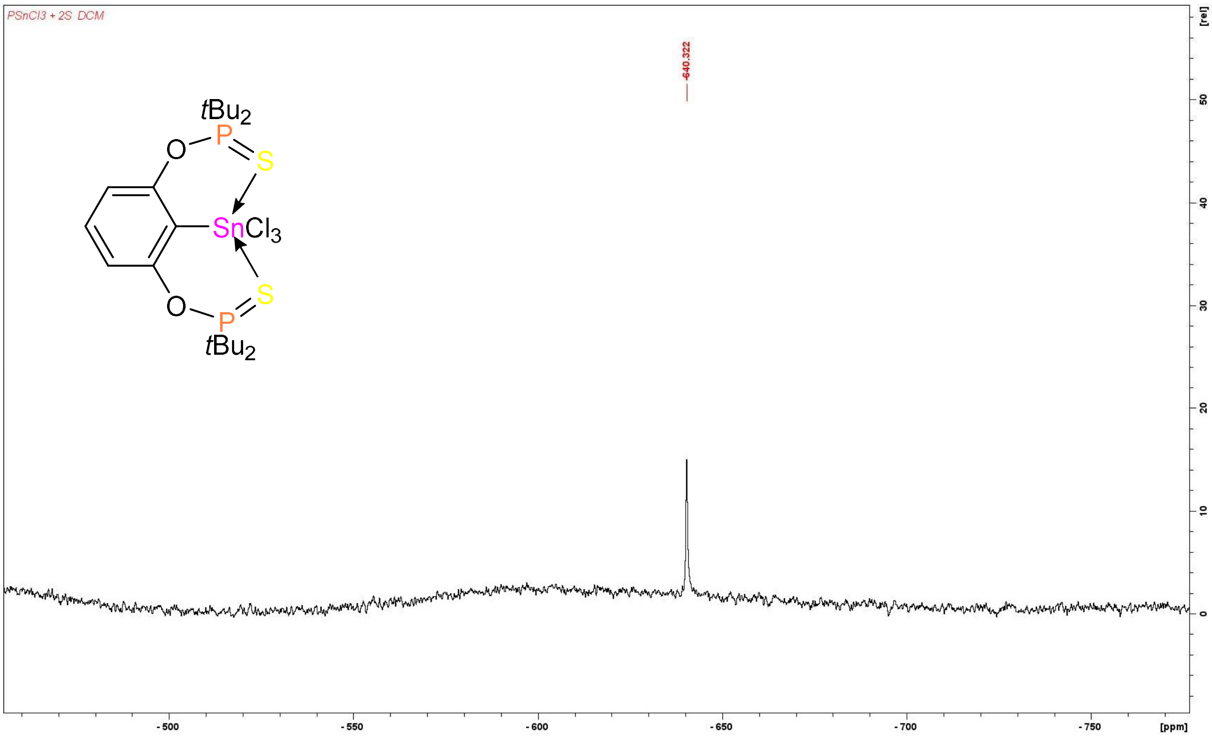

Figure S10. $^{119}Sn\{^{1}H\}$ NMR spectrum of **1**^{S} (186.5MHz, CD_2Cl_2).

Synthesis of [2,6-(*t*Bu$_2$(Se)PO)$_2$C$_6$H$_3$]SnCl$_3$ (1^{Se})

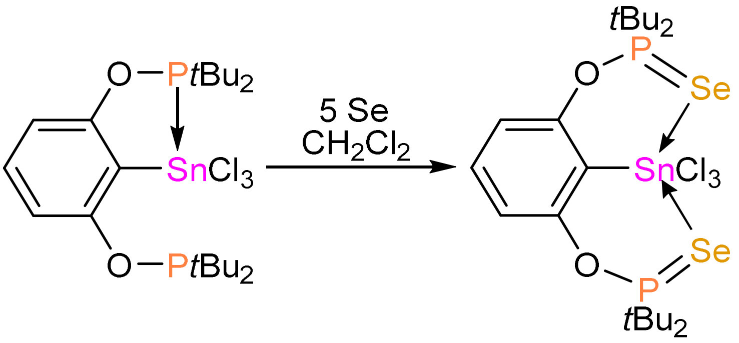

(**1**) (**1^{Se}**)

Elemental selenium (168 mg; 2.13 mmol) was added in one portion to solution of **1** (265 mg; 0.43 mmol) in dichloromethane (20 ml). The reaction mixture was stirred for 24 h at room temperature and then unreacted selenium was removed by filtration. Resulting yellowish solution was concentrated to 1/2 volume and layered with hexane. Crystallization at room temperature gave yellow crystals of compound **1^{Se}**, whereas another batch of crystals could be obtained from the mother liquor by crystallization at -30 °C. Combined yield of **1^{Se}** was 280 mg, (84 %), m. p. 201-204°C. Single-crystals suitable for *sc*-XRD diffraction analysis were obtained from saturated dichloromethane solution at 5°C. Anal. Calcd for $C_{22}H_{39}Cl_3O_2P_2Se_2Sn$ (MW 780.50): C, 33.9; H, 5.0 %. Found: C, 33.8; H, 4.7 %. **^{1}H NMR** (500 MHz, CDCl$_3$) δ (ppm): 1.50 [36H, d, $^{2}J(^{31}P,^{1}H)$ = 1.8 Hz, *t*Bu$_2$(Se)P-C*H*$_3$], 6.96 [2H, d, $^{3}J(^{1}H,^{1}H)$ = 7.8 Hz, $^{3}J(^{119/117}Sn,^{1}H)$ = 41.9 Hz, Ar-*H*], 7.29 [1H, t, $^{3}J(^{1}H,^{1}H)$ = 7.8 Hz, Ar-*H*]. **^{13}C{^{1}H} NMR** (125.78 MHz, CDCl$_3$) δ (ppm): 27.6 [d, $^{3}J(^{31}P,^{13}C)$ = 4.9 Hz, *t*Bu$_2$(Se)P-*C*H$_3$], 43.1 [d, $^{1}J(^{31}P,^{13}C)$ = 35.5 Hz *t*Bu$_2$(Se)P-*C*], 120.4 [d, $^{3}J(^{31}P,^{13}C)$ = 4.2 Hz, $^{3}J(^{119/117}Sn,^{13}C)$ = 38.1 Hz, Ar-*C*], 129.6 [s, Ar-*C*], 133.1 [s, Ar-*C*], 157.0 [d, $^{2}J(^{31}P,^{13}C)$ = 13.6 Hz, Ar-*C*]. **^{31}P{^{1}H} NMR** (202.5 MHz, CDCl$_3$) δ (ppm): 131.0 [s, $^{1}J(^{77}Se,^{31}P)$ = 621 Hz]. **^{77}Se{^{1}H} NMR** (95.4 MHz, CDCl$_3$) δ (ppm): -2.8 [d, $^{1}J(^{77}Se,^{31}P)$ = 621 Hz]. **^{119}Sn{^{1}H} NMR** (186.5 MHz, CDCl$_3$) δ (ppm): -773.4 [s(br)].

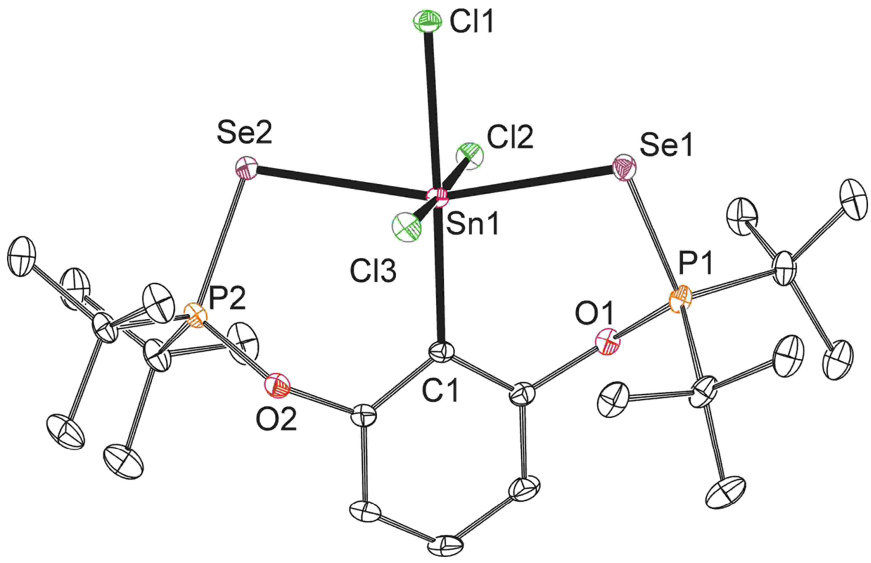

Figure S11. Molecular structure of **1**^{Se}. ORTEP with 30% probability ellipsoid level. Hydrogen atoms and dichloromethane solvate molecules are omitted. Selected structural bond lengths [Å]: Sn(1)-C(1) 2.1962(16), Sn(1)-Cl(1) 2.4765(5), Sn(1)-Cl(2) 2.4529(7), Sn(1)-Cl(3) 2.4445(7), Sn(1)-Se(1) 2.6908(5), Sn(1)-Se(2) 2.7022(5); bonding angles [°]: Cl(1)-Sn(1)-C(1) 176.33(4), Cl(2)-Sn(1)-Cl(3) 176.28(2), Se(1)-Sn(1)-Se(2) 162.02(1).

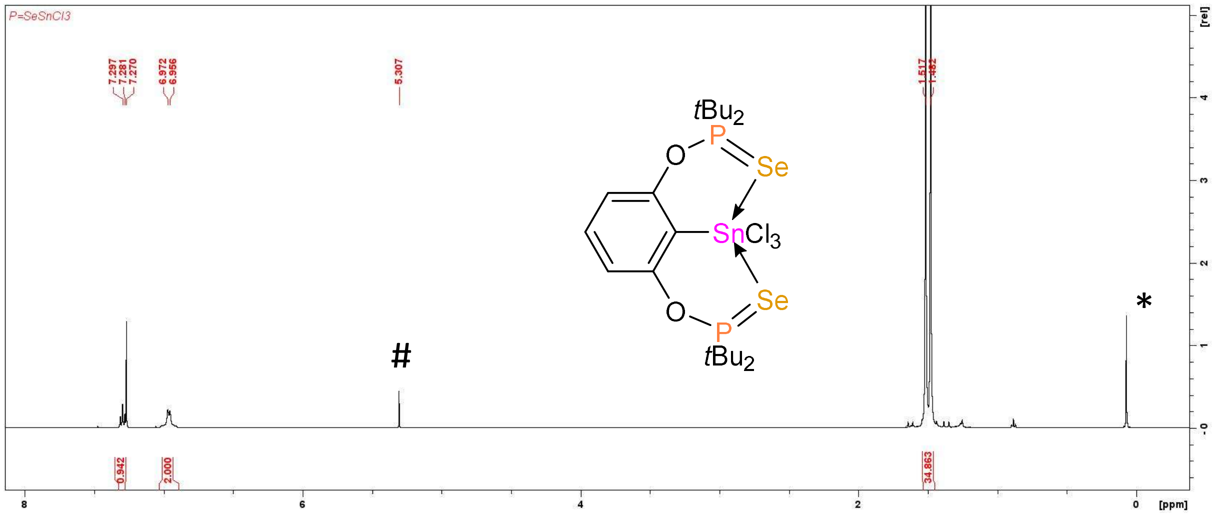

Figure S12. ^{1}H NMR spectrum of **1**^{Se} (500 MHz, $CDCl_3$). *Signal of silicon grease, #signal of co-crystalized dichloromethane.

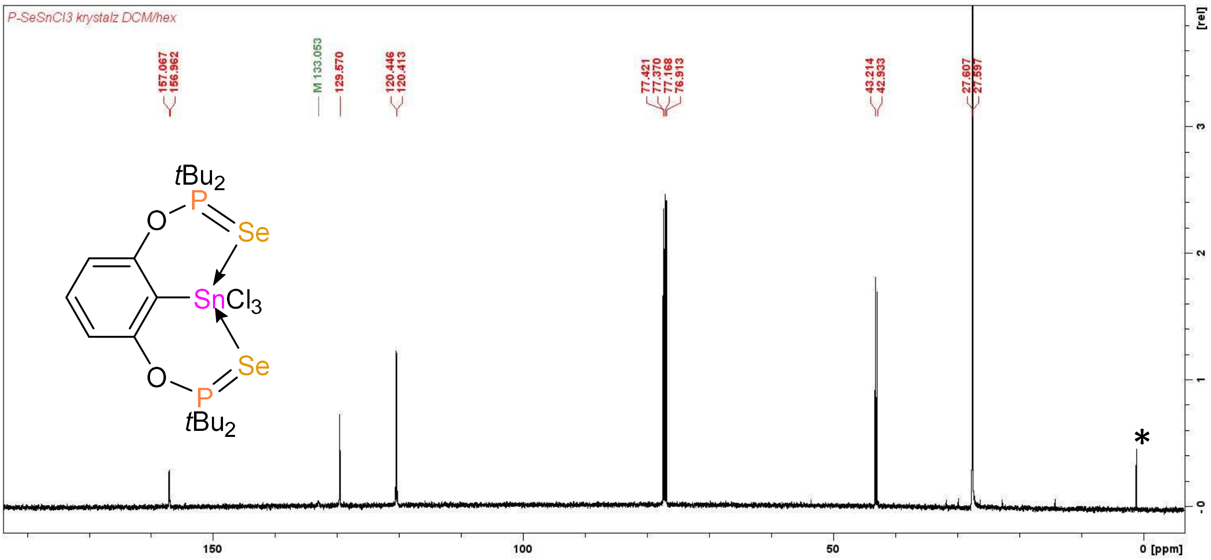

Figure S13. $^{13}C\{^{1}H\}$ NMR spectrum of **1**$^{\mathbf{Se}}$ (125.76 MHz, $CDCl_3$). *Signal of silicon grease.

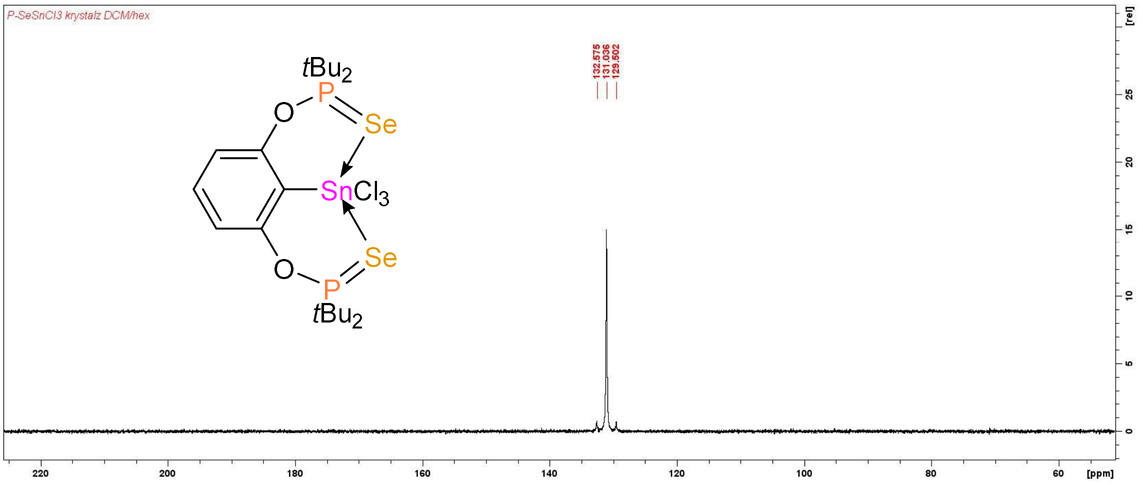

Figure S14. $^{31}P\{^{1}H\}$ NMR spectrum of **1**$^{\mathbf{Se}}$ (202.5 MHz MHz, $CDCl_3$).

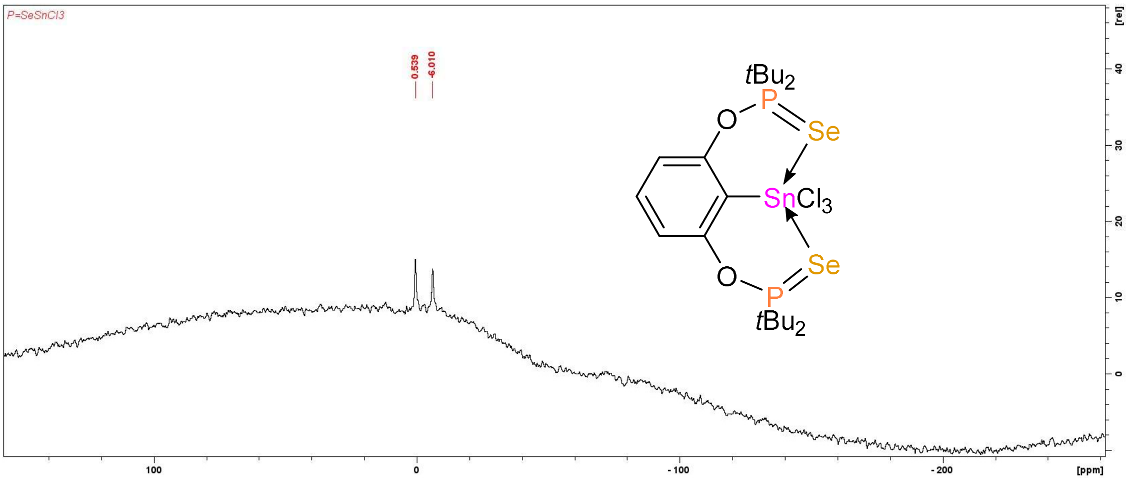

Figure S15. $^{77}Se\{^{1}H\}$ NMR spectrum of **1**$^{\mathbf{Se}}$ (95.4 MHz, $CDCl_3$).

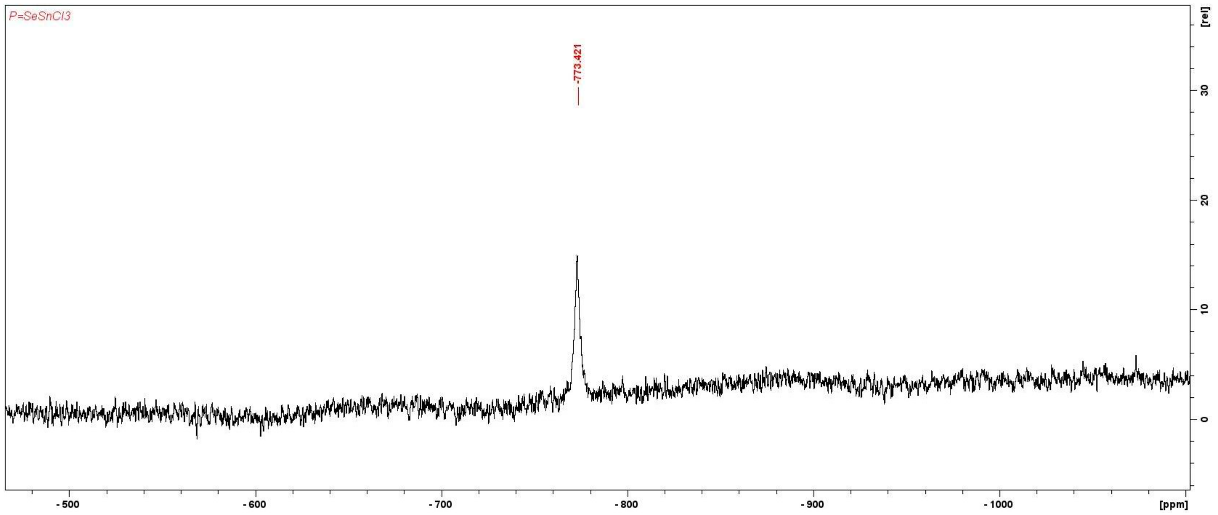

Figure S16. $^{119}Sn\{^{1}H\}$ NMR spectrum of $\mathbf{1^{Se}}$ (186.5MHz, $CDCl_3$).

Synthesis of $\{[2,6\text{-}(t\mathrm{Bu_2(O)PO})_2C_6H_3]SnCl_2\}\{[B[3,5\text{-}(CF_3)_2C_6H_3]_4\}$ ($\mathbf{1^{O+}[BArF]^{-}}$)

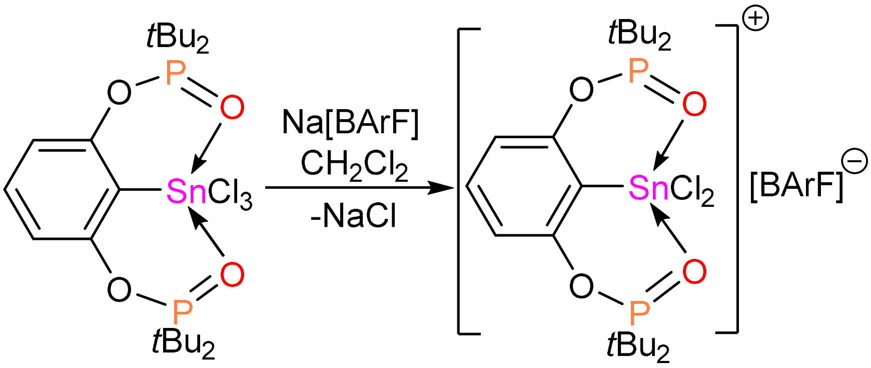

($\mathbf{1^{O}}$) ($\mathbf{1^{O+}[BArF]^{-}}$)

Solid Na[BArF] (42 mg; 0.05 mmol) was added in one portion to solution of $\mathbf{1^{O}}$ (31 mg; 0.05 mmol) in dichloromethane (10 ml). The reaction mixture was stirred for 1 h at room temperature and then incipient NaCl was removed by filtration. Resulting yellowish solution was layered with hexane. Crystallization at room temperature gave colorless crystals of compound $\mathbf{1^{O+}[BArF]^{-}}$. Yield of $\mathbf{1^{O+}[BArF]^{-}}$ was 58 mg, (83 %), m. p. 218-221°C. Single-crystals suitable for *sc*-XRD diffraction analysis were obtained by slow diffusion of hexane into saturated dichloromethane solution at room temperature. Anal. Calcd for $C_{54}H_{51}BCl_2F_{24}O_4P_2Sn$ (MW 1480.23): C, 43.8; H, 3.5 %. Found: C, 44.0; H, 3.8 %. **^{1}H NMR** (500 MHz, $CDCl_3$) δ

(ppm): 1.37 [36H, t, $^{3}J(^{31}P,^{1}H)$ = 16.8 Hz, *t*Bu$_2$P(O)-C*H*$_3$], 6.98 [2H, d, $^{3}J(^{1}H,^{1}H)$ = 8.1 Hz, $^{3}J(^{119/117}Sn,^{1}H)$ = 49.1 Hz, Ar-*H*], 7.37 [1H, t, $^{3}J(^{1}H,^{1}H)$ = 8.1 Hz, Ar-*H*], 7.52 [4H, s, Ar-*H*], 7.71 [8H, s(br), Ar-*H*]. **^{11}B{^{1}H} NMR** (160.42 MHz, CDCl$_3$) δ (ppm): -6.6 [s]. **^{13}C{^{1}H} NMR** (125.78 MHz, CDCl$_3$) δ (ppm): 25.8 [s, *t*Bu$_2$(O)P-*C*H$_3$], 38.2 [d, $^{1}J(^{31}P, ^{13}C)$ = 67.8 Hz, *t*Bu$_2$(O)P-*C*], 117.6 [m, Ar-*C*], 119.4 [d, $^{3}J(^{31}P,^{13}C)$ = 6.6 Hz, $^{3}J(^{119/117}Sn,^{13}C)$ = 51.8 Hz, Ar-*C*], 124.7 [q, $^{1}J(^{19}F,^{13}C)$ = 272 Hz, *C*F$_3$], 128.8 [s, Ar-*C*], 129.0 [qq, $^{2}J(^{19}F,^{13}C)$ = 31.6 Hz, $^{4}J(^{19}F,^{13}C)$ = 3.0 Hz, Ar-*C*], 134.9 [s, Ar-*C*], 136.6 [s, Ar-*C*], 159.1 [d, $^{2}J(^{31}P,^{13}C)$ = 11.8 Hz, Ar-*C*], 161.8 [q, $^{1}J(^{13}C,^{11}B)$ = 50 Hz, Ar-*C*]. **^{19}F{^{1}H} NMR** (376.3 MHz, CDCl$_3$) δ (ppm): -62.4 [s]. **^{31}P{^{1}H} NMR** (202.5 MHz, CDCl$_3$) δ (ppm): 91.8 [s, $^{n}J(^{119/117}Sn,^{31}P)$ = 73.6 Hz]. **^{119}Sn{^{1}H} NMR** (186.5 MHz, CDCl$_3$) δ (ppm): -393.1 [t, $^{n}J(^{119/117}Sn,^{31}P)$ = 73.6 Hz].

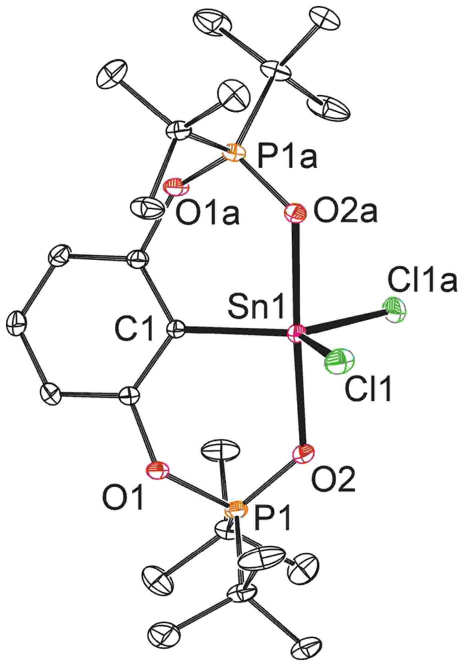

Figure S17. Molecular structure of **1^{O+}[BArF]$^{-}$**. ORTEP with 30% probability ellipsoid level. Hydrogen atoms and [BArF] anion are omitted. Symmetry operator a = 1-x, y, 3/2-z. Selected structural bond lengths [Å]: Sn(1)-C(1) 2.128(2), Sn(1)-Cl(1) 2.2892(7), Sn(1)-O(2) 2.1356(14); bonding angles [°]: O(2)-Sn(1)-O(2a) 177.43(5), C(1)-Sn(1)-Cl(1) 122.73(2), Cl(1)-Sn(1)-Cl(1a) 114.53(3).

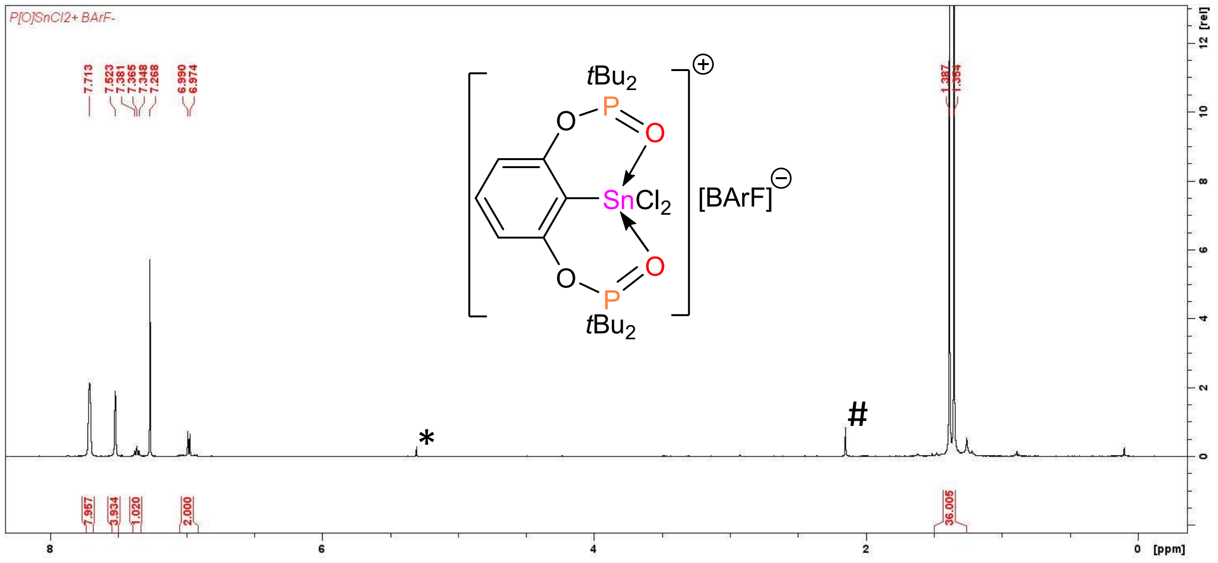

Figure S18. ^{1}H NMR spectrum of **1^{O+}[BArF]$^{-}$** (500 MHz, $CDCl_3$). *Signal of traces of dichloromethane, #unknown minor impurity.

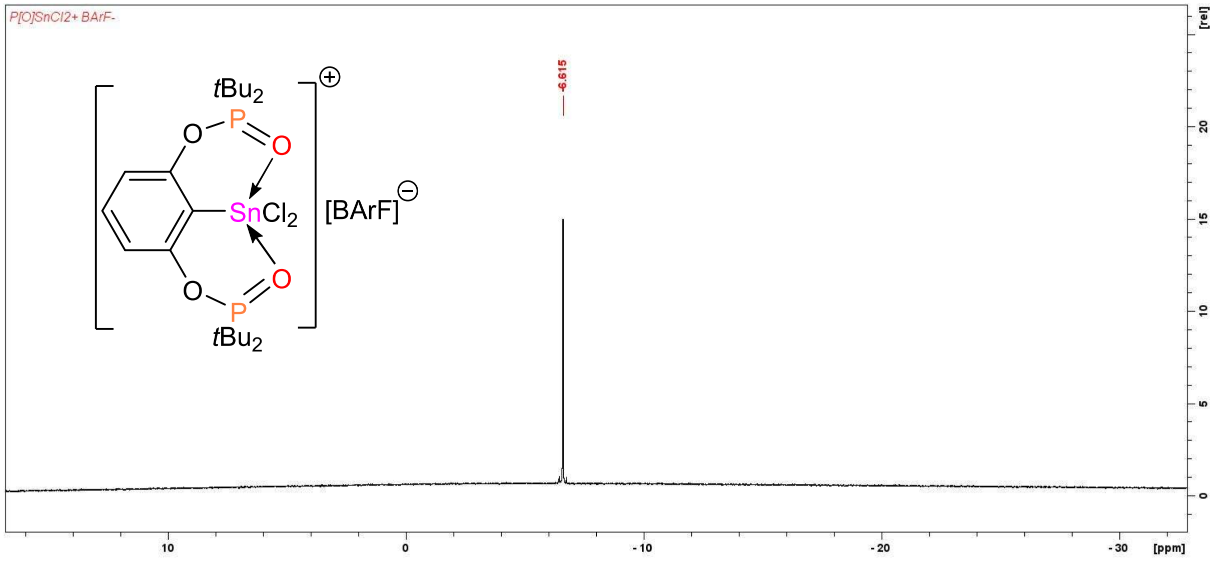

Figure S19. ^{11}B{^{1}H} NMR spectrum of **1^{O+}[BArF]$^{-}$** (160.42 MHz, $CDCl_3$).

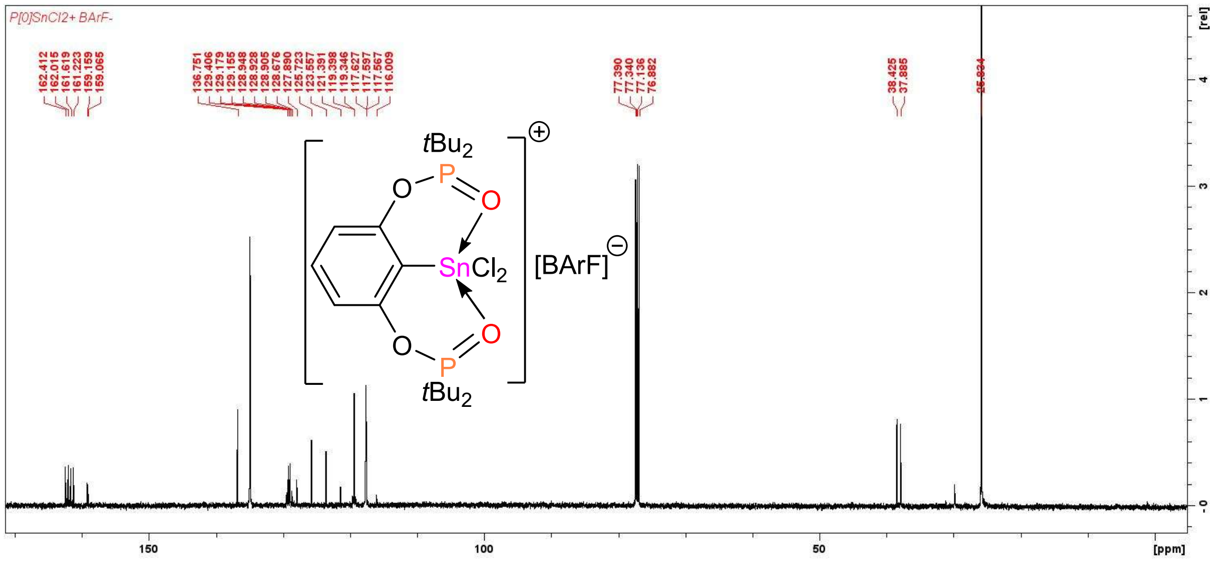

Figure S20. $^{13}C\{^1H\}$ NMR spectrum of **1^{O+}[BArF]$^-$** (125.76 MHz, $CDCl_3$).

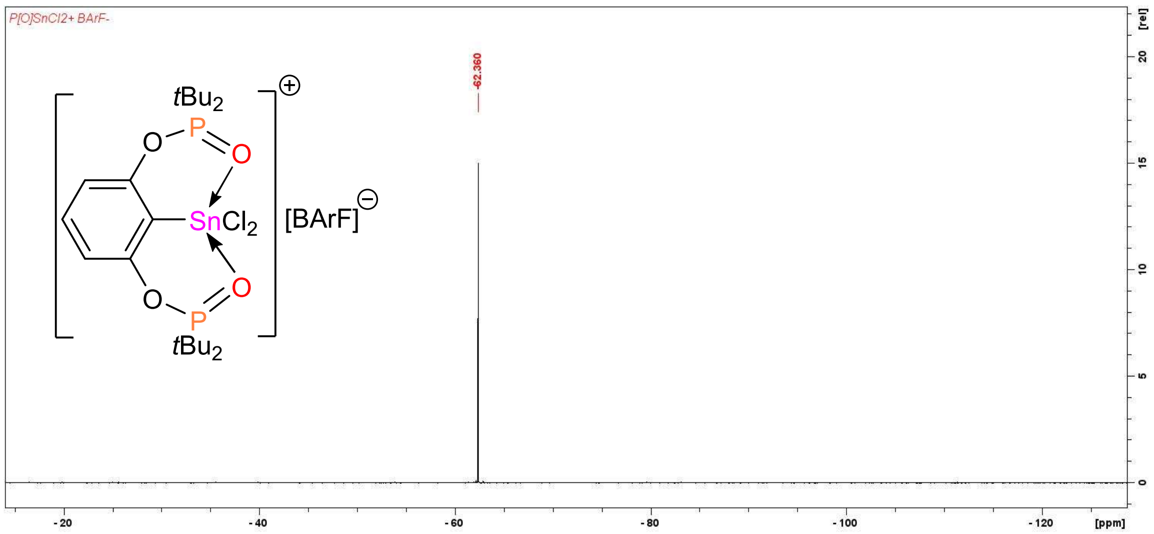

Figure S21. $^{19}F\{^1H\}$ NMR spectrum of **1^{O+}[BArF]$^-$** (470.5 MHz, $CDCl_3$).

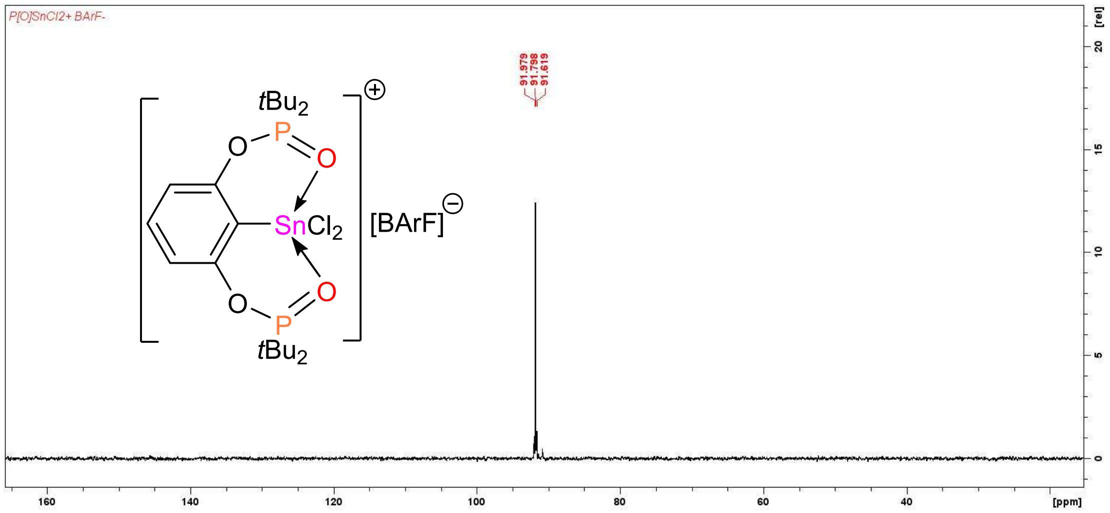

Figure S22. $^{31}P\{^1H\}$ NMR spectrum of **1^{O+}[BArF]$^-$** (202.5 MHz, $CDCl_3$).

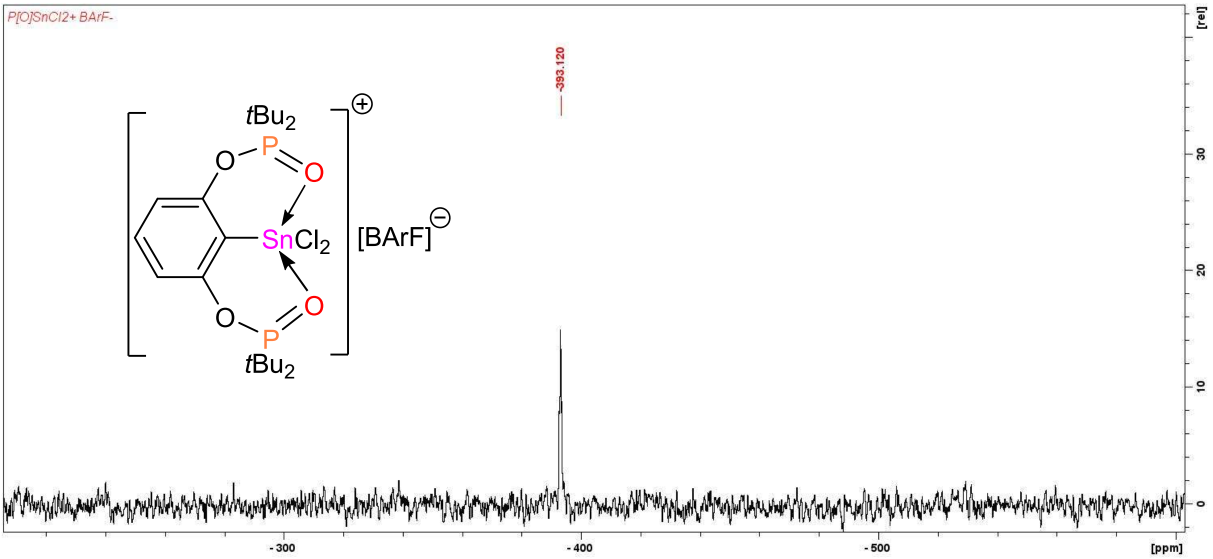

Figure S23. $^{119}Sn\{^{1}H\}$ NMR spectrum of $\mathbf{1^{O+}[BArF]^{-}}$ (186.5 MHz, $CDCl_3$).

Synthesis of {[2,6-(*t*Bu$_2$(S)PO)$_2$C$_6$H$_3$]SnCl$_2$}{[B[3,5-(CF$_3$)$_2$C$_6$H$_3$]$_4$} ($\mathbf{1^{S+}[BArF]^{-}}$)

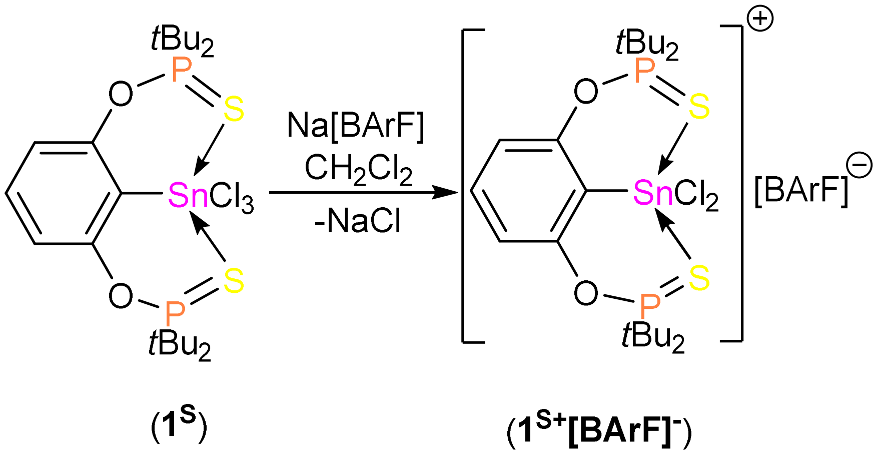

Solid Na[BArF] (107 mg; 0.12 mmol) was added in one portion to solution of $\mathbf{1^{S}}$ (83 mg; 0.12 mmol) in dichloromethane (10 ml). The reaction mixture was stirred for 1 h at room temperature and then incipient NaCl was removed by filtration. Resulting yellowish solution was layered with hexane. Crystallization at room temperature gave yellowish crystals of compound $\mathbf{1^{S+}[BArF]^{-}}$. Yield of $\mathbf{1^{S+}[BArF]^{-}}$ was 115 mg, (63 %), m. p. 249-253°C. Single-crystals suitable for *sc*-XRD diffraction analysis were obtained by slow diffusion of hexane into saturated dichloromethane solution at room temperature. Anal. Calcd for $C_{54}H_{51}BCl_2F_{24}O_2P_2S_2Sn$ (MW 1514.45): C, 42.8; H, 3.4 %. Found: C, 43.1; H, 3.8 %. **^{1}H NMR**

(500 MHz, CD_2Cl_2) δ (ppm): 1.44 [36H, t, $^3J(^{31}P,^1H)$ = 18.8 Hz, *t*Bu$_2$P(S)-C*H*$_3$], 7.29 [2H, m, Ar-*H*], 7.56 [4H, s(br), Ar-*H*], 7.72 [9H, m(br), Ar-*H*]. **^{11}B{^{1}H} NMR** (160.42 MHz, CD_2Cl_2) δ (ppm): -5.0 [s]. **^{13}C{^{1}H} NMR** (125.78 MHz, CD_2Cl_2) δ (ppm): 27.3 [s, *t*Bu$_2$(S)P-*C*H$_3$], 43.4 [d, $^1J(^{31}P, ^{13}C)$ = 40.8 Hz, *t*Bu$_2$(S)P-*C*], 118.0 [m, Ar-*C*], 122.4 [d, $^3J(^{31}P,^{13}C)$ = 3.6 Hz, $^3J(^{119/117}Sn,^{13}C)$ = 45 Hz, Ar-*C*], 125.1 [q, $^1J(^{19}F,^{13}C)$ = 272 Hz, *C*F$_3$], 128.8 [s, Ar-*C*], 129.3 [qq, $^2J(^{19}F,^{13}C)$ = 31.6 Hz, $^4J(^{19}F,^{13}C)$ = 3.0 Hz, Ar-*C*], 135.3 [s, Ar-*C*], 135.9 [s, Ar-*C*], 157.5 [d, $^2J(^{31}P,^{13}C)$ = 14.0 Hz, Ar-*C*], 162.2 [q, $^1J(^{13}C,^{11}B)$ = 50 Hz, Ar-*C*]. **^{19}F{^{1}H} NMR** (376.3 MHz, CD_2Cl_2) δ (ppm): -62.8 [s]. **^{31}P{^{1}H} NMR** (202.5 MHz, CD_2Cl_2) δ (ppm): 133.0 [s]. **^{119}Sn{^{1}H} NMR** (186.5 MHz, CD_2Cl_2) δ (ppm): -371.1 [s].

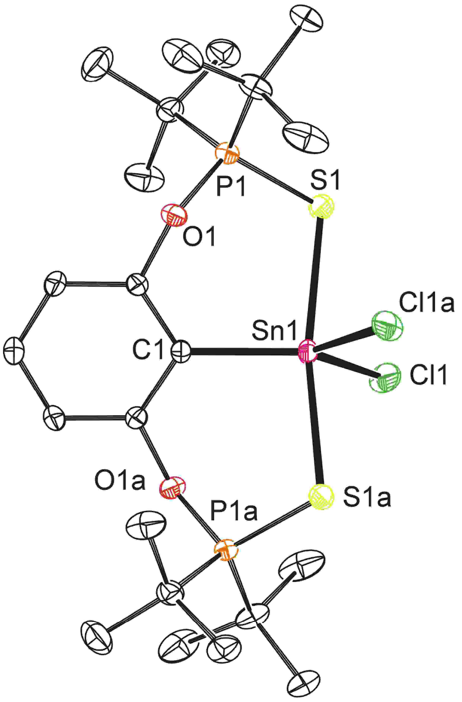

Figure S24. Molecular structure of **1^{S+}[BArF]$^-$**. ORTEP with 30% probability ellipsoid level. Hydrogen atoms and [BArF] anion are omitted. Symmetry operator a = 1-x, y, 3/2-z. Selected structural bond lengths [Å]: Sn(1)-C(1) 2.142(3), Sn(1)-Cl(1) 2.3207(10), Sn(1)-S(1) 2.5907(7); bonding angles [°]: S(1)-Sn(1)-S(1a) 169.94(3), C(1)-Sn(1)-Cl(1) 124.72(2), Cl(1)-Sn(1)-Cl(1a) 110.57(4).

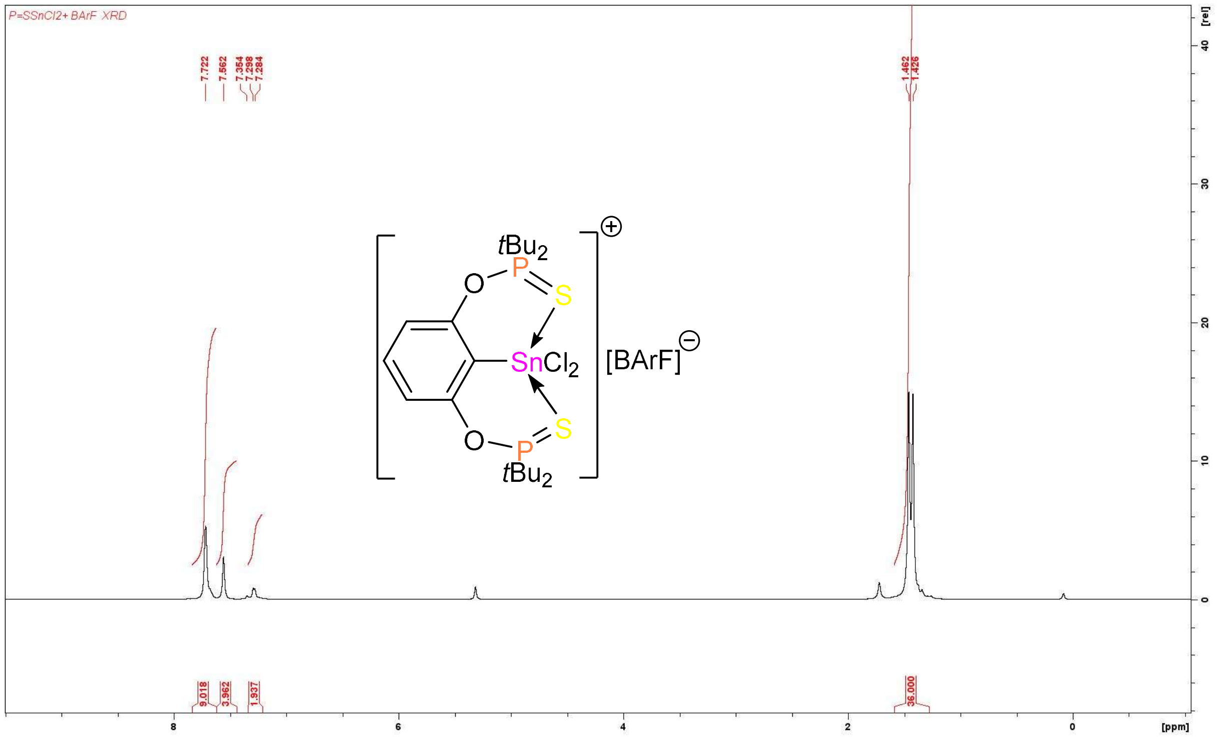

Figure S25. ^{1}H NMR spectrum of $\mathbf{1^{S+}[BArF]^{-}}$ (500 MHz, CD_2Cl_2).

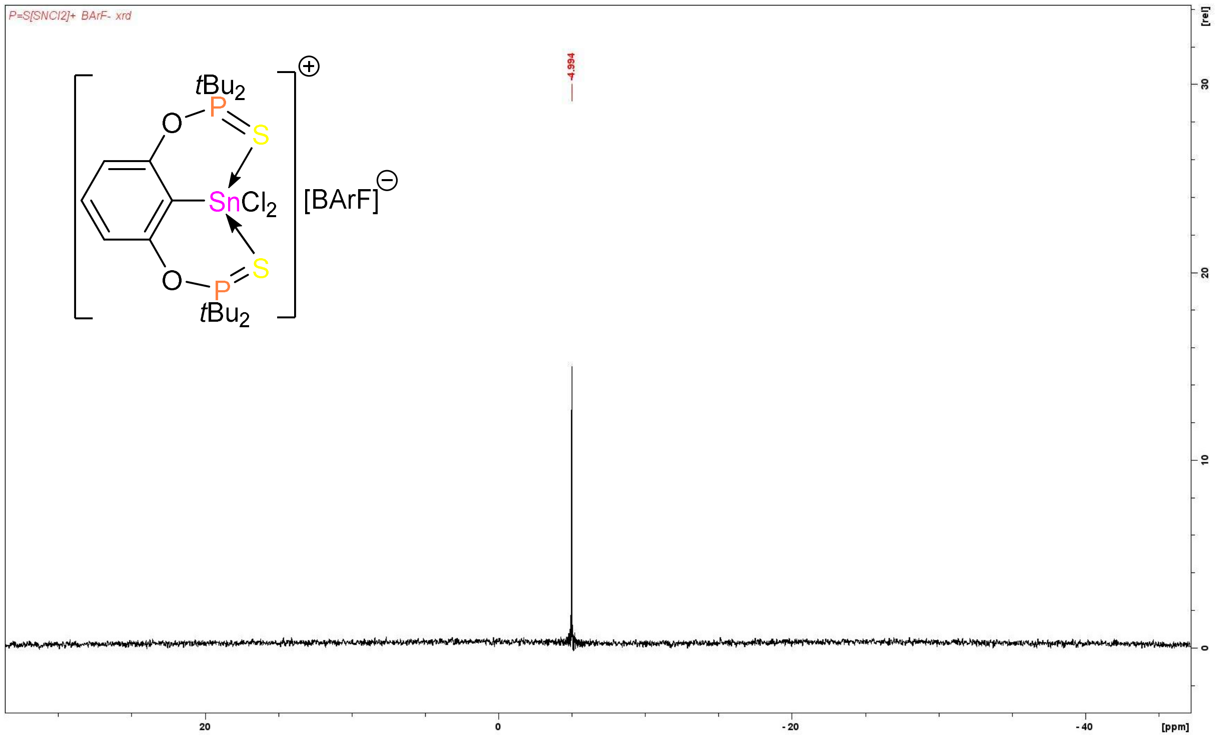

Figure S26. ^{11}B{^{1}H} NMR spectrum of $\mathbf{1^{S+}[BArF]^{-}}$ (160.42 MHz, CD_2Cl_2).

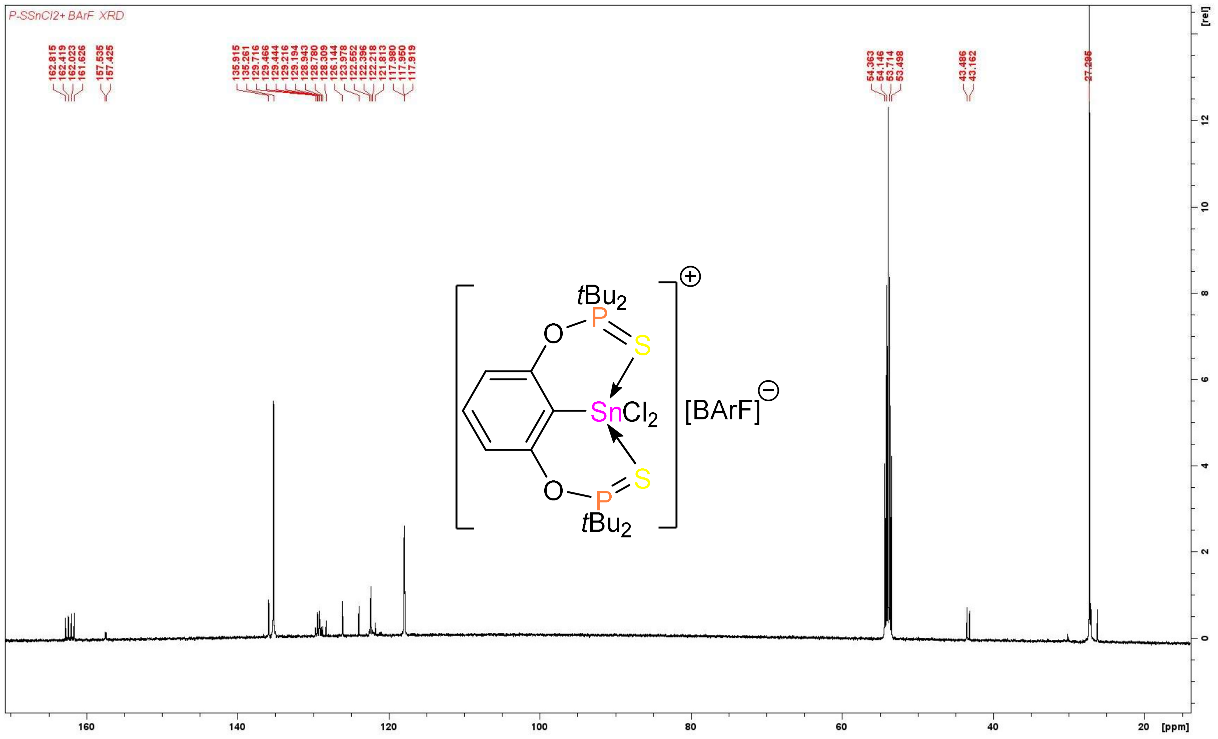

Figure S27. $^{13}C\{^1H\}$ NMR spectrum of $\mathbf{1^{S+}[BArF]^-}$ (125.76 MHz, CD_2Cl_2).

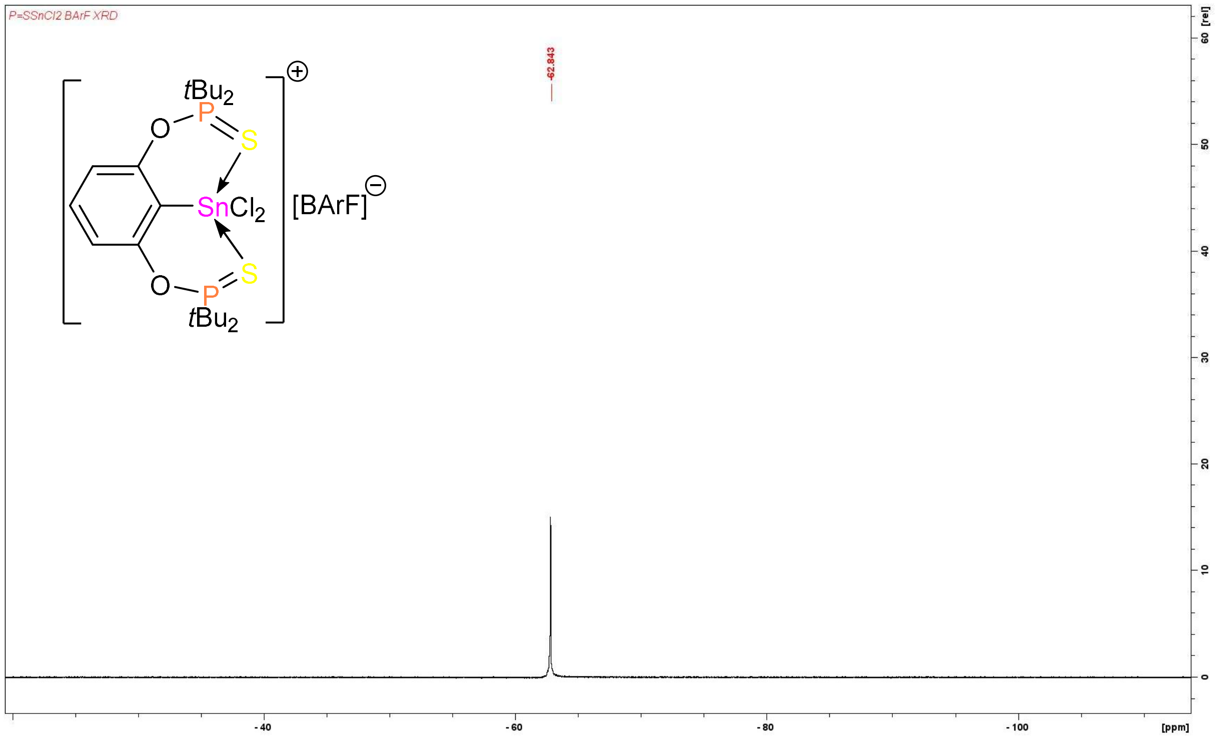

Figure S28. $^{19}F\{^1H\}$ NMR spectrum of $\mathbf{1^{S+}[BArF]^-}$ (470.5 MHz, CD_2Cl_2).

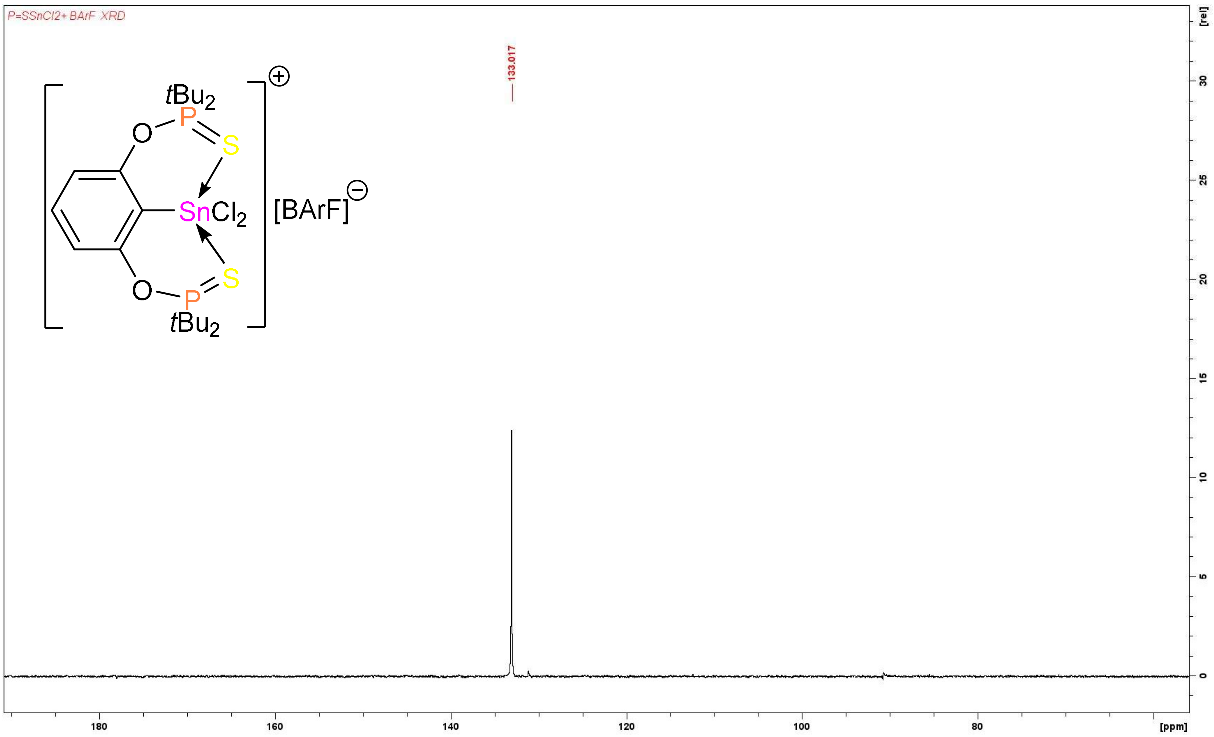

Figure S29. $^{31}P\{^{1}H\}$ NMR spectrum of $\mathbf{1^{S+}[BArF]^{-}}$ (202.5 MHz, CD_2Cl_2).

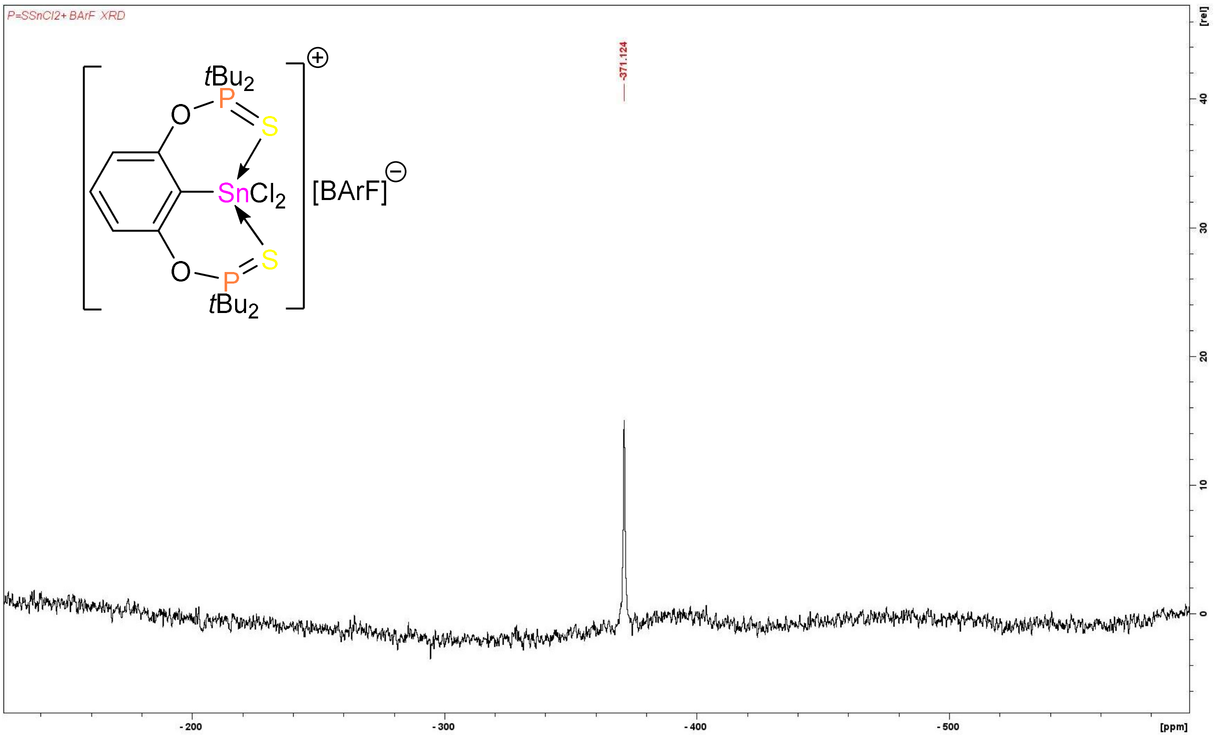

Figure S30. $^{119}Sn\{^{1}H\}$ NMR spectrum of $\mathbf{1^{S+}[BArF]^{-}}$ (186.5 MHz, CD_2Cl_2).

Synthesis of {[2,6-(tBu$_2$(Se)PO)$_2$C$_6$H$_3$]SnCl$_2$}{[B[3,5-(CF$_3$)$_2$C$_6$H$_3$]$_4$} (1^{Se+}[BArF]$^-$)

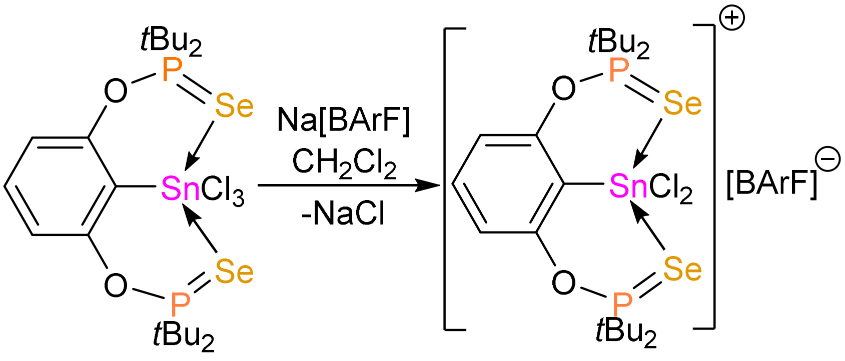

Solid Na[BArF] (209 mg; 0.24 mmol) was added in one portion to solution of $\mathbf{1^{Se}}$ (184 mg; 0.24 mmol) in dichloromethane (15 ml). The reaction mixture was stirred for 30 min at room temperature and then incipient NaCl was removed by filtration. Resulting yellowish solution was layered with hexane. Crystallization at room temperature gave yellow crystals of compound $\mathbf{1^{Se+}[BArF]^-}$. Yield of $\mathbf{1^{Se+}[BArF]^-}$ was 312 mg, (82 %), m. p. 206-208°C. Single-crystals suitable for *sc*-XRD diffraction analysis were obtained by slow diffusion of hexane into saturated dichloromethane solution at room temperature. Anal. Calcd for $C_{54}H_{51}BCl_2F_{24}O_2P_2Se_2Sn$ (MW 1608.27): C, 40.3; H, 3.2 %. Found: C, 40.4; H, 3.0 %. **^{1}H NMR** (500 MHz, CD$_3$CN) δ (ppm): 1.46 [36H, t, $^3J(^{31}P,^1H)$ = 18.4 Hz, tBu$_2$(Se)P-CH_3], 7.33 [2H, d, $^3J(^1H,^1H)$ = 8.3 Hz, $^3J(^{119/117}Sn,^1H)$ = 48.6 Hz, Ar-H], 7.58 [1H, t, $^3J(^1H,^1H)$ = 8.3 Hz, Ar-H], 7.67 [4H, s, Ar-H], 7.69 [8H, s(br), Ar-H]. **^{11}B{^{1}H} NMR** (160.42 MHz, CD$_3$CN) δ (ppm): -6.7 [s]. **^{13}C{^{1}H} NMR** (125.78 MHz, CD$_3$CN) δ (ppm): 27.2 [s, tBu$_2$(Se)P-CH$_3$], 44.1 [d, $^1J(^{31}P,^{13}C)$ = 31.2 Hz tBu$_2$(Se)P-C], 118.8 [m, Ar-C], 123.1 [d, $^3J(^{31}P,^{13}C)$ = 4.9 Hz, $^3J(^{119/117}Sn,^{13}C)$ = 37.6 Hz, Ar-C], 125.6 [q, $^1J(^{19}F,^{13}C)$ = 272 Hz, CF$_3$], 130.0 [qq, $^2J(^{19}F,^{13}C)$ = 31.5 Hz, $^4J(^{19}F,^{13}C)$ = 2.8 Hz, Ar-C], 133.9 [s, Ar-C], 135.7 [s, Ar-C], 157.8 [d, $^2J(^{31}P,^{13}C)$ = 13.7 Hz, Ar-C], 162.7 [q, $^1J(^{13}C,^{11}B)$ = 49.6 Hz, Ar-C]. **^{19}F{^{1}H} NMR** (376.3 MHz, CD$_3$CN) δ (ppm): -63.2 [s]. **^{31}P{^{1}H} NMR** (202.5 MHz, CD$_3$CN) δ (ppm): 134.4 [s, $^1J(^{77}Se,^{31}P)$ = 576 Hz].

^{77}Se{^{1}H} NMR (95.4 MHz, CD_3CN) δ (ppm): 6.6 [d, $^{1}J(^{77}Se,^{31}P)$ = 576 Hz]. **^{119}Sn{^{1}H} NMR** (186.5 MHz, CD_3CN) δ (ppm): -692.1 [s(br)].

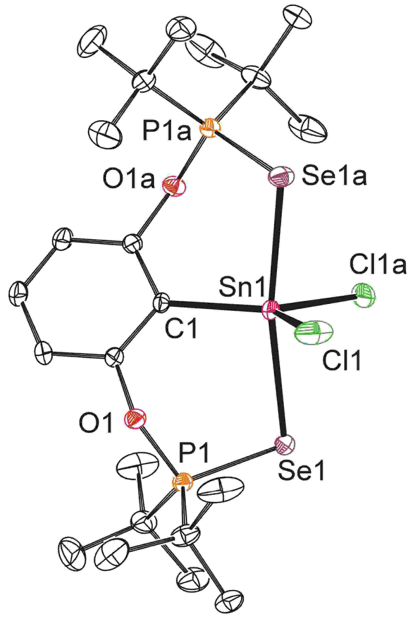

Figure S31. Molecular structure of **1^{Se+}[BArF]$^{-}$**. ORTEP with 30% probability ellipsoid level. Hydrogen atoms and [BArF] anion are omitted. Symmetry operator a = -x, y, 1/2-z. Selected structural bond lengths [Å]: Sn(1)-C(1) 2.153(3), Sn(1)-Cl(1) 2.3320(12), Sn(1)-Se(1) 2.6917(4); bonding angles [°]: Se(1)-Sn(1)-Se(1a) 169.61(2), C(1)-Sn(1)-Cl(1) 125.03(3), Cl(1)-Sn(1)-Cl(1a) 109.94(4).

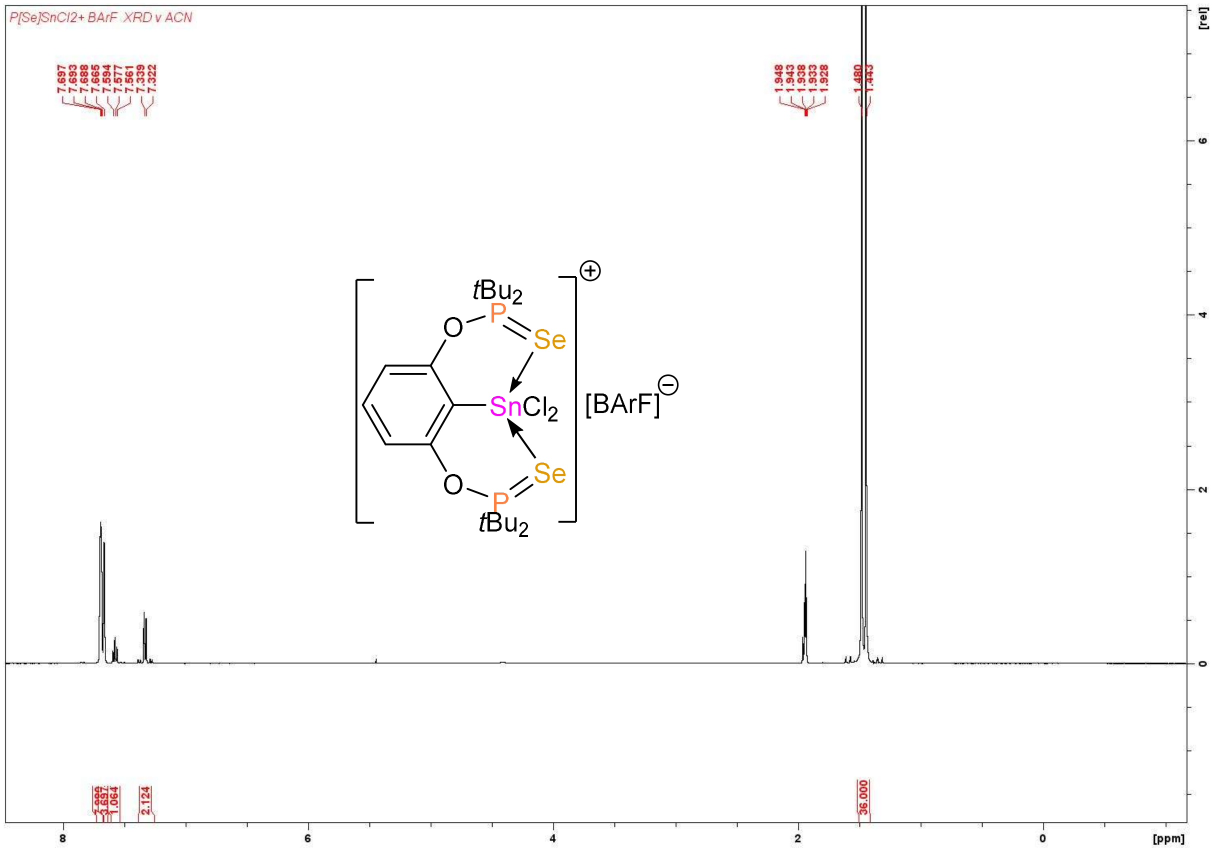

Figure S32. ^{1}H NMR spectrum of **1^{Se+}[BArF]$^{-}$** (500 MHz, CD_3CN).

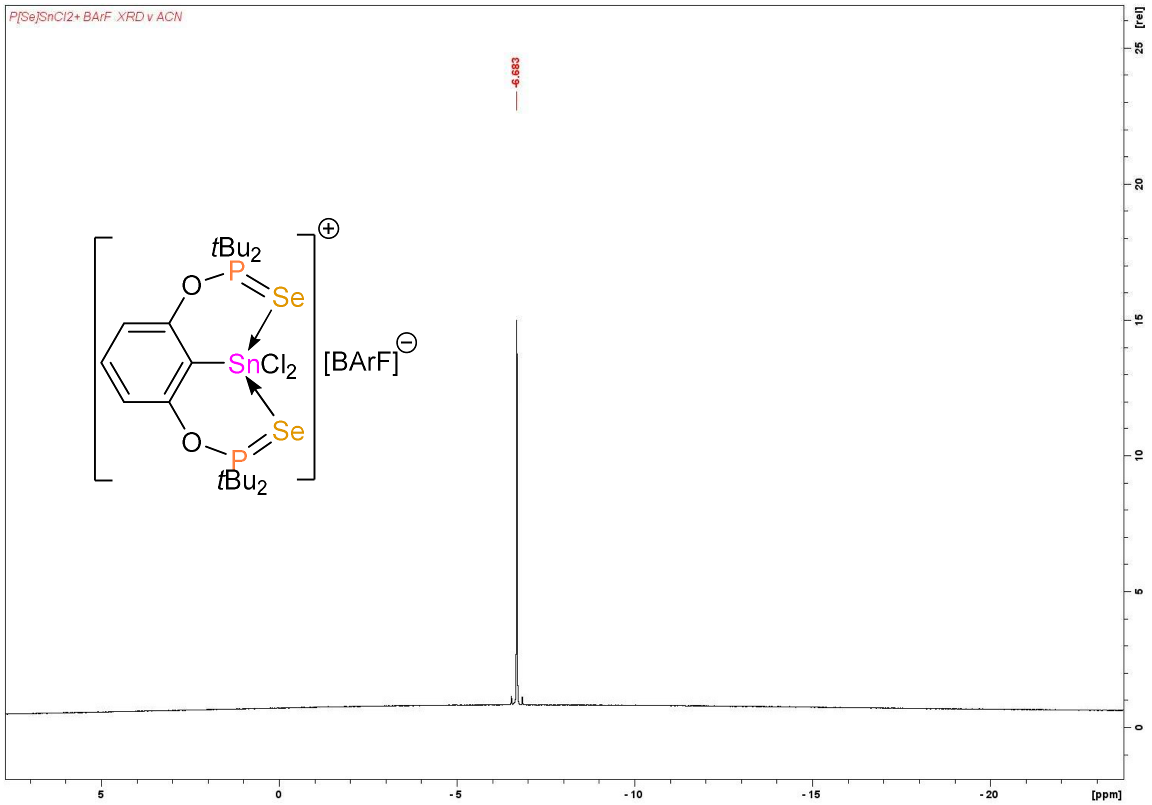

Figure S33. ^{11}B{^{1}H} NMR spectrum of **1^{Se+}[BArF]$^{-}$** (160.42 MHz, CD_3CN).

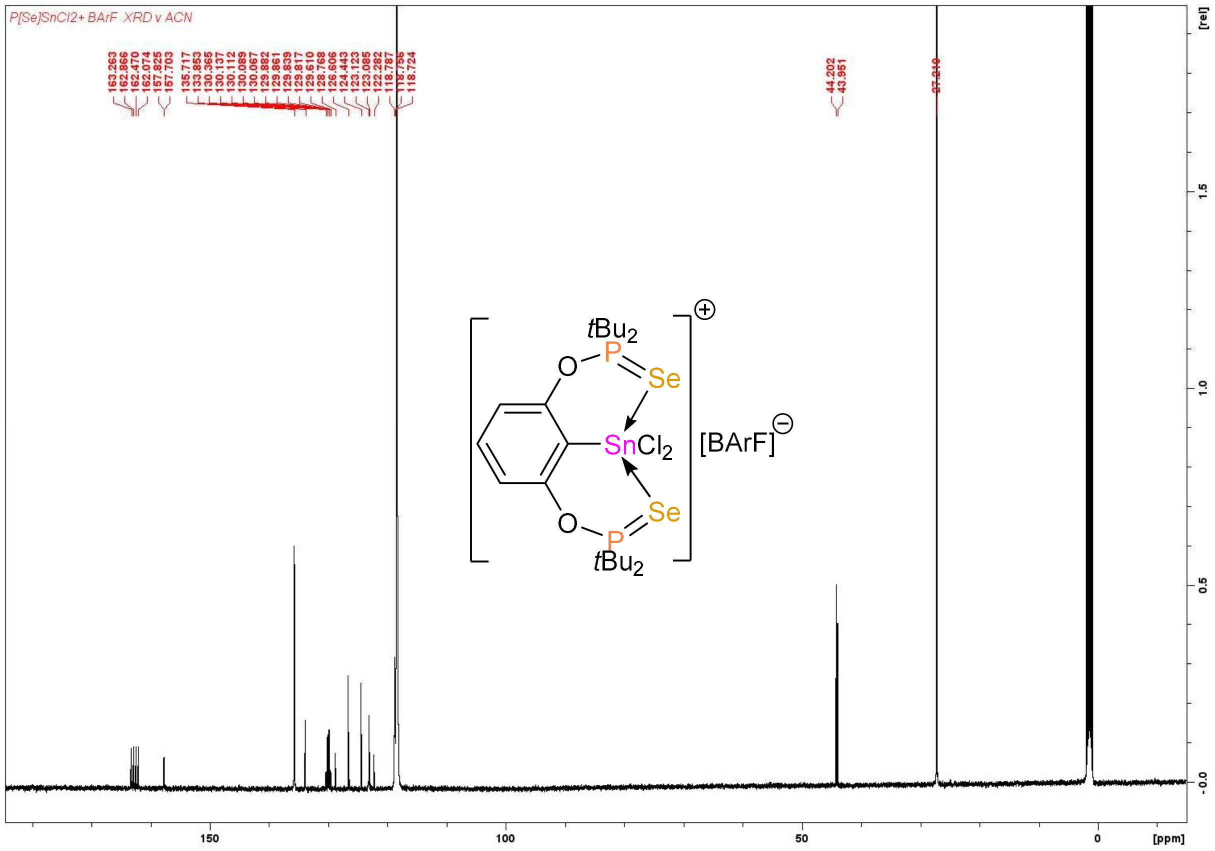

Figure S34. $^{13}C\{^{1}H\}$ NMR spectrum of $\mathbf{1^{Se+}[BArF]^{-}}$ (125.76 MHz, CD_3CN).

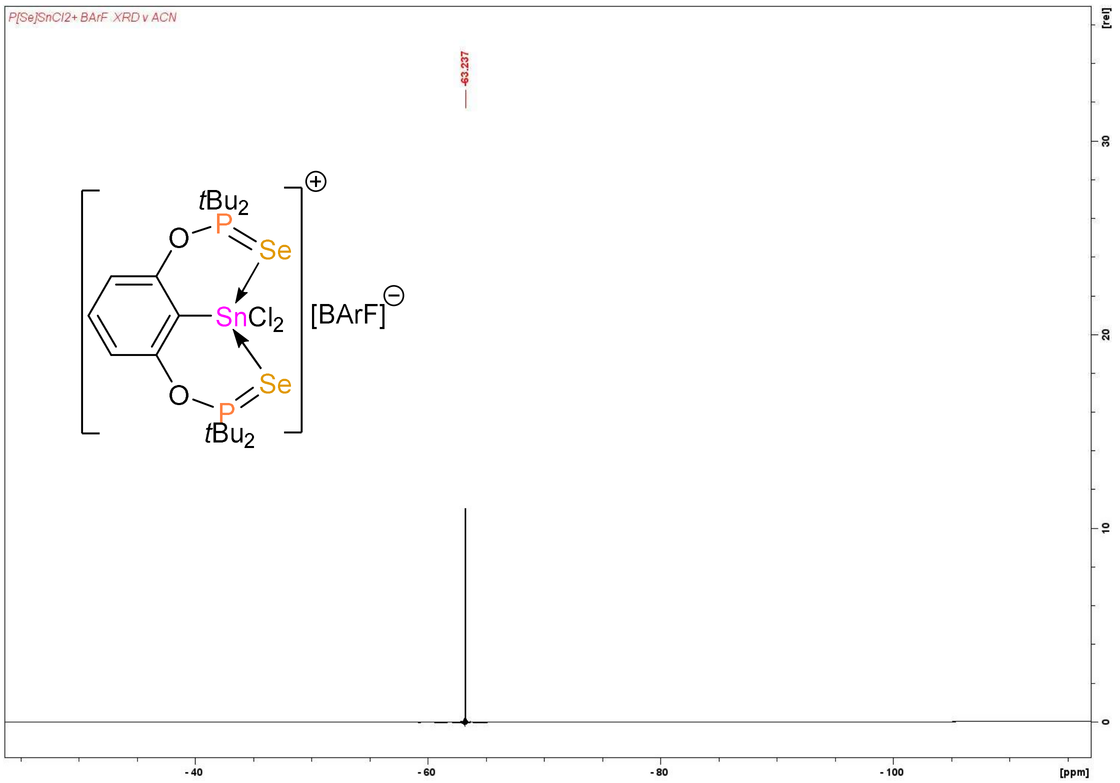

Figure S35. $^{19}F\{^{1}H\}$ NMR spectrum of $\mathbf{1^{Se+}[BArF]^{-}}$ (470.5 MHz, CD_3CN).

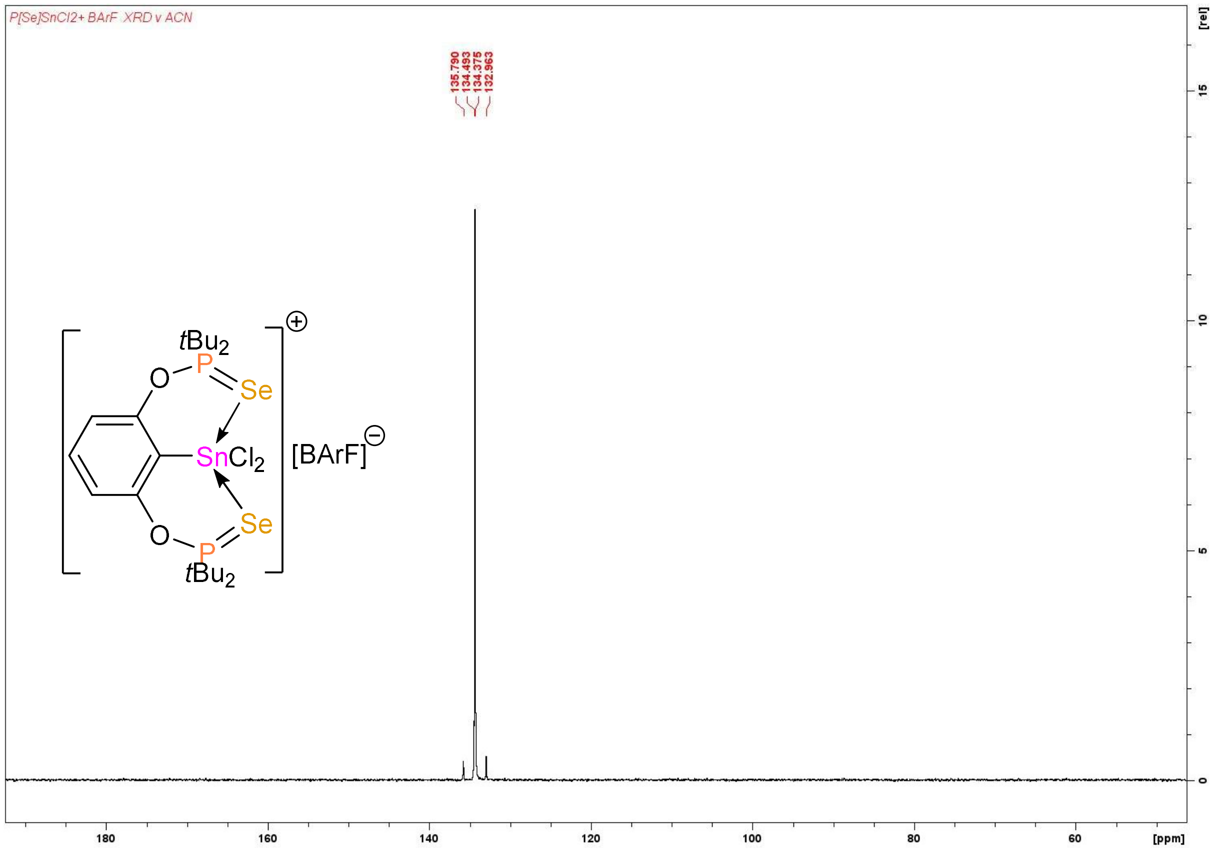

Figure S36. $^{31}P\{^{1}H\}$ NMR spectrum of $\mathbf{1^{Se+}[BArF]^{-}}$ (202.5 MHz, CD_3CN).

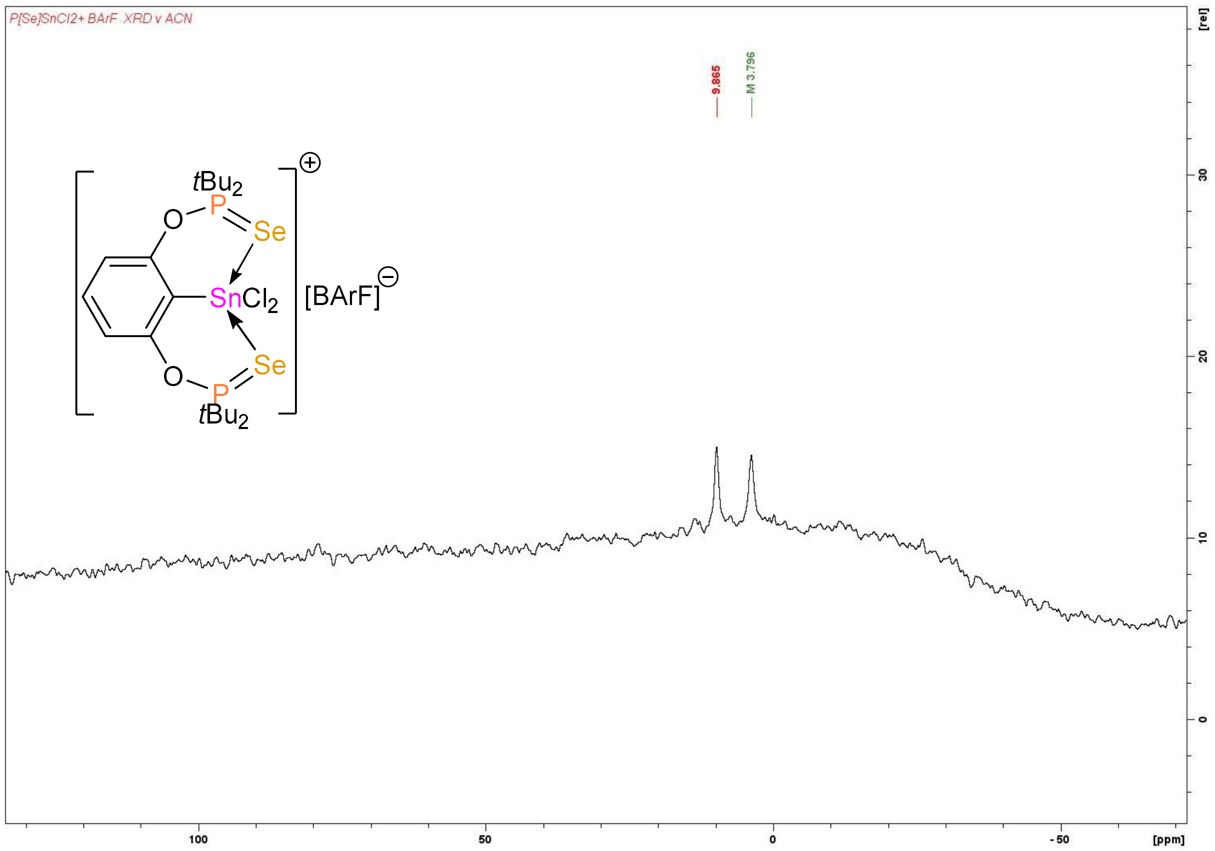

Figure S37. $^{77}Se\{^{1}H\}$ NMR spectrum of $\mathbf{1^{Se+}[BArF]^{-}}$ (95.4 MHz, CD_3CN).

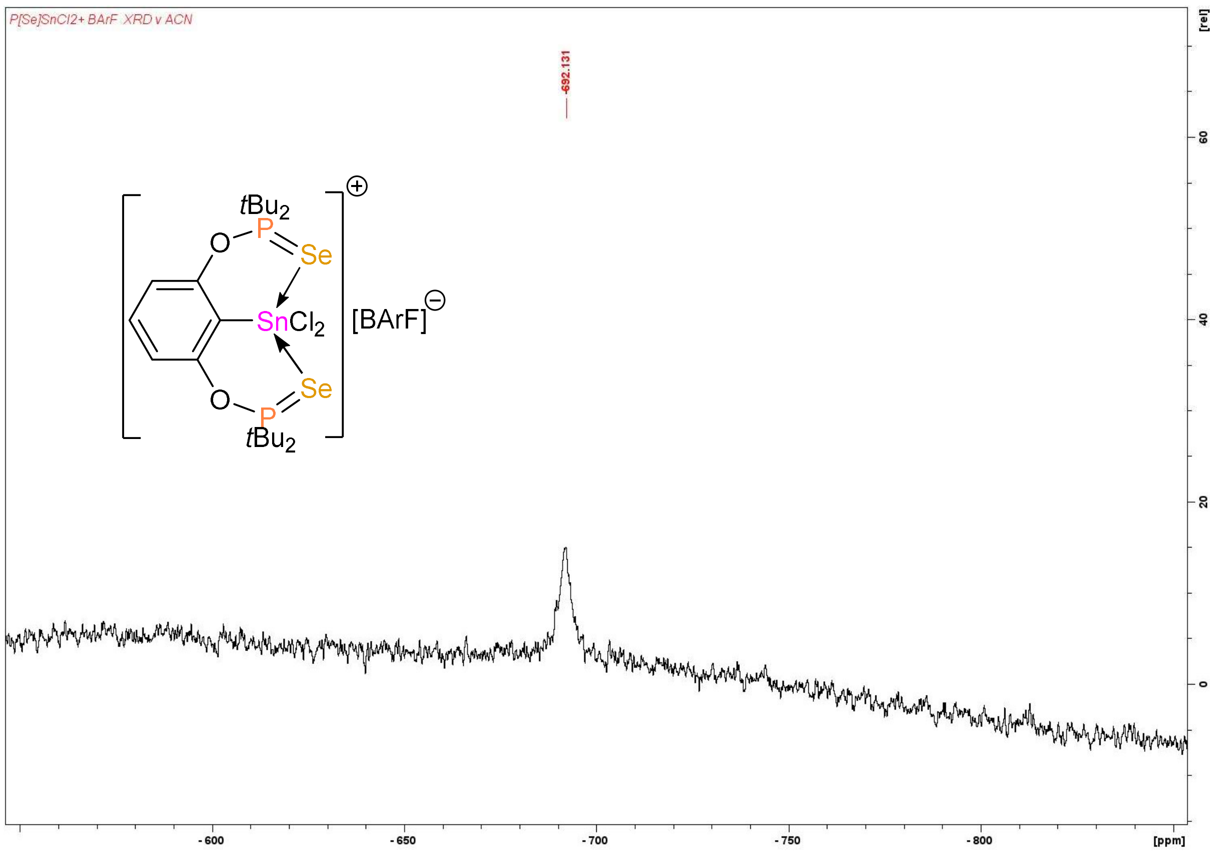

Figure S38. ^{119}Sn{^{1}H} NMR spectrum of $\mathbf{1^{Se+}[BArF]^-}$ (186.5 MHz, CD_3CN).

Synthesis of [2,6-(tBu$_2$(O)PO)$_2$C$_6$H$_3$]SnPhCl$_2$ ($\mathbf{2^O}$)

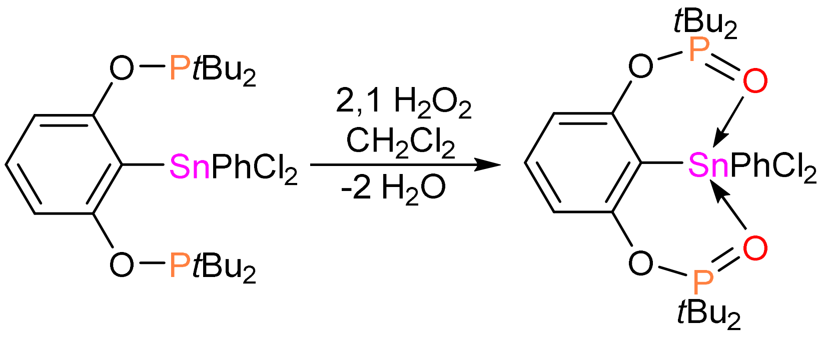

(**2**) (**2^O**)

Solution of **2** (237 mg; 0.36 mmol) in dichloromethane (20 ml) was transferred to Schlenk flask with dried molecular sieves. 30% solution of hydrogen peroxide in water (76 µl; 0.7465 mmol) was slowly added, while resulting mixture was stirred vigorously. The reaction mixture was stirred for 5 min at room temperature and then molecular sieves were removed by filtration.

Solvent was removed in vacuo and white powder was rigorously dried to remove any excess traces of water. Recrystallisation from dichloromethane/hexane solution gave colorless crystals of compound **2^O^**. Yield of **2^O^** was 221 mg, (89 %), m. p. 218-222°C. Single-crystals suitable for *sc*-XRD diffraction analysis were obtained from saturated solution using dichloromethane/hexane mixture at -30°C. Anal. Calcd for $C_{28}H_{44}Cl_2O_4P_2Sn$ (MW 696.21): C, 48.3; H, 6.4 %. Found: C, 48.6; H, 6.6 %. **^{1}H NMR** (500 MHz, $CDCl_3$) δ (ppm): 1.50 [36H, d, $^{3}J(^{31}P,^{1}H)$ = 15.4 Hz, *t*Bu_2P(O)-C*H_3*], 6.91 [2H, d, $^{3}J(^{1}H,^{1}H)$ = 8.1 Hz, $^{4}J(^{119/117}Sn, ^{1}H)$ = 40.3 Hz, Ar-*H*], 7.23 [1H, t, $^{3}J(^{1}H,^{1}H)$ = 8.1 Hz, Ar-*H*], 7.30 [1H, m, Ar-*H*], 7.37 [2H, t, $^{3}J(^{1}H,^{1}H)$ = 7.4 Hz, Ar-*H*], 8.08 [2H, d, $^{3}J(^{1}H,^{1}H)$ = 6.9 Hz, $^{3}J(^{119/117}Sn, ^{1}H)$ = 141.1 Hz Ar-*H*]. **^{13}C{^{1}H} NMR** (125.78 MHz, $CDCl_3$) δ (ppm): 27.4 [s, *t*Bu_2P(O)-*C*H_3], 37.8 [d, $^{1}J(^{31}P,^{13}C)$ = 74.0 Hz, *t*Bu_2P(O)-*C*], 118.3 [d, $^{3}J(^{31}P,^{13}C)$ = 6.8 Hz, $^{3}J(^{119/117}Sn, ^{13}C)$ = 53.6 Hz, Ar-*C*], 127.8 [s, $^{3}J(^{119/117}Sn,^{13}C)$ = 153.0/144.6 Hz, Ar-*C*], 128.1 [s, $^{4}J(^{119/117}Sn, ^{13}C)$ = 29.9 Hz, Ar-*C*], 130.0 [s, Ar-*C*], 133.6 [s, $^{2}J(^{119/117}Sn,^{13}C)$ = 85.8/81.5 Hz, Ar-*C*], 137.5 [s, Ar-*C*], 157.1 [t, $^{3}J(^{31}P,^{13}C)$ = 3.7 Hz, Ar-*C*], 157.4 [d, $^{2}J(^{31}P,^{13}C)$ = 10.9 Hz, Ar-*C*]. **^{31}P{^{1}H} NMR** (202.5 MHz, $CDCl_3$) δ (ppm): 82.9 [s, $^{n}J(^{119/117}Sn, ^{31}P)$ = 66.9 Hz]. **^{119}Sn{^{1}H} NMR** (186.5 MHz, $CDCl_3$) δ (ppm): -529.5 [t(br), $^{n}J(^{119/117}Sn, ^{31}P)$ = 66.9 Hz].

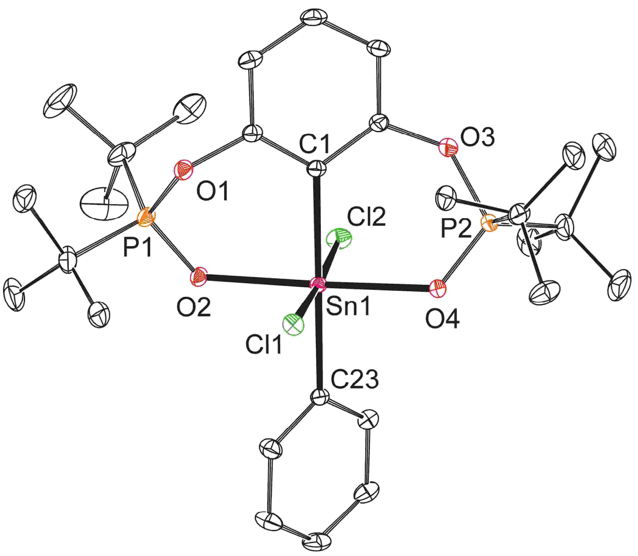

Figure S39. Molecular structure of **2^O^**. ORTEP with 30% probability ellipsoid level. Hydrogen atoms and dichloromethane solvate molecule are omitted. Selected structural bond lengths [Å]: Sn(1)-C(1) 2.1753(18), Sn(1)-Cl(1) 2.5242(5), Sn(1)-Cl(2) 2.4968(5), Sn(1)-C(23) 2.1324(18), Sn(1)-O(2) 2.2003(13), Sn(1)-O(4) 2.1919(14); bonding angles [°]: C(1)-Sn(1)-C(23) 179.33(7), Cl(1)-Sn(1)-Cl(2) 177.09(2), O(2)-Sn(1)-O(4) 178.15(5).

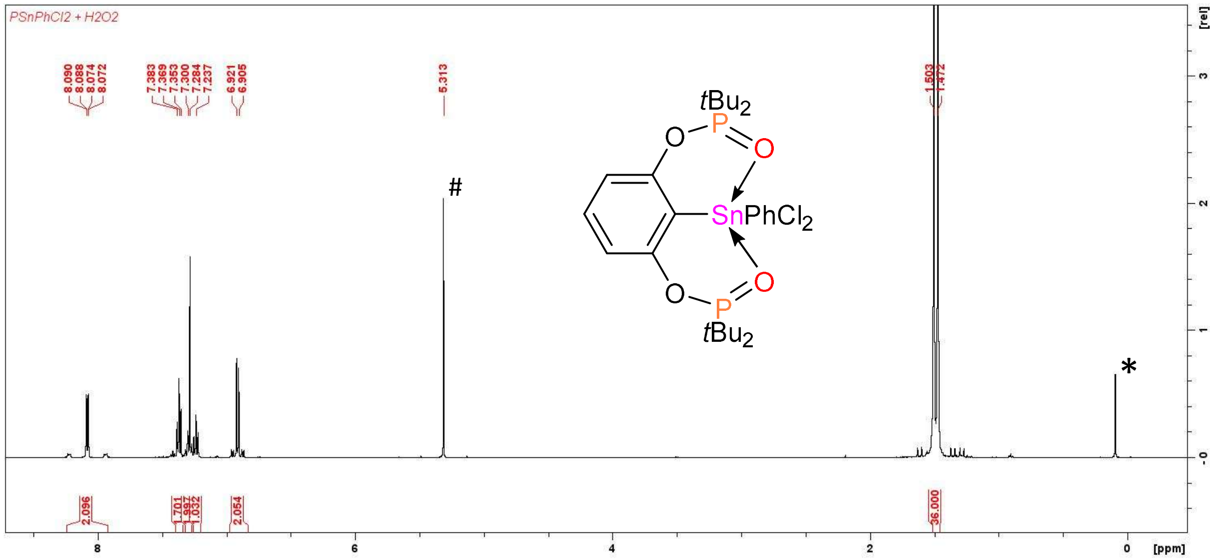

Figure S40. ¹H NMR spectrum of **2^O^** (500 MHz, $CDCl_3$). *Signal of silicon grease, #signal of co-crystallized dichloromethane.

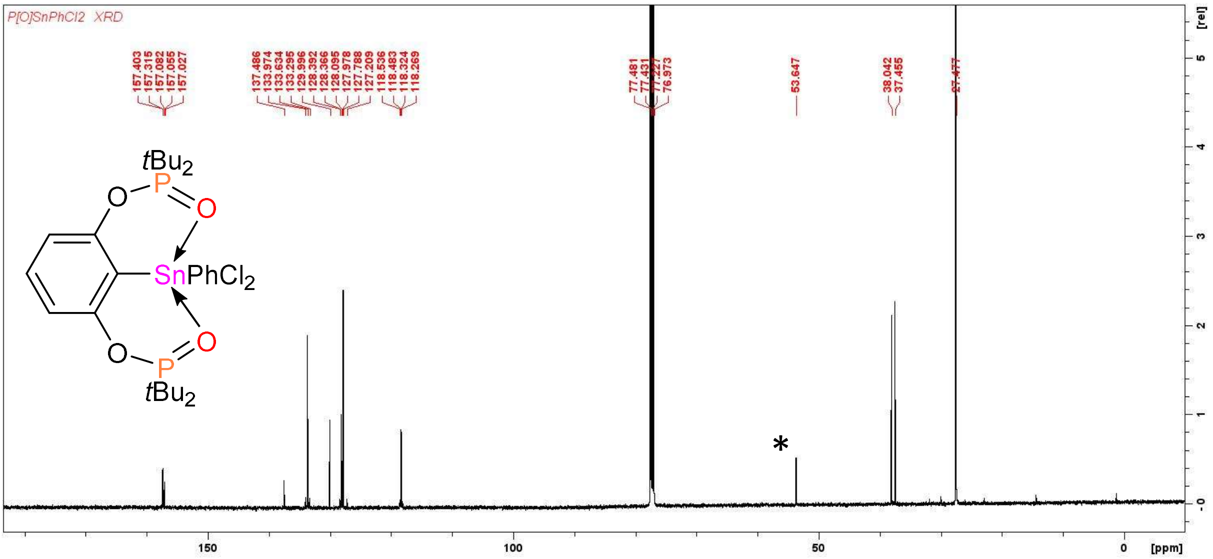

Figure S41. $^{13}C\{^{1}H\}$ NMR spectrum of **2^O^** (125.76 MHz, $CDCl_3$). *Signal of co-crystallized dichloromethane.

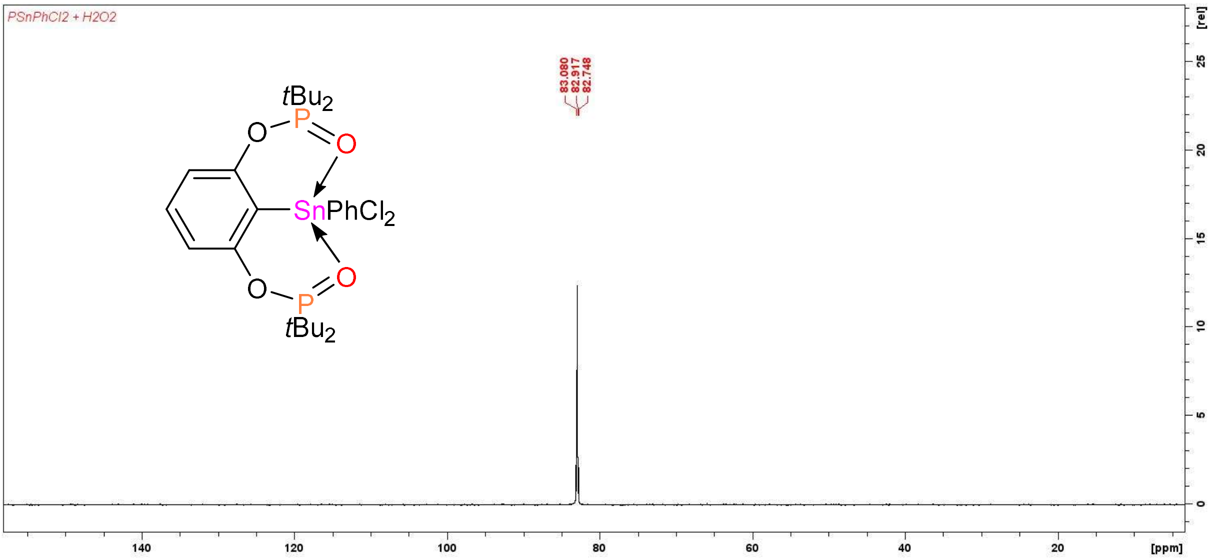

Figure S42. $^{31}P\{^{1}H\}$ NMR spectrum of **2^O^** (202.5 MHz, $CDCl_3$).

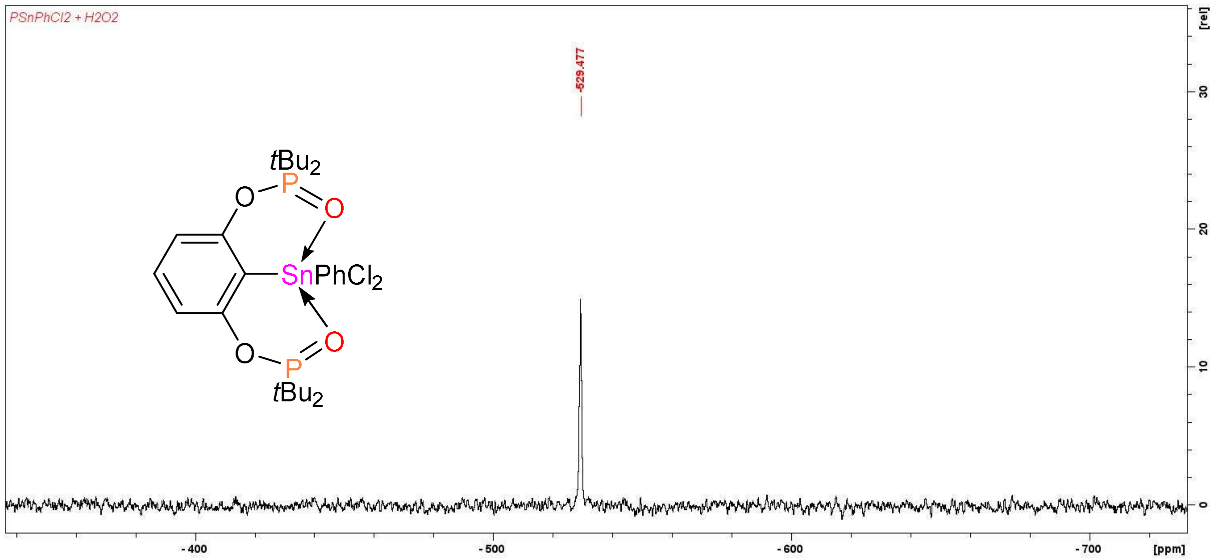

Figure S43. $^{119}Sn\{^1H\}$ NMR spectrum of $\mathbf{2^O}$ (186.5 MHz, $CDCl_3$).

Synthesis of [2,6-(*t*Bu$_2$(S)PO)$_2$C$_6$H$_3$]SnPhCl$_2$ ($\mathbf{2^S}$)

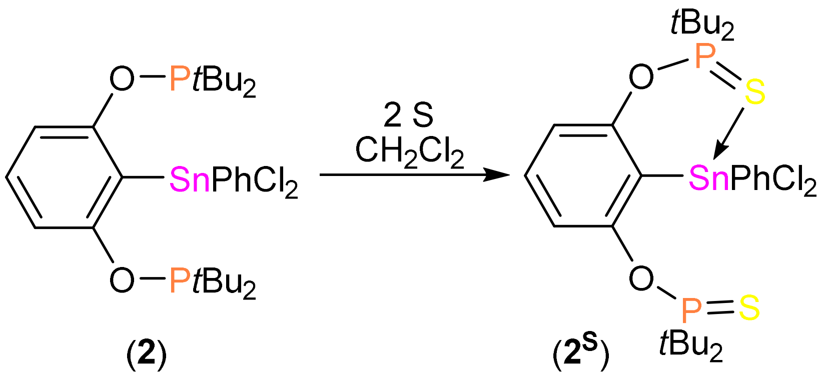

Elemental sulfur (35 mg; 1.09 mmol) was added in one portion to a solution of **2** (366 mg; 0.55 mmol) in dichloromethane (20 ml). The reaction mixture was stirred for 24 h at room temperature and then concentrated to 1/2 of the original volume. Colorless solution was layered with hexane. Crystallization at room temperature gave colorless crystals of compound $\mathbf{2^S}$ and another batch of crystals could be obtained from mother liquor by crystallization at -30 °C. Combined yield of $\mathbf{2^S}$ was 342 mg, (85 %), m. p. 179-182°C. Single-crystals suitable for *sc*-XRD diffraction analysis were obtained by slow diffusion of hexane into saturated dichloromethane solution at room temperature. Anal. Calcd for $C_{28}H_{44}Cl_2O_2P_2S_2Sn$ (MW 728.34): C, 46.2; H, 6.1 %. Found: C, 46.0; H, 6.3 %. ^{1}**H NMR** (500 MHz, C_6D_6) δ (ppm):

1.16 [36H, d, $^3J(^{31}P,^1H)$ = 16.6 Hz, *t*$Bu_2P(S)$-C*H_3*], 6.85 [1H, t, $^3J(^1H,^1H)$ = 8.3 Hz, Ar-*H*], 7.02 [1H, t, $^3J(^1H,^1H)$ = 7.7 Hz, Ar-*H*], 7.08 [2H, t, $^3J(^1H,^1H)$ = 7.6 Hz, Ar-*H*], 7.53 [2H, d(br), $^3J(^1H,^1H)$ = 6.9 Hz, Ar-*H*], 8.21 [4H, d, $^3J(^1H,^1H)$ = 7.2 Hz, $^2J(^{119/117}Sn, ^1H)$ = 102.5 Hz, Ar-*H*]. **$^{13}C\{^1H\}$ NMR** (125.78 MHz, C_6D_6) δ (ppm): 28.1 [s, *t*$Bu_2P(S)$-*C*H_3], 42.4 [d, $^1J(^{31}P,^{13}C)$ = 53.0 Hz, *t*$Bu_2P(S)$-*C*], 119.8 [d, $^3J(^{31}P,^{13}C)$ = 4.5 Hz, $^3J(^{119/117}Sn, ^{13}C)$ = 41.9 Hz, Ar-*C*], 129.4 [s, $^3J(^{119/117}Sn,^{13}C)$ = 106.2 Hz, Ar-*C*], 130.7 [s, $^4J(^{119/117}Sn, ^{13}C)$ = 21.2 Hz, Ar-*C*], 131.8 [s, Ar-*C*], 136.2 [s, $^2J(^{119/117}Sn,^{13}C)$ = 68.4 Hz, Ar-*C*], 158.4 [d, $^2J(^{31}P,^{13}C)$ = 11.5 Hz, Ar-*C*]. **$^{31}P\{^1H\}$ NMR** (202.5 MHz, C_6D_6) δ (ppm): 132.9 [s]. **$^{119}Sn\{^1H\}$ NMR** (186.5 MHz, C_6D_6) δ (ppm): -217.3 [s].

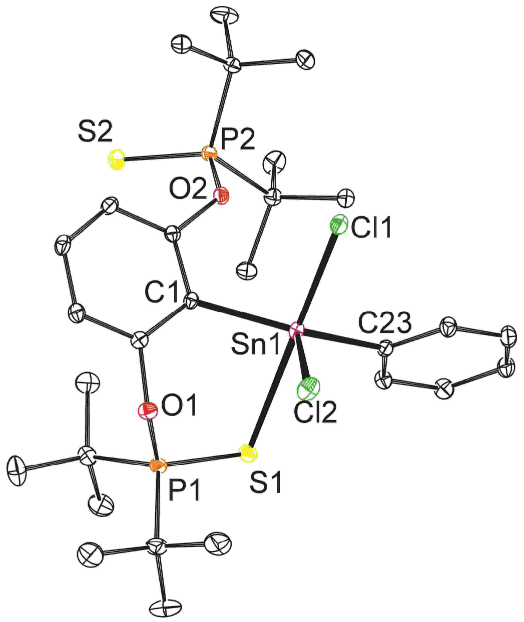

Figure S44. Molecular structure of **2**^{S}. ORTEP with 30% probability ellipsoid level. Only one of two independent molecules in the unit cell is presented. Hydrogen atoms are omitted. Selected structural bond lengths [Å]: Sn(1)-C(1) 2.1364(13), Sn(1)-Cl(1) 2.4367(7), Sn(1)-Cl(2) 2.3462(7), Sn(1)-C(23) 2.1372(14), Sn(1)-S(1) 2.8933(7), Sn(1)-S(2) 5.8217(6); bonding angles [°]: Cl(1)-Sn(1)-S(1) 176.31(2), C(1)-Sn(1)-Cl(2) 104.42(4), C(1)-Sn(1)-C(23) 143.83(6), Cl(2)-Sn(1)-C(23) 109.51(4). Selected structural bond lengths for the second independent molecule [Å]: Sn(2)-C(101) 2.1465(13), Sn(2)-Cl(3) 2.4457(6), Sn(2)-Cl(4) 2.3524(7), Sn(2)-C(123) 2.1401(15), Sn(2)-S(3) 2.8444(6), Sn(2)-S(4) 5.8810(6); bonding

angles [°]: Cl(3)-Sn(2)-S(3) 177.07(3), C(101)-Sn(2)-Cl(4) 110.39(4), C(101)-Sn(2)-C(123) 140.36(6), Cl(4)-Sn(2)-C(123) 1107.85(4).

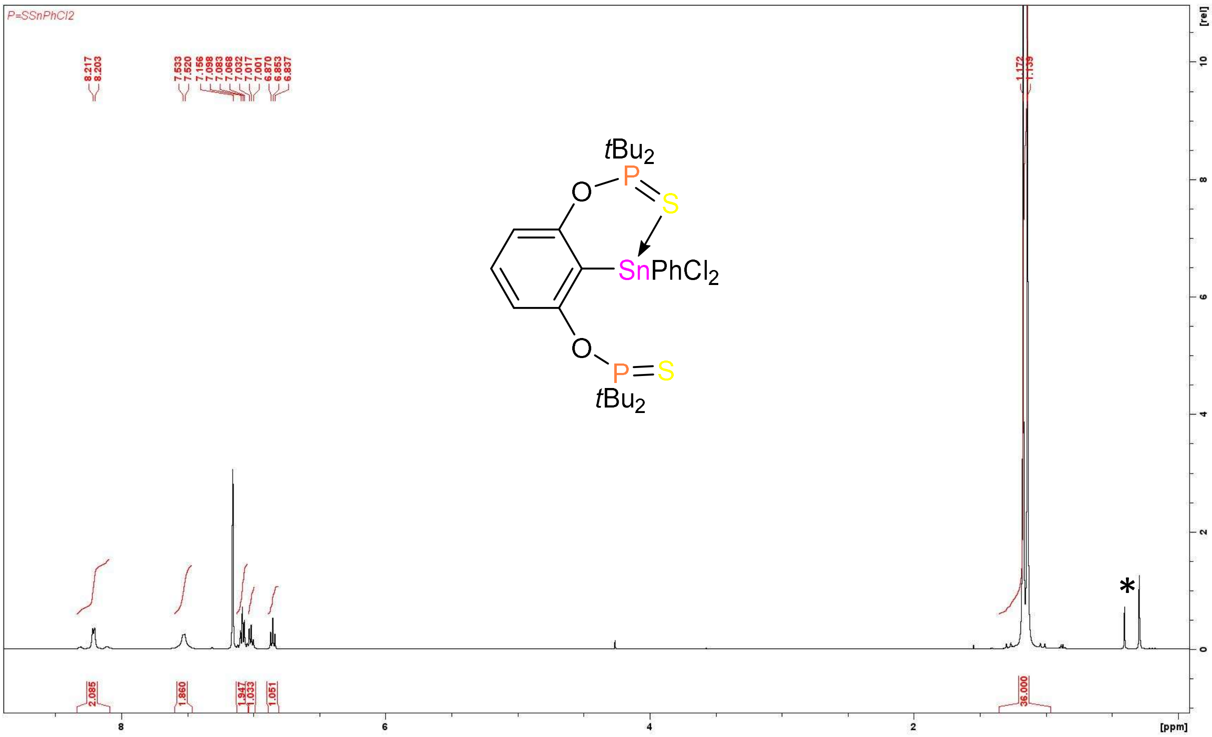

Figure S45. ^{1}H NMR spectrum of **2**^{S} (500 MHz, C_6D_6). *Signal of residual moisture in C_6D_6 and silicon grease.

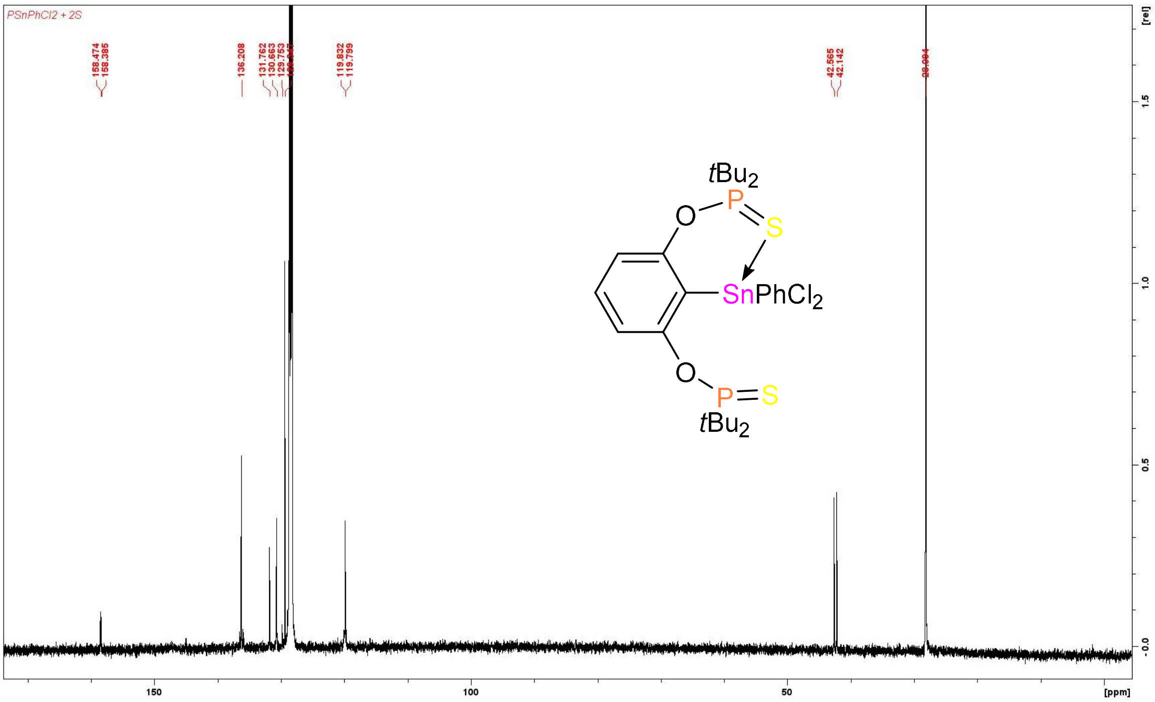

Figure S46. $^{13}C\{^{1}H\}$ NMR spectrum of **2**^{S} (125.76 MHz, C_6D_6).

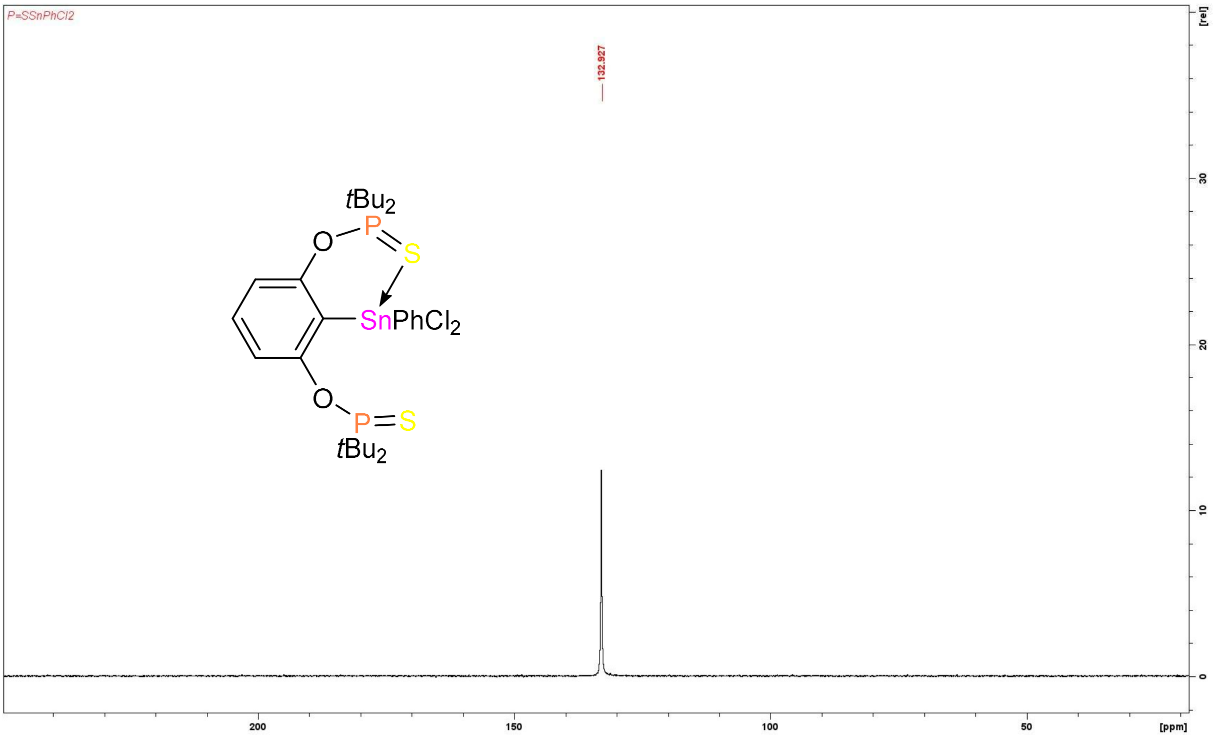

Figure S47. $^{31}P\{^1H\}$ NMR spectrum of **2**^{S} (202.5 MHz, C_6D_6).

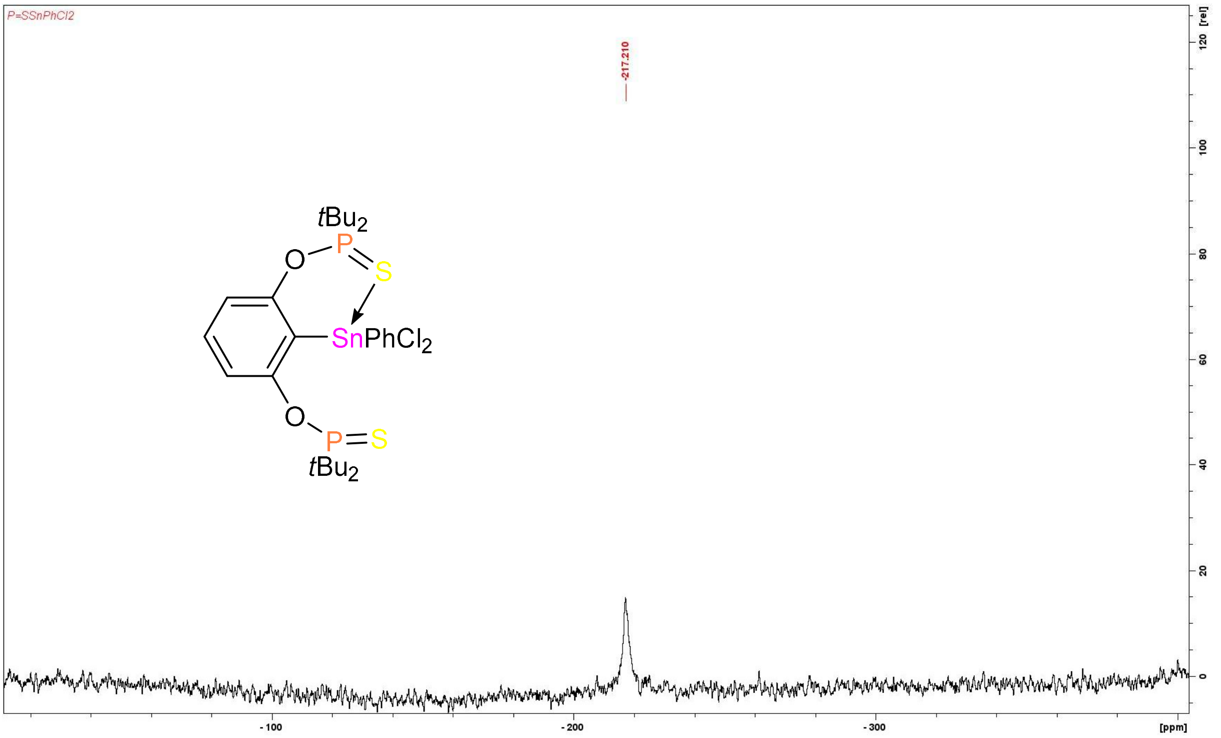

Figure S48. $^{119}Sn\{^1H\}$ NMR spectrum of **2**^{S} (186.5 MHz, C_6D_6).

Synthesis of [2,6-(tBu$_2$(Se)PO)$_2$C$_6$H$_3$]SnPhCl$_2$ (2^{Se})

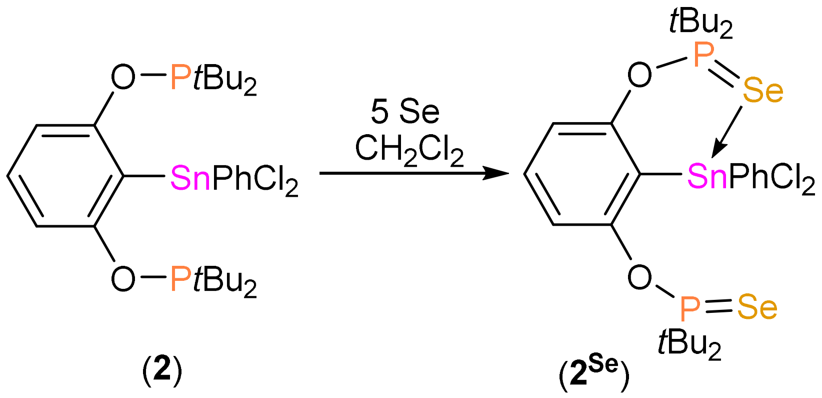

Elemental selenium (275 mg; 3.48 mmol) was added in one portion to solution of **2** (462 mg; 0.70 mmol) in dichloromethane (20 ml). The reaction mixture was stirred for 1 h at room temperature and then unreacted selenium was removed by filtration. Resulting colorless solution was concentrated to 1/2 of the original volume and was layered with hexane. Crystallization at room temperature gave colorless crystals of compound **2^{Se}**, and another batch of crystals could be gained from mother liquor by crystallization at -30 °C. Combined yield of **2^{Se}** was 482 mg, (84 %), m. p. 197-199°C. Single-crystals suitable for *sc*-XRD diffraction analysis were obtained from saturated solution using dichloromethane/hexane mixture at 5 °C. Anal. Calcd for $C_{28}H_{44}Cl_2O_2P_2Se_2Sn$ (MW 822.16): C, 40.2; H, 5.4 %. Found: C, 40.1; H, 5.7 %. **^{1}H NMR** (500 MHz, CDCl$_3$) δ (ppm): 1.35 [36H, d, $^{3}J(^{31}P,^{1}H)$ = 17.2 Hz, tBu$_2$(Se)P-CH_3], 7.41 [4H, m, Ar-H], 7.63 [2H, d(br), $^{3}J(^{1}H,^{1}H)$ = 8.1 Hz, Ar-H], 8.10 [2H, d, $^{3}J(^{1}H,^{1}H)$ = 7.2 Hz, $^{3}J(^{119/117}Sn, ^{1}H)$ = 109.0 Hz, Ar-H]. **^{13}C{^{1}H} NMR** (125.78 MHz, CDCl$_3$) δ (ppm): 28.1 [s, tBu$_2$(Se)P-CH$_3$], 43.1 [d, $^{1}J(^{31}P,^{13}C)$ = 40.6 Hz, tBu$_2$(Se)P-C], 119.7 [d, $^{3}J(^{31}P,^{13}C)$ = 3.7 Hz, $^{3}J(^{119/117}Sn,^{13}C)$ = 41.8 Hz, Ar-C], 127.6 [s(br), Ar-C], 128.9 [s, $^{3}J(^{119/117}Sn,^{13}C)$ = 110.8 Hz, Ar-C], 130.3 [s, $^{4}J(^{119/117}Sn,^{13}C)$ = 22.6 Hz, Ar-C], 130.8 [s, Ar-C], 135.2 [s, $^{2}J(^{119/117}Sn,^{13}C)$ = 71.1 Hz, Ar-C], 147.8 [s(br), Ar-C], 157.2 [d, $^{2}J(^{31}P,^{13}C)$ = 11.6 Hz, Ar-C]. **^{31}P{^{1}H} NMR** (202.5 MHz, CDCl$_3$) δ (ppm): 141.9 [s, $^{1}J(^{77}Se,^{31}P)$ = 733 Hz]. **^{77}Se{^{1}H} NMR** (95.4 MHz,

CDCl3) δ (ppm): -222.2 [d, $^{1}J(^{77}Se,^{31}P)$ = 733 Hz]. **^{119}Sn{^{1}H} NMR** (186.5 MHz, CDCl3) δ (ppm): -264.3 [s(br)].

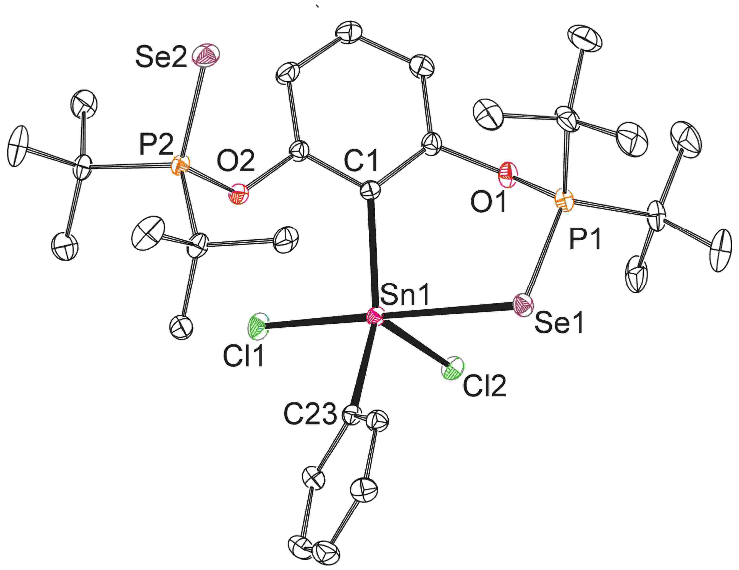

Figure S49. Molecular structure of **2**^{Se}. ORTEP with 30% probability ellipsoid level. Hydrogen atoms are omitted. Selected structural bond lengths [Å]: Sn(1)-C(1) 2.147(2), Sn(1)-Cl(1) 2.4417(7), Sn(1)-Cl(2) 2.3615(6), Sn(1)-C(23) 2.135(2), Sn(1)-Se(1) 2.9331(5), Sn(1)-Se(2) 6.0469(5); bonding angles [°]: Se(1)-Sn(1)-Cl(1) 176.28(2), C(1)-Sn(1)-Cl(2) 113.53(6), C(1)-Sn(1)-C(23) 140.44(9), C(1)-Sn(1)-C(23) 140.44(9).

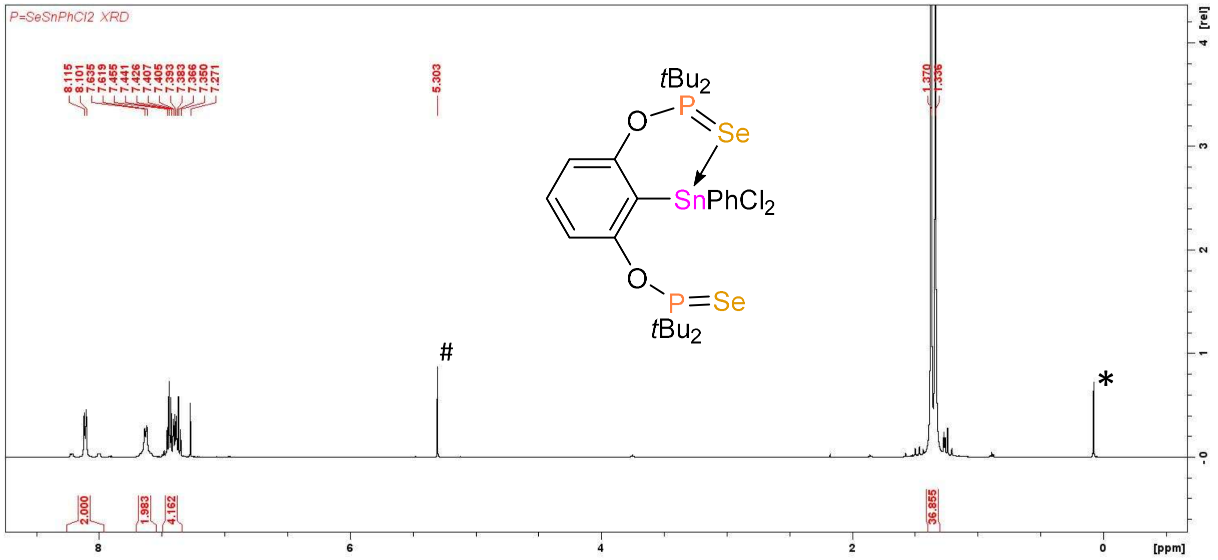

Figure S50. ^{1}H NMR spectrum of **2**^{Se} (500 MHz, CDCl3). *Signal of silicon grease, #signal of co-crystallized dichloromethane.

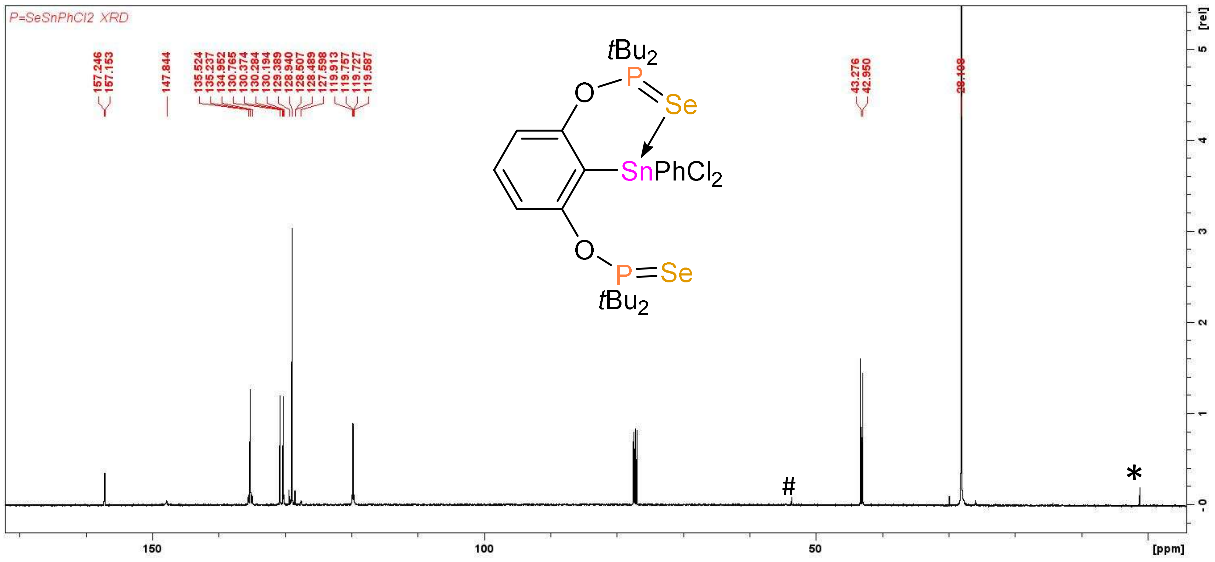

Figure S51. $^{13}C\{^{1}H\}$ NMR spectrum of **2**$^{\mathbf{Se}}$ (125.76 MHz, $CDCl_3$). *signal of silicon grease. #signal of co-crystallized dichloromethane.

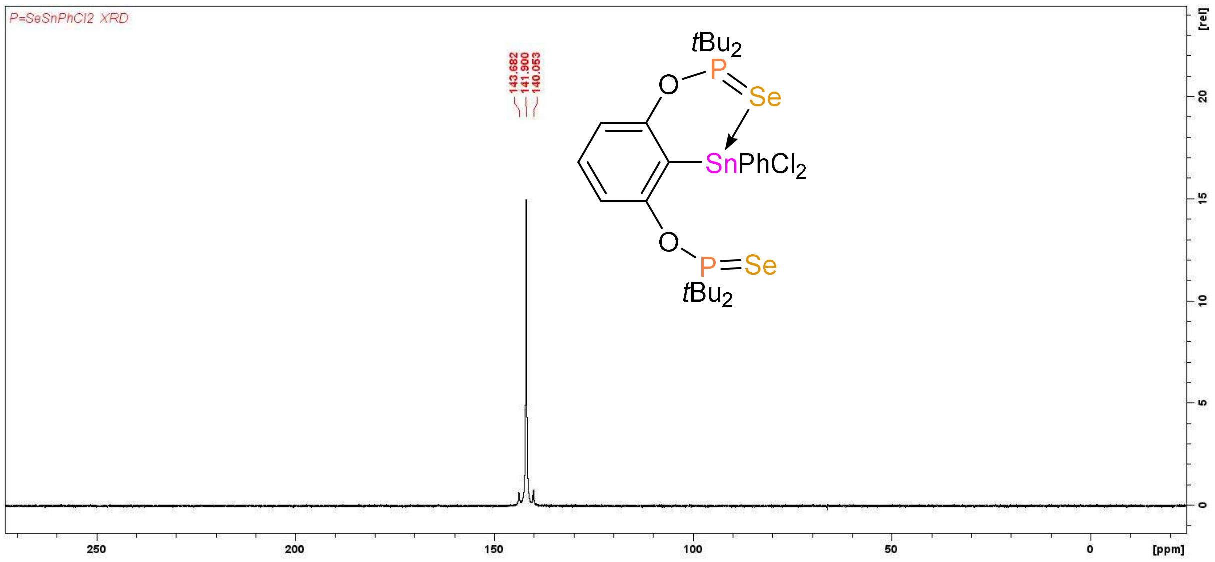

Figure S52. $^{31}P\{^{1}H\}$ NMR spectrum of **2**$^{\mathbf{Se}}$ (202.5 MHz, $CDCl_3$).

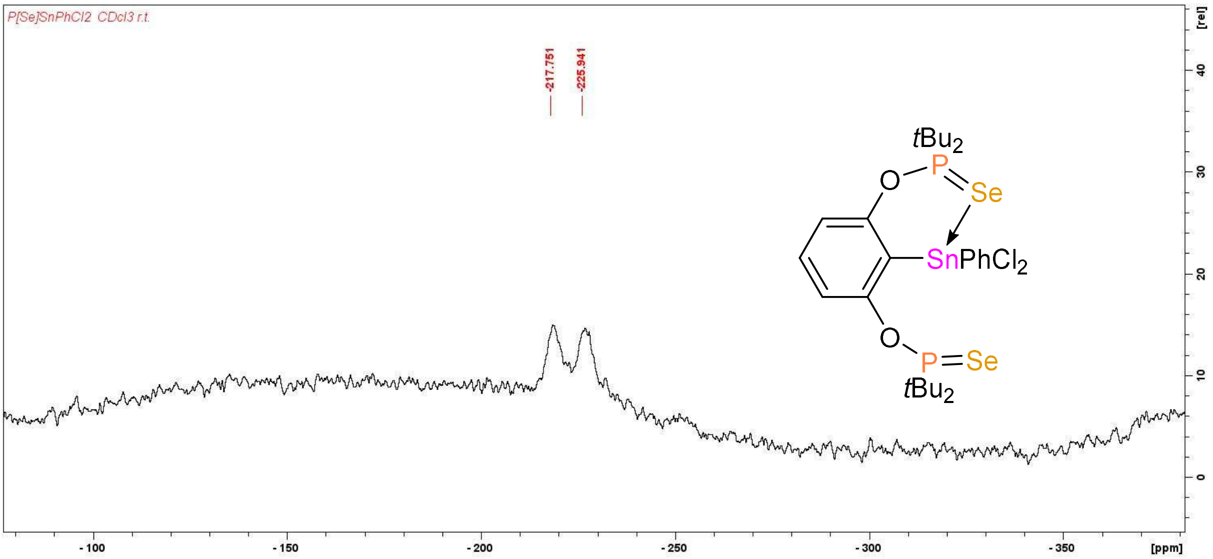

Figure S53. ^{77}Se{^{1}H} NMR spectrum of **1[BArF]** (95.4 MHz, $CDCl_3$). Measured at 323K.

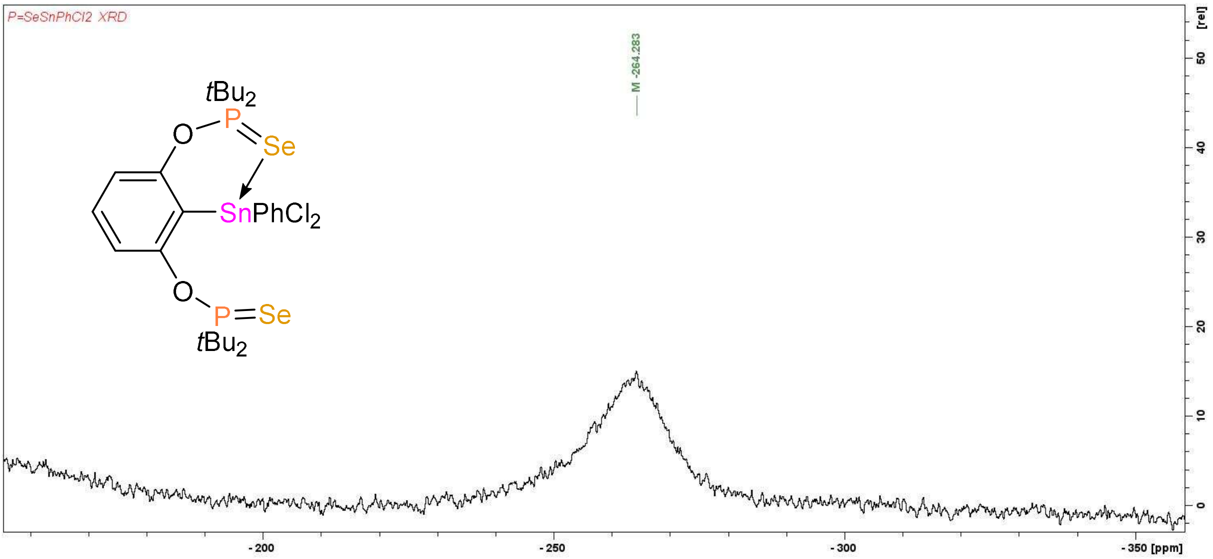

Figure S54. ^{119}Sn{^{1}H} NMR spectrum of $\mathbf{2^{Se}}$ (186.5MHz, $CDCl_3$).

Synthesis of {[2,6-(tBu$_2$(O)PO)$_2$C$_6$H$_3$]SnPhCl}{[B[3,5-(CF$_3$)$_2$C$_6$H$_3$]$_4$} (2^{O+}[BArF]$^-$)

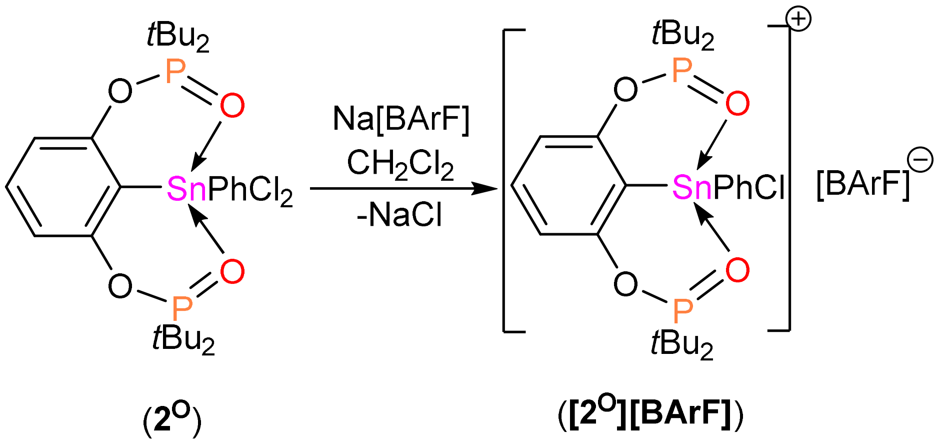

Solid Na[BArF] (183 mg; 0.21 mmol) was added in one portion to solution of $\mathbf{2^O}$ (144 mg; 0.21 mmol) in dichloromethane (10 ml). The reaction mixture was stirred for 30 min at room temperature and then incipient NaCl was removed by filtration. Solvent was removed in vacuo and resulting powder was washed with hexane (10 ml). Colorless powder of compound $\mathbf{2^{O+}[BArF]^-}$ was obtained. Yield of $\mathbf{2^{O+}[BArF]^-}$ was 287 mg, (91 %), m. p. 204-207 °C. Single-crystals suitable for *sc*-XRD diffraction analysis were obtained from saturated dichloromethane solution at -30 °C. Anal. Calcd for $C_{60}H_{56}BClF_{24}O_4P_2Sn$ (MW 1523.98): C, 47.3; H, 3.7 %. Found: C, 47.8; H, 4.3 %. **^{1}H NMR** (500 MHz, CDCl$_3$) δ (ppm): 1.19 [18H, d, $^3J(^{31}P,^1H)$ = 16.3 Hz, tBu$_2$P(O)-CH_3], 1.37 [18H, d, $^3J(^{31}P,^1H)$ = 16.6 Hz, tBu$_2$P(O)-CH_3], 7.06 [2H, d, $^3J(^1H,^1H)$ = 8.3 Hz, $^4J(^{119/117}Sn,^1H)$ = 32.1 Hz, Ar-*H*], 7.45 [1H, t, $^3J(^1H,^1H)$ = 8.3 Hz, Ar-*H*], 7.53 [7H, m, Ar-*H*], 7.72 [8H, s, Ar-*H*], 7.77 [2H, m, $^3J(^{119/117}Sn, ^1H)$ = 108.2 Hz, Ar-*H*]. **^{11}B{^{1}H} NMR** (160.42 MHz, CDCl$_3$) δ (ppm): -6.6 [s]. **^{13}C{^{1}H} NMR** (125.78 MHz, CDCl$_3$) δ (ppm): 26.0 [s, tBu$_2$P(O)-*C*H$_3$], 26.4 [s, tBu$_2$P(O)-*C*H$_3$], 37.8 [d, $^1J(^{31}P,^{13}C)$ = 70.3 Hz tBu$_2$P(O)-*C*], 117.7 [m, Ar-*C*], 119.5 [d, $^3J(^{31}P,^{13}C)$ = 6.5 Hz, $^3J(^{119/117}Sn,^{13}C)$ = 38.5 Hz, Ar-*C*], 120.2 [s, Ar-*C*], 124.8 [q, $^1J(^{19}F,^{13}C)$ = 273 Hz, *C*F$_3$], 129.2 [qq, $^2J(^{19}F,^{13}C)$ = 32.0 Hz, $^4J(^{19}F,^{13}C)$ = 3.0 Hz, Ar-*C*], 130.0 [s, $^3J(^{119/117}Sn,^{13}C)$ = 116.0 Hz, Ar-*C*], 132.2 [s, $^4J(^{119/117}Sn,^{13}C)$ = 22.7 Hz, Ar-*C*], 134.9 [s, $^2J(^{119/117}Sn, ^{13}C)$ = 69.0 Hz, Ar-C], 135.0 [s, Ar-*C*], 135.5 [s, Ar-*C*], 139.6 [s, Ar-*C*]; 158.8 [d, $^2J(^{31}P,^{13}C)$ = 11.1 Hz, Ar-C]; 161.9 [q, $^1J(^{13}C,^{11}B)$ =

49.8 Hz, Ar-*C*]. **^{19}F{^{1}H} NMR** (376.3 MHz, $CDCl_3$) δ (ppm): -62.8 [s]. **^{31}P{^{1}H} NMR** (202.5 MHz, $CDCl_3$) δ (ppm): 87.4 [s, ^{n}J($^{119/117}$Sn, ^{31}P) = 54.5 Hz]. **^{119}Sn{^{1}H} NMR** (186.5 MHz, $CDCl_3$) δ (ppm): -345.0 [t, ^{n}J($^{119/117}$Sn, ^{31}P) = 54.5 Hz].

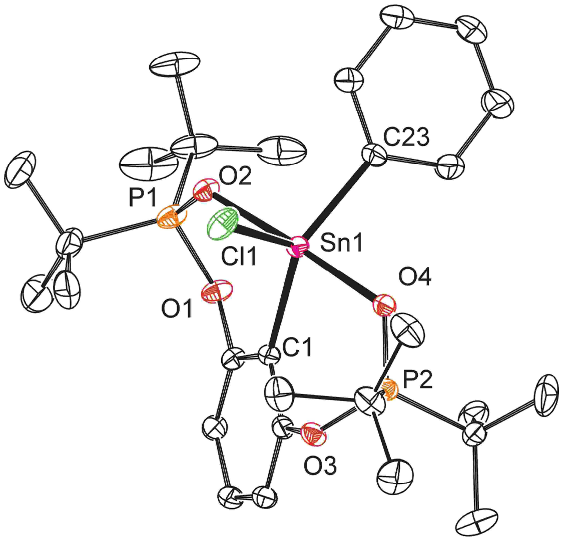

Figure S55. Molecular structure of **2^{O+}[BArF]$^{-}$**. ORTEP with 30% probability ellipsoid level. Hydrogen atoms and [BArF] anion are omitted. Selected structural bond lengths [Å]: Sn(1)-C(1) 2.142(2), Sn(1)-Cl(1) 2.3071(8), Sn(1)-C(23) 2.102(3), Sn(1)-O(2) 2.1601(17), Sn(1)-O(4) 2.1537(17); bonding angles [°]: O(2)-Sn(1)-O(4) 176.96(7), C(1)-Sn(1)-Cl(1) 111.75(7), C(1)-Sn(1)-C(23) 134.64(10), Cl(1)-Sn(1)-C(23) 113.54(8).

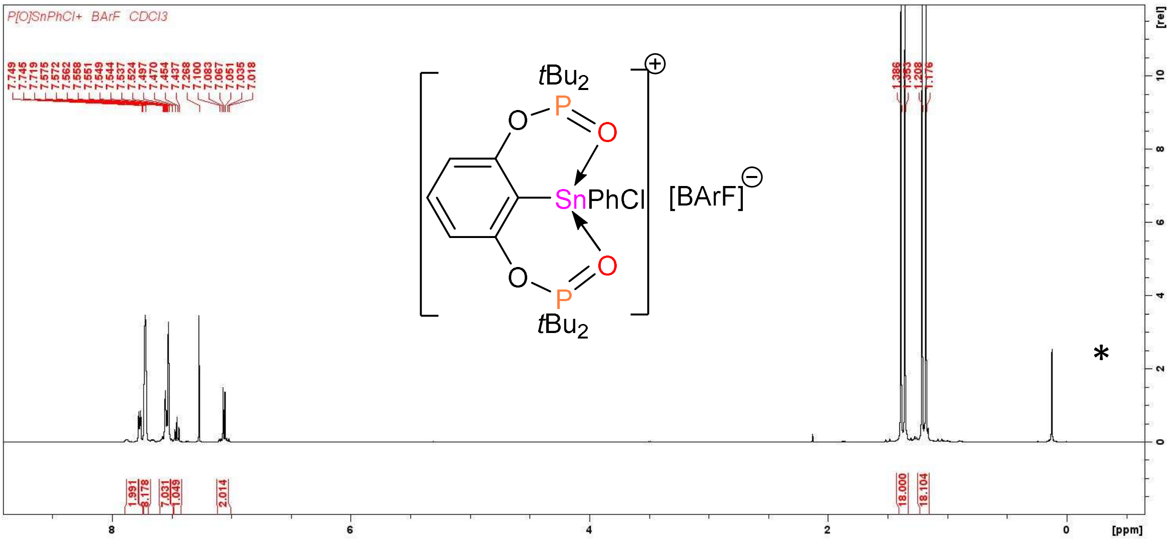

Figure S56. ^{1}H NMR spectrum of **2^{O+}[BArF]$^{-}$** (500 MHz, $CDCl_3$). *Signal of silicon grease.

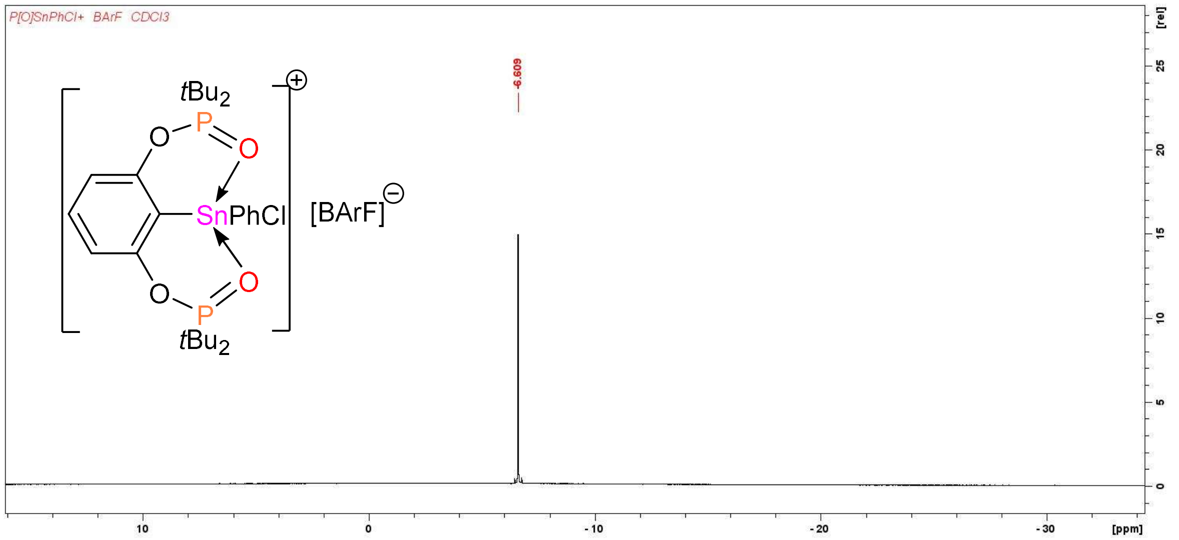

Figure S57. $^{11}B\{^{1}H\}$ NMR spectrum of **2^{O+}[BArF]$^{-}$** (160.42 MHz MHz, $CDCl_3$).

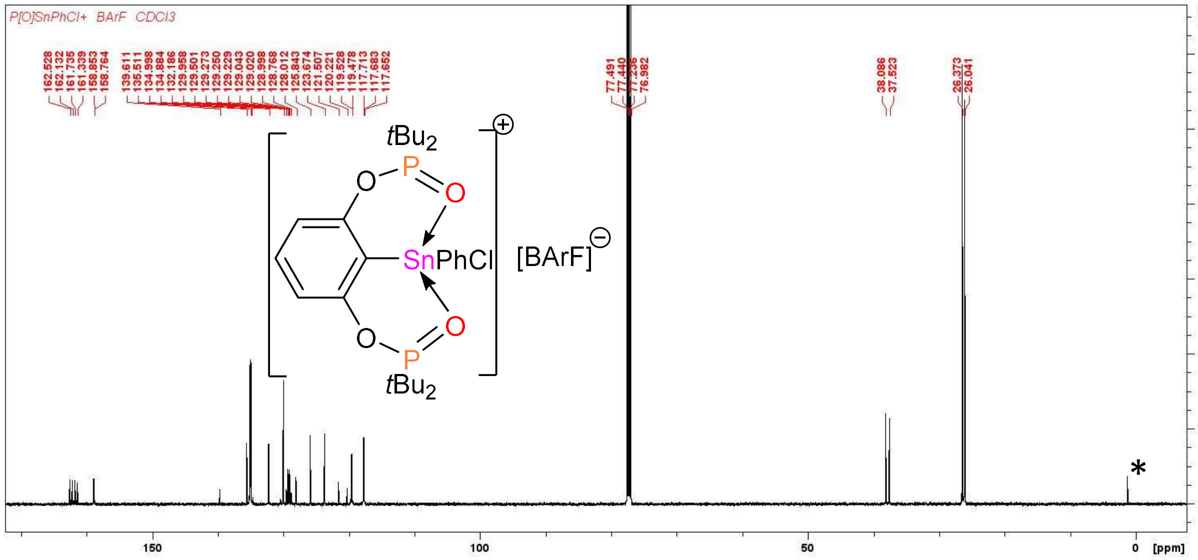

Figure S58. $^{13}C\{^{1}H\}$ NMR spectrum of **2^{O+}[BArF]$^{-}$** (125.76 MHz, $CDCl_3$). *signal of silicon grease.

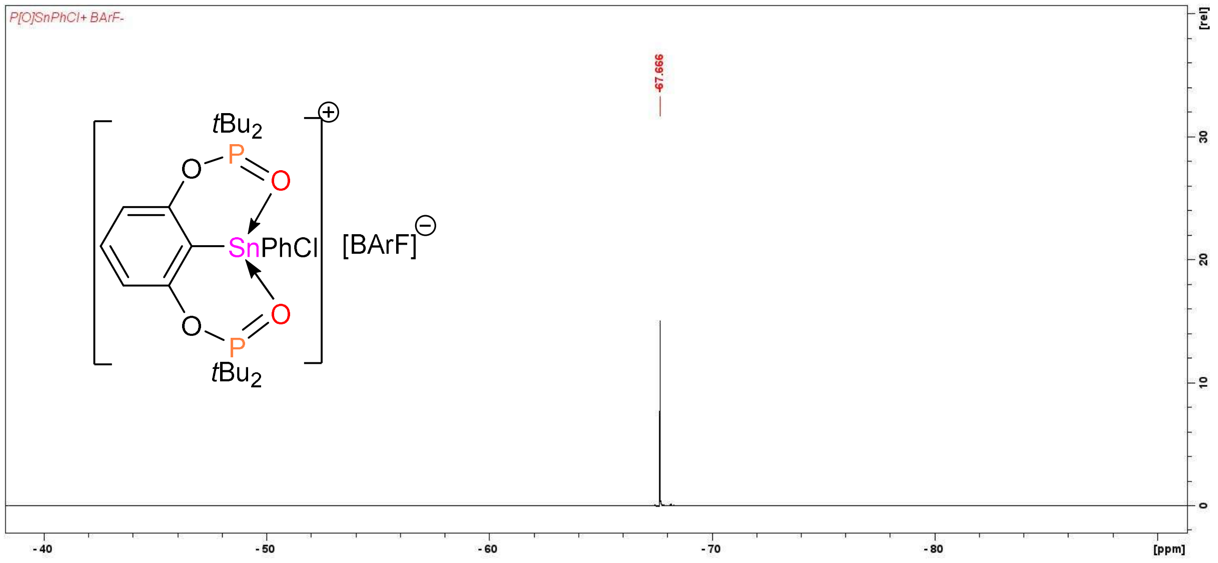

Figure S59. $^{19}F\{^1H\}$ NMR spectrum of $\mathbf{2^{O+}[BArF]^-}$ (470.5 MHz, $CDCl_3$).

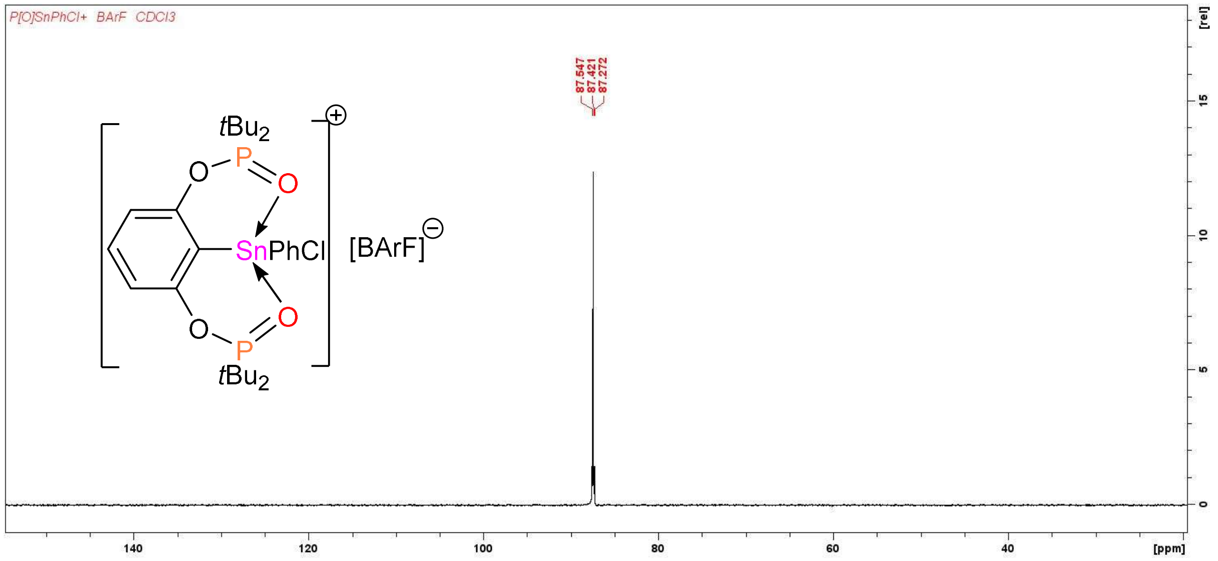

Figure S60. $^{31}P\{^1H\}$ NMR spectrum of $\mathbf{2^{O+}[BArF]^-}$ (202.5 MHz, $CDCl_3$).

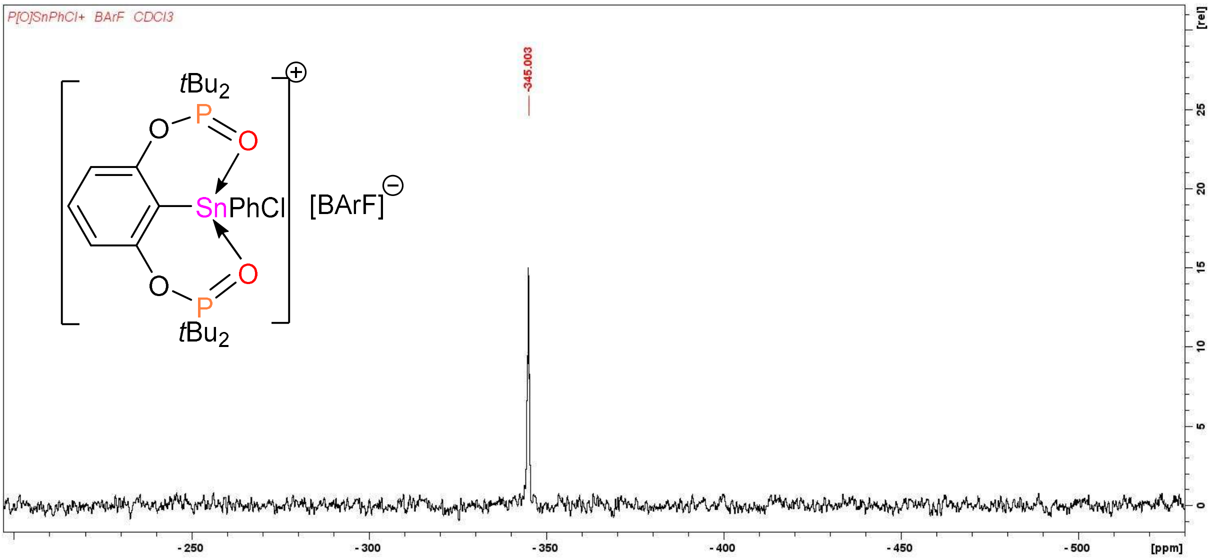

Figure S61. $^{119}Sn\{^1H\}$ NMR spectrum of $\mathbf{2^{O+}[BArF]^-}$ (186.5 MHz, $CDCl_3$).

Synthesis of {[2,6-(tBu$_2$(S)PO)$_2$C$_6$H$_3$]SnPhCl}{[B[3,5-(CF$_3$)$_2$C$_6$H$_3$]$_4$} ($\mathbf{2^{S+}[BArF]^-}$)

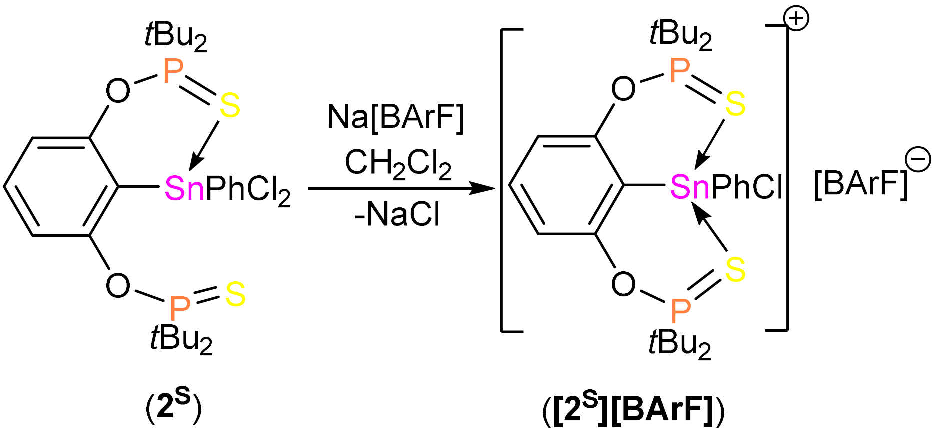

Solid Na[BArF] (136 mg; 0.15 mmol) was added in one portion to solution of $\mathbf{2^S}$ (112 mg; 0.15 mmol) in dichloromethane (10 ml). The reaction mixture was stirred for 30 min at room temperature and then incipient NaCl was removed by filtration. Colorless solution was concentrated to 1/2 of the original volume and layered with hexane. Crystallization at room temperature gave colorless crystals of compound $\mathbf{2^{S+}[BArF]^-}$. Yield of $\mathbf{2^{S+}[BArF]^-}$ was 201 mg, (84 %), m. p. 178-181 °C. Single-crystals suitable for *sc*-XRD diffraction analysis were obtained by slow diffusion of hexane into saturated dichloromethane solution at room temperature. Anal. Calcd for $C_{60}H_{56}BClF_{24}O_2P_2S_2Sn$ (MW 1556.11): C, 46.3; H, 3.6 %. Found:

C, 46.2; H, 3.4 %. **^{1}H NMR** (500 MHz, CD_2Cl_2) δ (ppm): 1.24 [18H, d, $^3J(^{31}P,^1H)$ = 17.8 Hz, *t*Bu_2P(S)-C*H_3*], 1.47 [18H, d, $^3J(^{31}P,^1H)$ = 17.9 Hz, *t*Bu_2P(S)-C*H_3*], 7.35 [2H, d, $^3J(^1H,^1H)$ = 8.5 Hz, $^4J(^{119/117}Sn,^1H)$ = 33.5 Hz, Ar-*H*], 7.56 [3H, m, Ar-*H*], 7.60 [4H, s, Ar-*H*], 7.72 [1H, t, $^3J(^1H,^1H)$ = 8.5 Hz, Ar-*H*], 7.76 [8H, s, Ar-*H*], 7.83 [2H, m, $^3J(^{119/117}Sn, ^1H)$ = 102.4 Hz, Ar-*H*]. **^{11}B{^{1}H} NMR** (160.42 MHz, CD_2Cl_2) δ (ppm): -7.2 [s]. **^{13}C{^{1}H} NMR** (125.78 MHz, CD_2Cl_2) δ (ppm): 27.2 [s, *t*Bu_2P(S)-*C*H_3], 27.6 [s, *t*Bu_2P(S)-*C*H_3], 42.9 [d, $^1J(^{31}P, ^{13}C)$ = 43.9 Hz, *t*Bu_2(S)P-*C*], 118.0 [m, Ar-*C*], 122.4 [d, $^3J(^{31}P,^{13}C)$ = 4.0 Hz, $^3J(^{119/117}Sn,^{13}C)$ = 35.3 Hz, Ar-*C*], 125.1 [q, $^1J(^{19}F,^{13}C)$ = 273 Hz, *C*F_3], 129.4 [qq, $^2J(^{19}F,^{13}C)$ = 32.0 Hz, $^4J(^{19}F,^{13}C)$ = 3.0 Hz, Ar-*C*], 130.4 [s, $^3J(^{119/117}Sn,^{13}C)$ = 114.2 Hz, Ar-*C*], 132.0 [s, $^4J(^{119/117}Sn,^{13}C)$ = 22.1 Hz, Ar-*C*], 134.3 [s, $^2J(^{119/117}Sn, ^{13}C)$ = 69.9 Hz, Ar-C], 135.2 [s, Ar-*C*], 135.3 [s, Ar-*C*], 146.6 [s, Ar-*C*]; 157.5 [d, $^2J(^{31}P,^{13}C)$ = 13.4 Hz, Ar-C]; 162.3 [q, $^1J(^{13}C,^{11}B)$ = 49.8 Hz, Ar-*C*]. **^{19}F{^{1}H} NMR** (376.3 MHz, CD_2Cl_2) δ (ppm): -62.8 [s]. **^{31}P{^{1}H} NMR** (202.5 MHz, CD_2Cl_2) δ (ppm): 134.2 [s]. **^{119}Sn{^{1}H} NMR** (186.5 MHz, CD_2Cl_2) δ (ppm): -304.4 [s].

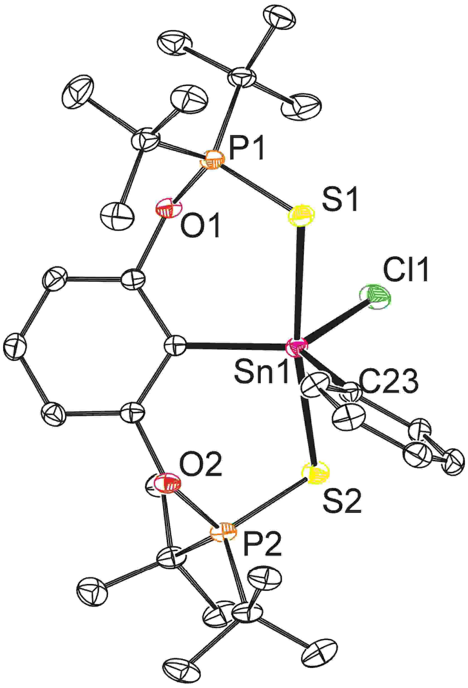

Figure S62. Molecular structure of $\mathbf{2^{S+}[BArF]^{-}}$. ORTEP with 30% probability ellipsoid level. Hydrogen atoms and [BArF] anion are omitted. Selected structural bond lengths [Å]: Sn(1)-C(1) 2.140(2), Sn(1)-Cl(1) 2.3454(6), Sn(1)-C(23) 2.114(2), Sn(1)-S(1) 2.6442(9), Sn(1)-S(2) 2.6383(8); bonding angles [°]: S(1)-Sn(1)-S(2) 170.44(2), C(1)-Sn(1)-Cl(1) 118.72(6), C(1)-Sn(1)-C(23) 123.66(8), Cl(1)-Sn(1)-C(23) 117.62(6).

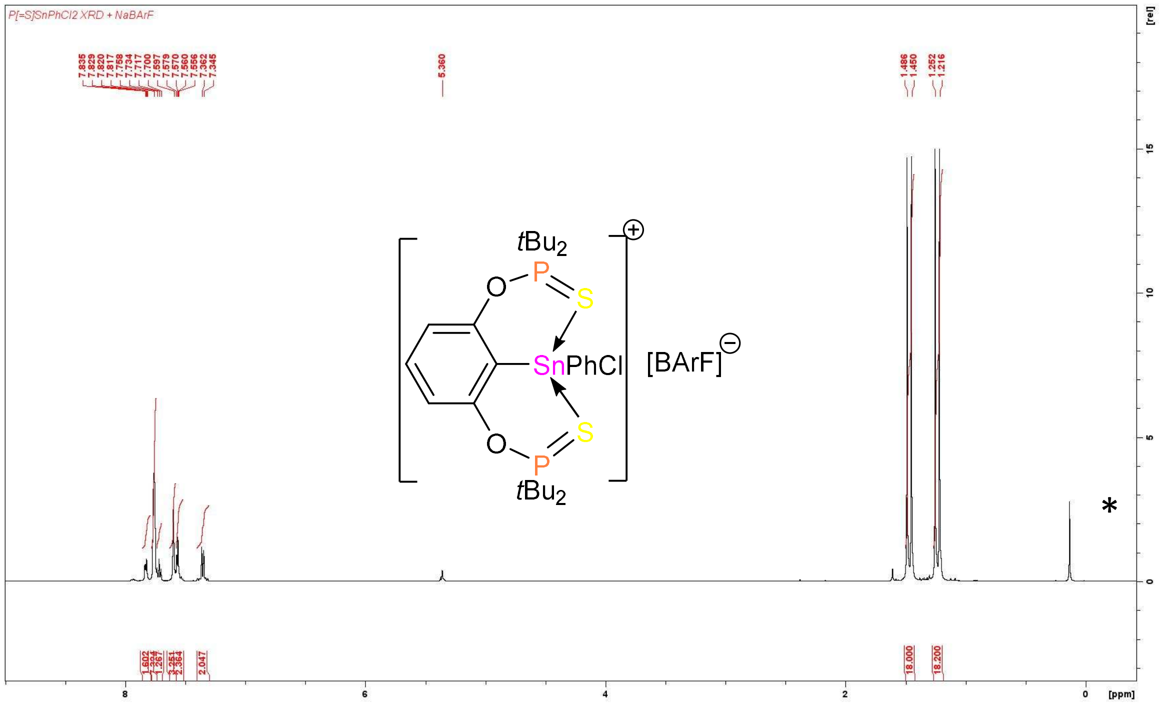

Figure S63. ^{1}H NMR spectrum of $\mathbf{[2^{S}][BArF]}$ (500 MHz, CD_2Cl_2). *Signal of silicon grease.

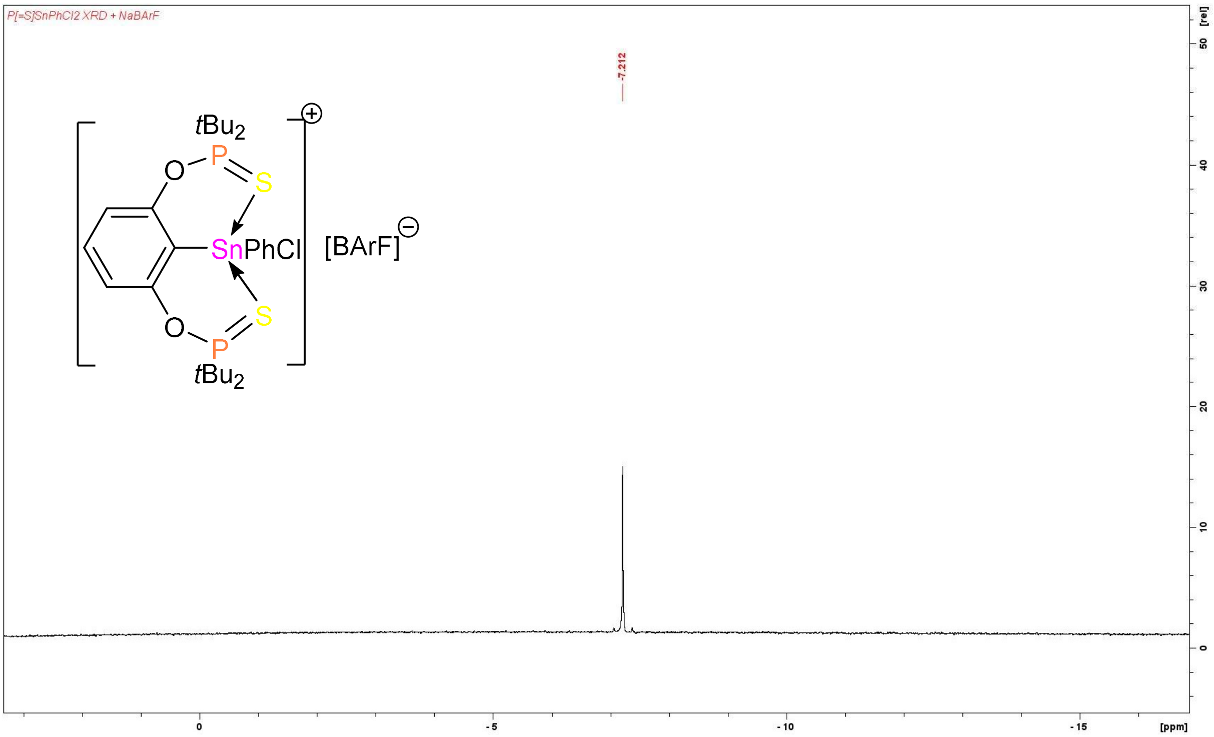

Figure S64. $^{11}B\{^1H\}$ NMR spectrum of **[2^S][BArF]** (160.42 MHz MHz, CD_2Cl_2).

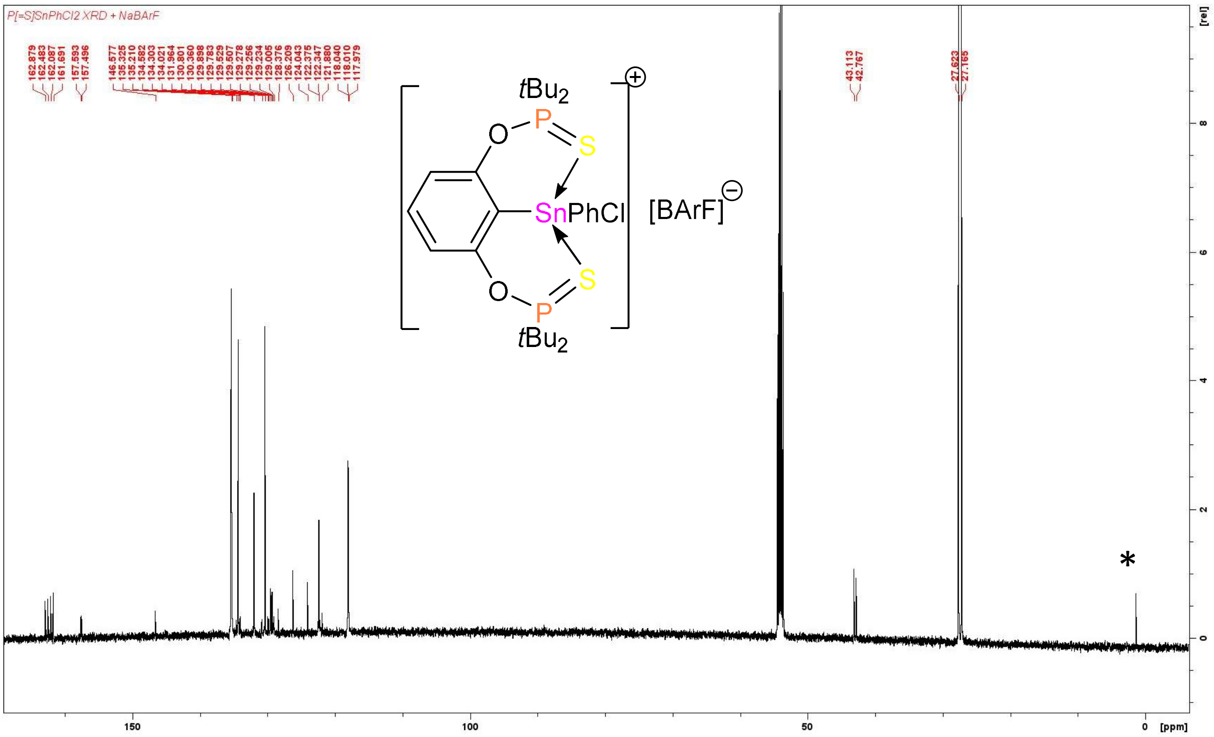

Figure S65. $^{13}C\{^1H\}$ NMR spectrum of **[2^S][BArF]** (125.76 MHz, CD_2Cl_2). *Signal of silicon grease.

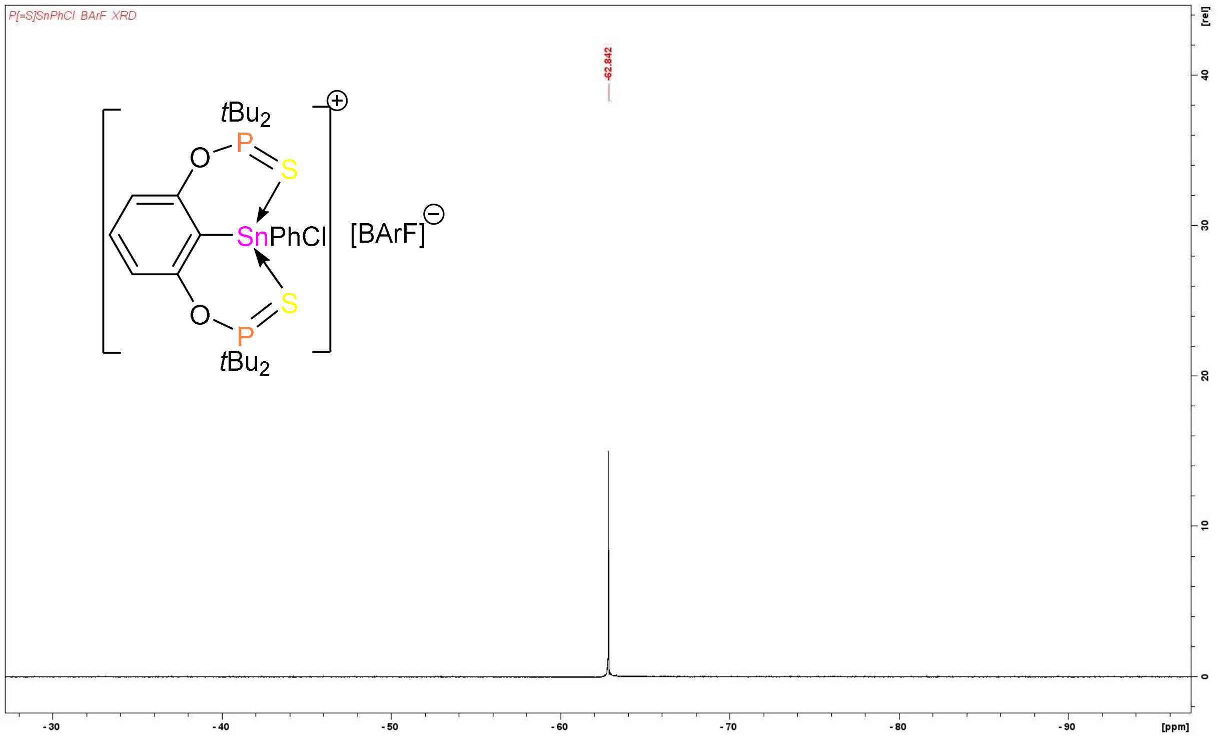

Figure S66. $^{19}F\{^1H\}$ NMR spectrum of **[2^S][BArF]** (470.5 MHz, CD_2Cl_2).

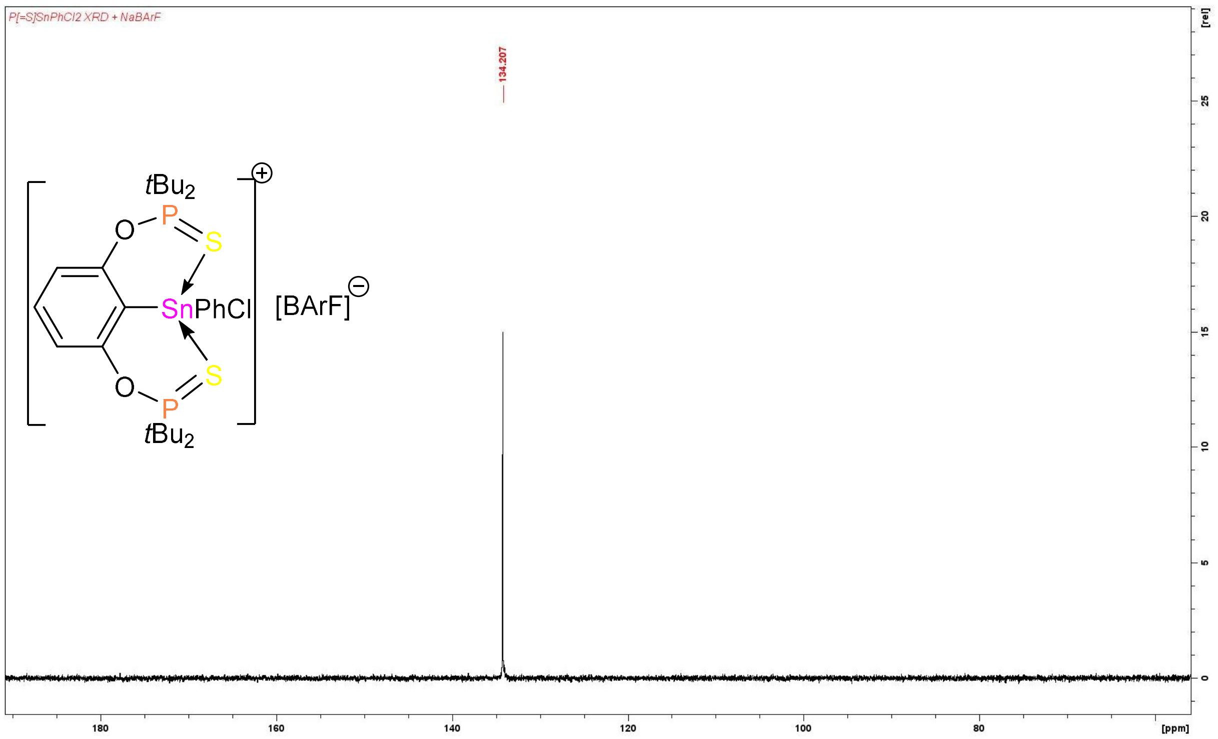

Figure S67. $^{31}P\{^1H\}$ NMR spectrum of **[2^S][BArF]** (202.5 MHz, CD_2Cl_2).

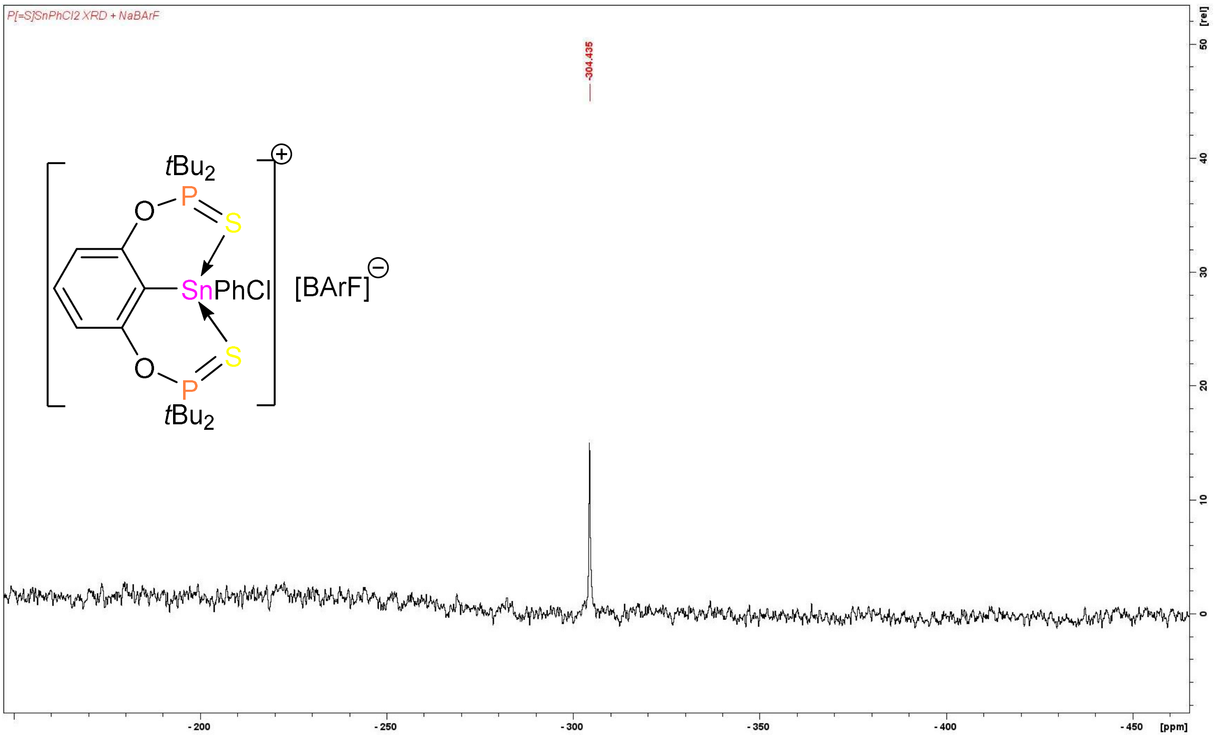

Figure S68. ^{119}Sn{^{1}H} NMR spectrum of **[2^{S}][BArF]** (186.5 MHz, CD_2Cl_2).

Synthesis of {[2,6-(*t*Bu$_2$(Se)PO)$_2$C$_6$H$_3$]SnPhCl}{[B[3,5-(CF$_3$)$_2$C$_6$H$_3$]$_4$} (2^{Se+}[BArF]$^{-}$)

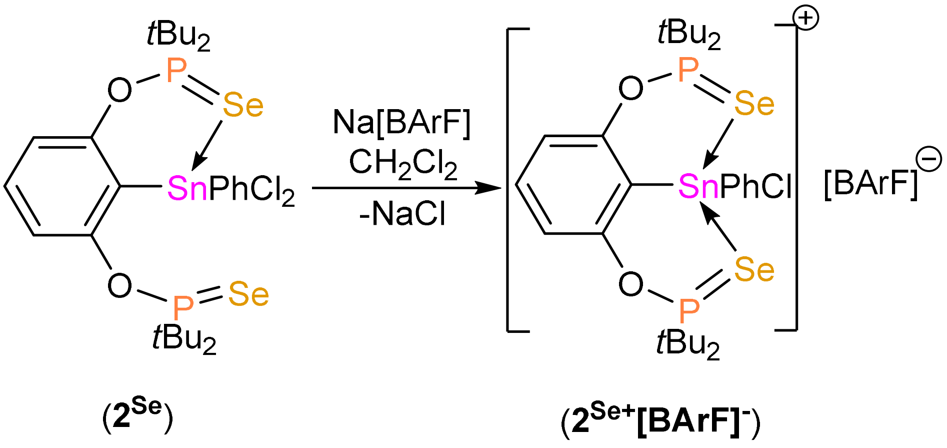

Solid Na[BArF] (410 mg; 0.46 mmol) was added in one portion to solution of **2^{Se}** (380 mg; 0.46 mmol) in dichloromethane (20 ml). The reaction mixture was stirred for 30 min at room temperature and then incipient NaCl was removed by filtration. Colorless solution was concentrated to ¼ of the original volume and layered with hexane. Crystallization at room temperature gave colorless crystals of compound **2^{Se+}[BArF]$^{-}$**, and another batch of crystals

could be obtained from mother liquor by crystallization at -30 °C. Combined yield of $\mathbf{2^{Se+}[BArF]^{-}}$ was 702 mg, (92 %), m. p. 174-176 °C. Single-crystals suitable for *sc*-XRD diffraction analysis were obtained from saturated solution using dichloromethane/hexane mixture at 5 °C. Anal. Calcd for $C_{60}H_{56}BClF_{24}O_2P_2Se_2Sn$ (MW 1649.93): C, 43.7; H, 3.4 %. Found: C, 44.0; H, 3.5 %. **^{1}H NMR** (500 MHz, $CDCl_3$) δ (ppm): 1.18 [18H, d, $^{3}J(^{31}P,^{1}H)$ = 17.6 Hz, *t*Bu$_2$P(Se)-C*H*$_3$], 1.44 [18H, d, $^{3}J(^{31}P,^{1}H)$ = 18.1 Hz, *t*Bu$_2$P(Se)-C*H*$_3$], 7.25 [2H, d, $^{3}J(^{1}H,^{1}H)$ = 8.2 Hz, $^{4}J(^{119/117}Sn,^{1}H)$ = 32.4 Hz, Ar-*H*], 7.48 [3H, m, Ar-*H*], 7.55 [5H, m, Ar-*H*], 7.73 [8H, s, Ar-*H*], 7.79 [2H, m, $^{3}J(^{119/117}Sn, ^{1}H)$ = 111.5 Hz, Ar-*H*]. **^{11}B{^{1}H} NMR** (160.42 MHz, $CDCl_3$) δ (ppm): -7.2 [s]. **^{13}C{^{1}H} NMR** (125.78 MHz, $CDCl_3$) δ (ppm): 27.1 [s, *t*Bu$_2$P(Se)-*C*H$_3$], 27.7 [s, *t*Bu$_2$P(Se)-*C*H$_3$], 43.3 [d, $^{1}J(^{31}P,^{13}C)$ = 33.1 Hz *t*Bu$_2$P(Se)-*C*], 117.7 [m, Ar-*C*], 121.4 [s(br), Ar-*C*], 122.5 [s, $^{3}J(^{119/117}Sn,^{13}C)$ = 34.8 Hz, Ar-*C*], 124.8 [q, $^{1}J(^{19}F,^{13}C)$ = 273 Hz, *C*F$_3$], 129.1 [qq, $^{2}J(^{19}F,^{13}C)$ = 32.0 Hz, $^{4}J(^{19}F,^{13}C)$ = 3.0 Hz, Ar-*C*], 130.0 [s, $^{3}J(^{119/117}Sn,^{13}C)$ = 113.9 Hz, Ar-*C*], 131.5 [s, $^{4}J(^{119/117}Sn,^{13}C)$ = 22.8 Hz, Ar-*C*], 133.7 [s, $^{2}J(^{119/117}Sn, ^{13}C)$ = 70.1 Hz, Ar-C], 134.0 [s, Ar-*C*], 135.0 [s, Ar-*C*], 146.6 [s, Ar-*C*]; 156.9 [d, $^{2}J(^{31}P,^{13}C)$ = 13.7 Hz, Ar-C]; 161.9 [q, $^{1}J(^{13}C,^{11}B)$ = 49.8 Hz, Ar-*C*]. **^{19}F{^{1}H} NMR** (376.3 MHz, $CDCl_3$) δ (ppm): -62.3 [s]. **^{31}P{^{1}H} NMR** (202.5 MHz, $CDCl_3$) δ (ppm): 141.7 [s, $^{1}J(^{77}Se, ^{31}P)$ = 635 Hz]. **^{77}Se{^{1}H} NMR** (95.4 MHz, $CDCl_3$) δ (ppm): -153.8 [d, $^{1}J(^{77}Se,^{31}P)$ = 635 Hz, $^{1}J(^{119/117}Sn, ^{77}Se)$ = 597 Hz,]. **^{119}Sn{^{1}H} NMR** (186.5 MHz, $CDCl_3$) δ (ppm): -322.7 [s(br)].

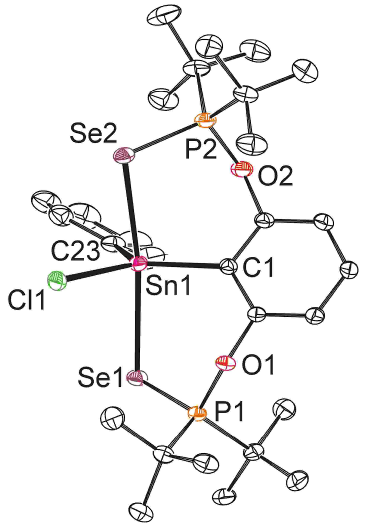

Figure S69. Molecular structure of **2^{Se+}[BArF]$^{-}$**. ORTEP with 30% probability ellipsoid level. Hydrogen atoms, [BArF] anion and dichloromethane solvate molecule are omitted. Selected structural bond lengths [Å]: Sn(1)-C(1) 2.149(4), Sn(1)-Cl(1) 2.3558(13), Sn(1)-C(23) 2.110(6), Sn(1)-Se(1) 2.7694(6), Sn(1)-Se(2) 2.7446(6); bonding angles [°]: Se(1)-Sn(1)-Se(2) 170.31(2), C(1)-Sn(1)-Cl(1) 129.48(12), C(1)-Sn(1)-C(23) 119.6(2), Cl(1)-Sn(1)-C(23) 110.88(18).

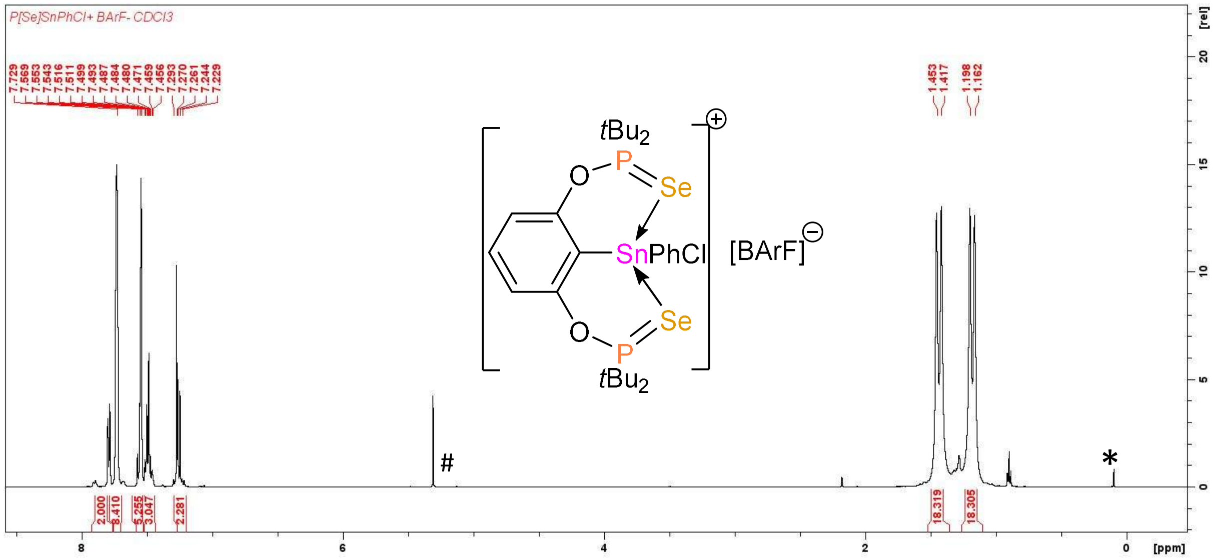

Figure S70. ^{1}H NMR spectrum of **2^{Se+}[BArF]$^{-}$** (500 MHz, $CDCl_3$). *Signal of silicon grease, #signal of co-crystallized dichloromethane.

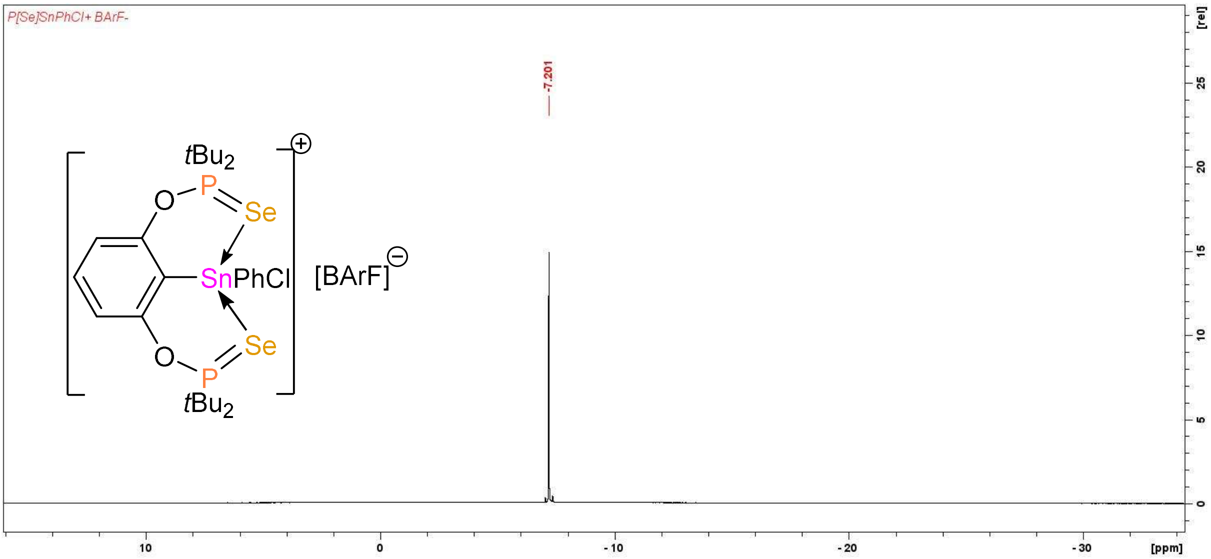

Figure S71. $^{11}B\{^{1}H\}$ NMR spectrum of $\mathbf{2^{Se+}[BArF]^{-}}$ (160.42 MHz, $CDCl_3$).

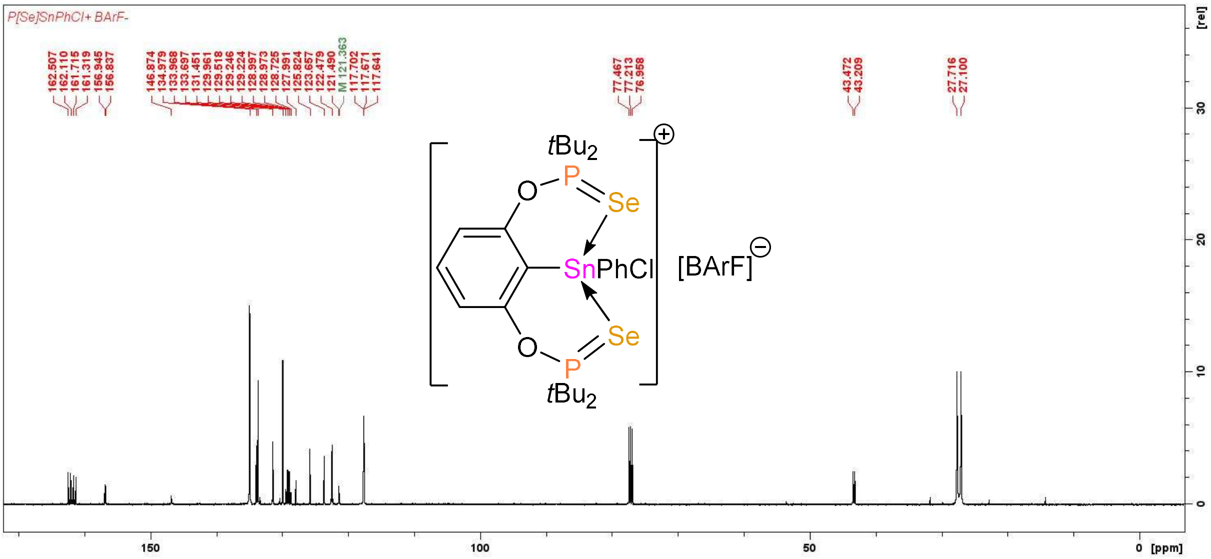

Figure S72. $^{13}C\{^{1}H\}$ NMR spectrum of $\mathbf{2^{Se+}[BArF]^{-}}$ (125.76 MHz, $CDCl_3$).

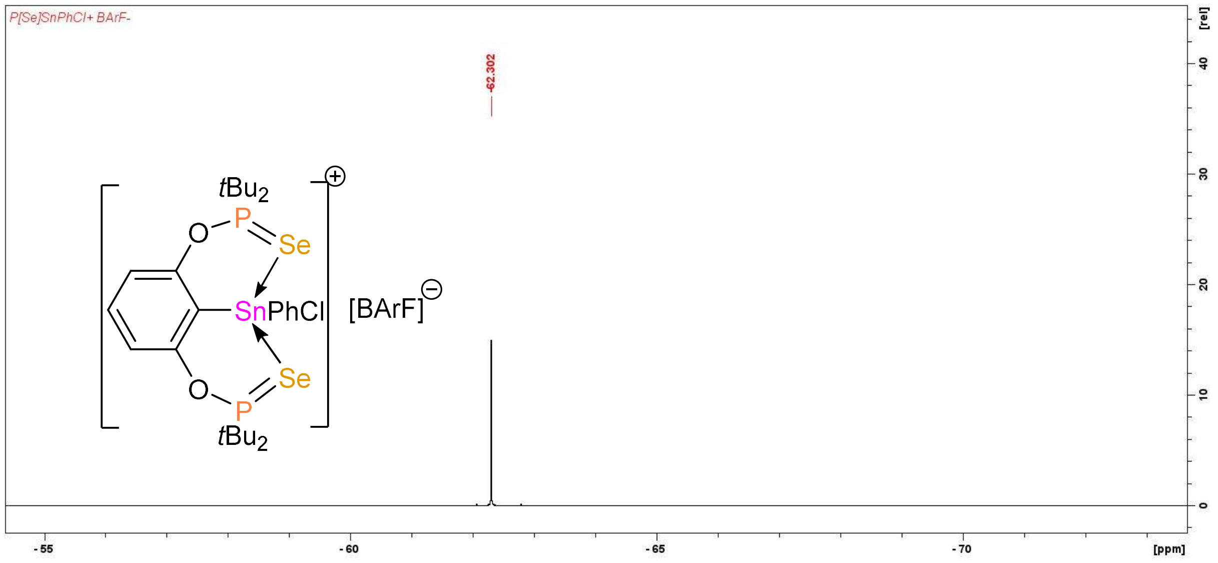

Figure S73. $^{19}F\{^{1}H\}$ NMR spectrum of $\mathbf{2^{Se+}[BArF]^{-}}$ (470.5 MHz, $CDCl_3$).

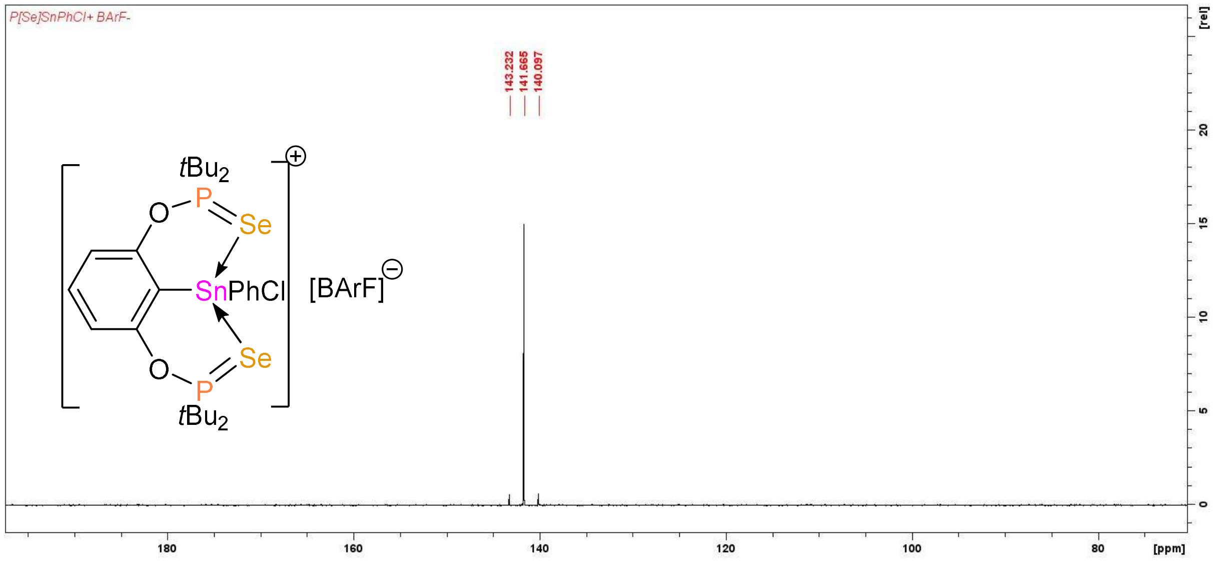

Figure S74. $^{31}P\{^{1}H\}$ NMR spectrum of $\mathbf{2^{Se+}[BArF]^{-}}$ (202.5 MHz, $CDCl_3$).

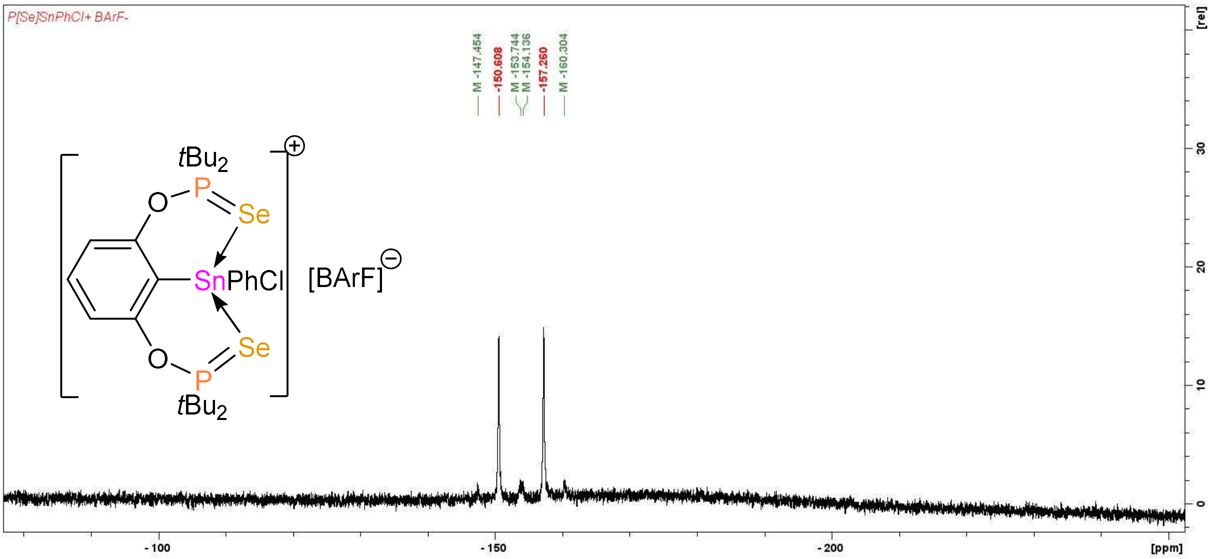

Figure S75. $^{77}Se\{^1H\}$ NMR spectrum of $\mathbf{2^{Se+}[BArF]^-}$ (95.4 MHz, $CDCl_3$).

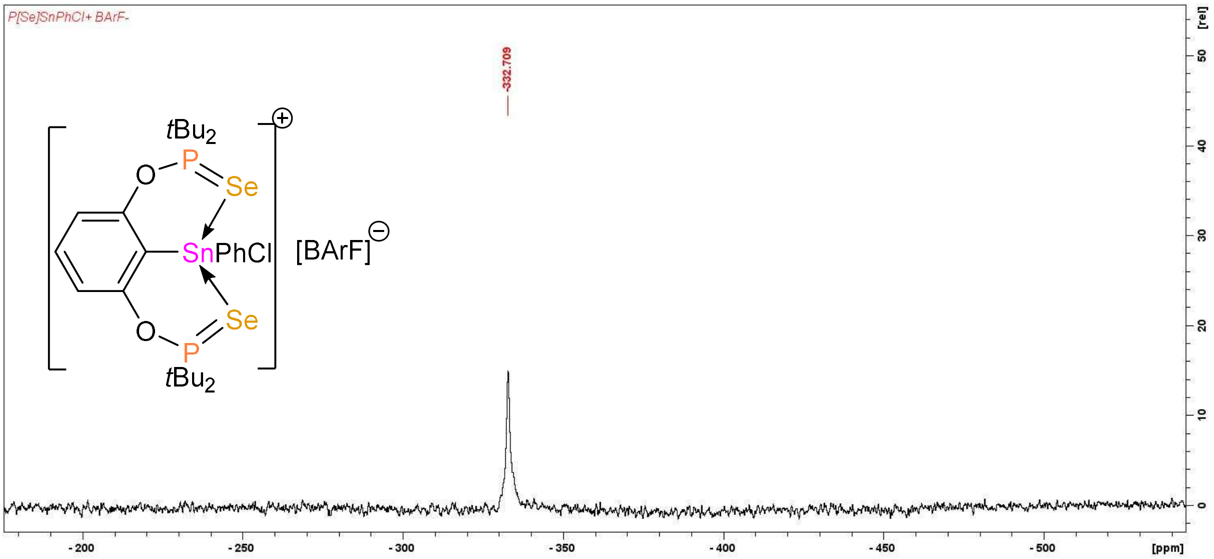

Figure S76. $^{119}Sn\{^1H\}$ NMR spectrum of $\mathbf{2^{Se+}[BArF]^-}$ (186.5 MHz, $CDCl_3$).

Synthesis of [2,6-(*t*Bu$_2$(S)PO)C$_6$H$_3$]SnPh$_2$Cl (3^S)

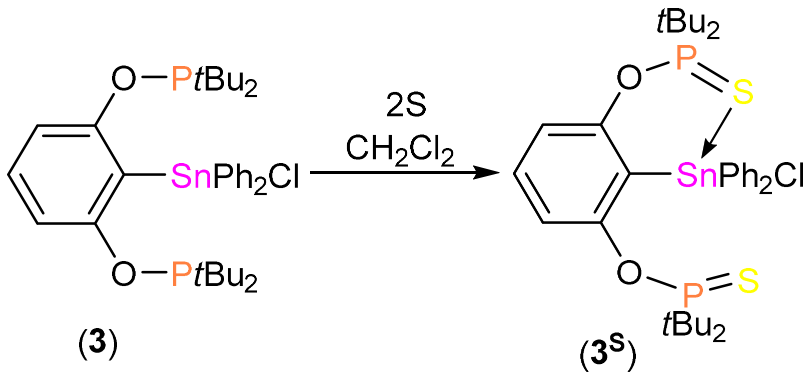

Elemental sulfur (38 mg; 1.19 mmol) was added in one portion to solution of **3** (417 mg; 0.59 mmol) in dichloromethane (20 ml). The reaction mixture was stirred for 24 h at room temperature and then was concentrated to ½ of the original volume. Colorless solution was layered with hexane. Crystallization at room temperature gave colorless crystals of compound **3^S**, whereas another batch of crystals could be obtained from mother liquor by crystallization at -30 °C. Combined yield of **3^S** was 285 mg, (90 %), m. p. 151-154 °C. Single-crystals suitable for *sc*-XRD diffraction analysis were obtained by slow diffusion of hexane into saturated dichloromethane solution at room temperature. Anal. Calcd for $C_{34}H_{49}ClO_2P_2S_2Sn$ (MW 769.99): C, 53.0; H, 6.4 %. Found: C, 53.4; H, 6.8 %. **^{1}H NMR** (500 MHz, C_6D_6) δ (ppm): 1.06 [36H, d, $^3J(^{31}P,^1H)$ = 16.5 Hz, *t*Bu$_2$(S)P-C*H*$_3$], 6.99 [1H, t, $^3J(^1H,^1H)$ = 8.3 Hz, Ar-*H*], 7.08 [2H, t, $^3J(^1H,^1H)$ = 7.3 Hz, Ar-*H*], 7.14 [4H, t, $^3J(^1H,^1H)$ = 7.3 Hz, Ar-*H*], 7.63 [2H, d(br), $^3J(^1H,^1H)$ = 8.3 Hz, Ar-*H*], 8.13 [4H, d, $^3J(^1H,^1H)$ = 7.2 Hz, $^2J(^{119/117}Sn, ^1H)$ = 72.5 Hz, Ar-*H*]. **^{13}C{^{1}H} NMR** (125.78 MHz, C_6D_6) δ (ppm): 27.6 [s, *t*Bu$_2$(S)P-*C*H$_3$], 41.9 [d, $^1J(^{31}P,^{13}C)$ = 54.3 Hz, *t*Bu$_2$(S)P-*C*], 119.2 [d, $^3J(^{31}P,^{13}C)$ = 3.7 Hz, $^3J(^{119/117}Sn,^{13}C)$ = 30.3 Hz, Ar-*C*], 125.9 [t, $^2J(^{31}P,^{13}C)$ = 4.6 Hz, Ar-*C*], 128.6 [s, $^3J(^{119/117}Sn,^{13}C)$ = 72 Hz, Ar-*C*], 129.2 [s, $^4J(^{119/117}Sn,^{13}C)$ = 14.8 Hz, Ar-*C*], 130.7 [s, Ar-*C*], 137.5 [s, $^2J(^{119/117}Sn,^{13}C)$ = 51.9 Hz, Ar-*C*], 145.5 [s, Ar-*C*], 159.0 [d, $^2J(^{31}P,^{13}C)$ = 11.6 Hz, Ar-*C*]. **^{31}P{^{1}H} NMR** (202.5 MHz, C_6D_6) δ (ppm): 131.3 [s]. **^{119}Sn{^{1}H} NMR** (186.5 MHz, C_6D_6) δ (ppm): -209.2 [s].

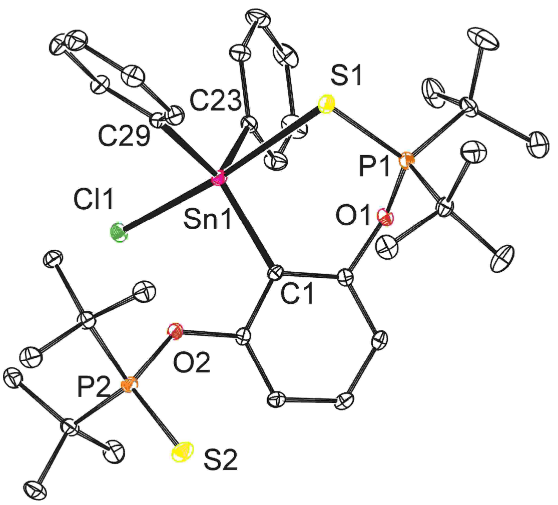

Figure S77. Molecular structure of $\mathbf{3}^{\mathrm{S}}$. ORTEP with 30% probability ellipsoid level. Only one of two independent molecules is presented. Hydrogen atoms and dichloromethane solvate molecules are omitted. Selected structural bond lengths [Å]: Sn(1)-C(1) 2.1448(16), Sn(1)-Cl(1) 2.4814(5), Sn(1)-C(23) 2.1356(17), Sn(1)-C(29) 2.1320(17), Sn(1)-S(1) 2.8587(5), Sn(1)-S(2) 6.1416(6); bonding angles [°] Cl(1)-Sn(1)-S(1) 174.75(2), C(1)-Sn(1)-C(23) 128.75(7), C(1)-Sn(1)-C(29) 113.23(6), C(23)-Sn(1)-C(29) 116.33(7). Selected structural bond lengths for the second independent molecule [Å]: Sn(2)-C(101) 2.1435(16), Sn(2)-Cl(2) 2.4897(5), Sn(2)-C(123) 2.1366(17), Sn(2)-C(129) 2.1316(17), Sn(2)-S(3) 2.8236(5), Sn(2)-S(4) 6.1478(6); bonding angles [°] Cl(2)-Sn(2)-S(3) 175.40(2), C(101)-Sn(2)-C(123) 128.40(7), C(101)-Sn(2)-C(129) 114.91(6), C(123)-Sn(2)-C(129) 115.34(7).

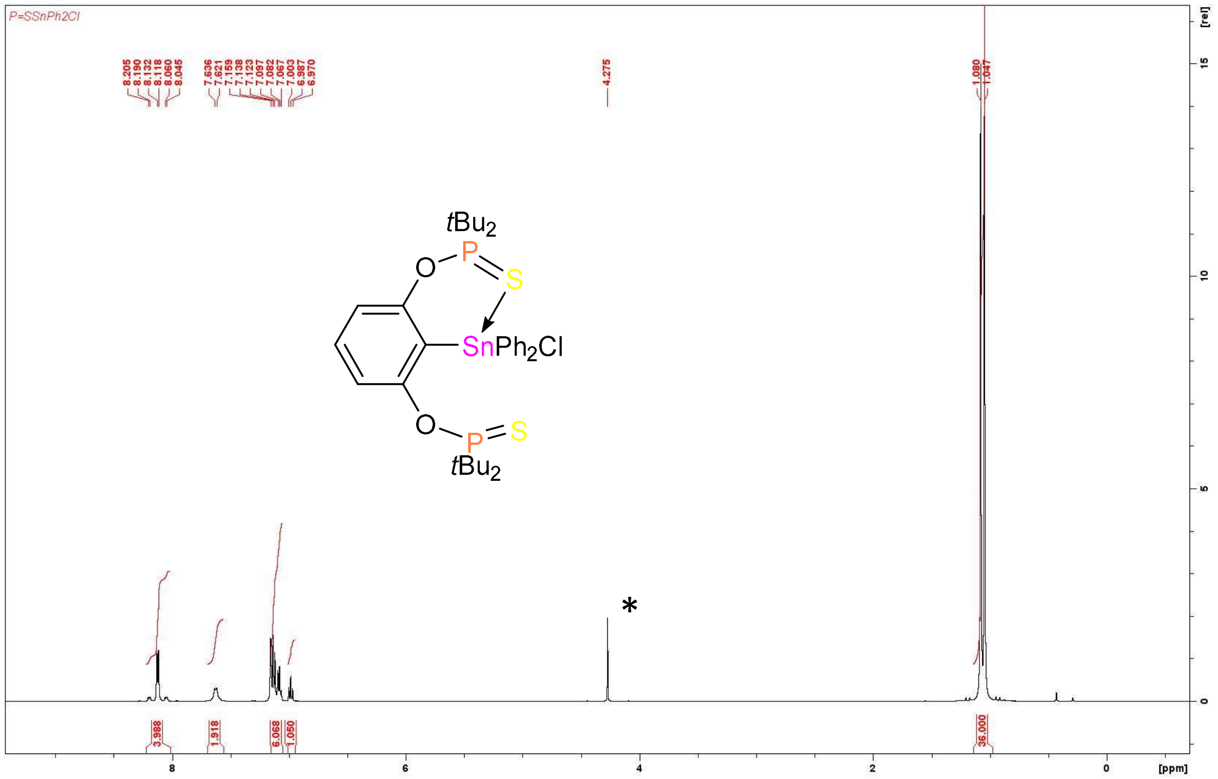

Figure S78. ^{1}H NMR spectrum of **3**^{S} (500 MHz, C_6D_6). *Signal of co-crystallized dichloromethane.

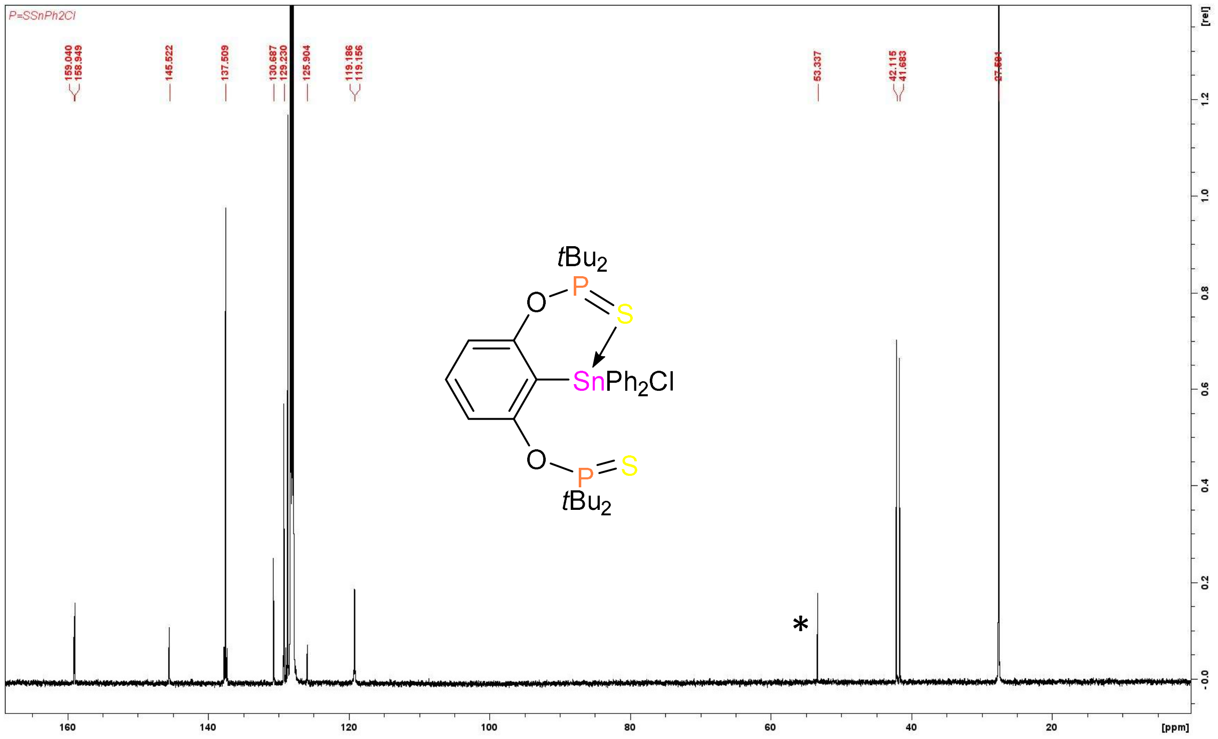

Figure S79. $^{13}C\{^{1}H\}$ NMR spectrum of **3**^{S} (125.76 MHz, C_6D_6). *Signal of co-crystallized dichloromethane.

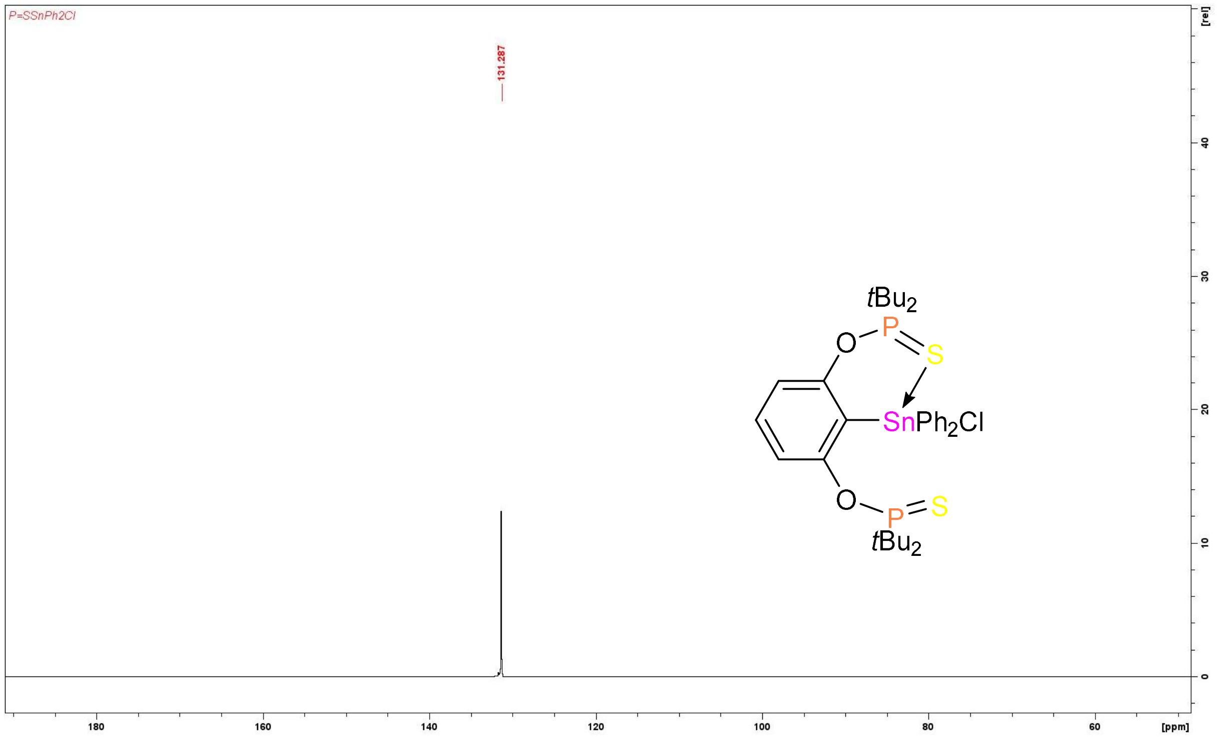

Figure S80. $^{31}P\{^1H\}$ NMR spectrum of **3**^{S} (202.5 MHz MHz, C_6D_6).

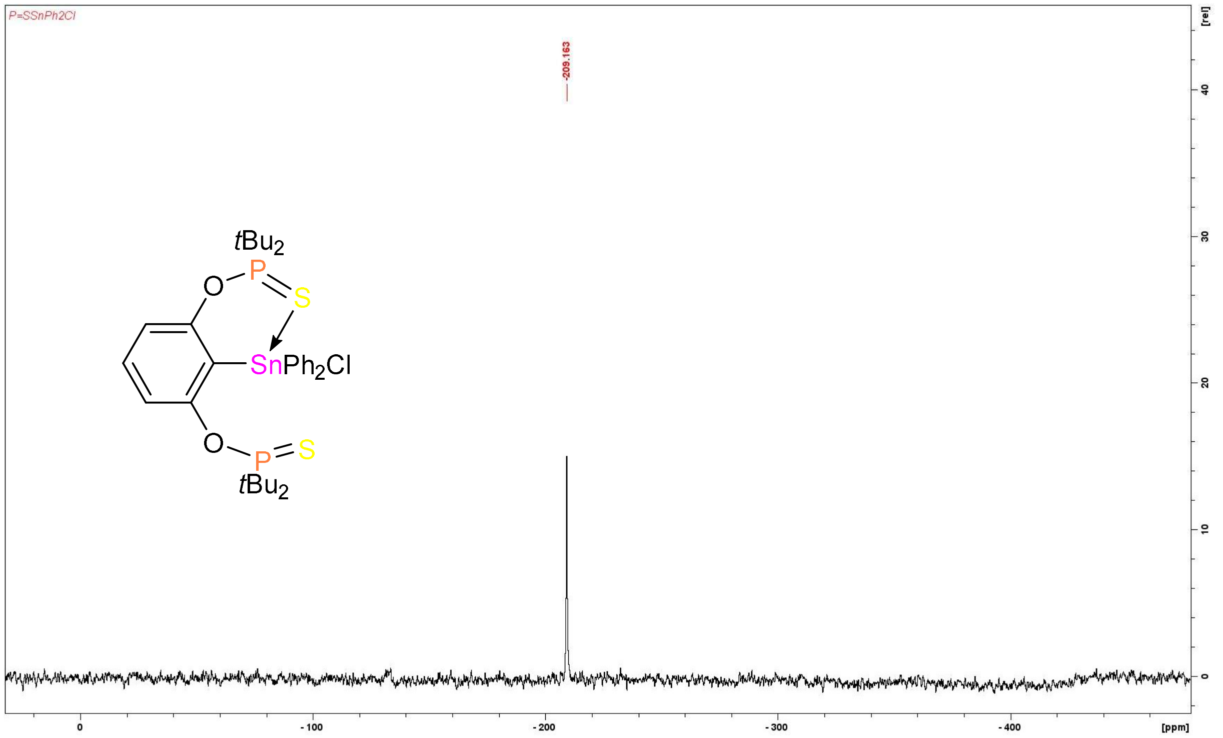

Figure S81. $^{119}Sn\{^1H\}$ NMR spectrum of **3**^{S} (186.5MHz, C_6D_6).

Synthesis of [2,6-(tBu$_2$(Se)PO)C$_6$H$_3$]SnPh$_2$Cl (3^{Se})

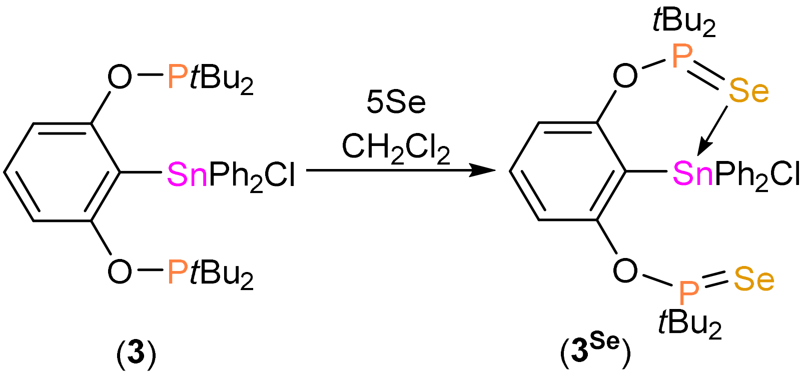

Elemental selenium (180 mg; 2.28 mmol) was added in one portion to solution of **3** (320 mg; 0.45 mmol) in dichloromethane (10 ml). The reaction mixture was stirred for 1 h at room temperature and then unreacted selenium was removed by filtration. Resulting colorless solution was concentrated to 1/4 of the original volume and was layered with hexane. Crystallization at -30 °C gave colorless crystals of compound **3^{Se}.** Yield of **3^{Se}** was 221 mg, (89 %), m. p. 218-22 °C. Single-crystals suitable for *sc*-XRD diffraction analysis were obtained from saturated solution using dichloromethane/hexane mixture at -30 °C. Anal. Calcd for $C_{34}H_{49}ClO_2P_2Se_2Sn$ (MW 863.81): C, 47.3; H, 5.7 %. Found: C, 47.0; H, 5.8 %.**^{1}H NMR** (500 MHz, CDCl$_3$) δ (ppm): 1.17 [36H, d, $^{3}J(^{31}P,^{1}H)$ = 17.0 Hz, tBu$_2$(Se)P-CH_3], 7.34 [7H, m, Ar-H], 7.68 [2H, d, $^{3}J(^{1}H,^{1}H)$ = 8.3 Hz, Ar-H], 7.86 [4H, d, $^{3}J(^{1}H,^{1}H)$ = 6.9 Hz, $^{2}J(^{119/117}Sn, ^{1}H)$ = 74.0 Hz, Ar-H]. **^{13}C{^{1}H} NMR** (125.78 MHz, CDCl$_3$) δ (ppm): 27.9 [s, tBu$_2$(Se)P-CH$_3$], 42.9 [d, $^{1}J(^{31}P,^{13}C)$ = 42.4 Hz, tBu$_2$(Se)P-C], 119.4 [d, $^{3}J(^{31}P,^{13}C)$ = 4.2 Hz, $^{3}J(^{119/117}Sn,^{13}C)$ = 30.9 Hz, Ar-C], 125.7 [7, $^{4}J(^{31}P,^{13}C)$ = 4.8 Hz, Ar-C], 128.5 [s, $^{3}J(^{119/117}Sn,^{13}C)$ = 77.6 Hz, Ar-C], 129.2 [s, $^{4}J(^{119/117}Sn,^{13}C)$ = 15.7 Hz, Ar-C], 130.2 [s, Ar-C], 137.0 [s, $^{2}J(^{119/117}Sn,^{13}C)$ = 51.8 Hz, Ar-C], 146.4 [s, Ar-C], 158.4 [d, $^{2}J(^{31}P,^{13}C)$ = 11.9 Hz, Ar-C]. **^{31}P{^{1}H} NMR** (202.5 MHz, CDCl$_3$) δ (ppm): 141.6 [s, $^{1}J(^{77}Se,^{31}P)$ = 748 Hz, tBu$_2$(Se)P]. **^{77}Se{^{1}H} NMR** (95.4 MHz, CDCl$_3$) δ (ppm): -252.7 [d, $^{1}J(^{77}Se,^{31}P)$ = 764 Hz] measured at 323K at r.t. the signal was too broad to be detected. **^{119}Sn{^{1}H} NMR** (186.5 MHz, CDCl$_3$) δ (ppm): -231.4 [s].

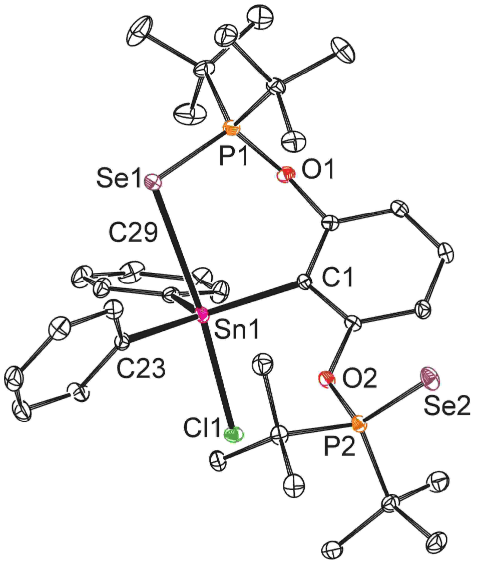

Figure S82. Molecular structure of $\mathbf{3}^{\mathbf{Se}}$. ORTEP with 30% probability ellipsoid level. Hydrogen atoms and dichloromethane solvate molecule are omitted. Hydrogen atoms and dichloromethane solvate molecules are omitted. Selected structural bond lengths [Å]: Sn(1)-C(1) 2.147(2), Sn(1)-Cl(1) 2.4844(6), Sn(1)-C(23) 2.135 (2), Sn(1)-C(29) 2.114(19), Sn(1)-Se(1) 3.0253(5), Sn(1)-Se(2) 6.2499(7); bonding angles [°] Cl(1)-Sn(1)-Se(1) 175.91(3), C(1)-Sn(1)-C(23) 112.06(8), C(1)-Sn(1)-C(29) 133.9(6), C(23)-Sn(1)-C(29) 112.9(6).

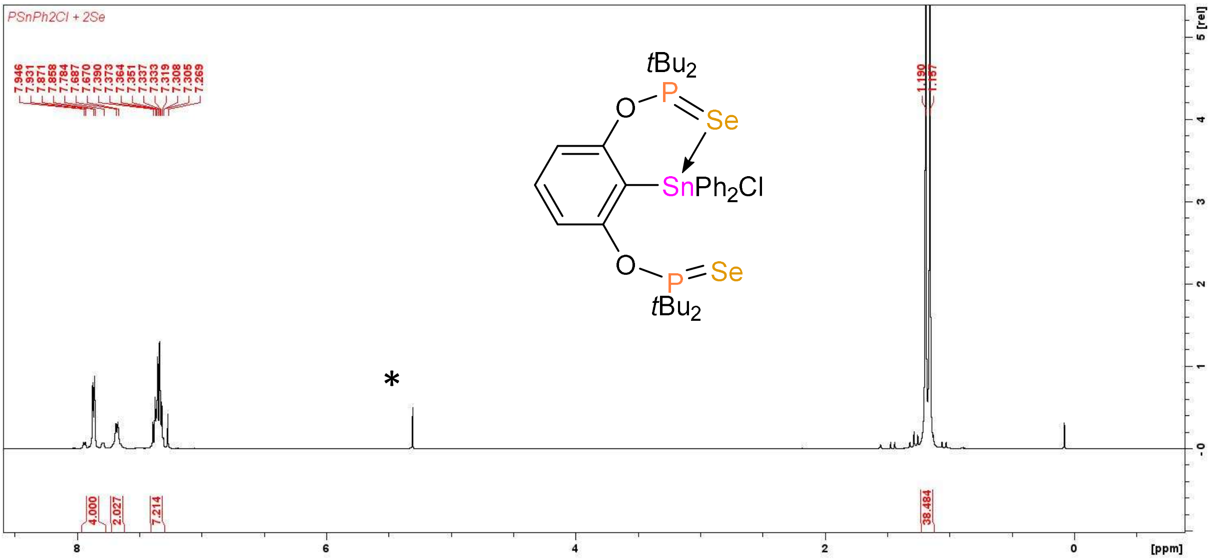

Figure S83. ^{1}H NMR spectrum of $\mathbf{3}^{\mathbf{Se}}$ (500 MHz, C_6D_6). *Signal of co-crystallized dichloromethane.

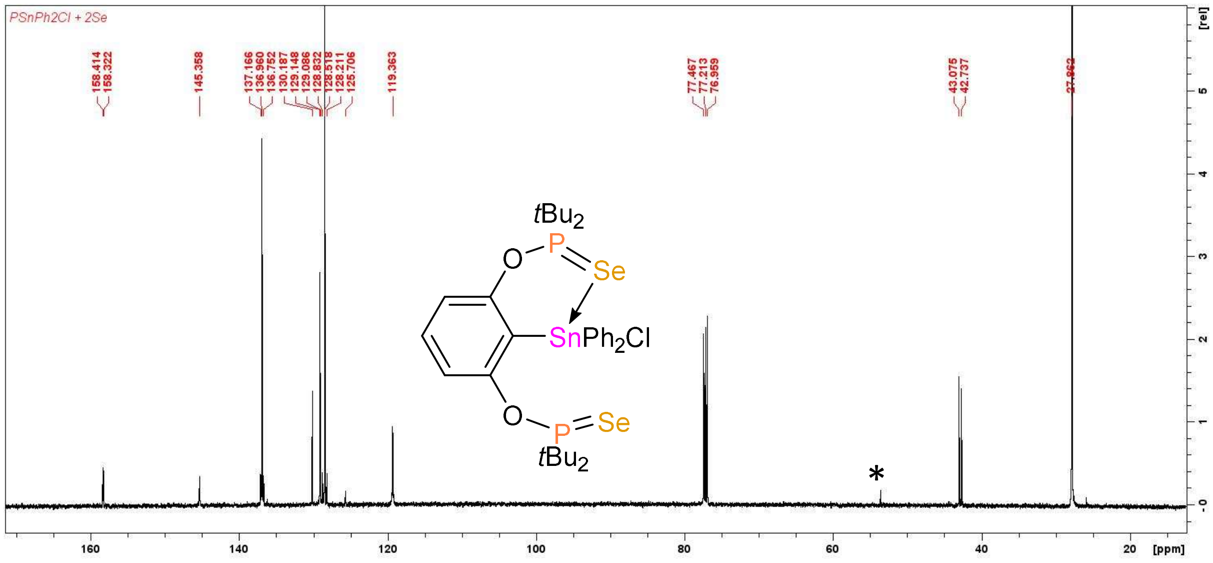

Figure S84. $^{13}C\{^1H\}$ NMR spectrum of **3**^{Se} (125.76 MHz, $CDCl_3$). *Signal of co-crystallized dichloromethane.

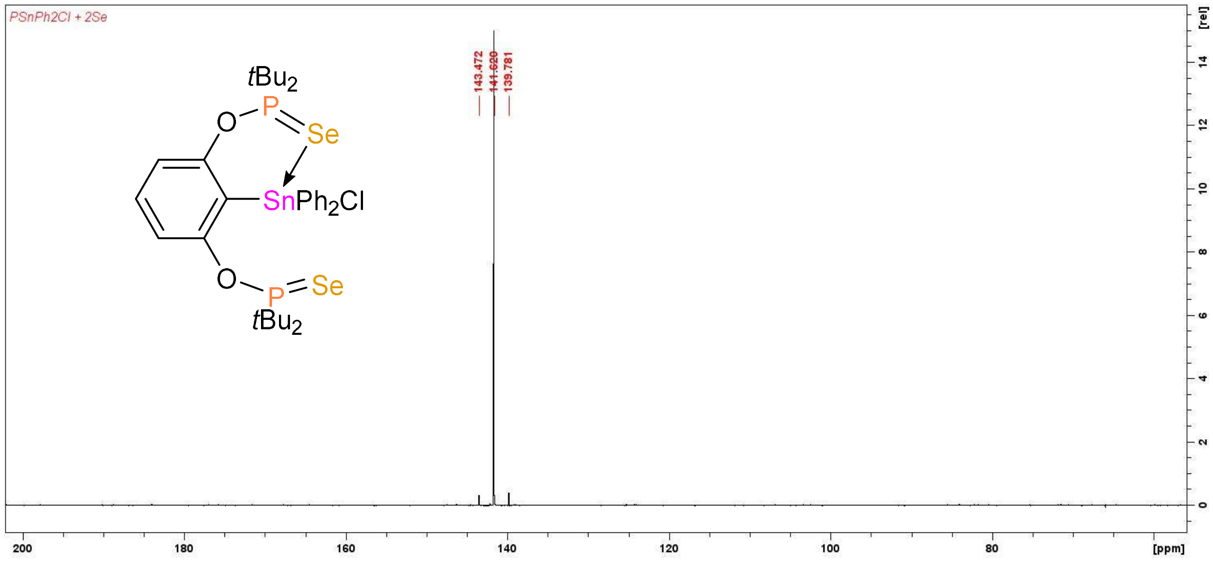

Figure S85. $^{31}P\{^1H\}$ NMR spectrum of **3**^{Se} (202.5 MHz MHz, $CDCl_3$).

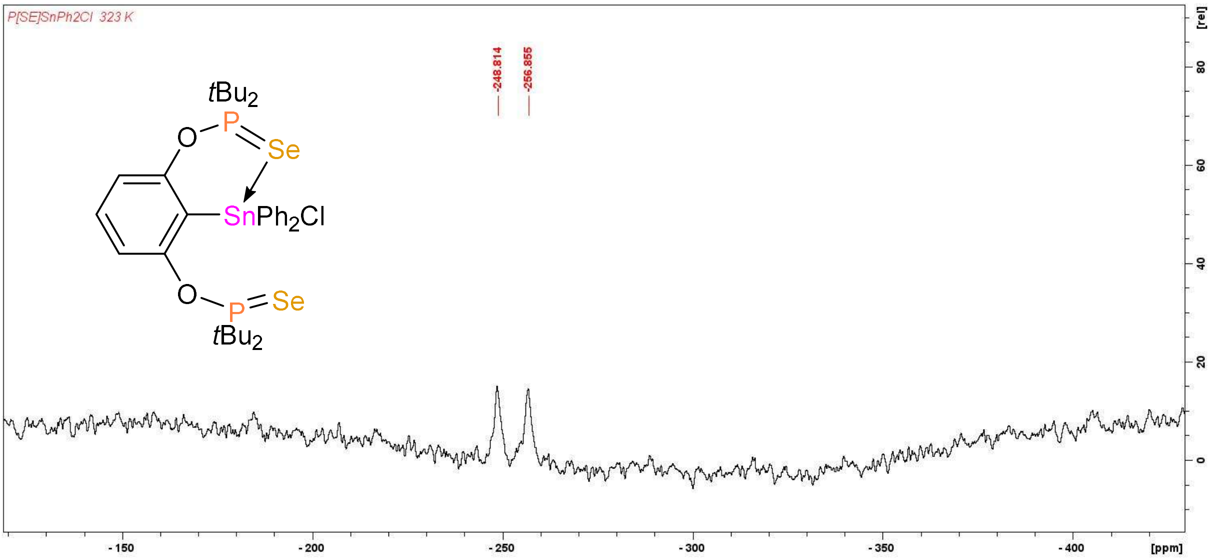

Figure S86. $^{77}Se\{^1H\}$ NMR spectrum of **3**$^{\mathbf{Se}}$ (95.4 MHz, $CDCl_3$). Measured at 323 K.

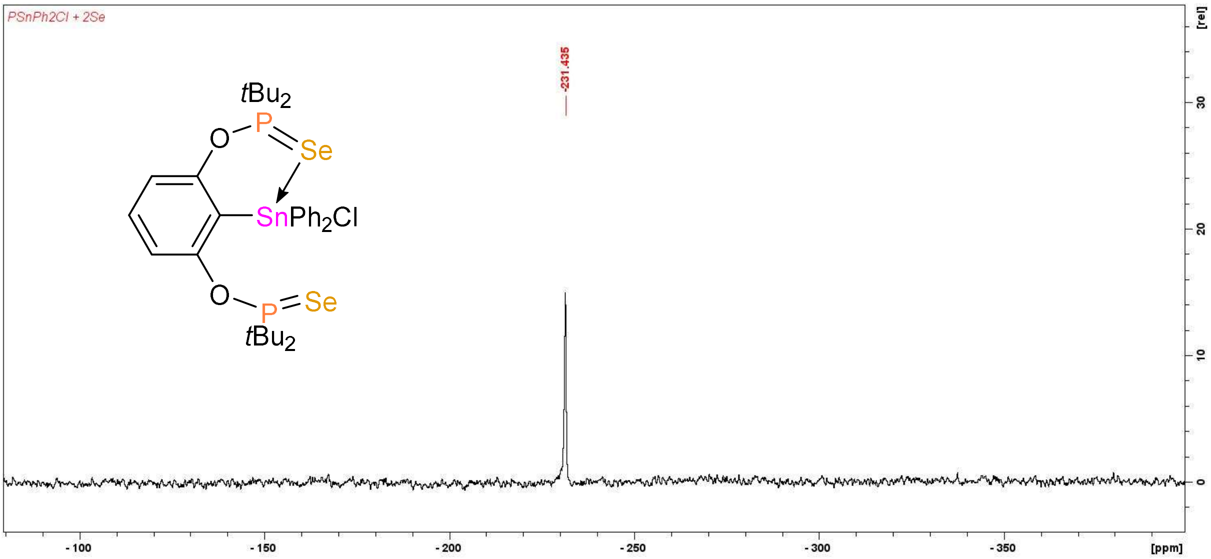

Figure S87. $^{119}Sn\{^1H\}$ NMR spectrum of **3**$^{\mathbf{Se}}$ (186.5MHz, $CDCl_3$).

Synthesis of {[2,6-(*t*Bu_2(S)PO)$_2C_6H_3$]SnPh_2}{B[3,5-(CF_3)$_2C_6H_3$]$_4$} (3^{S+}[BArF]$^-$)

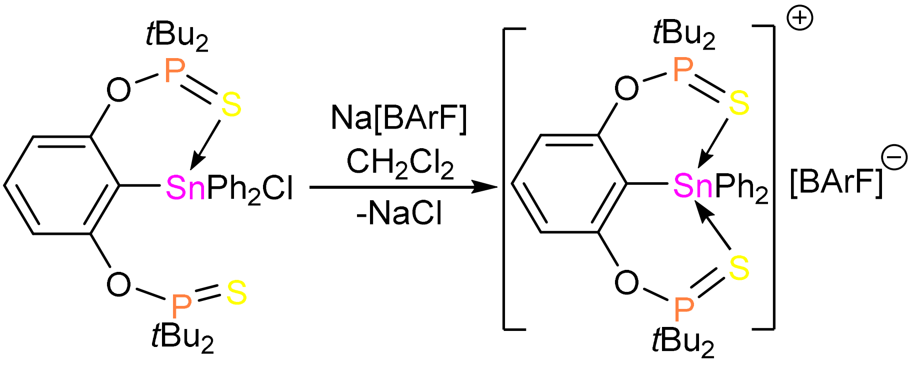

(3^S) (3^{S+}[BArF]$^-$)

Solid Na[BArF] (115 mg; 0.13 mmol) was added in one portion to solution of $\mathbf{3^S}$ (100 mg; 0.13 mmol) in dichloromethane (10 ml). The reaction mixture was stirred for 30 min at room temperature and then incipient NaCl was removed by filtration. Colorless solution was concentrated to 1/2 of the original volume and layered with hexane. Crystallization at room temperature gave colorless crystals of compound $\mathbf{3^{S+}[BArF]^-}$. Yield of $\mathbf{3^{S+}[BArF]^-}$ was 130 mg, (63 %), m. p. 218-220 °C. Single-crystals suitable for *sc*-XRD diffraction analysis were obtained from saturated solution using dichloromethane/hexane mixture at 5 °C. Anal. Calcd for $C_{66}H_{61}BF_{24}O_2P_2S_2Sn$ (MW 1597.76): C, 49.6; H, 3.9 %. Found: C, 49.9; H, 3.8 %. **^{1}H NMR** (500 MHz, CD_2Cl_2) δ (ppm): 1.15 [36H, d, $^3J(^{31}P,^1H)$ = 17.8 Hz, *t*Bu_2(S)P-CH_3], 7.31 [2H, d, $^3J(^1H,^1H)$ = 8.4 Hz, Ar-*H*], 7.48 [6H, m, Ar-*H*], 7.57 [4H, m, Ar-*H*], 7.65 [1H, t, $^3J(^1H,^1H)$ = 8.4 Hz, Ar-*H*], 7.74 [8H, s, Ar-*H*], 7.77 [4H, m, $^3J(^{119/117}Sn, ^1H)$ = 78.8 Hz, Ar-*H*]. **^{11}B{^{1}H} NMR** (160.42 MHz, CD_2Cl_2) δ (ppm): -7.0 [s]. **^{13}C{^{1}H} NMR** (125.78 MHz, CD_2Cl_2) δ (ppm): 27.2 [s(br), *t*Bu_2(S)P-CH_3], 42.5 [s(br), *t*Bu_2(S)P-*C*], 118.0 [m, Ar-*C*], 121.3 [t, $^nJ(^{31}P,^{13}C)$ = 3.5 Hz, Ar-*C*], 122.0 [d, $^3J(^{31}P,^{13}C)$ = 3.6 Hz, $^3J(^{119/117}Sn,^{13}C)$ = 28.5 Hz, Ar-*C*], 125.1 [q, $^1J(^{19}F,^{13}C)$ = 273 Hz, *C*F_3], 129.4 [qq, $^2J(^{19}F,^{13}C)$ = 31.8 Hz, $^4J(^{19}F,^{13}C)$ = 3.0 Hz, Ar-*C*], 130.0 [s, $^3J(^{119/117}Sn,^{13}C)$ = 82.1 Hz, Ar-*C*], 131.0 [s, $^4J(^{119/117}Sn,^{13}C)$ = 16.5 Hz, Ar-*C*], 134.3 [s, Ar-*C*], 135.3 [s, Ar-*C*], 136.4 [s, $^2J(^{119/117}Sn,^{13}C)$ = 52.5 Hz Ar-*C*], 143.2 [s, $^1J(^{119/117}Sn,^{13}C)$ = 858/819 Hz, Ar-*C*]; 158.2 [d, $^2J(^{31}P,^{13}C)$ = 13.2 Hz, Ar-C]; 162.3 [q,

$^{1}J(^{13}C,^{11}B)$ = 50 Hz, Ar-*C*]. **$^{19}F\{^{1}H\}$ NMR** (376.3 MHz, CD_2Cl_2) δ (ppm): -62.8 [s]. **$^{31}P\{^{1}H\}$ NMR** (202.5 MHz, CD_2Cl_2) δ (ppm): 132.6 [s]. **$^{119}Sn\{^{1}H\}$ NMR** (186.5 MHz, CD_2Cl_2) δ (ppm): -298.9 [s].

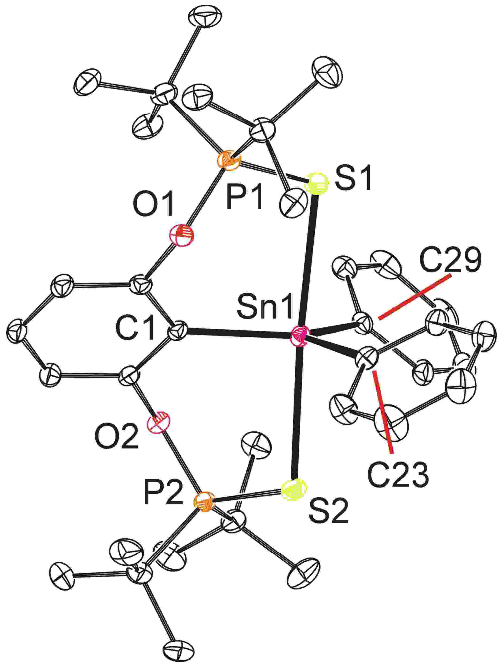

Figure S88. Molecular structure of **3^{S+}[BArF]$^{-}$**. ORTEP with 30% probability ellipsoid level. Hydrogen atoms and [BArF] anion are omitted. Selected structural bond lengths [Å]: Sn(1)-C(1) 2.150(2), Sn(1)-C(23) 2.124(2), Sn(1)-C(29) 2.117(2), Sn(1)-S(1) 2.6958(7), Sn(1)-S(2) 2.7070(7); bonding angles [°]: S(1)-Sn(1)-S(2) 176.35(2), C(1)-Sn(1)-C(23) 120.72(9), C(1)-Sn(1)-C(29) 123.55(9), C(23)-Sn(1)-C(29) 115.70(9).

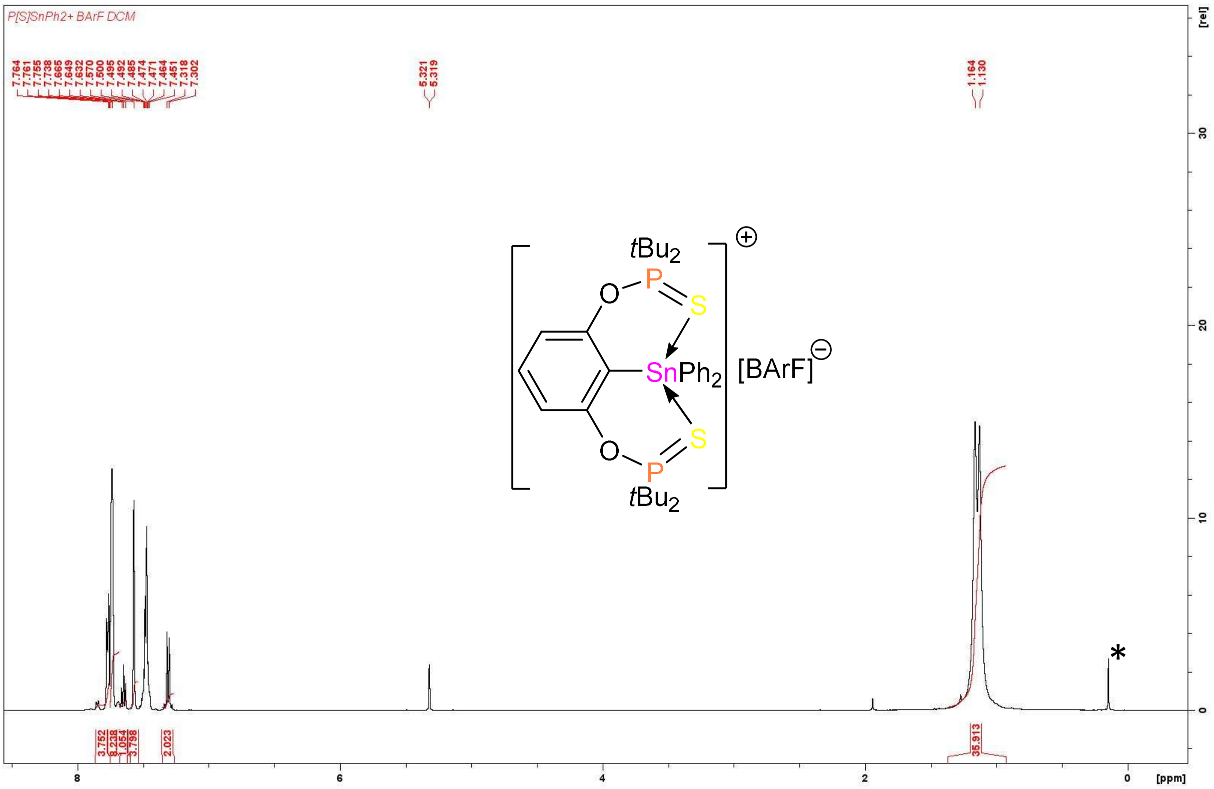

Figure S89. ^{1}H NMR spectrum of **3**$^{S+}$**[BArF]**$^{-}$ (500 MHz, CD_2Cl_2). *Signal of silicon grease.

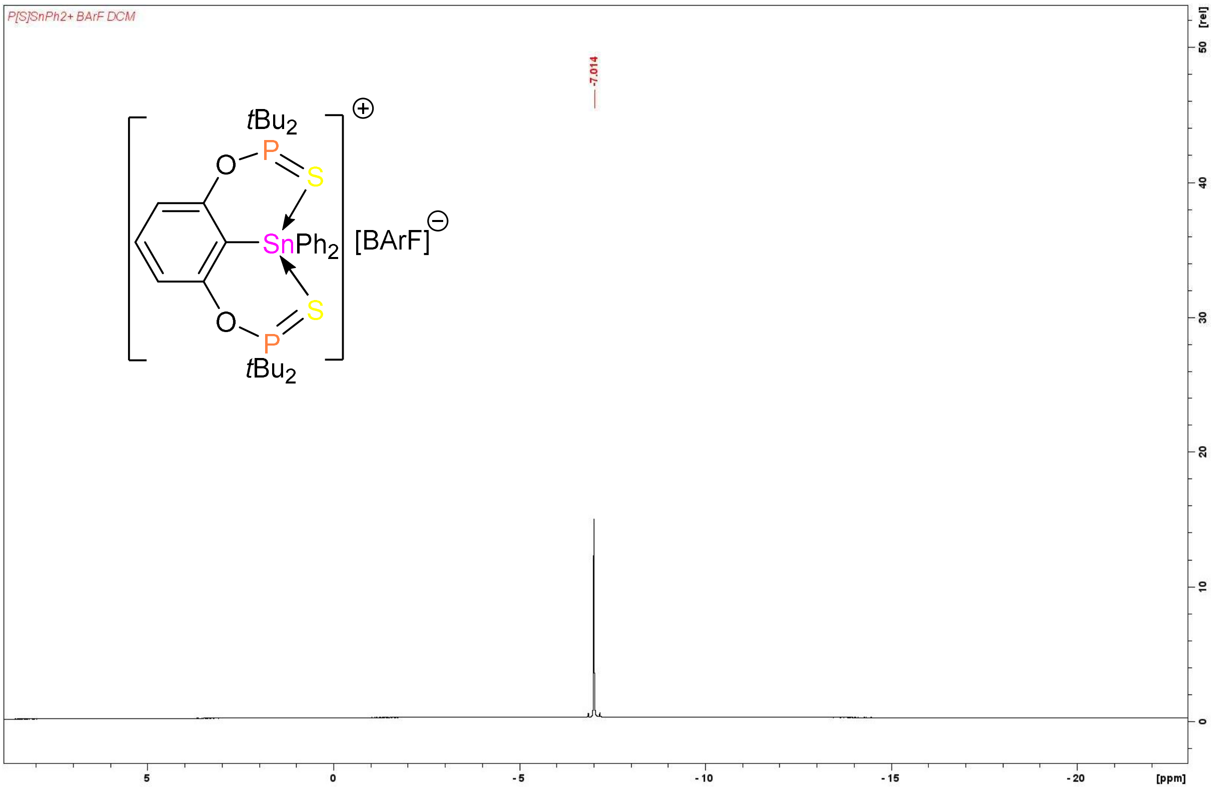

Figure S90. ^{11}B{^{1}H} NMR spectrum of **3**$^{S+}$**[BArF]**$^{-}$ (160.42 MHz MHz, CD_2Cl_2).

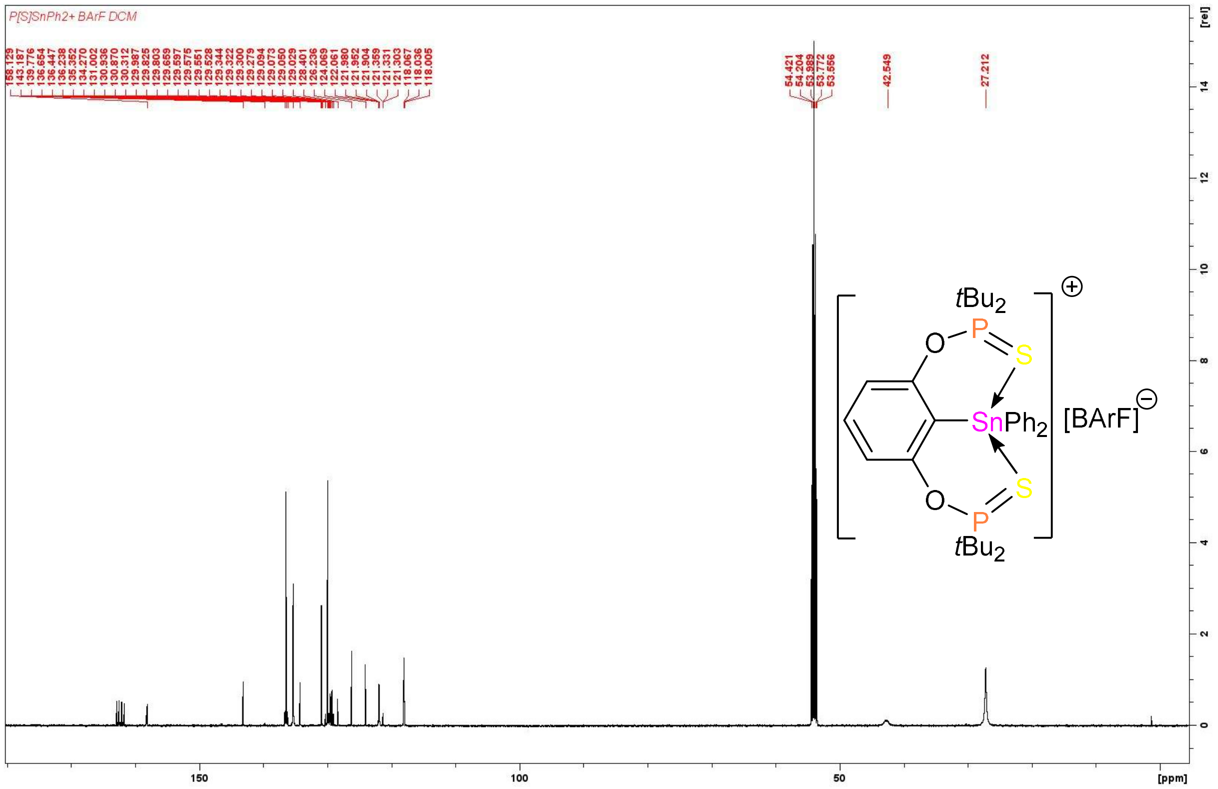

Figure S92. $^{13}C\{^1H\}$ NMR spectrum of **3^{S+}[BArF]$^-$** (125.76 MHz, CD_2Cl_2).

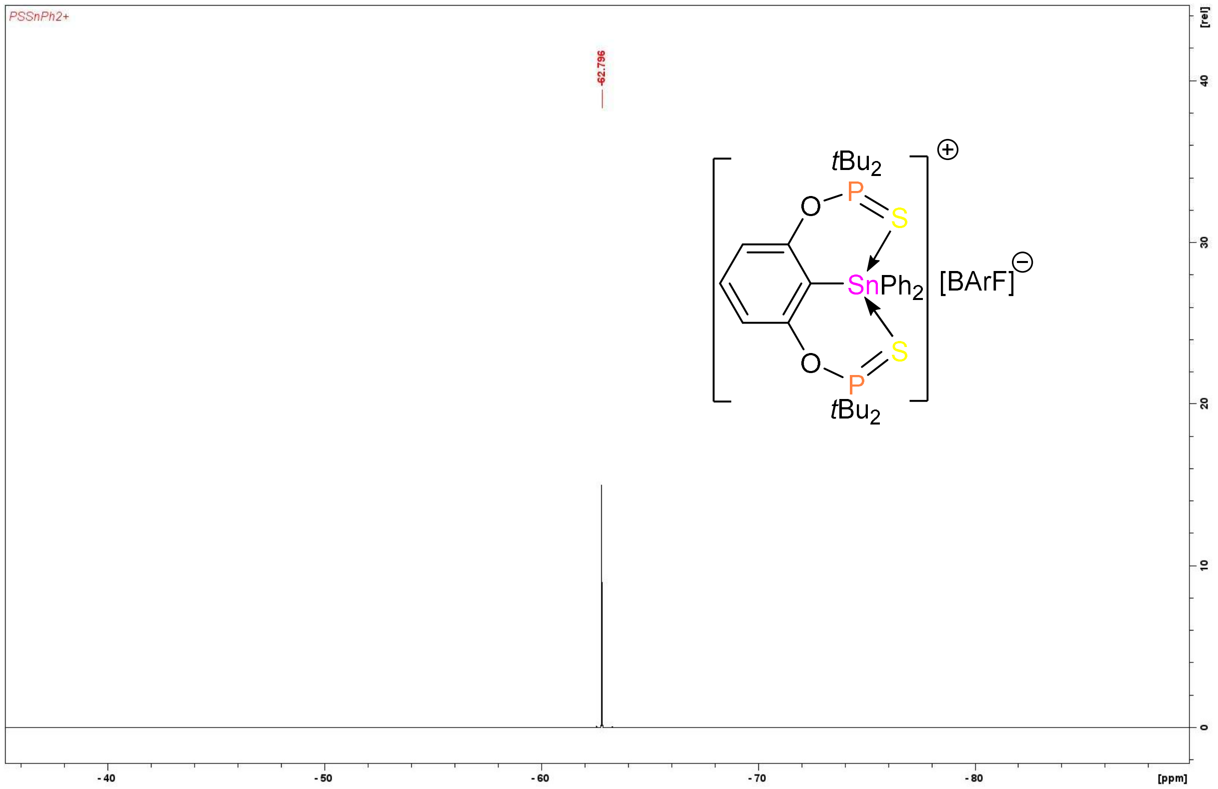

Figure S93. $^{19}F\{^1H\}$ NMR spectrum of **3^{S+}[BArF]$^-$** (470.5 MHz, CD_2Cl_2).

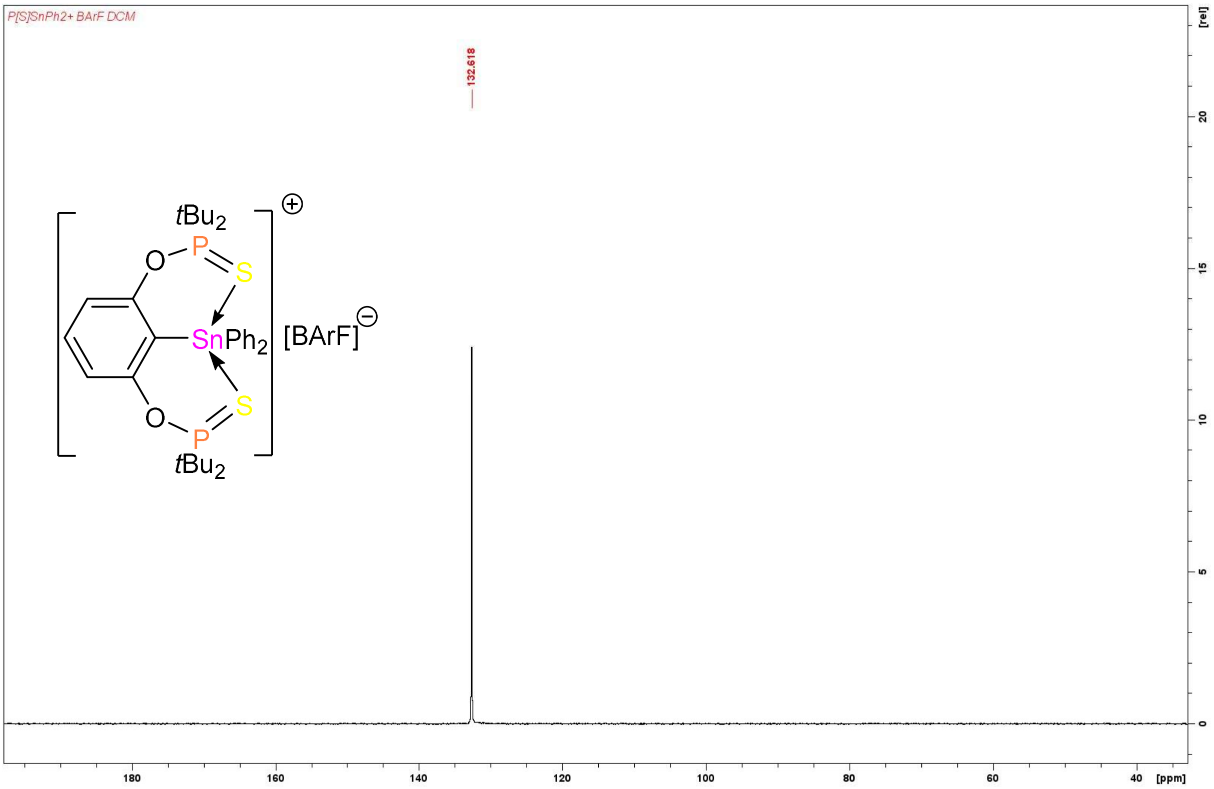

Figure S94. $^{31}P\{^1H\}$ NMR spectrum of $\mathbf{3^{S+}[BArF]^-}$ (202.5 MHz MHz, CD_2Cl_2).

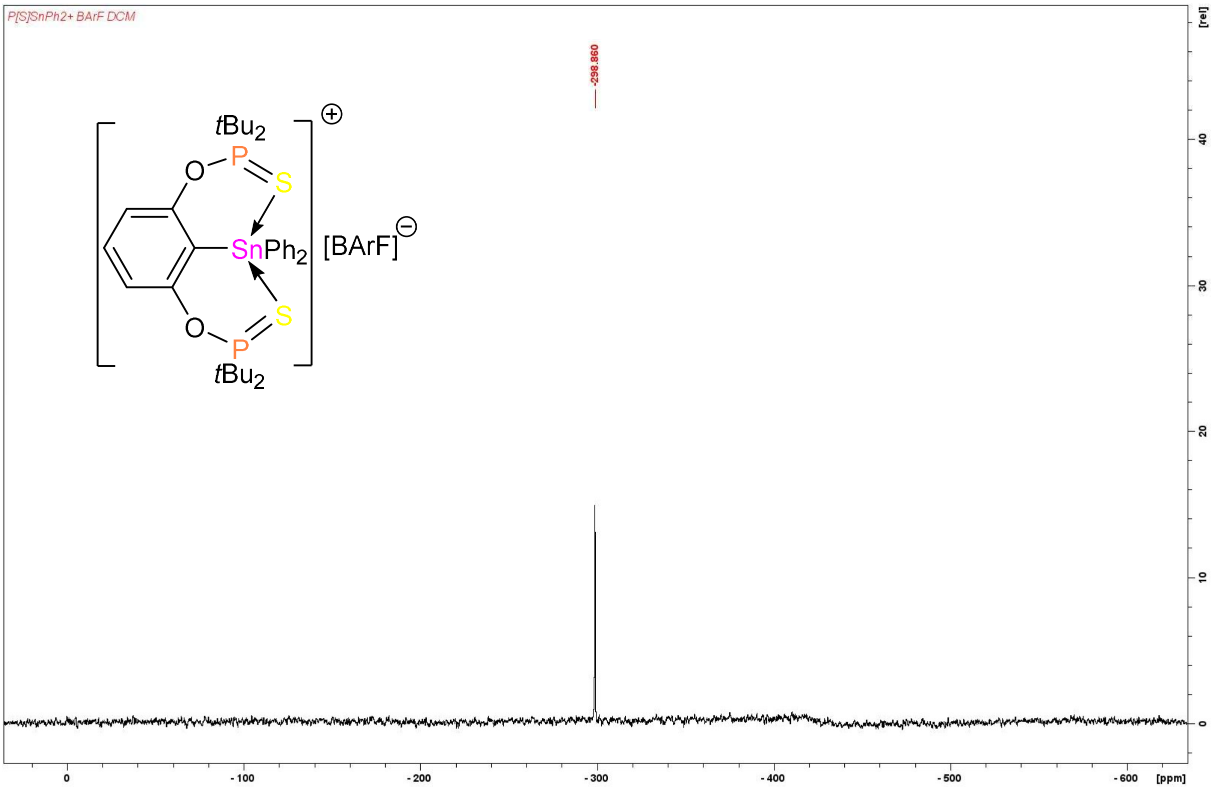

Figure S95. $^{119}Sn\{^1H\}$ NMR spectrum of $\mathbf{3^{S+}[BArF]^-}$ (186.5MHz, CD_2Cl_2).

Synthesis of {[2,6-(tBu$_2$(Se)PO)$_2$C$_6$H$_3$]SnPh$_2$}{[B[3,5-(CF$_3$)$_2$C$_6$H$_3$]$_4$} (3^{Se+}[BArF]$^-$)

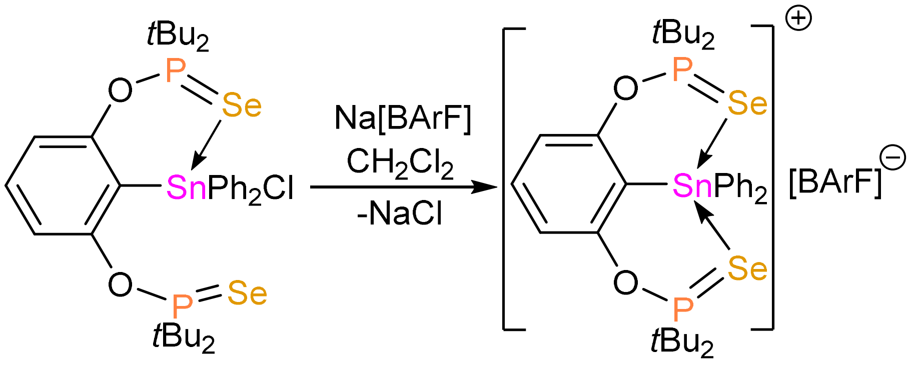

(**3**^{Se}) ([**3**^{Se}][BArF])

Solid Na[BArF] (78 mg; 0.09 mmol) was added in one portion to solution of **3**^{Se} (81 mg; 0.09 mmol) in dichloromethane (10 ml). The reaction mixture was stirred for one hour at room temperature and then incipient NaCl was removed by filtration. Colorless solution was concentrated to 1/2 of the original volume and layered with hexane. Crystallization at -30 °C gave colorless crystals of compound **3^{Se+}[BArF]$^-$**. Yield of **3^{Se+}[BArF]$^-$** was 103 mg, (69 %), m. p. 224-226 °C. Single-crystals suitable for *sc*-XRD diffraction analysis were obtained from saturated solution using dichloromethane/hexane mixture at 5 °C. Anal. Calcd for $C_{66}H_{61}BF_{24}O_2P_2Se_2Sn$ (MW 1691.58): C, 46.9; H, 3.6 %. Found: C, 47.0; H, 3.8 %. **^{1}H NMR** (500 MHz, CDCl$_3$) δ (ppm): 1.15 [36H, s(br), tBu$_2$(Se)P-C*H*$_3$], 7.25 [2H, m, Ar-*H*], 7.47 [6H, m, Ar-*H*], 7.54 [5H, m, Ar-*H*], 7.73 [8H, m, Ar-*H*], 7.79 [4H, m, ^3J($^{119/117}$Sn, ^{1}H) = 80.1 Hz, Ar-*H*]. **^{11}B{^{1}H} NMR** (160.42 MHz, CDCl$_3$) δ (ppm): -7.3 [s]. **^{13}C{^{1}H} NMR** (125.78 MHz, CDCl$_3$) δ (ppm): 27.3 [s(br), tBu$_2$(Se)P-*C*H$_3$], 43.4 [s(br), tBu$_2$(Se)P-*C*], 117.7 [m, Ar-*C*], 120.6 [t, ^nJ(^{31}P,^{13}C) = 3.6 Hz, Ar-*C*], 122.2 [s(br), ^nJ($^{119/117}$Sn,^{13}C) = 27.3 Hz, Ar-*C*], 124.8 [q, ^1J(^{19}F,^{13}C) = 273 Hz, *C*F$_3$], 129.1 [qq, ^2J(^{19}F,^{13}C) = 31.5 Hz, ^4J(^{19}F,^{13}C) = 2.8 Hz, Ar-*C*], 129.6 [s, ^3J($^{119/117}$Sn,^{13}C) = 81.7 Hz, Ar-*C*], 130.5 [s, ^4J($^{119/117}$Sn,^{13}C) = 16.7 Hz, Ar-*C*], 133.2 [s, Ar-*C*], 135.0 [s, Ar-*C*], 136.1 [s, ^2J($^{119/117}$Sn,^{13}C) = 52.6 Hz Ar-*C*], 143.2 [d, ^1J($^{119/117}$Sn,^{13}C) = 849/812 Hz, Ar-*C*], 157.5 [d, ^nJ(^{31}P,^{13}C) = 13.5 Hz, Ar-*C*], 161.9 [q, ^1J(^{13}C,^{11}B) = 50 Hz, Ar-*C*]. **^{19}F{^{1}H} NMR** (376.3 MHz, CDCl$_3$) δ (ppm): -62.4 [s]. **^{31}P{^{1}H} NMR** (202.5 MHz, CDCl$_3$)

δ (ppm): 141.4 [s, $^1J(^{77}Se,^{31}P)$ = 660 Hz, *t*Bu$_2$(Se)*P*]. **^{77}Se{^{1}H} NMR** (95.4 MHz, $CDCl_3$) δ (ppm): -207.1 [d, $^1J(^{77}Se,^{31}P)$ = 660 Hz, $^1J(^{119/117}Sn, ^{77}Se)$ = 400/381 Hz]. **^{119}Sn{^{1}H} NMR** (186.5 MHz, $CDCl_3$) δ (ppm): -311.7 [s, $^1J(^{119/117}Sn, ^{77}Se)$ = 400 Hz].

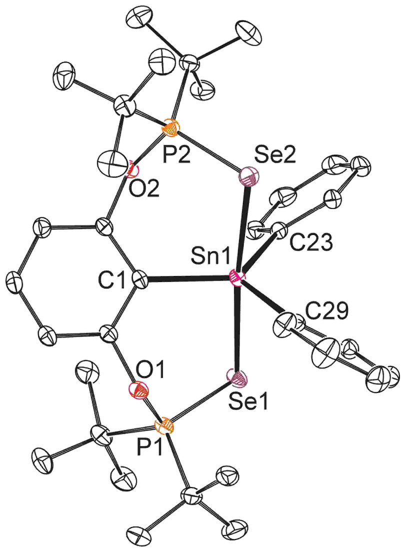

Figure S96. Molecular structure of **3^{Se+}[BArF]$^-$**. ORTEP with 30% probability ellipsoid level. Hydrogen atoms and [BArF] anion is not displayed. Selected structural bond lengths [Å]: Sn(1)-C(1) 2.157(2), Sn(1)-C(23) 2.125(3), Sn(1)-C(29) 2.118(3), Sn(1)-Se(1) 2.8097(6), Sn(1)-Se(2) 2.8257(6); bonding angles [°]: Se(1)-Sn(1)-Se(2) 175.05(2), C(1)-Sn(1)-C(23) 120.39(10), C(1)-Sn(1)-C(29) 122.63(10), C(23)-Sn(1)-C(29) 116.96(10).

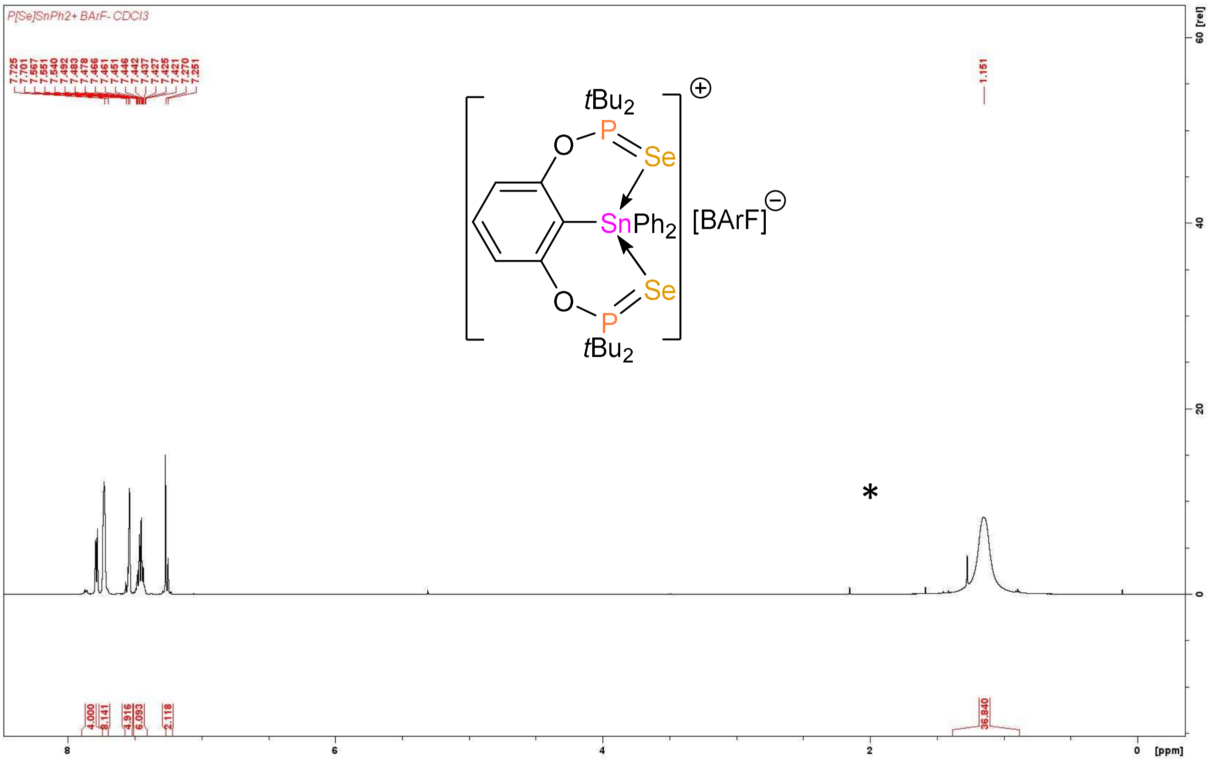

Figure S97. ^{1}H NMR spectrum of **3^{Se+}[BArF]$^{-}$** (500 MHz, CDCl$_3$). *Signal of minor impurity.

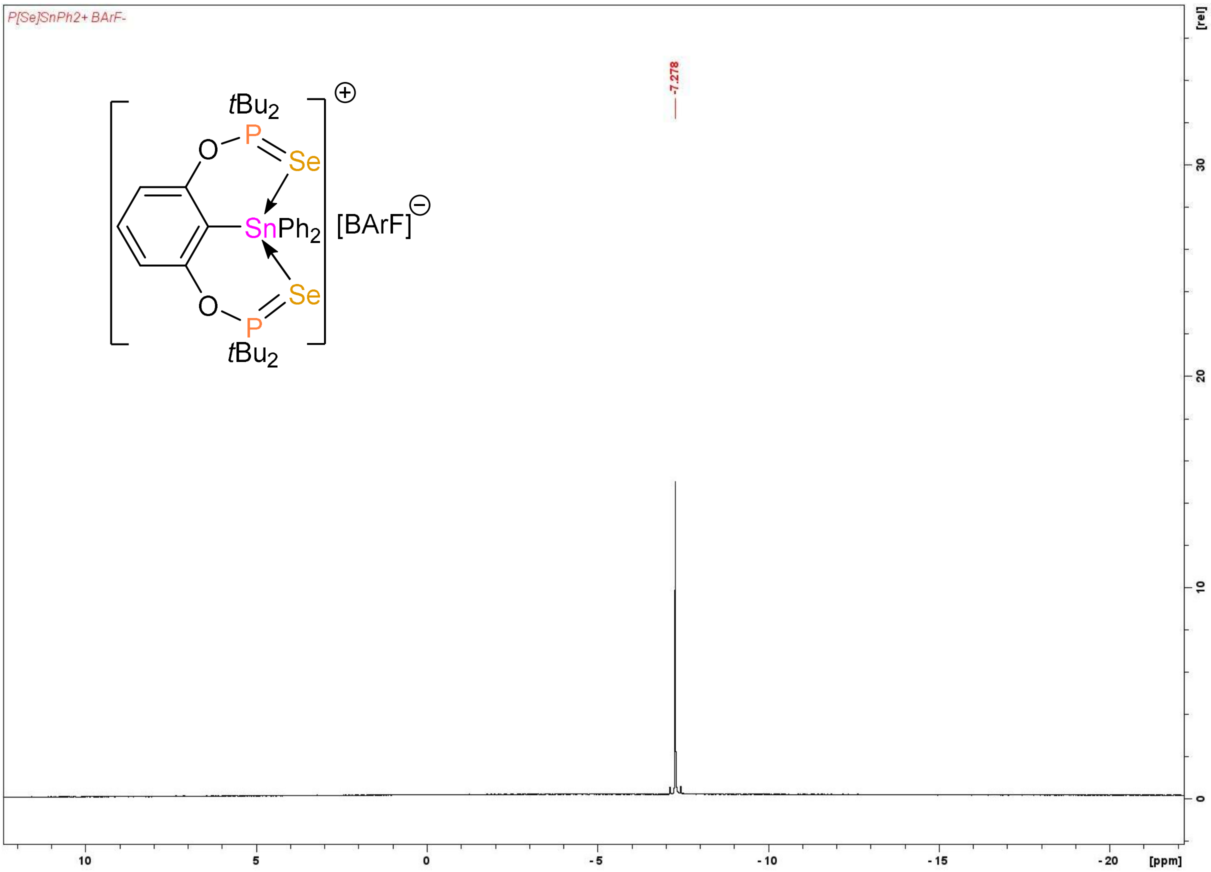

Figure S98. ^{11}B{^{1}H} NMR spectrum of **3^{Se+}[BArF]$^{-}$** (160.42 MHz MHz, CDCl$_3$).

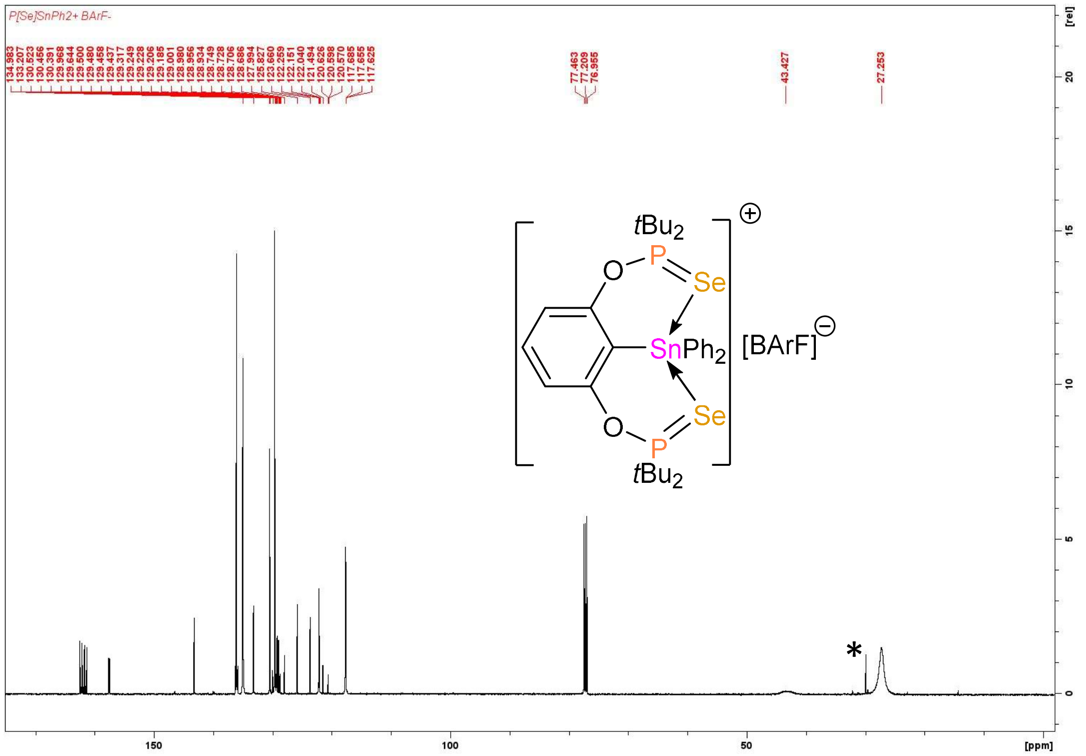

Figure S99. $^{13}C\{^1H\}$ NMR spectrum of $\mathbf{3^{Se+}[BArF]^-}$ (125.76 MHz, $CDCl_3$). *Signal of minor impurity.

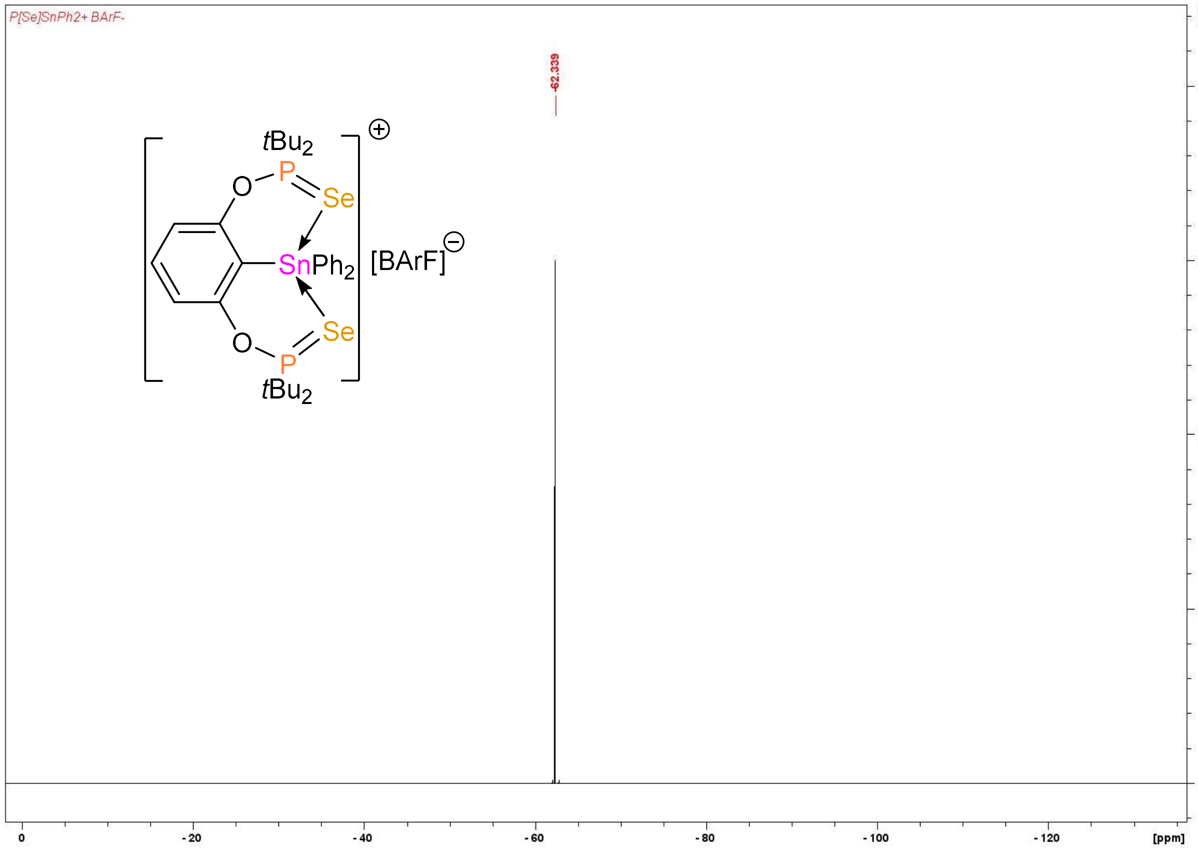

Figure S100. $^{19}F\{^1H\}$ NMR spectrum of $\mathbf{3^{Se+}[BArF]^-}$ (470.5 MHz, $CDCl_3$).

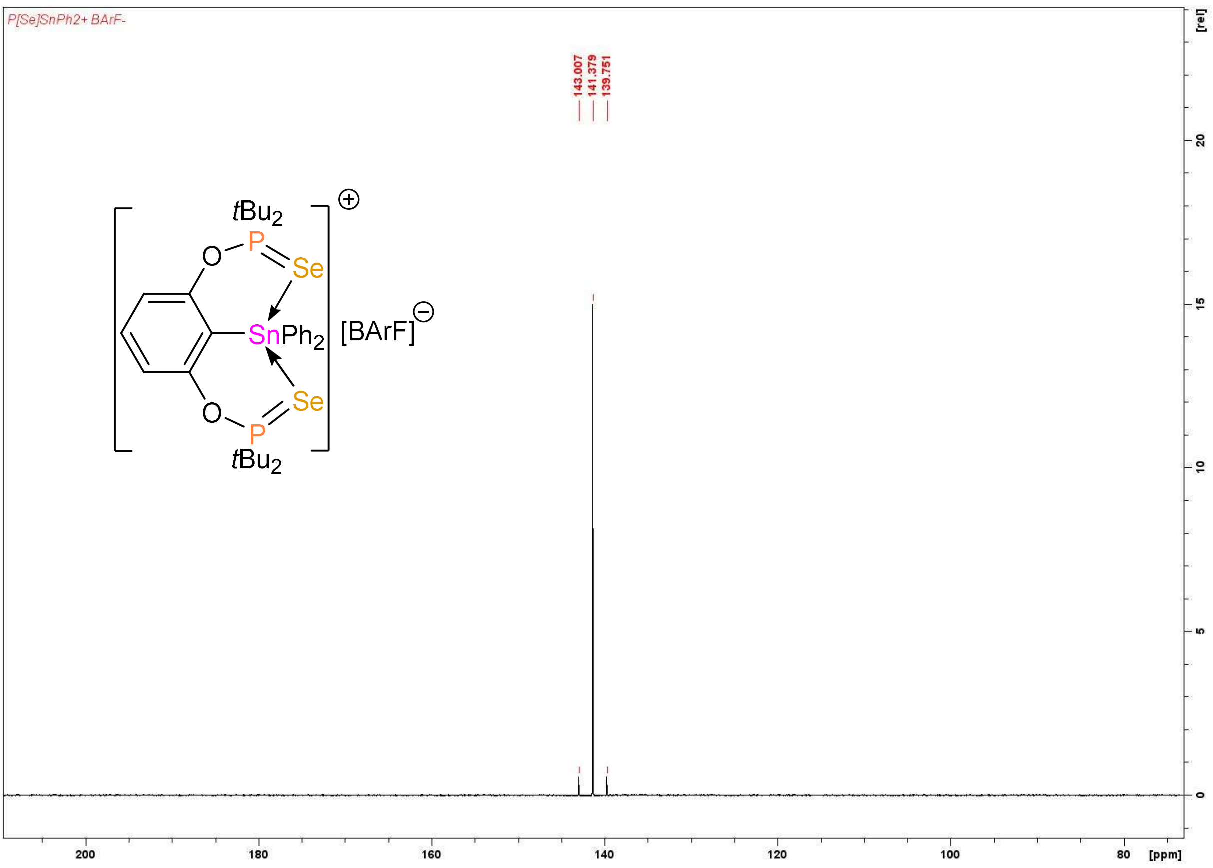

Figure S101. $^{31}P\{^1H\}$ NMR spectrum of **3^{Se+}[BArF]$^-$** (202.5 MHz MHz, $CDCl_3$).

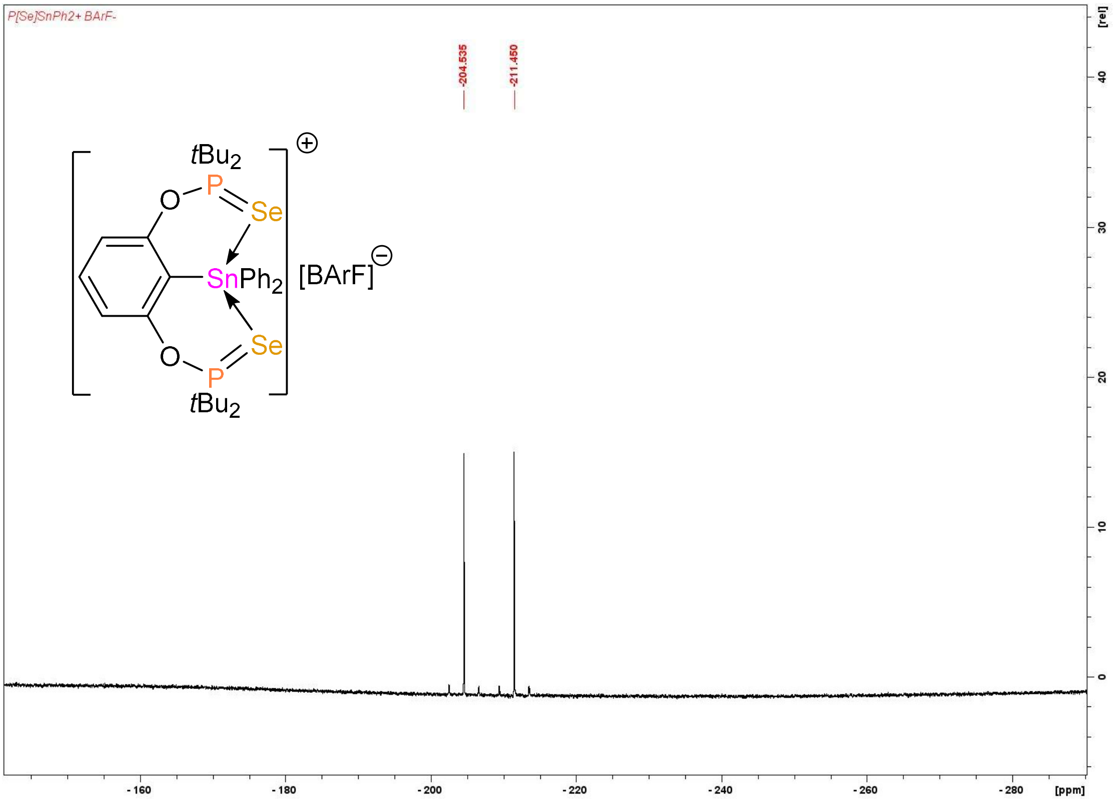

Figure S102. $^{77}Se\{^1H\}$ NMR spectrum of **3^{Se+}[BArF]$^-$** (95.4 MHz, $CDCl_3$).

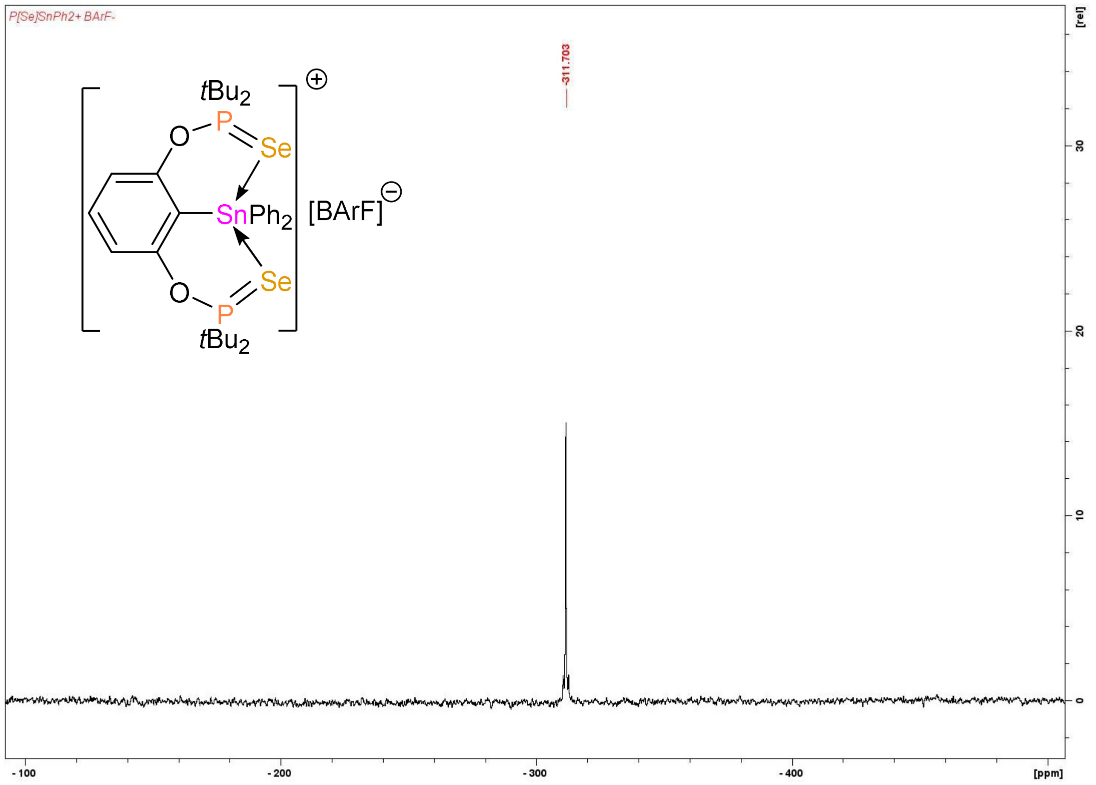

Figure S103. $^{119}Sn\{^{1}H\}$ NMR spectrum of $\mathbf{3^{Se+}[BArF]^{-}}$ (186.5MHz, $CDCl_3$).

Synthesis of [2-(tBu$_2$(O)PO)-6-(tBu$_2$PO)C$_6$H$_3$]SnPh$_2$Cl (3^{PO})

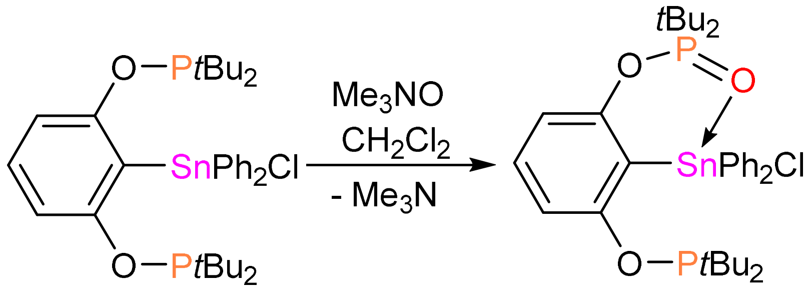

(**3**) (**3^{PO}**)

Solution of Me_3NO (0.110 g; 1.46 mmol) in dichloromethane (10 ml) was added to solution of **3** (1.121 g; 1,46 mmol) in dichloromethane (20 ml). The reaction mixture was stirred for 1 h at room temperature. Resulting colorless solution was concentrated to 1/2 of the original volume and was layered with hexane. Crystallization at room temperature gave colorless crystals of compound **3^{PO}**, whereas another batch of crystals could be obtained from mother liquor by crystallization at -30 °C. Combined yield of **3^{PO}** was 1.01 g, (88 %), m. p. 179 °C. Single-crystals suitable for *sc*-XRD diffraction analysis were obtained by slow diffusion of hexane into saturated dichloromethane solution at room temperature. Anal. Calcd for $C_{34}H_{49}ClO_3P_2Sn$ (MW 721.87): C, 56.6; H, 6.8 %. Found: C, 56.8; H, 7.1 %.**^{1}H NMR** (500 MHz, $CDCl_3$) δ (ppm): 0.97 [36H, m, *t*Bu$_2$P-C*H*$_3$ and *t*Bu$_2$(O)P-C*H*$_3$], 6.74 [1H, s(br), Ar-*H*], 7.33 [7H, m, Ar-*H*], 7.67 [1H, t, $^3J(^1H,^1H)$ = 7.0 Hz, Ar-*H*], 7.93 [4H, d, $^3J(^1H,^1H)$ = 5.5 Hz, $^2J(^{119/117}Sn, ^1H)$ = 72.7 Hz, Ar-*H*]. **$^{13}C\{^1H\}$ NMR** (125.78 MHz, $CDCl_3$) δ (ppm): 26.8 [s(br), *t*Bu$_2$(O)P-*C*H$_3$], 27.7 [d, $^2J(^{31}P,^{13}C)$ = 15.7 Hz *t*Bu$_2$P-*C*H$_3$], 35.7 [d, $^1J(^{31}P,^{13}C)$ = 26.2 Hz, *t*Bu$_2$P-*C*], 37.2 [d, $^1J(^{31}P,^{13}C)$ = 75.6 Hz, *t*Bu$_2$(O)P-*C*], 113.4 [s(br), Ar-*C*], 115.4 [d(br), $^nJ(^{31}P,^{13}C)$ = 29.4 Hz, Ar-*C*], 128.3 [s, $^3J(^{119/117}Sn,^{13}C)$ = 76.5 Hz, Ar-*C*], 128.9 [s, Ar-*C*], 131.4 [s, Ar-*C*], 136.8 [s, $^3J(^{119/117}Sn,^{13}C)$ = 53.4 Hz, Ar-*C*], 145.1 [s(br), Ar-*C*], 157.9 [s(br), Ar-*C*], 165.9 [d, $^2J(^{31}P,^{13}C)$ = 10.8 Hz, Ar-C]. **$^{31}P\{^1H\}$ NMR** (202.5 MHz, $CDCl_3$) δ (ppm): 77.1 [s, *t*Bu$_2$(O)*P*], 159.2 [s, *t*Bu$_2$*P*]. **$^{119}Sn\{^1H\}$ NMR** (186.5 MHz, $CDCl_3$) δ (ppm): -246.9 [s].

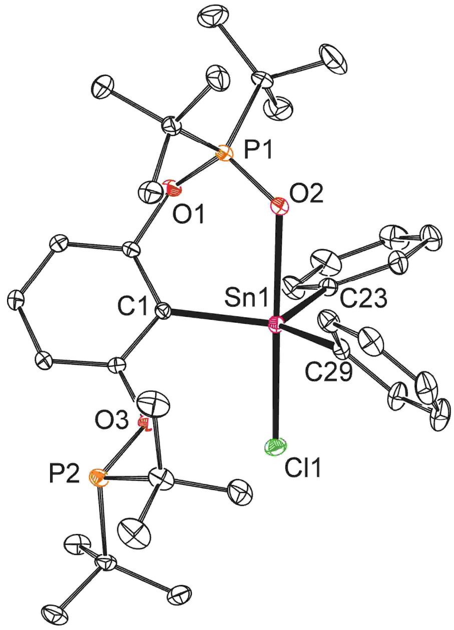

Figure S104. Molecular structure of $\mathbf{3^{PO}}$. ORTEP with 30% probability ellipsoid level. Hydrogen atoms and dichloromethane solvate molecule are omitted. Selected structural bond lengths [Å]: Sn(1)-C(1) 2.1447(13), Sn(1)-C(23) 2.1263(15), Sn(1)-C(29) 2.1263(15), Sn(1)-O(2) 2.3470(10), Sn(1)-P(2) 4.8636(5); bonding angles [°]: O(2)-Sn(1)-Cl(1) 176.74(3), C(1)-Sn(1)-C(23) 115.67(6), C(1)-Sn(1)-C(29) 125.59(6), C(23)-Sn(1)-C(29) 117.25(6).

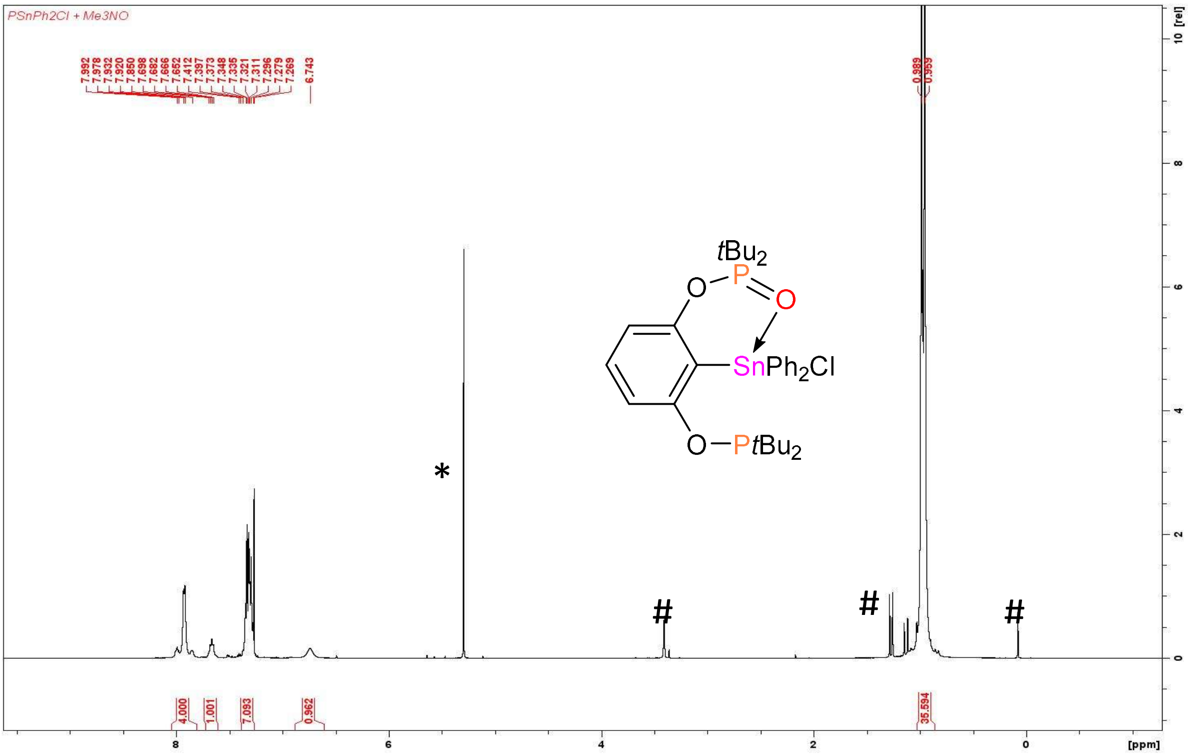

Figure S105. ^{1}H NMR spectrum of $\mathbf{3^{PO}}$ (500 MHz, $CDCl_3$). *Signal of co-crystallized dichloromethane, #signal of unknown impurities and silicon grease.

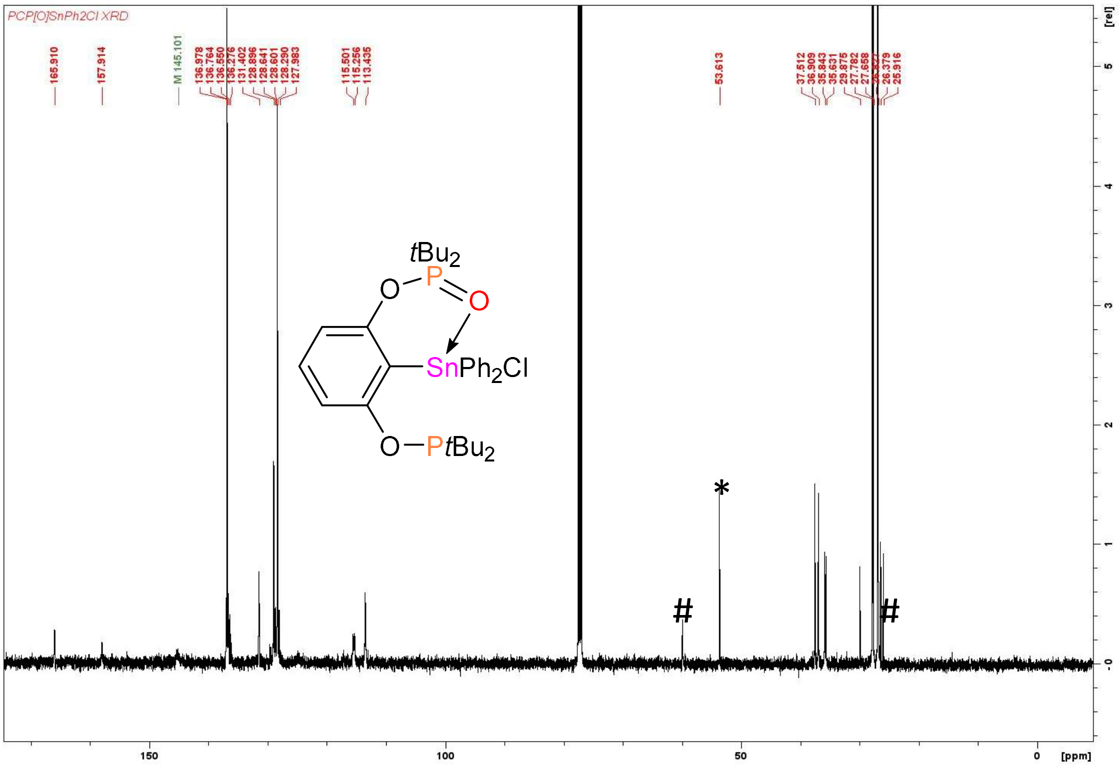

Figure S106. $^{13}C\{^{1}H\}$ NMR spectrum of $\mathbf{3^{PO}}$ (125.76 MHz, $CDCl_3$). *Signal of co-crystallized dichloromethane, #signal of unknown impurities.

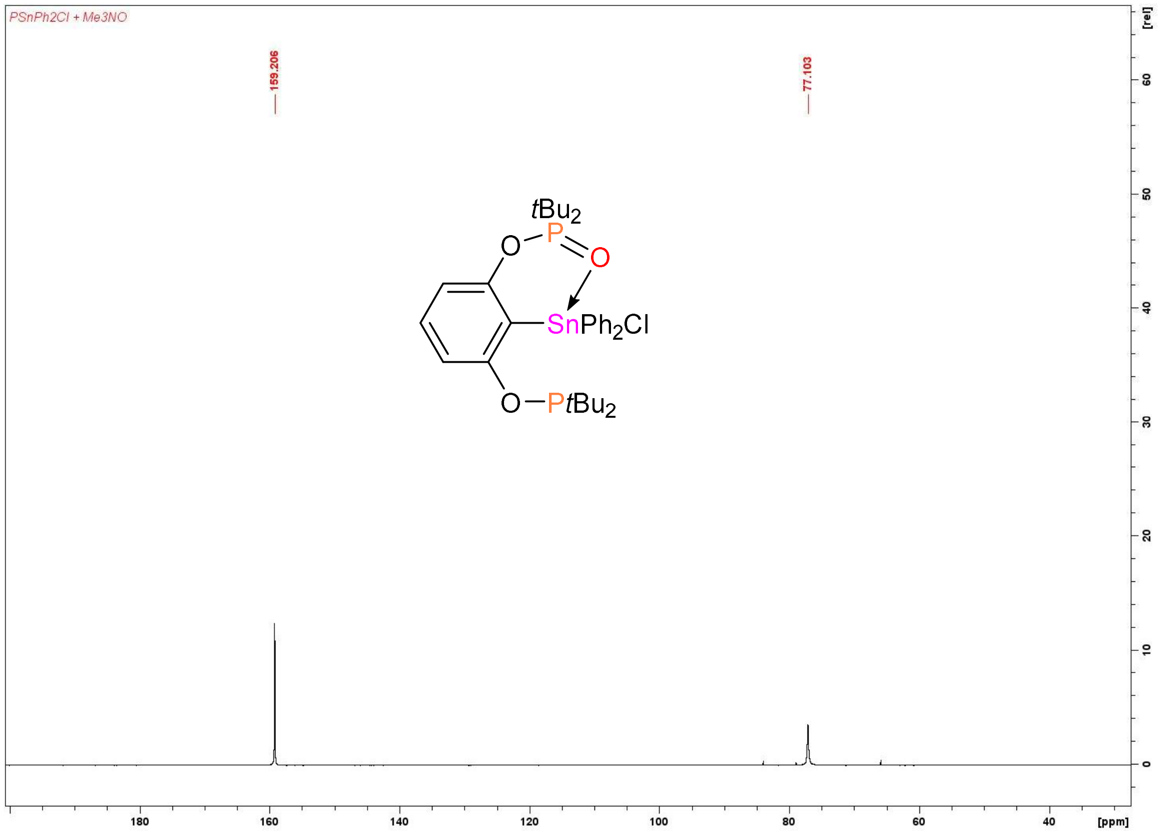

Figure S107. $^{31}P\{^{1}H\}$ NMR spectrum of $\mathbf{3^{PO}}$ (202.5 MHz MHz, $CDCl_3$).

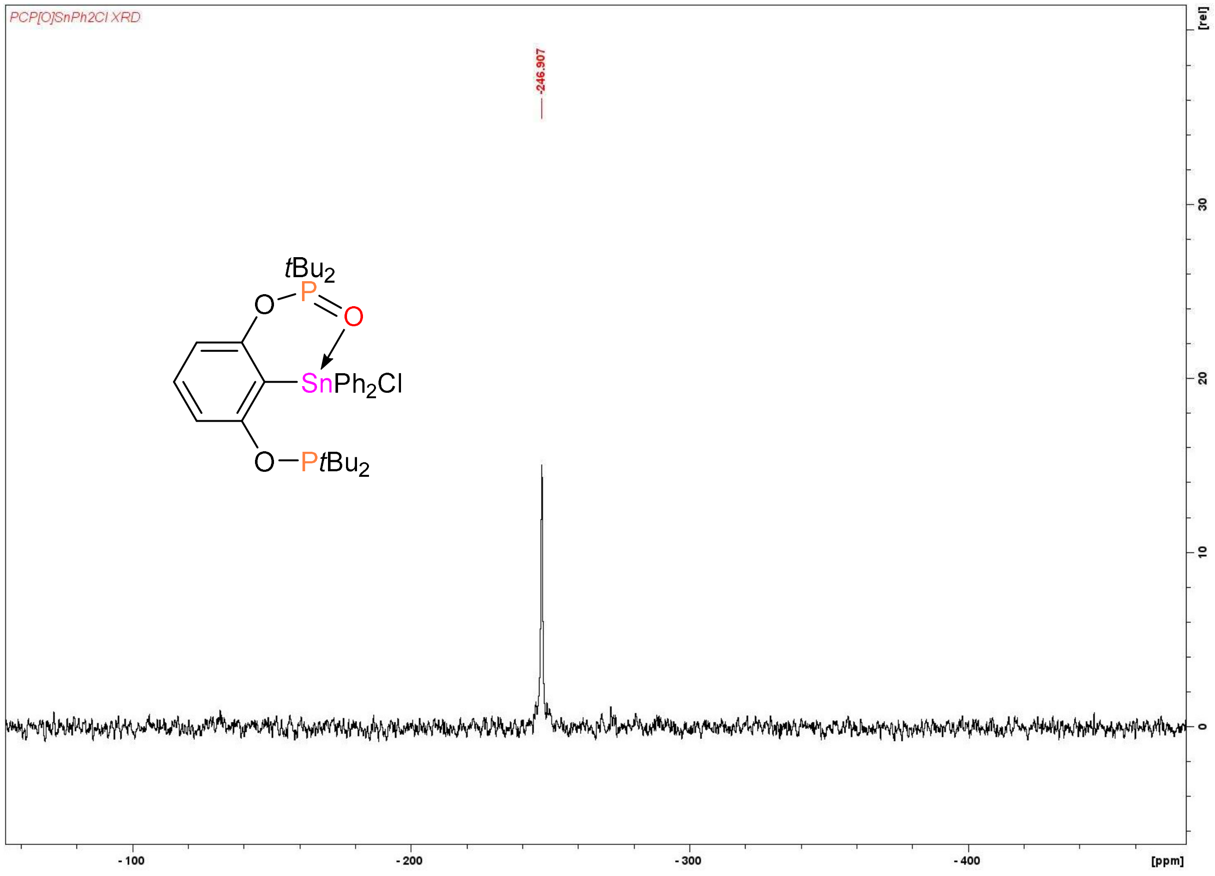

Figure S108. $^{119}Sn\{^{1}H\}$ NMR spectrum of $\mathbf{3^{PO}}$ (186.5MHz, $CDCl_3$).

Synthesis of [2-(*t*Bu2(O)PO)-6-(*t*Bu2(S)PO)C6H3]SnPh2Cl (3OS)

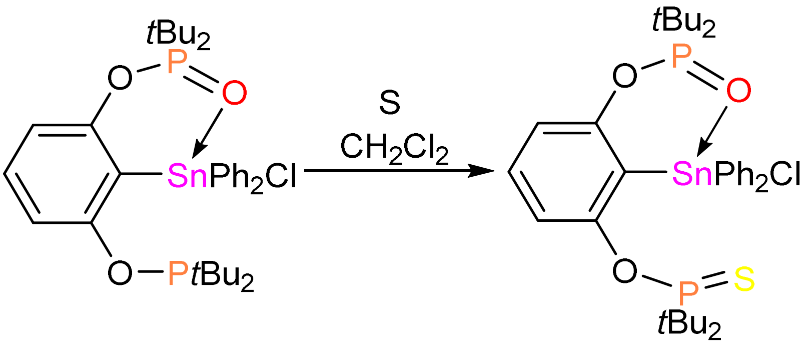

(3^{PO}) (3OS)

Elemental sulfur (6.5 mg; 0.2 mmol) was added in one portion to solution of $\mathbf{3^{PO}}$ (158 mg; 0.2 mmol) in dichloromethane (10 ml). The reaction mixture was stirred for 24 h at room temperature and then was concentrated to 1/2 of the original volume. Colorless solution was layered with hexane. Crystallization at room temperature gave colorless crystals of compound $\mathbf{3^{OS}}$, whereas another batch of crystals could be obtained from mother liquor by crystallization at -30 °C. Combined yield of $\mathbf{3^{OS}}$ was 150 mg, (91 %), m. p. 302-304 °C. Anal. Calcd for $C_{34}H_{49}ClO_3P_2SSn$ (MW 753.93): C, 54.2; H, 6.6 %. Found: C, 54.0; H, 6.8 %. **^{1}H NMR** (500 MHz, $CDCl_3$) δ (ppm): 0.80 [9H, d(br), $^{3}J(^{31}P,^{1}H)$ = 11.3 Hz, *t*Bu$_2$P(O/S)-C*H*$_3$], 0.96 [9H, d(br), $^{3}J(^{31}P,^{1}H)$ = 13.9 Hz, *t*Bu$_2$P(O/S)-C*H*$_3$], 1.13 [9H, d(br), $^{3}J(^{31}P,^{1}H)$ = 12.0 Hz, *t*Bu$_2$P(O/S)-C*H*$_3$], 1.46 [9H, d(br), $^{3}J(^{31}P,^{1}H)$ = 13.9 Hz, *t*Bu$_2$P(O/S)-C*H*$_3$], 6.90 [1H, d, $^{3}J(^{1}H,^{1}H)$ = 8.0 Hz, Ar-*H*], 7.25 [3H, m(br), Ar-*H*], 7.36 [1H, t, $^{3}J(^{1}H,^{1}H)$ = 8.1 Hz, Ar-*H*], 7.46 [5H, s(br), Ar-*H*], 7.97 [1H, d, $^{3}J(^{1}H,^{1}H)$ = 8.4 Hz, Ar-*H*], 8.23 [2H, s(br), Ar-*H*]. **^{13}C{^{1}H} NMR**: Reasonable ^{13}C NMR spectrum could not be obtained due to a significant line broadening even at low temperature. **^{31}P{^{1}H} NMR** (202.5 MHz, $CDCl_3$) δ (ppm): 78.9 [s, *t*Bu$_2$*P*(O)], 132.1 [s, *t*Bu$_2$*P*(S)]. **^{119}Sn{^{1}H} NMR** (186.5 MHz, $CDCl_3$) δ (ppm): -258.2 [s].

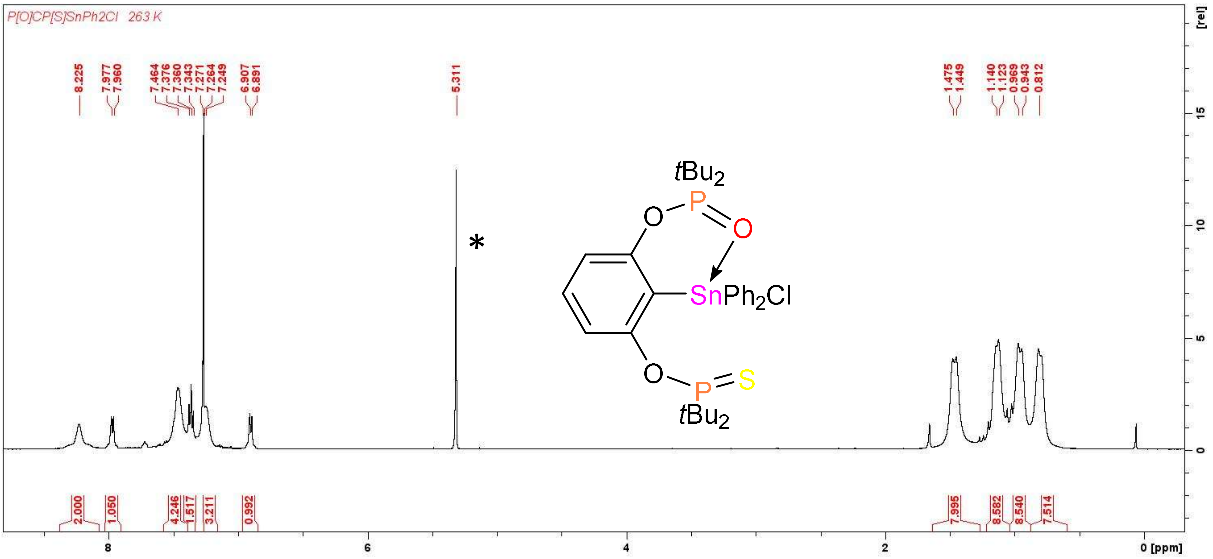

Figure S109. ^{1}H NMR spectrum of $\mathbf{3^{OS}}$ (500 MHz, $CDCl_3$). Measured at 263 K. *Signal of co-crystallized dichloromethane.

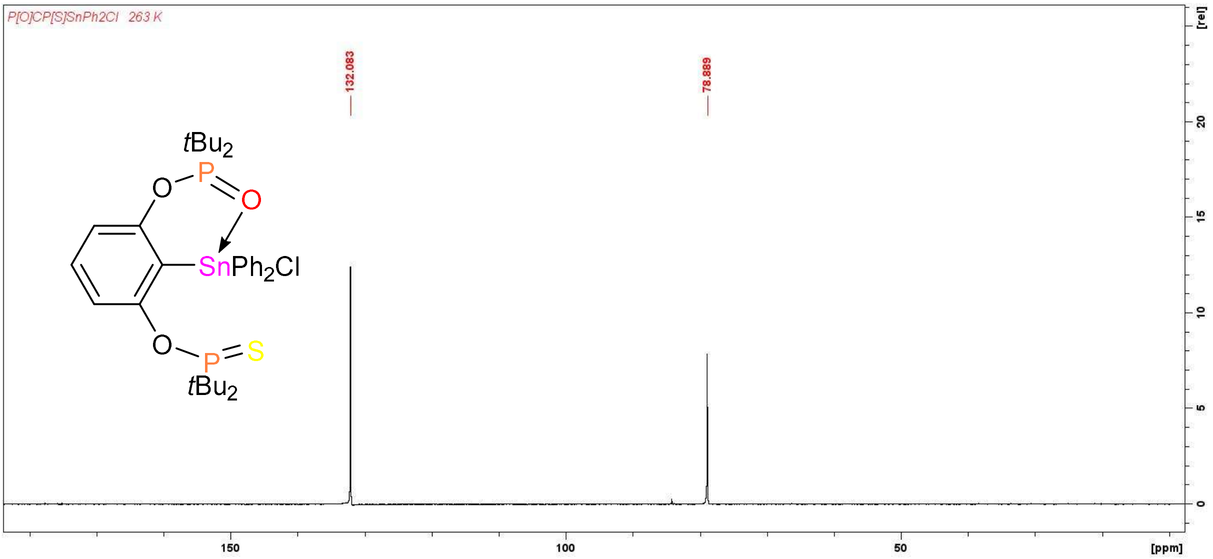

Figure S110. $^{31}P\{^1H\}$ NMR spectrum of $\mathbf{3^{OS}}$ (202.5 MHz MHz, $CDCl_3$). Measured at 263 K.

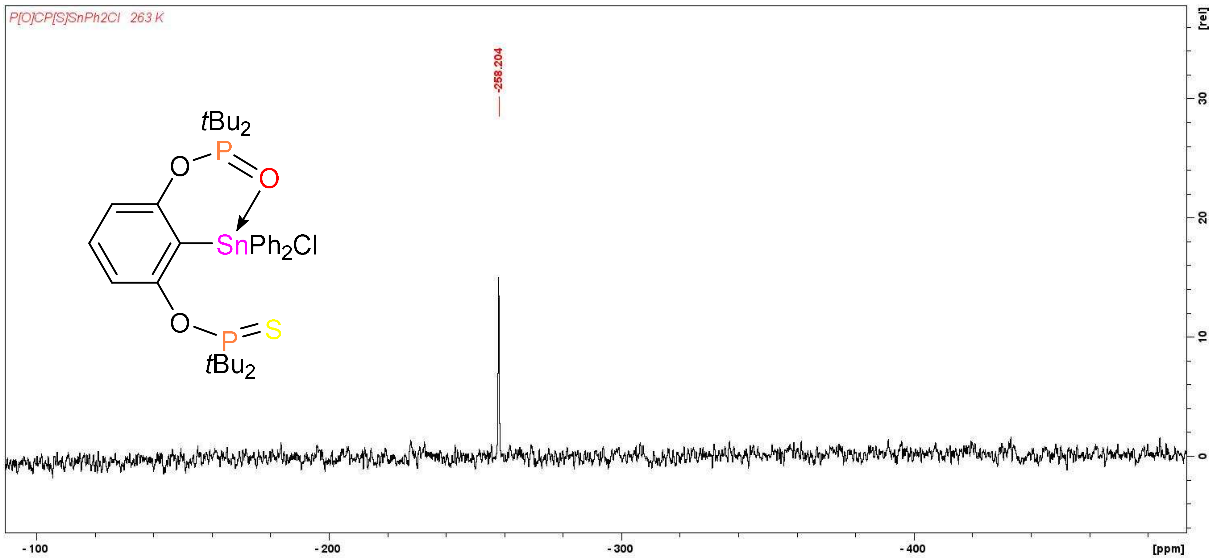

Figure S111. $^{119}Sn\{^{1}H\}$ NMR spectrum of **3**^{OS} (186.5MHz, $CDCl_3$). Measured at 263 K.

Synthesis of [2-(*t*Bu$_2$(O)PO)-6-(*t*Bu$_2$(Se)PO)C_6H_3]SnPh$_2$Cl (3^{OSe})

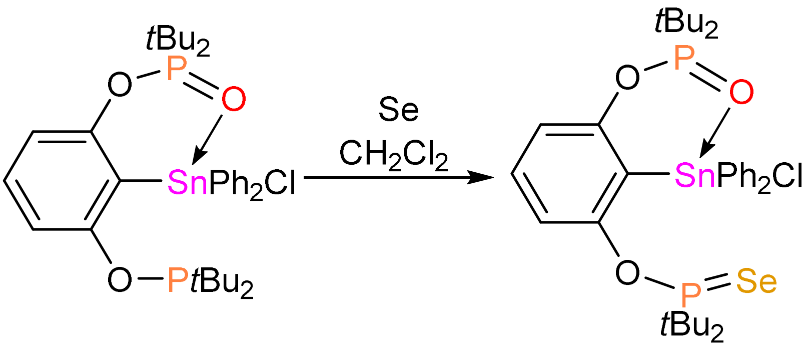

Elemental selenium (85 mg; 1.08 mmol) was added in one portion to solution of **3**^{PO} (282 mg; 0.36 mmol) in dichloromethane (20 ml). The reaction mixture was stirred for 24 h at room temperature and then unreacted selenium was removed by filtration. Resulting colorless solution was concentrated to 1/2 of the original volume and was layered with hexane. Crystallization at room temperature gave colorless crystals of compound **3**^{OSe}, and another batch of crystals could be obtained from mother liquor by crystallization at -30 °C. Combined yield of **3**^{OSe} was 271 mg, (70 %), m. p. 264 °C. Single-crystals suitable for *sc*-XRD diffraction analysis were obtained from saturated solution using dichloromethane/hexane mixture at 5 °C. Anal. Calcd for $C_{34}H_{49}ClO_3P_2SeSn$ (MW 800.84): C, 51.0; H, 6.2 %. Found: C, 50.8; H,

6.2 %.**^{1}H NMR** (500 MHz, $CDCl_3$) δ (ppm): 0.80 [9H, d(br), $^{3}J(^{31}P,^{1}H)$ = 14.6 Hz, *t*Bu_2P(O/Se)-C*H*$_3$], 0.99 [9H, d(br), $^{3}J(^{31}P,^{1}H)$ = 16.9 Hz, *t*Bu_2P(O/Se)-C*H*$_3$], 1.13 [9H, d(br), $^{3}J(^{31}P,^{1}H)$ = 14.6 Hz, *t*Bu_2P(O/Se)-C*H*$_3$], 1.49 [9H, d(br), $^{3}J(^{31}P,^{1}H)$ = 16.4 Hz, *t*Bu_2P(O/Se)-C*H*$_3$], 6.92 [1H, d, $^{3}J(^{1}H,^{1}H)$ = 8.0 Hz, Ar-*H*], 7.24 [3H, m(br), Ar-*H*], 7.38 [1H, t, $^{3}J(^{1}H,^{1}H)$ = 8.1 Hz, Ar-*H*], 7.46 [5H, s(br), Ar-*H*], 7.99 [1H, d, $^{3}J(^{1}H,^{1}H)$ = 8.5 Hz, Ar-*H*], 8.23 [2H, s(br), $^{2}J(^{119/117}Sn,^{1}H)$ = 74.4 Hz, Ar-*H*]. **^{13}C{^{1}H} NMR**: Reasonable ^{13}C NMR spectrum could not be obtained due to a significant line broadening even at low temperature. **^{31}P{^{1}H} NMR** (202.5 MHz, $CDCl_3$) δ (ppm): 79.1 [s, *t*Bu_2*P*(O)], 141.8 [s, $^{1}J(^{77}Se,^{31}P)$ = 780 Hz, *t*Bu_2*P*(Se)]. **^{77}Se{^{1}H} NMR** (95.4 MHz, $CDCl_3$) δ (ppm): -320.9 [d, $^{1}J(^{77}Se,^{31}P)$ = 780 Hz] measured at 263K at r.t. the signal was too broad to be detected. **^{119}Sn{^{1}H} NMR** (186.5 MHz, $CDCl_3$) δ (ppm): -258.9 [s].

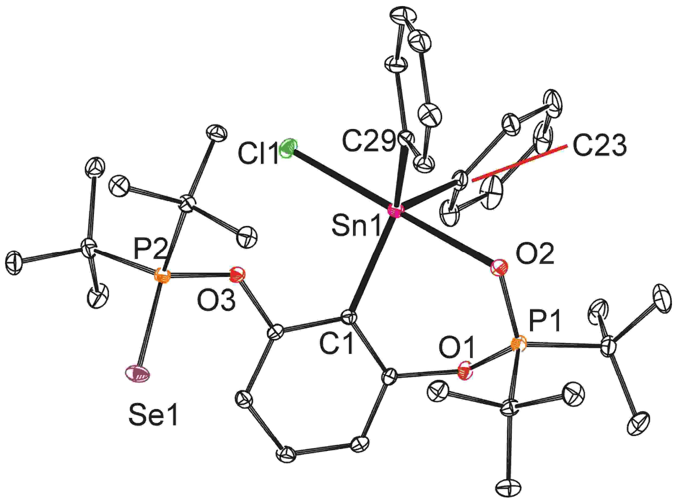

Figure S112. Molecular structure of **3^{OSe}**. ORTEP with 30% probability ellipsoid level. Only one of two independent molecules is presented. Hydrogen atoms and dichloromethane solvate molecules are omitted. Selected structural bond lengths [Å]: Sn(1)-C(1) 2.1437(19), Sn(1)-Cl(1) 2.4944(6), Sn(1)-C(23) 2.125(2), Sn(1)-C(29) 2.125(2), Sn(1)-O(2) 2.3558(14), Sn(1)-Se(1) 6.2614(5); bonding angles [°]: O(2)-Sn(1)-Cl(1) 178.51(4), C(1)-Sn(1)-C(23) 126.34(8), C(1)-Sn(1)-C(29) 117.30(8), C(23)-Sn(1)-C(29) 114.81(8). Selected structural bond lengths for the second independent molecule [Å]: Sn(2)-C(101) 2.143(2), Sn(2)-Cl(2) 2.4968(6), Sn(2)-

C(123) 2.123(2), Sn(2)-C(129) 2.123(2), Sn(2)-O(5) 2.3489(15), Sn(2)-Se(2) 6.2824(5); bonding angles [°]: O(5)-Sn(2)-Cl(2) 177.19(4), C(101)-Sn(2)-C(123) 126.97(8), C(101)-Sn(2)-C(129) 117.61(8).

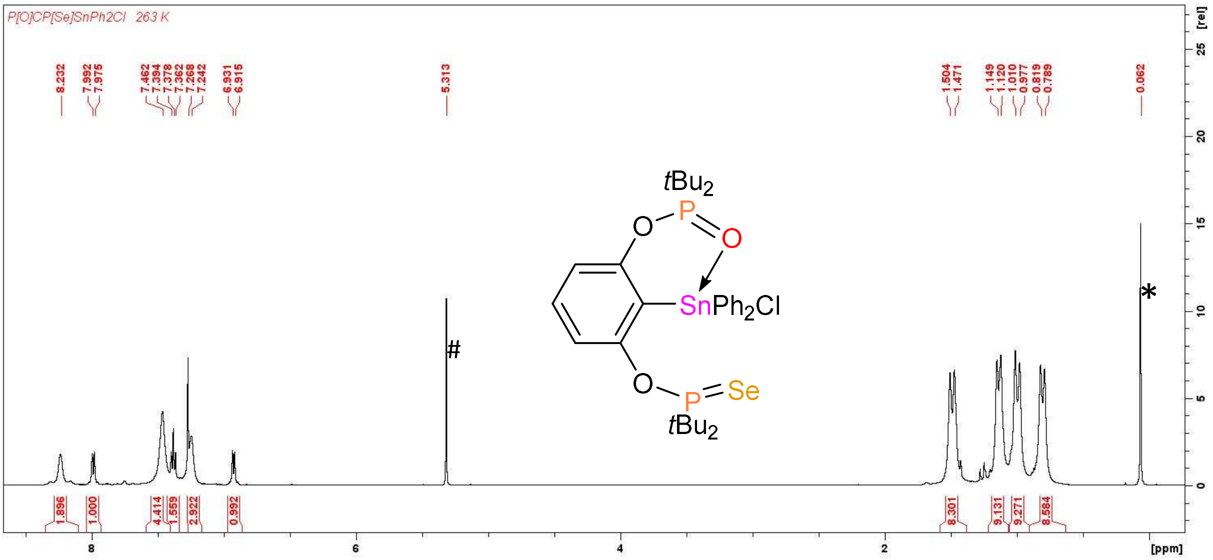

Figure S113. ^{1}H NMR spectrum of $\mathbf{3^{OSe}}$ (500 MHz, $CDCl_3$). Measured at 263 K. *Signal of silicon grease, #signal of co-crystallized dichloromethane.

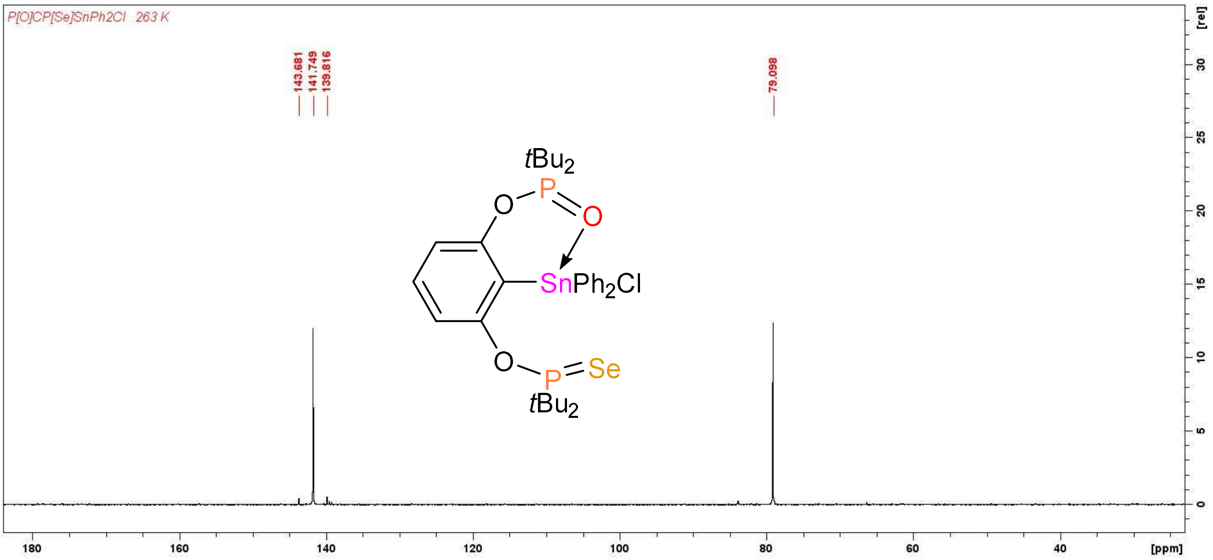

Figure S114. ^{31}P{^{1}H} NMR spectrum of $\mathbf{3^{OSe}}$ (202.5 MHz MHz, $CDCl_3$). Measured at 263 K.

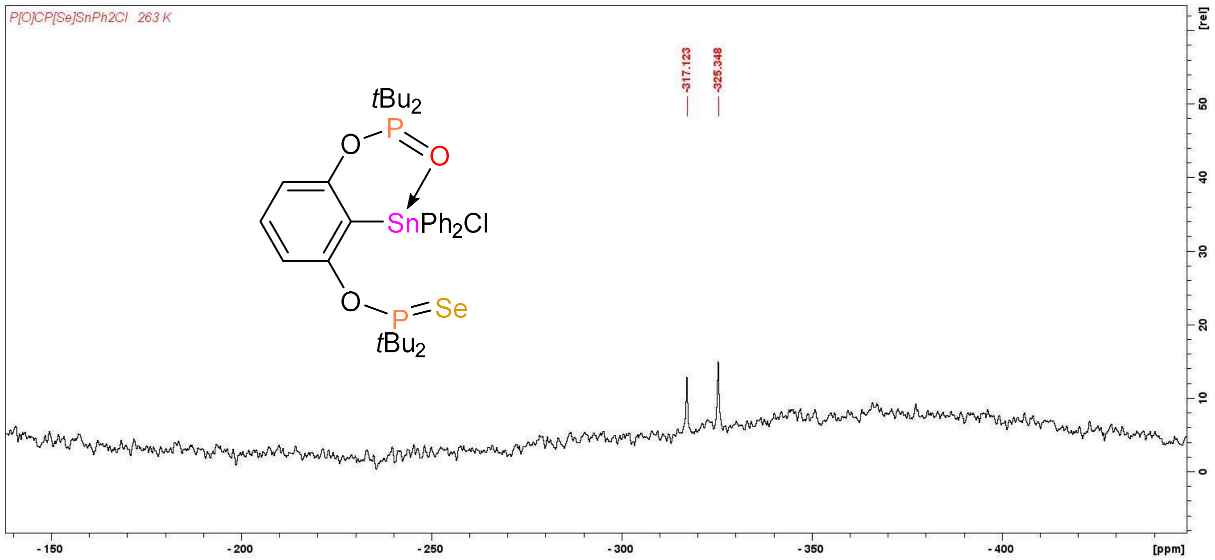

Figure S115. $^{77}Se\{^{1}H\}$ NMR spectrum of **3^{OSe}** (95.4 MHz, $CDCl_3$). Measured at 263 K.

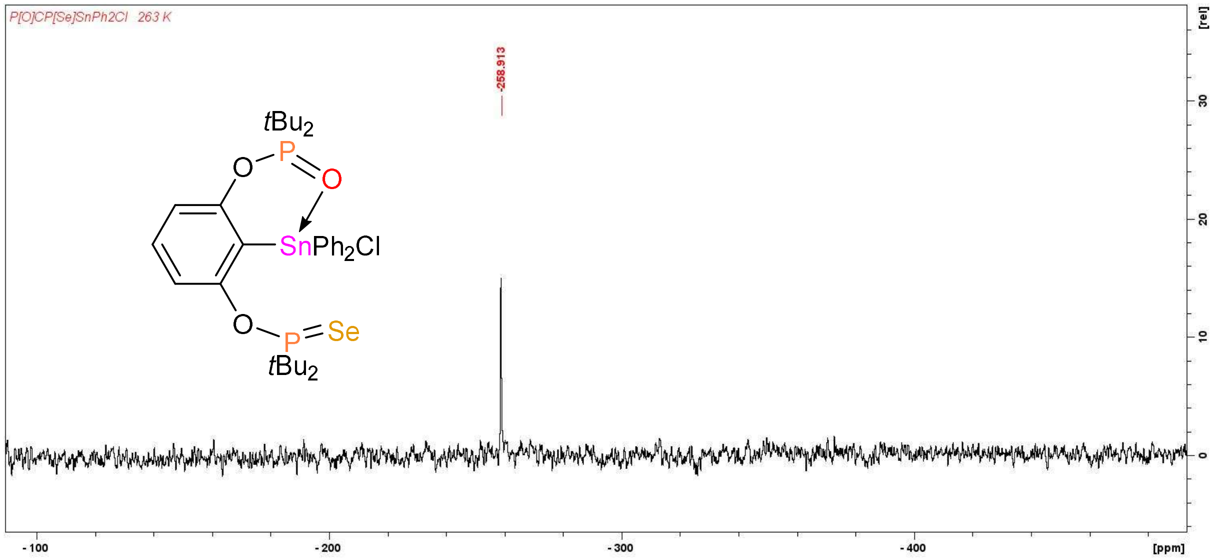

Figure S116. $^{119}Sn\{^{1}H\}$ NMR spectrum of **3^{OSe}** (186.5MHz, $CDCl_3$). Measured at 263 K.

Synthesis of {[2-(*t*Bu$_2$(O)PO)-6-(*t*Bu$_2$PO)C_6H_3]SnPh$_2$}{[B[3,5-(CF_3)$_2$$C_6H_3$]$_4$} ($3^{PO+}$[BArF]$^-$)

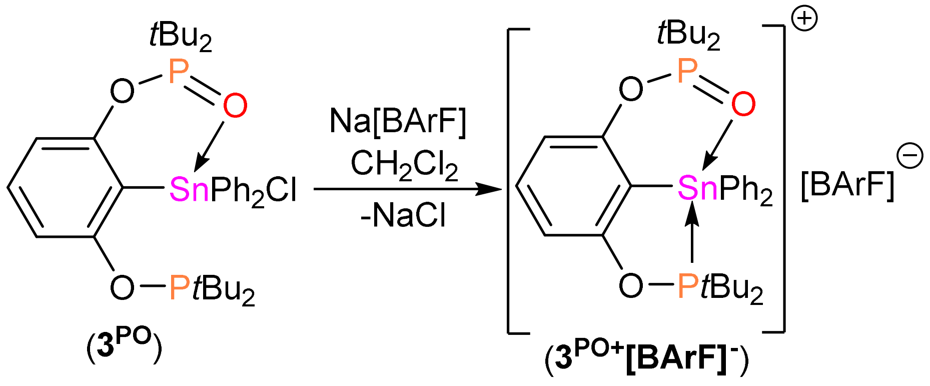

Solid Na[BArF] (226 mg; 0.26 mmol) was added in one portion to solution of $\mathbf{3^{PO}}$ (200 mg; 0.26 mmol) in dichloromethane (10 ml). The reaction mixture was stirred for 30 minutes at room temperature and then incipient NaCl was removed by filtration. Colorless solution was concentrated to 1/2 of the original volume and layered with hexane. Crystallization at room temperature gave colorless crystals of compound $\mathbf{3^{PO+}[BArF]^-}$. Yield of $\mathbf{3^{PO+}[BArF]^-}$ was 353 mg, (86 %), m. p. 189-191 °C. Single-crystals suitable for *sc*-XRD diffraction analysis were obtained by slow diffusion of hexane into saturated dichloromethane solution at room temperature. Anal. Calcd for $C_{66}H_{61}BF_{24}O_3P_2Sn$ (MW 1549.64): C, 51.2; H, 4.0 %. Found: C, 51.6; H, 4.3 %. **^{1}H NMR** (500 MHz, $CDCl_3$) δ (ppm): 1.07 [18H, d, $^3J(^{31}P,^1H)$ = 14.8 Hz, *t*Bu$_2$P-C*H*$_3$], 1.16 [18H, d, $^3J(^{31}P,^1H)$ = 16.0 Hz, *t*Bu$_2$(O)P-C*H*$_3$], 6.92 [1H, d, $^3J(^1H,^1H)$ = 8.1 Hz, Ar-*H*], 7.10 [1H, d, $^3J(^1H,^1H)$ = 8.3 Hz, Ar-*H*], 7.45 [1H, t, $^3J(^1H,^1H)$ = 8.2 Hz, Ar-*H*], 7.50 [6H, m, Ar-*H*], 7.53 [4H, m, Ar-*H*], 7.67 [4H, m, $^3J(^{119/117}Sn, ^1H)$ = 76.6 Hz, Ar-*H*], 7.73 [8H, m, Ar-*H*]. **^{11}B{^{1}H} NMR** (160.42 MHz, $CDCl_3$) δ (ppm): -7.2 [s]. **^{13}C{^{1}H} NMR** (125.78 MHz, $CDCl_3$) δ (ppm): 26.1 [s, *t*Bu$_2$(O)P-*C*H$_3$], 27.1 [d, $^2J(^{31}P,^{13}C)$ = 6.2 Hz *t*Bu$_2$P-*C*H$_3$], 37.2 [d, $^1J(^{31}P,^{13}C)$ = 72.7 Hz, *t*Bu$_2$(O)P-*C*], 39.2 [s, $^nJ(^{119/117}Sn,^{13}C)$ = 14.4 Hz, *t*Bu$_2$P-*C*], 115.2 [d, $^nJ(^{31}P,^{13}C)$ = 55.6 Hz, Ar-*C*], 116.1 [m, Ar-*C*], 116.8 [d, $^nJ(^{31}P,^{13}C)$ = 3.4 Hz, $^nJ(^{119/117}Sn,^{13}C)$ = 32.6 Hz, Ar-*C*], 117.7 [m, Ar-*C*], 124.7 [q, $^1J(^{19}F,^{13}C)$ = 273 Hz, *C*F$_3$], 129.2

[qq, $^{2}J(^{19}F,^{13}C)$ = 31.5 Hz, $^{4}J(^{19}F,^{13}C)$ = 2.8 Hz, Ar-*C*], 130.0 [s, $^{3}J(^{119/117}Sn,^{13}C)$ = 78.1 Hz, Ar-*C*], 131.5 [s, $^{4}J(^{119/117}Sn,^{13}C)$ = 15.8 Hz, Ar-*C*], 134.9 [s, Ar-*C*], 135.0 [s, Ar-*C*], 136.1 [s, $^{2}J(^{119/117}Sn,^{13}C)$ = 51.7 Hz Ar-*C*], 139.2 [d, $^{n}J(^{31}P,^{13}C)$ = 1.7 Hz, Ar-*C*], 139.5 [d, $^{n}J(^{31}P,^{13}C)$ = 1.8 Hz, Ar-*C*] 159.8 [m, Ar-*C*], 161.9 [q, $^{1}J(^{13}C,^{11}B)$ = 50 Hz, Ar-*C*]. **^{19}F{^{1}H} NMR** (376.3 MHz, $CDCl_3$) δ (ppm): -62.4 [s]. **^{31}P{^{1}H} NMR** (202.5 MHz, $CDCl_3$) δ (ppm): 84.4 [s, $^{n}J(^{119/117}Sn,^{31}P)$ = 48.2 Hz, *t*Bu_2(O)*P*], 101.8 [s, $^{n}J(^{119/117}Sn,^{31}P)$ = 960/920 Hz, *t*Bu_2*P*]. **^{119}Sn{^{1}H} NMR** (186.5 MHz, $CDCl_3$) δ (ppm): -255.2 [dd, $^{n}J(^{119/117}Sn,^{31}P)$ = 960/920 Hz].

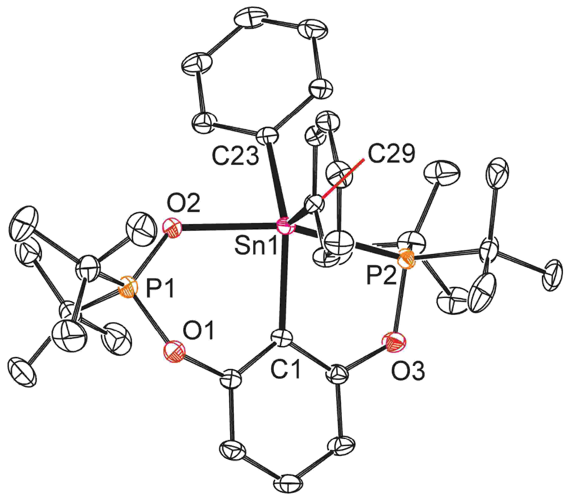

Figure S117. Molecular structure of **3^{PO+}[BArF]$^{-}$**. ORTEP with 30% probability ellipsoid level. Hydrogen atoms and [BArF] anion are omitted. Sn(1)-C(1) 2.171(2), Sn(1)-C(23) 2.133(2), Sn(1)-C(29) 2.118(2), Sn(1)-O(2) 2.2161(18), Sn(1)-P(2) 2.7298(8); bonding angles [°]: O(2)-Sn(1)-P(2) 154.59(5), C(1)-Sn(1)-C(23) 140.43(8), C(1)-Sn(1)-C(29) 105.83(8), C(23)-Sn(1)-C(29) 113.70(8).

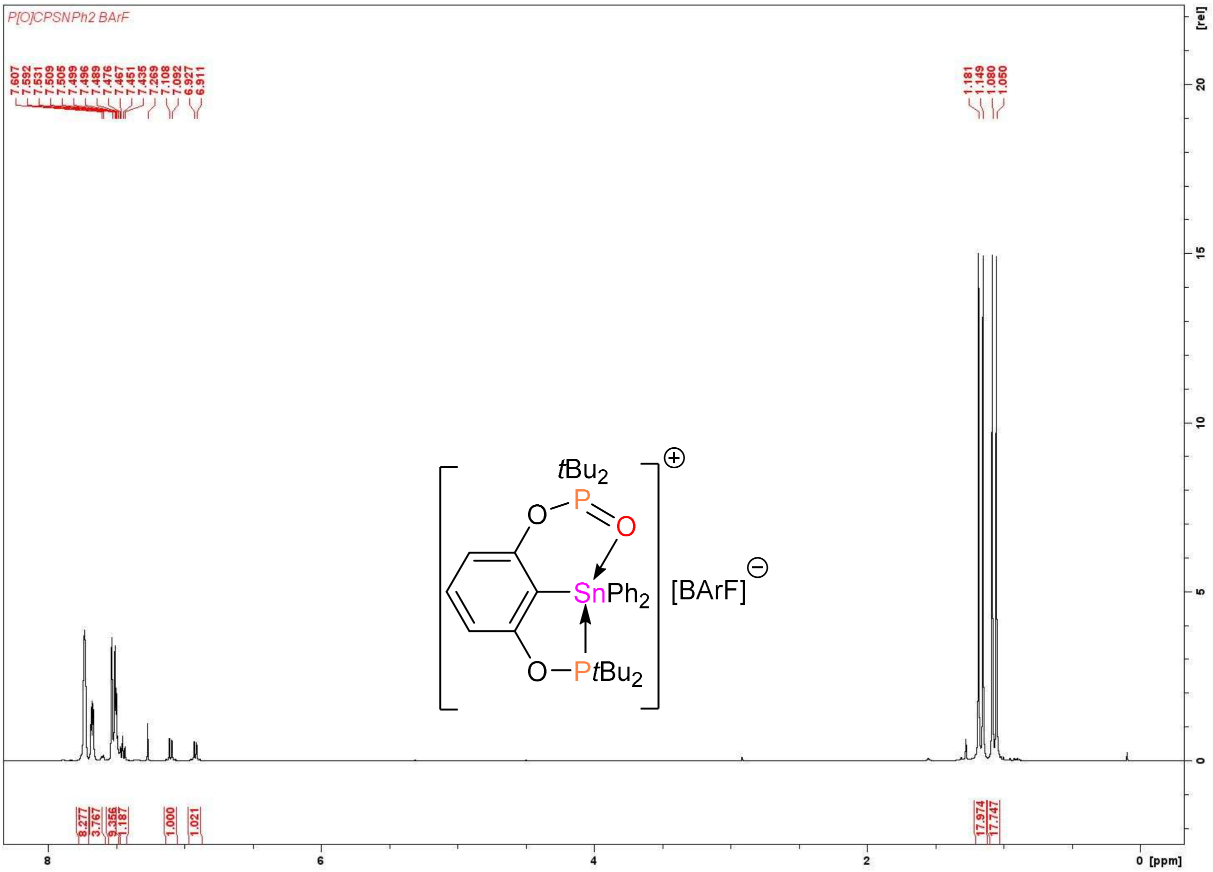

Figure S118. ^{1}H NMR spectrum of $\mathbf{3^{PO+}[BArF]^-}$ (500 MHz, $CDCl_3$).

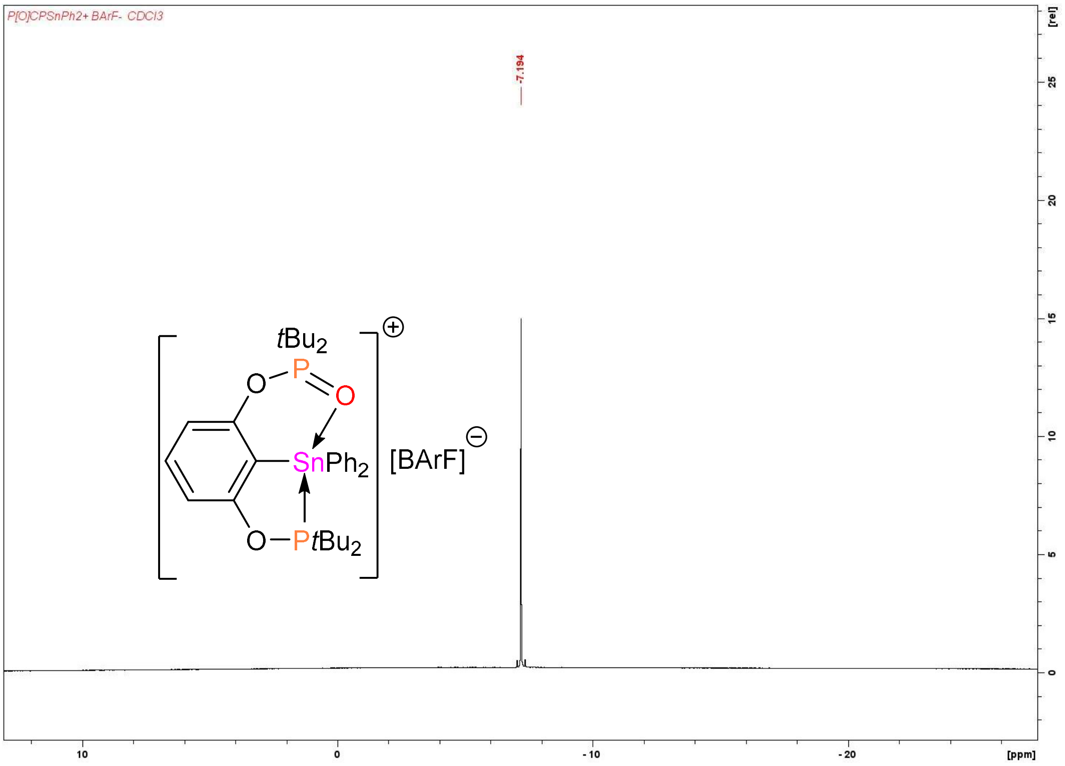

Figure S119. ^{11}B{^{1}H} NMR spectrum of $\mathbf{3^{PO+}[BArF]^-}$ (160.42 MHz MHz, $CDCl_3$).

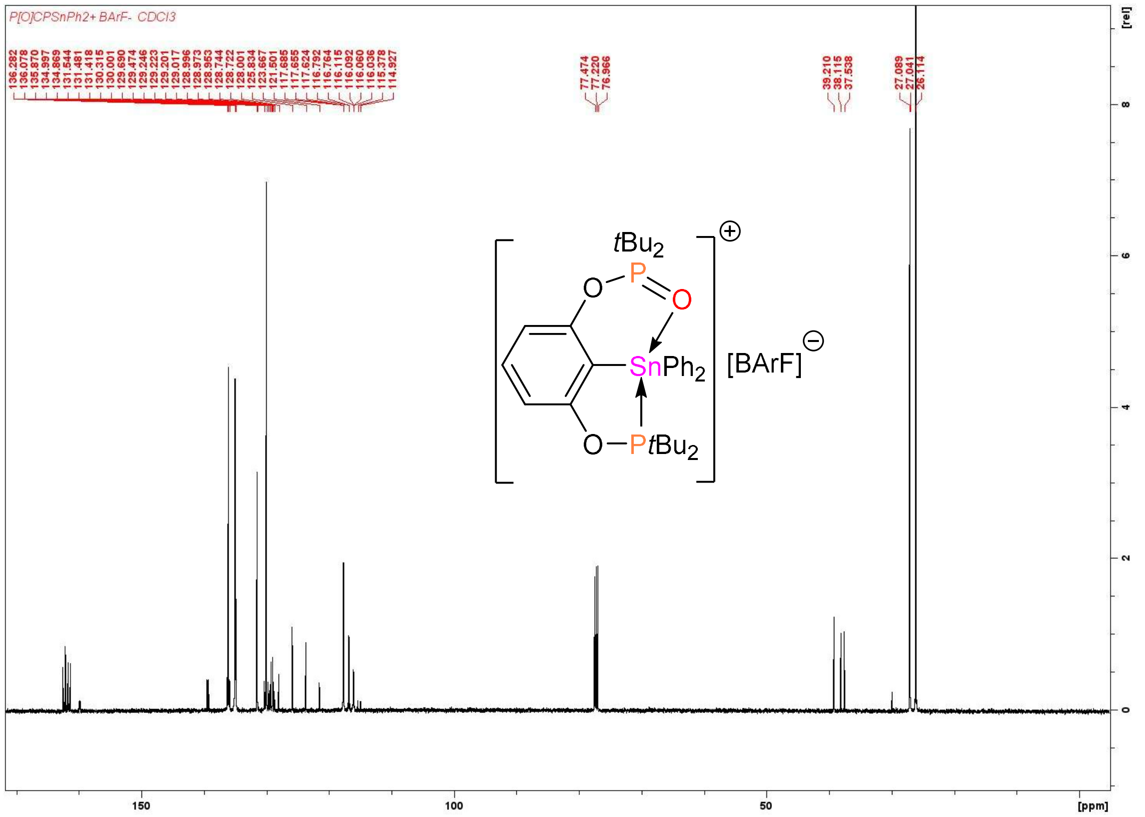

Figure S120. $^{13}C\{^{1}H\}$ NMR spectrum of $\mathbf{3^{PO+}[BArF]^{-}}$ (125.76 MHz, $CDCl_3$).

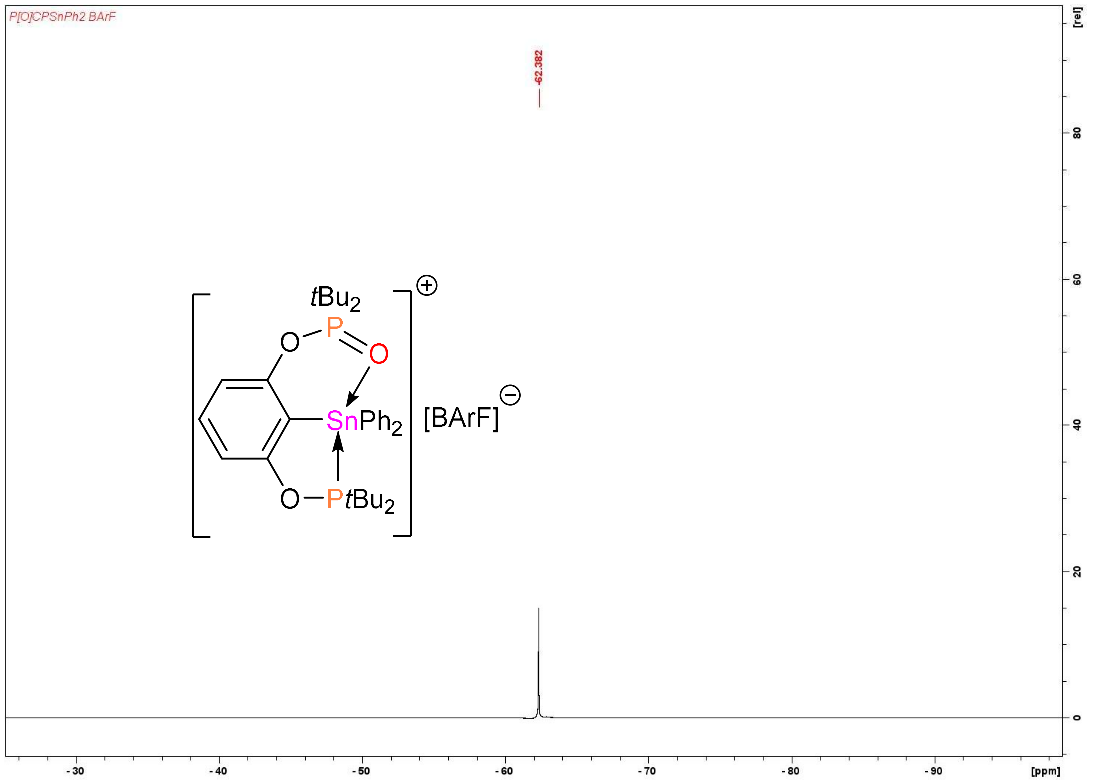

Figure S121. $^{19}F\{^{1}H\}$ NMR spectrum of $\mathbf{3^{PO+}[BArF]^{-}}$ (470.5 MHz, $CDCl_3$).

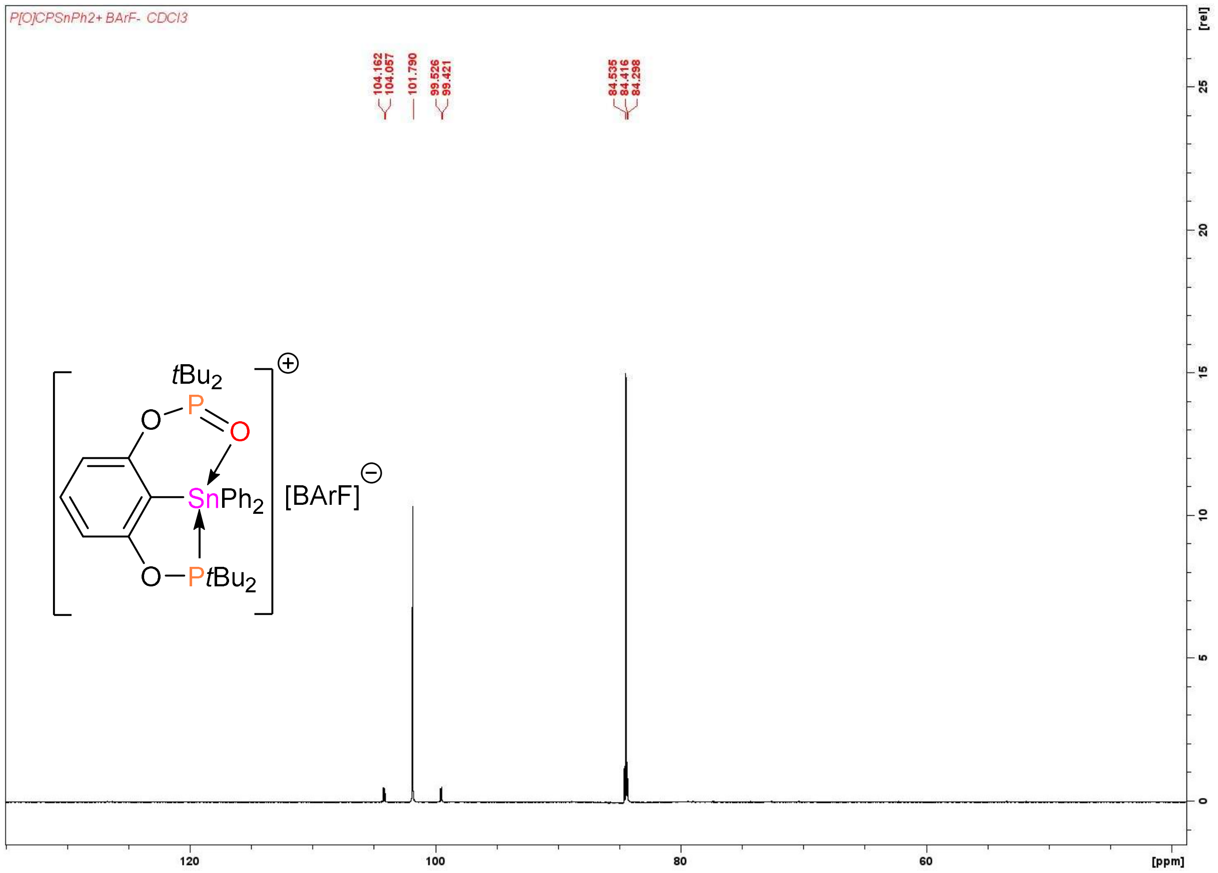

Figure S122. $^{31}P\{^{1}H\}$ NMR spectrum of $\mathbf{3^{PO+}[BArF]^{-}}$ (202.5 MHz MHz, $CDCl_3$).

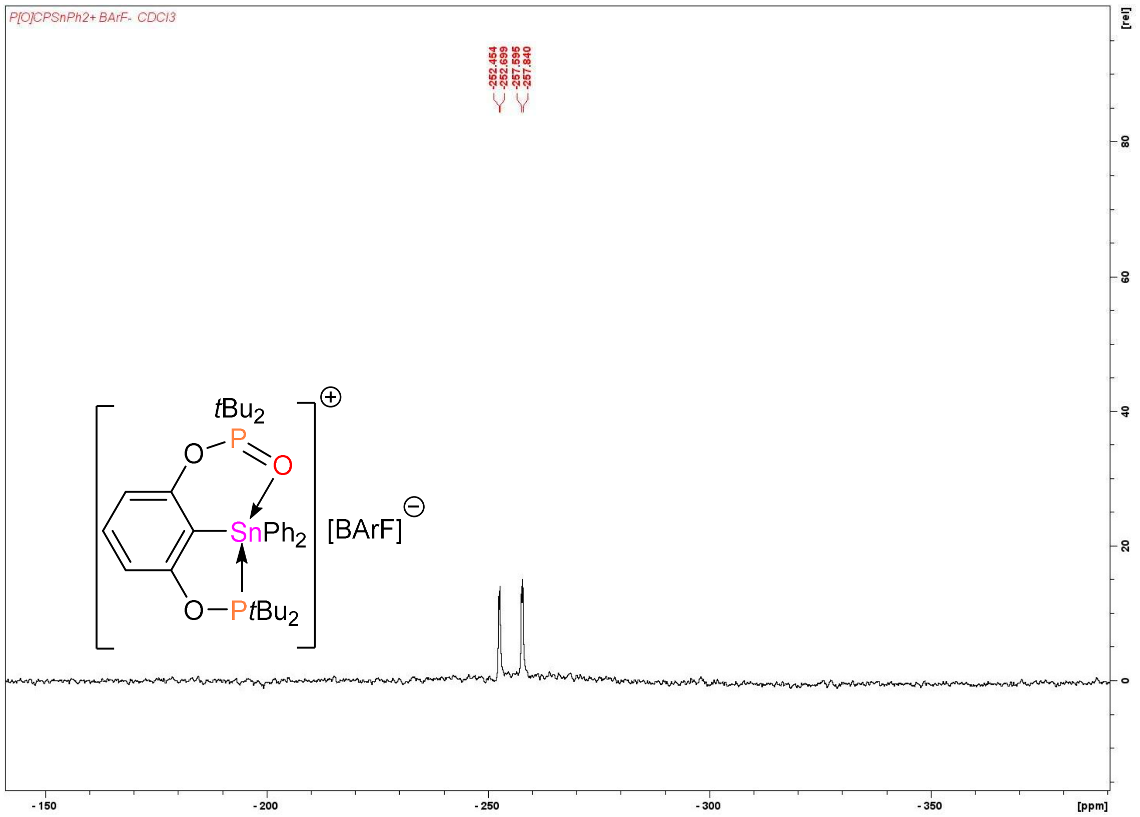

Figure S123. $^{119}Sn\{^{1}H\}$ NMR spectrum of $\mathbf{3^{PO+}[BArF]^{-}}$ (186.5MHz, $CDCl_3$).

Synthesis of {[2-(*t*Bu$_2$(O)PO)-6-(*t*Bu$_2$(S)PO)C$_6$H$_3$]SnPh$_2$}{[B[3,5-(CF$_3$)$_2$C$_6$H$_3$]$_4$} (3^{OS+}[BArF]$^-$)

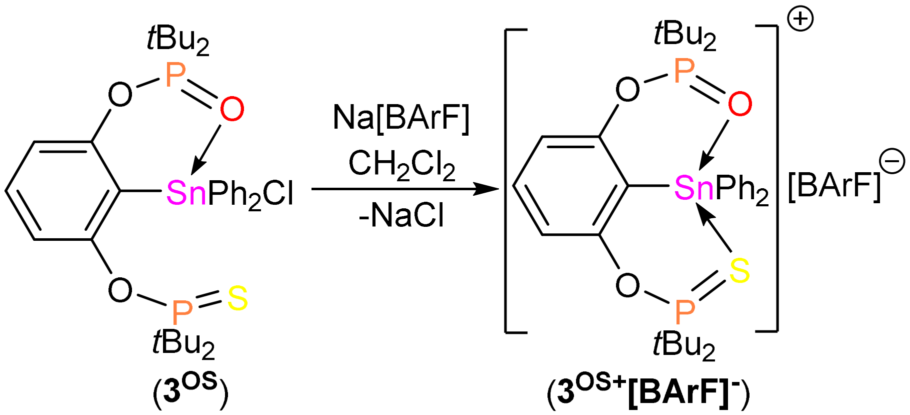

Solid Na[BArF] (60 mg; 0.07 mmol) was added in one portion to solution of **3OS** (55 mg; 0.07 mmol) in dichloromethane (10 ml). The reaction mixture was stirred for 30 minutes at room temperature and then incipient NaCl was removed by filtration. Colorless solution was concentrated to 1/2 of the original volume and layered with hexane. Crystallization at room temperature gave colorless crystals of compound **3^{OS+}[BArF]$^-$**. Yield of **3^{OS+}[BArF]$^-$** was 96 mg, (87 %), m. p. 216-219 °C. Single-crystals suitable for *sc*-XRD diffraction analysis were obtained by slow diffusion of hexane into saturated dichloromethane solution at room temperature. Anal. Calcd for $C_{66}H_{61}BF_{24}O_3P_2SSn$ (MW 1581.70): C, 50.1; H, 3.9 %. Found: C, 50.3; H, 4.1 %. **^{1}H NMR** (500 MHz, CDCl$_3$) δ (ppm): 1.03 and 1.17 [18+18H, d, ^3J(^{31}P,^{1}H) = 16/17.4 Hz, *t*Bu$_2$(O/S)P-C*H*$_3$], 7.16 [2H, m, Ar-*H*], 7.49 [6H, m, Ar-*H*], 7.53 [5H, m, Ar-*H*], 7.73 [12H, m, Ar-*H*]. **^{11}B{^{1}H} NMR** (160.42 MHz, CDCl$_3$) δ (ppm): -6.6 [s]. **^{13}C{^{1}H} NMR** (125.78 MHz, CDCl$_3$) δ (ppm): 26.1 and 27.0 [s, *t*Bu$_2$(O/S)P-*C*H$_3$], 37.5 [d, ^1J(^{31}P,^{13}C) = 72.3 Hz, *t*Bu$_2$(O)P-*C*], 42.4 [d, ^1J(^{31}P,^{13}C) = 46.6 Hz, *t*Bu$_2$(S)P-*C*], 117.7 [m, Ar-*C*], 120.3 [d, ^nJ(^{31}P,^{13}C) = 5.5 Hz, ^nJ($^{119/117}$Sn,^{13}C) = 28.7 Hz, Ar-*C*], 120.6 [d, ^nJ(^{31}P,^{13}C) = 4.2 Hz, ^nJ($^{119/117}$Sn,^{13}C) = 29.4 Hz, Ar-*C*], 121.4 [m, Ar-*C*], 124.8 [q, ^1J(^{19}F,^{13}C) = 273 Hz, *C*F$_3$], 129.1 [qq, ^2J(^{19}F,^{13}C) = 31.5 Hz, ^4J(^{19}F,^{13}C) = 2.8 Hz, Ar-*C*], 129.7 [s, ^3J($^{119/117}$Sn,^{13}C) = 81.3 Hz, Ar-*C*], 131.9 [s, ^4J($^{119/117}$Sn,^{13}C) = 16.5 Hz, Ar-*C*], 134.1 [s, Ar-*C*], 135.0 [s, Ar-*C*], 135.9 [s,

$^{2}J(^{119/117}Sn,^{13}C)$ = 52.9 Hz Ar-*C*], 141.4 [d, $^{1}J(^{119/117}Sn,^{13}C)$ = 886/845 Hz, Ar-*C*], 158.2 [d, $^{n}J(^{31}P,^{13}C)$ = 10.5 Hz, Ar-*C*], 158.5 [d, $^{n}J(^{31}P,^{13}C)$ = 13.9 Hz, Ar-*C*], 161.9 [q, $^{1}J(^{13}C,^{11}B)$ = 50 Hz, Ar-*C*]. **^{19}F{^{1}H} NMR** (376.3 MHz, $CDCl_3$) δ (ppm): -62.4 [s]. **^{31}P{^{1}H} NMR** (202.5 MHz, $CDCl_3$) δ (ppm): 84.6 [s, *t*Bu_2(O)*P*], 132.4 [s, *t*Bu_2(S)*P*]. **^{119}Sn{^{1}H} NMR** (186.5 MHz, $CDCl_3$) δ (ppm): -293.9 [s].

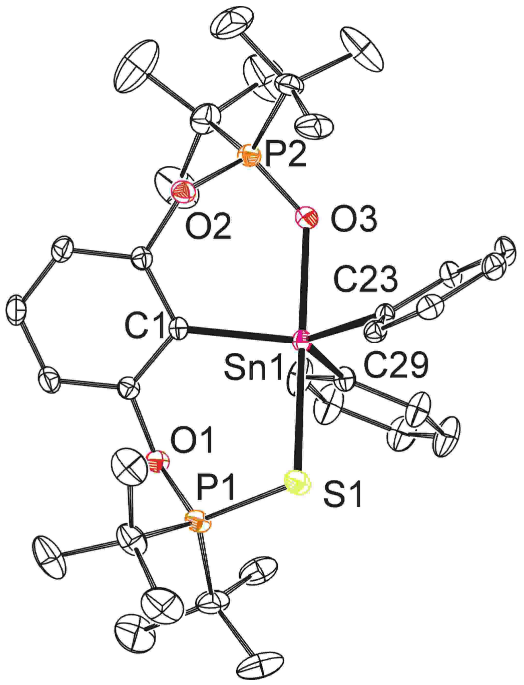

Figure S124. Molecular structure of **3^{OS+}[BArF]$^-$**. ORTEP with 30% probability ellipsoid level. Hydrogen atoms and [BArF] are omitted. Selected structural bond lengths [Å]: Sn(1)-C(1) 2.156(3), Sn(1)-C(23) 2.118(3), Sn(1)-C(29) 2.118(2), Sn(1)-O(3) 2.225(2), Sn(1)-S(1) 2.7065(8); bonding angles [°]: O(3)-Sn(1)-S(1) 171.33(6), C(1)-Sn(1)-C(23) 128.28(11), C(1)-Sn(1)-C(29) 114.91(13), C(23)-Sn(1)-C(29) 116.73(13).

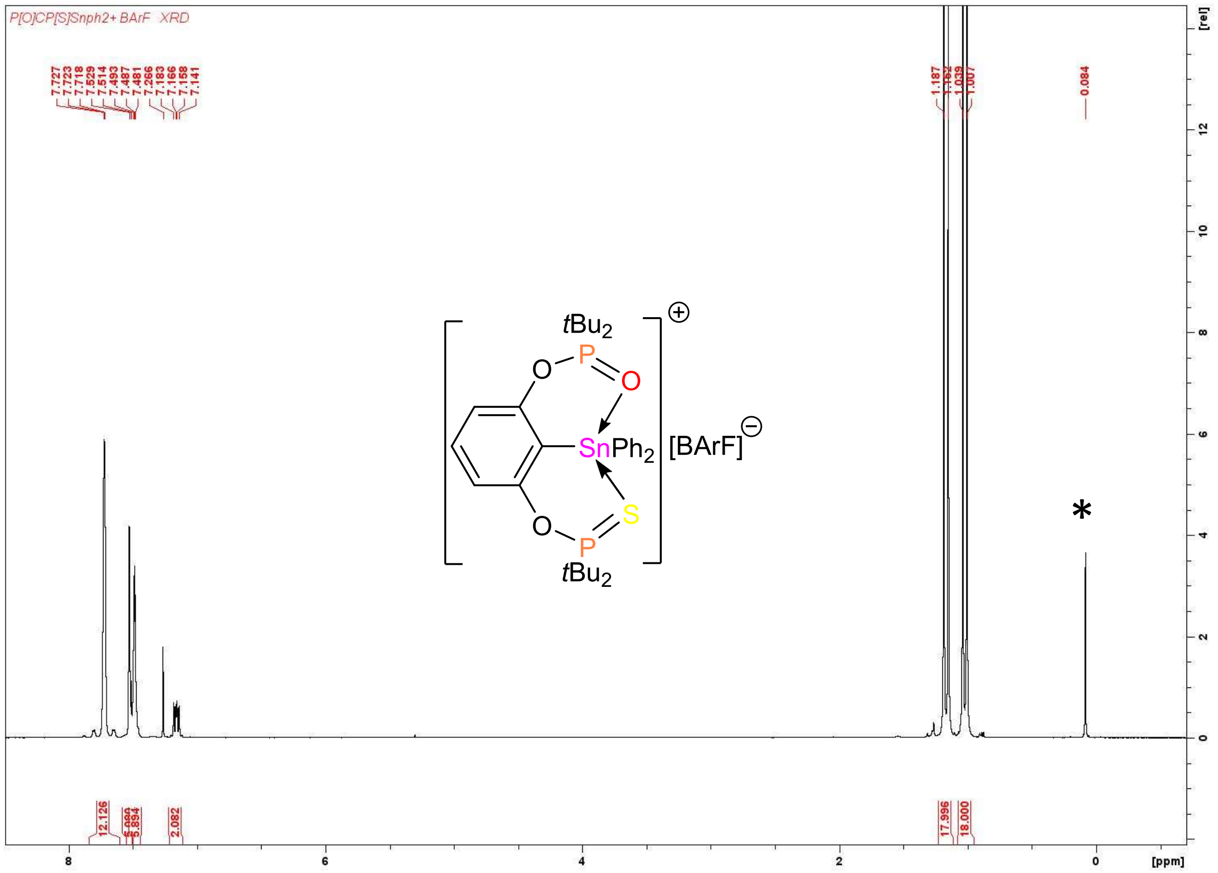

Figure 125 ^{1}H NMR spectrum of **3^{OS+}[BArF]$^-$** (500 MHz, $CDCl_3$). *Signal of silicon grease.

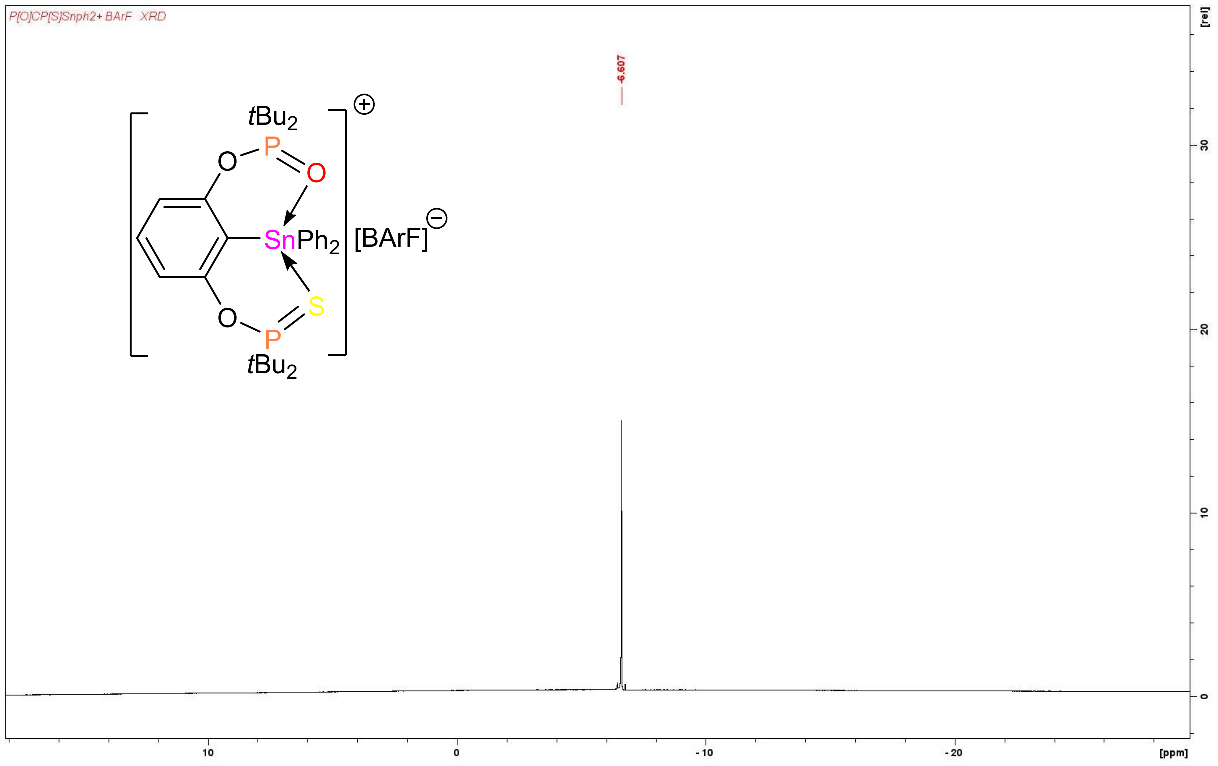

Figure S126. $^{11}B\{^1H\}$ NMR spectrum of **3^{OS+}[BArF]$^-$** (160.42 MHz MHz, $CDCl_3$).

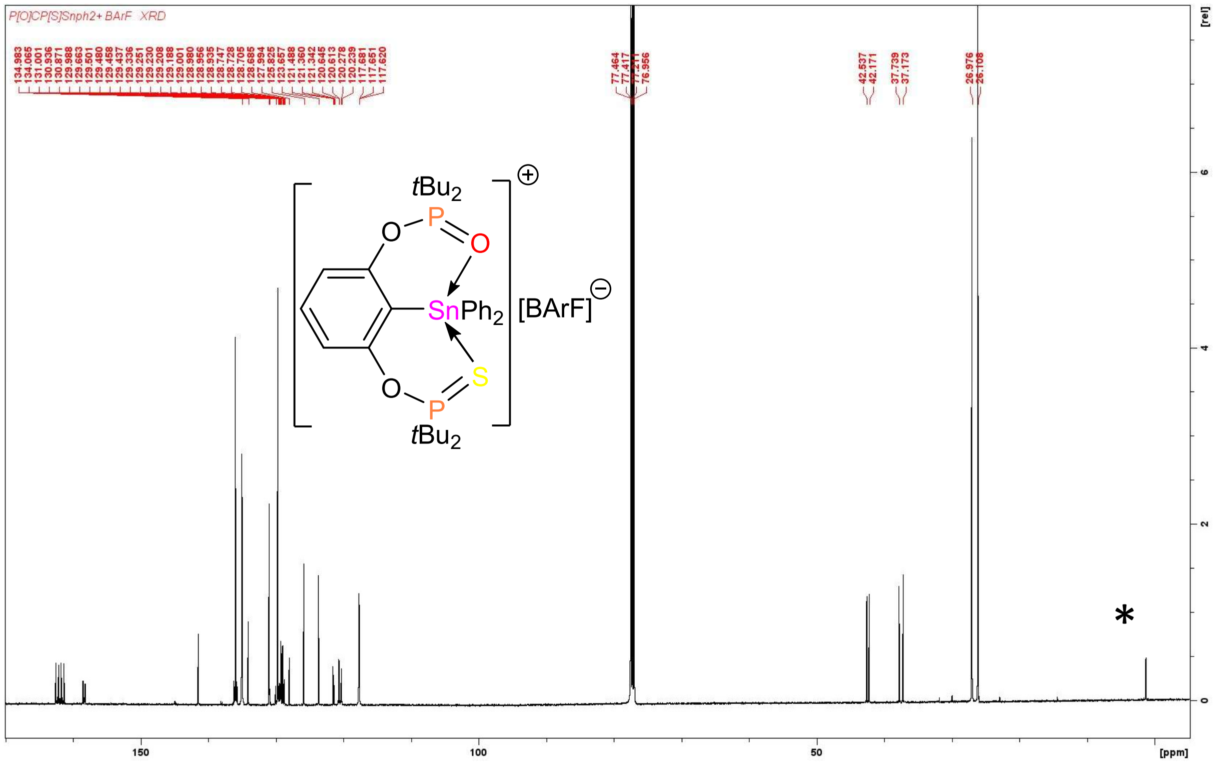

Figure S127. $^{13}C\{^{1}H\}$ NMR spectrum of $\mathbf{3^{OS+}[BArF]^{-}}$ (125.76 MHz, $CDCl_3$). *Signal of silicon grease.

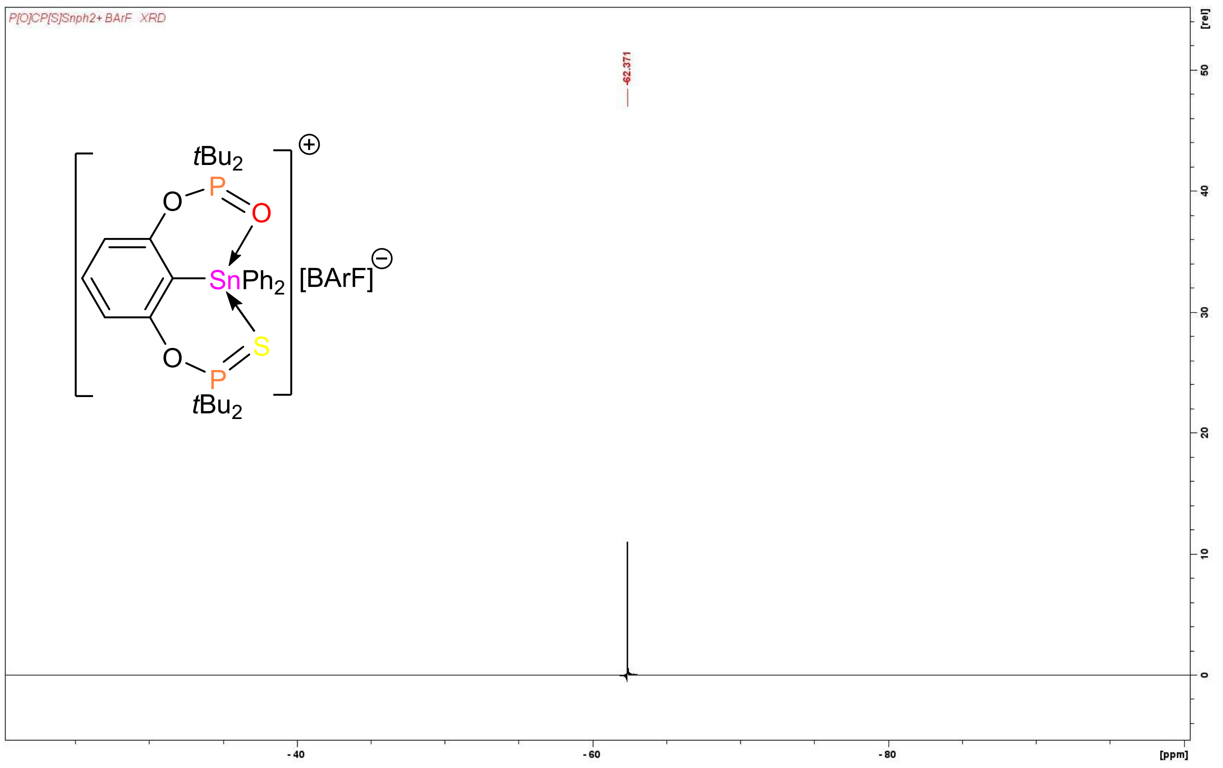

Figure S128. $^{19}F\{^{1}H\}$ NMR spectrum of $\mathbf{3^{OS+}[BArF]^{-}}$ (470.5 MHz, $CDCl_3$).

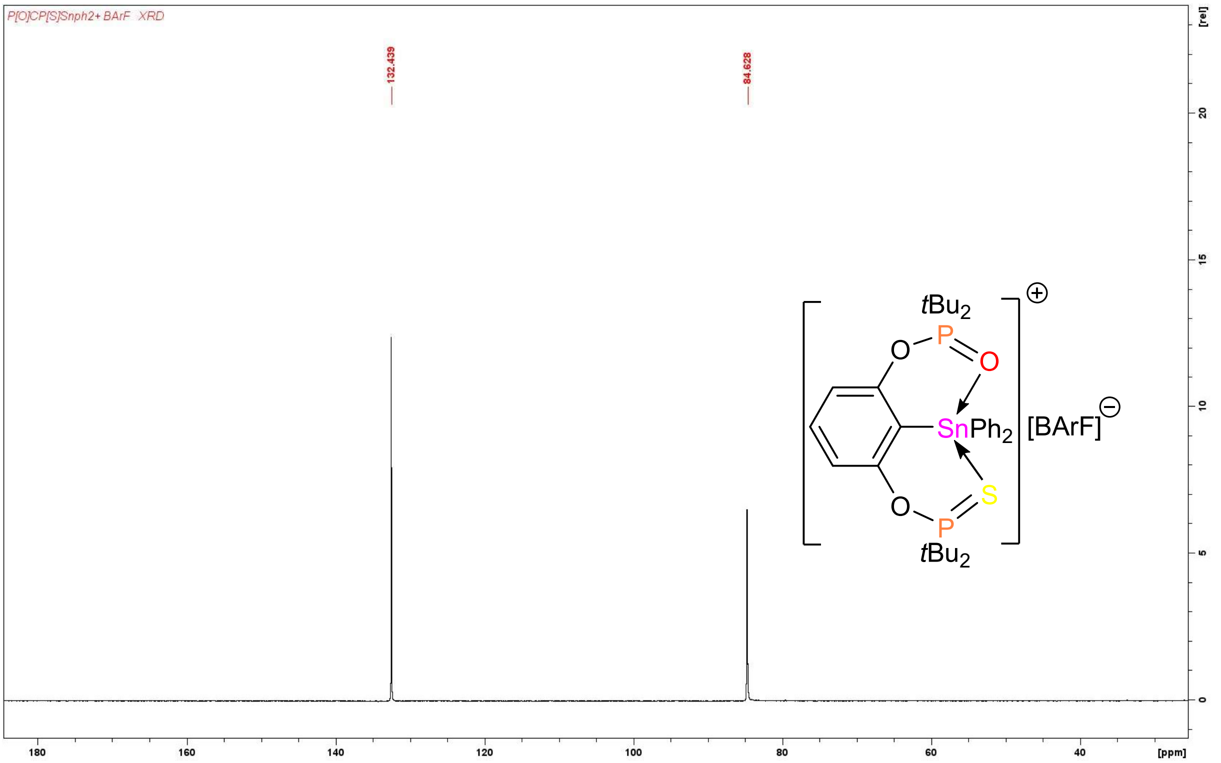

Figure S129. $^{31}P\{^{1}H\}$ NMR spectrum of $\mathbf{3^{OS+}[BArF]^{-}}$ (202.5 MHz MHz, $CDCl_3$).

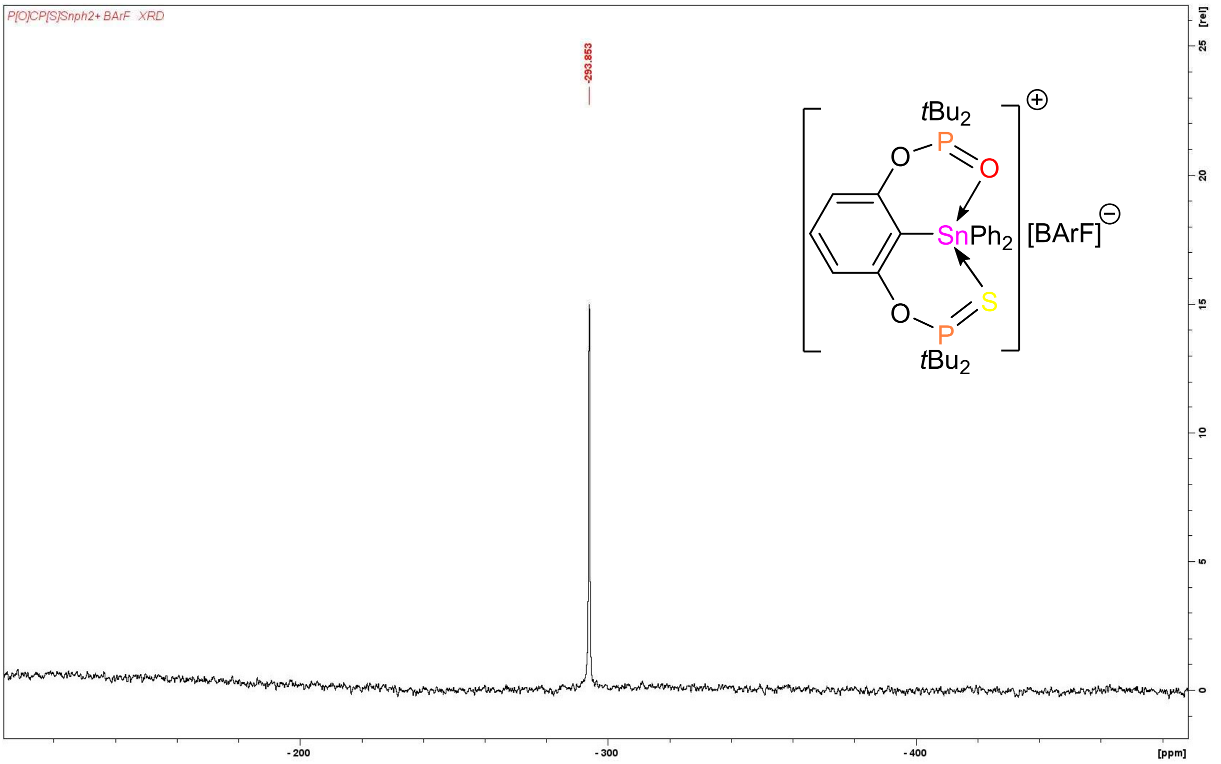

Figure S130. $^{119}Sn\{^{1}H\}$ NMR spectrum of $\mathbf{3^{OS+}[BArF]^{-}}$ (186.5MHz, $CDCl_3$).

Synthesis of {[2-(*t*Bu$_2$(O)PO)-6-(*t*Bu$_2$(Se)PO)C$_6$H$_3$]SnPh$_2$}{[B[3,5-(CF$_3$)$_2$C$_6$H$_3$]$_4$} (3^{OSe+}[BArF]$^-$)

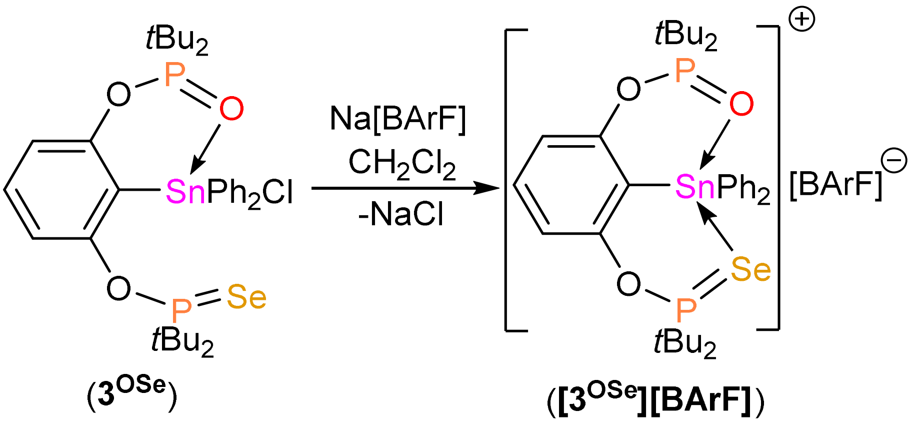

Solid Na[BArF] (118 mg; 0.13 mmol) was added in one portion to solution of **3^{OSe}** (115 mg; 0.13 mmol) in dichloromethane (10 ml). The reaction mixture was stirred for 30 minutes at room temperature and then incipient NaCl was removed by filtration. Colorless solution was concentrated to 1/2 of the original volume and layered with hexane. Crystallization at room temperature gave colorless crystals of compound **3^{OSe+}[BArF]$^-$**. Yield of **3^{OSe+}[BArF]$^-$** was 192 mg, (85 %), m. p. 220-222 °C. Single-crystals suitable for *sc*-XRD diffraction analysis were obtained from saturated solution using dichloromethane/hexane mixture at -30 °C. Anal. Calcd for $C_{66}H_{61}BF_{24}O_3P_2SeSn$ (MW 1628.61): C, 48.7; H, 3.8 %. Found: C, 49.0; H, 3.9 %. **^{1}H NMR** (500 MHz, CDCl$_3$) δ (ppm): 1.02 and 1.20 [18+18H, d, $^3J(^{31}P,^1H)$ = 16/17.9 Hz, *t*Bu$_2$(O/Se)P-C*H*$_3$], 7.18 [2H, m, Ar-*H*], 7.48 [6H, m, Ar-*H*], 7.54 [5H, m, Ar-*H*], 7.73 [8H, m, Ar-*H*], 7.75 [4H, m, $^3J(^{119/117}Sn, ^1H)$ = 77.3 Hz, Ar-*H*]. **^{11}B{^{1}H} NMR** (160.42 MHz, CDCl$_3$) δ (ppm): -7.2 [s]. **^{13}C{^{1}H} NMR** (125.78 MHz, CDCl$_3$) δ (ppm): 26.2 and 27.2 [s, *t*Bu$_2$(O/Se)P-*C*H$_3$], 37.5 [d, $^1J(^{31}P,^{13}C)$ = 71.2 Hz, *t*Bu$_2$(O)P-*C*], 43.1 [d, $^1J(^{31}P,^{13}C)$ = 35.1 Hz, *t*Bu$_2$(Se)P-*C*], 117.7 [m, Ar-*C*], 120.6 [d, $^nJ(^{31}P,^{13}C)$ = 5.6 Hz, $^nJ(^{119/117}Sn,^{13}C)$ = 27.9 Hz, Ar-*C*], 121.0 [d, $^nJ(^{31}P,^{13}C)$ = 3.5 Hz, $^nJ(^{119/117}Sn,^{13}C)$ = 29.9 Hz, Ar-*C*], 121.3 [m, Ar-*C*], 124.8 [q, $^1J(^{19}F,^{13}C)$ = 273 Hz, *C*F$_3$], 129.1 [qq, $^2J(^{19}F,^{13}C)$ = 31.5 Hz, $^4J(^{19}F,^{13}C)$ = 2.8 Hz, Ar-*C*], 129.6 [s, $^3J(^{119/117}Sn,^{13}C)$ = 81.8 Hz, Ar-*C*], 130.8 [s, $^4J(^{119/117}Sn,^{13}C)$ = 16.6 Hz, Ar-*C*], 133.8 [s, Ar-

C], 135.0 [s, Ar-*C*], 135.9 [s, $^{2}J(^{119/117}Sn,^{13}C)$ = 52.6 Hz Ar-*C*], 141.8 [d, $^{1}J(^{119/117}Sn,^{13}C)$ = 881/841 Hz, Ar-*C*], 157.9 [d, $^{n}J(^{31}P,^{13}C)$ = 10.7 Hz, Ar-*C*], 158.6 [d, $^{n}J(^{31}P,^{13}C)$ = 13.7 Hz, Ar-*C*], 161.9 [q, $^{1}J(^{13}C,^{11}B)$ = 50 Hz, Ar-*C*]. **^{19}F{^{1}H} NMR** (376.3 MHz, $CDCl_3$) δ (ppm): -62.4 [s]. **^{31}P{^{1}H} NMR** (202.5 MHz, $CDCl_3$) δ (ppm): 84.4 [s, $^{n}J(^{119/117}Sn,^{31}P)$ = 36.4 Hz, *t*Bu_2(O)*P*], 139.9 [s, $^{1}J(^{77}Se,^{31}P)$ = 647 Hz, *t*Bu_2(Se)*P*]. **^{77}Se{^{1}H} NMR** (95.4 MHz, $CDCl_3$) δ (ppm): -220.1 [d, $^{1}J(^{77}Se,^{31}P)$ = 647 Hz]. **^{119}Sn{^{1}H} NMR** (186.5 MHz, $CDCl_3$) δ (ppm): -298.1 [d, $^{n}J(^{119/117}Sn,^{31}P)$ = 36.4 Hz, $^{1}J(^{119/117}Sn, ^{77}Se)$ = 440 Hz].

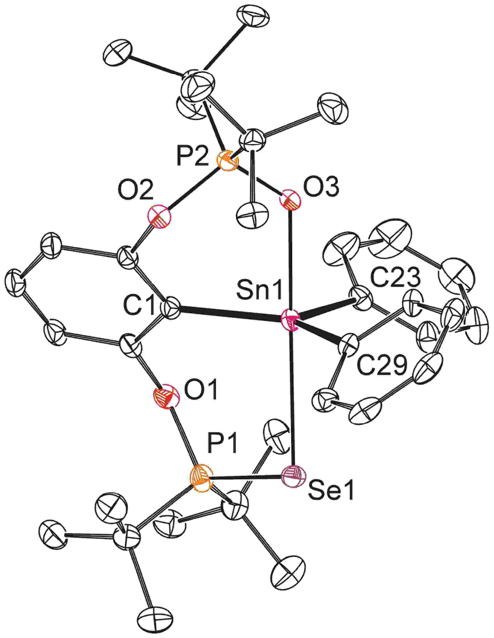

Figure S131. Molecular structure of **3^{OSe+}[BArF]$^{-}$**. ORTEP with 30% probability ellipsoid level. Hydrogen atoms and [BArF] anion are omitted. Selected structural bond lengths [Å]: Sn(1)-C(1) 2.115(3), Sn(1)-C(23) 2.115(3), Sn(1)-C(29) 2.127(3), Sn(1)-O(3) 2.236(3), Sn(1)-Se(1) 2.8031(6); bonding angles [°]: O(3)-Sn(1)-Se(1) 170.48(7), C(1)-Sn(1)-C(23) 126.23(11), C(1)-Sn(1)-C(29) 118.70(11), C(23)-Sn(1)-C(29) 114.95(11).

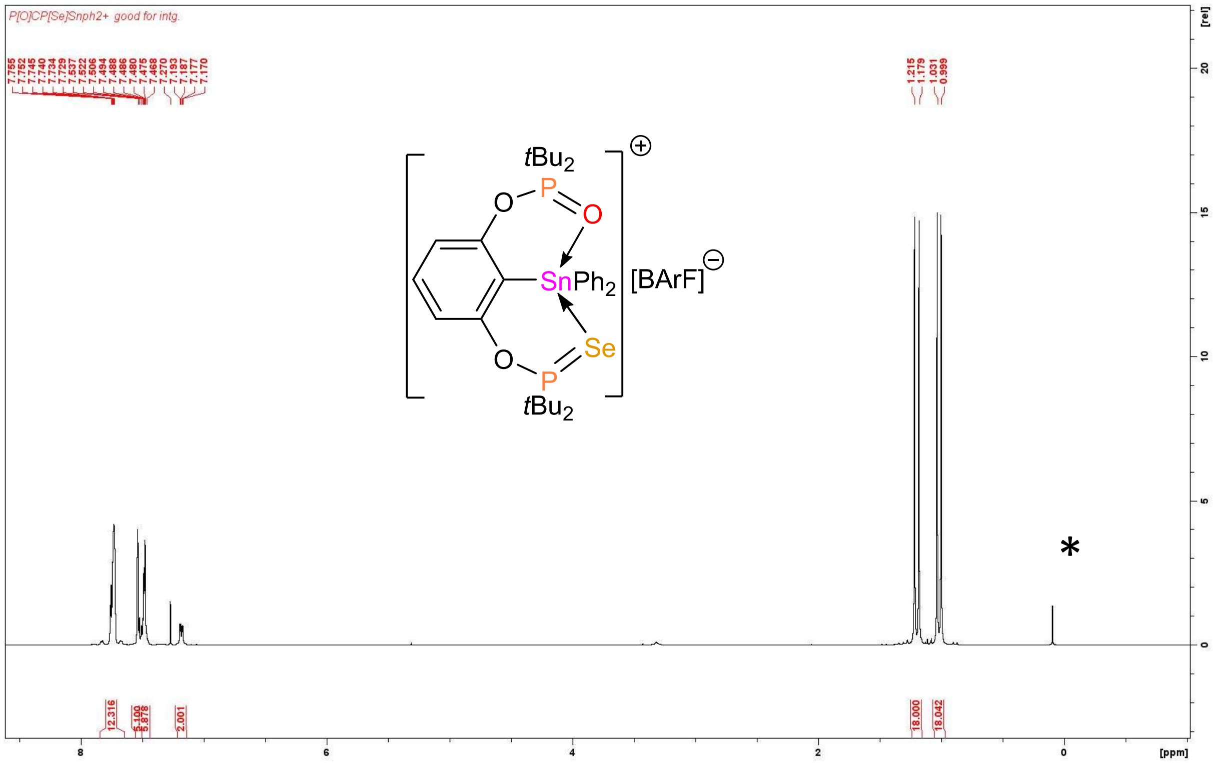

Figure S132. ^{1}H NMR spectrum of $\mathbf{3^{OSe+}[BArF]^-}$ (500 MHz, $CDCl_3$). *Signal of silicon grease.

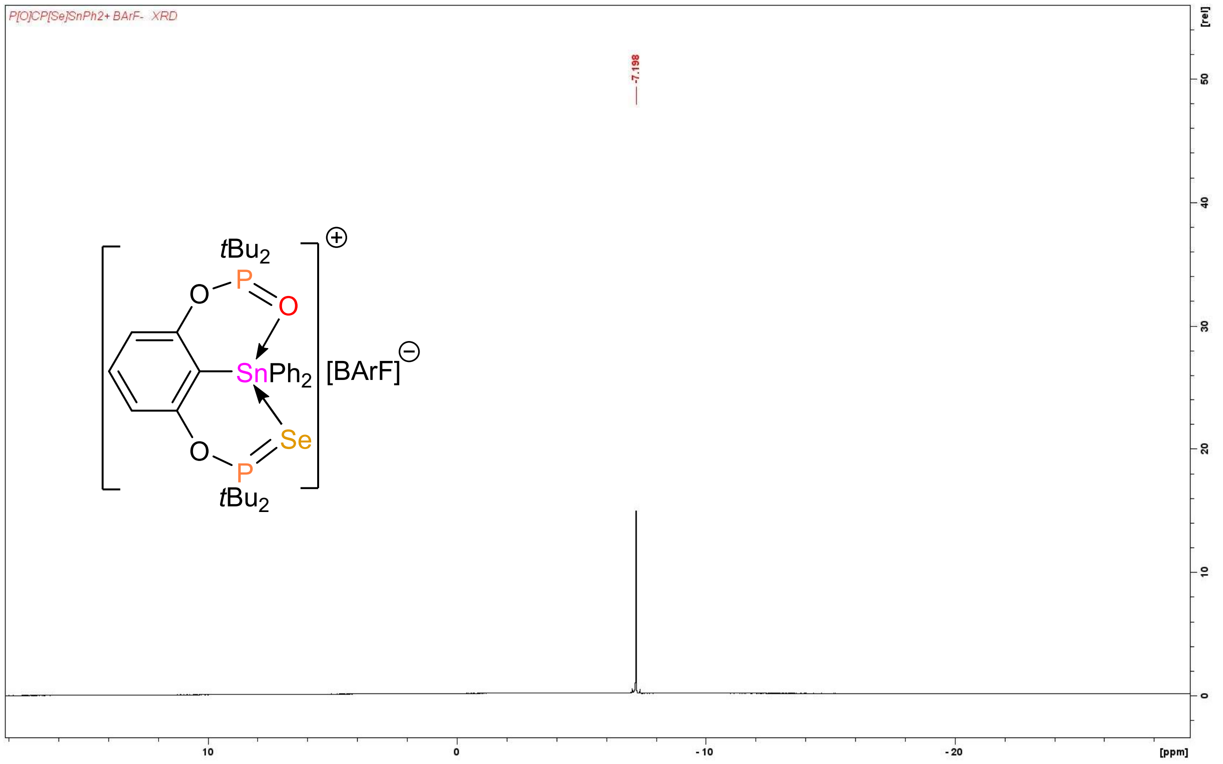

Figure S133. $^{11}B\{^1H\}$ NMR spectrum of $\mathbf{3^{OSe+}[BArF]^-}$ (160.42 MHz MHz, $CDCl_3$).

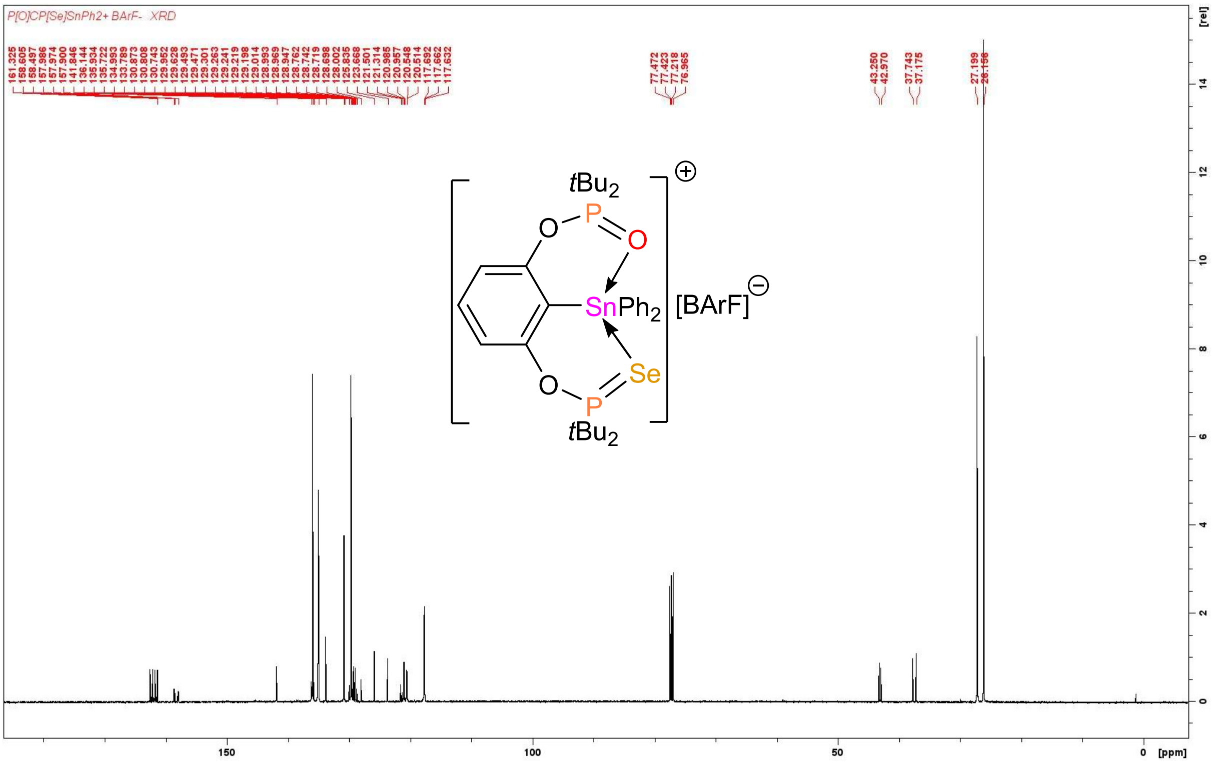

Figure S134. $^{13}C\{^1H\}$ NMR spectrum of $\mathbf{3^{OSe+}[BArF]^-}$ (125.76 MHz, $CDCl_3$).

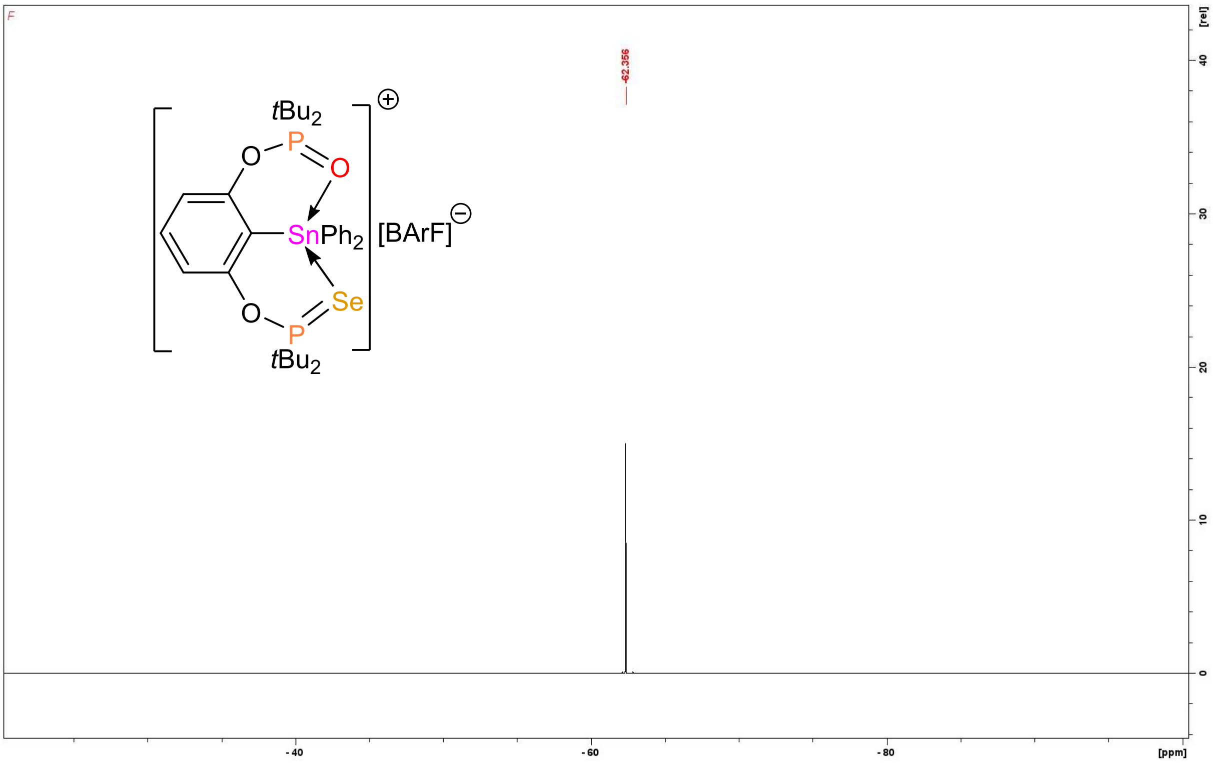

Figure S135. $^{19}F\{^1H\}$ NMR spectrum of $\mathbf{3^{OSe+}[BArF]^-}$ (470.5 MHz, $CDCl_3$).

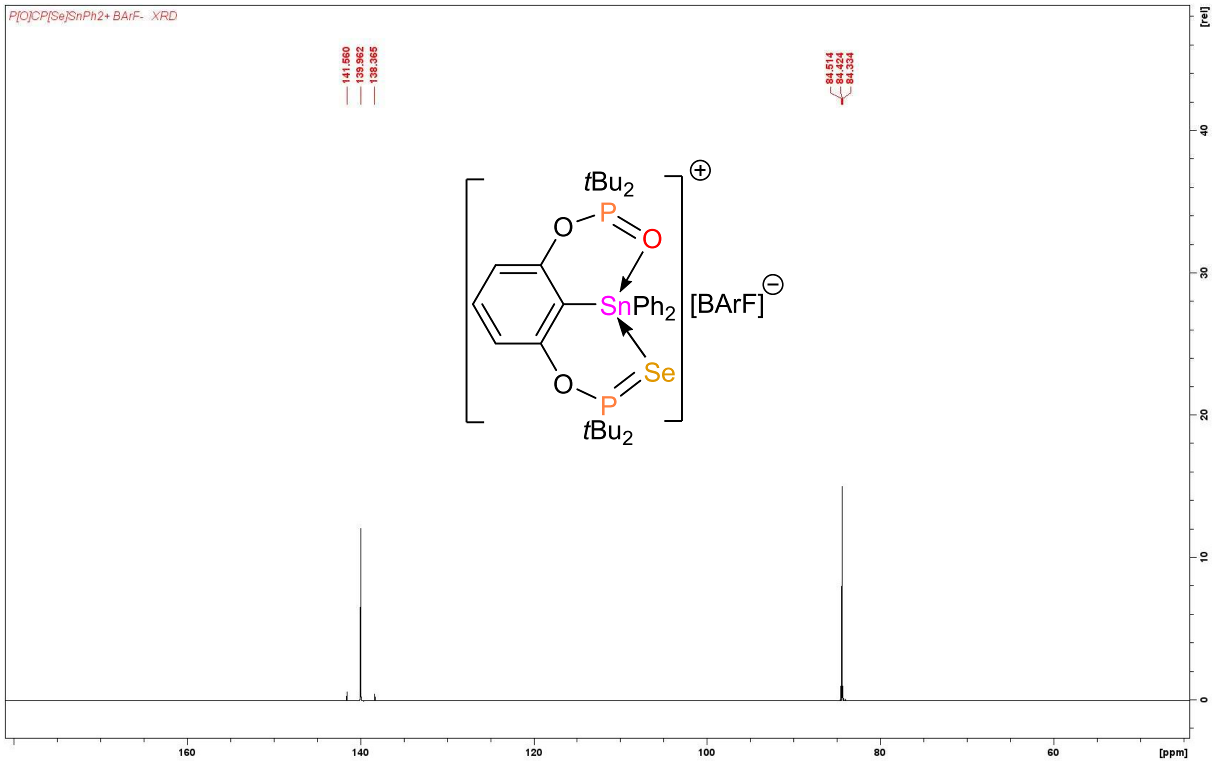

Figure S136. $^{31}P\{^{1}H\}$ NMR spectrum of $\mathbf{3^{OSe+}[BArF]^{-}}$ (202.5 MHz MHz, $CDCl_3$).

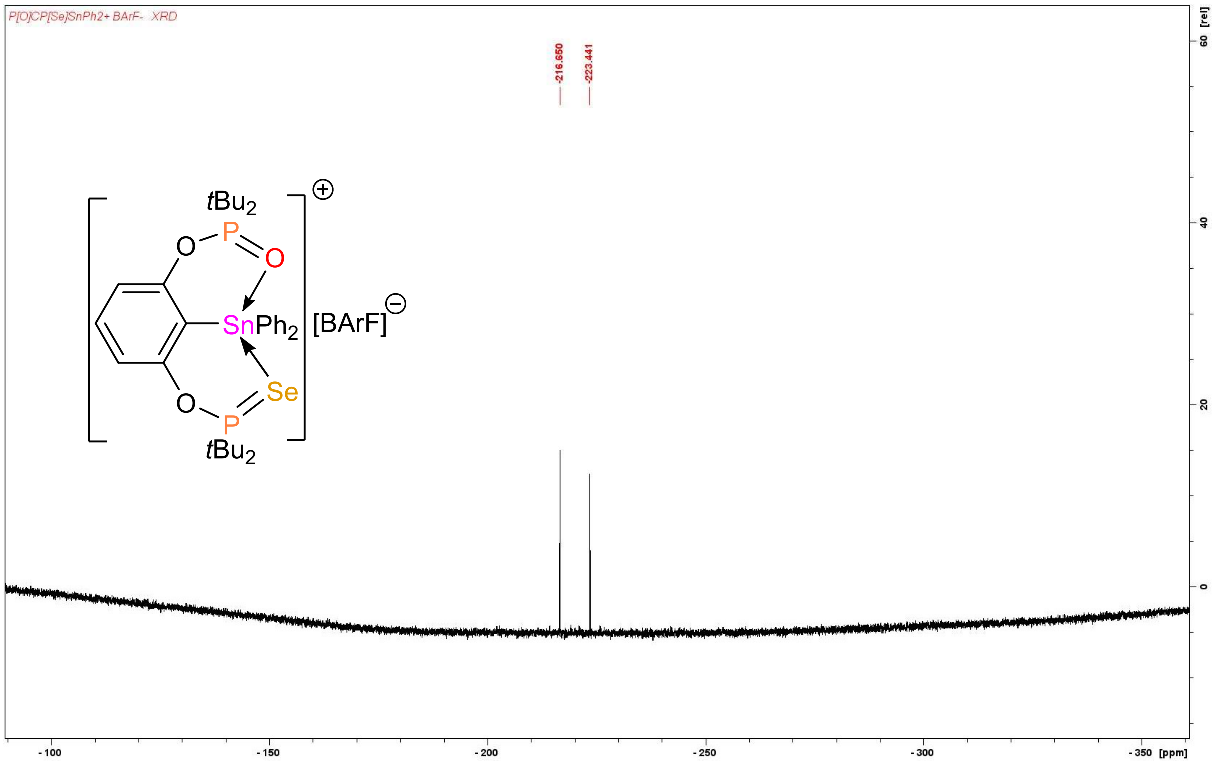

Figure S137. $^{77}Se\{^{1}H\}$ NMR spectrum of $\mathbf{3^{OSe+}[BArF]^{-}}$ (95.4 MHz, $CDCl_3$).

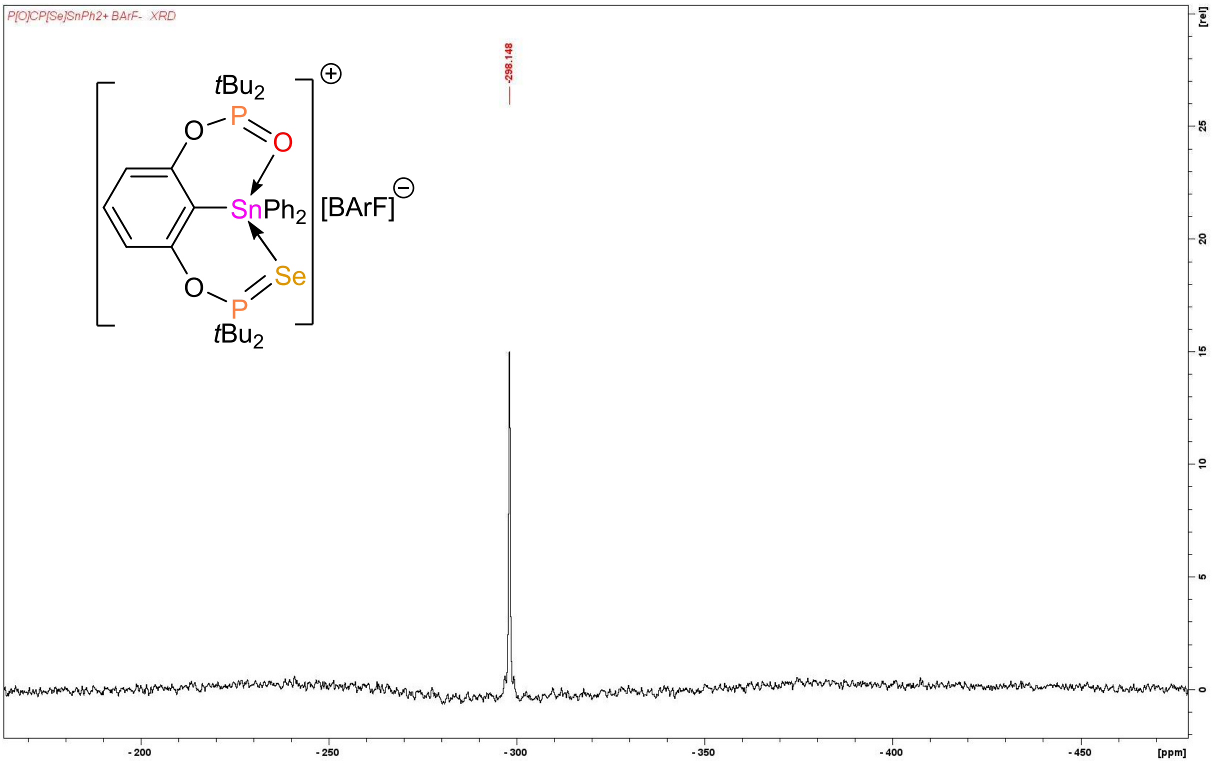

Figure S138. $^{119}Sn\{^1H\}$ NMR spectrum of $\mathbf{3^{OSe+}[BArF]^-}$ (186.5MHz, $CDCl_3$).

Synthesis of 2,6-($t$$Bu_2$(O)PO)$_2$$C_6H_3$Br (Ar^{O}Br)

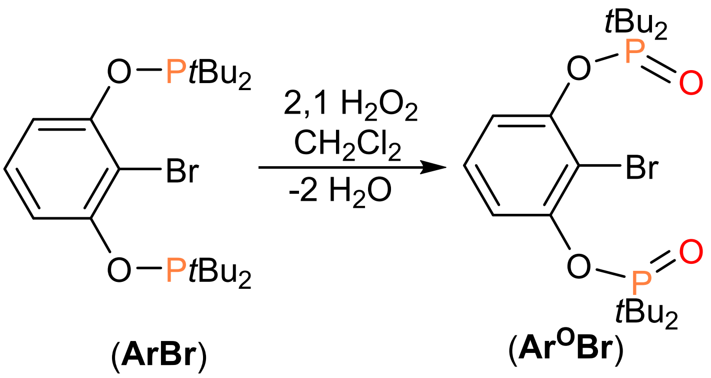

30% solution of hydrogen peroxide in water (0.24 ml; 2.35 mmol) was slowly added to solution of **ArBr** (526 mg; 1.10 mmol) in dichloromethane (20 ml). Mixture was stirred for 5 min and transferred to Schlenk flask with dried molecular sieves. After 10 min of vigorous stirring, the molecular sieves were removed by filtration. Solvent was removed in vacuo and white powder dried to remove any excess traces of water. Recrystallisation from dichloromethane/hexane solution gave colorless crystals of compound **Ar^{O}Br**. Yield of **Ar^{O}Br** was 380 mg, (68 %), m.

p. 208-210°C. Single-crystals suitable for *sc*-XRD diffraction analysis were obtained from saturated solution using dichloromethane/hexane mixture at -30°C. Anal. Calcd for $C_{22}H_{39}BrO_4P_2$ (MW 509.40): C, 51.9; H, 7.8 %. Found: C, 52.1; H, 7.9 %. **^{1}H NMR** (400 MHz, $CDCl_3$) δ (ppm): 1.33 [36H, d, $^3J(^{31}P,^1H)$ = 15.0 Hz, *t*Bu$_2$(S)P-C*H*$_3$], 7.08 [1H, t, $^3J(^1H,^1H)$ = 9.0 Hz, Ar-*H*], 7.64 [2H, d, $^3J(^1H,^1H)$ = 9.0 Hz, Ar-*H*]. **^{13}C{^{1}H} NMR** (125.78 MHz, $CDCl_3$) δ (ppm): 26.6 [s, *t*Bu$_2$P-*C*H$_3$], 37.5 [d, $^1J(^{31}P,^{13}C)$ = 80.2 Hz, *t*Bu$_2$P-*C*], 104.7 [t, $^3J(^{31}P,^{13}C)$ = 7.3 Hz], 115.1 [d, $^3J(^{31}P,^{13}C)$ = 1.9 Hz, Ar-*C*], 128.6 [s, Ar-*C*], 152.4 [d, $^2J(^{31}P,^{13}C)$ = 9.4 Hz, Ar-*C*]. **^{31}P{^{1}H} NMR** (202.5 MHz, $CDCl_3$) δ (ppm): 69.9 [s].

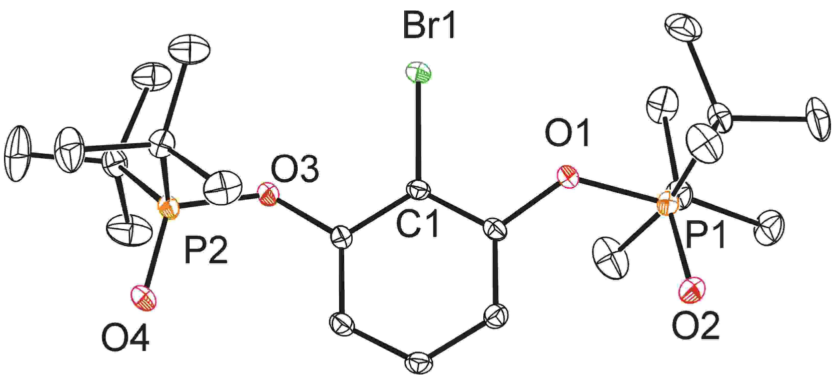

Figure S139. Molecular structure of **Ar^{O}Br**. ORTEP with 30% probability ellipsoid level. Hydrogen atoms are omitted. Selected structural bond lengths [Å]: P(1)-O(2) 1.469(2), P(2)-O(4) 1.4778(18).

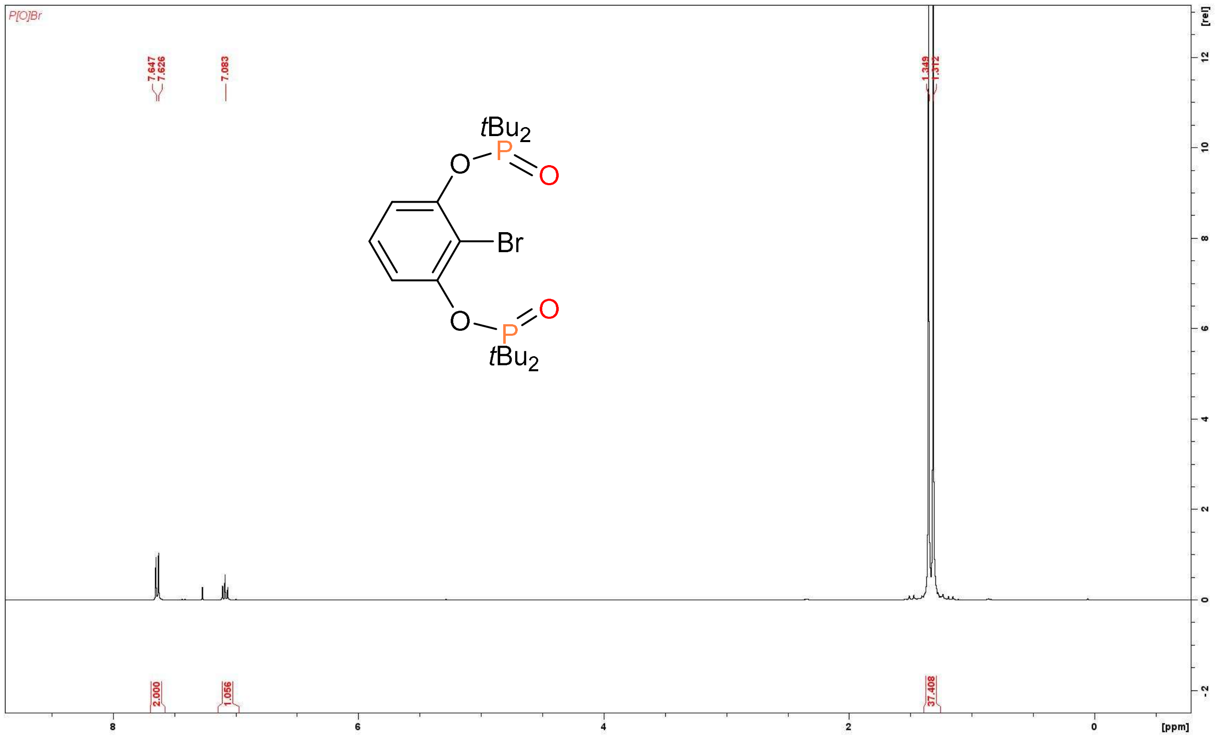

Figure S140. ^{1}H NMR spectrum of **Ar^{O}Br** (400 MHz, $CDCl_3$).

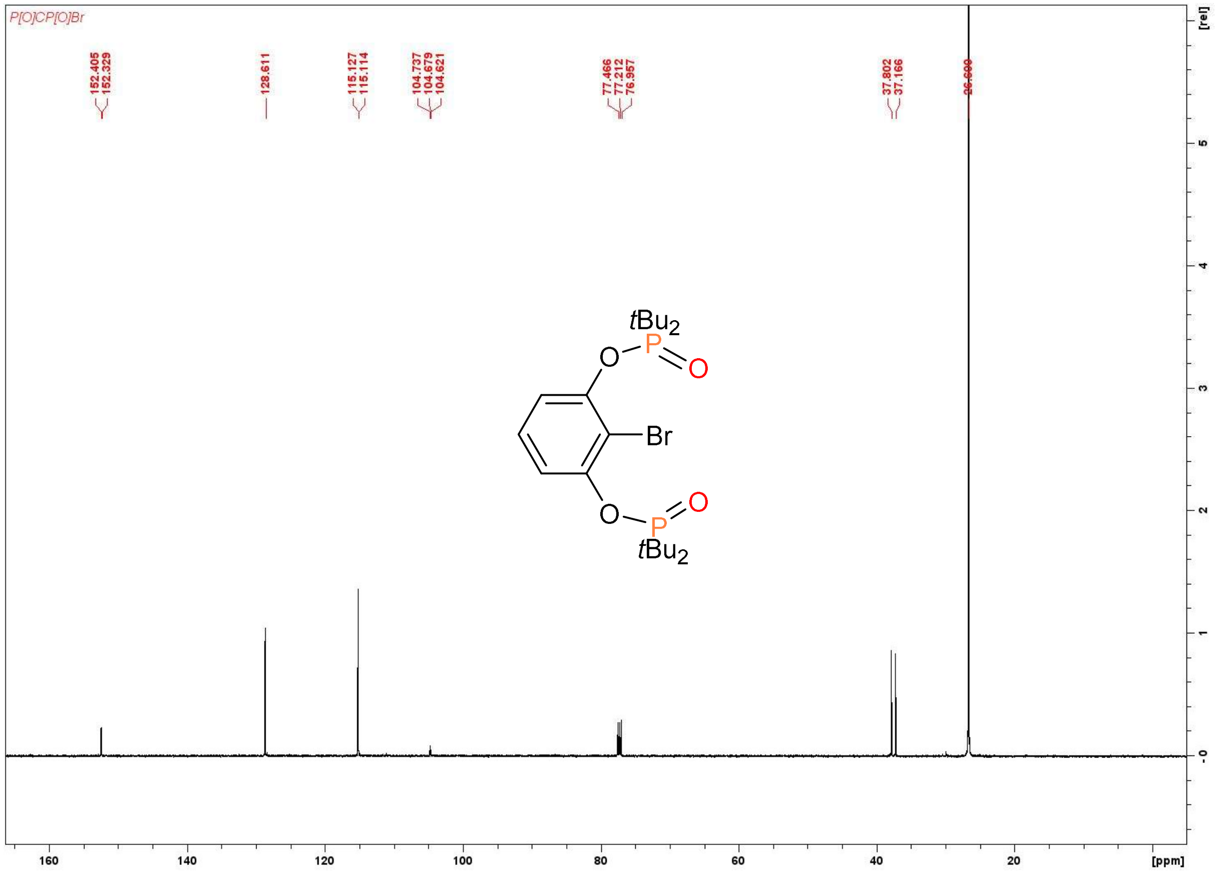

Figure S141. $^{13}C\{^{1}H\}$ NMR spectrum of **Ar^{O}Br** (125.76 MHz, $CDCl_3$).

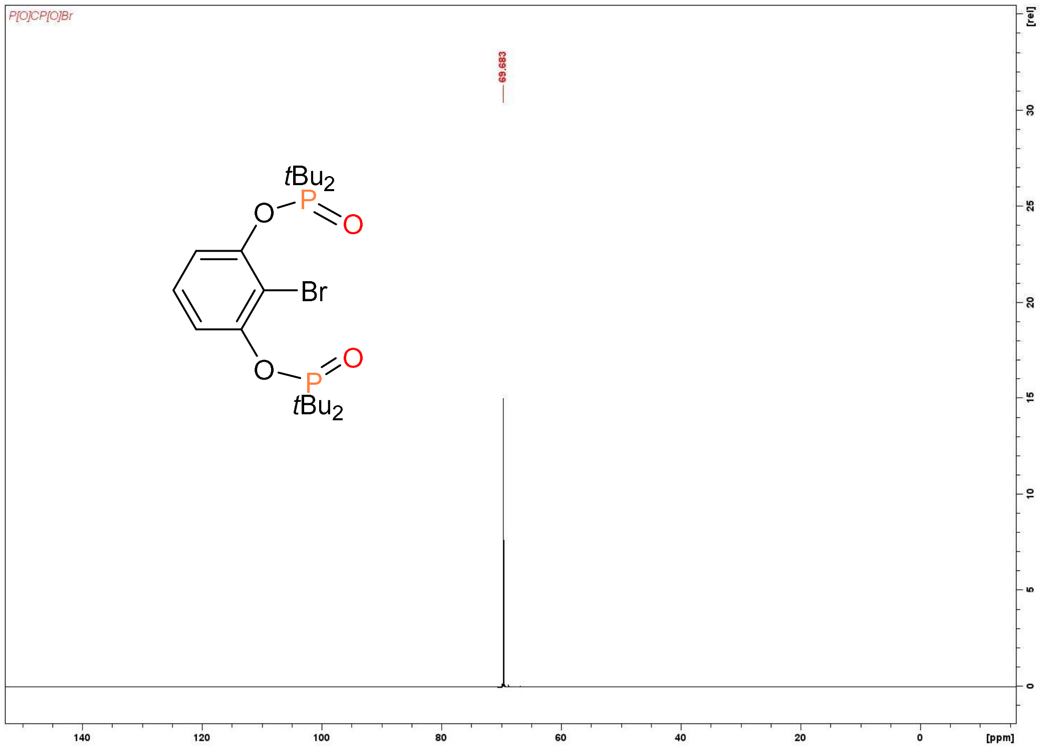

Figure S142. $^{31}P\{^{1}H\}$ NMR spectrum of **Ar^{O}Br** (202.5 MHz, $CDCl_3$).

Synthesis of 2,6-(*t*Bu$_2$(S)PO)$_2$C$_6$H$_3$Br (Ar^{S}Br)

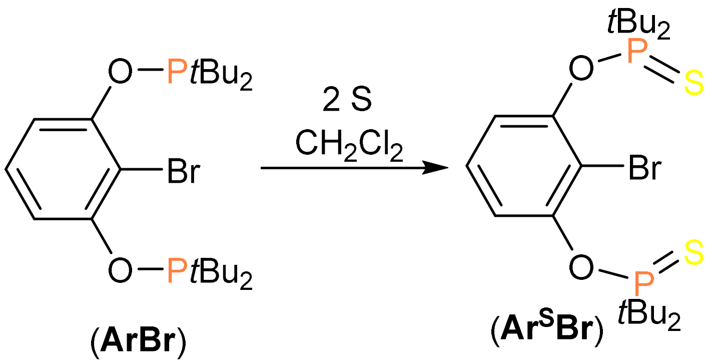

Elemental sulfur (38 mg; 1.19 mmol) was added in one portion to solution of **ArBr** (280 mg; 0.59 mmol) in dichloromethane (10 ml). The reaction mixture was stirred for 24 hours at room temperature and then was concentrated to ½ of the original volume. Yellowish solution was layered with hexane. Crystallization at room temperature gave colorless crystals of compound **Ar^{S}Br**. Yield of **Ar^{S}Br** was 285 mg, (90 %), m. p. 208-210°C. Single-crystals suitable for *sc*-XRD diffraction analysis were obtained by slow diffusion of hexane into saturated dichloromethane solution at room temperature. Anal. Calcd for $C_{22}H_{39}BrO_2P_2S_2$ (MW 541.52):

C, 48.8; H, 7.3 %. Found: C, 48.9; H, 7.4 %. **^{1}H NMR** (500 MHz, C_6D_6) δ (ppm): 1.32 [36H, d, $^3J(^{31}P,^1H)$ = 16.6 Hz, *t*Bu$_2$(S)P-C*H*$_3$], 6.74 [1H, t, $^3J(^1H,^1H)$ = 8.6 Hz, Ar-*H*], 8.31 [2H, d, $^3J(^1H,^1H)$ = 8.6 Hz, Ar-*H*]. **^{13}C{^{1}H} NMR** (125.78 MHz, C_6D_6) δ (ppm): 27.8 [s, *t*Bu$_2$P-*C*H$_3$], 42.4 [d, $^1J(^{31}P,^{13}C)$ = 58.1 Hz, *t*Bu$_2$P-*C*], 106.7 [t, $^3J(^{31}P,^{13}C)$ = 6.6 Hz], 116.6 [d, $^3J(^{31}P,^{13}C)$ = 3.9 Hz, Ar-*C*], 127.9 [s, Ar-*C*], 153.1 [d, $^2J(^{31}P,^{13}C)$ = 11.0 Hz, Ar-*C*]. **^{31}P{^{1}H} NMR** (202.5 MHz, C_6D_6) δ (ppm): 128.9 [s].

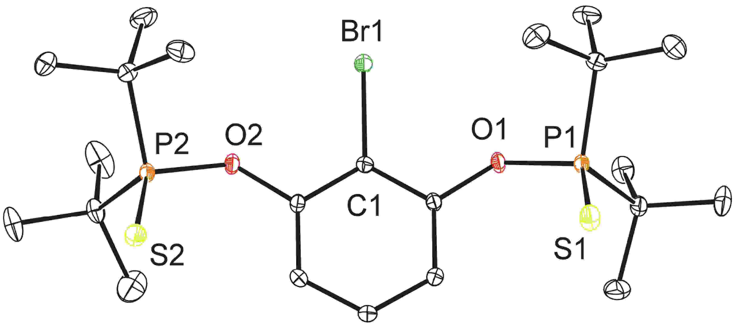

Figure S143. Molecular structure of **Ar^{S}Br**. ORTEP with 30% probability ellipsoid level. Hydrogen atoms are omitted. Selected structural bond lengths [Å]: P(1)-S(1) 1.9331(6), P(2)-S(2) 1.9422(6).

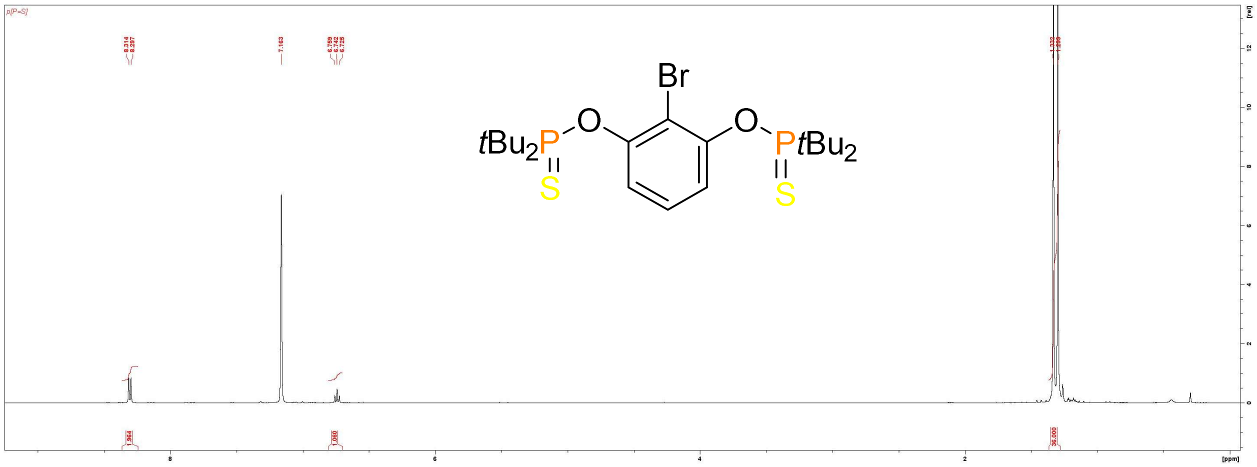

Figure S144. ^{1}H NMR spectrum of **Ar^{S}Br** (500 MHz, C_6D_6).

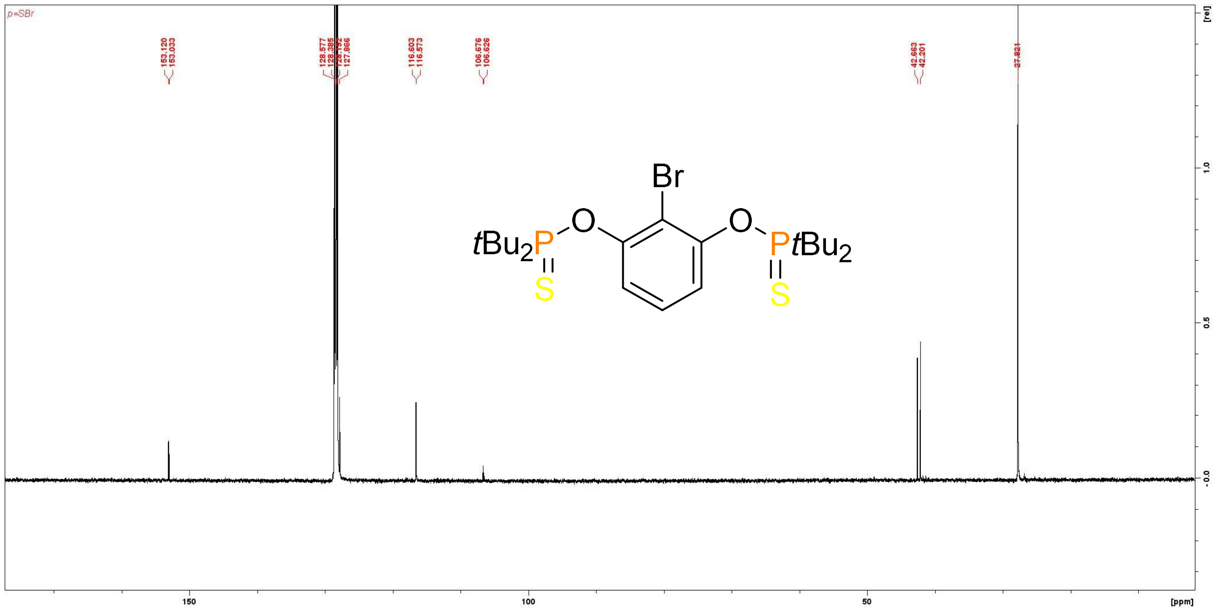

Figure S145. $^{13}C\{^1H\}$ NMR spectrum of **Ar^{S}Br** (125.76 MHz, C_6D_6).

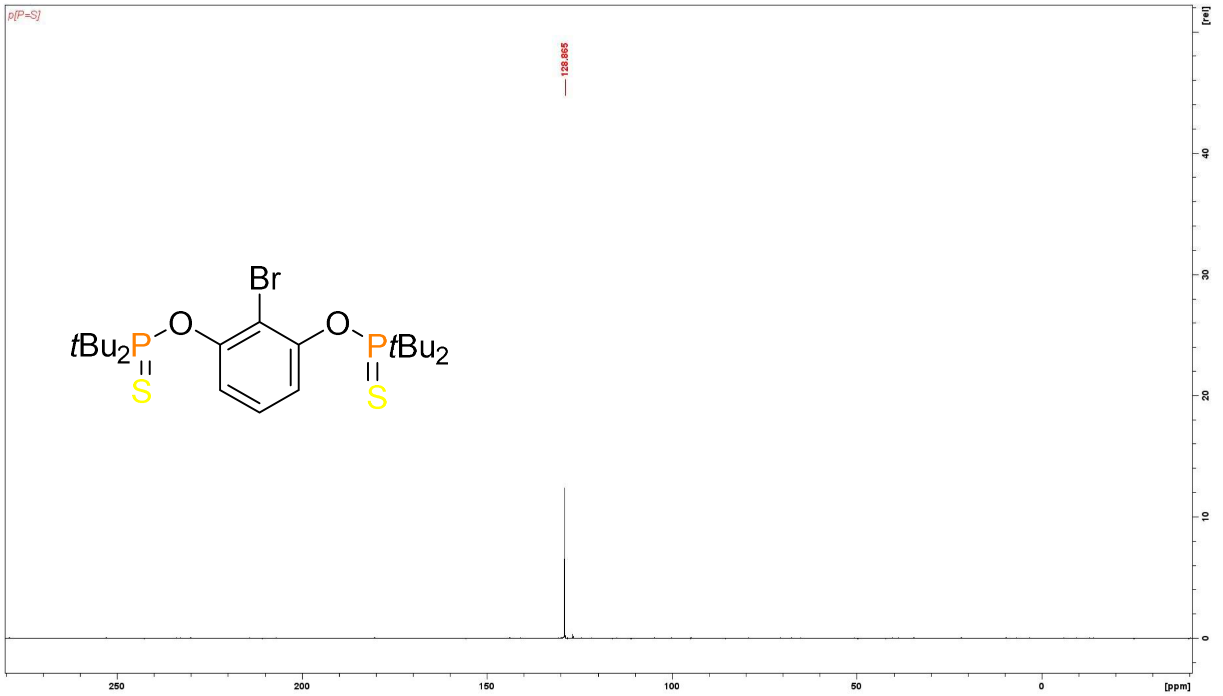

Figure S146. $^{31}P\{^1H\}$ NMR spectrum of **Ar^{S}Br** (202.5 MHz, C_6D_6).

Synthesis of 2,6-(*t*Bu$_2$(Se)PO)$_2$C$_6$H$_3$Br (Ar^{Se}Br)

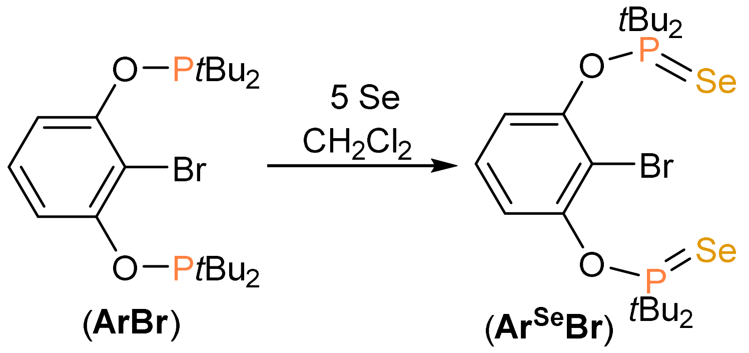

Elemental selenium (1.9 g; 24.1 mmol) was added in one portion to solution of **ArBr** (2.3 g; 4.82 mmol) in dichloromethane (40 ml). The reaction mixture was stirred for 1 h at room temperature and then unreacted selenium was removed by filtration. Resulting colorless solution was concentrated to 1/4 of the original volume and was layered with hexane. Crystallization at -30 °C gave colorless crystals of compound **Ar^{Se}Br**. Yield of **Ar^{Se}Br** was 2.04 g, (67 %), m. p. 261-263°C. Single-crystals suitable for *sc*-XRD diffraction analysis were obtained from saturated solution using dichloromethane/hexane mixture at -30°C. Anal. Calcd for $C_{22}H_{39}BrO_2P_2Se_2$ (MW 635.35): C, 41.6; H, 6.2 %. Found: C, 41.7; H, 6.4 %. **^{1}H NMR** (500 MHz, $CDCl_3$) δ (ppm): 1.48 [36H, d, $^{3}J(^{31}P,^{1}H)$ = 16.5 Hz, *t*Bu$_2$(Se)P-C*H*$_3$], 7.14 [1H, t, $^{3}J(^{1}H,^{1}H)$ = 8.2 Hz, Ar-*H*], 8.14 [2H, d, $^{3}J(^{1}H,^{1}H)$ = 8.2 Hz, Ar-*H*]. **^{13}C{^{1}H} NMR** (125.78 MHz, $CDCl_3$) δ (ppm): 27.9 [d, $^{2}J(^{31}P,^{13}C)$ = 1.9 Hz, *t*Bu$_2$(Se)P-*C*H$_3$], 43.2 [d, $^{2}J(^{31}P,^{13}C)$ = 46.5 Hz, *t*Bu$_2$(Se)P-*C*], 106.5 [t, $^{3}J(^{31}P,^{13}C)$ = 5.9 Hz, Ar-*C*], 116.0 [d, $^{3}J(^{31}P,^{13}C)$ = 3.9 Hz, Ar-*C*], 126.7 [s, Ar-*C*], 152.3 [d, $^{2}J(^{31}P,^{13}C)$ = 10.7 Hz, Ar-*C*]. **^{31}P{^{1}H} NMR** (202.5 MHz, $CDCl_3$) δ (ppm): 138.9 [s, $^{1}J(^{77}Se,^{31}P)$ = 800 Hz, *t*Bu$_2$(Se)*P*]. **^{77}Se{^{1}H} NMR** (95.4 MHz, $CDCl_3$) δ (ppm): -311.2 [d, $^{1}J(^{77}Se,^{31}P)$ = 800 Hz].

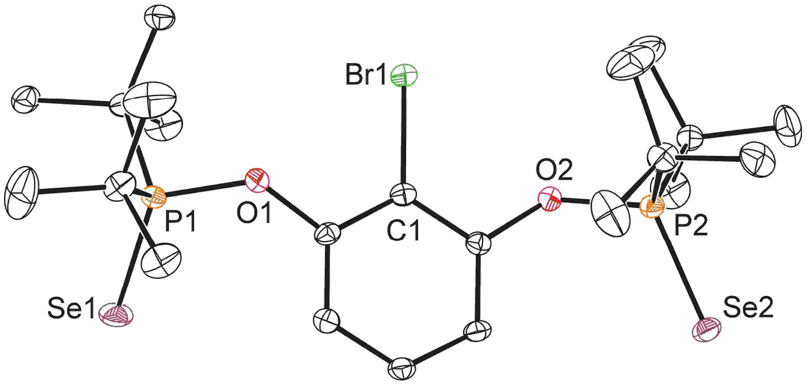

Figure S147. Molecular structure of **Ar^{Se}Br**. ORTEP with 30% probability ellipsoid level. Hydrogen atoms are omitted. Selected structural bond lengths [Å]: P(1)-Se(1) 2.0985(15), P(2)-Se(2) 2.0905(14).

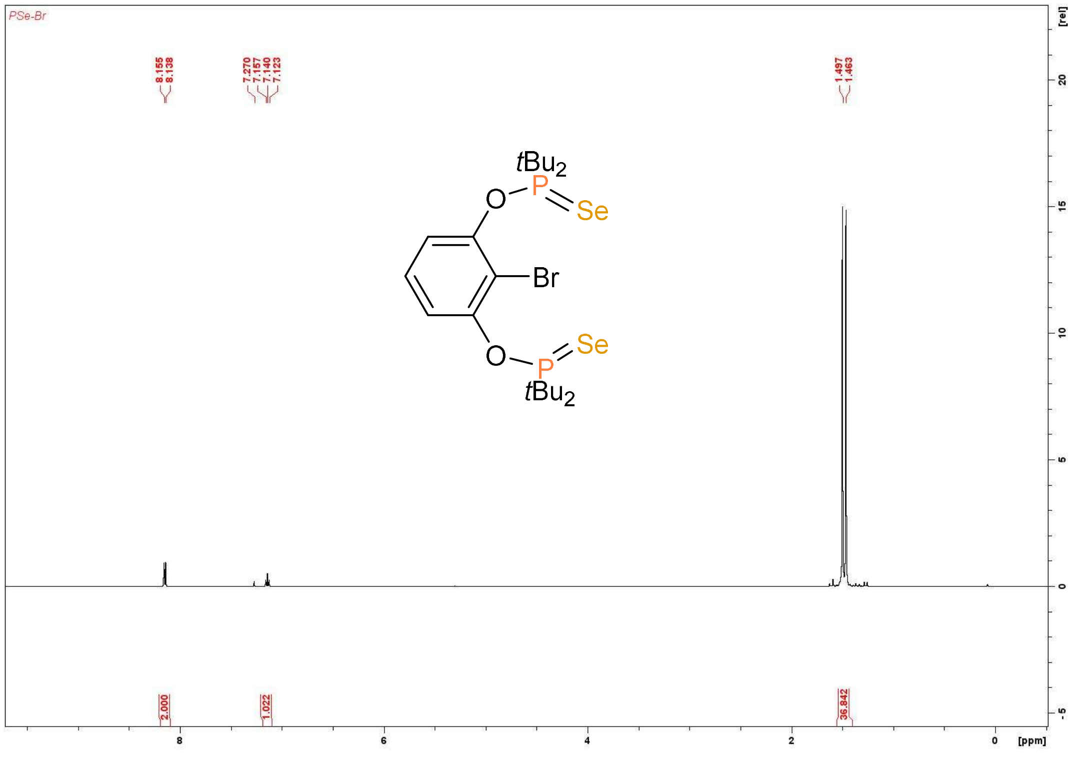

Figure S148. ^{1}H NMR spectrum of **Ar^{Se}Br** (500 MHz, $CDCl_3$).

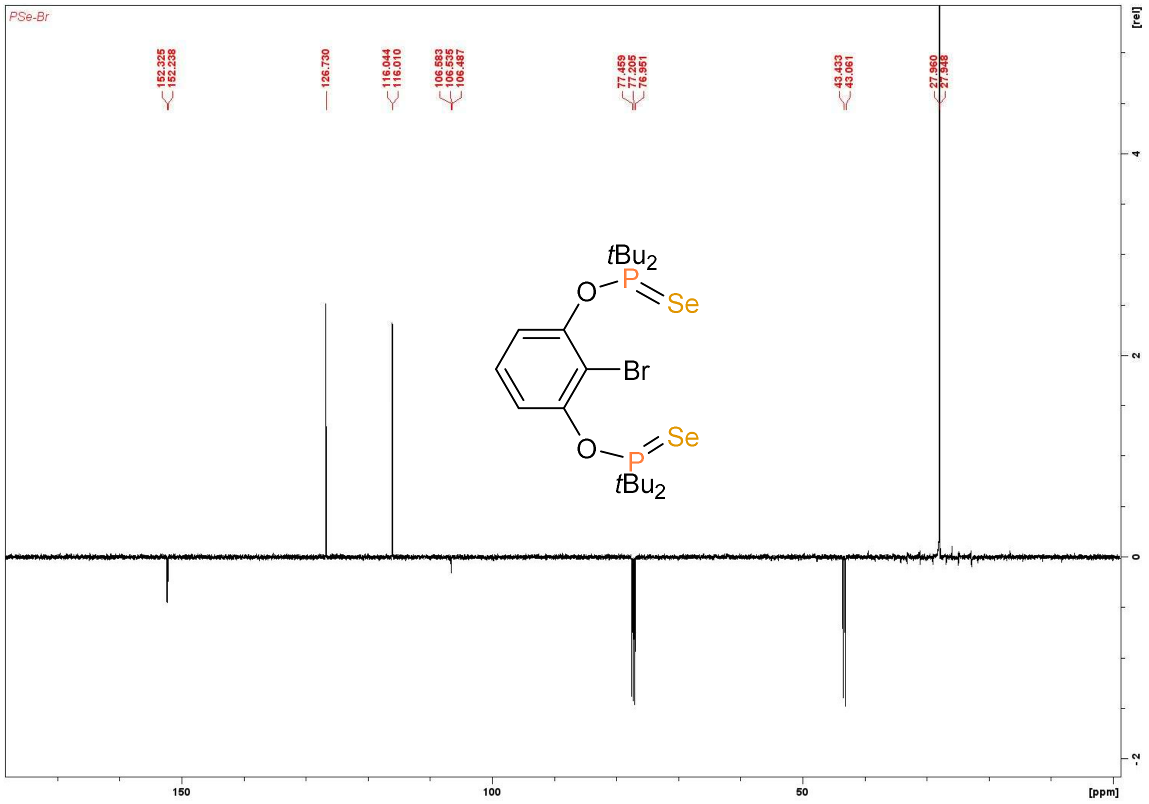

Figure S149. $^{13}C\{^1H\}$ NMR spectrum of **Ar^{Se}Br** (125.76 MHz, $CDCl_3$).

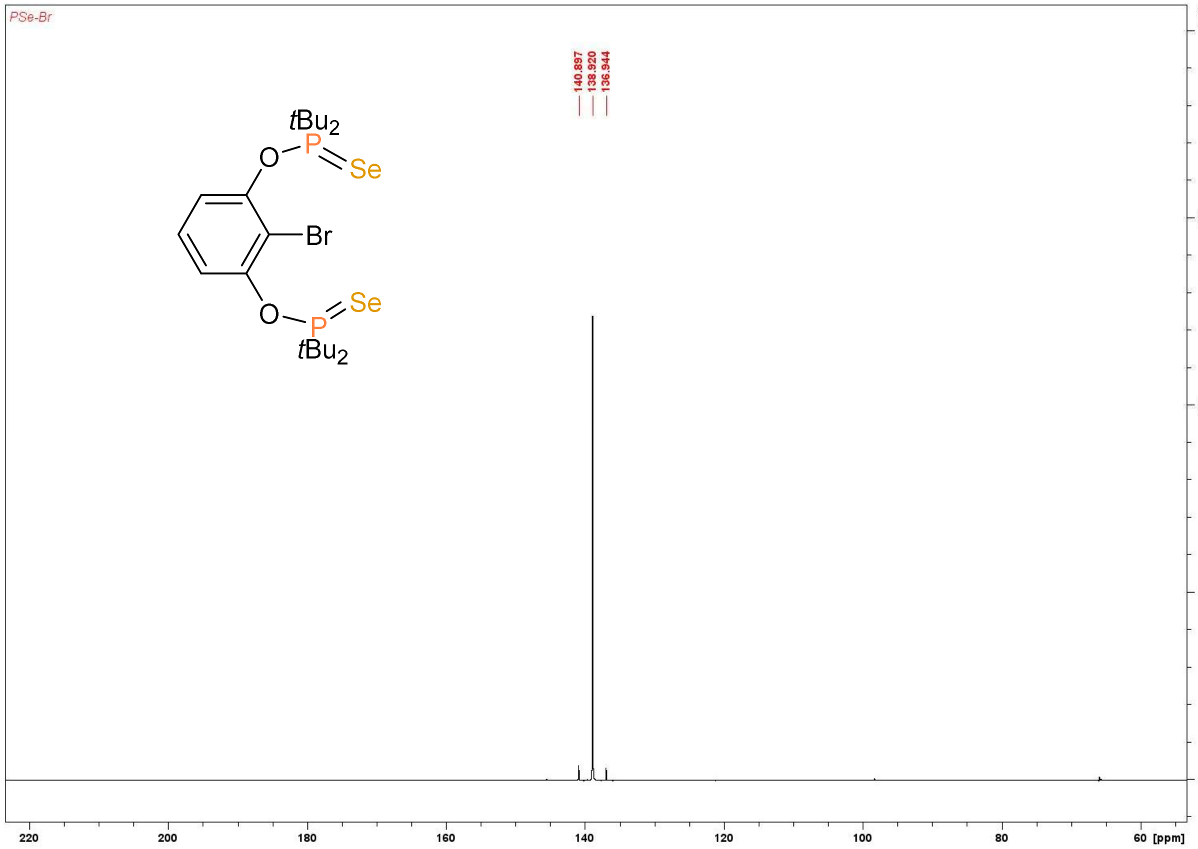

Figure S150. $^{31}P\{^1H\}$ NMR spectrum of **Ar^{Se}Br** (202.5 MHz, $CDCl_3$).

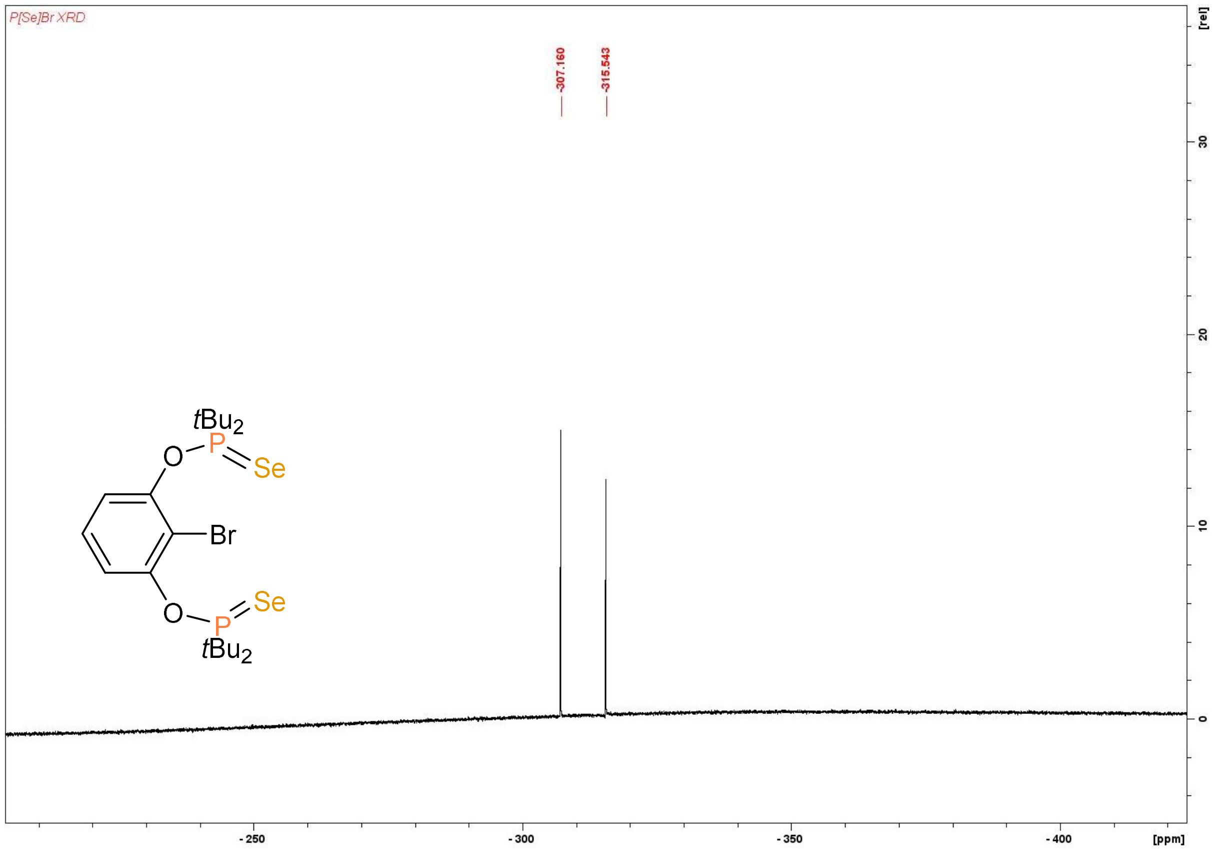

Figure S151. $^{77}Se\{^1H\}$ NMR spectrum of **Ar^{Se}Br** (95.4 MHz, $CDCl_3$).

VT-NMR experiments.

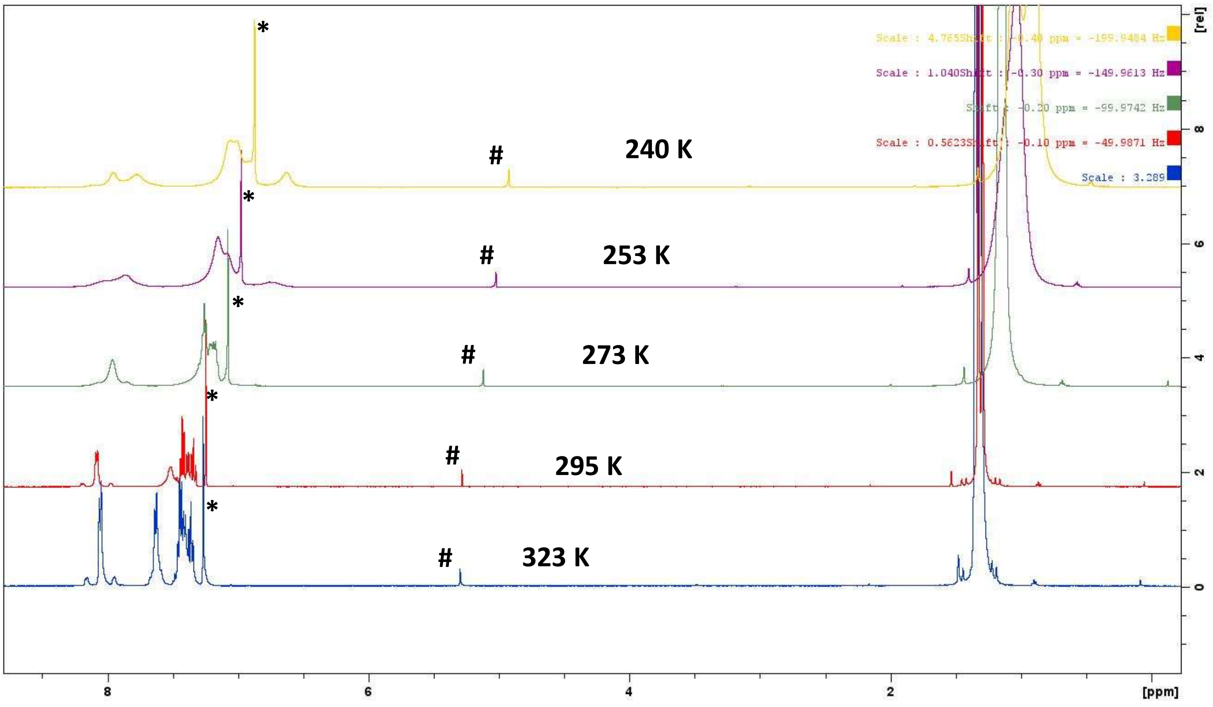

Figure S152. VT-^{1}H NMR spectra of **2**^{S} (500 MHz, $CDCl_3$(*)). $^{\#}$residual signal of CH_2Cl_2

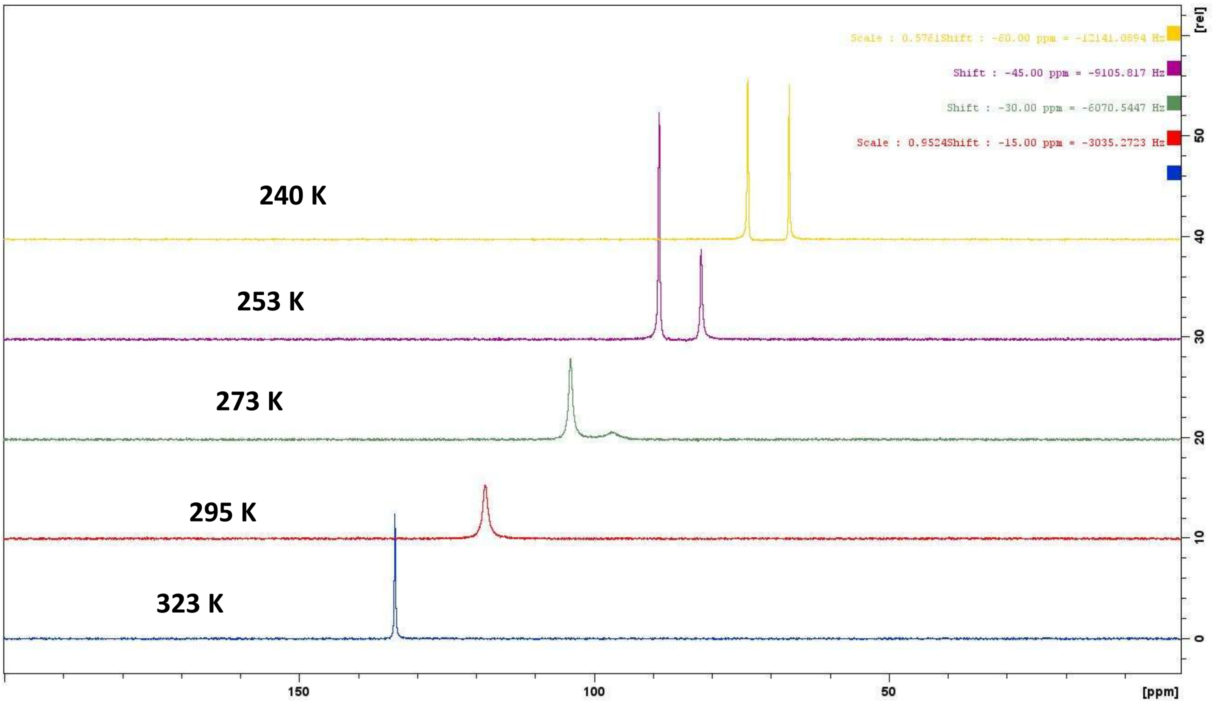

Figure S153. VT-^{31}P NMR spectra of **2**^{S} (202.5 MHz, $CDCl_3$).

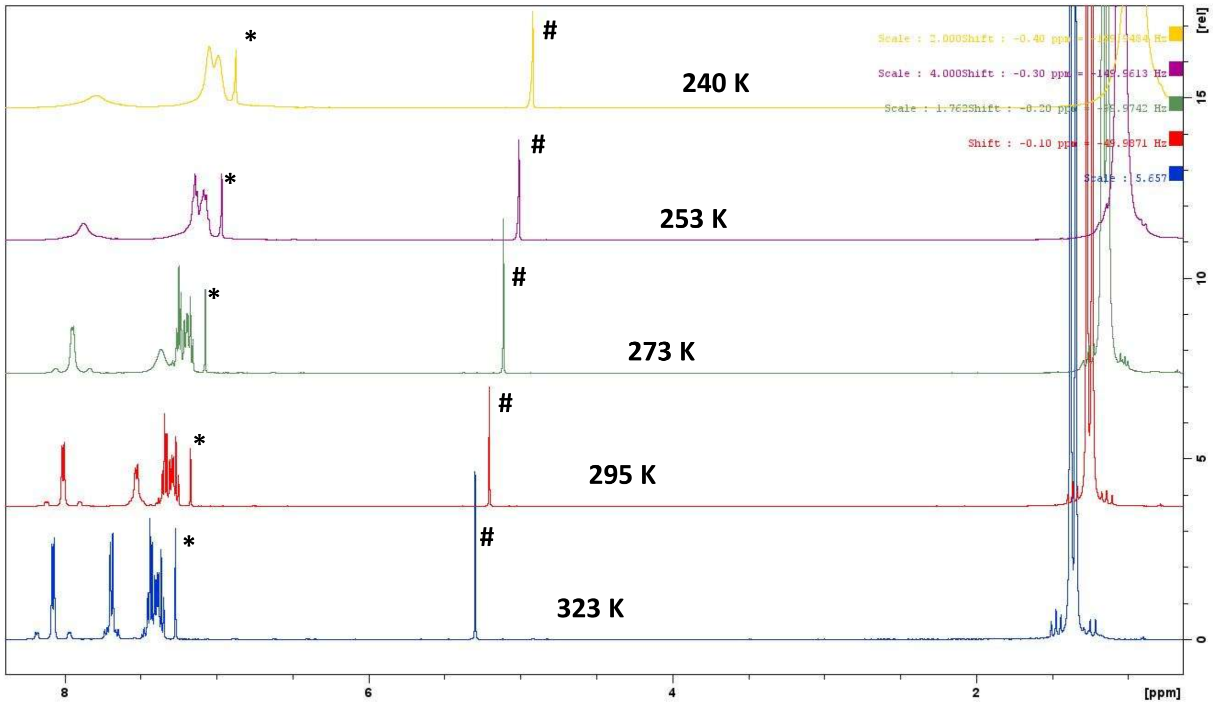

Figure S154. VT-^{1}H NMR spectra of $\mathbf{2^{Se}}$ (500 MHz, $CDCl_3$(*)). $^{\#}$residual signal of CH_2Cl_2

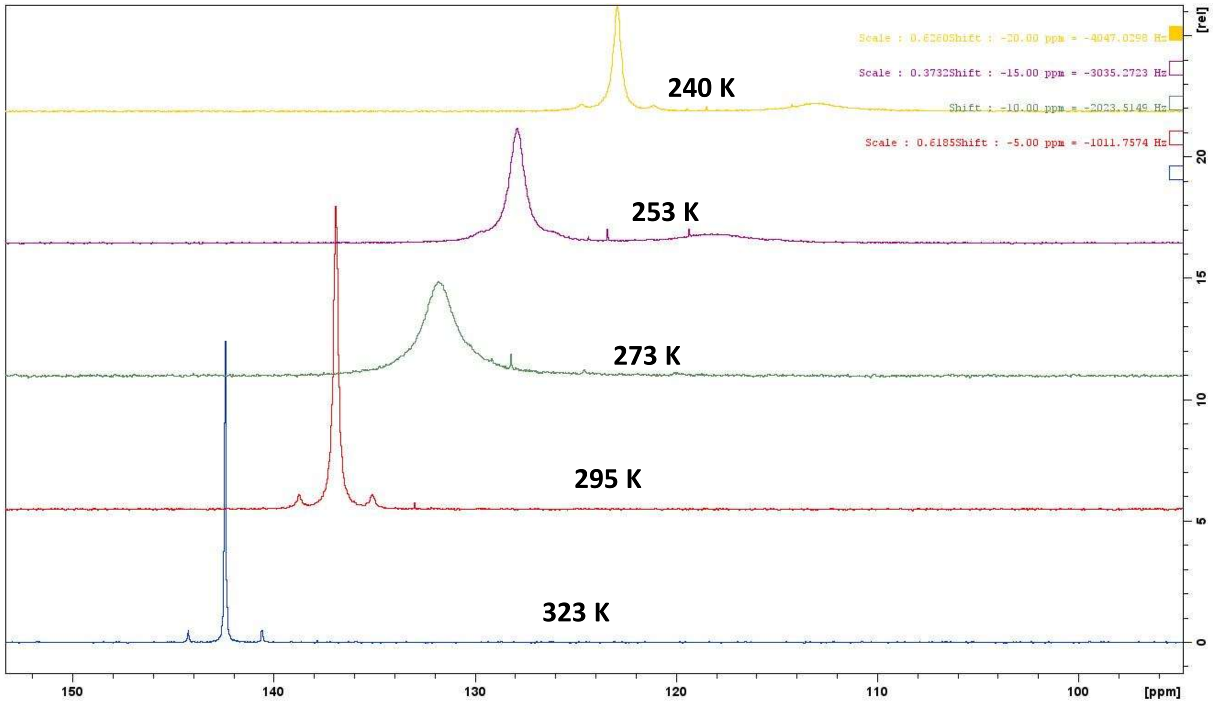

Figure S155. VT-^{31}P NMR spectra of $\mathbf{2^{Se}}$ (202.5 MHz, $CDCl_3$).

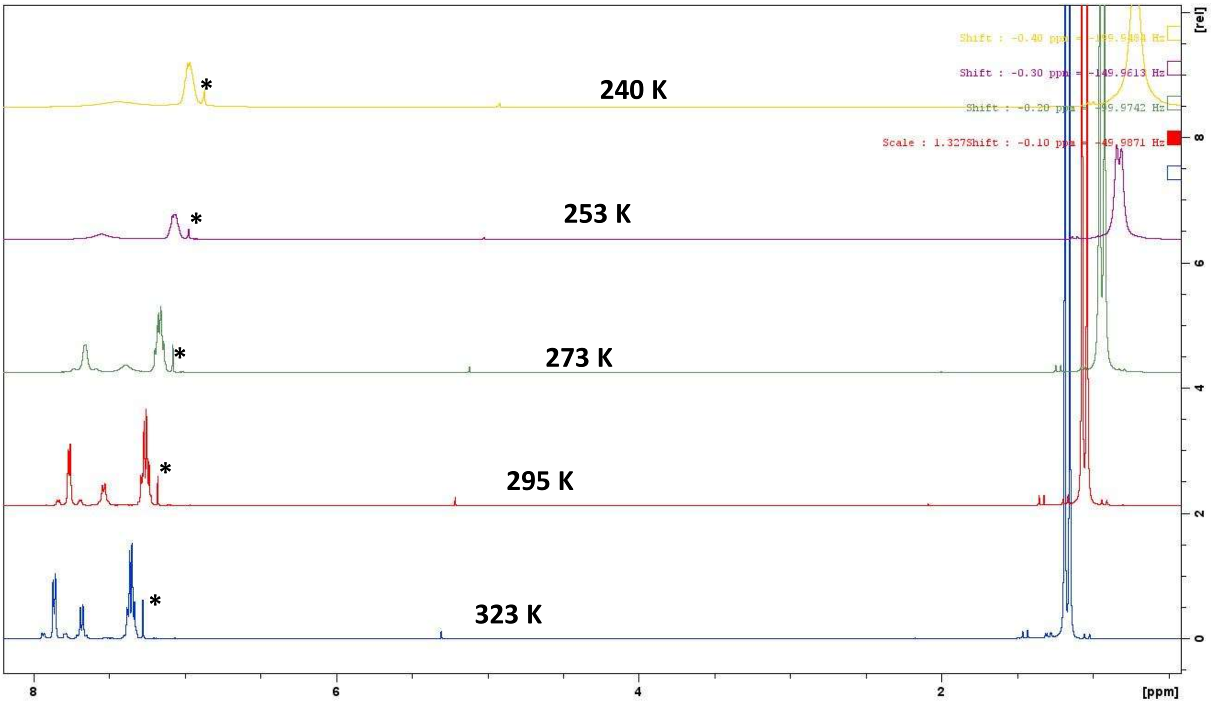

Figure S156. VT-^{1}H NMR spectra of $\mathbf{3^S}$ (500 MHz, $CDCl_3$(*)).

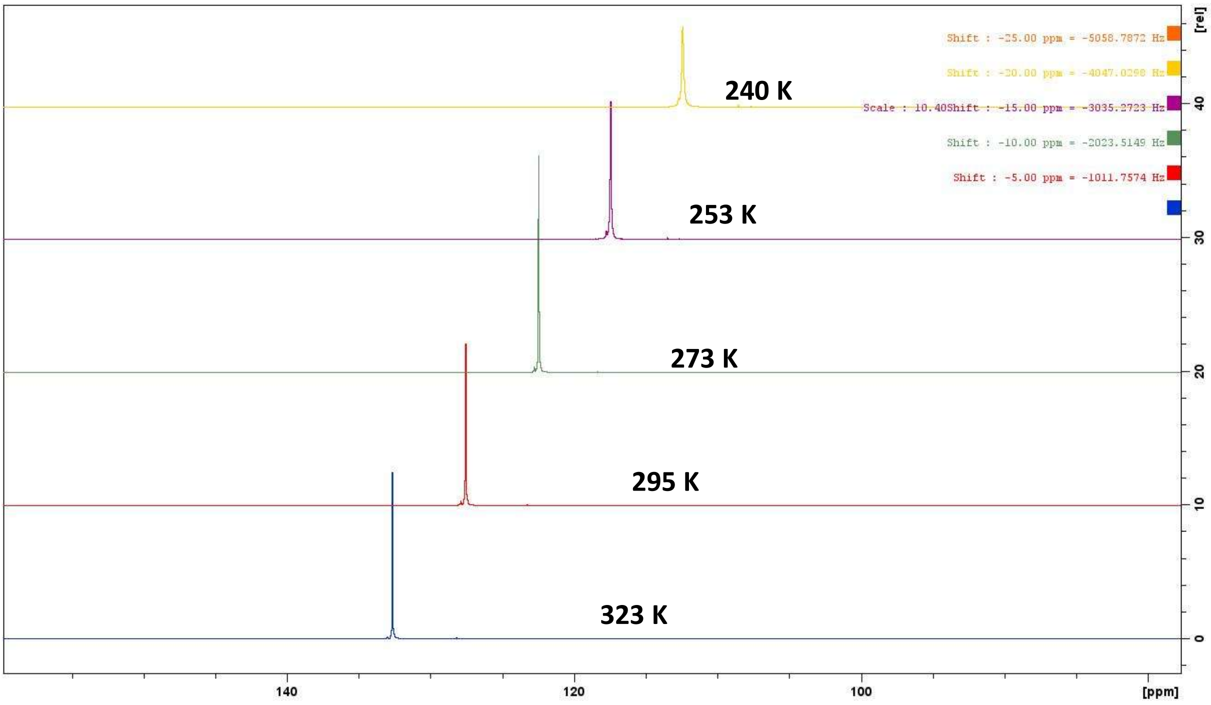

Figure S157. VT-^{31}P NMR spectra of $\mathbf{3^S}$ (202.5 MHz, $CDCl_3$).

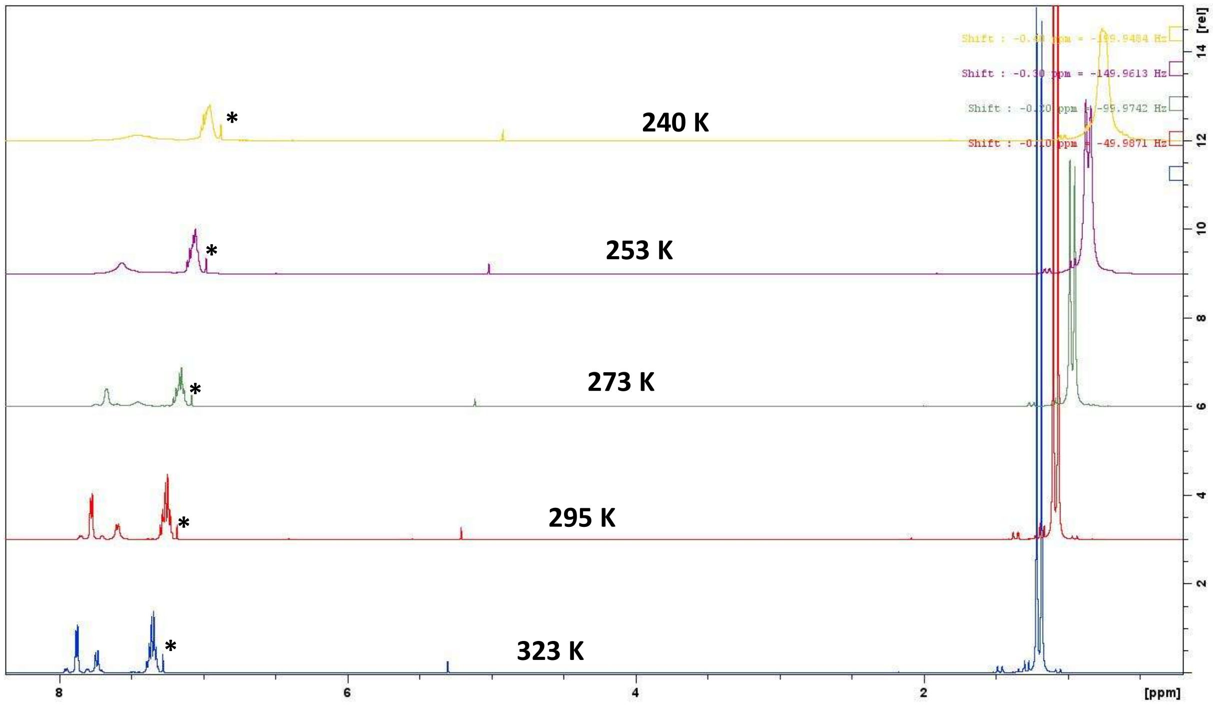

Figure S158. VT-^{1}H NMR spectra of $\mathbf{3^{Se}}$ (500 MHz, $CDCl_3$(*)).

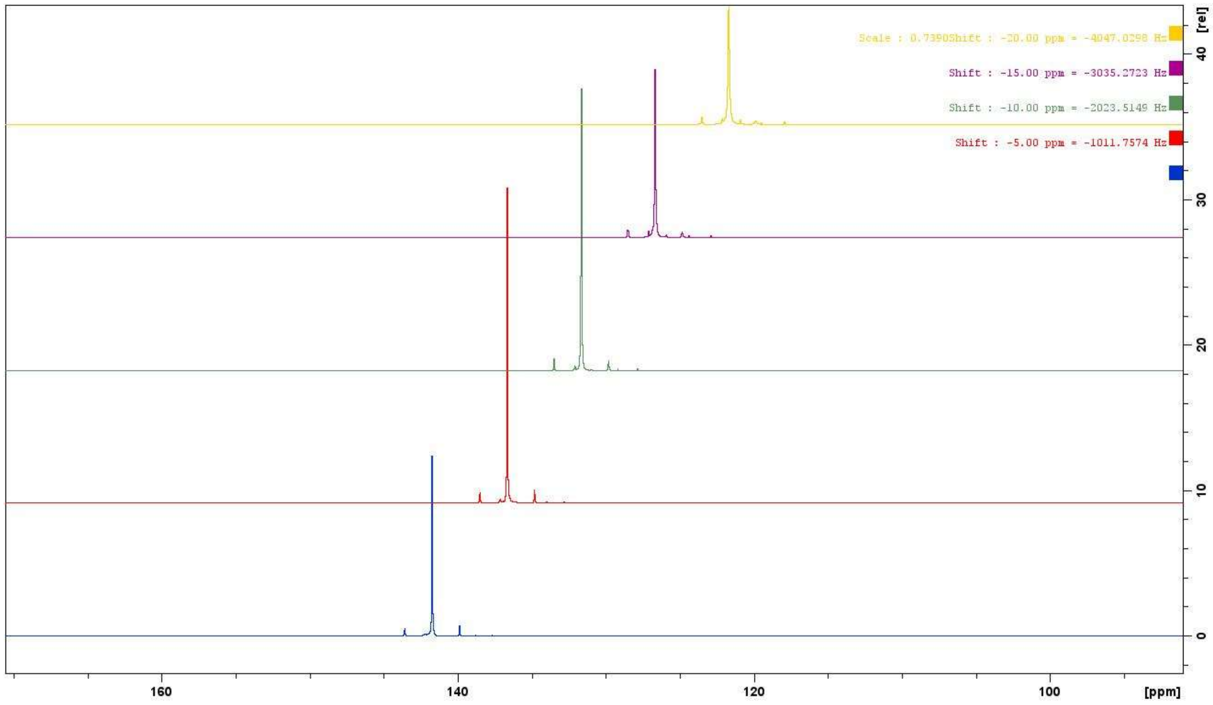

Figure S159. VT-^{31}P NMR spectra of $\mathbf{3^{Se}}$ (202.5 MHz, $CDCl_3$).

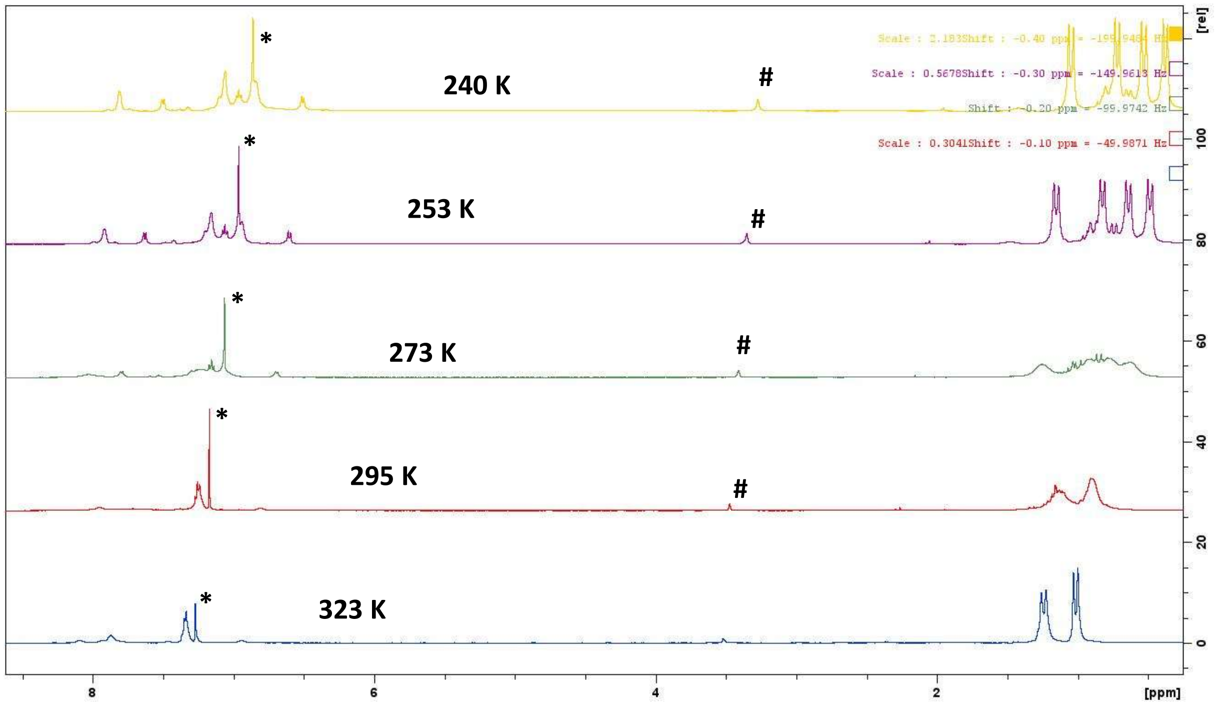

Figure S160. VT-^{1}H NMR spectra of **3OS** (500 MHz, $CDCl_3$(*)). $^{\#}$minor unknown impurity

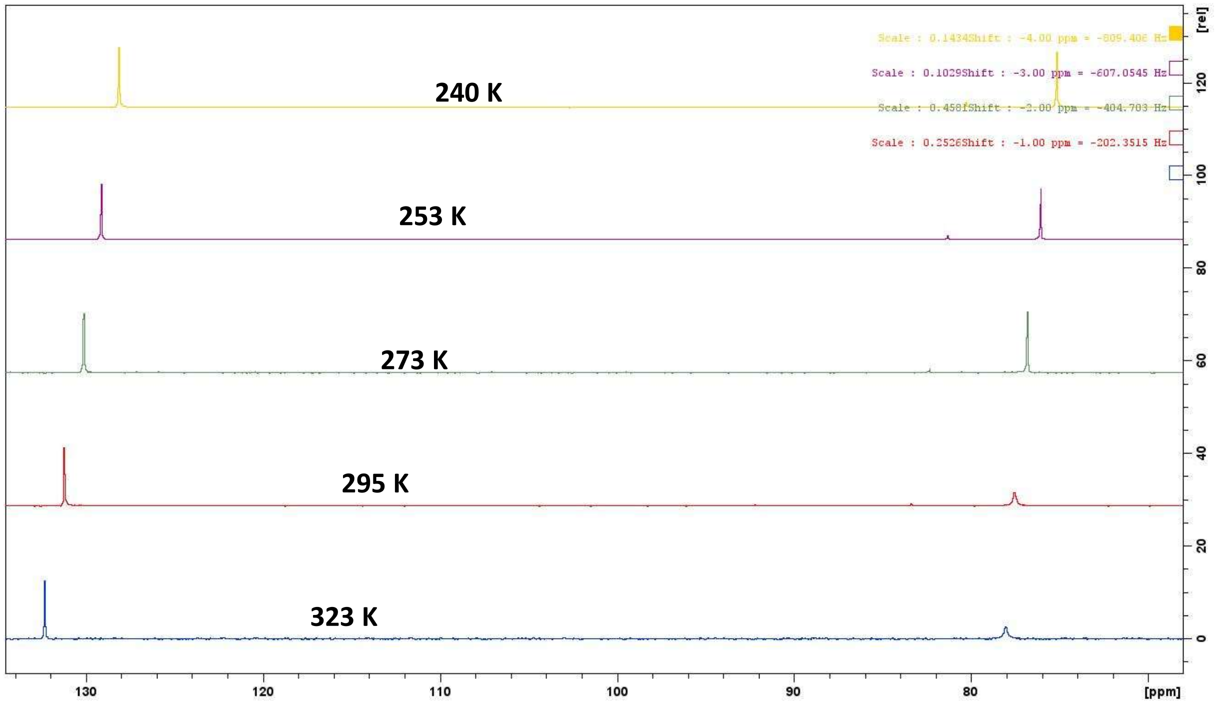

Figure S161. VT-^{31}P NMR spectra of **3OS** (202.5 MHz, $CDCl_3$).

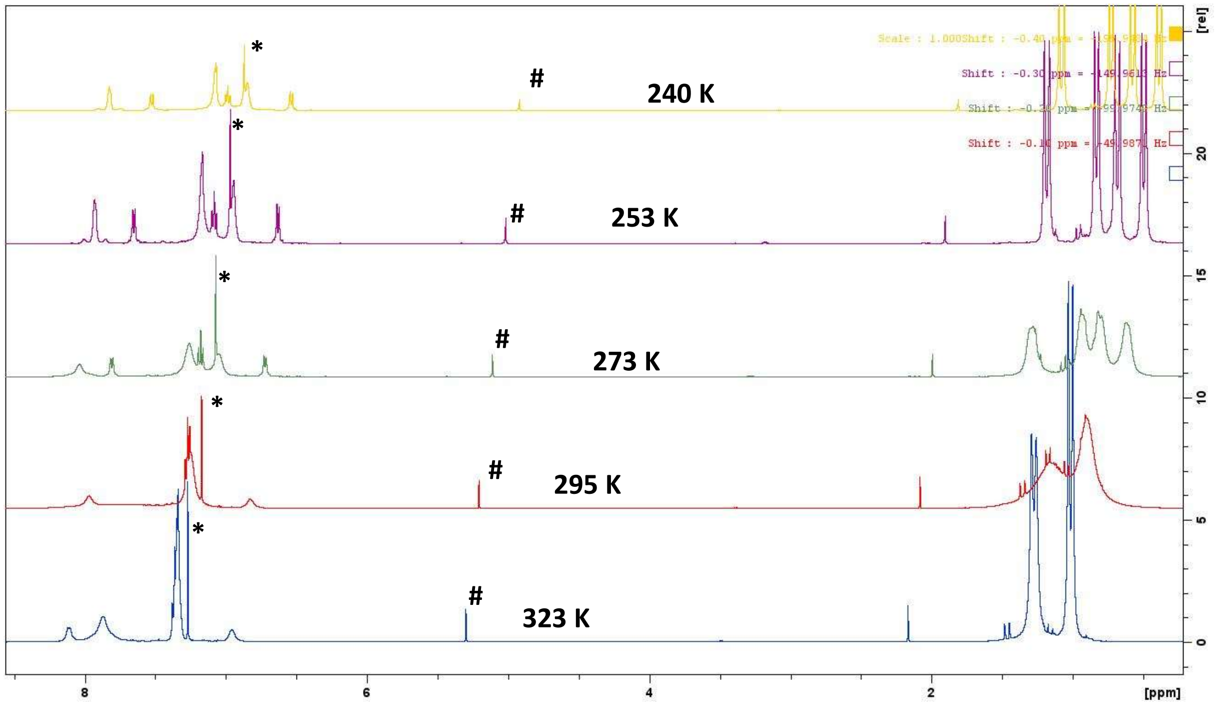

Figure S162. VT-^{1}H NMR spectra of $\mathbf{3^{OSe}}$ (500 MHz, $CDCl_3$(*)). #residual signal of CH_2Cl_2

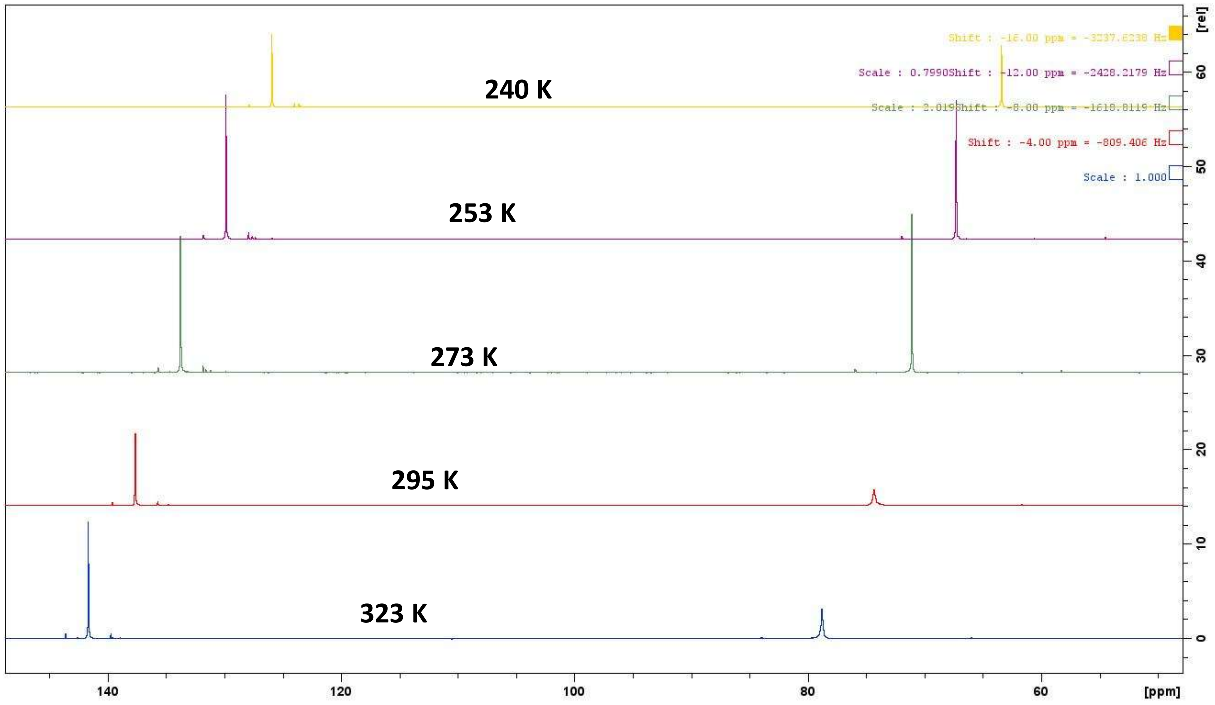

Figure S163. VT-^{31}P NMR spectra of $\mathbf{3^{OSe}}$ (202.5 MHz, $CDCl_3$).

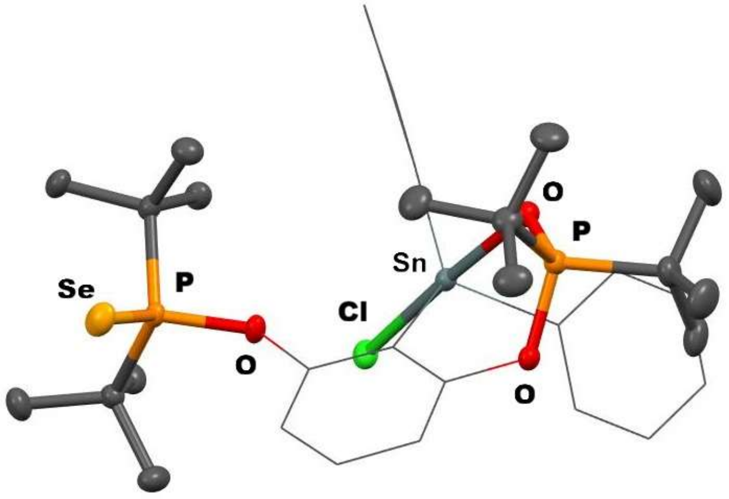

Figure S164. View on the molecular structure of $\mathbf{3^{OSe}}$, while similar one is expected to be obtained for this compound at low temperatures in solution.

Crystallographic data for studied compounds.

Full-sets of diffraction data for all studied compounds were collected at 150(2)K with a Bruker D8-Venture diffractometer equipped with Mo (Mo/K_{α} radiation; λ = 0.71073 Å) microfocus X-ray (IµS) sources, Photon CMOS detector and Oxford Cryosystems cooling device was used for data collection. The frames were integrated with the Bruker SAINT software package using a narrow-frame algorithm. Data were corrected for absorption effects using the Multi-Scan method (SADABS). Obtained data were treated by XT-version 2014/5 and SHELXL-2014/7 software implemented in APEX3 v2016.5-0 or APEX4 v2021.10-0 (Bruker AXS) system.[S1] Hydrogen atoms were mostly localized on a difference Fourier map, however to ensure uniformity of treatment of crystal, all hydrogen were recalculated into idealized positions (riding model) and assigned temperature factors $H_{iso}(H)$ = 1.2 U_{eq} (pivot atom) or of 1.5U_{eq} (methyl). Hydrogen atoms in methyl moieties and hydrogen atoms in aromatic rings were placed with C-H distances of 0.96 and 0.93Å.

Disordered CF_3 moieties of the BArF anion in $\mathbf{1^{Y+}[BArF]^{-}}$, $\mathbf{2^{Y+}[BArF]^{-}}$, $\mathbf{3^{Y+}[BArF]^{-}}$, $\mathbf{3^{PO+}[BArF]^{-}}$, $\mathbf{3^{OS+}[BArF]^{-}}$ and $\mathbf{3^{OSe+}[BArF]^{-}}$, *t*Bu of the ligand in $\mathbf{2^{O}}$, $\mathbf{2^{O+}[BArF]^{-}}$, $\mathbf{3^{Se}}$ and $\mathbf{3^{Se+}[BArF]^{-}}$ and the phenyl group in $\mathbf{3^{Se}}$ were split to two positions with equal occupancy. In the molecular structure of $\mathbf{3^{OSe+}[BArF]^{-}}$, the static positional disorder of O and Se atoms was treated by standard methods.

Molecule of $\mathbf{2^{Se}}$ crystallized together with three toluene molecules per unit cell. These disordered molecules have been masked by PLATON /SQUEZZE program,[S2] which resulted in 150 electrons per unit cell (voids of 150 electrons were found theoretically). Molecule of $\mathbf{2^{Se+}[BArF]^{-}}$ crystallized together with eight CH_2Cl_2 per unit cell. Four of them have been masked by PLATON /SQUEZZE program,[S2] which resulted in 194 electrons per unit cell (voids of 168 electrons were found theoretically).

Crystallographic data (excluding structure factors) for the structural analyses have been deposited with the Cambridge Crystallographic Data Centre, particular CCDC numbers for all

compounds are listed below in Table S1. Copies of this information may be obtained free of charge from The Director, CCDC, 12 Union Road, Cambridge CB2 1EZ, UK (Fax: +44-1223-336033; e-mail: deposit@ccdc.cam.ac.uk or http://www.ccdc.cam.ac.uk).

Table S1. Crystal data and structure refinement.

	**1**^{O}	**1**^{S}	**1**^{Se}
Formula	$C_{22}H_{39}Cl_3O_4P_2Sn$. (CH_2Cl_2)	$C_{22}H_{39}Cl_3O_2P_2S_2Sn$	$C_{22}H_{39}Cl_3O_2P_2Se_2Sn$. 2($CH_2Cl_2$)
Formula weight, g mol^{-1}	739.44	686.63	950.28
Crystal system	Monoclinic	Monoclinic	Monoclinic
Crystal size, mm	0.37 × 0.16 × 0.14	0.22 × 0.19 × 0.12	0.38 × 0.08 × 0.08
Space group	$P2_1/c$	$P2_1/c$	$P2_1/n$
a, Å	13.6533(3)	17.3504(4)	10.0646(2)
b, Å	12.8307(2)	19.0304(5)	13.6952(3)
c, Å	19.0181(3)	19.4613(5)	27.3048(5)
α, °	90	90	90
β, °	109.4780(10)	112.8670(10)	98.7280(10)
γ, °	90	90	90
V, Å^3	3140.95(10)	5920.8(3)	3720.02(13)
Z	4	8	4
ρ_{calcd}, Mg m^{-3}	1.564	1.541	1.697
μ (Mo *K*α), mm^{-1}	1.084	1.401	3.254
F(000)	1504	2800	1880
θ range, deg	1 to 28	1 to 28	1 to 28
Index ranges	$-18 \le h \le 18$	$-23 \le h \le 22$	$-13 \le h \le 13$
	$-17 \le k \le 17$	$-25 \le k \le 25$	$-18 \le k \le 18$
	$-24 \le l \le 25$	$-25 \le l \le 25$	$-36 \le l \le 36$
No. of reflns collected	95882	203776	112895
No. indep. Reflns	7756	14671	9248
No. obsd reflns with ($I>2\sigma(I)$)	7075	12938	8537
No. refined params	328	601	327
GooF (F^2)	1.130	1.101	1.041
R_1 (*F*) ($I > 2\sigma(I)$)	0.020	0.029	0.020
wR_2 (F^2) (all data)	0.046	0.065	0.044
Largest diff peak/hole, e Å^{-3}	0.484/ –0.467	1.411 / –1.342	0.700 / –0.658
CCDC	2514815	2514826	2514821

$R_{int} = \sum|F_o^2 - F_{o,mean}^2|/\sum F_o^2$, $S = [\sum(w(F_o^2 - F_c^2)^2)/(N_{diffrs} - N_{params})]^{½}$ for all data, $R(F) = \sum||F_o| - |F_c||/\sum|F_o|$ for observed data, $wR(F^2) = [\sum(w(F_o^2 - F_c^2)^2)/(\sum w(F_o^2)^2)]^{½}$ for all data.

Table S1 (continue). Crystal data and structure refinement.

	1^{O+}[BArF]$^-$	1^{S+}[BArF]$^-$	1^{Se+}[BArF]$^-$
Formula	$C_{32}H_{12}BF_{24}.C_{22}H_{39}$ $Cl_2O_4P_2Sn$	$C_{32}H_{12}BF_{24}.C_{22}H_{39}$ $Cl_2O_2P_2S_2Sn$	$C_{32}H_{12}BF_{24}.$ $C_{22}H_{39}Cl_2O_2P_2Se_2Sn$
Formula weight, g mol^{-1}	1482.28	1514.41	1608.21
Crystal system	Monoclinic	Monoclinic	Monoclinic
Crystal size, mm	0.36 × 0.16 × 0.07	0.26 × 0.21 × 0.13	0.41 × 0.22 × 0.22
Space group	C2/c	C2/c	C2/c
a, Å	17.7612(4)	17.2672(3)	17.1754(5)
b, Å	20.6344(4)	21.1600(4)	21.3481(5)
c, Å	17.0678(4)	17.3250(4)	17.3584(5)
α, °	90	90	90
β, °	104.1020(10)	103.8820(10)	102.973(2)
γ, °	90	90	90
V, Å^3	6066.7(2)	6145.2(2)	6202.2(3)
Z	4	4	4
ρ_{calcd}, Mg m^{-3}	1.623	1.637	1.722
μ (Mo $K\alpha$), mm^{-1}	0.680	0.736	1.836
F(000)	2968	3032	3176
θ range, deg	1 to 28	1 to 28	1 to 28
Index ranges	$-23 \le h \le 23$	$-23 \le h \le 23$	$-22 \le h \le 22$
	$-27 \le k \le 27$	$-28 \le k \le 28$	$-28 \le k \le 28$
	$-22 \le l \le 22$	$-23 \le l \le 23$	$-23 \le l \le 23$
No. of reflns collected	102653	105573	160834
No. indep. Reflns	7545	7559	7693
No. obsd reflns with ($I>2\sigma(I)$)	6802	6775	6492
No. refined params	489	433	462
GooF (F^2)	1.037	1.081	1.147
R_1 (F) ($I > 2\sigma(I)$)	0.032	0.039	0.033
wR_2 (F^2) (all data)	0.085	0.097	0.078
Largest diff peak/hole, e Å^{-3}	0.596 / –0.424	1.673 / –0.715	0.812 / –0.694
CCDC	2514830	2514818	2514822

$R_{int} = \sum|F_o^2 - F_{o,mean}^2|/\sum F_o^2$, $S = [\sum(w(F_o^2 - F_c^2)^2)/(N_{diffrs} - N_{params})]^{½}$ for all data, $R(F) = \sum||F_o| - |F_c||/\sum|F_o|$ for observed data, $wR(F^2) = [\sum(w(F_o^2 - F_c^2)^2)/(\sum w(F_o^2)^2)]^{½}$ for all data.

Table S1 (continue). Crystal data and structure refinement.

	$\mathbf{2^{O}}$	$\mathbf{2^{S}}$	$\mathbf{2^{Se}}$
Formula	$C_{28}H_{44}Cl_2O_4P_2Sn.(CH_2Cl_2)$	$C_{28}H_{44}Cl_2O_2P_2S_2Sn$	$C_{28}H_{44}Cl_2O_2P_2Se_2Sn.0.75(C_6H_{14})$
Formula weight, g mol^{-1}	781.09	728.28	886.71
Crystal system	Triclinic	Monoclinic	Monoclinic
Crystal size, mm	0.34 × 0.32 × 0.24	0.34 × 0.22 × 0.20	0.25 × 0.17 × 0.06
Space group	P-1	$P2_1/c$	$P2_1/c$
a, Å	10.2479(3)	26.7850(5)	15.1447(3)
b, Å	12.4766(3)	19.4585(4)	19.1593(3)
c, Å	14.7947(4)	12.8218(2)	13.1230(2)
α, °	95.7860(10)	90	90
β, °	104.5050(10)	94.1260(10)	112.2050(10)
γ, °	105.5070(10)	90	90
V, Å^3	1735.90(8)	6665.3(2)	3525.40(11)
Z	2	8	4
ρ_{calcd}, Mg m^{-3}	1.494	1.451	1.671
μ (Mo $K\alpha$), mm^{-1}	1.168	1.172	3.061
F(000)	800	2992	1790
θ range, deg	1 to 27	1 to 28	1 to 28
Index ranges	$-13 \le h \le 13$	$-35 \le h \le 35$	$-20 \le h \le 20$
	$-15 \le k \le 15$	$-25 \le k \le 25$	$-25 \le k \le 25$
	$-18 \le l \le 18$	$-17 \le l \le 17$	$-17 \le l \le 17$
No. of reflns collected	75317	180813	106532
No. indep. Reflns	7468	16422	8730
No. obsd reflns with ($I>2\sigma(I)$)	7115	15005	7616
No. refined params	465	691	346
GooF (F^2)	1.063	1.112	1.052
R_1 (F) ($I > 2\sigma(I)$)	0.023	0.021	0.024
wR_2 (F^2) (all data)	0.053	0.047	0.058
Largest diff peak/hole, e Å^{-3}	1.118 / –0.769	0.545 / –0.485	0.549 / –0.719
CCDC	2514814	2514817	2514825

$R_{int} = \sum|F_o^2 - F_{o,mean}^2|/\sum F_o^2$, S = $[\sum(w(F_o^2 - F_c^2)^2)/(N_{diffrs} - N_{params})]^{½}$ for all data, $R(F) = \sum||F_o| - |F_c||/\sum|F_o|$ for observed data, $wR(F^2) = [\sum(w(F_o^2 - F_c^2)^2)/(\sum w(F_o^2)^2)]^{½}$ for all data.

Table S1 (continue). Crystal data and structure refinement.

	$2^{O+}[BArF]^-$	$2^{S+}[BArF]^-$	$2^{Se+}[BArF]^-$
Formula	$C_{32}H_{12}BF_{24}$. $C_{28}H_{44}ClO_4P_2Sn$	$C_{32}H_{12}BF_{24}$. $C_{28}H_{44}ClO_2P_2S_2Sn$	$C_{32}H_{12}BF_{24}$. $C_{28}H_{44}ClO_2P_2Se_2Sn$. $2(CH_2Cl_2)$
Formula weight, g mol^{-1}	1523.93	1556.05	1819.71
Crystal system	Monoclinic	Monoclinic	Monoclinic
Crystal size, mm	0.29 × 0.09 × 0.08	0.37 × 0.25 × 0.20	0.45 × 0.14 × 0.10
Space group	$P2_1/c$	$P2_1/c$	$P2_1/c$
a, Å	13.6321(3)	21.8042(4)	20.2091(15)
b, Å	24.0148(7)	18.4617(4)	22.0498(16)
c, Å	20.0710(5)	17.9756(4)	17.0391(11)
α, °	90	90	90
β, °	91.3050(10)	113.2650(10)	106.904(2)
γ, °	90	90	90
V, Å^3	6569.0(3)	6647.6(2)	7264.7(9)
Z	4	4	4
ρ_{calcd}, Mg m^{-3}	1.541	1.555	1.664
μ (Mo *K*α), mm^{-1}	0.591	0.644	1.685
F(000)	3064	3128	3608
θ range, deg	1 to 28	1 to 28	1 to 26.5
Index ranges	$-18 \le h \le 18$	$-27 \le h \le 29$	$-25 \le h \le 25$
	$-32 \le k \le 32$	$-24 \le k \le 24$	$-27 \le k \le 27$
	$-26 \le l \le 26$	$-23 \le l \le 23$	$-19 \le l \le 21$
No. of reflns collected	258739	226127	222374
No. indep. Reflns	16313	16448	15025
No. obsd reflns with ($I>2\sigma(I)$)	13522	14466	11396
No. refined params	1014	904	952
GooF (F^2)	1.072	1.068	0.950
R_1 (F) ($I > 2\sigma(I)$)	0.040	0.035	0.050
wR_2 (F^2) (all data)	0.103	0.086	0.132
Largest diff peak/hole, e Å^{-3}	1.399 / –1.025	1.969 / –1.058	1.281 / –0.986
CCDC	2514820	2514827	2514823

$R_{int} = \sum|F_o^2 - F_{o,mean}^2|/\sum F_o^2$, $S = [\sum(w(F_o^2 - F_c^2)^2)/(N_{diffrs} - N_{params})]^{½}$ for all data, $R(F) = \sum||F_o| - |F_c||/\sum|F_o|$ for observed data, $wR(F^2) = [\sum(w(F_o^2 - F_c^2)^2)/(\sum w(F_o^2)^2)]^{½}$ for all data.

Table S1 (continue). Crystal data and structure refinement.

	3^{S}	3^{Se}	3^{S+}[BArF]$^{-}$
Formula	$C_{34}H_{49}ClO_2P_2S_2Sn$.(CH_2Cl_2)	$C_{34}H_{49}ClO_2P_2Se_2Sn$.(CH_2Cl_2)	$C_{32}H_{12}BF_{24}.C_{34}H_{49}O_2P_2S_2Sn$
Formula weight, g mol^{-1}	854.85	948.65	1597.70
Crystal system	Triclinic	Orthorhombic	Monoclinic
Crystal size, mm	0.59 × 0.16 × 0.10	0.39 × 0.11 × 0.09	0.27 × 0.17 × 0.11
Space group	P-1	Pbca	P2$_1$/c
a, Å	10.3001(2)	23.7834(5)	18.2979(4)
b, Å	17.0680(4)	13.7113(2)	20.3173(5)
c, Å	22.9622(5)	24.9988(5)	18.9141(4)
α, º	83.7630(10)	90	90
β, º	89.3370(10)	90	95.4690(10)
γ, º	85.5790(10)	90	90
V, Å^3	4001.05(15)	8152.1(3)	6999.6(3)
Z	4	8	4
ρ_{calcd}, Mg m^{-3}	1.419	1.546	1.516
μ (Mo $K\alpha$), mm^{-1}	1.052	2.717	0.577
F(000)	1760	3808	3224
θ range, deg	1 to 26.5	1 to 28	1 to 28
Index ranges	$-12 \le h \le 12$	$-31 \le h \le 31$	$-24 \le h \le 24$
	$-21 \le k \le 21$	$-18 \le k \le 17$	$-27 \le k \le 27$
	$-28 \le l \le 28$	$-33 \le l \le 33$	$-25 \le l \le 25$
No. of reflns collected	173794	195605	198741
No. indep. Reflns	16380	10121	17359
No. obsd reflns with ($I>2\sigma(I)$)	15455	8289	14430
No. refined params	839	504	993
GooF (F^2)	1.045	1.041	1.038
R_1 (F) ($I > 2\sigma(I)$)	0.024	0.027	0.038
wR_2 (F^2) (all data)	0.056	0.057	0.093
Largest diff peak/hole, e Å^{-3}	0.935 / –0.822	1.007 / –1.324	1.329/ –1.057
CCDC	2514819	2514829	2514831

$R_{int} = \sum|F_o^2 - F_{o,mean}^2|/\sum F_o^2$, $S = [\sum(w(F_o^2 - F_c^2)^2)/(N_{diffrs} - N_{params})]^{½}$ for all data, $R(F) = \sum||F_o| - |F_c||/\sum|F_o|$ for observed data, $wR(F^2) = [\sum(w(F_o^2 - F_c^2)^2)/(\sum w(F_o^2)^2)]^{½}$ for all data.

Table S1 (continue). Crystal data and structure refinement.

	$\mathbf{3^{Se+}[BArF]^-}$	$\mathbf{3^{PO}}$	$\mathbf{3^{OSe}}$
Formula	$C_{32}H_{12}BF_{24}$. $C_{34}H_{49}O_2P_2Se_2Sn$	$C_{34}H_{49}ClO_3P_2Sn$. (CH_2Cl_2)	$C_{34}H_{49}ClO_3P_2SeSn$. (CH_2Cl_2)
Formula weight, g mol^{-1}	1691.50	806.73	885.70
Crystal system	Monoclinic	Triclinic	Triclinic
Crystal size, mm	0.39 × 0.16 × 0.10	0.59 × 0.21 × 0.21	0.45 × 0.11 × 0.07
Space group	$P2_1/c$	P-1	P-1
a, Å	18.3635(9)	10.1843(2)	10.2331(3)
b, Å	20.2988(9)	11.6933(3)	16.9785(5)
c, Å	19.0187(7)	16.4515(3)	22.9313(7)
α, °	90	98.7750(10)	83.8710(10)
β, °	95.1050(10)	95.2680(10)	88.9590(10)
γ, °	90	92.3030(10)	84.0190(10)
V, Å^3	7061.2(5)	1925.14(7)	3939.7(2)
Z	4	2	4
ρ_{calcd}, Mg m^{-3}	1.591	1.392	1.493
μ (Mo $K\alpha$), mm^{-1}	1.544	0.987	1.889
$F(000)$	3368	832	1800
θ range, deg	1 to 28	1 to 26.5	1 to 28
Index ranges	$-24 \le h \le 24$	$-12 \le h \le 12$	$-13 \le h \le 13$
	$-27 \le k \le 27$	$-14 \le k \le 14$	$-22 \le k \le 22$
	$-24 \le l \le 25$	$-20 \le l \le 20$	$-30 \le l \le 30$
No. of reflns collected	295412	67867	179576
No. indep. Reflns	17546	7868	19535
No. obsd reflns with $(I>2\sigma(I))$	13418	7690	17242
No. refined params	1032	437	835
GooF (F^2)	1.164	1.036	1.061
R_1 (F) ($I > 2\sigma(I)$)	0.035	0.021	0.029
wR_2 (F^2) (all data)	0.074	0.061	0.067
Largest diff peak/hole, e Å^{-3}	1.310 /–0.686	0.884 /–0.625	1.488 / –1.183
CCDC	2514828	2514816	2514761

$R_{int} = \sum|F_o^2 - F_{o,mean}^2|/\sum F_o^2$, $S = [\sum(w(F_o^2 - F_c^2)^2)/(N_{diffrs} - N_{params})]^{1/2}$ for all data, $R(F) = \sum||F_o| - |F_c||/\sum|F_o|$ for observed data, $wR(F^2) = [\sum(w(F_o^2 - F_c^2)^2)/(\sum w(F_o^2)^2)]^{1/2}$ for all data.

Table S1 (continue). Crystal data and structure refinement.

	3^{PO+}[BArF]$^-$	3^{OS+}[BArF]$^-$	3^{OSe+}[BArF]$^-$
Formula	$C_{32}H_{12}BF_{24}$. $C_{34}H_{49}O_3P_2Sn$	$C_{32}H_{12}BF_{24}$. $C_{34}H_{49}O_3P_2SSn$	$C_{32}H_{12}BF_{24}$. $C_{34}H_{49}O_3P_2SeSn$
Formula weight, g mol^{-1}	1549.58	1581.64	1628.54
Crystal system	Monoclinic	Monoclinic	Monoclinic
Crystal size, mm	0.43 × 0.25 × 0.22	0.43 × 0.21 × 0.18	0.38 × 0.18 × 0.09
Space group	$P2_1/c$	$P2_1$	$P2_1/c$
a, Å	21.0354(5)	9.7733(2)	18.3383(5)
b, Å	17.8985(4)	27.3036(6)	20.4805(6)
c, Å	18.7152(4)	13.1764(3)	18.8239(4)
α, º	90	90	90
β, º	103.6420(10)	96.3560(10)	95.7910(10)
γ, º	90	90	90
V, Å^3	6847.5(3)	3494.46(13)	7033.8(3)
Z	4	2	4
ρ_{calcd}, Mg m^{-3}	1.503	1.503	1.538
μ (Mo $K\alpha$), mm^{-1}	0.530	0.549	1.034
F(000)	3128	1596	3264
θ range, deg	1 to 28	1 to 28	1 to 28
Index ranges	$-28 \le h \le 28$	$-13 \le h \le 13$	$-24 \le h \le 24$
	$-23 \le k \le 23$	$-36 \le k \le 36$	$-27 \le k \le 27$
	$-24 \le l \le 24$	$-17 \le l \le 17$	$-25 \le l \le 22$
No. of reflns collected	212438	89694	227891
No. indep. Reflns	16966	17183	17425
No. obsd reflns with ($I>2\sigma(I)$)	14264	16741	14461
No. refined params	913	979	1099
GooF (F^2)	1.056	1.026	0.937
R_1 (F) ($I > 2\sigma(I)$)	0.035	0.026	0.044
wR_2 (F^2) (all data)	0.090	0.063	0.125
Largest diff peak/hole, e Å^{-3}	1.652/ –0.644	0.648 / –0.358	1.457 / –1.200
CCDC	2514824	2514762	2514763

$R_{int} = \sum|F_o^2 - F_{o,mean}^2|/\sum F_o^2$, $S = [\sum(w(F_o^2 - F_c^2)^2)/(N_{diffrs} - N_{params})]^{½}$ for all data, $R(F) = \sum||F_o| - |F_c||/\sum|F_o|$ for observed data, $wR(F^2) = [\sum(w(F_o^2 - F_c^2)^2)/(\sum w(F_o^2)^2)]^{½}$ for all data.

Table S1 (continue). Crystal data and structure refinement.

	**Ar^O^Br**	**Ar^S^Br**	**Ar^Se^Br**
Formula	$C_{20}H_{39}BrO_4P_2$	$C_{20}H_{39}BrO_2P_2S_2$	$C_{20}H_{39}BrO_2P_2Se_2$
Formula weight, g mol^{-1}	509.38	541.50	1568.65
Crystal system	Orthorhombic	Monoclinic	Monoclinic
Crystal size, mm	0.30 × 0.25 × 0.18	0.37 × 0.13 × 0.11	0.31 × 0.14 × 0.04
Space group	$P2_12_12_1$	$P2_1/n$	$P2_1$
a, Å	11.1009(3)	14.2503(3)	8.5268(8)
b, Å	12.6503(3)	14.4528(3)	11.7480(10)
c, Å	18.6429(5)	14.4613(3)	13.6815(12)
α, °	90	90	90
β, °	90	118.6450(10)	96.634(4)
γ, °	90	90	90
V, Å^3	2618.02(12)	2613.86(10)	1361.3(2)
Z	4	4	2
ρ_{calcd}, Mg m^{-3}	1.292	1.376	1.550
μ (Mo $K\alpha$), mm^{-1}	1.715	1.871	4.317
F(000)	1072	1136	640
θ range, deg	1 to 28	1 to 28	1 to 27.5
Index ranges	$-14 \le h \le 14$	$-18 \le h \le 19$	$-11 \le h \le 11$
	$-16 \le k \le 16$	$-19 \le k \le 19$	$-15 \le k \le 15$
	$-23 \le l \le 24$	$-19 \le l \le 19$	$-17 \le l \le 17$
No. of reflns collected	26560	66629	39787
No. indep. Reflns	6459	6471	6247
No. obsd reflns with ($I>2\sigma(I)$)	5781	5576	5159
No. refined params	275	274	274
GooF (F^2)	1.028	1.022	1.019
R_1 (*F*) ($I > 2\sigma(I)$)	0.028	0.025	0.033
wR_2 (F^2) (all data)	0.060	0.058	0.059
Largest diff peak/hole, e Å^{-3}	0.289 / –0.429	0.414 / –0.445	0.425 / –0.469
CCDC	2514811	2514813	2514812

$R_{int} = \sum|F_o^2 - F_{o,mean}^2|/\sum F_o^2$, $S = [\sum(w(F_o^2 - F_c^2)^2)/(N_{diffrs} - N_{params})]^{1/2}$ for all data, $R(F) = \sum||F_o| - |F_c||/\sum|F_o|$ for observed data, $wR(F^2) = [\sum(w(F_o^2 - F_c^2)^2)/(\sum w(F_o^2)^2)]^{1/2}$ for all data.

Details for the theoretical studies.

All the calculations were carried out using the Gaussian 16 suite of programs.[S3] All structures were optimized using the ωB97X-D functional combined with the def2-SVP basis set. Single point energy calculations were performed at the ωB97X-D/def2-TZVP level of theory and the Gibbs free energies values were corrected with the entropy term obtained at the ωB97X-D/def2-SVP level. Harmonic vibrational frequency calculations were carried out to characterize the stationary points located on the potential energy hypersurface. If the stationary point is a minimum, no imaginary frequencies were obtained. To obtain WBI values and NPA charges, NBO analysis was carried out with the NBO 7.0 program.[S4] For the Atoms-in-Molecules (AIM) the Multiwfn program was employed.[S5] The Natural Bond Orbitals were visualised using by the IQmol 2.15.3. program.[S6]

Table S2. Comparison of the properties of the Y→Sn interactions in compounds $\mathbf{1^{Y}}$ (Y = O, S, Se), experimental and calculated d [Å], Y-Sn-Y bonding angle [°], WBI [-], NPA charges (q) [e] and parameters in the Y-Sn bond critical points (BCPs) in a.u., calculated at the ωB97X-D/def2-TZVP//ωB97X-D/def2-SVP level of theory.

	$\mathbf{1^{O}}$		$\mathbf{1^{S}}$		$\mathbf{1^{Se}}$	
Number of Y→Sn interactions	2		2		2	
$d_{experimental}$	2.139	2.143	2.572	2.610	2.691	2.702
$d_{calculated}$	2.174	2.174	2.663	2.677	2.759	2.776
Y-C-Y $angle_{exp}$	178.5		161.8		162.0	
Y-C-Y $angle_{calc}$	179.8		165.5		161.7	
WBI	0.25	0.25	0.43	0.43	0.49	0.48
q(Sn)	1.87		1.48		1.39	
q(Ch)	−1.14		−0.50		−0.35	
ρ	0.070	0.070	0.049	0.050	0.049	0.048
ε	0.014	0.014	0.032	0.029	0.031	0.034
$\nabla^2\rho$	0.267	0.267	0.068	0.070	0.049	0.047
η	0.198	0.198	0.283	0.283	0.316	0.316
H	−0.013	−0.013	−0.011	−0.011	−0.011	−0.011
G	0.080	0.080	0.028	0.029	0.023	0.022
V	−0.092	−0.092	−0.038	−0.040	−0.035	−0.033
|V|/G	1.161	1.161	1.384	1.389	1.477	1.471

Table S3. Comparison of the properties of the Y→Sn interactions in compounds $\mathbf{2^{Y}}$ (Y = O, S, Se), experimental and calculated d [Å], Y-Sn-Y bonding angle [°], WBI [-], NPA charges (q) [e] and parameters in the Y-Sn bond critical points (BCPs) in a.u., calculated at the ωB97X-D/def2-TZVP//ωB97X-D/def2-SVP level of theory.

	$\mathbf{2^{O}}$		$\mathbf{2^{S}}$		$\mathbf{2^{Se}}$	
Number of Y→Sn interactions	2		1		1	
$d_{experimental}$	2.192	2.200	2.844	4.649	2.933	4.679
$d_{calculated}$	2.217	2.217	3.049	4.643	3.151	4.652
Y−C−Y $angle_{exp}$	178.1					
Y−C−Y $angle_{calc}$	179.4					
WBI	0.23	0.23	0.18		0.21	
q(Sn)	1.94		1.73		1.72	
q(Ch)	−1.15		−0.63/−0.62		−0.51/−0.52	
ρ	0.063	0.063	0.024	no BCP	0.023	no BCP
ε	0.014	0.014	0.055		0.046	
$\nabla^2\rho$	0.237	0.237	0.041		0.032	
η	0.196	0.196	0.223		0.243	
H	−0.010	−0.010	−0.002		−0.002	
G	0.069	0.069	0.012		0.010	
V	−0.080	−0.080	−0.014		−0.012	
|V|/G	1.147	1.147	1.145		1.2100	

Table S4. Comparison of the properties of the Y→Sn interactions in compounds **3^Y** (Y = O, S, Se), experimental and calculated d [Å], Y-Sn-Y bonding angle [°], WBI [-], NPA charges (q) [e] and parameters in the Y-Sn bond critical points (BCPs) in a.u., calculated at the ωB97X-D/def2-TZVP//ωB97X-D/def2-SVP level of theory.

	3°		3^S		3^{Se}	
Number of Y→Sn interactions	1		1		1	
$d_{experimental}$	not observed		2.859	6.142	3.025	6.250
$d_{calculated}$	2.503	5.805	3.131	6.062	3.302	6.200
Y−C−Y $angle_{exp}$						
Y−C−Y $angle_{calc}$						
WBI	0.12		0.15		0.15	
q(Sn)	1.84		1.80		1.80	
q(Ch)	−1.16/−1.12		−0.64/−0.62		−0.53/−0.52	
ρ	0.034		0.020		0.037	
ε	0.066		0.066		0.025	
$\nabla^2\rho$	0.113		0.037		0.124	
η	0.180	no BCP	0.203	no BCP	0.181	no BCP
H	−0.002		−0.001		−0.002	
G	0.030		0.010		0.033	
V	−0.031		−0.011		−0.035	
|V|/G	1.051		1.084		1.066	

Table S5. Comparison of the properties of the Y→Sn interactions in compounds **3^{PO}**, **3OS** and **3^{OSe}**, experimental and calculated d [Å], Y-Sn-Y bonding angle [°], WBI [-], NPA charges (q) [e] and parameters in the Y-Sn bond critical points (BCPs) in a.u., calculated at the ωB97X-D/def2-TZVP//ωB97X-D/def2-SVP level of theory.

	**3^{PO}**		**3OS**		**3^{OSe}**	
Number of Y→Sn interactions	1		1		1	
Bond type	O−Sn	P−Sn	O−Sn	S−Sn	O−Sn	Se−Sn
$d_{experimental}$	2.347	4.864			2.356	6.261
$d_{calculated}$	2.517	4.764	2.469	6.058	2.464	6.166
Y−C−Y $angle_{exp}$						
Y−C−Y $angle_{calc}$						
WBI	0.12		0.13		0.13	
q(Sn)	1.83		1.84		1.84	
q	−1.15	1.09	−1.15	−0.63	−1.16	−0.53
ρ	0.033	no BCP	0.036	no BCP	0.037	no BCP
ε	0.073		0.027		0.025	
$\nabla^2\rho$	0.109		0.122		0.124	
η	0.179		0.180		0.181	
H	−0.001		−0.002		−0.002	
G	0.029		0.033		0.033	
V	−0.030		−0.035		−0.035	
|V|/G	1.045		1.064		1.066	

Table S6. Comparison of the properties of the Y→Sn interactions in compounds **1**$^{Y+}$ (Y = O, S, Se), experimental and calculated d [Å], Y-Sn-Y bonding angle [°], WBI [-], NPA charges (q) [e] and parameters in the Y-Sn bond critical points (BCPs) in a.u., calculated at the ωB97X-D/def2-TZVP//ωB97X-D/def2-SVP level of theory.

	**1**$^{O+}$		**1**$^{S+}$		**1**$^{Se+}$	
Number of Y→Sn interactions	2		2		2	
$d_{experimental}$	2.136	2.136	2.591	2.591	2.692	2.692
$d_{calculated}$	2.138	2.138	2.639	2.639	2.739	2.739
Y-C-Y $angle_{exp}$	177.4		169.9		169.6	
Y-C-Y $angle_{calc}$	174.9		172.1		171.3	
WBI	0.28	0.28	0.48	0.48	0.54	0.54
q(Sn)	1.94		1.54		1.44	
q(Ch)	−1.16		−0.52		−0.35	
ρ	0.077	0.077	0.055	0.055	0.053	0.053
ε	0.020	0.020	0.049	0.049	0.056	0.056
$\nabla^2\rho$	0.297	0.297	0.069	0.069	0.046	0.046
η	0.200	0.200	0.298	0.298	0.335	0.335
H	−0.016	−0.016	−0.013	−0.013	−0.013	−0.013
G	0.090	0.090	0.031	0.031	0.025	0.025
V	−0.107	−0.107	−0.044	−0.044	−0.038	−0.038
|V|/G	1.179	1.179	1.437	1.437	1.532	1.532

Table S7. Comparison of the properties of the Y→Sn interactions in compounds $\mathbf{2^{Y+}}$ (Y = O, S, Se), experimental and calculated d [Å], Y-Sn-Y bonding angle [°], WBI [-], NPA charges (q) [e] and parameters in the Y-Sn bond critical points (BCPs) in a.u., calculated at the ωB97X-D/def2-TZVP//ωB97X-D/def2-SVP level of theory.

	$\mathbf{2^{O+}}$		$\mathbf{2^{S+}}$		$\mathbf{2^{Se+}}$	
Number of Y→Sn interactions	2		2		2	
$d_{experimental}$	2.154	2.160	2.638	2.644	2.745	2.769
$d_{calculated}$	2.196	2.205	2.687	2.714	2.804	2.832
Y−C−Y $angle_{exp}$	177.0		170.4		170.3	
Y−C−Y $angle_{calc}$	177.3		174.6		172.9	
WBI	0.25	0.24	0.42	0.39	0.46	0.43
q(Sn)	1.98		1.65		1.58	
q(Ch)	−1.17		−0.55		−0.40	
ρ	0.066	0.067	0.046	0.049	0.044	0.046
ε	0.028	0.012	0.063	0.027	0.065	0.031
$\nabla^2\rho$	0.245	0.253	0.066	0.069	0.045	0.048
η	0.197	0.195	0.278	0.274	0.309	0.303
H	−0.011	−0.012	−0.009	−0.010	−0.009	−0.010
G	0.073	0.075	0.026	0.028	0.020	0.022
V	−0.084	−0.087	−0.035	−0.038	−0.029	−0.031
|V|/G	1.157	1.159	1.364	1.375	1.443	1.448

Table S8. Comparison of the properties of the Y→Sn interactions in compounds **3^{Y+}** (Y = O, S, Se), experimental and calculated d [Å], Y-Sn-Y bonding angle [°], WBI [-], NPA charges (q) [e] and parameters in the Y-Sn bond critical points (BCPs) in a.u., calculated at the ωB97X-D/def2-TZVP//ωB97X-D/def2-SVP level of theory.

	**3^{O+}**		**3^{S+}**		**3^{Se+}**	
Number of Y→Sn interactions	2		2		2	
$d_{experimental}$			2.696	2.707	2.810	2.826
$d_{calculated}$	2.241	2.241	2.743	2.744	2.860	2.860
Y−C−Y $angle_{exp}$			176.3		175.1	
Y−C−Y $angle_{calc}$	171.6		175.8		175.2	
WBI	0.23	0.22	0.36	0.36	0.40	0.40
q(Sn)	2.02		1.75		1.69	
q(Ch)	−1.16		−0.57		−0.43	
ρ	0.060	0.060	0.043	0.043	0.040	0.040
ε	0.025	0.022	0.045	0.045	0.045	0.045
$\nabla^2\rho$	0.225	0.224	0.067	0.067	0.048	0.048
η	0.192	0.192	0.258	0.259	0.282	0.282
H	−0.009	−0.009	−0.008	−0.008	-0.007	-0.007
G	0.065	0.065	0.024	0.024	0.019	0.019
V	−0.074	−0.074	−0.032	−0.032	-0.027	-0.027
|V|/G	1.137	1.140	1.316	1.317	1.379	1.379

Table S9. Comparison of the properties of the Y→Sn interactions in compounds $\mathbf{3^{PO+}}$, $\mathbf{3^{OS+}}$ and $\mathbf{3^{OSe+}}$, experimental and calculated d [Å], Y-Sn-Y bonding angle [°], WBI [-], NPA charges (q) [e] and parameters in the Y-Sn bond critical points (BCPs) in a.u., calculated at the ωB97X-D/def2-TZVP//ωB97X-D/def2-SVP level of theory.

	$\mathbf{3^{PO+}}$		$\mathbf{3^{OS+}}$		$\mathbf{3^{OSe+}}$	
Number of Y→Sn interactions	2		2		2	
Bond type	O−Sn	P−Sn	O−Sn	S−Sn	O−Sn	Se−Sn
$d_{experimental}$	2.216	2.730	2.225	2.707	2.236	2.803
$d_{calculated}$	2.233	2.746	2.250	2.731	2.264	2.828
Y−C−Y $angle_{exp}$	154.6		171.3		170.5	
Y−C−Y $angle_{calc}$	155.5		172.5		172.0	
WBI	0.22	0.47	0.22	0.37	0.21	0.43
q(Sn)	1.82		1.89		1.85	
q	−1.17	1.29	−1.16	−0.58	−1.15	−0.44
ρ	0.061	0.052	0.059	0.044	0.057	0.043
ε	0.021	0.007	0.028	0.046	0.029	0.052
$\nabla^2\rho$	0.230	0.049	0.219	0.069	0.210	0.051
η	0.191	0.321	0.192	0.259	0.192	0.286
H	−0.009	−0.013	−0.009	−0.008	−0.008	−0.008
G	0.067	0.025	0.063	0.025	0.060	0.021
V	−0.076	−0.037	−0.072	−0.033	−0.068	−0.029
|V|/G	1.138	1.508	1.136	1.321	1.132	1.394

Table S10. Calculated interaction energies (ΔE_{int}), interaction Gibbs free energies (ΔG_{int}) and the sum of the second order perturbation energies ($\sum E^{(2)}$) for the Y→Sn interactions in kcal/mol, calculated at the ωB97X-D/def2-TZVP//ωB97X-D/def2-SVP level of theory.

	**1^O^**	**1^S^**	**1^Se^**	**1^O+^**	**1^S+^**	**1^Se+^**
ΔE_{int}	−16.2	3.4	4.6	−88.2	−69.9	−70.4
ΔG_{int}	−7.8	10.7	11.7	−81.3	−64.0	−63.0
$\sum E^{(2)}$	−178.2	−286.1	−333.5	−184.3	−307.5	−362.1
	**2^{O}**	**2^{S}**	**2^{Se}**	**2^{O+}**	**2^{S+}**	**2^{Se+}**
ΔE_{int}	−15.3	−5.0	−4.3	−78.0	−59.9	−60.0
ΔG_{int}	−7.7	2.9	3.5	−69.8	−53.8	−52.3
$\sum E^{(2)}$	−168.9	−44.7	−48.9	−164.8	−262.5	−295.3
	**3°**	**3^{S}**	**3^{Se}**	**3^{O+}**	**3^{S+}**	**3^{Se+}**
ΔE_{int}	−16.8	−9.3	−9.3	−73.3	−57.2	−56.6
ΔG_{int}	−7.0	−0.8	−0.5	−65.2	−51.1	−49.6
$\sum E^{(2)}$	−41.0	−35.0	−33.9	−152.3	−231.8	−256.8
	**3^{OP}**	**3OS**	**3^{OSe}**	**3^{OP+}**	**3^{OS+}**	**3^{OSe+}**
ΔE_{int}	−16.7	−15.6	−15.6	−68.3	−65.6	−65.2
ΔG_{int}	−7.9	−6.7	−7.1	−60.8	−57.9	−57.7
$\sum E^{(2)}$	−39.5	−45.0	−45.6	−183.2	−186.1	−197.2

Table S11. Second Order Perturbation Theory Analysis of Fock Matrix in NBO Basis for $\mathbf{1^{O}}$. Second Order Perturbation energies in kcal/mol, s% and p% are the s and p-characters of the lone pair denoted as donor, respectively. F [a.u.] is the off-diagonal NBO Fock matrix element. Calculated at the ωB97X-D/def2-TZVP//ωB97X-D/def2-SVP level of theory.

**Donor NBO**	**Acceptor NBO**	$\mathbf{E^{(2)}}$	**s%**	**p%**	**F**
69. LP (1) O 17	154. LV (1)Sn 11	24.46	45.7	54.1	0.121
71. LP (3) O 17	154. LV (1)Sn 11	64.68	14.9	85.0	0.179
72. LP (1) O 18	154. LV (1)Sn 11	24.45	45.8	54.1	0.121
74. LP (3) O 18	154. LV (1)Sn 11	64.65	14.9	85.0	0.179

Table S12. Second Order Perturbation Theory Analysis of Fock Matrix in NBO Basis for $\mathbf{1^{S}}$. Second Order Perturbation energies in kcal/mol, s% and p% are the s and p-characters of the lone pair denoted as donor, respectively. F [a.u.] is the off-diagonal NBO Fock matrix element. Calculated at the ωB97X-D/def2-TZVP//ωB97X-D/def2-SVP level of theory.

**Donor NBO**	**Acceptor NBO**	$\mathbf{E^{(2)}}$	**s%**	**p%**	**F**
80. LP (1) S 18	162. LV (1)Sn 11	14.31	71.7	28.2	0.082
82. LP (3) S 18	162. LV (1)Sn 11	131.24	12.5	86.9	0.188
83. LP (1) S 19	162. LV (1)Sn 11	14.09	71.9	28.0	0.082
85. LP (3) S 19	162. LV (1)Sn 11	126.47	12.3	87.7	0.184

Table S13. Second Order Perturbation Theory Analysis of Fock Matrix in NBO Basis for $\mathbf{1^{Se}}$. Second Order Perturbation energies in kcal/mol, s% and p% are the s and p-characters of the lone pair denoted as donor, respectively. F [a.u.] is the off-diagonal NBO Fock matrix element. Calculated at the ωB97X-D/def2-TZVP//ωB97X-D/def2-SVP level of theory.

**Donor NBO**	**Acceptor NBO**	$\mathbf{E^{(2)}}$	**s%**	**p%**	**F**
90. LP (1)Se 12	180. LV (1)Sn 11	13.35	77.3	22.6	0.079
92. LP (3)Se 12	180. LV (1)Sn 11	156.98	11.8	87.5	0.188
98. LP (1)Se 18	180. LV (1)Sn 11	13.01	77.4	22.5	0.078
100. LP (3)Se 18	180. LV (1)Sn 11	150.12	11.6	87.8	0.183

Table S14. Second Order Perturbation Theory Analysis of Fock Matrix in NBO Basis for $\mathbf{2^{O}}$. Second Order Perturbation energies in kcal/mol, s% and p% are the s and p-characters of the lone pair denoted as donor, respectively. F [a.u.] is the off-diagonal NBO Fock matrix element. Calculated at the ωB97X-D/def2-TZVP//ωB97X-D/def2-SVP level of theory.

**Donor NBO**	**Acceptor NBO**	$\mathbf{E^{(2)}}$	**s%**	**p%**	**F**
70. LP (1) O 23	167. LV (2)Sn 7	26.35	48.9	50.9	0.127
71. LP (2) O 23	166. LV (1)Sn 7	4.22	0.2	99.5	0.039
72. LP (3) O 23	167. LV (2)Sn 7	53.91	11.9	88.0	0.161
77. LP (1) O 25	167. LV (2)Sn 7	26.35	48.9	50.9	0.127
78. LP (2) O 25	166. LV (1)Sn 7	4.22	0.2	99.5	0.039
79. LP (3) O 25	167. LV (2)Sn 7	53.89	11.9	88.0	0.161

Table S15. Second Order Perturbation Theory Analysis of Fock Matrix in NBO Basis for $\mathbf{2^{S}}$. Second Order Perturbation energies in kcal/mol, s% and p% are the s and p-characters of the lone pair denoted as donor, respectively. F [a.u.] is the off-diagonal NBO Fock matrix element. Calculated at the ωB97X-D/def2-TZVP//ωB97X-D/def2-SVP level of theory.

**Donor NBO**	**Acceptor NBO**	$\mathbf{E^{(2)}}$	**s%**	**p%**	**F**
80. LP (1) S 24	180. BD*(1) C 2-Sn 7	0.65	78.1	21.8	0.020
80. LP (1) S 24	189. BD*(1)Sn 7- C 8	1.33			0.029
80. LP (1) S 24	190. BD*(1)Sn 7-Cl 22	5.15			0.055
80. LP (1) S 24	191. BD*(1)Sn 7-Cl 23	0.75			0.021
81. LP (2) S 24	191. BD*(1)Sn 7-Cl 23	0.95	0.0	99.7	0.018
82. LP (3) S 24	180. BD*(1) C 2-Sn 7	6.33	3.4	96.2	0.049
82. LP (3) S 24	189. BD*(1)Sn 7- C 8	5.96			0.048
82. LP (3) S 24	190. BD*(1)Sn 7-Cl 22	19.22			0.081
82. LP (3) S 24	191. BD*(1)Sn 7-Cl 23	4.37			0.039

Table S16. Second Order Perturbation Theory Analysis of Fock Matrix in NBO Basis for $\mathbf{2^{Se}}$. Second Order Perturbation energies in kcal/mol, s% and p% are the s and p-characters of the lone pair denoted as donor, respectively. F [a.u.] is the off-diagonal NBO Fock matrix element. Calculated at the ωB97X-D/def2-TZVP//ωB97X-D/def2-SVP level of theory.

Donor NBO	Acceptor NBO	E(2)	s%	p%	F
98. LP (1)Se 24	207. BD*(1)Sn 7-Cl 8	4.58	82.6	17.3	0.052
98. LP (1)Se 24	208. BD*(1)Sn 7- C 17	1.12			0.027
98. LP (1)Se 24	209. BD*(1)Sn 7-Cl 23	0.61			0.019
99. LP (2)Se 24	209. BD*(1)Sn 7-Cl 23	1.10			0.019
100. LP (3)Se 24	198. BD*(1) C 2-Sn 7	7.02	0.0	99.8	0.051
100. LP (3)Se 24	207. BD*(1)Sn 7-Cl 8	22.59	4.0	95.7	0.086
100. LP (3)Se 24	208. BD*(1)Sn 7- C 17	7.21			0.052
100. LP (3)Se 24	209. BD*(1)Sn 7-Cl 23	4.65			0.039

Table S17. Second Order Perturbation Theory Analysis of Fock Matrix in NBO Basis for $\mathbf{3^{O}}$. Second Order Perturbation energies in kcal/mol, s% and p% are the s and p-characters of the lone pair denoted as donor, respectively. F [a.u.] is the off-diagonal NBO Fock matrix element. Calculated at the ωB97X-D/def2-TZVP//ωB97X-D/def2-SVP level of theory.

Donor NBO	Acceptor NBO	E(2)	s%	p%	F
63. LP (1) O 8	184. BD*(1) C 2-Sn 7	2.30	58.9	41.1	0.040
63. LP (1) O 8	193. BD*(1)Sn 7- C 12	1.78			0.035
63. LP (1) O 8	194. BD*(1)Sn 7- C 23	2.45			0.042
63. LP (1) O 8	195. BD*(1)Sn 7-Cl 29	11.14			0.086
65. LP (3) O 8	184. BD*(1) C 2-Sn 7	2.06	3.2	96.5	0.032
65. LP (3) O 8	193. BD*(1)Sn 7- C 12	5.15			0.050
65. LP (3) O 8	194. BD*(1)Sn 7- C 23	4.02			0.045
65. LP (3) O 8	195. BD*(1)Sn 7-Cl 29	12.11			0.074

Table S18. Second Order Perturbation Theory Analysis of Fock Matrix in NBO Basis for **3**^{s}. Second Order Perturbation energies in kcal/mol, s% and p% are the s and p-characters of the lone pair denoted as donor, respectively. F [a.u.] is the off-diagonal NBO Fock matrix element. Calculated at the ωB97X-D/def2-TZVP//ωB97X-D/def2-SVP level of theory.

**Donor NBO**	**Acceptor NBO**	**E$^{(2)}$**	**s%**	**p%**	**F**
78. LP (1) S 31	192. BD*(1) C 2-Sn 7	1.15	78.7	21.2	0.027
78. LP (1) S 31	201. BD*(1)Sn 7-Cl 8	5.55			0.057
78. LP (1) S 31	203. BD*(1)Sn 7- C 23	0.80			0.023
80. LP (3) S 31	192. BD*(1) C 2-Sn 7	3.84	2.4	97.2	0.039
80. LP (3) S 31	201. BD*(1)Sn 7-Cl 8	16.57			0.076
80. LP (3) S 31	202. BD*(1)Sn 7- C 9	3.73			0.038
80. LP (3) S 31	203. BD*(1)Sn 7- C 23	3.34			0.037

Table S19. Second Order Perturbation Theory Analysis of Fock Matrix in NBO Basis for **3**^{Se}. Second Order Perturbation energies in kcal/mol, s% and p% are the s and p-characters of the lone pair denoted as donor, respectively. F [a.u.] is the off-diagonal NBO Fock matrix element. Calculated at the ωB97X-D/def2-TZVP//ωB97X-D/def2-SVP level of theory.

**Donor NBO**	**Acceptor NBO**	**E$^{(2)}$**	**s%**	**p%**	**F**
99. LP (1)Se 30	219. BD*(1)Sn 7- C 17	0.90	83.7	16.3	0.024
99. LP (1)Se 30	220. BD*(1)Sn 7- C 23	0.56			0.019
99. LP (1)Se 30	221. BD*(1)Sn 7-Cl 29	4.58			0.052
100. LP (2)Se 30	220. BD*(1)Sn 7- C 23	0.77	0.0	99.8	0.017
101. LP (3)Se 30	210. BD*(1) C 2-Sn 7	3.37	2.4	97.2	0.035
101. LP (3)Se 30	219. BD*(1)Sn 7- C 17	4.07			0.039
101. LP (3)Se 30	220. BD*(1)Sn 7- C 23	2.88			0.034
101. LP (3)Se 30	221. BD*(1)Sn 7-Cl 29	16.76			0.074

Table S20. Second Order Perturbation Theory Analysis of Fock Matrix in NBO Basis for **3^{PO}**. Second Order Perturbation energies in kcal/mol, s% and p% are the s and p-characters of the lone pair denoted as donor, respectively. F [a.u.] is the off-diagonal NBO Fock matrix element. Calculated at the ωB97X-D/def2-TZVP//ωB97X-D/def2-SVP level of theory.

Donor NBO	Acceptor NBO	$E^{(2)}$	s%	p%	F
62. LP (1) O 8	174. LV (1)Sn 7	17.08	59.3	40.6	0.100
64. LP (3) O 8	174. LV (1)Sn 7	21.51	2.8	96.9	0.089
67. LP (1) O 13	174. LV (1)Sn 7	0.89	36.2	63.7	0.021

Table S21. Second Order Perturbation Theory Analysis of Fock Matrix in NBO Basis for **3OS**. Second Order Perturbation energies in kcal/mol, s% and p% are the s and p-characters of the lone pair denoted as donor, respectively. F [a.u.] is the off-diagonal NBO Fock matrix element. Calculated at the ωB97X-D/def2-TZVP//ωB97X-D/def2-SVP level of theory.

Donor NBO	Acceptor NBO	$E^{(2)}$	s%	p%	F
67. LP (1) O 8	182. LV (1)Sn 7	18.71	58.1	41.8	0.104
69. LP (3) O 8	182. LV (1)Sn 7	25.60	3.9	95.9	0.098
70. LP (1) O 18	182. LV (1)Sn 7	0.72	26.4	73.5	0.019

Table S22. Second Order Perturbation Theory Analysis of Fock Matrix in NBO Basis for **3^{OSe}**. Second Order Perturbation energies in kcal/mol, s% and p% are the s and p-characters of the lone pair denoted as donor, respectively. F [a.u.] is the off-diagonal NBO Fock matrix element. Calculated at the ωB97X-D/def2-TZVP//ωB97X-D/def2-SVP level of theory.

Donor NBO	Acceptor NBO	$E^{(2)}$	s%	p%	F
76. LP (1) O 8	191. LV (1)Sn 7	18.90	58.0	41.9	0.105
78. LP (3) O 8	191. LV (1)Sn 7	25.99	4.0	95.8	0.099
79. LP (1) O 18	191. LV (1)Sn 7	0.69	26.0	73.9	0.018

Table S23. Second Order Perturbation Theory Analysis of Fock Matrix in NBO Basis for $\mathbf{1^{O+}}$. Second Order Perturbation energies in kcal/mol, s% and p% are the s and p-characters of the lone pair denoted as donor, respectively. F [a.u.] is the off-diagonal NBO Fock matrix element. Calculated at the ωB97X-D/def2-TZVP//ωB97X-D/def2-SVP level of theory.

**Donor NBO**	**Acceptor NBO**	$\mathbf{E^{(2)}}$	**s%**	**p%**	**F**
58. LP (1) O 12	145. LV (1)Sn 11	20.14	43.4	56.5	0.108
60. LP (3) O 12	145. LV (1)Sn 11	71.99	17.8	82.0	0.189
69. LP (1) O 19	145. LV (1)Sn 11	20.14	43.4	56.4	0.108
71. LP (3) O 19	145. LV (1)Sn 11	71.99	17.8	82.0	0.189

Table S24. Second Order Perturbation Theory Analysis of Fock Matrix in NBO Basis for $\mathbf{1^{S+}}$. Second Order Perturbation energies in kcal/mol, s% and p% are the s and p-characters of the lone pair denoted as donor, respectively. F [a.u.] is the off-diagonal NBO Fock matrix element. Calculated at the ωB97X-D/def2-TZVP//ωB97X-D/def2-SVP level of theory.

**Donor NBO**	**Acceptor NBO**	$\mathbf{E^{(2)}}$	**s%**	**p%**	**F**
66. LP (1) S 12	153. LV (1)Sn 11	12.52	71.7	28.2	0.076
68. LP (3) S 12	153. LV (1)Sn 11	141.21	12.8	86.5	0.189
77. LP (1) S 19	153. LV (1)Sn 11	12.52	71.7	28.2	0.076
79. LP (3) S 19	153. LV (1)Sn 11	141.21	12.8	86.5	0.189

Table S25. Second Order Perturbation Theory Analysis of Fock Matrix in NBO Basis for $\mathbf{1^{Se+}}$. Second Order Perturbation energies in kcal/mol, s% and p% are the s and p-characters of the lone pair denoted as donor, respectively. F [a.u.] is the off-diagonal NBO Fock matrix element. Calculated at the ωB97X-D/def2-TZVP//ωB97X-D/def2-SVP level of theory.

**Donor NBO**	**Acceptor NBO**	$\mathbf{E^{(2)}}$	**s%**	**p%**	**F**
84. LP (1)Se 12	171. LV (1)Sn 11	11.99	77.8	22.1	0.073
86. LP (3)Se 12	171. LV (1)Sn 11	169.08	11.5	87.8	0.189
95. LP (1)Se 19	171. LV (1)Sn 11	11.99	77.8	22.1	0.073
97. LP (3)Se 19	171. LV (1)Sn 11	169.08	11.5	87.8	0.189

Table S26. Second Order Perturbation Theory Analysis of Fock Matrix in NBO Basis for $\mathbf{2^{O+}}$. Second Order Perturbation energies in kcal/mol, s% and p% are the s and p-characters of the lone pair denoted as donor, respectively. F [a.u.] is the off-diagonal NBO Fock matrix element. Calculated at the ωB97X-D/def2-TZVP//ωB97X-D/def2-SVP level of theory.

Donor NBO	Acceptor NBO	$E^{(2)}$	s%	p%	F
64. LP (1) O 21	157. LV (1)Sn 7	22.23	47.1	52.7	0.115
66. LP (3) O 21	157. LV (1)Sn 7	59.90	13.8	86.0	0.169
67. LP (1) O 22	157. LV (1)Sn 7	21.64	46.0	53.8	0.113
69. LP (3) O 22	157. LV (1)Sn 7	60.98	15.1	84.8	0.171

Table S27. Second Order Perturbation Theory Analysis of Fock Matrix in NBO Basis for $\mathbf{2^{S+}}$. Second Order Perturbation energies in kcal/mol, s% and p% are the s and p-characters of the lone pair denoted as donor, respectively. F [a.u.] is the off-diagonal NBO Fock matrix element. Calculated at the ωB97X-D/def2-TZVP//ωB97X-D/def2-SVP level of theory.

Donor NBO	Acceptor NBO	$E^{(2)}$	s%	p%	F
65. LP (1) S 8	165. LV (1)Sn 7	12.69	72.3	27.5	0.077
67. LP (3) S 8	165. LV (1)Sn 7	114.35	11.0	88.4	0.171
75. LP (1) S 24	165. LV (1)Sn 7	13.05	71.3	28.6	0.078
77. LP (3) S 24	165. LV (1)Sn 7	122.37	12.4	87.1	0.179

Table S28. Second Order Perturbation Theory Analysis of Fock Matrix in NBO Basis for $\mathbf{2^{Se+}}$. Second Order Perturbation energies in kcal/mol, s% and p% are the s and p-characters of the lone pair denoted as donor, respectively. F [a.u.] is the off-diagonal NBO Fock matrix element. Calculated at the ωB97X-D/def2-TZVP//ωB97X-D/def2-SVP level of theory.

Donor NBO	Acceptor NBO	$E^{(2)}$	s%	p%	F
90. LP (1)Se 23	183. LV (1)Sn 7	12.18	77.1	22.8	0.074
92. LP (3)Se 23	183. LV (1)Sn 7	141.08	11.2	88.2	0.176
93. LP (1)Se 24	183. LV (1)Sn 7	11.35	77.9	22.1	0.072
95. LP (3)Se 24	183. LV (1)Sn 7	130.67	10.3	89.1	0.168

Table S29. Second Order Perturbation Theory Analysis of Fock Matrix in NBO Basis for $\mathbf{3^{O+}}$. Second Order Perturbation energies in kcal/mol, s% and p% are the s and p-characters of the lone pair denoted as donor, respectively. F [a.u.] is the off-diagonal NBO Fock matrix element. Calculated at the ωB97X-D/def2-TZVP//ωB97X-D/def2-SVP level of theory.

Donor NBO	Acceptor NBO	$E^{(2)}$	s%	p%	F
58. LP (1) O 8	169. LV (1)Sn 7	24.40	49.4	50.4	0.120
60. LP (3) O 8	169. LV (1)Sn 7	51.97	11.5	88.4	0.154
65. LP (1) O 29	169. LV (1)Sn 7	22.61	49.4	50.4	0.043
67. LP (3) O 29	169. LV (1)Sn 7	53.33	11.9	87.9	0.157

Table S30. Second Order Perturbation Theory Analysis of Fock Matrix in NBO Basis for $\mathbf{3^{S+}}$. Second Order Perturbation energies in kcal/mol, s% and p% are the s and p-characters of the lone pair denoted as donor, respectively. F [a.u.] is the off-diagonal NBO Fock matrix element. Calculated at the ωB97X-D/def2-TZVP//ωB97X-D/def2-SVP level of theory.

Donor NBO	Acceptor NBO	$E^{(2)}$	s%	p%	F
66. LP (1) S 8	177. LV (1)Sn 7	14.08	71.8	28.1	0.082
68. LP (3) S 8	177. LV (1)Sn 7	101.71	11.0	88.5	0.165
73. LP (1) S 29	177. LV (1)Sn 7	14.08	71.8	28.1	0.082
75. LP (3) S 29	177. LV (1)Sn 7	101.97	11.1	88.4	0.166

Table S31. Second Order Perturbation Theory Analysis of Fock Matrix in NBO Basis for $\mathbf{3^{Se+}}$. Second Order Perturbation energies in kcal/mol, s% and p% are the s and p-characters of the lone pair denoted as donor, respectively. F [a.u.] is the off-diagonal NBO Fock matrix element. Calculated at the ωB97X-D/def2-TZVP//ωB97X-D/def2-SVP level of theory.

Donor NBO	Acceptor NBO	$E^{(2)}$	s%	p%	F
84. LP (1)Se 8	195. LV (1)Sn 7	13.01	77.1	22.8	0.078
86. LP (3)Se 8	195. LV (1)Sn 7	115.36	10.5	88.9	0.164
91. LP (1)Se 29	195. LV (1)Sn 7	13.01	77.1	22.8	0.078
93. LP (3)Se 29	195. LV (1)Sn 7	115.43	10.6	88.9	0.164

Table S32. Second Order Perturbation Theory Analysis of Fock Matrix in NBO Basis for $\mathbf{3^{PO+}}$. Second Order Perturbation energies in kcal/mol, s% and p% are the s and p-characters of the lone pair denoted as donor, respectively. F [a.u.] is the off-diagonal NBO Fock matrix element. Calculated at the ωB97X-D/def2-TZVP//ωB97X-D/def2-SVP level of theory.

Donor NBO	Acceptor NBO	$E^{(2)}$	s%	p%	F
57. LP (1) O 8	165. LV (1)Sn 7	24.29	48.0	51.9	0.115
58. LP (2) O 8	165. LV (1)Sn 7	1.15	0.2	99.5	0.021
59. LP (3) O 8	165. LV (1)Sn 7	54.22	13.0	86.8	0.152
62. LP (1) P 19	165. LV (1)Sn 7	103.36	54.1	45.7	0.193

Table S33. Second Order Perturbation Theory Analysis of Fock Matrix in NBO Basis for $\mathbf{3^{OS+}}$. Second Order Perturbation energies in kcal/mol, s% and p% are the s and p-characters of the lone pair denoted as donor, respectively. F [a.u.] is the off-diagonal NBO Fock matrix element. Calculated at the ωB97X-D/def2-TZVP//ωB97X-D/def2-SVP level of theory.

Donor NBO	Acceptor NBO	$E^{(2)}$	s%	p%	F
62. LP (1) O 8	173. LV (1)Sn 7	25.52	49.6	50.2	0.118
64. LP (3) O 8	173. LV (1)Sn 7	58.21	11.6	88.3	0.156
69. LP (1) S 29	173. LV (1)Sn 7	13.04	72.1	27.8	0.082
71. LP (3) S 29	173. LV (1)Sn 7	89.29	10.8	88.7	0.166

Table S34. Second Order Perturbation Theory Analysis of Fock Matrix in NBO Basis for $\mathbf{3^{OSe+}}$. Second Order Perturbation energies in kcal/mol, s% and p% are the s and p-characters of the lone pair denoted as donor, respectively. F [a.u.] is the off-diagonal NBO Fock matrix element. Calculated at the ωB97X-D/def2-TZVP//ωB97X-D/def2-SVP level of theory.

Donor NBO	Acceptor NBO	$E^{(2)}$	s%	p%	F
75. LP (1) O 28	182. LV (1)Sn 7	25.83	50.4	49.4	0.118
77. LP (3) O 28	182. LV (1)Sn 7	56.20	10.9	88.9	0.150
78. LP (1)Se 29	182. LV (1)Sn 7	12.03	77.3	22.7	0.078
80. LP (3)Se 29	182. LV (1)Sn 7	103.10	10.5	88.9	0.170

Table S35. s and p-characters of the donor atom lone pairs in the complexes **Ar^{YY}Br** (Y= P, O, S and Se), calculated at the ωB97X-D/def2-TZVP//ωB97X-D/def2-SVP level of theory.

	**Ar^{PP}Br**		**Ar^{OO}Br**		**Ar^{SS}Br**		**Ar^{SeSe}Br**	
	**s%**	**p%**	**s%**	**p%**	**s%**	**p%**	**s%**	**p%**
LP (1)	55.4	44.6	62.3	37.6	80.1	19.9	85.2	14.8
LP (2)	55.4	44.6	0.0	99.5	0.0	99.6	0.0	99.7
LP (3)			0.1	99.5	0.1	99.3	0.1	99.5

Table S36. BSSE corrected complex formation reaction energy and Gibbs free energy in kcal/mol for the model compounds at the ωB97X−D/def2−TZVP// ωB97X−D/def2−SVP level of theory.

	**Y = O**		**Y = S**		**Y = Se**	
	ΔE	ΔG	ΔE	ΔG	ΔE	ΔG
**$SnCl_4$**	−16.3	−2.4	−7.3	7.0	−8.0	6.7
**$SnPhCl_3$**	−9.6	4.0	–[a]	–[a]	−1.4	12.3
**$SnPh_2Cl_2$**	−8.3	5.4	–[a]	–[a]	–[a]	–[a]
**$SnPh_3Cl$**	–[a]	–[a]	–[a]	–[a]	–[a]	–[a]
**$SnPh_4$**	–[a]	–[a]	–[a]	–[a]	–[a]	–[a]
**$SnCl_3^+$**	−88.5	−75.4	−87.1	−73.0	−90.6	−76.1
**$SnPhCl_2^+$**	−73.2	−58.4	−69.5	−54.5	−72.2	−56.8
**$SnPh_2Cl^+$**	−62.8	−47.8	−58.1	−43.5	−60.4	−45.7
**$SnPh_3^+$**	−57.5	−42.8	−51.2	−36.4	−53.4	−37.8

[a]: No adducts could be optimized due to the spontaneous dissociation.

Total energies (in hartee) and xyz coordinates (in angstrom) of 1[O] (ωB97X-D/def2-TZVP// ωB97X-D/def2-SVP).

E = -3440.89014726

C -1.144137 3.347517 0.355949

C -1.130076 1.953387 0.337618

C -0.000058 1.192320 -0.000199

C 1.130033 1.953052 -0.338627

C 1.144248 3.347148 -0.357953

C 0.000097 4.047748 -0.001252

O -2.344382 1.384535 0.694404

P -3.112356 0.134987 0.006930

C -3.753942 0.670145 -1.647079

C -4.991667 1.567167 -1.526265

Sn 0.000032 -1.041622 0.000295

Cl -0.122012 -0.986627 2.453694

O 2.344180 1.383736 -0.695073

P 3.112314 0.134791 -0.006804

C 4.368553 -0.266926 -1.294187

C 5.358776 -1.294820 -0.724782

O -2.165553 -1.045849 -0.189883

O 2.165687 -1.045971 0.191128

Cl 0.122114 -0.987317 -2.453119

Cl -0.000000 -3.416851 0.000377

C -4.368613 -0.265926 1.294487

C -5.358120 -1.294961 0.725896

C -3.587000 -0.907166 2.454952

C -5.097791 0.985114 1.804924

C 3.753932 0.671465 1.646678

C 4.991217 1.568958 1.524878

C 2.638995 1.449195 2.370375

C 4.062945 -0.601048 2.455834

C 5.096946 0.983941 -1.806182

C 3.587038 -0.909835 -2.453793

C -4.062313 -0.603225 -2.455152

C -2.639314 1.447810 -2.371313

H 2.066487 3.857662 -0.638189

H 0.000170 5.139190 -0.001645

H -2.066349 3.858282 0.635806

H 4.301742 -1.167992 -3.250702

H 2.831531 -0.228822 -2.867615

H 3.065331 -1.823239 -2.139253

H 6.003763 -1.650836 -1.542771

H 4.836724 -2.169164 -0.308198

H 6.013985 -0.870483 0.049615

H 5.757461 0.685384 -2.635101

H 5.723897 1.459910 -1.041621

H 4.386709 1.728018 -2.192153

H 4.430705 -0.300428 3.449358

H 4.835694 -1.227665 1.989718

H 3.154919 -1.204996 2.589357

H 5.259835 1.931929 2.529102

H 4.797850 2.451089 0.895896

H 5.865594 1.035866 1.125859

H 2.971309 1.629523 3.404824

H 1.699024 0.880967 2.421768

H 2.445098 2.426153 1.907383

H -2.971600 1.627128 -3.405951

H -1.699080 0.879984 -2.422214

H -2.445925 2.425225 -1.909082

H -5.260370 1.928938 -2.530901

H -4.798745 2.450059 -0.898218

H -5.865828 1.034097 -1.126736

H -4.430361 -0.303641 -3.448880

H -4.834662 -1.229871 -1.988407

H -3.153979 -1.206803 -2.588228

H -5.758162 0.687162 2.634179

H -5.725005 1.459753 1.039769

H -4.388019 1.730113 2.189989

H -6.003203 -1.650435 1.544046

H -4.835447 -2.169467 0.310435

H -6.013292 -0.871820 -0.049190

H -4.301686 -1.164504 3.252146

H -2.831688 -0.225463 2.867983

H -3.065060 -1.820815 2.141547

Total energies (in hartee) and xyz coordinates (in angstrom) of 1^{S} (ωB97X-D/def2-TZVP// ωB97X-D/def2-SVP).

E = -4086.76693688

C -1.147964 3.131399 0.377008

C -1.118389 1.736545 0.362696

C 0.008046 0.988396 0.000064

C 1.117191 1.754495 -0.383485

C 1.100339 3.148370 -0.447508

C -0.034923 3.841939 -0.049488

O -2.298077 1.140272 0.758956
P -3.344092 0.206519 -0.062819
C -3.833170 1.110859 -1.628589
C -4.540928 2.436165 -1.323640
Sn 0.005991 -1.239978 0.005463
Cl -0.414526 -1.151711 2.420890
O 2.316137 1.185025 -0.768418
P 3.342294 0.234057 0.059859
C 3.792440 1.143106 1.634683
C 4.107300 2.625773 1.393915
Cl 0.008169 -3.639612 0.002987
S 2.601956 -1.572646 0.497682
S -2.590414 -1.579707 -0.550028
Cl 0.425148 -1.144266 -2.412862
C 4.684803 0.064202 -1.221946
C 5.666983 -1.041916 -0.807837
C 5.428351 1.386623 -1.445146
C 3.977086 -0.357126 -2.523910
C -4.655203 0.067396 1.257191
C -4.090390 -0.880367 2.331125
C -4.938511 1.432954 1.902045
C -5.938075 -0.530509 0.665688
C 2.591648 1.038709 2.593698
C 5.004870 0.437538 2.261755
C -4.747262 0.198248 -2.464626
C -2.550433 1.376964 -2.442961
H 5.164736 0.847455 3.270981
H 5.926863 0.607899 1.687109
H 4.840762 -0.645552 2.364065
H 4.354222 3.083582 2.364722
H 3.241904 3.165979 0.990972
H 4.963886 2.785536 0.729033
H 2.871924 1.521551 3.543104
H 2.306722 -0.000860 2.802183
H 1.701418 1.555419 2.208743
H -2.852922 1.699485 -3.451561
H -1.922038 0.480192 -2.552921
H -1.938421 2.178346 -2.010259
H -4.664332 2.993159 -2.265520
H -3.960045 3.069910 -0.638479
H -5.543767 2.283744 -0.899979
H -4.993651 0.726039 -3.399185
H -5.692808 -0.042237 -1.962081
H -4.240249 -0.740192 -2.728067
H -6.617370 -0.787868 1.492852
H -5.739228 -1.454729 0.101972

H -6.468312 0.178834 0.013553

H -4.801202 -0.907359 3.171838

H -3.113367 -0.544711 2.706403

H -3.962584 -1.901175 1.947726

H -5.681496 1.286546 2.701429

H -5.354155 2.164697 1.198148

H -4.031696 1.854522 2.355090

H 1.990251 3.669149 -0.802803

H -0.051175 4.932884 -0.073543

H -2.059419 3.635686 0.700897

H 4.750702 -0.572722 -3.277258

H 3.321506 0.434257 -2.908651

H 3.362337 -1.257593 -2.386979

H 6.402998 -1.168070 -1.616859

H 5.152900 -2.002699 -0.665724

H 6.221610 -0.802531 0.109325

H 6.071201 1.279151 -2.332483

H 6.081451 1.645955 -0.599653

H 4.736822 2.220287 -1.635274

Total energies (in hartee) and xyz coordinates (in angstrom) of 1^{Se} (ωB97X-D/def2-TZVP// ωB97X-D/def2-SVP).

E = -8093.59054940

C 1.144435 3.253099 0.382343

C 1.113216 1.857999 0.372336

C -0.007910 1.108289 -0.003949

C -1.107903 1.876163 -0.409940

C -1.084315 3.269442 -0.488902

C 0.043406 3.964144 -0.072782

O 2.284654 1.262997 0.794288

P 3.394007 0.385882 -0.007984

C 3.877164 1.317394 -1.565364

C 2.603426 1.529461 -2.409437

Sn -0.007937 -1.126151 0.011596

Se -2.689665 -1.558812 0.493112

P -3.389375 0.417835 0.010010

C -4.713678 0.315097 -1.303795

C -5.766267 -0.732591 -0.913469

O -2.304249 1.310651 -0.809043

Cl 0.397914 -1.020719 2.433926

Se 2.676850 -1.570318 -0.536080

Cl -0.010269 -3.548145 0.017266
Cl -0.413600 -1.031283 -2.414136
C -3.826010 1.360778 1.574307
C -4.091074 2.850903 1.319552
C -2.638830 1.229631 2.547477
C -5.068719 0.703081 2.193467
C 4.680532 0.321984 1.347551
C 4.884957 1.708185 1.978322
C 6.003665 -0.224218 0.797550
C 4.131641 -0.632600 2.424508
C -5.378929 1.675778 -1.544461
C -3.999921 -0.142876 -2.590470
C 4.521563 2.672010 -1.247799
C 4.851908 0.448849 -2.379306
H -1.964670 3.789429 -0.868244
H 0.062881 5.054826 -0.104253
H 2.048399 3.757712 0.725922
H -4.767763 -0.321812 -3.359217
H -3.431551 -1.071801 -2.442315
H -3.299610 0.615820 -2.961683
H -6.499570 -0.803475 -1.731675
H -6.315579 -0.470178 0.000732
H -5.316830 -1.726614 -0.779431
H -5.996329 1.605195 -2.453329
H -4.638979 2.472732 -1.707214
H -6.045704 1.965174 -0.719812
H -5.228606 1.132611 3.194520
H -4.943361 -0.383361 2.314618
H -5.976535 0.895888 1.603509
H -4.324295 3.324009 2.286426
H -4.941330 3.033125 0.652760
H -3.208059 3.359486 0.914679
H -2.914080 1.733134 3.487595
H -1.728810 1.713265 2.166111
H -2.388016 0.184894 2.774215
H 5.112874 0.999371 -3.296684
H 4.389162 -0.502471 -2.676209
H 5.786939 0.233672 -1.846170
H 2.916155 1.859114 -3.412637
H 1.951602 2.308259 -1.994353
H 2.010913 0.609705 -2.529903
H 4.637113 3.233232 -2.188211
H 5.522721 2.562545 -0.807401
H 3.902867 3.281096 -0.574285
H 4.803675 -0.587152 3.295799
H 4.092603 -1.670578 2.069045

H 3.117522 -0.355907 2.747051

H 5.617566 1.607525 2.794199

H 3.950451 2.091300 2.408523

H 5.279878 2.449905 1.273390

H 6.672548 -0.439025 1.645145

H 6.517568 0.499665 0.148308

H 5.863214 -1.164764 0.242873

Total energies (in hartee) and xyz coordinates (in angstrom) of 1^{O+} (ωB97X-D/def2-TZVP// ωB97X-D/def2-SVP).

E = -2980.44700807

C -1.154634 -1.904205 -0.300186

C -1.161194 -3.296778 -0.310382

C -0.000015 -3.991732 -0.000273

C 1.161178 -3.296843 0.309934

C 1.154645 -1.904269 0.299932

C 0.000002 -1.152864 -0.000080

O 2.358626 -1.333634 0.642681

P 3.135699 -0.053286 0.006844

C 4.351486 0.307274 1.335459

C 5.347760 1.366404 0.837825

Sn -0.000000 1.004745 0.000071

O -2.130676 1.100257 0.146468

P -3.135678 -0.053288 -0.006844

C -4.351462 0.307486 -1.335404

C -5.347727 1.366547 -0.837608

O -2.358605 -1.333500 -0.642855

Cl -0.085061 2.151332 -2.031518

Cl 0.085058 2.151047 2.031821

O 2.130675 1.100282 -0.146312

C 3.756620 -0.501243 -1.670257

C 4.899391 -1.520802 -1.592535

C 2.574983 -1.096707 -2.460084

C 4.208349 0.793937 -2.370821

C -3.756641 -0.501520 1.670167

C -4.899408 -1.521068 1.592249

C -2.575024 -1.097111 2.459927

C -4.208391 0.793545 2.370931

C 5.082597 -0.964592 1.792920

C 3.526817 0.878597 2.504372

C -5.082581 -0.964294 -1.793093

C -3.526794 0.879026 -2.504210
H 2.089310 -3.812313 0.557890
H -0.000025 -5.082921 -0.000349
H 4.212796 1.091940 3.337532
H 2.775130 0.161254 2.864319
H 3.014117 1.809986 2.231095
H 5.956625 1.699268 1.691498
H 4.839908 2.252312 0.428322
H 6.037963 0.967156 0.080878
H 5.746627 -0.695341 2.628026
H 5.708677 -1.404915 1.006647
H 4.380707 -1.728303 2.154112
H 4.519101 0.540275 -3.395561
H 5.064429 1.270495 -1.876538
H 3.387661 1.522081 -2.432415
H 5.152668 -1.845580 -2.612907
H 4.618625 -2.416826 -1.018814
H 5.810242 -1.090876 -1.152873
H 2.905306 -1.253874 -3.497819
H 1.712640 -0.414632 -2.495370
H 2.249621 -2.069944 -2.067732
H -2.089334 -3.812197 -0.558410
H -4.212770 1.092504 -3.337338
H -2.775093 0.161758 -2.864278
H -3.014110 1.810376 -2.230767
H -5.956577 1.699560 -1.691234
H -4.839868 2.252390 -0.427969
H -6.037934 0.967189 -0.080723
H -5.746603 -0.694891 -2.628155
H -5.708670 -1.404750 -1.006902
H -4.380696 -1.727948 -2.154415
H -4.519169 0.539715 3.395622
H -5.064469 1.270172 1.876715
H -3.387705 1.521680 2.432666
H -5.152711 -1.846013 2.612561
H -4.618632 -2.416990 1.018374
H -5.810254 -1.091066 1.152650
H -2.905373 -1.254448 3.497628
H -1.712682 -0.415041 2.495347
H -2.249650 -2.070283 2.067424

Total energies (in hartee) and xyz coordinates (in angstrom) of 1^{S+} (ωB97X-D/def2-TZVP// ωB97X-D/def2-SVP).

E = -3626.32568270

C 1.124711 -1.659877 -0.394946
C 1.123009 -3.052097 -0.433154
C -0.000021 -3.746824 0.000001
C -1.123041 -3.052074 0.433148
C -1.124720 -1.659854 0.394924
C -0.000011 -0.920343 -0.000004
O -2.281389 -1.038383 0.805246
P -3.363634 -0.180802 -0.079077
C -4.544927 0.278305 1.284616
C -3.815564 1.292623 2.180218
Sn 0.000006 1.231701 -0.000004
S 2.461362 1.413877 0.933935
P 3.363633 -0.180830 0.079061
C 4.544935 0.278279 -1.284623
C 3.815588 1.292617 -2.180214
O 2.281390 -1.038429 -0.805276
Cl -0.529174 2.467173 1.924514
Cl 0.529239 2.467165 -1.924513
S -2.461347 1.413915 -0.933943
C -4.035092 -1.252827 -1.456436
C -4.958031 -0.396307 -2.343621
C -4.799702 -2.459095 -0.896121
C -2.853539 -1.733796 -2.321637
C 4.035053 -1.252831 1.456457
C 4.957959 -0.396294 2.343658
C 4.799687 -2.459103 0.896184
C 2.853475 -1.733794 2.321628
C -4.917506 -0.952786 2.126649
C -5.797115 0.929943 0.681389
C 4.917529 -0.952815 -2.126648
C 5.797114 0.929927 -0.681388
H -2.016666 -3.572861 0.778499
H -0.000030 -4.837971 0.000008
H -6.383986 1.370699 1.500828
H -6.444177 0.201511 0.173381
H -5.549292 1.742742 -0.018048
H -4.489680 1.562055 3.006999
H -3.551157 2.211201 1.640118
H -2.899169 0.872741 2.616614
H -5.553430 -0.610958 2.957092
H -4.030455 -1.432604 2.560923
H -5.489372 -1.702344 1.567445
H -3.269951 -2.209444 -3.222376
H -2.231889 -2.481601 -1.814789

H -2.216068 -0.900894 -2.649840
H -5.313350 -1.031202 -3.169392
H -4.424129 0.457001 -2.784775
H -5.842813 -0.023327 -1.814439
H -5.025430 -3.144650 -1.726727
H -5.758409 -2.168717 -0.445780
H -4.216328 -3.020476 -0.152141
H 2.016619 -3.572902 -0.778513
H 6.383989 1.370683 -1.500824
H 6.444184 0.201498 -0.173387
H 5.549279 1.742727 0.018044
H 4.489709 1.562051 -3.006991
H 3.551191 2.211193 -1.640103
H 2.899189 0.872754 -2.616617
H 5.553458 -0.610987 -2.957087
H 4.030486 -1.432640 -2.560927
H 5.489395 -1.702367 -1.567436
H 3.269861 -2.209426 3.222387
H 2.231835 -2.481596 1.814765
H 2.215990 -0.900890 2.649799
H 5.313256 -1.031175 3.169450
H 4.424038 0.457017 2.784783
H 5.842756 -0.023316 1.814499
H 5.025392 -3.144645 1.726807
H 5.758415 -2.168729 0.445885
H 4.216340 -3.020498 0.152194

Total energies (in hartee) and xyz coordinates (in angstrom) of 1^{Se+} (ωB97X-D/def2-TZVP// ωB97X-D/def2-SVP).

E = -7633.15217506

C -1.113716 -1.764755 -0.422507
C -1.109668 -3.157062 -0.463927
C -0.000034 -3.852873 -0.000017
C 1.109614 -3.157084 0.463895
C 1.113688 -1.764777 0.422478
C -0.000006 -1.023696 -0.000010
O 2.259870 -1.144160 0.866534
P 3.416232 -0.352359 0.014078
C 4.535569 0.114320 1.433249
C 4.812787 -1.101190 2.333314

Sn 0.000002 1.137128 0.000006

Se 2.534000 1.344152 -1.020206

O -2.259895 -1.144135 -0.866566

P -3.416252 -0.352352 -0.014087

C -4.535600 0.114354 -1.433243

C -4.812803 -1.101133 -2.333344

Cl -0.541967 2.391091 -1.927056

Cl 0.541979 2.391059 1.927086

Se -2.533994 1.344154 1.020220

C 4.146847 -1.494349 -1.281187

C 5.132557 -0.688627 -2.148280

C 4.862738 -2.685166 -0.630017

C 3.012846 -2.000774 -2.193495

C -4.146816 -1.494382 1.281171

C -5.132432 -0.688667 2.148379

C -4.862779 -2.685149 0.629991

C -3.012771 -2.000880 2.193384

C 3.795720 1.179927 2.256772

C 5.843534 0.701610 0.886107

C -3.795735 1.179976 -2.256735

C -5.843580 0.701613 -0.886106

H 1.992339 -3.678100 0.835933

H -0.000036 -4.943972 -0.000015

H 6.399749 1.142592 1.726561

H 5.667967 1.504858 0.154203

H 6.490143 -0.063315 0.434297

H 5.406792 -0.752966 3.191696

H 5.392468 -1.886459 1.834996

H 3.884361 -1.537216 2.724980

H 4.431554 1.447906 3.113869

H 2.840609 0.807328 2.650242

H 3.598329 2.093149 1.679873

H 3.476549 -2.515359 -3.048665

H 2.401376 -1.178801 -2.591418

H 2.356289 -2.722450 -1.693683

H 5.540461 -1.369328 -2.910925

H 5.981101 -0.286227 -1.582661

H 4.635398 0.137722 -2.675838

H 5.121627 -3.407663 -1.418625

H 4.232592 -3.207685 0.103833

H 5.800523 -2.388850 -0.141240

H -1.992404 -3.678060 -0.835967

H -6.399768 1.142618 -1.726567

H -5.668035 1.504834 -0.154169

H -6.490192 -0.063333 -0.434339

H -5.406799 -0.752888 -3.191725

H -5.392472 -1.886426 -1.835048

H -3.884370 -1.537135 -2.725025

H -4.431548 1.447997 -3.113834

H -2.840618 0.807386 -2.650193

H -3.598339 2.093182 -1.679809

H -3.476435 -2.515508 3.048551

H -2.401266 -1.178942 2.591320

H -2.356255 -2.722538 1.693492

H -5.540293 -1.369388 2.911029

H -5.981019 -0.286234 1.582852

H -4.635204 0.137645 2.675931

H -5.121623 -3.407684 1.418580

H -4.232700 -3.207640 -0.103935

H -5.800595 -2.388788 0.141302

Total energies (in hartee) and xyz coordinates (in angstrom) of 2^{O} (ωB97X-D/def2-TZVP// ωB97X-D/def2-SVP).

E = -3212.28063655

C -1.126569 -2.280293 -0.351903

C -0.000107 -1.519002 -0.000072

C 1.126302 -2.280386 0.351726

C 1.137994 -3.674545 0.372767

C -0.000194 -4.375740 -0.000202

C -1.138346 -3.674445 -0.373093

Sn -0.000046 0.702866 -0.000100

Cl -0.130363 0.506813 -2.516698

O 2.337274 -1.714369 0.720813

P 3.131880 -0.493838 0.010584

C 3.790934 -1.085763 -1.618422

C 5.006180 -2.006302 -1.454133

O -2.337556 -1.714200 -0.720813

P -3.131974 -0.493566 -0.010566

C -3.790698 -1.085282 1.618688

C -2.673157 -1.856366 2.345586

C 0.000311 2.852677 -0.000098

C 0.207037 3.559844 -1.188829

C 0.209509 4.955404 -1.186858

C 0.000830 5.656403 -0.000143

C -0.208109 4.955507 1.186587

C -0.206153 3.559944 1.188588

O -2.205171 0.691895 0.228368

Cl 0.129840 0.506858 2.516570

O 2.205117 0.691588 -0.228766

C -4.382924 -0.084747 -1.303538

C -5.411972 0.894758 -0.717910

C -5.067234 -1.337670 -1.868369

C -3.605220 0.619172 -2.429812

C 4.382583 -0.084946 1.303792

C 5.412108 0.894106 0.718274

C 3.604694 0.619466 2.429621

C 5.066398 -1.337908 1.869152

C 4.140983 0.159635 -2.452532

C 2.673239 -1.856188 -2.345792

C -4.139745 0.160279 2.452968

C -5.006492 -2.005182 1.454840

H 2.056699 -4.184375 0.665622

H -0.000219 -5.467176 -0.000244

H -2.057091 -4.184185 -0.665977

H 3.019283 -2.062827 -3.370945

H 1.743687 -1.273197 -2.423308

H 2.454182 -2.820323 -1.867817

H 5.285565 -2.397547 -2.444823

H 4.781107 -2.869713 -0.809914

H 5.884637 -1.484395 -1.049301

H 4.519588 -0.172172 -3.432049

H 4.917724 0.782807 -1.988429

H 3.247019 0.776675 -2.616931

H -0.356103 3.017379 2.124095

H -0.372103 5.496939 2.121698

H 0.001030 6.749249 -0.000146

H 0.373683 5.496757 -2.121985

H 0.356806 3.017194 -2.124316

H 5.730033 -1.030116 2.692344

H 5.683162 -1.862600 1.128403

H 4.328996 -2.043492 2.275533

H 6.053946 1.258552 1.535162

H 4.922870 1.769003 0.264481

H 6.067137 0.426292 -0.030803

H 4.314631 0.879567 3.230330

H 2.817349 -0.019897 2.850202

H 3.120868 1.539933 2.076693

H -5.285769 -2.396195 2.445655

H -4.782070 -2.868779 0.810645

H -5.884809 -1.482868 1.050242

H -3.018986 -2.062957 3.370824

H -1.743304 -1.273835 2.422920

H -2.454732 -2.820539 1.867402

H -4.518411 -0.171356 3.432523

H -4.916127 0.784028 1.989029

H -3.245312 0.776665 2.617275

H -4.315388 0.879352 -3.230289

H -2.818252 -0.020557 -2.850543

H -3.120939 1.539539 -2.077255

H -6.054093 1.259052 -1.534641

H -4.922300 1.769692 -0.264663

H -6.066759 0.427368 0.031643

H -5.730907 -1.029955 -2.691559

H -5.684036 -1.861937 -1.127359

H -4.330109 -2.043611 -2.274648

Total energies (in hartee) and xyz coordinates (in angstrom) of 2^{S} (ωB97X-D/def2-TZVP// ωB97X-D/def2-SVP).

E = -3858.17214460

C 0.899145 -1.546551 -0.954210

C -0.319254 -0.842561 -0.911422

C -1.482647 -1.571690 -1.193150

C -1.434846 -2.879179 -1.672593

C -0.205590 -3.511885 -1.796616

C 0.961769 -2.865537 -1.408358

Sn -0.247170 1.307316 -0.909505

C 0.483456 2.787685 0.456970

C 1.276820 3.801680 -0.093899

C 1.818358 4.793754 0.722629

C 1.578240 4.780916 2.095436

C 0.786605 3.775864 2.648378

C 0.238095 2.786028 1.833368

O -2.743374 -1.044395 -1.007250

P -3.355087 -0.556902 0.442706

C -5.110379 -0.201086 -0.096925

C -5.866960 -1.480872 -0.472956

O 2.022737 -0.878497 -0.566068

P 3.270162 -1.420241 0.413297

C 4.710167 -1.241576 -0.774458

C 4.737765 0.115837 -1.490967

Cl 1.450720 1.535973 -2.620875

Cl -1.970222 2.177019 -2.282593

S -2.476060 1.065440 1.156861

C -3.164092 -1.994036 1.648456
C -4.075508 -1.710082 2.854289
C -1.702619 -2.038596 2.134120
C -3.519229 -3.356516 1.037413
C 3.188306 -0.135075 1.772011
C 3.446190 1.281342 1.244974
S 3.068025 -3.225108 1.129778
C 1.768563 -0.246405 2.355807
C 4.195611 -0.479209 2.878495
C -5.854747 0.559408 1.010951
C -5.008877 0.712309 -1.331263
C 4.530111 -2.342642 -1.834127
C 6.017637 -1.477235 -0.006532
H -2.367234 -3.378877 -1.936716
H -0.154149 -4.533767 -2.176531
H 1.917297 -3.381820 -1.452184
H -6.029142 1.005720 -1.623966
H -4.533384 0.206297 -2.181125
H -4.433345 1.622641 -1.116932
H -6.818573 -1.196248 -0.948192
H -6.113794 -2.094327 0.405035
H -5.305841 -2.092800 -1.193994
H -6.846569 0.843686 0.626339
H -5.321128 1.478229 1.291034
H -6.011471 -0.042127 1.915551
H -3.417849 -4.119582 1.825301
H -2.832266 -3.632396 0.229353
H -4.546596 -3.409729 0.660762
H -3.836008 -2.431418 3.650942
H -5.139406 -1.835847 2.608015
H -3.919180 -0.698665 3.257939
H -1.619041 -2.831632 2.893708
H -1.397940 -1.087657 2.590654
H -0.994407 -2.278458 1.329456
H 6.849361 -1.535682 -0.726199
H 5.990162 -2.416894 0.564753
H 6.241406 -0.651001 0.683929
H 5.466946 0.055857 -2.314610
H 5.060281 0.931916 -0.834154
H 3.761698 0.379903 -1.921198
H 5.398389 -2.323541 -2.511017
H 3.631986 -2.164980 -2.444267
H 4.466697 -3.341627 -1.380083
H 4.070680 0.242222 3.701708
H 5.236447 -0.409274 2.533739
H 4.029282 -1.491362 3.272432

H 3.099878 2.016595 1.988013

H 2.913539 1.488217 0.308156

H 4.518205 1.457196 1.080561

H 1.671256 0.464860 3.190300

H 1.572923 -1.260829 2.731186

H 0.992543 0.001185 1.619583

H -0.375995 2.007753 2.288291

H 0.590920 3.758862 3.722962

H 2.006512 5.556178 2.734821

H 2.434787 5.579197 0.279857

H 1.497150 3.807803 -1.163637

Total energies (in hartee) and xyz coordinates (in angstrom) of 2^{Se} (ωB97X-D/def2-TZVP// ωB97X-D/def2-SVP).

E = -7864.99670278

C 1.018099 -1.273788 -1.127765

C -0.267403 -0.707751 -1.032584

C -1.348657 -1.534373 -1.365715

C -1.166716 -2.791001 -1.940052

C 0.120984 -3.279559 -2.110413

C 1.215985 -2.542130 -1.676250

Sn -0.427486 1.435368 -0.917397

Cl 1.205778 1.894909 -2.650779

O -2.657473 -1.155268 -1.150253

P -3.344091 -0.880634 0.322067

C -5.124116 -0.699327 -0.237920

C -5.988172 -0.151827 0.907482

O 2.069554 -0.517806 -0.699262

P 3.379672 -0.973684 0.240816

C 3.192557 0.255393 1.646617

C 3.298337 1.704760 1.156358

C 0.191710 2.949019 0.472010

C 0.010831 2.882391 1.856620

C 0.471843 3.909165 2.679963

C 1.112607 5.016478 2.127129

C 1.289786 5.092976 0.746907

C 0.834926 4.064704 -0.077856

Cl -2.253441 2.175290 -2.233998

Se -2.640694 0.896476 1.260456

C -2.995358 -2.390601 1.403537

C -3.188336 -3.725601 0.670538

C -1.540599 -2.316123 1.905456
C -3.942499 -2.322765 2.613062
C 4.779521 -0.602454 -0.958974
C 6.117432 -0.716175 -0.216875
Se 3.370908 -2.953267 0.949674
C 4.644832 0.774821 -1.625132
C 4.708125 -1.673323 -2.061042
C -5.697034 -2.025516 -0.753025
C -5.117633 0.329467 -1.382330
C 1.801183 -0.010911 2.247539
C 4.247791 -0.009605 2.729602
H -2.042765 -3.364259 -2.244232
H 0.277523 -4.259112 -2.565405
H 2.220390 -2.948305 -1.763304
H -6.161429 0.511127 -1.682681
H -4.559421 -0.029708 -2.256166
H -4.675273 1.285201 -1.070554
H -7.003911 0.019427 0.518311
H -5.600680 0.807112 1.278934
H -6.071641 -0.845461 1.754058
H -6.663255 -1.820809 -1.239690
H -5.888254 -2.741177 0.058838
H -5.041553 -2.494094 -1.501200
H -3.626811 -3.082487 3.344822
H -4.982645 -2.546571 2.336676
H -3.907681 -1.342458 3.111325
H -3.004320 -4.536818 1.392519
H -2.468658 -3.850523 -0.145961
H -4.200171 -3.863746 0.274903
H -1.370290 -3.168380 2.581812
H -1.347824 -1.389265 2.461589
H -0.806033 -2.391187 1.092101
H 4.069286 0.686072 3.565050
H 5.271353 0.162674 2.369763
H 4.184455 -1.037685 3.112083
H 2.897972 2.379224 1.929065
H 2.727633 1.885932 0.236757
H 4.344041 1.990812 0.979304
H 1.647337 0.672789 3.096329
H 1.714325 -1.045275 2.608856
H 0.992217 0.168643 1.527086
H 5.563702 -1.532545 -2.739732
H 3.792247 -1.566326 -2.660900
H 4.756379 -2.692308 -1.651590
H 6.936999 -0.643589 -0.949088
H 6.212487 -1.676596 0.311061

H 6.254497 0.099754 0.507440

H 5.358404 0.821164 -2.463098

H 4.892414 1.597916 -0.945399

H 3.637327 0.946677 -2.028644

H 1.006344 4.125278 -1.154738

H 1.788613 5.957844 0.304082

H 1.472773 5.820929 2.772334

H 0.328161 3.839425 3.760593

H -0.477518 2.021026 2.314897

Total energies (in hartee) and xyz coordinates (in angstrom) of 2^{O+} (ωB97X-D/def2-TZVP// ωB97X-D/def2-SVP).

E = -2751.84889202

C -1.427487 3.298058 0.401974

C -0.275270 2.619072 -0.012592

C 0.685977 3.289764 -0.776839

C 0.479004 4.617653 -1.149295

C -0.675461 5.286321 -0.745722

C -1.623734 4.628836 0.037325

Sn 0.008257 0.606618 0.581128

Cl 0.260365 0.228454 2.890306

C 0.089338 -1.298852 -0.459593

C 1.242353 -2.108015 -0.448552

C 1.288845 -3.362070 -1.053027

C 0.177141 -3.835766 -1.737857

C -0.976757 -3.065588 -1.797837

C -1.014947 -1.833373 -1.148415

O 2.408439 -1.725733 0.181076

P 3.170206 -0.300001 -0.022104

C 4.457384 -0.348960 1.290905

C 5.620920 -1.280517 0.927220

O -2.207750 -1.151598 -1.258545

P -3.080173 -0.532192 -0.025642

O -2.170564 0.363912 0.820783

O 2.165895 0.830787 0.239261

C -4.312664 0.478308 -0.945356

C -5.000631 1.438753 0.040367

C -3.685931 -1.941282 1.003265

C -4.178167 -3.108965 0.136670

C -5.345437 -0.399550 -1.664214

C -3.515839 1.301587 -1.976858

C 3.735721 -0.216036 -1.783444

C 4.746652 0.935638 -1.918724

C -2.500471 -2.403577 1.873502

C -4.811577 -1.429969 1.918417

C 2.506009 0.094274 -2.659632

C 4.357814 -1.543349 -2.241829

C 4.950381 1.090817 1.525045

C 3.767672 -0.857280 2.570712

H -5.730027 -1.197753 1.360887

H -5.059797 -2.221032 2.641872

H -4.505250 -0.542067 2.491288

H -3.372225 -3.525785 -0.482031

H -4.529990 -3.910992 0.803220

H -5.015150 -2.835365 -0.517973

H -1.668946 -2.799861 1.272962

H -2.115117 -1.596448 2.510873

H -2.851622 -3.218750 2.524133

H 3.637379 -2.370820 -2.188633

H 4.663891 -1.439216 -3.293909

H 5.249659 -1.820365 -1.666932

H 4.345283 1.885020 -1.533317

H 5.696292 0.721683 -1.409132

H 4.974230 1.079437 -2.985716

H 1.761911 -0.713913 -2.642735

H 2.012315 1.030789 -2.367725

H 2.846311 0.204624 -3.700384

H -1.862117 -3.403248 -2.337266

H 1.608920 2.784814 -1.064458

H 1.229009 5.134786 -1.751243

H -0.832467 6.327664 -1.034228

H -2.520112 5.155880 0.370742

H -2.171937 2.788282 1.015661

H 0.211090 -4.809757 -2.228641

H 2.207535 -3.945033 -0.981274

H -4.209925 1.997370 -2.471531

H -3.062721 0.664504 -2.747552

H -2.726714 1.903142 -1.503130

H -5.657598 2.109959 -0.532766

H -4.270711 2.059749 0.576734

H -5.624825 0.919563 0.778653

H -5.977229 0.245931 -2.292767

H -6.011187 -0.921308 -0.962162

H -4.869611 -1.140692 -2.322943

H 4.495154 -0.799561 3.394120

H 3.440453 -1.900793 2.473585

H 2.898560 -0.243637 2.843260

H 6.293755 -1.348364 1.795161

H 6.217433 -0.904805 0.084090

H 5.277044 -2.299614 0.696939

H 5.643120 1.081804 2.379716

H 4.118049 1.765142 1.769377

H 5.495158 1.502775 0.665467

Total energies (in hartee) and xyz coordinates (in angstrom) of 2^{S+} (ωB97X-D/def2-TZVP// ωB97X-D/def2-SVP).

E = -3397.72804768

C -0.466020 2.826535 -1.590087

C -0.671062 2.593289 -0.226883

C -1.378348 3.518708 0.546483

C -1.885382 4.672780 -0.048874

C -1.696903 4.897231 -1.412826

C -0.987209 3.976362 -2.182561

Sn 0.028611 0.801066 0.660218

S -2.427398 0.205092 1.649427

P -3.230325 -0.786555 0.100868

C -3.797469 -2.472592 0.688806

C -4.129850 -3.425382 -0.468243

C 0.174657 -1.113702 -0.313555

C -0.911935 -1.662817 -1.015906

C -0.811574 -2.866704 -1.710446

C 0.360423 -3.609634 -1.633886

C 1.442256 -3.124779 -0.910499

C 1.354577 -1.872285 -0.306752

O -2.120418 -1.003575 -1.097358

O 2.490981 -1.425900 0.328702

P 3.488028 -0.196297 -0.101250

C 4.714928 -0.332040 1.294408

C 5.162626 -1.789310 1.492699

Cl 0.595116 0.906593 2.941336

S 2.486592 1.550436 -0.124915

C 4.147179 -0.504752 -1.827252

C 4.974512 -1.795191 -1.886674

C 2.954178 -0.599578 -2.798624

C 5.005719 0.701600 -2.251072

C -4.488110 0.241190 -0.815257

C -5.245070 -0.592394 -1.857639

C -5.465964 0.866588 0.192451
C -3.716607 1.370064 -1.519847
C 3.995125 0.143833 2.566615
C 5.920001 0.576797 1.015164
C -5.039355 -2.268719 1.573603
C -2.667793 -3.088072 1.536927
H 6.525148 0.637168 1.931933
H 5.617525 1.602052 0.753381
H 6.569358 0.178935 0.222967
H 5.827842 -1.820140 2.368807
H 5.727501 -2.187910 0.641872
H 4.310841 -2.451621 1.695701
H 4.696607 0.049260 3.409112
H 3.110757 -0.466703 2.792568
H 3.679427 1.193200 2.498002
H -3.004612 -4.077982 1.880656
H -2.435963 -2.478004 2.419141
H -1.741858 -3.236883 0.963175
H -4.454092 -4.383307 -0.033890
H -3.256265 -3.634593 -1.095431
H -4.943027 -3.066843 -1.107809
H -5.268518 -3.222701 2.072061
H -5.924541 -1.986253 0.986854
H -4.874030 -1.516622 2.359301
H -4.448523 2.019165 -2.023342
H -3.020534 0.988025 -2.277920
H -3.158300 1.990811 -0.806997
H -5.841907 0.092537 -2.478483
H -5.945611 -1.301374 -1.395845
H -4.565880 -1.138845 -2.527924
H -6.151561 1.524172 -0.362959
H -4.941508 1.478749 0.939269
H -6.078593 0.122628 0.717324
H -1.548736 3.344122 1.611881
H -2.431858 5.399349 0.555792
H -2.100683 5.799160 -1.877276
H -0.834666 4.155048 -3.248885
H 0.097283 2.117447 -2.202750
H 2.374663 -3.685009 -0.835550
H 0.432173 -4.569487 -2.147872
H -1.661262 -3.213141 -2.297649
H 5.344192 0.528197 -3.283942
H 5.900565 0.833924 -1.631955
H 4.428204 1.636546 -2.239302
H 5.195372 -2.020890 -2.940888
H 4.439689 -2.659299 -1.467503

H 5.937576 -1.692869 -1.368607

H 3.354549 -0.604862 -3.823724

H 2.283377 0.265808 -2.706044

H 2.370009 -1.518397 -2.669691

Total energies (in hartee) and xyz coordinates (in angstrom) of 2^{Se+} (ωB97X-D/def2-TZVP// ωB97X-D/def2-SVP).

E = -7404.55390785

C -0.443723 2.704735 1.741994

C -0.653824 2.536473 0.369079

C -1.324044 3.519664 -0.363425

C -1.791038 4.664754 0.280211

C -1.598553 4.824238 1.652612

C -0.924677 3.846197 2.383143

Sn 0.022110 0.770427 -0.595394

Cl 0.582333 0.974826 -2.882465

C 0.152567 -1.151803 0.375220

C -0.940447 -1.658995 1.096220

C -0.874501 -2.853754 1.810254

C 0.277249 -3.627566 1.744949

C 1.366409 -3.184640 1.004781

C 1.310056 -1.944987 0.371140

O -2.128475 -0.965548 1.170973

P -3.308459 -0.807036 0.030648

C -4.505790 0.223220 1.033647

C -4.673656 -0.353019 2.449026

O 2.445184 -1.556477 -0.303644

P 3.546543 -0.402780 0.082910

C 4.670639 -0.597009 -1.396906

C 4.998306 -2.078324 -1.646944

Se 2.594044 1.534018 0.218318

Se -2.508851 0.173329 -1.715982

C 4.307994 -0.799937 1.752391

C 5.261197 0.343450 2.146688

C 5.066401 -2.133239 1.716347

C 3.185295 -0.865473 2.805813

C -3.928930 -2.505261 -0.478855

C -4.948609 -2.333608 -1.620878

C -4.574560 -3.244232 0.700331

C -2.744203 -3.324449 -1.027234

C 5.953347 0.217966 -1.184471
C 3.908393 -0.049472 -2.613712
C -3.912549 1.636083 1.143105
C -5.857540 0.296460 0.310701
H 3.658292 -0.922274 3.797972
H 2.548032 -1.750329 2.694351
H 2.552220 0.032721 2.788119
H 5.679572 0.105788 3.136666
H 4.734483 1.304596 2.229766
H 6.103898 0.463408 1.455979
H 5.342461 -2.405172 2.746382
H 5.997965 -2.064065 1.138781
H 4.460025 -2.954097 1.307894
H -6.476726 1.053255 0.815049
H -6.408245 -0.653356 0.351717
H -5.751934 0.606894 -0.739994
H -5.345723 0.316163 3.007445
H -3.717209 -0.393310 2.986228
H -5.126756 -1.351042 2.459440
H -4.592837 2.246079 1.756221
H -3.808895 2.120277 0.163151
H -2.930619 1.635194 1.634553
H -3.153019 -4.234199 -1.492532
H -2.046986 -3.641722 -0.243635
H -2.185530 -2.776432 -1.798821
H -5.281844 -3.337441 -1.925517
H -4.499048 -1.851692 -2.500426
H -5.840707 -1.768542 -1.327439
H -4.774039 -4.282157 0.393657
H -5.536896 -2.801663 0.991025
H -3.923100 -3.280225 1.584890
H -1.496772 3.397560 -1.435997
H 2.280885 -3.774123 0.933212
H -2.309299 5.435838 -0.293473
H -1.970157 5.719966 2.154453
H -0.768052 3.973595 3.456258
H 0.093210 1.950722 2.324566
H 0.326391 -4.581131 2.273027
H -1.742889 -3.175940 2.385454
H 4.543161 -0.187203 -3.501988
H 3.681198 1.020562 -2.517878
H 2.965853 -0.585882 -2.786003
H 5.598743 -2.137587 -2.567299
H 4.089876 -2.675007 -1.801668
H 5.589674 -2.533023 -0.844049
H 6.502491 0.249079 -2.137280

H 6.620939 -0.237345 -0.439853

H 5.743998 1.258701 -0.893623

Total energies (in hartee) and xyz coordinates (in angstrom) of 3° (ωB97X-D/def2-TZVP// ωB97X-D/def2-SVP).

E = -2983.66891460

C 0.258201 -0.845449 2.754633

C 0.522781 -1.584495 1.594455

C 1.377733 -2.691131 1.677779

C 1.969873 -3.040241 2.891759

C 1.714995 -2.286587 4.036839

C 0.855670 -1.190734 3.967123

Sn -0.387583 -1.108398 -0.277065

O -2.051960 0.243511 1.014037

P -2.806812 1.356095 0.314598

C -2.402645 2.974689 1.145050

C -2.770172 4.203375 0.303301

C -0.023418 0.817409 -1.175076

C 1.299154 1.206163 -1.457704

C 1.579000 2.321111 -2.256725

C 0.527891 3.084140 -2.752498

C -0.788582 2.781048 -2.426472

C -1.037829 1.672587 -1.622220

O 2.294838 0.440502 -0.916672

P 3.858681 0.895415 -0.566293

C 3.794842 1.334515 1.242770

C 2.497048 2.132833 1.445273

O -2.361103 1.473259 -1.256006

C -2.135640 -2.282854 -0.641922

C -2.500711 -2.693407 -1.928056

C -3.642661 -3.471111 -2.124749

C -4.416447 -3.866883 -1.034195

C -4.041760 -3.489327 0.255463

C -2.909112 -2.698775 0.448528

Cl 0.970463 -2.406738 -1.850046

C -4.617903 0.995197 0.130236

C -5.084210 0.298957 1.419760

C -4.793394 0.029141 -1.054607

C -5.456271 2.250547 -0.144830

C 4.772169 -0.649314 -1.008241

C 6.247967 -0.410591 -0.651591
O 4.350410 2.066191 -1.358961
C 4.612317 -0.764356 -2.536535
C 4.250887 -1.931631 -0.354121
C -3.126722 3.002990 2.502640
C -0.885719 2.990617 1.410927
C 3.798648 0.131177 2.187152
C 5.003575 2.240528 1.526859
H -0.645599 3.885020 2.007332
H -0.297320 3.039519 0.485357
H -0.563576 2.106145 1.974855
H -2.446238 5.107233 0.843325
H -3.847523 4.297202 0.123719
H -2.253525 4.196758 -0.665504
H -2.779006 3.879114 3.072027
H -2.901097 2.105222 3.097611
H -4.217432 3.091472 2.401221
H 4.958932 2.580985 2.574041
H 5.006489 3.116217 0.864501
H 5.957357 1.710267 1.388501
H 2.494560 2.571132 2.456104
H 1.615265 1.483672 1.356919
H 2.402768 2.954318 0.718242
H 3.631533 0.483068 3.217796
H 4.761714 -0.399330 2.173808
H 2.999598 -0.583061 1.951140
H 4.785288 -2.788769 -0.795337
H 3.181947 -2.073931 -0.556201
H 4.420186 -1.960675 0.729033
H 6.854590 -1.238086 -1.052303
H 6.408510 -0.383815 0.437452
H 6.619385 0.530242 -1.083910
H 5.243472 -1.590860 -2.899560
H 4.921121 0.160978 -3.042739
H 3.569662 -0.991436 -2.803504
H 1.590785 -3.282965 0.783797
H 2.636296 -3.904523 2.940984
H 2.183161 -2.556758 4.986244
H 0.645300 -0.602406 4.863572
H -0.437416 -0.005244 2.710887
H -1.885842 -2.414183 -2.787229
H -3.925559 -3.774842 -3.135359
H -5.307945 -4.479244 -1.188640
H -4.635876 -3.809783 1.114787
H -2.634171 -2.390872 1.461053
H -1.625437 3.386525 -2.776659

H 0.746261 3.944682 -3.388072

H 2.615882 2.580124 -2.463831

H -6.496309 1.938584 -0.327882

H -5.110162 2.783650 -1.042425

H -5.472204 2.951590 0.700857

H -6.141946 0.015342 1.304816

H -5.006481 0.946149 2.304628

H -4.501658 -0.613118 1.605197

H -5.851806 -0.271003 -1.100075

H -4.193478 -0.879619 -0.938706

H -4.524045 0.502584 -2.007612

Total energies (in hartee) and xyz coordinates (in angstrom) of 3^S (ωB97X-D/def2-TZVP// ωB97X-D/def2-SVP).

E = -3629.56502745

C -0.348426 1.994998 2.612035

C -0.438818 2.391032 1.272860

C -1.044470 3.619764 0.974485

C -1.552136 4.427840 1.991443

C -1.464837 4.018950 3.321029

C -0.861082 2.800876 3.628525

Sn 0.312926 1.269597 -0.397293

Cl -1.150274 2.160544 -2.132308

C 0.023721 -0.811461 -0.896200

C -1.261202 -1.281116 -1.230381

C -1.442915 -2.419081 -2.020030

C -0.334713 -3.132251 -2.461108

C 0.942898 -2.754796 -2.071368

C 1.101497 -1.628613 -1.265303

O -2.330934 -0.559161 -0.789550

P -3.751205 -1.113562 -0.101756

C -3.658994 -0.267460 1.564668

C -2.345017 -0.757903 2.198420

O 2.395237 -1.360345 -0.860315

P 2.937422 -1.361931 0.697085

C 2.381811 -2.978712 1.494538

C 2.546088 -4.206422 0.588200

C 2.112644 2.058115 -1.226429

C 2.511416 1.695093 -2.516785

C 3.664380 2.240978 -3.080320

C 4.422019 3.165983 -2.362425

C 4.022072 3.546236 -1.081504

C 2.873252 2.992706 -0.516000

C 4.772101 -1.244954 0.342617

C 5.534186 -0.907833 1.632668

S 2.306006 0.179150 1.756645

C 4.949213 -0.081858 -0.648844

C 5.323097 -2.530335 -0.286123

C -4.987850 -0.382326 -1.306517

C -6.401824 -0.571023 -0.741536

S -3.910843 -3.050495 0.097403

C -4.840318 -1.188301 -2.608873

C -4.721550 1.096242 -1.622958

C 3.209782 -3.164794 2.777359

C 0.895813 -2.849568 1.880154

C -3.657204 1.259724 1.435411

C -4.827809 -0.722425 2.449859

H 0.601143 -3.764397 2.417973

H 0.238363 -2.755079 1.005323

H 0.721031 -1.988306 2.538466

H 2.242053 -5.095802 1.162652

H 3.577627 -4.368664 0.256796

H 1.896159 -4.152585 -0.292488

H 2.775102 -3.994040 3.356871

H 3.192040 -2.265217 3.410255

H 4.254847 -3.429086 2.561394

H -4.706913 -0.270679 3.447464

H -4.846197 -1.815412 2.560401

H -5.801596 -0.398936 2.056712

H -2.251372 -0.320351 3.204274

H -1.464805 -0.448556 1.619964

H -2.333116 -1.853621 2.287043

H -3.308662 1.705964 2.379860

H -4.667438 1.645746 1.240322

H -2.988428 1.614171 0.641200

H -5.344594 1.381482 -2.485731

H -3.671144 1.283563 -1.886417

H -4.995185 1.759400 -0.794240

H -7.136324 -0.292185 -1.513559

H -6.583304 0.074930 0.130047

H -6.587402 -1.615702 -0.450990

H -5.598584 -0.838396 -3.326665

H -4.989146 -2.264599 -2.443112

H -3.852603 -1.026862 -3.066002

H -1.149237 3.940423 -0.064974

H -2.022668 5.381051 1.739790

H -1.866152 4.649913 4.117464

H -0.786485 2.471809 4.667671

H 0.118630 1.045191 2.876890

H 1.920580 0.980147 -3.096523

H 3.970656 1.945790 -4.086745

H 5.323645 3.596153 -2.804675

H 4.608885 4.275548 -0.518095

H 2.579267 3.286568 0.495631

H 1.826327 -3.316545 -2.376399

H -0.475430 -4.007124 -3.098427

H -2.446601 -2.744349 -2.282313

H 6.350182 -2.336859 -0.633007

H 4.732325 -2.846233 -1.158217

H 5.374539 -3.360033 0.432990

H 6.594327 -0.753891 1.377492

H 5.487108 -1.709221 2.381476

H 5.153455 0.016306 2.089045

H 6.027377 0.077250 -0.806245

H 4.520497 0.852855 -0.262590

H 4.484338 -0.294541 -1.619345

Total energies (in hartee) and xyz coordinates (in angstrom) of 3^{Se} (ωB97X-D/def2-TZVP// ωB97X-D/def2-SVP).

E = -7636.39068323

C -1.007843 -1.577475 -1.308689

C 0.001115 -0.682972 -0.930856

C 1.322036 -1.066592 -1.230680

C 1.597099 -2.211719 -1.982510

C 0.551478 -3.014590 -2.423937

C -0.757931 -2.713485 -2.076723

Sn -0.480164 1.370134 -0.473321

Se 4.148724 -2.690631 0.252478

P 3.794969 -0.647396 -0.104040

C 4.960028 0.107001 -1.372637

C 4.572876 1.541998 -1.758903

O 2.327448 -0.252323 -0.801245

O -2.321112 -1.376021 -0.940919

P -2.954128 -1.524820 0.575701

C -2.308963 -3.128403 1.341941

C -2.720846 -4.365739 0.535433

C 0.143022 2.606363 1.169649

C 0.121866 2.232775 2.517874

C 0.544111 3.118116 3.509456

C 0.987015 4.394623 3.167227

C 1.004752 4.780949 1.828220

C 0.587842 3.893459 0.836451

C -2.330964 1.958846 -1.356665

C -2.649689 1.532015 -2.650851

C -3.832076 1.948742 -3.261874

C -4.700800 2.807728 -2.588295

C -4.383952 3.247932 -1.303775

C -3.205085 2.822904 -0.690587

Cl 0.929849 2.349094 -2.205033

Se -2.475473 0.112135 1.836756

C -4.758508 -1.575359 0.055435

C -5.638064 -2.030966 1.226066

C -4.965457 -2.486587 -1.164419

C -5.145007 -0.141357 -0.341861

C 3.636282 0.307528 1.502747

C 3.462441 1.811813 1.258910

C 4.858302 0.057615 2.396829

C 2.391248 -0.269021 2.199650

C 6.393437 0.062182 -0.828255

C 4.858234 -0.770665 -2.632024

C -2.860691 -3.237615 2.775377

C -0.770671 -3.062048 1.442945

H 2.625481 -2.472443 -2.219593

H 0.768747 -3.896251 -3.029559

H -1.594054 -3.342853 -2.383562

H 5.580438 -0.399256 -3.375726

H 3.857075 -0.704411 -3.083206

H 5.091077 -1.823989 -2.420598

H 5.152496 1.824652 -2.652156

H 4.815392 2.268071 -0.974672

H 3.505726 1.636418 -2.003721

H 7.086761 0.369621 -1.627055

H 6.674404 -0.949063 -0.498930

H 6.534146 0.758211 0.011380

H 2.257036 0.246485 3.162822

H 2.501898 -1.346197 2.387995

H 1.478496 -0.112160 1.609634

H 3.072367 2.284024 2.174039

H 2.754357 2.031488 0.449904

H 4.422034 2.292087 1.023798

H 4.698703 0.576800 3.355392

H 5.785747 0.451678 1.958839

H 4.999059 -1.013128 2.599588

H -0.440517 -3.877457 2.105298

H -0.430648 -2.113633 1.882006

H -0.268755 -3.200643 0.478321

H -2.201206 -5.242963 0.951667

H -2.439321 -4.289413 -0.523015

H -3.799819 -4.563619 0.603015

H -2.463698 -4.162989 3.221614

H -3.955164 -3.295653 2.814806

H -2.535048 -2.390801 3.394612

H -1.971536 0.870153 -3.196955

H -4.073750 1.605476 -4.270552

H -5.625597 3.137242 -3.067537

H -5.059509 3.923590 -0.773951

H -2.976459 3.162338 0.323613

H -6.186573 -0.150085 -0.699715

H -4.510990 0.239987 -1.153103

H -5.072729 0.553800 0.504856

H -6.692251 -1.858019 0.959491

H -5.428440 -1.458846 2.142571

H -5.526738 -3.103557 1.440730

H -6.029567 -2.446180 -1.445685

H -4.718643 -3.536759 -0.967198

H -4.374457 -2.141971 -2.022733

H 0.639717 4.201544 -0.210734

H 1.349927 5.779072 1.549123

H 1.317734 5.088117 3.943824

H 0.526591 2.804729 4.555943

H -0.212834 1.236322 2.811077

Total energies (in hartee) and xyz coordinates (in angstrom) of 3^{O+} (ωB97X-D/def2-TZVP// ωB97X-D/def2-SVP).

E = -2523.24468030

C -0.568513 -1.065192 2.890704

C -0.483069 0.242846 2.399132

C -0.782524 1.315202 3.248071

C -1.167895 1.080814 4.568339

C -1.254549 -0.225695 5.048424

C -0.951334 -1.299070 4.210687

Sn 0.010656 0.589913 0.373368

O 2.194982 0.235702 0.727279

P 3.132439 -0.565825 -0.175052

C 3.796351 -2.004042 0.778300

C 4.331249 -3.109489 -0.142271
C -0.033150 -1.277953 -0.744350
C 1.090036 -1.836367 -1.377554
C 1.060020 -3.073297 -2.019796
C -0.109292 -3.823365 -2.005121
C -1.245171 -3.322778 -1.380472
C -1.201093 -2.061516 -0.790492
O 2.295016 -1.167909 -1.440461
O -2.390916 -1.628765 -0.233762
P -3.100140 -0.195601 -0.573291
C -3.542456 -0.205338 -2.373671
C -4.165103 -1.539638 -2.808944
C 0.485399 2.552660 -0.271369
C -0.180335 3.182432 -1.327885
C 0.182526 4.470712 -1.721052
C 1.197379 5.148276 -1.046895
C 1.846974 4.538836 0.026285
C 1.495896 3.245409 0.409786
O -2.099417 0.929517 -0.300414
C -4.490607 -0.153479 0.636272
C -4.987998 1.299781 0.739132
C -5.630338 -1.101582 0.241660
C -3.918633 -0.576728 2.001549
C 4.335429 0.527209 -1.042296
C 5.427643 -0.278635 -1.757928
C 4.948496 1.489953 -0.010951
C 3.532645 1.341732 -2.075650
C -2.247799 0.031710 -3.175795
C -4.510665 0.959719 -2.642500
C 4.897962 -1.504149 1.727732
C 2.623420 -2.551209 1.614832
H -4.651708 1.055317 -3.729740
H -4.114872 1.917272 -2.272063
H -5.502091 0.791331 -2.199669
H -4.394073 -1.481805 -3.884076
H -5.101622 -1.768158 -2.286490
H -3.471818 -2.378925 -2.665414
H -2.512696 0.099886 -4.241850
H -1.527567 -0.790482 -3.067250
H -1.751492 0.968168 -2.890429
H 2.989713 -3.401012 2.210721
H 1.799841 -2.921033 0.985691
H 2.225832 -1.792426 2.302134
H 4.701861 -3.935636 0.483619
H 5.164540 -2.776585 -0.773374
H 3.543580 -3.514858 -0.791144

H 5.175517 -2.324176 2.407223
H 4.554941 -0.661723 2.346675
H 5.808368 -1.203830 1.189907
H 4.210777 2.077261 -2.533874
H 2.706828 1.899180 -1.611921
H 3.127987 0.704432 -2.872724
H -2.177154 -3.888104 -1.350770
H 6.044766 0.415726 -2.347910
H 5.004588 -1.018151 -2.453539
H 6.098918 -0.792771 -1.055588
H 5.605455 2.194962 -0.542193
H 5.558010 0.976838 0.744320
H 4.171992 2.072641 0.502115
H -0.725270 2.345064 2.883530
H -1.402161 1.921570 5.224714
H -1.558266 -0.408359 6.081335
H -1.019341 -2.322321 4.586408
H -0.351935 -1.917474 2.240038
H -0.997347 2.673480 -1.841761
H -0.334919 4.950365 -2.554564
H 1.477383 6.158273 -1.353408
H 2.631537 5.072653 0.566948
H 2.023130 2.771130 1.241408
H -0.137186 -4.801552 -2.488001
H 1.960518 -3.431422 -2.519595
H -4.725523 -0.505157 2.746186
H -3.103358 0.082452 2.327546
H -3.548803 -1.610308 1.993630
H -5.736709 1.352051 1.543797
H -5.469408 1.654750 -0.181205
H -4.167717 1.985968 0.991720
H -6.372291 -1.111076 1.054224
H -5.277854 -2.134402 0.104122
H -6.152903 -0.778266 -0.669349

Total energies (in hartee) and xyz coordinates (in angstrom) of 3^{S+} (ωB97X-D/def2-TZVP// ωB97X-D/def2-SVP).

E = -3169.12711773

C 0.645205 -1.301005 2.976853
C 0.735724 -1.886358 1.709681
C 1.349897 -3.135875 1.566983
C 1.872809 -3.789907 2.682207

C 1.794462 -3.194364 3.940945
C 1.180301 -1.950866 4.088209
Sn 0.000515 -0.870612 -0.000142
S -2.447419 -0.971492 1.234709
P -3.352845 0.611893 0.406451
C -3.872236 1.792980 1.767891
C -4.281855 3.175726 1.241654
C 0.000035 1.287485 -0.000073
C -1.149631 2.032445 -0.311019
C -1.141630 3.424799 -0.376086
C 0.000064 4.120689 0.000229
C 1.141756 3.424714 0.376418
C 1.149626 2.032399 0.311058
O -2.346113 1.415706 -0.617397
O 2.346101 1.415430 0.617368
P 3.352892 0.611942 -0.406511
C 3.871774 1.792814 -1.768331
C 4.282004 3.175472 -1.242350
C -0.735660 -1.886777 -1.709308
C -0.645332 -1.301844 -2.976695
C -1.180953 -1.951929 -4.087687
C -1.795375 -3.195235 -3.939843
C -1.873462 -3.790371 -2.680899
C -1.350047 -3.136115 -1.566041
S 2.447481 -0.971818 -1.234543
C 4.684854 0.103554 0.796540
C 5.573029 -0.972017 0.151278
C 5.529432 1.303728 1.244355
C 3.975453 -0.507998 2.016865
C -4.684617 0.103485 -0.796824
C -5.529068 1.303694 -1.244797
C -5.572949 -0.972044 -0.151729
C -3.975062 -0.508107 -2.017038
C 2.684125 1.959587 -2.735714
C 5.048407 1.149897 -2.522432
C -5.049477 1.150293 2.521261
C -2.685129 1.959606 2.735948
H 5.239520 1.738200 -3.432721
H 4.829881 0.117705 -2.833983
H 5.973490 1.156872 -1.929203
H 4.597654 3.784600 -2.103165
H 5.122450 3.143684 -0.541276
H 3.446054 3.698925 -0.765854
H 2.989785 2.655896 -3.531588
H 1.797658 2.387642 -2.245477
H 2.401268 1.006660 -3.201218

H -2.990895 2.656477 3.531292

H -1.798148 2.386949 2.245995

H -2.403023 1.006748 3.202049

H -4.599432 3.784417 2.102075

H -5.120826 3.144081 0.538798

H -3.445002 3.699567 0.767174

H -5.240972 1.738520 3.431517

H -4.831463 0.117986 2.832786

H -5.974186 1.157607 1.927445

H -4.746838 -0.877800 -2.708709

H -3.341239 -1.360204 -1.738476

H -3.359706 0.227051 -2.551221

H 2.040181 3.947066 0.703321

H -6.178059 0.978177 -2.071680

H -4.909774 2.132119 -1.618163

H -6.186390 1.674157 -0.446199

H -6.307165 -1.301695 -0.902256

H -6.134060 -0.604095 0.716710

H -4.986739 -1.848901 0.156135

H 1.443205 -3.603146 0.582572

H 2.347368 -4.766587 2.566636

H 2.210187 -3.704267 4.812494

H 1.115123 -1.486132 5.074313

H 0.158061 -0.330635 3.107342

H -0.157969 -0.331641 -3.107643

H -1.115958 -1.487551 -5.073970

H -2.211520 -3.705290 -4.811102

H -2.348225 -4.766896 -2.564863

H -1.443129 -3.603060 -0.581454

H 0.000071 5.211917 0.000328

H -2.040087 3.947227 -0.702785

H 4.747311 -0.878020 2.708270

H 3.341316 -1.359892 1.738400

H 3.360482 0.227269 2.551329

H 6.307712 -1.301293 0.901513

H 6.133618 -0.604283 -0.717591

H 4.986810 -1.849071 -0.155992

H 6.178114 0.978326 2.071528

H 4.910240 2.132435 1.617264

H 6.187061 1.673761 0.445817

Total energies (in hartee) and xyz coordinates (in angstrom) of 3^{Se+} (ωB97X-D/def2-TZVP// ωB97X-D/def2-SVP).

E = -7175.95170076

C -0.603773 -1.289959 2.988900

C -0.712109 -1.852612 1.712636

C -1.313999 -3.106983 1.560323

C -1.807222 -3.787639 2.673130

C -1.711908 -3.214011 3.940848

C -1.109865 -1.965962 4.098405

Sn 0.000044 -0.813583 0.001092

Se -2.547150 -0.935463 -1.294291

P -3.418860 0.743861 -0.266882

C -4.675788 0.217322 1.014544

C -5.655961 -0.790244 0.394432

C -0.000020 1.346379 -0.001377

C 1.134916 2.091645 -0.364087

C 1.119816 3.483631 -0.436726

C -0.000374 4.180427 -0.003319

C -1.120416 3.483947 0.431007

C -1.135166 2.091879 0.360229

O 2.319282 1.476091 -0.720999

P 3.419101 0.744827 0.264065

C 4.019820 2.002755 1.527833

C 4.399263 3.353633 0.904510

O -2.319374 1.476402 0.717668

C 0.711493 -1.859173 -1.706770

C 0.602223 -1.302086 -2.985386

C 1.107959 -1.982707 -4.092253

C 1.710522 -3.229852 -3.929623

C 1.806790 -3.797921 -2.659474

C 1.313983 -3.112603 -1.549353

Se 2.547054 -0.931193 1.296652

C -4.017234 1.999357 -1.534269

C -4.398186 3.351324 -0.914285

C -2.891419 2.223855 -2.562108

C -5.239176 1.397666 -2.249391

C 4.674452 0.214225 -1.017228

C 5.653985 -0.793143 -0.395743

C 3.895575 -0.484219 -2.143926

C 5.433473 1.417039 -1.593289

C -5.433840 1.422183 1.587591

C -3.898468 -0.479652 2.143280

C 5.243224 1.402559 2.241681

C 2.895977 2.229224 2.557384
H -5.479594 2.033477 -3.114995
H -6.127757 1.377029 -1.603392
H -5.047582 0.382680 -2.628269
H -4.736500 4.010989 -1.727940
H -3.545460 3.842829 -0.434742
H -5.216504 3.283064 -0.190674
H -3.244702 2.967074 -3.293272
H -2.640639 1.302780 -3.103687
H -1.974191 2.621346 -2.104668
H 3.250169 2.974727 3.285775
H 2.646916 1.309419 3.101897
H 1.977496 2.624755 2.100695
H 4.741719 4.013940 1.715915
H 3.544709 3.845259 0.428358
H 5.214237 3.284013 0.177275
H 5.485241 2.039982 3.105653
H 6.130539 1.380824 1.593961
H 5.052503 0.388250 2.622825
H -1.998485 4.007290 0.806753
H -1.420807 -3.558214 0.569697
H -2.271923 -4.767958 2.548639
H -2.104295 -3.744646 4.810835
H -1.031048 -1.517746 5.091152
H -0.125147 -0.316496 3.128489
H 0.123175 -0.329409 -3.128906
H 1.028442 -1.538809 -5.086880
H 2.102584 -3.764126 -4.797526
H 2.271919 -4.777522 -2.530999
H 1.421625 -3.559460 -0.556836
H -0.000517 5.271604 -0.004064
H 1.997743 4.006634 -0.813261
H -4.627653 -0.849368 2.879730
H -3.210114 0.204138 2.655860
H -3.327271 -1.343124 1.777560
H -6.344258 -1.124142 1.185581
H -5.135332 -1.676434 0.005409
H -6.265744 -0.360909 -0.410591
H -6.011595 1.080831 2.459730
H -6.152014 1.842775 0.870855
H -4.755911 2.216646 1.931494
H 6.011034 1.073000 -2.464499
H 6.151926 1.838984 -0.877605
H 4.756115 2.211070 -1.939326
H 6.341210 -1.129536 -1.186766
H 5.132604 -1.677844 -0.004364

H 6.264924 -0.362818 0.407875

H 4.623726 -0.855668 -2.880535

H 3.207010 0.199074 -2.656913

H 3.324364 -1.346738 -1.776041

Total energies (in hartee) and xyz coordinates (in angstrom) of 3^{PO} (ωB97X-D/def2-TZVP// ωB97X-D/def2-SVP).

E = -2908.37130949

C -1.319446 1.208485 -1.517504

C -0.003525 0.797356 -1.228951

C 1.032761 1.610415 -1.697448

C 0.816295 2.694430 -2.545654

C -0.491487 3.015467 -2.889734

C -1.561141 2.300051 -2.362360

Sn 0.273396 -1.102935 -0.255924

O 1.963759 0.262059 1.014031

P 2.752883 1.329029 0.284050

C 2.365752 2.988004 1.040879

C 2.778670 4.175109 0.161551

O 2.344584 1.397554 -1.301428

O -2.317842 0.507670 -0.924328

P -3.901398 1.135091 -0.762501

C -4.844142 -0.440994 -1.148797

C -6.327046 -0.161306 -0.865527

C -0.691549 -1.478458 1.609817

C -1.584763 -2.552386 1.714961

C -2.217482 -2.832032 2.926371

C -1.966753 -2.040005 4.046144

C -1.071829 -0.974877 3.954147

C -0.432778 -0.699796 2.744812

C 2.002046 -2.331931 -0.531309

C 2.385301 -2.802423 -1.791433

C 3.514419 -3.610585 -1.932229

C 4.257242 -3.976951 -0.810379

C 3.864014 -3.539207 0.454556

C 2.744507 -2.718695 0.591118

Cl -1.076178 -2.416381 -1.820231

C 4.558821 0.922531 0.151584

C 4.984752 0.271628 1.478170

C 4.734464 -0.098190 -0.986664

C 5.429952 2.146668 -0.159339

C -3.919758 1.512132 1.090342

C -4.005967 0.332870 2.058050
C -5.111970 2.459301 1.307998
C -2.613975 2.277050 1.359851
C 3.061667 3.053670 2.411846
C 0.843752 3.046609 1.272355
C -4.631455 -0.627888 -2.663578
C -4.387259 -1.718144 -0.439371
H 1.668607 3.267565 -2.912248
H -0.686020 3.854153 -3.561509
H -2.586478 2.585097 -2.595670
H 0.612093 3.964427 1.835724
H 0.487668 2.187600 1.854966
H 0.277232 3.079728 0.332497
H 2.462893 5.105959 0.659022
H 2.283684 4.141569 -0.817968
H 3.861650 4.238981 0.003452
H 2.722868 3.959211 2.939079
H 4.156107 3.113308 2.330408
H 2.802778 2.185268 3.036068
H 6.465632 1.805164 -0.312159
H 5.447814 2.880427 0.658257
H 5.110039 2.650996 -1.082943
H 6.038521 -0.037518 1.397326
H 4.379471 -0.620008 1.688627
H 4.902373 0.956405 2.333765
H 5.785430 -0.426678 -0.995135
H 4.498660 0.339510 -1.965268
H 4.108703 -0.985925 -0.845263
H -4.899087 -2.582095 -0.895978
H -4.625769 -1.722364 0.630126
H -3.308793 -1.873234 -0.566592
H -6.938807 -0.985527 -1.267097
H -6.672500 0.771668 -1.340557
H -6.533935 -0.096512 0.213817
H -5.239508 -1.476190 -3.018282
H -3.577932 -0.857753 -2.884021
H -4.928795 0.265709 -3.235066
H -2.645622 2.715234 2.371055
H -2.453577 3.098042 0.641566
H -1.747631 1.602945 1.310884
H -3.881963 0.696776 3.092011
H -3.216160 -0.405620 1.872029
H -4.980500 -0.173395 2.004851
H -5.133827 2.789501 2.359867
H -6.075092 1.969846 1.097331
H -5.042119 3.355007 0.671606

H 1.793639 -2.546468 -2.673915

H 3.811443 -3.961184 -2.923484

H 5.138646 -4.613072 -0.920688

H 4.433430 -3.836159 1.338739

H 2.455447 -2.364368 1.584315

H 0.290327 0.116127 2.684911

H -0.865975 -0.355705 4.830687

H -2.467497 -2.255278 4.992988

H -2.913826 -3.671216 2.992885

H -1.797358 -3.172239 0.840034

Total energies (in hartee) and xyz coordinates (in angstrom) of 3OS (ωB97X-D/def2-TZVP// ωB97X-D/def2-SVP).

E = -3306.62087764

C -0.350167 -1.714998 2.663752

C -0.054963 -2.187707 1.379739

C 0.535557 -3.450471 1.244252

C 0.839910 -4.216496 2.369491

C 0.567183 -3.725221 3.645159

C -0.030009 -2.473294 3.789820

Sn -0.533437 -1.096099 -0.399582

O -2.141285 0.071117 1.066763

P -2.723371 1.398117 0.623646

C -2.087322 2.761927 1.724868

C -2.839681 2.681678 3.065252

C 0.023233 0.928959 -0.953367

C 1.358210 1.282092 -1.233221

C 1.675193 2.446922 -1.938558

C 0.663899 3.317581 -2.325238

C -0.654513 3.057537 -1.977570

C -0.948397 1.886697 -1.282956

O 2.342677 0.438556 -0.811201

P 3.797372 0.805977 -0.074543

C 3.598550 -0.136542 1.530549

C 3.408223 -1.640163 1.303653

O -2.275665 1.740175 -0.911772

C -2.292474 -1.805778 -1.370497

C -3.153618 -2.669896 -0.684353

C -4.299563 -3.169190 -1.304748

C -4.597784 -2.801814 -2.616815

C -3.741476 -1.945997 -3.309942

C -2.590934 -1.456880 -2.691414

Cl 0.987901 -2.247297 -1.945893
C -4.566321 1.327027 0.439174
C -5.126329 0.531535 1.630745
C -5.211941 2.715456 0.347574
C -4.875929 0.554212 -0.854461
C 4.983257 0.044113 -1.311036
C 6.402389 0.082303 -0.728906
S 4.140388 2.701205 0.259359
C 4.597710 -1.387045 -1.709362
C 4.919343 0.930842 -2.566681
C -2.237336 4.155738 1.100891
C -0.595728 2.494053 2.002637
C 2.345288 0.457767 2.194194
C 4.805712 0.116948 2.443794
H -0.246676 3.221137 2.752534
H 0.032046 2.613253 1.110256
H -0.432416 1.485386 2.403585
H -2.371295 3.381801 3.774319
H -2.780071 1.673910 3.503382
H -3.897682 2.965034 2.977774
H -1.826582 4.898932 1.802486
H -3.280863 4.430106 0.904309
H -1.670498 4.241308 0.164298
H 4.618341 -0.371182 3.413583
H 4.959954 1.191077 2.616878
H 5.734910 -0.304953 2.036451
H 2.993629 -2.092791 2.218138
H 4.364315 -2.138984 1.092103
H 2.712071 -1.858742 0.483070
H 2.180861 -0.043007 3.160847
H 1.449078 0.290958 1.584194
H 2.456570 1.538004 2.364374
H 5.655569 0.559947 -3.296806
H 5.155403 1.979926 -2.338552
H 3.928013 0.878654 -3.040895
H 7.118328 -0.212974 -1.512221
H 6.521436 -0.624586 0.105047
H 6.670864 1.089768 -0.378194
H 5.202090 -1.677348 -2.583683
H 3.536832 -1.472474 -1.982945
H 4.807751 -2.113752 -0.915876
H 0.785278 -3.830139 0.250152
H 1.300876 -5.199324 2.246740
H 0.814340 -4.320548 4.527347
H -0.255885 -2.086278 4.786469
H -0.845834 -0.750722 2.782894

H -2.936163 -2.953555 0.349666

H -4.963544 -3.845378 -0.760868

H -5.496892 -3.187903 -3.102701

H -3.969088 -1.661421 -4.339993

H -1.922471 -0.796606 -3.251111

H -1.465379 3.742681 -2.227881

H 0.915336 4.222795 -2.880893

H 2.712841 2.678761 -2.164291

H -5.968625 0.447310 -0.938521

H -4.440263 -0.451193 -0.840843

H -4.507763 1.078519 -1.745445

H -6.206235 0.381033 1.477436

H -4.996376 1.049592 2.590538

H -4.646876 -0.454657 1.702099

H -6.286936 2.587509 0.146278

H -4.789508 3.305780 -0.478903

H -5.122328 3.293527 1.277549

Total energies (in hartee) and xyz coordinates (in angstrom) of 3^{OSe} (ωB97X-D/def2-TZVP// ωB97X-D/def2-SVP).

E = -5310.03381843

C -0.708801 -1.756200 2.692022

C -0.462966 -2.272344 1.414300

C 0.003238 -3.587677 1.295568

C 0.234936 -4.364157 2.430976

C 0.013156 -3.831945 3.700029

C -0.460824 -2.526620 3.828013

Sn -0.837650 -1.162344 -0.378644

O -2.306426 0.179166 1.076196

P -2.758853 1.550475 0.616219

C -1.973566 2.864670 1.681024

C -2.717576 2.896528 3.027960

C -0.093195 0.788016 -0.980471

C 1.263915 1.003965 -1.290921

C 1.676911 2.108059 -2.041648

C 0.746896 3.059873 -2.441384

C -0.582789 2.940007 -2.062414

C -0.974545 1.824537 -1.325901

O 2.173413 0.086710 -0.852400

P 3.656560 0.330790 -0.122678

C 3.380008 -0.597696 1.484975

C 3.042710 -2.074196 1.246547
O -2.303047 1.817861 -0.931624
C -2.657440 -1.699271 -1.347662
C -3.590846 -2.493169 -0.671378
C -4.780533 -2.874241 -1.293451
C -5.049551 -2.458020 -2.597188
C -4.121432 -1.672167 -3.280721
C -2.928591 -1.301067 -2.660574
Cl 0.564296 -2.480753 -1.907156
C -4.602022 1.659771 0.458377
C -5.221218 0.961065 1.681024
C -5.105356 3.103144 0.330845
C -5.006853 0.882160 -0.805725
C 4.767029 -0.540715 -1.364541
C 6.188480 -0.630448 -0.795008
Se 4.205650 2.330544 0.239971
C 4.248008 -1.934451 -1.746868
C 4.774610 0.331780 -2.631414
C -1.983989 4.250838 1.022861
C -0.515731 2.449073 1.955810
C 2.193888 0.106269 2.163944
C 4.610300 -0.476525 2.393978
H -0.085225 3.154671 2.683514
H 0.113269 2.479177 1.056921
H -0.455257 1.439536 2.382610
H -2.170713 3.559709 3.716007
H -2.760868 1.899052 3.490888
H -3.740311 3.288421 2.940793
H -1.497678 4.965669 1.705336
H -2.995305 4.624234 0.820546
H -1.414748 4.255673 0.083799
H 4.379373 -0.962725 3.355327
H 4.868505 0.573884 2.587694
H 5.492944 -0.978711 1.974643
H 2.592211 -2.489872 2.161408
H 3.944747 -2.661725 1.026992
H 2.323819 -2.219543 0.429260
H 1.975475 -0.402915 3.115305
H 1.286746 0.051845 1.549663
H 2.415539 1.163551 2.366506
H 5.475637 -0.110967 -3.356126
H 5.099423 1.360953 -2.421739
H 3.781958 0.354373 -3.104840
H 6.862096 -1.010460 -1.579340
H 6.246817 -1.329675 0.051495
H 6.560538 0.351504 -0.467352

H 4.807547 -2.279010 -2.631173
H 3.178661 -1.928032 -2.000176
H 4.410120 -2.673883 -0.954257
H 0.213178 -4.003382 0.306789
H 0.599223 -5.388245 2.321173
H 0.203855 -4.436319 4.590043
H -0.646326 -2.106194 4.819432
H -1.108298 -0.746902 2.798240
H -3.395719 -2.814273 0.356163
H -5.501404 -3.496167 -0.757306
H -5.982390 -2.751531 -3.084216
H -4.326556 -1.349880 -4.304378
H -2.205727 -0.693289 -3.212258
H -1.328840 3.692470 -2.320834
H 1.073475 3.918181 -3.031232
H 2.726648 2.230455 -2.295283
H -6.105787 0.885804 -0.875235
H -4.676240 -0.161957 -0.762973
H -4.598942 1.336382 -1.717629
H -6.312226 0.909313 1.541847
H -5.031857 1.495658 2.621539
H -4.837170 -0.063656 1.781575
H -6.191067 3.079428 0.148705
H -4.639094 3.621698 -0.519883
H -4.941389 3.697239 1.240456

Total energies (in hartee) and xyz coordinates (in angstrom) of 3^{PO+} (ωB97X-D/def2-TZVP// ωB97X-D/def2-SVP).

E = -2447.93930733

C -1.809394 1.989226 -2.204783
C -0.729110 1.957176 -1.314755
C 0.000190 3.129481 -1.090738
C -0.362932 4.317837 -1.723211
C -1.443053 4.341386 -2.605451
C -2.161801 3.173459 -2.853579
Sn -0.244990 0.084246 -0.417434
O 1.873068 0.782935 -0.529660
P 3.063162 0.258899 0.275095
C 4.305479 -0.560470 -0.809261
C 3.490239 -1.415877 -1.797097
C 0.183597 -0.744668 1.551374

C -0.870291 -1.070231 2.424351

C -0.665244 -1.703429 3.651717

C 0.621482 -2.052575 4.033259

C 1.695652 -1.779448 3.193000

C 1.465323 -1.143622 1.976860

O -2.186958 -0.806340 2.149659

P -2.732142 -0.178205 0.715757

C -3.594801 1.354415 1.361179

C -4.438764 1.055788 2.609408

O 2.595255 -0.992175 1.197377

C -0.109653 -1.505626 -1.818762

C 0.189897 -2.803097 -1.382825

C 0.346317 -3.839874 -2.301476

C 0.207600 -3.587206 -3.666406

C -0.085425 -2.298528 -4.110784

C -0.242008 -1.260632 -3.191081

C 3.644632 1.597726 1.406994

C 4.882353 1.166036 2.202653

C 3.936233 2.861971 0.579803

C 2.481921 1.897416 2.374211

C -3.871882 -1.504961 0.062081

C -4.090756 -1.196799 -1.429335

C -5.211596 -1.615487 0.795767

C -3.107454 -2.830959 0.206178

C 5.252461 -1.467139 -0.008608

C 5.095252 0.506325 -1.582767

C -2.463043 2.321528 1.757426

C -4.457090 1.981397 0.259006

H 5.687545 0.006284 -2.363672

H 5.800390 1.050195 -0.938173

H 4.434096 1.230898 -2.081440

H 4.191407 -1.940987 -2.462847

H 2.818265 -0.805777 -2.416020

H 2.886088 -2.174482 -1.280291

H 5.931738 -1.966939 -0.715820

H 4.702218 -2.243976 0.538805

H 5.874264 -0.910890 0.704476

H -4.867809 2.933329 0.629703

H -5.310464 1.344508 -0.015192

H -3.875220 2.207581 -0.645795

H -4.785461 2.013278 3.028358

H -3.851719 0.542394 3.382823

H -5.328403 0.455649 2.388755

H -2.904910 3.212992 2.228429

H -1.883488 2.661902 0.888885

H -1.778704 1.869505 2.492907

H -3.695418 -3.630996 -0.269398

H -2.956886 -3.099863 1.261055

H -2.130518 -2.805424 -0.295811

H 2.715830 -2.060514 3.455584

H -4.751336 -1.964205 -1.861437

H -3.145041 -1.224584 -1.993484

H -4.571948 -0.220881 -1.593137

H -5.761298 -2.482084 0.396188

H -5.846533 -0.731343 0.647756

H -5.074236 -1.778580 1.874085

H 0.861367 3.116432 -0.420953

H 0.204985 5.230743 -1.531487

H -1.721292 5.271410 -3.105200

H -3.000880 3.183148 -3.552528

H -2.396180 1.086444 -2.403172

H 0.311393 -3.014017 -0.315384

H 0.580100 -4.847838 -1.952111

H 0.330448 -4.398356 -4.387078

H -0.189390 -2.098807 -5.179380

H -0.457627 -0.252668 -3.557459

H 0.790313 -2.551811 4.988936

H -1.530244 -1.918772 4.279448

H 2.754519 2.773803 2.981337

H 2.282366 1.066086 3.064108

H 1.550824 2.139238 1.840397

H 5.104880 1.932572 2.960426

H 5.772136 1.071835 1.564312

H 4.723245 0.212966 2.728617

H 4.148653 3.691439 1.271359

H 3.075912 3.148481 -0.041075

H 4.809356 2.749381 -0.074602

Total energies (in hartee) and xyz coordinates (in angstrom) of 3^{OS+} (ωB97X-D/def2-TZVP// ωB97X-D/def2-SVP).

E = -2846.18632163

C 0.667058 2.649817 -2.159481

C 0.964048 2.395804 -0.816447

C 1.869399 3.226143 -0.142466

C 2.473478 4.292570 -0.807158

C 2.186071 4.529394 -2.151405

C 1.282535 3.709391 -2.826107

Sn 0.169208 0.732754 0.235639
O 2.285675 0.191920 0.773281
P 3.158325 -0.805094 0.011791
C 3.630557 -2.175545 1.164111
C 4.741634 -1.665858 2.098697
C -0.048285 -1.276049 -0.545837
C 1.030053 -2.009141 -1.072743
C 0.905340 -3.330128 -1.498689
C -0.303663 -3.992048 -1.325560
C -1.386204 -3.322439 -0.768707
C -1.260559 -1.976118 -0.429797
O 2.285789 -1.453981 -1.211956
O -2.390836 -1.380641 0.096020
P -3.304102 -0.173585 -0.540413
C -4.609179 -0.028941 0.783025
C -5.540672 -1.248121 0.792310
C -0.389312 0.877186 2.269702
C -0.516809 -0.278852 3.047269
C -0.928062 -0.191655 4.376738
C -1.216567 1.052732 4.936285
C -1.090411 2.209721 4.167514
C -0.679092 2.123184 2.837635
S -2.263330 1.530696 -0.714719
C -3.887577 -0.725994 -2.233106
C -5.007674 0.227136 -2.682309
C -4.394365 -2.175311 -2.240956
C -2.702206 -0.609114 -3.209916
C 4.520309 0.026661 -0.913579
C 5.578325 -0.971333 -1.403409
C 5.143919 1.083292 0.015238
C 3.892487 0.735125 -2.128025
C -3.866532 0.060651 2.127804
C -5.412908 1.264282 0.573476
C 4.081650 -3.439247 0.419447
C 2.384074 -2.493542 2.012353
H -6.123570 1.361462 1.408065
H -5.996820 1.263269 -0.355692
H -4.763167 2.150392 0.578645
H -6.174938 -1.190910 1.689743
H -4.984423 -2.195460 0.840992
H -6.212878 -1.267325 -0.076669
H -4.613628 0.226560 2.918418
H -3.158479 0.900695 2.153768
H -3.320591 -0.862276 2.360443
H 2.645689 -3.289997 2.725600
H 1.543421 -2.859386 1.404363

H 2.051806 -1.615694 2.582115

H 4.911867 -2.418294 2.883599

H 4.458414 -0.726033 2.595566

H 5.697032 -1.518174 1.576251

H 4.338540 -4.208995 1.163230

H 4.968976 -3.277579 -0.204453

H 3.281425 -3.846563 -0.212151

H 4.683019 1.309308 -2.634107

H 3.107698 1.442921 -1.833150

H 3.472142 0.020464 -2.847588

H 6.307353 -0.427627 -2.022965

H 5.139003 -1.761718 -2.029602

H 6.136937 -1.437368 -0.580007

H -2.335282 -3.828260 -0.592635

H 5.910847 1.633095 -0.550819

H 5.633421 0.645130 0.895032

H 4.390116 1.805450 0.357028

H 2.122518 3.036967 0.904494

H 3.173629 4.939555 -0.274289

H 2.664646 5.360280 -2.673894

H 1.054106 3.895796 -3.877521

H -0.045414 2.020363 -2.698578

H -0.312560 -1.262801 2.615935

H -1.029264 -1.098450 4.977052

H -1.541171 1.121658 5.976637

H -1.315354 3.184683 4.604988

H -0.598889 3.038378 2.243898

H -0.401224 -5.038328 -1.619772

H 1.769899 -3.827884 -1.938692

H -3.043143 -0.949016 -4.199821

H -1.851503 -1.242174 -2.918602

H -2.351932 0.426864 -3.303940

H -5.223881 0.032648 -3.743758

H -4.716724 1.284267 -2.591126

H -5.939657 0.062681 -2.123714

H -4.765454 -2.398628 -3.252950

H -5.221010 -2.352601 -1.544743

H -3.592153 -2.890324 -2.025967

Total energies (in hartee) and xyz coordinates (in angstrom) of 3^{OSe+} (ωB97X-D/def2-TZVP// ωB97X-D/def2-SVP).

E = -4849.59851546

C -0.345767 -0.512353 3.043010

C -0.253257 0.690236 2.334452

C -0.549264 1.894156 2.983836

C -0.931122 1.893342 4.325323

C -1.022124 0.690113 5.024909

C -0.727974 -0.512993 4.384022

Sn 0.272087 0.675856 0.284558

C 1.087084 2.401228 -0.651802

C 1.973766 3.192843 0.090288

C 2.580623 4.304227 -0.493345

C 2.314731 4.626263 -1.824329

C 1.430662 3.844869 -2.567027

C 0.812994 2.739541 -1.981430

C 0.073855 -1.290154 -0.607366

C 1.154845 -1.972097 -1.194775

C 1.042817 -3.265940 -1.700374

C -0.149573 -3.960298 -1.541245

C -1.230055 -3.346247 -0.920161

C -1.123012 -2.018672 -0.508477

O 2.401295 -1.391780 -1.312253

P 3.278317 -0.829878 -0.047149

C 4.636790 0.068557 -0.915213

C 5.256802 1.061285 0.083964

O -2.251643 -1.477752 0.079525

P -3.253490 -0.303002 -0.479435

C -3.888510 -0.831785 -2.166054

C -4.358504 -2.293372 -2.192718

O 2.403918 0.103202 0.787603

Se -2.236356 1.591374 -0.647581

C 3.763747 -2.278419 1.001135

C 4.220247 -3.483824 0.168494

C 2.524078 -2.664097 1.830851

C 4.875512 -1.829323 1.965720

C -4.506045 -0.275667 0.907208

C -5.392224 0.973141 0.781679

C -3.709315 -0.193062 2.220701

C -5.366496 -1.546280 0.912585

C -5.052043 0.098575 -2.547136

C -2.746128 -0.662281 -3.185567

C 5.698786 -0.888868 -1.472926

C 4.008044 0.856772 -2.078535

H -5.949513 -1.562522 1.845722

H -4.757634 -2.461565 0.887916

H -6.086197 -1.566795 0.082770

H -6.081219 0.986895 1.639676

H -6.003816 0.978940 -0.129569

H -4.799486 1.898004 0.812771

H -4.427830 -0.091038 3.047804

H -3.040386 0.678692 2.248378

H -3.110730 -1.095677 2.395647

H 4.798117 1.463739 -2.545619

H 3.223795 1.542729 -1.735727

H 3.586794 0.192465 -2.844337

H 6.020567 1.652469 -0.443371

H 5.749145 0.565508 0.931015

H 4.499970 1.754448 0.475526

H 6.424661 -0.300512 -2.054224

H 5.262393 -1.636280 -2.151732

H 6.260438 -1.407895 -0.684101

H -2.162400 -3.883181 -0.748235

H 2.210055 2.938242 1.127237

H 3.266153 4.920132 0.092741

H 2.795236 5.492922 -2.283008

H 1.220051 4.096310 -3.608580

H 0.119153 2.139173 -2.575897

H -0.496711 2.845149 2.445712

H -1.160547 2.836279 4.826122

H -1.323556 0.690592 6.074488

H -0.801864 -1.456218 4.929745

H -0.137100 -1.464764 2.547901

H -0.234842 -4.989328 -1.894224

H 1.906388 -3.720176 -2.186871

H -3.117743 -0.991515 -4.168015

H -1.868456 -1.278145 -2.941395

H -2.425516 0.383850 -3.272917

H -4.743028 -2.504706 -3.202235

H -5.167427 -2.504958 -1.486007

H -3.535737 -2.992655 -2.008176

H -5.303920 -0.078260 -3.603798

H -4.790842 1.162459 -2.443738

H -5.956151 -0.106981 -1.957291

H 5.052676 -2.634163 2.695179

H 4.588960 -0.928449 2.528465

H 5.828175 -1.639898 1.451965

H 4.485893 -4.301455 0.856035

H 5.103562 -3.273035 -0.446170

H 3.420244 -3.852491 -0.486474

H 2.794120 -3.508406 2.483341

H 1.681261 -2.989123 1.203027

H 2.191806 -1.830943 2.463958

Total energies (in hartee) and xyz coordinates (in angstrom) of Ar^{PP} (ωB97X-D/def2-TZVP// ωB97X-D/def2-SVP).

E = -4269.33287300

C -1.213261 -1.515274 1.893334

C -0.000004 -1.841208 2.490486

C 1.213249 -1.515237 1.893339

C 1.220667 -0.841304 0.667681

C -0.000005 -0.504933 0.062122

C -1.220680 -0.841345 0.667650

O 2.348591 -0.506347 0.010519

P 3.842285 -0.185400 0.757076

C 3.910810 1.671167 0.436379

C 3.901324 2.110629 -1.028099

Br -0.000001 0.413416 -1.584945

O -2.348603 -0.506416 0.010483

P -3.842279 -0.185425 0.757066

C -3.910755 1.671160 0.436439

C -3.901346 2.110648 -1.028033

C 4.887551 -1.133000 -0.494044

C 4.423915 -1.038883 -1.952155

C 4.753061 -2.596627 -0.030555

C 6.350778 -0.693611 -0.361470

C -4.887599 -1.132945 -0.494072

C -4.423954 -1.038789 -1.952177

C -4.753148 -2.596594 -0.030643

C -6.350818 -0.693530 -0.361474

C 2.652617 2.224186 1.132665

C 5.152671 2.214398 1.160555

C -5.152561 2.214406 1.160691

C -2.652518 2.224135 1.132678

H -2.161646 -1.772246 2.366292

H 0.000010 -2.370089 3.445868

H 2.161628 -1.772189 2.366315

H -5.149777 3.316144 1.119490

H -5.164524 1.919565 2.221931

H -6.091112 1.871644 0.702215

H -2.704993 3.324779 1.163134

H -1.736738 1.947661 0.590077

H -2.562170 1.863393 2.170110

H -3.774531 3.205037 -1.084747

H -4.844500 1.865438 -1.537379

H -3.069598 1.646209 -1.577793

H -5.344371 -3.253789 -0.689407

H -5.114252 -2.733298 1.000455

H -3.705848 -2.934679 -0.074923

H -6.999553 -1.397580 -0.907886

H -6.518644 0.305990 -0.789295

H -6.687343 -0.681283 0.688361

H -4.980073 -1.774597 -2.556956

H -3.351557 -1.258232 -2.045301

H -4.607569 -0.049757 -2.388576

H 2.705090 3.324832 1.163041

H 2.562341 1.863515 2.170131

H 1.736803 1.947656 0.590158

H 5.149923 3.316136 1.119314

H 6.091199 1.871597 0.702060

H 5.164666 1.919611 2.221810

H 3.774492 3.205017 -1.084808

H 3.069544 1.646183 -1.577807

H 4.844443 1.865414 -1.537508

H 5.344358 -3.253836 -0.689239

H 3.705771 -2.934725 -0.074937

H 5.114066 -2.733278 1.000585

H 6.999499 -1.397713 -0.907831

H 6.687294 -0.681281 0.688368

H 6.518614 0.305868 -0.789380

H 4.979930 -1.774811 -2.556883

H 4.607685 -0.049915 -2.388629

H 3.351485 -1.258179 -2.045280

Total energies (in hartee) and xyz coordinates (in angstrom) of Ar^{OO} (ωB97X-D/def2-TZVP// ωB97X-D/def2-SVP).

E = -1846.31288259

C -0.000014 -0.000060 2.781540

C -1.198834 -0.222850 2.106996

C -1.185050 -0.213383 0.710710

C -0.000012 -0.000020 0.008281

C 1.185029 0.213330 0.710715

C 1.198815 0.222768 2.107001

O -2.303317 -0.442943 -0.045020

P -3.867224 -0.017236 0.277477

O -4.250128 -0.024123 1.721920

O 2.303294 0.442918 -0.045010

P 3.867208 0.017233 0.277486

O 4.250108 0.024107 1.721930

C -4.001566 1.697103 -0.424821
C -5.377189 2.266150 -0.042715
C -4.707023 -1.364099 -0.681938
C -4.062993 -1.654694 -2.043222
C -3.785459 1.753042 -1.939474
C -2.912885 2.524340 0.285995
C 4.706988 1.364125 -0.681909
C 4.062957 1.654727 -2.043192
C 4.001621 -1.697083 -0.424853
C 2.912960 -2.524377 0.285927
C 4.578137 2.607793 0.218024
C 6.191285 1.009325 -0.850336
C 3.785538 -1.752992 -1.939509
C 5.377257 -2.266090 -0.042742
C -4.578196 -2.607783 0.217979
C -6.191315 -1.009272 -0.850365
H -0.000012 -0.000009 -1.082050
H 2.137296 0.386188 2.636074
H -0.000021 -0.000069 3.873751
H -2.137317 -0.386264 2.636067
H 4.569313 2.521419 -2.498339
H 2.997274 1.897853 -1.937090
H 4.156923 0.814768 -2.743863
H 5.088798 3.456719 -0.263769
H 5.028613 2.433606 1.204389
H 3.523717 2.884037 0.367429
H 6.735144 1.889499 -1.228549
H 6.341140 0.195347 -1.575459
H 6.648275 0.716085 0.106831
H 3.056700 -3.589006 0.042015
H 1.900453 -2.240864 -0.034973
H 2.974160 -2.411397 1.378122
H 3.731988 -2.805744 -2.260780
H 4.613071 -1.284941 -2.492735
H 2.845609 -1.263006 -2.234558
H 5.422012 -3.328894 -0.330184
H 5.542397 -2.189877 1.041086
H 6.202074 -1.750077 -0.553305
H -5.421901 3.328962 -0.330130
H -5.542347 2.189916 1.041108
H -6.202017 1.750179 -0.553302
H -3.731849 2.805799 -2.260715
H -4.613010 1.285050 -2.492724
H -2.845543 1.263030 -2.234522
H -3.056583 3.588981 0.042108
H -1.900392 2.240787 -0.034910

H -2.974103 2.411335 1.378185

H -4.569351 -2.521370 -2.498398

H -2.997314 -1.897842 -1.937139

H -4.156944 -0.814724 -2.743882

H -5.088884 -3.456688 -0.263818

H -5.028671 -2.433601 1.204345

H -3.523779 -2.884047 0.367383

H -6.735191 -1.889438 -1.228574

H -6.341155 -0.195294 -1.575490

H -6.648303 -0.716037 0.106805

Total energies (in hartee) and xyz coordinates (in angstrom) of Ar^{SS} (ωB97X-D/def2-TZVP// ωB97X-D/def2-SVP).

E = -2492.22086258

C 1.194362 -2.182370 0.227539

C 0.000124 -2.861036 -0.003916

C -1.194353 -2.182273 -0.233879

C -1.185643 -0.787346 -0.217804

C -0.000343 -0.086642 -0.000842

C 1.185206 -0.787412 0.214595

O -2.306005 -0.046901 -0.481454

P -3.810742 -0.102223 0.218415

C -4.827192 -0.025571 -1.356486

C -6.285565 0.296364 -1.007633

O 2.305239 -0.047012 0.479728

P 3.810461 -0.101296 -0.219199

C 3.792476 1.482109 -1.226855

C 5.105272 1.618916 -2.010498

S -4.210643 -1.654312 1.327590

C -3.790846 1.477059 1.232516

C -2.630549 1.316465 2.231801

S 4.209288 -1.649296 -1.334474

C 4.826361 -0.031954 1.356404

C 6.285077 0.290394 1.009327

C -5.103668 1.612496 2.016353

C -3.550417 2.722883 0.372761

C -4.278003 0.985182 -2.373795

C -4.742468 -1.431174 -1.977752

C 4.277551 0.974965 2.377717

C 4.740469 -1.440021 1.971854

C 2.631638 1.327088 -2.226370

C 3.554032 2.724776 -0.361992

H -0.000546 1.003869 0.000376

H 2.125208 -2.721477 0.397709

H 0.000328 -3.952791 -0.005147

H -2.125039 -2.721264 -0.405322

H 4.843784 0.863182 3.316181

H 3.217333 0.789449 2.595544

H 4.388979 2.016242 2.053188

H 5.318932 -1.452829 2.909079

H 5.147080 -2.204306 1.296252

H 3.700792 -1.707698 2.214677

H 6.912044 0.135440 1.901571

H 6.411338 1.337995 0.698209

H 6.663484 -0.363693 0.209532

H 2.638552 2.188054 -2.913447

H 1.655175 1.305321 -1.723272

H 2.734204 0.408307 -2.820738

H 3.350131 3.584022 -1.020732

H 4.435782 2.981977 0.241684

H 2.690689 2.599846 0.307550

H 5.037388 2.501778 -2.666244

H 5.286970 0.734482 -2.636668

H 5.974355 1.760354 -1.353601

H -5.034594 2.492358 2.675994

H -5.286933 0.725603 2.638584

H -5.972315 1.758228 1.359823

H -3.345329 3.579142 1.035012

H -4.431663 2.983879 -0.230012

H -2.687171 2.599370 -0.297167

H -2.636510 2.174763 2.922218

H -1.653906 1.295323 1.729028

H -2.734612 0.395527 2.822547

H -4.844477 0.877541 -3.312598

H -3.217918 0.799969 -2.592526

H -4.388841 2.025184 -2.045001

H -5.320938 -1.439610 -2.915021

H -5.149773 -2.197880 -1.305325

H -3.703015 -1.698782 -2.221606

H -6.912934 0.145396 -1.900277

H -6.410981 1.342801 -0.692296

H -6.664155 -0.360639 -0.210318

Total energies (in hartee) and xyz coordinates (in angstrom) of Ar^{SeSe} (ωB97X-D/def2-TZVP// ωB97X-D/def2-SVP).

E = -6499.04650213

C 1.177648 -1.986027 0.301812

C 0.000009 -2.665606 -0.000641

C -1.177682 -1.986053 -0.302958

C -1.169978 -0.591339 -0.287781

C -0.000110 0.109466 -0.000323

C 1.169818 -0.591306 0.286998

O -2.272795 0.147232 -0.622118

P -3.810575 0.127749 0.006144

C -4.750920 0.096502 -1.623245

C -6.223232 0.446240 -1.375636

O 2.272509 0.147379 0.621489

P 3.810537 0.127767 -0.006166

C 3.840244 1.783342 -0.900759

C 5.192156 1.988350 -1.597632

Se -4.294673 -1.455326 1.294466

C -3.839271 1.782112 0.903002

C -2.735426 1.702739 1.972864

Se 4.294124 -1.453808 -1.296506

C 4.750508 0.093836 1.623395

C 6.222951 0.443566 1.376564

C -5.190865 1.986751 1.600608

C -3.544142 2.955518 -0.038732

C -4.142427 1.037143 -2.674362

C -4.643115 -1.344243 -2.152521

C 4.142039 1.033081 2.675771

C 4.642258 -1.347660 2.150519

C 2.736745 1.705834 -1.971115

C 3.545239 2.955635 0.042401

H -0.000143 1.199824 -0.000214

H 2.096038 -2.525673 0.529128

H 0.000067 -3.757255 -0.000780

H -2.096058 -2.525675 -0.530406

H 4.658935 0.861250 3.633330

H 3.072975 0.832208 2.825549

H 4.265713 2.093015 2.425823

H 5.169280 -1.409935 3.115691

H 5.091611 -2.071792 1.457892

H 3.593494 -1.633542 2.322993

H 6.800274 0.236307 2.291380

H 6.357437 1.509141 1.139261

H 6.651625 -0.157440 0.560616

H 2.764075 2.628466 -2.572296

H 1.735944 1.623542 -1.525830

H 2.886615 0.849893 -2.643939

H 3.367488 3.860176 -0.560811

H 4.392659 3.172315 0.707647

H 2.649733 2.776268 0.654661

H 5.151858 2.924785 -2.176927

H 5.413689 1.163245 -2.288701

H 6.024517 2.074855 -0.886148

H -5.149959 2.922361 2.181196

H -5.412530 1.160791 2.290612

H -6.023434 2.074603 0.889537

H -3.365713 3.859178 0.565600

H -4.391751 3.173431 -0.703334

H -2.648996 2.776545 -0.651635

H -2.762176 2.624599 2.575257

H -1.734799 1.620600 1.527170

H -2.885430 0.845972 2.644604

H -4.659687 0.866982 -3.632025

H -3.073487 0.836118 -2.824795

H -4.265639 2.096735 -2.422723

H -5.170163 -1.404917 -3.117782

H -5.092706 -2.069251 -1.460969

H -3.594436 -1.630184 -2.325387

H -6.800792 0.240352 -2.290610

H -6.357423 1.511529 -1.136882

H -6.651887 -0.155747 -0.560401

Total energies (in hartee) and xyz coordinates (in angstrom) of Ar^{OP} (ωB97X-D/def2-TZVP// ωB97X-D/def2-SVP).

E = -1771.01537220

C -0.105956 -0.599547 2.728463

C 1.104869 -0.260878 2.129387

C 1.090226 0.124656 0.786690

C -0.101290 0.180004 0.067040

C -1.304563 -0.159668 0.689285

C -1.311307 -0.556821 2.030242

O 2.221765 0.501535 0.113422

P 3.758022 -0.082105 0.275562

O 4.139240 -0.519977 1.652741

O -2.418414 -0.109306 -0.086544

P -3.988108 0.115287 0.516896

C -4.385770 1.713987 -0.400614

C -3.307137 2.701283 0.083222

C 3.799511 -1.519905 -0.899753

C 5.139829 -2.249883 -0.719004

C 4.673357 1.439766 -0.259542

C 4.048563 2.150498 -1.466471

C 3.585674 -1.117745 -2.361444

C 2.663767 -2.458485 -0.448018

C -4.774284 -1.371376 -0.341634

C -4.236531 -1.686656 -1.741388

C -4.423068 -2.546539 0.591539

C -6.296718 -1.192244 -0.374998

C -4.353287 1.645538 -1.927936

C -5.755691 2.206782 0.090866

C 4.611609 2.369967 0.966880

C 6.136076 1.071306 -0.545811

H -0.097941 0.486037 -0.979486

H -2.250328 -0.820266 2.518391

H -0.110142 -0.908203 3.776280

H 2.049783 -0.303497 2.670072

H -4.633181 -2.661159 -2.072495

H -3.139558 -1.747165 -1.740201

H -4.536495 -0.939895 -2.486435

H -4.846055 -3.482865 0.191449

H -4.825430 -2.396707 1.605366

H -3.332818 -2.681812 0.673198

H -6.776790 -2.147187 -0.645024

H -6.605272 -0.447168 -1.123299

H -6.699817 -0.885752 0.604295

H -3.546775 3.714687 -0.278420

H -2.311354 2.431278 -0.297096

H -3.251938 2.740868 1.183046

H -4.428068 2.662806 -2.348117

H -5.194853 1.064003 -2.331492

H -3.414418 1.199651 -2.287804

H -5.946741 3.218426 -0.303795

H -5.794525 2.264168 1.190119

H -6.580563 1.562846 -0.245105

H 5.126499 -3.182412 -1.305906

H 5.304891 -2.503733 0.337454

H 5.993991 -1.652349 -1.066374

H 3.474931 -2.025640 -2.976179

H 4.439793 -0.553465 -2.764043

H 2.675308 -0.512876 -2.487731

H 2.749727 -3.411964 -0.993158

H 1.669968 -2.039258 -0.660439

H 2.726343 -2.671910 0.629025

H 4.601719 3.085054 -1.654502

H 2.997285 2.406592 -1.278853

H 4.098413 1.549493 -2.383840
H 5.169568 3.294228 0.747728
H 5.049495 1.890213 1.852496
H 3.573725 2.646376 1.205271
H 6.728542 1.993332 -0.657419
H 6.242108 0.498998 -1.479677
H 6.574246 0.486237 0.276834

Total energies (in hartee) and xyz coordinates (in angstrom) of Ar^{OS} (ωB97X-D/def2-TZVP// ωB97X-D/def2-SVP).

E = -2169.26674253

C 1.071839 -1.299137 -1.749046
C -0.128536 -1.431105 -2.444240
C -1.320947 -0.894417 -1.963572
C -1.299874 -0.215291 -0.743569
C -0.111058 -0.062619 -0.031852
C 1.070102 -0.598363 -0.542873
O -2.412240 0.360815 -0.192137
P -3.982869 -0.147737 -0.272516
C -4.803401 1.506430 -0.102053
C -6.291472 1.297463 0.213527
O 2.195018 -0.468319 0.226639
P 3.697156 0.120315 -0.163228
C 3.632401 1.834457 0.599458
C 4.945489 2.580224 0.323708
O -4.366388 -0.847319 -1.536183
C -4.137842 -1.294231 1.180607
C -3.058617 -2.374652 0.974415
S 4.152854 0.204501 -2.056880
C 4.706187 -1.141007 0.791128
C 6.155606 -0.653266 0.907035
C -5.520132 -1.963239 1.118556
C -3.923075 -0.601102 2.528715
C -4.152338 2.420326 0.943554
C -4.661518 2.146045 -1.496302
C 4.126066 -1.446033 2.179772
C 4.660504 -2.431247 -0.046877
C 2.485205 2.569217 -0.118390
C 3.345804 1.795049 2.104522
H -0.110634 0.465203 0.922374
H 2.000273 -1.715160 -2.137539

H -0.135511 -1.971006 -3.393414

H -2.261789 -1.006311 -2.501863

H 4.698912 -2.276968 2.621739

H 3.073645 -1.753080 2.117351

H 4.200459 -0.597060 2.869549

H 5.230942 -3.214464 0.477026

H 5.097498 -2.283625 -1.043391

H 3.627752 -2.791101 -0.170729

H 6.785004 -1.475289 1.282993

H 6.251886 0.183275 1.614907

H 6.554427 -0.338026 -0.068777

H 2.472157 3.618100 0.217940

H 1.504786 2.130119 0.111883

H 2.621299 2.556760 -1.208907

H 3.115970 2.815357 2.450833

H 4.214378 1.445725 2.680437

H 2.483344 1.155384 2.341662

H 4.852055 3.613149 0.695786

H 5.161254 2.617421 -0.753035

H 5.804803 2.121676 0.831771

H -5.578549 -2.746664 1.891223

H -5.683484 -2.428052 0.136218

H -6.338772 -1.254040 1.303506

H -3.883260 -1.360635 3.326149

H -4.744647 0.087590 2.775002

H -2.977015 -0.039793 2.551232

H -3.213878 -3.180344 1.709495

H -2.043027 -1.979538 1.119718

H -3.118821 -2.812694 -0.032531

H -4.648748 3.403866 0.914067

H -3.084712 2.569873 0.734218

H -4.252573 2.032681 1.965811

H -5.160697 3.128044 -1.495541

H -5.116514 1.515299 -2.271749

H -3.604249 2.301198 -1.758097

H -6.823410 2.256510 0.109832

H -6.449268 0.945161 1.244006

H -6.754742 0.578356 -0.478774

Total energies (in hartee) and xyz coordinates (in angstrom) of Ar^{OSe} (ωB97X-D/def2-TZVP// ωB97X-D/def2-SVP).

E = -4172.67959647

C 0.329150 0.869282 -2.593037
C 1.537661 0.436861 -2.051682
C 1.566111 0.055739 -0.708653
C 0.409056 0.090073 0.068081
C -0.789455 0.515591 -0.502114
C -0.839428 0.920413 -1.835951
O 2.698684 -0.396806 -0.087783
P 4.263212 0.078908 -0.330553
O 4.595859 0.497883 -1.725784
O -1.879536 0.590787 0.323069
P -3.399342 -0.059896 0.164601
Se -3.919416 -0.836068 -1.713649
C 4.465784 1.504187 0.843285
C 5.841936 2.140844 0.592192
C 5.097418 -1.505210 0.151993
C 4.486854 -2.179487 1.386880
C 4.302768 1.110322 2.313775
C 3.375258 2.520837 0.453677
C -4.378963 1.472188 0.648590
C -3.761605 2.215908 1.842479
C -3.340952 -1.407842 1.476498
C -2.218760 -2.369345 1.045259
C -4.336660 2.408605 -0.571655
C -5.831089 1.082637 0.949791
C -3.017002 -0.847930 2.866565
C -4.667958 -2.178006 1.508988
C 4.910423 -2.420827 -1.072714
C 6.594313 -1.238709 0.365517
H 0.449168 -0.202394 1.117756
H -1.779864 1.254013 -2.272378
H 0.297251 1.176732 -3.640310
H 2.454672 0.408247 -2.639579
H -4.308004 3.162446 1.981167
H -2.704860 2.455635 1.665055
H -3.838757 1.655905 2.781482
H -4.884765 3.332003 -0.326327
H -4.799557 1.949654 -1.455455
H -3.303081 2.689152 -0.825531
H -6.437704 1.997474 1.041004
H -5.920598 0.534829 1.899393
H -6.261630 0.468058 0.145027
H -2.194255 -3.218446 1.746600
H -1.231923 -1.887120 1.065751
H -2.390182 -2.761525 0.033060

H -2.787489 -1.687126 3.542363

H -3.868686 -0.306546 3.301483

H -2.143443 -0.180494 2.847835

H -4.575510 -3.009732 2.225797

H -4.910020 -2.596740 0.522324

H -5.510506 -1.552758 1.834220

H 5.924591 3.068807 1.180625

H 5.967611 2.388707 -0.471064

H 6.669894 1.483700 0.892050

H 4.288056 2.020532 2.934821

H 5.135810 0.486300 2.669561

H 3.360858 0.568919 2.487862

H 3.554302 3.463080 0.995625

H 2.367436 2.169093 0.716651

H 3.396361 2.735859 -0.624641

H 4.984599 -3.150112 1.544187

H 3.412804 -2.364022 1.251358

H 4.622941 -1.589034 2.302373

H 5.413319 -3.382676 -0.884517

H 5.335943 -1.966966 -1.977768

H 3.845442 -2.623899 -1.259897

H 7.126994 -2.199564 0.446091

H 6.785253 -0.679460 1.293766

H 7.030391 -0.681090 -0.477047

Total energies (in hartee) and xyz coordinates (in angstrom) of $SnPhCl_3$ (ωB97X-D/def2-TZVP// ωB97X-D/def2-SVP).

E = -1826.79732763

C -4.176189 -0.000295 0.054266

C -3.481714 -1.209559 0.042032

C -2.087724 -1.214421 0.022548

C -1.393018 -0.000159 0.020737

C -2.087834 1.214041 0.022821

C -3.481820 1.209050 0.042326

Sn 0.720852 0.000006 -0.004245

Cl 1.521326 -1.907317 -1.094418

Cl 1.701506 -0.000810 2.118468

Cl 1.520163 1.908656 -1.092937

H -1.551043 2.165869 -0.000420

H -4.026936 2.155167 0.044134

H -5.268168 -0.000354 0.068545

H -4.026728 -2.155735 0.043611

H -1.550838 -2.166186 -0.000940

Total energies (in hartee) and xyz coordinates (in angstrom) of $SnPh_2Cl_2$ (ωB97X-D/def2-TZVP// ωB97X-D/def2-SVP).

E = -1598.18924862

C 1.802541 -1.659958 -0.835961

C 1.788540 -0.469847 -0.096996

C 2.942708 -0.061272 0.581072

C 4.102122 -0.832608 0.511401

C 4.113154 -2.013826 -0.230061

C 2.964153 -2.428804 -0.902325

Sn -0.000005 0.666596 -0.000067

Cl 0.233112 2.103256 1.857146

C -1.788500 -0.469953 0.096765

C -1.802256 -1.660200 0.835522

C -2.963800 -2.429106 0.902043

C -4.112994 -2.014084 0.230136

C -4.102218 -0.832740 -0.511129

C -2.942859 -0.061340 -0.580945

Cl -0.233342 2.103783 -1.856772

H -0.906420 -1.999919 1.363870

H -2.970298 -3.355719 1.480095

H -5.022516 -2.616591 0.282616

H -5.001310 -0.509936 -1.040479

H -2.937528 0.858116 -1.172199

H 2.937195 0.858090 1.172471

H 5.001063 -0.509845 1.041035

H 5.022720 -2.616279 -0.282405

H 2.970833 -3.355316 -1.480540

H 0.906853 -1.999629 -1.364591

Total energies (in hartee) and xyz coordinates (in angstrom) of $SnPh_3Cl$ (ωB97X-D/def2-TZVP// ωB97X-D/def2-SVP).

E = -1369.575193

C	0.000000	0.000000	0.000000	C	-0.751614	-2.086296	4.513412
C	0.000000	0.000000	1.401458	C	-0.538164	-2.681847	5.756660
C	1.225305	0.000000	2.081690	C	-1.088975	-2.113385	6.904422
C	2.427271	0.005245	1.374193	C	-1.850053	-0.948899	6.807576
C	2.415491	0.008284	-0.020316	C	-2.063959	-0.353192	5.564684
C	1.201986	0.005279	-0.707484	H	-0.303061	-2.542462	3.625151
Sn	-1.835928	-0.048334	2.482183	H	0.062325	-3.591540	5.828849
Cl	-2.627295	2.146948	2.947837	H	-0.921676	-2.578079	7.878753
C	-3.363411	-0.918663	1.272433	H	-2.278520	-0.498880	7.706033
C	-3.085341	-2.086382	0.548222	H	-2.652634	0.566042	5.500618
C	-4.068715	-2.682128	-0.241700	H	-4.871740	0.565191	1.731974
C	-5.339759	-2.114065	-0.316825	H	-6.618357	-0.500082	0.334655
C	-5.624700	-0.949779	0.395847	H	-6.110426	-2.578902	-0.935895
C	-4.641860	-0.353880	1.185994	H	-3.840190	-3.591660	-0.801939
C	-1.519432	-0.918361	4.404962	H	-2.090850	-2.542221	0.586863

H 1.248727 -0.006631 3.175574

H 3.376897 0.006866 1.914058

H 3.356897 0.012194 -0.574420

H 1.190912 0.006911 -1.799784

H -0.945021 -0.006615 -0.551421

Total energies (in hartee) and xyz coordinates (in angstrom) of $SnPhCl_2^+$ (ωB97X-D/def2-TZVP// ωB97X-D/def2-SVP).

E = -1366.23938674

C -1.939165 -1.231715 -0.000022

C -1.265817 -0.000368 -0.000011

C -1.938820 1.231221 0.000060

C -3.330001 1.215937 0.000099

C -4.017729 0.000111 0.000027

C -3.330384 -1.215932 -0.000066

Sn 0.807764 -0.000025 -0.000039

Cl 2.064978 1.900820 0.000006

Cl 2.065714 -1.900474 0.000081

H -1.405035 2.184537 -0.000013

H -3.878813 2.159213 0.000121

H -5.109525 0.000274 0.000009

H -3.879494 -2.159036 -0.000171

H -1.405616 -2.185157 -0.000012

Total energies (in hartee) and xyz coordinates (in angstrom) of $SnPh_2Cl^+$ (ωB97X-D/def2-TZVP// ωB97X-D/def2-SVP).

E = -1137.65768086

C -2.127212 -1.714408 -0.000084

C -1.927546 -0.322602 0.000010

C -3.016028 0.564884 0.000118

C -4.308481 0.049034 0.000131

C -4.510464 -1.331994 0.000038

C -3.426011 -2.211824 -0.000069

Sn 0.000001 0.455284 -0.000041

Cl 0.000075 2.762190 -0.000016

C 1.927506 -0.322690 -0.000008

C 2.127203 -1.714484 0.000023

C 3.426008 -2.211848 0.000045

C 4.510441 -1.331993 0.000056

C 4.308426 0.049029 0.000046

C 3.015957 0.564850 0.000024

H 1.289169 -2.415368 0.000047

H 3.593488 -3.290201 0.000053

H 5.527402 -1.729223 0.000073

H 5.161837 0.729181 0.000071

H 2.864560 1.646977 0.000000

H -2.864641 1.647012 0.000191

H -5.161908 0.729166 0.000198

H -5.527423 -1.729229 0.000049

H -3.593466 -3.290180 -0.000141

H -1.289147 -2.415260 -0.000201

Total energies (in hartee) and xyz coordinates (in angstrom) of $SnPh_3^+$ (ωB97X-D/def2-TZVP// ωB97X-D/def2-SVP).

E = -909.060934867

C 2.372326 -1.861212 -0.453190

C 2.009229 -0.580496 0.000009

C 2.998622 0.310135 0.453118

C 4.334109 -0.082488 0.455431

C 4.688241 -1.353237 0.000063

C 3.711814 -2.240214 -0.455358

Sn 0.000424 -0.000381 -0.000287

C -0.501716 2.029275 0.000434

C -1.767855 2.440778 0.453140

C -2.095584 3.793724 0.455218

C -1.171944 4.735731 0.000133

C 0.084598 4.333496 -0.454713

C 0.426049 2.983995 -0.452373

C -1.507039 -1.449274 -0.000294

C -2.797259 -1.122471 -0.454069

C -3.796168 -2.091991 -0.456002

C -3.517771 -3.381103 0.000373

C -2.240645 -3.710728 0.456370

C -1.231914 -2.751421 0.453795

H -2.500392 1.715033 0.816306

H -3.075568 4.114915 0.812689

H -1.434379 5.795556 0.000004

H 0.801488 5.074828 -0.812195

H 1.412668 2.683939 -0.815308

H -0.237560 -3.023502 0.817601

H -2.030004 -4.719834 0.814983

H -4.305076 -4.137560 0.000570

H -4.796170 -1.841276 -0.814352

H -3.029433 -0.118114 -0.818165

H 2.736340 1.307365 0.816409

H 5.102208 0.605511 0.813159

H 5.737310 -1.655815 0.000088

H 3.995522 -3.231532 -0.813256

H 1.619295 -2.565429 -0.816679

Total energies (in hartee) and xyz coordinates (in angstrom) of PhH (ωB97X-D/def2-TZVP// ωB97X-D/def2-SVP). E= -232.2458959

C	0.000000	0.000000	0.000000
C	0.000000	0.000000	1.395038
C	1.208138	0.000000	2.092557
C	2.416260	0.000000	1.394991
C	2.416260	0.000000	0.000006
C	1.208168	0.000000	-0.697486
H	-0.946062	0.000000	1.941247
H	1.208329	0.000000	3.185001
H	3.362304	0.000000	1.941190
H	3.362347	0.000000	-0.546217
H	1.208168	0.000000	-1.789883
H	-0.945989	0.000000	-0.546387

References.

[S1] Sheldrick, G.M. *SHELXT* - Integrated space-group and crystal-structure determination. *Acta Cryst.* **2015**, *A71*, 3.

[S2] Spek, A.L. PLATON SQUEEZE: a tool for the calculation of the disordered solvent contribution to the calculated structure factors. *Acta Cryst.* **2015**, *C71*, 9.

[S3] Gaussian 16, Revision B.01, M. J. Frisch, G. W. Trucks, H. B. Schlegel, G. E. Scuseria, M. A. Robb, J. R. Cheeseman, G. Scalmani, V. Barone, G. A. Petersson, H. Nakatsuji, X. Li, M. Caricato, A. V. Marenich, J. Bloino, B. G. Janesko, R. Gomperts, B. Mennucci, H. P. Hratchian, J. V. Ortiz, A. F. Izmaylov, J. L. Sonnenberg, D. Williams-Young, F. Ding, F. Lipparini, F. Egidi, J. Goings, B. Peng, A. Petrone, T. Henderson, D. Ranasinghe, V. G. Zakrzewski, J. Gao, N. Rega, G. Zheng, W. Liang, M. Hada, M. Ehara, K. Toyota, R. Fukuda, J. Hasegawa, M. Ishida, T. Nakajima, Y. Honda, O. Kitao, H. Nakai, T. Vreven, K. Throssell, J. A. Montgomery, Jr., J. E. Peralta, F. Ogliaro, M. J. Bearpark, J. J. Heyd, E. N. Brothers, K. N. Kudin, V. N. Staroverov, T. A. Keith, R. Kobayashi, J. Normand, K. Raghavachari, A. P. Rendell, J. C. Burant, S. S. Iyengar, J. Tomasi, M. Cossi, J. M. Millam, M. Klene, C. Adamo, R. Cammi, J. W. Oahterski, R. L. Martin, K. Morokuma, O. Farkas, J. B. Foresman, and D. J. Fox, Gaussian, Inc., Wallingford CT, **2016**.

[S4] Lu, T.; Chen, F. Multiwfn: A Multifunctional Wavefunction Analyzer, *J. Comput. Chem.* **2012**, *33*, 580.

[S5] NBO 7.0. Glendening, E.D.; Badenhoop, J.K.; Reed, A. E.; Carpenter, J.E.; Bohmann, J.A. Morales, C.M.; Karafiloglou, P.; Landis, C.R.; Weinhold, F. Theoretical Chemistry Institute, University of Wisconsin, Madison, WI, **2018**.

[S6] http://iqmol.org/
